# Photoinduced Selective Construction of Densely Functionalized Spirocyclic Ethers With Carbyne Equivalents

**DOI:** 10.1002/anie.1489528

**Published:** 2026-03-18

**Authors:** Jianke Su, Chu Wang, Zhongping Cai, Jiale Wu, Xu Yan Chen, Jie Wu

**Affiliations:** ^1^ Department of Chemistry National University of Singapore Singapore; ^2^ State Key Laboratory of Precision and Intelligent Chemistry Anhui Provincial Key Laboratory of Biomass Chemistry Department of Chemistry University of Science and Technology of China Hefei Anhui China; ^3^ Hwa Chong Institution Singapore

**Keywords:** carbyne equivalents, photocatalysis, spirocyclic ethers, trivalent carbon synthon

## Abstract

Carbyne equivalents represent ideal trivalent carbon synthons, capable of forming C─C and C─X bonds through their three nonbonded electrons. This unique reactivity profile offers high modularity for rapid assemble complicate molecular scaffolds. However, the selective introduction of three σ‐bonds at a single carbon center remains a formidable challenge, owing to the multifaceted reactivity of the carbyne center. Herein, we report a cascade reaction, which sequentially regulates the radical, nucleophilic, and electrophilic reactivity of carbyne equivalents under light irradiation. This cascade enables precise spatial and temporal activation, facilitating the selective and efficient construction of densely functionalized spirocyclic ethers from allylic and homoallylic acetates, with three σ‐bonds introduced at the carbyne center. A wide range of spirocyclic ethers bearing diverse functional groups and structural motifs was obtained with excellent diastereoselectivity, underscoring the capability of carbyne equivalents for rapid construction of drug‐like molecules. The reaction was readily scalable up to 50 mmol, either in batch mode or using a high‐speed circulation‐flow reactor. Mechanistic studies, including UV–vis spectroscopy, cyclic voltammetry, radical trapping, structure‐reactivity relationship experiments, isotope labeling, as well as theoretical calculations, provide insights into the dynamic evolution of reactive intermediates, shedding light on the diverse reactivity of carbyne equivalents in organic synthesis.

## Graphical abstract

A reactivity programmed cascade between carbyne equivalents and allylic benzoates was developed that sequentially orchestrates all three reactivity modes of carbyne equivalents, enabling the rapid installation of three σ‐bonds at a single carbon center and providing efficient, scalable access to structurally diverse spirocyclic ethers.

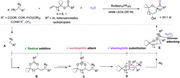

## 1 Introduction

Tetrasubstituted carbon centers are prevalent in natural products, pharmaceuticals, and functional materials, yet their efficient and selective construction remains a long‐standing challenge in organic synthesis [1]. Conventional strategies typically involve stepwise substitution or addition at a central carbon atom, approaches that, while reliable, often suffer from poor step economy and limited overall efficiency. In contrast, electron‐deficient carbon species such as carbenes [2] and carbynes [3, 4, 5] offer unique opportunities for rapid molecular assembly by enabling the formation of multiple bonds in a single transformation [6, 7]. Recent advances in the design of stable precursors and surrogates have made it possible to harness the high reactivity and short lifetime of carbenes under controlled conditions [8, 9, 10]. Carbyne equivalents, as ideal trivalent carbon synthons, theoretically allow the simultaneous formation of three new C─C or C─X bonds through their three nonbonded electrons, offering a powerful platform for complexity‐generating transformations.

**FIGURE 1:**
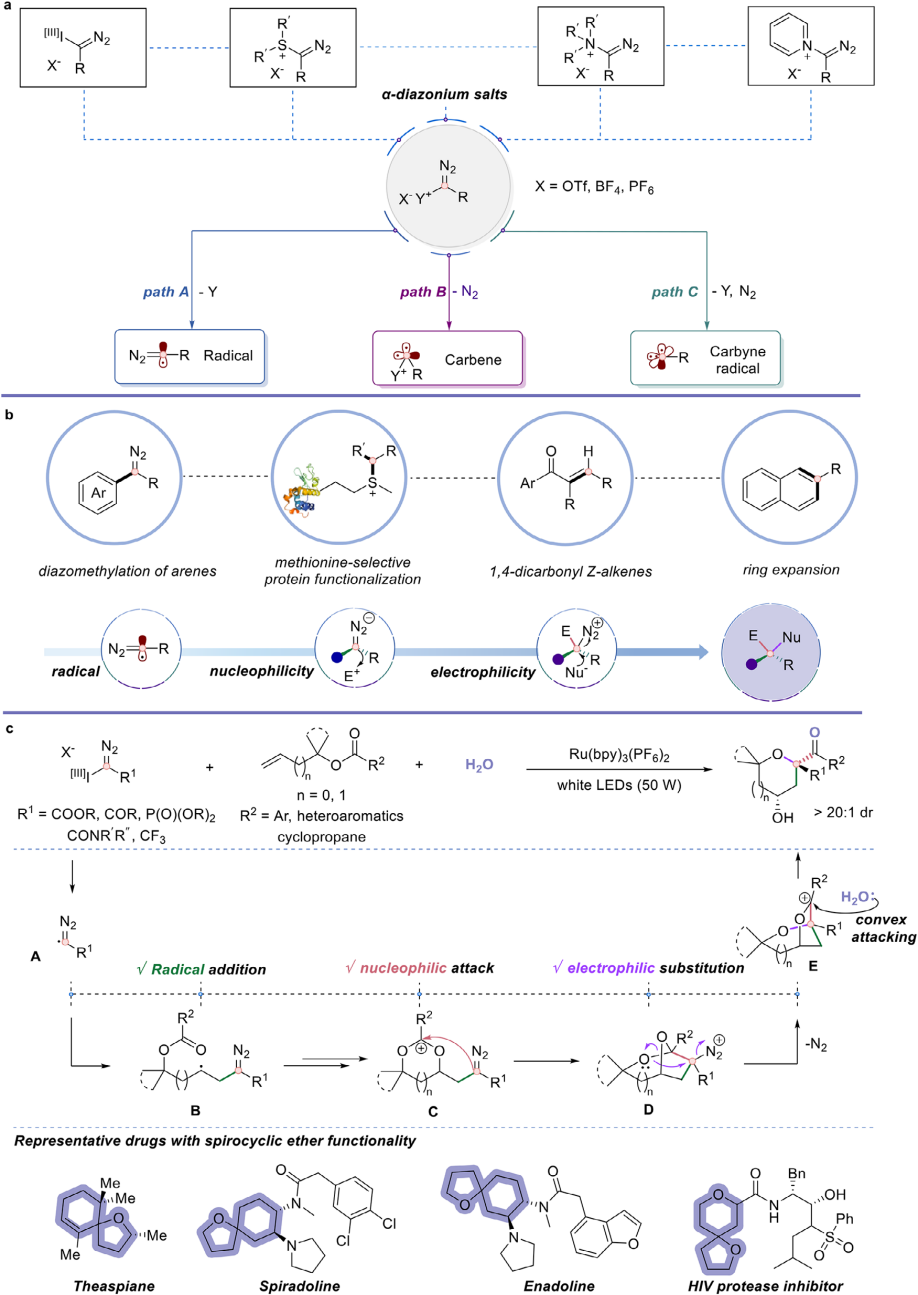
Reactions using carbyne equivalents. (a) Reagent profiles and reactivity characteristics of α‐diazonium salts. (b) Applications and characteristic reactivity of α‐diazonium salts. (c) Photoredox‐enabled carbyne‐equivalent‐induced cascade for modular synthesis of spirocyclic ethers.

Since the first preparation of a carbyne complex by Fischer in 1973 [11], significant progress has been made in the synthesis of carbyne equivalents and the modulation of their reactivity. These carbon intermediates have introduced transformative paradigms in carbon‐centered chemistry, complementing the traditional monovalent (carbocations, carbanions, radicals) and divalent (carbenes) carbon intermediates [9]. Among them, α‐diazonium salts (general formula [Y^+^C( = N_2_)RX^−^], where Y = I(III), R'_2_S, R'_3_N, or pyridinium, and X = OTf, BF_4_, PF_6_, Figure 1a), first reported by Weiss [12, 13], have been widely used as electrophilic reagents [13, 14, 15, 16, 17, 18]. Upon activation, carbyne equivalents can diverge into three distinct reactive species (Figure 1a): carbon radicals (path A), carbenes (path B), and carbyne radicals (path C). The convergence of multiple reactivity modes on a single carbon atom presents a challenge in achieving site‐ and sequence‐selective bond formation. Programming such multifaceted reactivity with spatial and temporal precision remains a critical bottleneck in the development of efficient and selective carbyne‐mediated transformations.

In recent years, the Suero group has made pioneering contributions by developing various efficient transformations using carbyne equivalents under transition‐metal catalysis [16, 17, 18, 19] and photocatalysis [20, 21, 22]. Meanwhile, the groups of Gaunt [23], Wang [24], Glorius [25, 26], Takashi Ooi [27], and others [28, 29, 30, 31, 32] have significantly expanded the synthetic utility of these intermediates, enabling applications in C─H functionalization, skeletal editing, protein functionalization, carbon unit transfer, and heterocycle synthesis (Figure 1b). Despite these advances, examples of fully exploiting carbyne equivalents for three σ‐bond formation in a single transformation remain scarce, and typically rely on sequential activation strategies rather than a single catalytic cascade approach.

Herein, we report a reactivity‐programmed cascade strategy that harnesses the multifaceted nature of carbyne equivalents under photoinduced single‐electron transfer (SET) conditions (Figure 1b). Upon photo‐promoted SET activation, the carbyne equivalent generates a radical species N_2_ = C(•)–R^1^, which undergoes addition to radical acceptors (such as alkenes, arenes, or imines) to afford diazo intermediate N_2_ = C–R^1^R^2^. When the resulting diazo compound contains an electron‐withdrawing group (e.g., carbonyl, ester, or sulfonyl) at the α‐position, the adjacent carbyne carbon center becomes electronically activated and functions as a nucleophile [33], enabling reacting with an electrophile to yield a R^1^R^2^R^3^C−N_2_^+^ intermediate. The strong polarization of the C─N bond, induced by the positively charged N_2_ unit, significantly lowers the lowest unoccupied molecular orbital (LUMO) energy level of the carbon center, imparting the potent electrophilic character [34, 35, 36]. This facilitates a subsequent nucleophilic attack, which triggers rapid nitrogen extrusion, offering both kinetic acceleration and thermodynamic favorability. Through this sequence, carbyne equivalents exhibit radical, nucleophilic, and electrophilic reactivity in a defined order, providing a mechanistic basis for stepwise bond formation at a single carbon center. By orchestrating these reactive modes with spatial and temporal precision, this strategy paves the way toward fully exploiting the synthetic potential of carbyne equivalents as trivalent carbon synthons for the construction of three new σ‐bonds in a single cascade.

Building on this tailored platform, we evaluated several types of alkenes and carbyne equivalents, ultimately discovering that the reaction of allylic benzoate substrates with carbyne equivalents under photoredox conditions afforded densely functionalized spirocyclic ethers with excellent diastereoselectivity (Figure 1c). Upon photoinduced SET, the carbyne equivalent releases radical species N_2_ = C(•)−R (**A**), which undergoes intermolecular addition to the alkene, forming intermediate **B**. The resulting radical is then oxidized to a carbocation, which undergoes intramolecular nucleophilic attack by the ester carbonyl oxygen, forming a carbocation intermediate **C**. Subsequent intramolecular nucleophilic attack affords the intermediate R_3_C–N_2_^+^ (**D**). This is followed by neighboring group‐assisted C─O bond cleavage and oxygen [1,2]‐shift, concomitant with N_2_ extrusion, generating a carbocation intermediate **E**. Finally, water captures **E** through a diastereoselective ring‐opening to furnish highly substituted spirocyclic ether products. Oxygen‐containing heterocycles are among the most frequently observed ring systems in FDA (U.S. Food and Drug Administration)‐approved therapeutics and are predominantly derived from commercially available pyranoses and furanoses [37, 38]. Notably, such spirocyclic ether scaffolds are prevalent in numerous bioactive molecules, highlighting the potential of our approach for direct access to drug‐like structures [39, 40]. This strategy enables a controlled interplay among the radical, nucleophilic, and electrophilic reactivities of carbyne equivalents, facilitating the sequential construction of three new σ‐bonds within a single reaction system, which not only significantly enhances molecular complexity but also demonstrates high synthetic efficiency.

## 2 Results and Discussion

**FIGURE 2:**
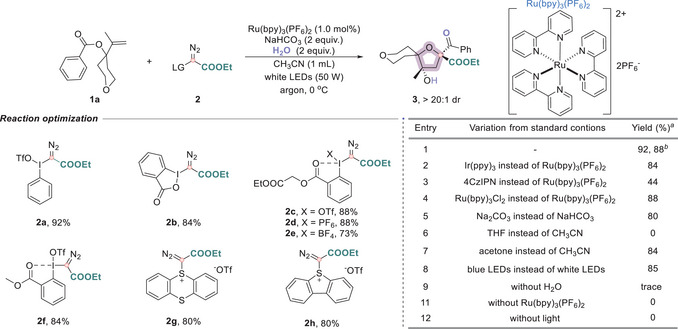
Optimization of the reaction conditions. Conditions: 1a (0.1 mmol), 2 (0.15 mmol), Ru(bpy)_3_(PF_6_)_2_ (1 mol%), NaHCO_3_ (0.2 mmol), and H_2_O (0.2 mmol) in CH_3_CN (0.1 M), irradiation with a 50 W white LED under an argon atmosphere at 0°C for 2 h. ^a^ Yields were determined by analysis of the crude ^1^H NMR spectra using CH_2_Br_2_ as an internal standard. ^b^ Isolated yields. The dr values were determined by ^1^H NMR analysis of the crude reaction mixtures. If not otherwise specified, the dr values of products were >20:1.

Following the discovery of spirocyclic ether formation, we investigated a range of bench‐stable and readily accessible carbyne precursors capable of generating α‐diazo radicals under photoredox conditions. Our evaluation employed 1,1‐disubstituted allylic benzoate (**1a**) as the model substrate, using Ru(bpy)_3_(PF_6_)_2_ as the photocatalyst, NaHCO_3_ as the base, and CH_3_CN as the solvent under white LED irradiation. A variety of carbyne equivalents were examined, including hypervalent iodine diazo compounds (**2a–2f**) and sulfonium triflates (**2g–2h**). Encouragingly, all tested reagents furnished the desired spirocyclic ether product **3**, with hypervalent iodonium diazo compound **2a** affording the highest efficiency, with an 88% isolated yield (Figure 2, entry 1). Subsequent investigation of photoredox catalysts revealed that replacing Ru(bpy)_3_(PF_6_)_2_ (*E*_red_* = −0.81 V, *E*ox* = +0.77 V vs. SCE) [41] with Ir(ppy)_3_ (*E*_red_* = −1.73 V, *E*ox* = +0.31 V vs. SCE) [41] resulted in a slightly diminished yield of 84% (entry 2), while the use of the organic photocatalyst 1,2,3,5‐tetrakis‐(carbazol‐9‐yl)‐4,6‐dicyanobenzene (4CzIPN) (*E*_red_* = −1.21 V, *E*ox* = +1.52 V vs. SCE) [41] significantly lowered the yield to 44% (entry 3). Notably, Ru(bpy)_3_Cl_2_ (*E*_red_* = −0.81 V, *E*ox* = +0.77 V vs. SCE) [41] proved to be a viable alternative to its PF_6_^−^ counterpart, delivering a comparable yield (entry 4). The choice of base influenced the reaction outcome, with Na_2_CO_3_ giving a modest decrease in yield (entry 5). Solvent effects were pronounced: no product formation was observed in THF (entry 6), and a moderate yield reduction was noted in acetone (entry 7). Replacing white LEDs with blue LEDs afforded a comparable yield (entry 8). Control experiments further confirmed the critical roles of water, photocatalyst, and light irradiation during the reaction (entries 9–12).

**FIGURE 3:**
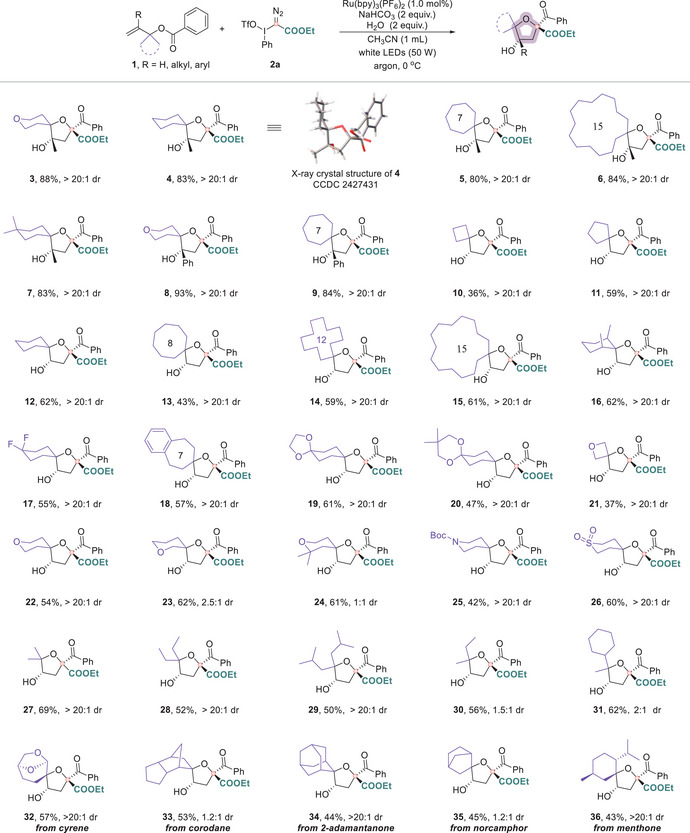
Substrate scope for the divergent synthesis of tetrahydrofuran. Reaction conditions: **1** (0.1 mmol), **2a** (0.15 mmol), Ru(bpy)_3_(PF_6_)_2_ (1 mol%), NaHCO_3_ (0.2 mmol), and H_2_O (0.2 mmol) in CH_3_CN (0.1 M), irradiation with a 50 W white LED under an argon atmosphere at 0°C for 2 h. The dr values were determined by ^1^H NMR analysis of the crude reaction mixtures. Isolated yields were reported.

With the optimized conditions in hand, we next examined the generality of the protocol by investigating a series of 1,1‐disubstituted allylic benzoates (Figure 3), beginning with the influence of substituents at the olefinic R‐group. When R = methyl or phenyl, the reaction proceeded smoothly, affording the corresponding spirocyclic ethers (**3–9**) in good yields. We then systematically explored a wide range of spirocyclic frameworks derived from allylic benzoates bearing rings of varying sizes. Remarkably, carbocyclic substrates ranging from four‐ to fifteen‐membered rings were efficiently transformed into spiro[n.4]oxanes (*n* = 3, 4, 5, 7, 11, 14; compounds **10–15**). To further explore the structural diversity of spirocyclic scaffolds, we introduced a variety of substituents, including methyl, gem‐difluoro, phenyl, and cyclic ketals, on the ring moiety, which were well tolerated to give a series of functionally enriched spirocyclic tetrahydrofurans (**16–20**). These findings highlight the method's robust compatibility with both steric and electronic variations within the cyclic backbone. Heteroatom‐embedded cyclic systems were next evaluated. The inclusion of an oxetane unit in the allylic benzoate led to the formation of 2,5‐dioxaspiro[3.4]octane (**21**) in moderate yield (37%). This reduced efficiency is attributed to the pronounced angle strain of the four‐membered ring, torsional strain from the fused tetrahydrofuran, and limited conformational flexibility—factors that collectively destabilize the “four‐on‐five” spirocyclic architecture and render it synthetically challenging [42, 43]. In contrast, tetrahydropyran‐based substrates, irrespective of substitution patterns, were smoothly converted into dioxaspiro[4.5]decanes (**22–24**) in better yields. The scope was further extended to include nitrogen‐ and sulfur‐containing rings, affording 1‐oxa‐8‐azaspiro[4.5]decane (**25**) and 1‐oxa‐8‐thiaspiro[4.5]decane 8,8‐dioxide (**26**), respectively. These spirocyclic frameworks, characterized by their rigid and preorganized three‐dimensional architectures, are frequently encountered in natural products, pharmaceuticals, and functional materials, thus highlighting the synthetic application potential of this methodology. Beyond cyclic motifs, we also evaluated acyclic allylic benzoates with both symmetrical and asymmetrical substitution patterns. These substrates were efficiently converted into densely functionalized tetrahydrofuran derivatives (**27–31**). Encouraged by the broad applicability, we extended the protocol to molecular frameworks resembling natural products, including cyrene and corodane, which underwent smooth spirocyclization, delivering architecturally complex spiroethers (**32**, **33**) in good yields. Furthermore, late‐stage spirocyclization of 2‐adamantanone, norcamphor, and menthone furnished the corresponding spirocyclic products (**34–36**), highlighting the method's applicability to structurally diverse substrates and its potential for late‐stage functionalization.

**FIGURE 4:**
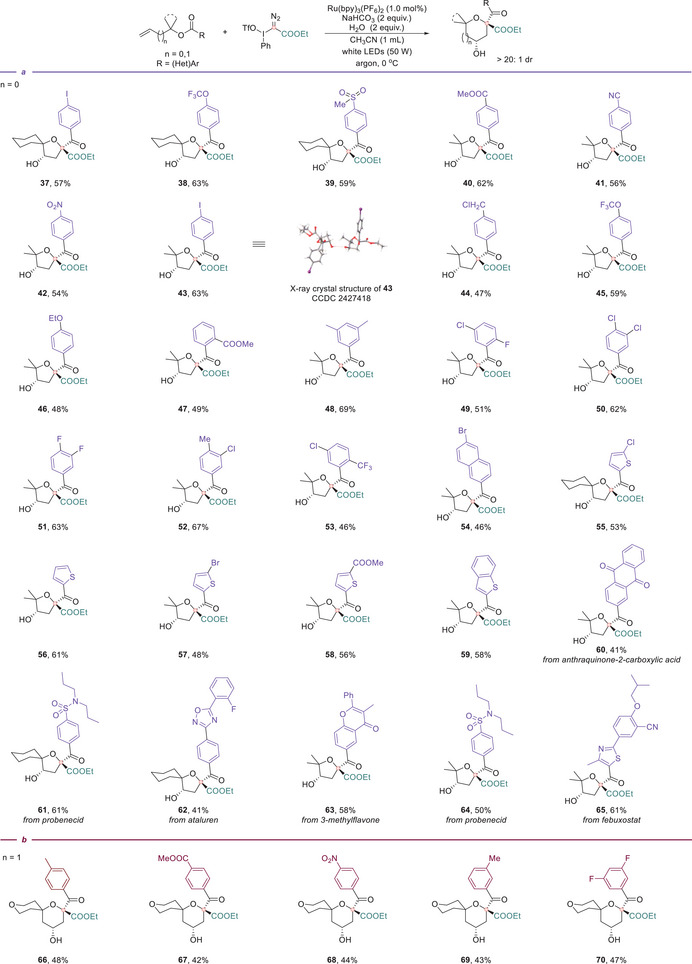
Substrate scope for the divergent synthesis of tetrahydrofurans or tetrahydropyrans. (a) Evaluation of various benzoates for tetrahydrofuran synthesis. (b) Evaluation of various benzoates for tetrahydropyran synthesis. Reaction conditions: **1** (0.1 mmol), **2a** (0.15 mmol), Ru(bpy)_3_(PF_6_)_2_ (1 mol%), NaHCO_3_ (0.2 mmol), and H_2_O (0.2 mmol) in CH_3_CN (0.1 M), irradiation with a 50 W white LED under an argon atmosphere at 0°C for 2 h. The dr values were determined by ^1^H NMR analysis of the crude reaction mixtures. Unless otherwise noted, product dr values are >20:1.

Subsequently, we evaluated the aryl group compatibility in the benzoate moiety of allylic benzoates (Figure 4a). When the allylic moiety contained a cyclohexyl group, para‐substituted aryl esters bearing different electron‐withdrawing groups, iodo (**37**), trifluoromethoxy (**38**), or methyl sulfone (**39**) were all successfully converted to the corresponding 1‐oxaspiro[4.5]decanes in 57%–63% yields. We further examined substituted aryl groups when the allylic unit was gem‐dimethyl substituted. A broad range of mono‐substituted arenes underwent smooth transformations, regardless of electronic nature, including electron‐withdrawing (‐COOMe, **40**; ‐CN, **41**; ‐NO_2_, **42**; ‐I, **43**; ‐CH_2_Cl, **44**; ‐OCF_3_, **45**), electron‐donating (‐OEt, **46**) substituted arenes. Sterically hindered *ortho*‐substituted esters all furnished the desired products in good yields (**47**). Moreover, disubstituted aromatic rings bearing combinations of methyl, halogen, or strongly electron‐withdrawing trifluoromethyl groups (**48**–**53**) were compatible with the transformation. To further extend the scope of the aryl substituents, fused ring and heteroaromatic systems were evaluated, including naphthyl (**54**) and various five‐membered heterocycles (**55**–**59**), which all underwent efficient conversion to the corresponding multi‐substituted tetrahydrofurans. Notably, we incorporated a series of biorelevant and pharmacologically active molecules into the spirocyclic ether framework to showcase its potential application. These included anthraquinone‐2‐carboxylic acid (**60**), a candidate for antibacterial and anticancer agents; probenecid (**61, 64**), a clinically used uricosuric agent for gout treatment; ataluren (**62**), a therapeutic for nonsense mutation readthrough in genetic diseases; 3‐methylflavone (**63**), investigated for anxiolytic properties; and febuxostat (**65**), a xanthine oxidase inhibitor for hyperuricemia. Lastly, we expanded the ring system by introducing one additional methylene unit into the allylic starting materials (Figure 4b), enabling a rapid and modular synthesis of tetrahydropyran‐fused spirocycles, constructing 1,9‐dioxaspiro[5.5]undecane cores (**66**–**70**). These motifs, with their rigid 3D conformation and dense functionality, are highly valuable in the discovery of pharmaceuticals and functional materials.

**FIGURE 5:**
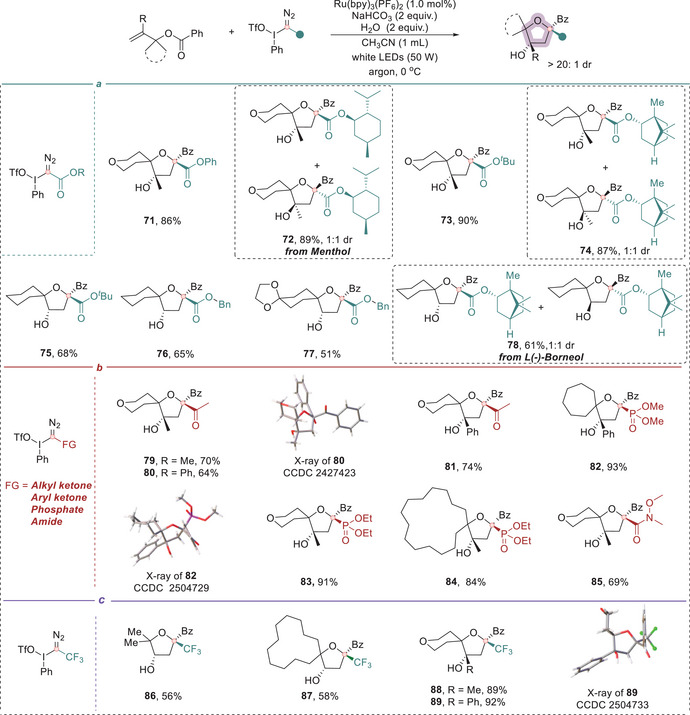
Scope of α‐iodonium diazo compounds for the divergent synthesis of densely functionalized tetrahydrofurans. (a) Synthesis of various 2‐COOR‐tetrahydrofurans. (b) Synthesis of tetrahydrofurans bearing an alkyl ketone, aryl ketone, phosphate, or amide moiety. (c) Synthesis of various 2‐CF_3_‐tetrahydrofurans. Reaction conditions: **1** (0.1 mmol), **2** (0.15 mmol), Ru(bpy)_3_(PF_6_)_2_ (1 mol%), NaHCO_3_ (0.2 mmol), and H_2_O (0.2 mmol) in CH_3_CN (0.1 M), irradiation with a 50 W white LED under an argon atmosphere at 0°C for 2 h. The dr values were determined by ^1^H NMR analysis of the crude reaction mixtures. If not otherwise noted, the dr values of products are >20:1.

To further expand the scope of this methodology, we systematically investigated the reactivity of a broad array of structurally and functionally diverse α‐iodonium diazo compounds (Figure 5). Remarkably, the transformation proved amenable to ester substrates derived from primary, secondary, and tertiary alcohols, as well as phenols (Figure 5a). These alcohols could be smoothly converted to the corresponding α‐iodo diazo precursors and subsequently participated in the cyclization cascade to afford functionalized tetrahydrofurans in good to high yields (**71**–**78**). Of particular interest was the successful incorporation of natural products such as menthol **(72)** and borneol **(78)**. Spirocyclic products bearing aliphatic ketones, aryl ketones, phosphates, and amides (**79**–**85**) were obtained in good to moderate yields (Figure 5b), further highlighting the robustness and versatility of this protocol. Notably, this methodology also proved applicable to trifluoromethylated substrates through the use of a hypervalent iodonium trifluoro diazo reagent as the carbyne equivalent, enabling the synthesis of 2‐trifluoromethyl‐tetrahydrofurans (**86**) and structurally diverse CF_3_‐containing spirocyclic ethers (**87–89**) in good to excellent yields (Figure 5c). CF_3_‐substituted compounds are of high interest across pharmaceuticals, agrochemicals, and materials science due to their lipophilicity, metabolic stability, and unique electronic properties [44]. The successful incorporation of trifluoromethyl substituents to spirocyclic and heterocyclic scaffolds provides a valuable synthetic entry point into this underexplored class of fluorinated molecules.

**FIGURE 6:**
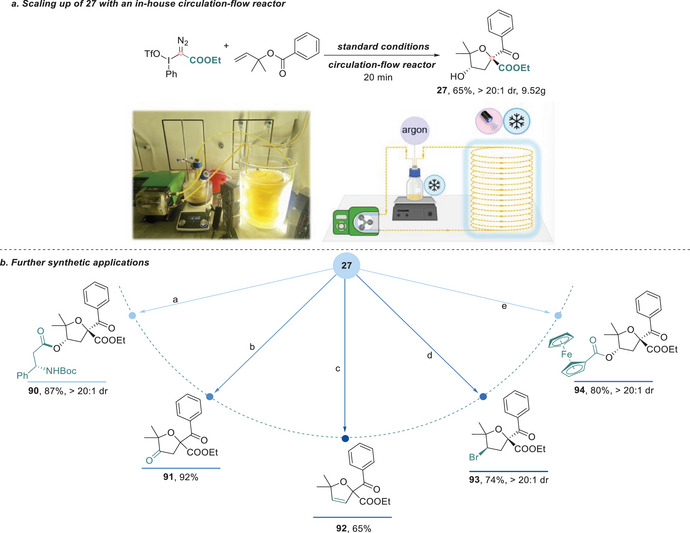
Synthetic applications. (a) Scaling up using a circulation‐flow reactor. **b**. Further diversification of product **27**.

To further demonstrate the synthetic utility of this strategy, we implemented a circulation‐flow platform developed in our laboratory to evaluate its scalability and efficiency [45]. Compared to traditional batch conditions, which required 1.5 h to achieve a good conversion, the transformation at a 50 mmol scale was completed within 20 min using the circulation‐flow platform, producing over 9 grams of the target product with an isolated yield comparable to that observed in small‐scale reactions (Figure 6). In addition, performing the reaction on a 50 mmol scale under batch conditions afforded the desired product in 53% yield, demonstrating that the protocol is amenable to scale‐up in both batch and flow modes. These results collectively highlight the excellent scalability and practical utility of this transformation. To demonstrate the usage of the synthesized ethers, we explored post‐functionalization of representative product **27**. The hydroxyl group on its backbone provides a versatile handle for derivatization. For instance, esterification with a β‐amino acid delivered compound **90**, while oxidation using Dess‐Martin periodinane afforded the corresponding ketone **91** in 92% yield. Under acidic conditions, dehydration gave access to the synthetically valuable 2,5‐dihydrofuran scaffold **92**. Moreover, Appel reaction with CBr_4_ yielded the corresponding alkyl bromide **93**, and acylation with ferrocenecarbonyl chloride enabled efficient introduction of the ferrocene motif via ester formation (**94**) (Figure 6). Protection of the hydroxyl group with TBSCl furnished the silyl ether derivative **95** in 61% yield. Additionally, the carbonyl moiety in the product could be converted into an oxime (**96**) via condensation with hydroxylamine hydrochloride (Figure S11). These diverse downstream transformations demonstrate the rich synthetic potential of the spirocyclic ether scaffold, not only as a structurally rigid and functionalized core but also as a highly valuable intermediate for further elaboration toward complex and bioactive molecular targets.

**FIGURE 7:**
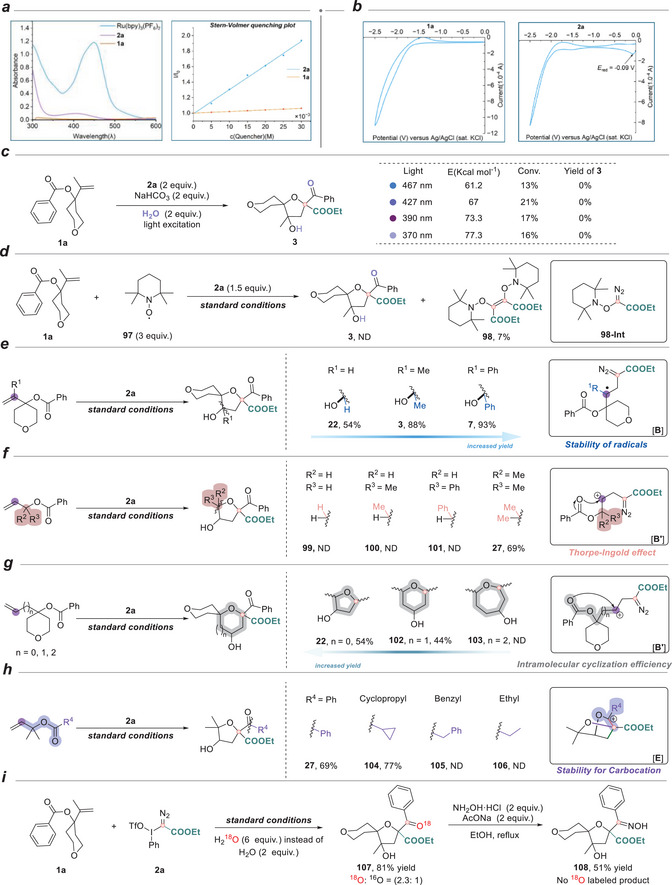
Control experiments and mechanistic studies. (a) The UV‐vis absorption spectrum and Stern–Volmer quenching studies. (b) Cyclic voltammetry measurements of **1a** and **2a**. (c) Infeasibility of direct photosensitization. (d) Radical scavenger experiments. (e–h) Structure‐reactivity relationship experiments. (i) ^18^O labelling experiments.

After developing a suitable method for densely functionalized spirocyclic ethers via the carbyne‐mediated cascade, subsequent efforts were directed towards exploring the reaction mechanism (Figure 7). UV–vis absorption spectroscopy indicated that both the photocatalyst Ru(bpy)_3_(PF_6_)_2_ and reagent **2a** were photoactive within the emission range of white LED light sources (Figure 7a). Stern‐Volmer analysis demonstrated that Ru(bpy)_3_(PF_6_)_2_ luminescence could be quenched by **2a** but not by the alkene, suggesting a specific interaction between the photocatalyst and the diazo compound under the reaction conditions. Cyclic voltammetry measurements revealed that **2a** (*E*_red_(**2a**/**2a^−^**) = −0.09 V versus SCE in CH_3_CN) could undergo a single‐electron transfer with the excited‐state Ru(bpy)_3_(PF_6_)_2_ (*E*_1/2_(Ru^III/II*^) = −0.81 V) [46] (Figure 7b). When the reaction mixture was irradiated at higher‐energy wavelengths (e.g., λ_max_ = 467, 427, 390, and 370 nm) in the absence of Ru(bpy)_3_(PF_6_)_2_, no product was detected except iodobenzene (Figure 7c). These results excluded the possibility of direct photoexcitation of **2a**. Radical trapping experiments using 2,2,6,6‐tetramethylpiperidin‐1‐oxyl (TEMPO) provided evidence for the generation of the radical intermediate N_2_ = C(•)−COOEt after the single‐electron transfer, as indicated by inhibition of product formation and the initial formation of a TEMPO‐trapped diazo adduct (**98‐Int**), which subsequently underwent photoinduced N_2_ extrusion and dimerization to afford the dimeric product (**98**) in 7% isolated yield (Figure 7d).

Following these preliminary mechanistic investigations, additional control experiments were performed to support the sequential activation of the radical, nucleophilic, and electrophilic properties of carbyne equivalents, as well as the involvement of relevant intermediates, as proposed in our design (Figure 1c). By varying the substituent (R^1^) on the alkene motif of the allylic benzoate, we observed differences in product yield—hydrogen (54%), methyl (88%), and phenyl (93%). This variation was attributed to the enhanced stability of radical intermediate **B** with different substituents, supporting the participation of this radical species in the reaction pathway (Figure 7e). Further studies on the cyclization step were conducted by varying the substituents (R^2^ and R^3^) and the carbon chain length at the allylic position (Figures 7f,g). When either R^2^ or R^3^ was hydrogen, no desired product was detected, regardless of the identity of the other group (hydrogen, methyl, or phenyl), and no by‐products were observed in the reaction mixture other than benzoic acid, which arose from the decomposition of the allylic benzoates. In contrast, the desired product was successfully obtained when both were methyl groups. This observation could be rationalized by the Thorpe–Ingold (gem‐dimethyl) effect [47], where the disubstitution‐induced conformational preorganization facilitates intramolecular nucleophilic attack of the carbonyl oxygen to the carbocation center, favoring the ring‐closing transition state. Additionally, increasing the number of methylene units in the allylic chain led to a gradual decrease in yield (*n* = 0, 62%; *n* = 1, 44%), with no product observed when the chain contained two or more methylenes. This trend could be attributed to enthalpic and entropic factors associated with ring formation. Five‐ and six‐membered rings experienced minimal ring strain, enabling effective cyclization. In contrast, the formation of larger rings was hampered by increased conformational flexibility and a higher entropic penalty [48, 49]. Collectively, these results strongly implicate the generation and involvement of the carbocation intermediate **B'** and the corresponding cyclized intermediate during the reaction process. Next, modification of the ester substituent (R^4^) (Figure 7h) revealed that a phenyl group gave the desired product in 69% yield, while cyclopropyl substitution afforded product **104** in 77% yield. However, benzyl and ethyl substituents failed to deliver the product. This outcome could be explained by the ability of the phenyl group to stabilize the carbocation intermediate **E** through p–π conjugation, and the cyclopropyl group to provide hyperconjugative stabilization via its bent “banana” bonds [50, 51].

Finally, we used an isotope labeling experiment to determine which oxygen atom in the product originated from the external source. When the reaction of **1a** and **2a** was performed using H_2_^18^O instead of H_2_O, product **107** was obtained, and high‐resolution mass spectrometry (HRMS) analysis showed that the ^18^O to ^16^O ratio in the product was approximately 2.3:1 (Figure 7i). Conversion of **107** into an oxime **108** using hydroxylamine hydrochloride led to the complete loss of the ^18^O label, suggesting that the oxygen in the carbonyl group originated from water. These findings provide compelling evidence for the formation and mechanistic relevance of the carbocation intermediate **E**.

**FIGURE 8:**
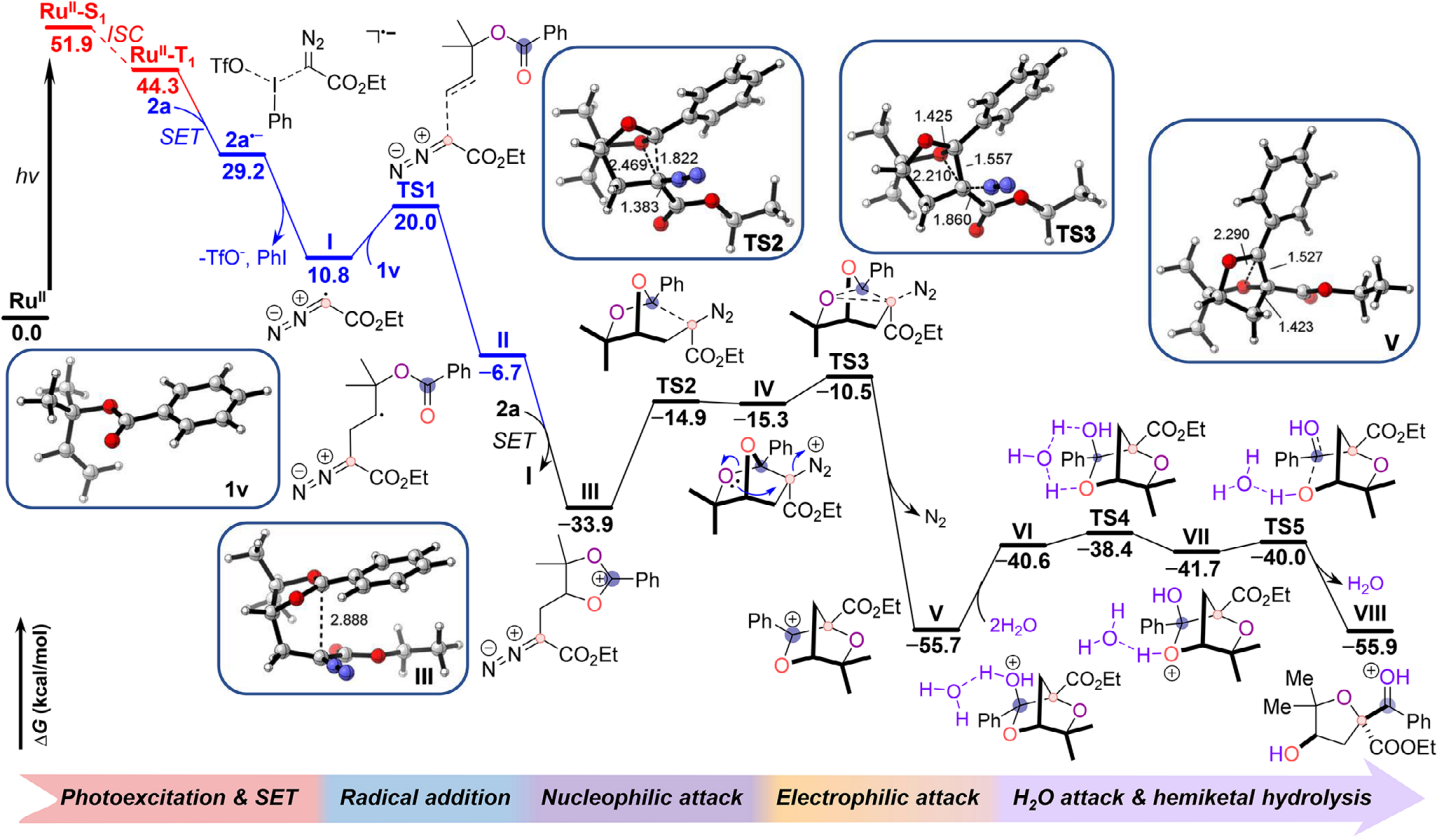
Computational studies and plausible mechanisms. Calculated energy profiles for the photoredox catalytic reaction of the allylic benzoate substrate **1v** with hypervalent iodonium diazo compound **2a**, yielding the densely functionalized ether **27**, at the B3LYP‐D3/def2‐TZVPP//def2‐SVP/PCM(acetonitrile) level of theory.

Drawing on our mechanistic design and experimental observations, we employed density functional theory (DFT) calculations to elucidate the complete energy profiles of this carbyne‐mediated photoredox catalytic reaction (Figure 8). Upon light irradiation, the photocatalyst Ru(bpy)_3_(PF_6_)_2_ (**Ru^II^**) is prompted to its excited singlet state (**Ru^II–^S_1_**), which then relaxes to the triplet state (**Ru^II–^T_1_**). Hypervalent iodine diazo compound (**2a**) is reduced by **Ru^II–^T_1_** to generate the radical intermediate (**I**).

The resulting radical **I** then adds to the allylic benzoate **1v** to afford radical intermediate (**II**), overcoming an energy barrier of around 9.2 kcal/mol (**TS1**). Computational studies indicate that intermediate **II** can be oxidized by **2a** with a driving force of approximately 27.2 kcal/mol to produce the carbocation intermediate **III**, while simultaneously regenerating radical **I** and thereby initiating a radical propagation process. This mechanistic scenario is further supported by a high quantum yield (*φ* =  9.8) (see Supplementary Information), which is indicative of a radical chain mechanism. Alternatively, for monosubstituted allylic benzoates, intermediate **II** can also undergo a [1,2]‐benzoyloxy radical migration to generate a more stable tertiary carbon radical intermediate (**IX**), which may then be oxidized to yield intermediate **III** (Figure S7) [50, 52]. Subsequently, the carbocation **III** is intramolecularly attacked by the carbyne carbon center, surmounting an activation barrier of around 19.0 kcal/mol to yield a meta‐stable bridged bicyclic intermediate **IV**. This intermediate then engages in a concerted process involving neighboring‐group participation and C─O bond heterolysis, effecting a formal oxygen [1,2]‐shift that is energetically favored by a thermodynamic driving force of roughly 40.4 kcal/mol. After crossing a three‐membered transition state **TS3** (barrier: ∼4.8 kcal/mol), intermediate **IV** is converted into a stable intermediate **V** with concomitant extrusion of molecular nitrogen. The carbyne fragment functions as an electrophile during this transformation. Finally, water acts as a nucleophile, attacking the carbocation of intermediate **V** to generate the hemiacetal intermediate **VII**, assisted by a second water molecule. This intermediate then undergoes hydrolytic ring opening to yield intermediate **VIII**. Subsequent deprotonation affords the stable, densely functionalized ether product (**27**). In this transformation, the tetrahydrofuran ring‐opening event proceeds within a conformationally constrained bridged‐ring structure, which restricts the trajectory of the nucleophilic water attack. The ring strain and geometric rigidity enforce a convex nucleophilic attack that occurs in a concerted fashion with the cleavage of the C─O bond, resulting in the hydroxyl and carbonyl groups being positioned on the same face. This spatial control leads to excellent diastereoselectivity in the final spirocyclic ether products. Notably, our computational results are highly consistent with the outcomes of the control experiments. For example, the calculated thermodynamic stabilities of radical intermediate **B** for compounds **22**, **3**, and **7** increase progressively (Figures 7e and S8). Similarly, the thermodynamic stability of intermediate **E** for **27** and **104** is significantly higher than that of the corresponding intermediate **E** for **106** (Figures 7h and S9).

## 3 Conclusion

In summary, we have developed a multifaceted reactivity programming of carbyne equivalents with spatial and temporal control, enabling the construction of spirocyclic ethers bearing a wide range of functional groups and structural motifs in a highly selective manner. Initiated by photoinduced SET processes, this cascade reaction sequentially triggers the radical, nucleophilic, and electrophilic reactivities of carbyne equivalents, facilitating the formation of three σ‐bonds at the carbyne carbon center and rapidly increasing molecular complexity. Control experiments and DFT calculations further validated the formation and transformation of key intermediates, providing strong mechanistic support for the proposed reaction pathway. Overall, this work not only expands the reactivity landscape of carbyne equivalents as versatile trivalent carbon synthons but also establishes a controlled strategy for harnessing complex reactive species, laying a foundation for the rapid assembly of drug‐like molecules.

## Conflicts of Interest

The authors declare no conflict of interest.

## Supporting information




**Supporting File 1**: anie71868‐sup‐0001‐SuppMat.pdf.

## Data Availability

The data that support the findings of this study are available in the Supporting Information of this article.
