# Supplementary material for: Photoinduced Selective Construction of Densely Functionalized Spirocyclic Ethers With Carbyne Equivalents
Source: Angew Chem Int Ed Engl. 2026 Mar 18;65(18):e1489528. doi: 10.1002/anie.1489528 (PMC13110766; doi:10.1002/anie.1489528)

Supporting Information

©Wiley-VCH 2025

69451 Weinheim, Germany

Photoinduced Selective Construction of Densely Functionalized Spirocyclic Ethers with Carbyne Equivalents

Jianke Su[1,4], Chu Wang[1,4], Zhongping Cai[1,2], Jiale Wu[1], Xu Yan Chen[3], Jie Wu[1]*

[1]Department of Chemistry, National University of Singapore, 3 Science Drive 3, Singapore, 117543.

[2]State Key Laboratory of Precision and Intelligent Chemistry, Anhui Provincial Key Laboratory of Biomass Chemistry, Department of Chemistry, University of Science and Technology of China; Hefei, Anhui 230026, China.

[3]Hwa Chong Institution, 661 Bukit Timah Road, Singapore 269734

[4]These authors contributed equally: Jianke Su, Chu Wang

*Corresponding author. Email: chmjie@nus.edu.sg (J.W.)

SUPPORTING INFORMATION

Table of Contents

1. Supporting Methods 3

1.1 General information........ 3

1.2 General process 3

2. Reaction Development 7

2.1 Optimization of the reaction conditions 7

3. Control Experiments and Mechanistic Studies........ 12

3.1 UV-vis absorption spectra........ 12

3.2 Stern-Volmer quenching studies........ 12

3.3 Cyclic voltammetry studies 13

3.4 Radical capture experiments........ 14

3.5 ^{18}O labelling experiments 14

3.6 allylic amine derivatives instead of allylic benzoates........ 16

3.7 Determination of quantum yields........ 16

3.8 DFT calculations 18

4. Synthetic Applications 71

4.1 Scaling up **27** with a circulation-flow reactor 71

4.2 Further synthetic applications........ 72

5. Crystal Data 75

6. Characterization Data for Products........ 80

7. Supporting References........ 156

8. NMR Spectroscopic Data 158

SUPPORTING INFORMATION

1. Supporting Methods

1.1 General information

^{1}H NMR, ^{13}C NMR and ^{19}F NMR spectra were recorded on a Bruker AV-III400 (400 MHZ), AMX500 (500 MHz) or AMX600 (600 MHz) spectrometer. Chemical shifts were reported in parts per million (ppm) and calibrated using residual undeuterated solvent as an internal reference ($CDCl_3$: 7.26 ppm ^{1}H NMR, 77.0 ppm ^{13}C NMR). Multiplicity was indicated as follows: s (singlet), d (doublet), t (triplet), q (quartet), m (multiplet), dd (doublet of doublet). All high-resolution mass spectra (HRMS) were obtained on a Finnigan/MAT 95XL-T spectrometer. Gas chromatography-mass spectrometry (GC-MS) was performed on Aglilent 7820A with FID detection. Flash column chromatography were performed on Merck 60 (0.040-0.063 mm) mesh silica gel and run under positive air pressure. Analytical thin layer chromatography (TLC) was performed with Merck pre-coated TLC plates (silica gel 60F-254, layer thickness 0.25 mm). Visualization was achieved by short wavelength (254 nm) ultraviolet light or by staining with potassium permanganate ($KMnO_4$).

1.2 General process

General Procedure A (for the synthesis of 1,1-disubstituted allylic benzoates):

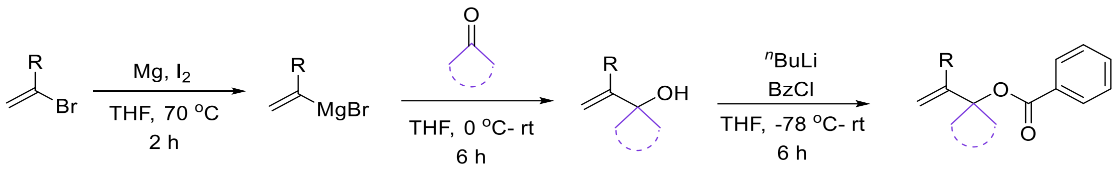

Step 1:

A dried three-necked flask equipped with a reflux condenser was charged with magnesium turnings (2.92 g, 120 mmol, 1.2 equiv.). A small crystal of iodine was added and treated with the heat gun until the iodine vapors were evenly distributed inside the flask. A solution of corresponding bromopropene (13.5 g, 100 mmol, 1.0 equiv.) in THF (100 mL) was added first portion-wise until the formation of a grey slurry and then drop-wise trough a dropping funnel over the course of 30 min, so that the reaction mixture was continuously boiling. The dark reaction mixture was cooled down to room temperature. The material was used without further purification in the next step.

Step 2:

The appropriate ketone (1.0 equiv.) and THF were added to a round-bottom flask and cooled to 0 °C under a positive pressure of nitrogen. Vinylmagnesium bromide (1.2 equiv.) was then added. This solution was stirred at 0 °C for 30 min and then warmed to room temperature and stirred for an additional 6 h. Then the reaction mixture was quenched by sat. NH_4Cl and extracted with EtOAc (three times). The combined organic phases were dried over anhydrous Na_2SO_4, filtered, and

concentrated under vacuum. The residue was purified by flash column chromatography with hexane/EtOAc.

Step 3:

The appropriate alcoholic (1.0 equiv.) and THF were added to a round-bottom flask and cooled to -78 °C under a positive pressure of nitrogen. ^{n}BuLi (1.2 equiv.) was then added. This solution was stirred at 0 °C for 30 min and then added benzoyl chloride (1.2 equiv.). This solution was stirred at room temperature for an additional 12 h. Then the reaction mixture was quenched by sat. NH_4Cl and extracted with EtOAc (three times). The combined organic phases were dried over anhydrous Na_2SO_4, filtered, and concentrated under vacuum. The crude reaction mixtures were purified by flash column chromatography using neutral alumina as the stationary phase (eluting with hexanes:EtOAc) to yield 1,1-disubstituted allylic benzoates.

General Procedure B (for the synthesis of monosubstituted allylic benzoates)[1]:

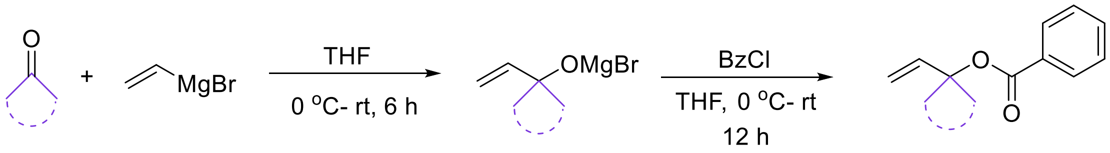

The appropriate ketone (1.0 equiv.) and THF were added to a round-bottom flask and cooled to 0 °C under a positive pressure of nitrogen. Vinylmagnesium bromide (1.2 equiv. as 1.00 M solution in THF) was then added. This solution was stirred at 0 °C for 30 min and then warmed to room temperature and stirred for an additional 6 h. The reaction mixture was cooled to 0 °C, then, benzoyl chloride (1.4 equiv.) was added. This solution was stirred at 0 °C for 30 min and then warmed to room temperature and stirred for an additional 12 h. The crude reaction mixture was quenched with 1 M HCl. The organic layer was separated and washed with saturated aqueous $NaHCO_3$ and brine, then dried over $MgSO_4$, filtered, and concentrated. The crude reaction mixtures were purified by flash column chromatography using neutral alumina as the stationary phase (eluting with hexanes:EtOAc) to yield allylic benzoates.

General Procedure C (for the synthesis of 2-methylbut-3-en-2-yl esters)[2]:

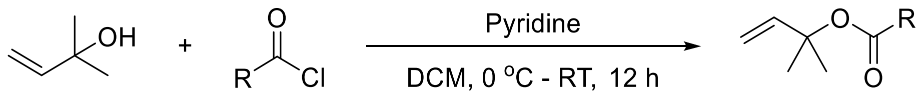

The 2-methylbut-3-en-2-yl esters were synthesized according to the literature procedure. To a solution of 2 methylbut-3-en-2-ol (860 mg, 10.0 mmol, 1.0 equiv.) in DCM (1.00 M, 10.0 mL) added pyridine (1.60 mL, 20.0 mmol, 2.0 equiv.) and benzoyl chloride (12.0 mmol, 1.2 equiv) at

0 °C. The reaction mixture stirred at room temperature for 12 h, then quenched with saturated $NaHCO_3$ solution (20.0 mL) and extracted with DCM (three times). The organic layer was collected, washed with brine, dried with anhydrous $MgSO_4$ and filtered, and concentrated. The crude reaction mixtures were purified by flash column chromatography using neutral alumina as the stationary phase (eluting with hexanes:EtOAc) to yield allylic benzoates.

General Procedure D (for the synthesis of homoallylic benzoate):

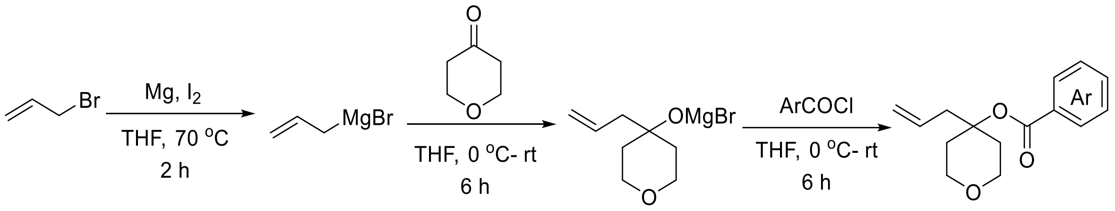

Step 1:

A dried three-necked flask equipped with a reflux condenser was charged with magnesium turnings (2.92 g, 120 mmol, 1.2 equiv.). A small crystal of iodine was added and treated with the heat gun until the iodine vapors were evenly distributed inside the flask. A solution of corresponding bromopropene (13.5 g, 100 mmol, 1.0 equiv.) in THF (100 mL) was added first portion-wise until the formation of a grey slurry and then drop-wise trough a dropping funnel over the course of 30 min, so that the reaction mixture was continuously boiling. The dark reaction mixture was cooled down to room temperature. The material was used without further purification in the next step.

Step 2:

The appropriate ketone (1.0 equiv) and THF were added to a round-bottom flask and cooled to 0 °C under a positive pressure of nitrogen. Allylmagnesium bromide (1.2 equiv) was then added. This solution was stirred at 0 °C for 30 min and then warmed to room temperature and stirred for an additional 6 h. This solution was stirred at 0 °C for 30 min and then added benzoyl chloride (1.2 equiv.). This solution was stirred at room temperature for an additional 12 h. The crude reaction mixture was quenched with 1 M HCl. The organic layer was separated and washed with saturated aqueous $NaHCO_3$ and brine, then dried over $MgSO_4$, filtered, and concentrated. The crude reaction mixtures were purified by flash column chromatography using neutral alumina as the stationary phase (eluting with hexanes:EtOAc) to yield homoallylic benzoates.

General Procedure E (for the synthesis of hypervalent iodonium diazo compounds)[3]:

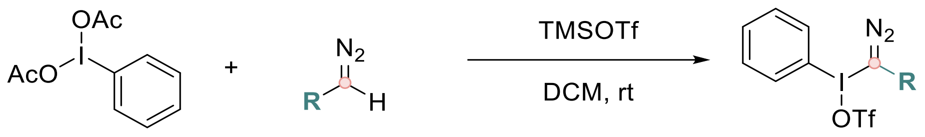

In an oven-dried 50 mL Schlenk tube equipped with a PTFE-coated oval stirring bar, phenyliodoso diacetate (1.6 g, 5 mmol, 1.0 equiv.) were charged under air, then the vessel was evacuated and re-filled with argon for three times. Dichloromethane (20 mL, 0.5 M) was treated with trimethylsilyl trifluoromethanesulfonate (0.9 mL, 5 mmol, 1.0 equiv.) in one-pot at room temperature under argon atmosphere. Then the corresponding diazoacetate (11 mmol, 2.2 equiv.) was added dropwise for 10 minutes. The resulting reaction mixture was stirred at room temperature for 2 hours. Solvent was removed under vacuum and the crude was recrystallized from a mixture of diethyl ether/dichloromethane (5/1) during 12 hours at -30 °C. The desired product was collected by filtration washed with cold diethyl ether (100 mL), dried under high vacuum and stored a -30 °C.

General Procedure F (for the synthesis of spirocyclic ethers or tetrahydrofuran):

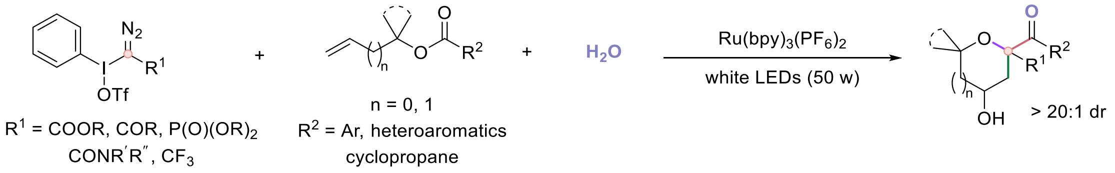

In an oven-dried 8 mL Schlenk tube equipped with a PTFE-coated oval stirring bar, $Ru(bpy)_3(PF_6)_2$ (0.001 mmol, 0.01 equiv.), hypervalent iodine reagents **2** (0.15 mmol, 1.5 equiv.) and $NaHCO_3$ (0.2 mmol, 2.0 equiv.) were charged under air, then the vessel was evacuated and re-filled with argon for three times. Acetonitrile (1.0 mL, 0.1 M), allylic benzoate **1** (0.1 mmol, 1.0 equiv.) and H_2O (0.2 mmol, 2.0 equiv.) were added under argon atmosphere. The vessel was sealed with the screw cap, then irradiated with white LEDs (50 W) at 0 °C for 2 hours. After irradiation, the crude reaction mixtures were purified by flash column chromatography to give the desired products.

SUPPORTING INFORMATION

2. Reaction Development

2.1 Optimization of the reaction conditions

Table S1. Screening of photocatalysts.

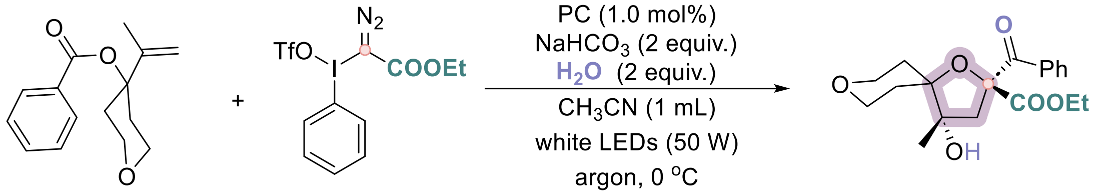

entries	PC	yield (%)[a]
1	4CzIPN	44
2	$Ir(ppy)_3$	84
3	$Ir[dF(CF_3)ppy]_2(dtbbpy)PF_6$	trace
4	$Mes\text{-}Acr^+BF_4^-$	ND
5	$Mes\text{-}Acr^+Cl_4^-$	ND
6	$Ru(bpy)_3Cl_2{\cdot}6H_2O$	88
7	$Ru(bpy)_3(PF_6)_2$	92, 88[b]
8	$[Ru(bpz)_3][PF_6]_2$	56
9	$Ru(dpp)_3(PF_6)_2$	84

Conditions: **1a** (0.1 mmol), **2a** (0.15 mmol), PC (1 mol%) and $NaHCO_3$ (0.2 mmol) in CH_3CN (0.1 M), irradiation with a 50 W white LEDs under an argon atmosphere at 0 °C for 2 h. [a] Yields were determined by analysis of the crude ^1H NMR spectra using CH_2Br_2 as an internal standard. [b] Isolated yields. The dr values were determined by ^1H NMR analysis of the crude reaction mixtures. If not otherwise specified, the dr values of products were >20:1.

Table S2. Screening of bases.

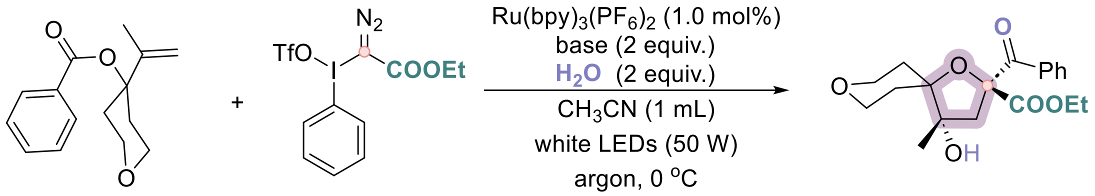

entries	PC	yield (%)[a]
1	Na_2CO_3	80
2	$NaHCO_3$	92
3	K_2CO_3	24
4	DMAP	ND
5	K_3PO_4	56
6	NaOAc	trace
7	Li_2CO_3	36
8	imidazole	ND
9	DIPEA	ND
10	Et_3N	ND

Conditions: **1a** (0.1 mmol), **2a** (0.15 mmol), $Ru(bpy)_3(PF_6)_2$ (1 mol%) and base (0.2 mmol) in CH_3CN (0.1 M), irradiation with a 50 W white LEDs under an argon atmosphere at 0 °C for 2 h. [a] Yields were determined by analysis of the crude ^1H NMR spectra using CH_2Br_2 as an internal standard. If not otherwise specified, the dr values of products were >20:1.

Table S3. Screening of solvents.

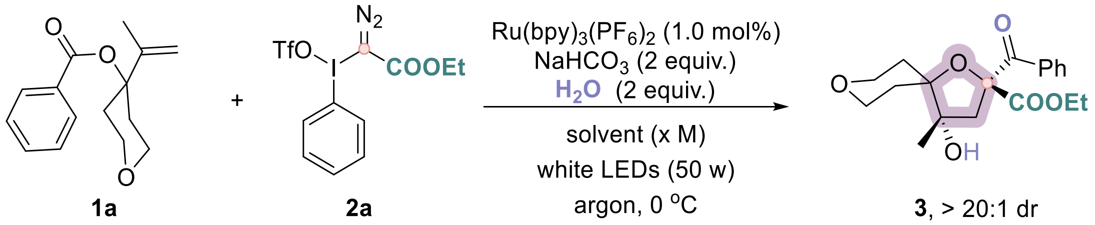

entries	solvent	yield (%)^{a}
1	THF (0.1 M)	trace
2	DCM (0.1 M)	ND
3	acetone (0.1 M)	84
4	HFIP (0.1 M)	ND
5	EtOAc (0.1 M)	64
6	CH_3CN (0.1 M)	92
7	CH_3CN (0.05 M)	90
8	CH_3CN (0.2 M)	91

Conditions: **1a** (0.1 mmol), **2a** (0.15 mmol), $Ru(bpy)_3(PF_6)_2$ (1 mol%) and $NaHCO_3$ (0.2 mmol) in solvent (x M), irradiation with a 50 W white LEDs under an argon atmosphere at 0 °C for 2 h. ^{a} Yields were determined by analysis of the crude ^{1}H NMR spectra using CH_2Br_2 as an internal standard. If not otherwise specified, the dr values of products were >20:1.

Table S4. Screening of carbyne equivalents.

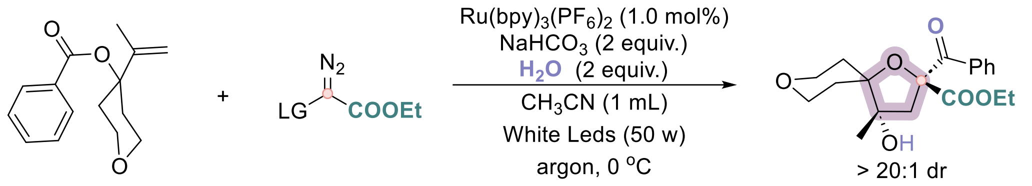

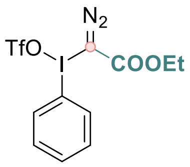

2a

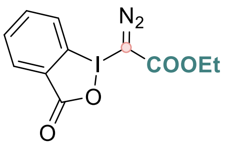

2b

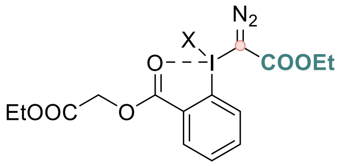

2c, X = OTf
2d, X = PF_6
2e, X = BF_4

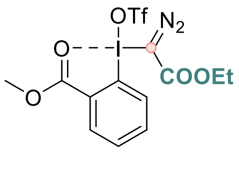

2f

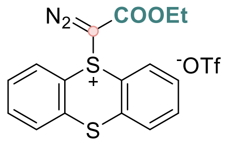

2g

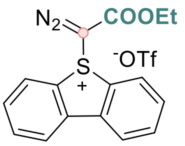

2h

entries	carbyne equivalents	yield (%)^a
1	**2a**	92
2	**2b**	84
3	**2c**	88
4	**2d**	88
5	**2e**	73
6	**2f**	84
7	**2g**	80
8	**2h**	80

Conditions: **1** (0.1 mmol), **2** (0.15 mmol), $Ru(bpy)_3(PF_6)_2$ (1 mol%) and $NaHCO_3$ (0.2 mmol) in CH_3CN (0.1 M), irradiation with a 50 W white LEDs under an argon atmosphere at 0 °C for 2 h. ^{a} Yields were determined by analysis of the crude ^1H NMR spectra using CH_2Br_2 as an internal standard. If not otherwise specified, the dr values of products were >20:1.

Table S5. Screening of temperature.

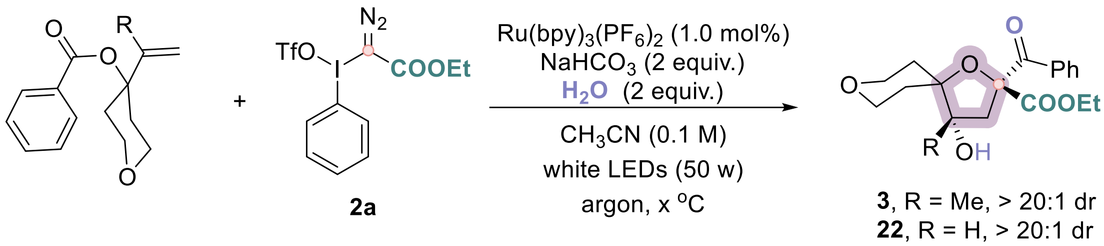

3, R = Me, > 20:1 dr
22, R = H, > 20:1 dr

entries	Temperature (°C)	Yield of 3 (%)^a	Yield of 22 (%)^a
1	25	85	38
2	10	87	44
3	0	92	54
4	-10	92	56
5	-20	93	55

Conditions: **1** (0.1 mmol), **2a** (0.15 mmol), Ru(bpy)$_3$(PF$_6$)$_2$ (1 mol%) and NaHCO$_3$ (0.2 mmol) in CH$_3$CN (0.1 M), irradiation with a 50 W white LEDs under an argon atmosphere at xx °C for 2 h. ^{a} Yields were determined by analysis of the crude ^{1}H NMR spectra using CH$_2$Br$_2$ as an internal standard. If not otherwise specified, the dr values of products were >20:1.

3. Control Experiments and Mechanistic Studies

3.1 UV-vis absorption spectra

As outlined in Figure S1, the ultraviolet-visible absorption spectrum of **1a** (1×10^{-3} mol l^{-1}), **2a** (1×10^{-3} mol l^{-1}) and $Ru(bpy)_3(PF_6)_2$ (1×10^{-5} mol l^{-1}) in MeCN was collected, respectively. Accordingly, the photocatalyst $Ru(bpy)_3(PF_6)_2$ and **2a** were found to be absorbing species near the excitation wavelength. Ultraviolet-visible absorption spectroscopy first indicated that both the photocatalyst $Ru(bpy)_3(PF_6)_2$ and reagent **2a** are photoactive within the emission range of white LED light sources.

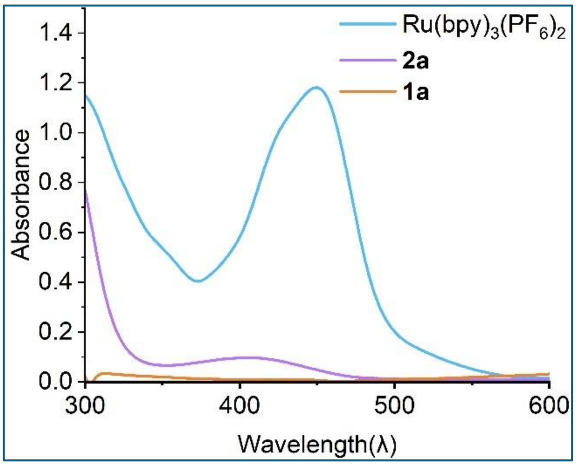

Figure S1. UV-vis absorption spectra of **1a**, **2a**, and $Ru(bpy)_3(PF_6)_2$ in MeCN

3.2 Stern-Volmer quenching studies

The luminescence quenching experiment was taken using a F-7000 FL Spectrophotometer (Hitachi, Japan). The experiments were carried out in 1×10^{-4} mol/L of $Ru(bpy)_3(PF_6)_2$ in CH_3CN at 25 °C. The excitation wavelength was 500 nm and the emission intensity was collected at 620 nm. The concentrations of quenchers **2a** (1x 10^{-2} mmol/mL). The ratio of I_0/I was plotted as a function of the quencher concentration [Quencher] (I_0 = emission intensity of the photocatalyst in isolation at the specified wavelength; I = observed emission intensity of the photocatalyst with added quencher). Stern-Volmer analysis revealed that the luminescence emission of $Ru(bpy)_3(PF_6)_2$ was quenched efficiently by α-iodonium diazo **2a**, whereas no quenching was observed with **1a**.

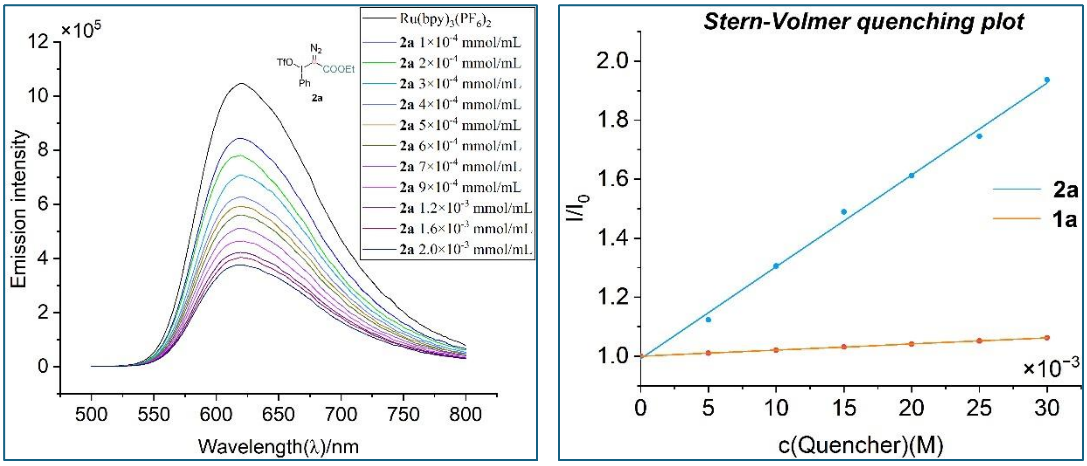

Figure S2. Stern-Volmer luminescence quenching analysis using $Ru(bpy)_3(PF_6)_2$ as the photocatalyst

3.3 Cyclic voltammetry studies

Measurement of the redox potential of **1a** and **2a** in MeCN: a standard three electrode cell configuration was used to collect cyclic voltammograms (CVs) at room temperature with VersaSTAT 3 Potentiostat Galvanostat from Princeton Applied Research. Samples were prepared with 0.15 mmol of the substrate in 5.0 mL of 0.1 M tetrabutylammonium tetrafluoroborate in dry acetonitrile. The samples were bubbled with argon for 2 min. Measurements were conducted using glassy carbon working electrode, platinum wire counter electrode, and silver-silver chloride reference electrode in a scan rate of 0.1 V/s. The electrolyte solution contains 0.1 M tetrabutylammonium hexafluorophosphate ($TBAPF_6$) and **1a** or **2a** in MeCN.

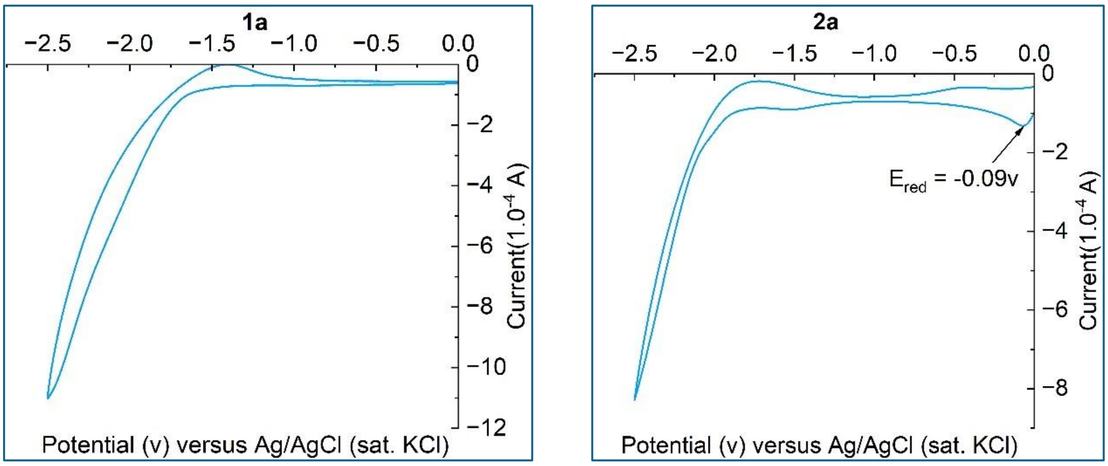

Figure S3. Cyclic voltammetry for **1a** and **2a**

3.4 Radical capture experiments

Radical capture experiments with TEMPO as the radical scavenger

In an oven-dried 8 ml Schlenk tube equipped with a PTFE-coated oval stirring bar, $Ru(bpy)_3(PF_6)_2$ (1 mol%, 0.9 mg), hypervalent iodine reagents **2a** (68.4 mg, 0.15 mmol, 1.5 equiv.), **1a**(24.6 mg, 0.1 mmol, 1.0 equiv.) and $NaHCO_3$ (16.8 mg, 0.2 mmol, 2.0 equiv.) were charged under air, then the vessel was evacuated and re-filled with argon for three times. Acetonitrile (1.0 mL, 0.1 M), TEMPO (46.8 mg, 0.3 mmol, 3 equiv.) and H_2O (3.6 mg, 0.2 mmol, 2.0 equiv.) were added under argon atmosphere. The vessel was sealed with the screw cap, then irradiated at white LEDs (50 W) at 0 °C for 2 hours. After irradiation, the analysis showed that the desired product **3** cannot be detected by GC-MS, and isolation of radical-trapped species **98** in 7% yield.

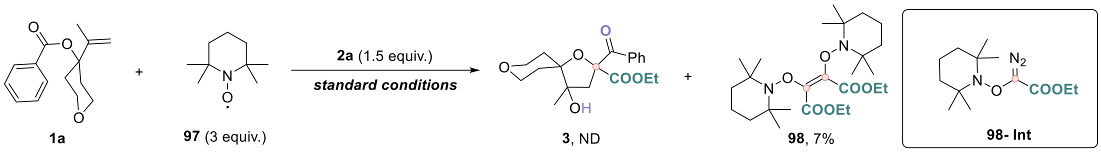

3.5 ^{18}O labelling experiments

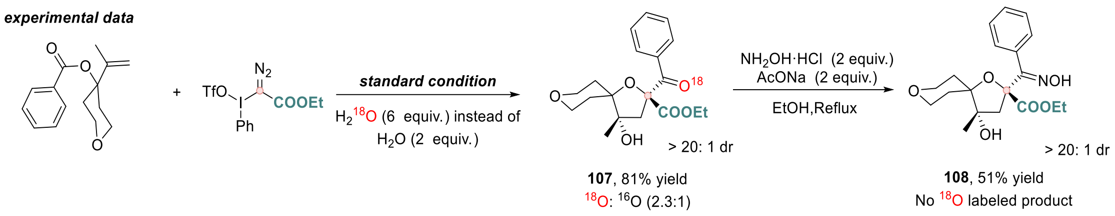

In an oven-dried 8 ml Schlenk tube equipped with a PTFE-coated oval stirring bar, $Ru(bpy)_3(PF_6)_2$ (1 mol%, 0.9 mg), hypervalent iodine reagents **2a** (68.4 mg, 0.15 mmol, 1.5 equiv.), **1a** (24.6 mg, 0.1 mmol, 1.0 equiv.) and $NaHCO_3$ (16.8 mg, 0.2 mmol, 2.0 equiv.) were charged under air, then the vessel was evacuated and re-filled with argon for three times. Acetonitrile (1.0 mL, 0.1 M) and $H_2{}^{18}O$ (10.8 mg, 0.6 mmol, 6.0 equiv.) were added under argon atmosphere. The vessel was sealed with the screw cap, then irradiated at white LEDs (50 W) at 0 °C for 2 hours. After irradiation, the solvent was evaporated under reduced pressure and the residue was purified by flash column chromatography (silica gel, hexane: EtOAc = 3:1, v/v) to give the desired products **107**. Then high-purity acetonitrile was added for HRMS analysis.

In an oven-dried 8 ml Schlenk tube equipped with a PTFE-coated oval stirring bar, **107** (35.0 mg, 0.1 mmol, 1.0 equiv.), hydroxylamine hydrochloride (13.6 mg, 0.2 mmol, 2.0 equiv.), sodium acetate (16.4 mg, 0.20 mmol, 2.0 equiv.), were charged under air, then the vessel was evacuated and re-filled with argon for three times. EtOH (1 mL) were added under argon atmosphere. The mixture

was heated under reflux for 24 h. The solvent was evaporated under reduced pressure and the residue was purified by flash column chromatography (silica gel, hexane: EtOAc = 3:1, v/v) to give the desired products. Then high-purity acetonitrile was added for HRMS analysis.

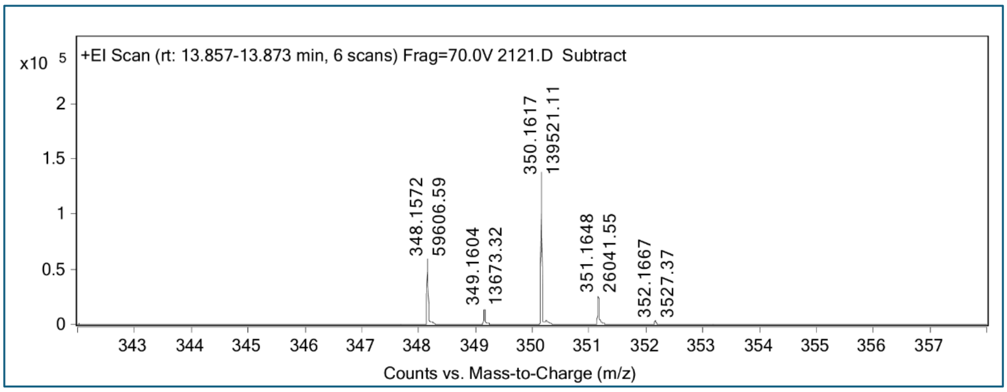

Figure S4. Mass Spectrum SmartFormula Report for **107**

Tandem MS analysis showed that fragment **107**-A contained the ^{18}O label while **107**-B did not, suggesting that the labeled oxygen was positioned in the carbonyl group of the benzoyl moiety.

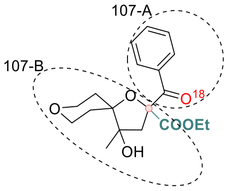

Fragment	Formula	Calc. Mass	Detected
107-A-^{18}O	$C_7H_5{}^{18}O$	107.0377	√
107-A-O	C_7H_5O	105.0335	√
107-B-^{18}O	$C_{12}H_{19}O_4{}^{18}O$	245.1275	×
107-B-O	$C_{12}H_{19}O_5$	243.1232	√

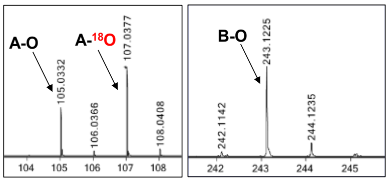

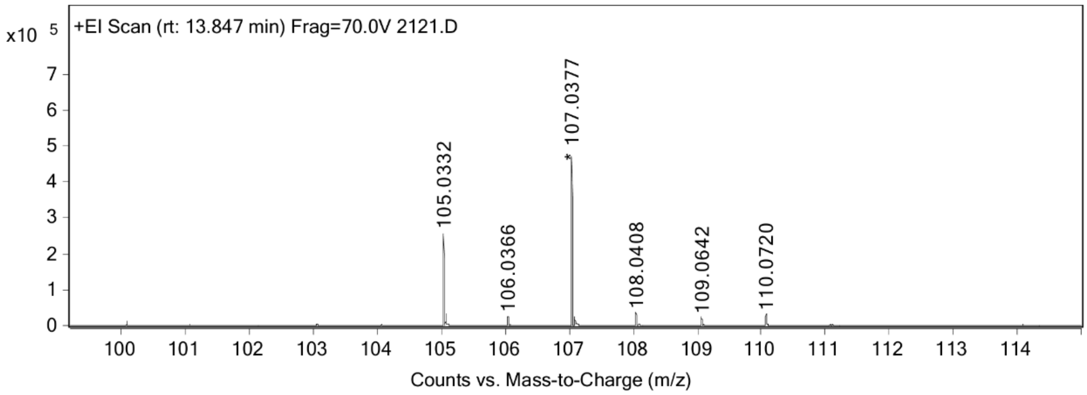

Figure S5. Secondary resolution mass spectrometry report for **107-A**

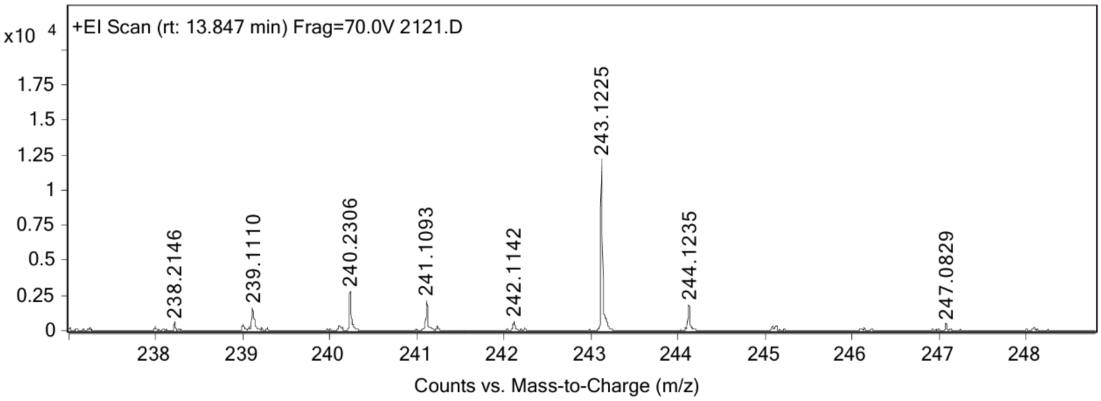

Figure S6. Secondary resolution mass spectrometry report for **107-B**

3.6 allylic amine derivatives instead of allylic benzoates

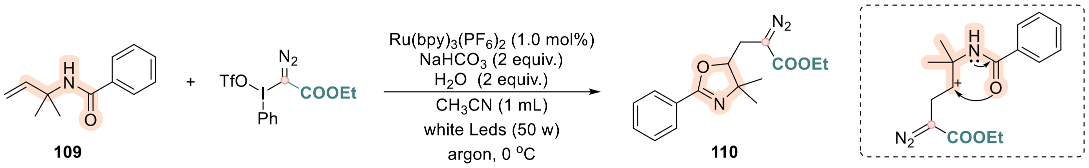

In an oven-dried 8 ml Schlenk tube equipped with a PTFE-coated oval stirring bar, $Ru(bpy)_3(PF_6)_2$ (1 mol%, 0.9 mg), hypervalent iodine reagents **2a** (68.4 mg, 0.15 mmol, 1.5 equiv.), **109** (24.6 mg, 0.1 mmol, 1.0 equiv.) and $NaHCO_3$ (16.8 mg, 0.2 mmol, 2.0 equiv.) were charged under air, then the vessel was evacuated and re-filled with argon for three times. Acetonitrile (1.0 mL, 0.1 M) and H_2O (0.2 mmol, 2.0 equiv.) were added under argon atmosphere. The vessel was sealed with the screw cap, then irradiated at white LEDs (50 W) at 0 °C for 2 hours. After irradiation, the solvent was evaporated under reduced pressure and the residue was purified by flash column chromatography (silica gel, hexane: EtOAc = 2:1, v/v) to give the desired products **107**.

3.7 Determination of quantum yields

Photon flux was determined by chemical actinometry using potassium ferrioxalate, which was purchased from Alfa Aesar, in a setup using blue LEDs as a source of irradiation. Blue LEDs were used because their emission profile lines up with known photochemical data for the actinometer. [4,5] Great care is taken to ensure that the compound is kept in the dark when not in use.

Procedure for photon flux: The following solutions must be prepared ahead of time:

1. 0.05 M sulfuric acid stock solution
In a 100 mL volumetric flask, 0.281 mL of concentrated sulfuric acid (17.8 M) was added to 90 mL deionized water. Then, water was added until the 100 mL graduation mark was reached.

SUPPORTING INFORMATION

2. Ferrioxalate solution

A 0.15 M solution of potassium ferrioxolate was prepared by dissolving potassium ferrioxolate ($K_3FeC_2O_4*3H_2O$, MW 491.243) (1.842 g, 3.75 mmol) with the 0.05 M sulfuric acid solution prepared in a 25 mL volumetric flask. Make every precaution to prepare and store the solution in the dark.

3. Developer solution

225 g of sodium acetate trihydrate was dissolved in 1 liter of 0.5 M sulfuric acid. 10 g of 1,10- phenantroline was added to this solution. Store in the dark. A 1cm x 1cm quartz cuvette was charged with 3 mL of 0.15 M aqueous potassium ferrioxalate solution. Two sides of the cuvette are taped over with black electrical tape to ensure a minimum pathway of the light of 1 cm. To agitate the solution sufficiently, the solution was continually sparged with a steady stream of nitrogen gas. To determine the photon flux of the LED, the ferrioxalate solution (2.0 mL) was placed in a cuvette and irradiated for 10 seconds at λmax= 456 nm. Aluquots of solution were taken at time points between 1 and 6 minutes of irradiation. 10 μL of irradiated ferrioxalate solution is immediately added to 5 mL of the developer solution and the flask is wrapped in aluminum foil. A nonirradiated sample is prepared by adding 10 μL of the ferrioxalate solution to 5 mL of developer solution. The solutions were left in the dark for 30 minutes to an hour, eventually becoming bright red. Solutions were transferred to a separate cuvette and the absorbance spectrum of the $Fe(phen)_3^{2+}$ complex was obtained. The absorbance at 510 nm (ε = 11,100 M^{-1} cm^{-1}) was measured for each sample. Each sample preparation and measurements were repeated two more times. The average of the absorption of the irradiated and non-irradiated samples were determined and used for the calculation of photon flux.

$$\text{mol Fe}^{2+} = \frac{\text{V1}\times\text{V3}\times\Delta\text{A (510 nm)}}{\text{V2}\times \text{l}\times\varepsilon}$$

Where:

ΔA_{510} nm = the difference between the absorbance between the sample and the blank as measured at 510 nm.

l= the path length of the cuvette (5 cm)

ε510nm= the extinction coefficient of $Fe(phen)_3^{2+}$ complex at 510 nm (11,100 M^{-1} cm^{-1})

V1 = the total volume of the irradiated solution (3 mL; 3 * 10^{-3} L)

V2 = the volume of the aliquot removed from solution (10 μL; 10^{-5} L)

V3 = the volume that aliquots are diluted with (5 mL; 5 * 10^{-3} L)

Photon flux may be determined by:

$$\text{photon flux} = \frac{\text{mol Fe}^{2+}}{\phi\bullet \text{t}\bullet f}$$

Where Φ is the quantum yield for the ferrioxalate actinometer (1.01 for a 0.15 M solution at λ = 456 nm), t is the irradiation time (10 s), and f is the fraction of light absorbed at λ = 456

nm (F ~ 1 at 456 nm at 0.15 M ferrioxalate). The average photon flux was thus calculated to be 6.133×10^{-9} einsteins s^{-1}

In an oven-dried 8 ml Schlenk tube equipped with a PTFE-coated oval stirring bar, $Ru(bpy)_3(PF_6)_2$ (1 mol%, 0.9 mg), hypervalent iodine reagents **2a** (68.4 mg, 0.15 mmol, 1.5 equiv.), **1a**(24.6 mg, 0.1 mmol, 1.0 equiv.) and $NaHCO_3$ (16.8 mg, 0.2 mmol, 2.0 equiv.) were charged under air, then the vessel was evacuated and re-filled with argon for three times. Acetonitrile (1.0 mL, 0.1 M), and H_2O (3.6 mg, 0.2 mmol, 2.0 equiv.) were added under argon atmosphere. The vessel was sealed with the screw cap, then irradiated at blue LEDs for 60 s. The reaction yield was determined by ^1H NMR spectroscopy using CH_2Br_2 as an internal standard, with all measurements performed in triplicate and the average yield reported. The quantum yield of the reaction can be calculated

$$\phi = \frac{\mathrm{nproduct}}{\phi_q \bullet t \bullet f_R}$$

where ϕ_q is the photon flux, t is the irradiation time (60 s). The fraction of light absorbed (f_R) by the reaction was determined by measuring the absorbance of a non-irradiated control reaction.

$$f = 1 - 10^{-A(455\ nm)}$$

$\phi_q = 6.133\times10^{-9}$

$f_R = 0.999$

yield (60 s) = 3.6% $\phi = 9.8$

Thus, the reactions´ quantum yield (ϕ) came out to be: $\phi = 9.8$

3.8 DFT calculations

All calculations were performed with the Gaussian 16 program package[6]. The geometries were optimized using B3LYP[7,8] functional with def2-SVP[9] basis sets. Grimme's D3 empirical dispersion correction was applied to account for long-range interactions[10]. Solvation effects were incorporated using the polarizable continuum model (PCM) with acetonitrile[11]. Harmonic vibration frequency calculations at the same level were performed to verify all stationary points as local minima with no imaginary frequency or transition states with one imaginary frequency, and to derive the thermochemistry correction terms at 298 K and 1 atm. Key transition states were further confirmed to connect corresponding reactants and products by intrinsic reaction coordinate (IRC) calculations[12,13]. Single-point energies were corrected using def2-TZVPP basis sets[9].

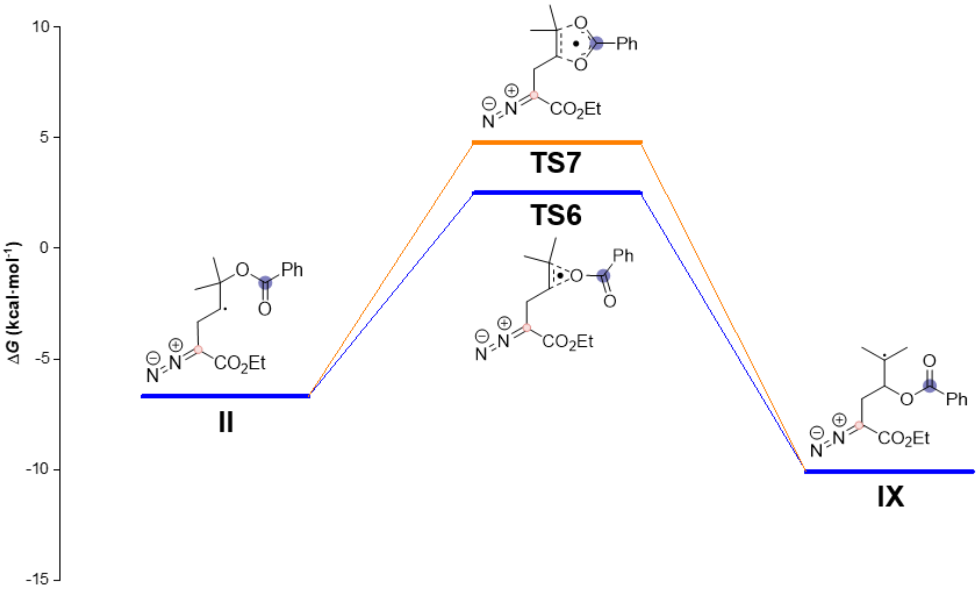

Figure S7. Energy profiles for the [1,2]-benzoyloxy radical migration of the radical intermediate **II** at the B3LYP-D3/def2-TZVPP//def2-SVP/PCM(acetonitrile) level of theory.

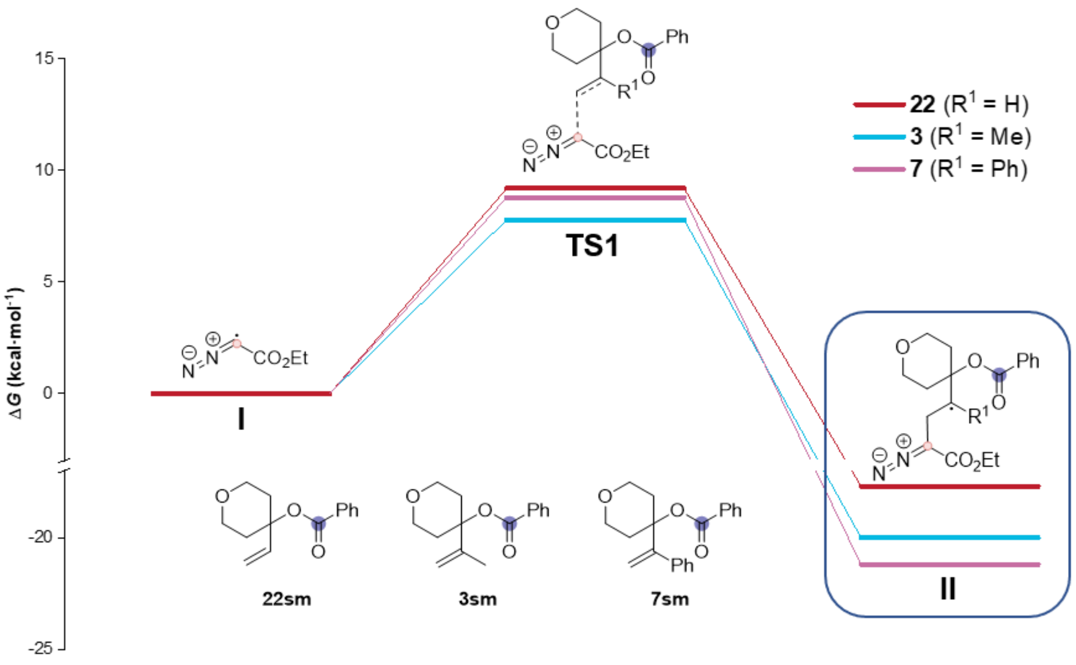

Figure S8. Energy profiles for the radical addition of the different substrates with the radical intermediate **I** at the B3LYP-D3/def2-TZVPP//def2-SVP/PCM(acetonitrile) level of theory.

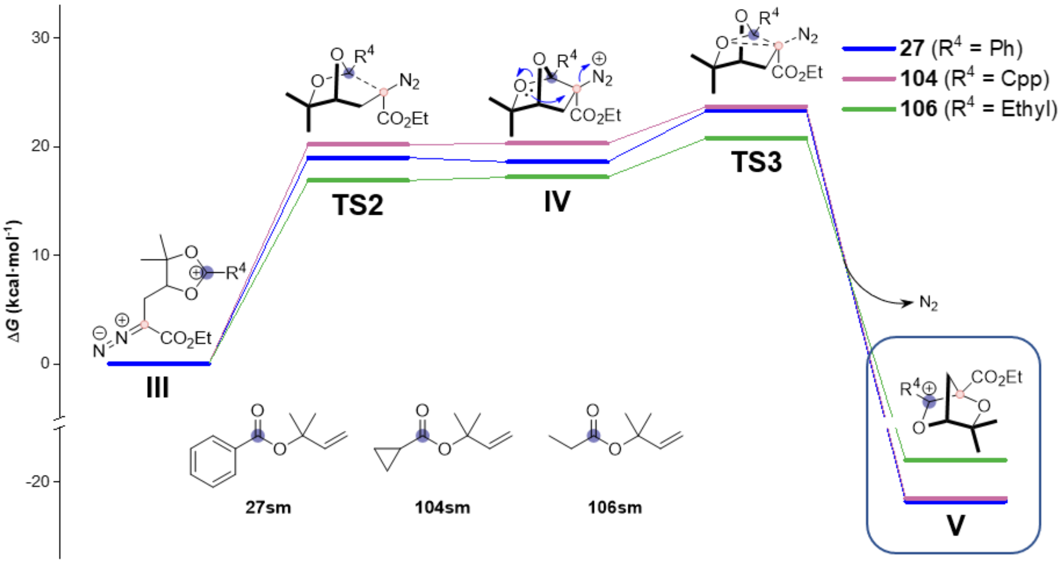

Figure S9. Energy profiles for the nucleophilic and electrophilic attack to generate intermediate **V** of the different substrates at the B3LYP-D3/def2-TZVPP//def2-SVP/PCM(acetonitrile) level of theory.

Table S6. Thermal corrections to free energies (ΔG_c), single-point energies (E), free energies (G), and relative free energies (ΔG) of key points.

Key points	ΔG_c (H)	E (H)	G (H)	ΔG (kcal mol^{-1})
**Ru^{II}**	0.426659	−1581.72208096	−1581.295422	0.0
**Ru^{II}–S$_1$**	0.424234	−1581.636948	−1581.212714	51.9
**Ru^{II}–T$_1$**	0.422698	−1581.647572	−1581.224874	44.3
**2a**	0.152889	−1906.836763	−1906.683874	–
**Ru^{III}**	0.426223	−1581.512168	−1581.085945	–
**2a$^{\bullet-}$**	0.14753	−1906.994292	−1906.846762	29.2
**TfO$^-$**	−0.005355	−961.9549637	−961.9603187	–
**PhI**	0.058365	−529.5634364	−529.5050714	–
**I**	0.056606	−415.4673507	−415.4107447	10.8
**1v**	0.190831	−616.452918	−616.262087	–
**TS1**	0.271136	−1031.92932	−1031.658184	20.0
**II**	0.275881	−1031.976534	−1031.700653	−6.7
**TS6**	0.27188	−1031.95796	−1031.68608	2.5
**TS7**	0.274104	−1031.956494	−1031.68239	4.8
**IX**	0.271238	−1031.977451	−1031.706213	−10.1
**III**	0.283204	−1031.834975	−1031.551771	−33.9
**TS2**	0.284118	−1031.805654	−1031.521536	−14.9
**IV**	0.283423	−1031.805555	−1031.522132	−15.3

**TS3**	0.280586	−1031.795169	−1031.514583	−10.5
**V**	0.276788	−1031.86335	−1031.586562	−55.7
**V′** (no N_2)	0.276839	−922.2858315	−922.0089925	–
**H_2O**	0.002842	−76.4737612	−76.4709192	–
**VI**	0.320661	−1075.247404	−1074.926743	−40.6
**TS4**	0.322961	−1075.246181	−1074.92322	−38.4
**VII**	0.322302	−1075.250839	−1074.928537	−41.7
**TS5**	0.322447	−1075.248247	−1074.9258	−40.0
**VIII**	0.300939	−998.7811823	−998.4802433	−55.9
**22sm**	0.230827	−769.1523726	−768.9215456	–
**TS1(22)**	0.31188	−1184.629567	−1184.317687	20.0
**II(22)**	0.31665	−1184.677165	−1184.360515	−6.9
**3sm**	0.258287	−808.4873725	−808.2290855	–
**TS1(3)**	0.337045	−1223.964446	−1223.627401	18.6
**II(3)**	0.343253	−1224.014899	−1223.671646	−9.2
**7sm**	0.307442	−1000.31504	−1000.007598	–
**TS1(7)**	0.389494	−1415.793772	−1415.404278	19.6
**II(7)**	0.392566	−1415.844764	−1415.452198	−9.4
**III(104)**	0.264452	−917.4305617	−917.1661097	0.0
**TS2(104)**	0.266344	−917.400186	−917.133842	20.2
**IV(104)**	0.267039	−917.400731	−917.133692	20.3
**TS3(104)**	0.263716	−917.3920688	−917.1283528	23.7
**V(104)**	0.259272	−917.4597203	−917.2004483	−21.5
**III(106)**	0.259804	−879.3393513	−879.0795473	0.0
**TS2(106)**	0.260795	−879.3134437	−879.0526487	16.9
**IV(106)**	0.26125	−879.313388	−879.052138	17.2
**TS3(106)**	0.258617	−879.3050193	−879.0464023	20.8
**V(106)**	0.254904	−879.3630876	−879.1081836	−18.0

Cartesian coordinates

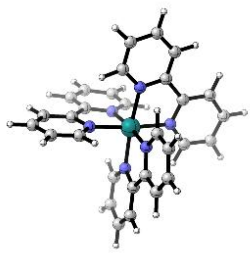

Ru^{II}

6	-2.788956000	0.677882000	0.586294000
6	-4.049137000	0.668475000	1.192136000

6	-4.279246000	-0.162407000	2.286452000
6	-3.238974000	-0.968603000	2.752263000
6	-2.007500000	-0.914700000	2.106824000
7	-1.172387000	1.354260000	-1.042703000
6	-2.439964000	1.516735000	-0.573289000
6	-3.319739000	2.420589000	-1.177178000
6	-2.891830000	3.167495000	-2.272617000
6	-1.588506000	2.992755000	-2.741728000
6	-0.761042000	2.077634000	-2.098669000
6	-0.098639000	-2.867012000	-0.573866000
6	-0.446083000	-4.080378000	-1.175653000
6	-1.314562000	-4.083386000	-2.264999000
6	-1.817617000	-2.867245000	-2.730589000
6	-1.433672000	-1.693004000	-2.090479000
7	0.996998000	-1.484920000	1.043477000
6	0.811984000	-2.751269000	0.578634000
6	1.456327000	-3.838544000	1.177509000
6	2.299978000	-3.622587000	2.264882000
6	2.482737000	-2.318861000	2.729556000
6	1.814946000	-1.278764000	2.090690000
7	0.795461000	1.603428000	1.043704000
6	1.984609000	2.072042000	0.574787000
6	2.616812000	3.162091000	1.181036000
6	2.019700000	3.780425000	2.277264000
6	0.798255000	3.292137000	2.745048000
6	0.219728000	2.202808000	2.100929000
7	1.749553000	0.334155000	-1.054130000
6	2.529353000	1.347597000	-0.586999000
6	3.750240000	1.654273000	-1.196407000
6	4.176317000	0.909565000	-2.294030000
6	3.367748000	-0.128412000	-2.761002000
6	2.162531000	-0.383076000	-2.113966000
1	1.302477000	-4.846823000	0.796181000
1	-5.258672000	-0.179483000	2.766876000
1	2.808804000	-4.462156000	2.740906000
1	-1.168459000	-1.525284000	2.439894000
1	1.928822000	-0.247392000	2.423013000
1	-4.331120000	2.542951000	-0.792837000
1	3.569999000	3.524695000	0.800167000
1	-3.567936000	3.877257000	-2.751755000
1	0.292564000	3.742605000	3.599588000
1	0.260787000	1.905732000	-2.435498000
1	-0.729724000	1.787408000	2.437685000
1	4.363914000	2.470017000	-0.817315000

1	-1.593237000	-5.023706000	-2.743075000
1	3.658460000	-0.738089000	-3.616971000
1	-1.798484000	-0.722307000	-2.425844000
1	1.499220000	-1.180225000	-2.448970000
44	-0.001866000	0.001447000	0.001318000
7	-1.782683000	-0.112171000	1.051912000
7	-0.593747000	-1.687830000	-1.040549000
1	-1.208722000	3.554333000	-3.595695000
1	3.133654000	-2.100607000	3.576619000
1	2.503581000	4.631715000	2.758565000
1	5.127077000	1.138879000	-2.777769000
1	-4.845901000	1.303950000	0.809488000
1	-3.370881000	-1.635433000	3.604787000
1	-2.499145000	-2.818899000	-3.580344000
1	-0.042139000	-5.016781000	-0.794653000

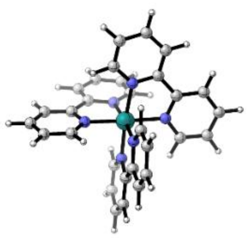

Ru^{II}–S$_1$

6	0.461872000	-2.802353000	0.541257000
6	1.014007000	-3.964805000	1.149010000
6	1.918052000	-3.847751000	2.179745000
6	2.305712000	-2.557967000	2.635489000
6	1.747823000	-1.455392000	2.020874000
7	-0.847112000	-1.552937000	-1.021728000
6	-0.462423000	-2.802281000	-0.541243000
6	-1.014772000	-3.964642000	-1.148975000
6	-1.918823000	-3.847440000	-2.179688000
6	-2.306273000	-2.557593000	-2.635432000
6	-1.748168000	-1.455110000	-2.020846000
6	2.702410000	0.971892000	-0.619781000
6	3.956146000	1.055963000	-1.229337000
6	4.260323000	0.197609000	-2.284749000
6	3.307264000	-0.730384000	-2.706977000
6	2.076852000	-0.769988000	-2.058369000
7	1.022216000	1.555342000	0.979312000
6	2.271423000	1.814640000	0.508515000
6	3.053532000	2.820956000	1.080312000
6	2.540573000	3.562431000	2.143298000
6	1.254914000	3.286103000	2.610982000

6	0.523509000	2.273578000	1.997778000
7	-1.783614000	0.066143000	1.049022000
6	-2.702218000	0.972522000	0.619763000
6	-3.955957000	1.056835000	1.229278000
6	-4.260393000	0.198431000	2.284575000
6	-3.307586000	-0.729856000	2.706725000
6	-2.077157000	-0.769690000	2.058162000
7	-1.021898000	1.555592000	-0.979333000
6	-2.270992000	1.815265000	-0.508446000
6	-3.052778000	2.821926000	-1.080081000
6	-2.539616000	3.563345000	-2.143009000
6	-1.254087000	3.286607000	-2.610808000
6	-0.523002000	2.273760000	-1.997755000
1	4.052693000	3.025496000	0.699804000
1	2.338785000	-4.743001000	2.641426000
1	3.141189000	4.350437000	2.600046000
1	2.009838000	-0.444707000	2.337075000
1	-0.482945000	2.019724000	2.330397000
1	-0.719955000	-4.949169000	-0.787457000
1	-4.689726000	1.782206000	0.882594000
1	-2.339722000	-4.742620000	-2.641352000
1	-3.506959000	-1.420962000	3.525731000
1	-2.010006000	-0.444382000	-2.337054000
1	-1.298921000	-1.475932000	2.345409000
1	-4.051839000	3.026786000	-0.699486000
1	5.236203000	0.252770000	-2.769557000
1	-0.814969000	3.842511000	-3.439197000
1	1.298425000	-1.475999000	-2.345667000
1	0.483343000	2.019586000	-2.330463000
44	-0.000015000	0.025331000	-0.000025000
7	0.846779000	-1.553069000	1.021729000
7	1.783565000	0.065790000	-1.049108000
1	-3.020100000	-2.426195000	-3.447766000
1	0.815944000	3.842073000	3.439406000
1	-5.236281000	0.253776000	2.769348000
1	-3.139973000	4.351626000	-2.599621000
1	0.719030000	-4.949282000	0.787489000
1	3.019538000	-2.426685000	3.447843000
1	3.506428000	-1.421436000	-3.526080000
1	4.690103000	1.781121000	-0.882606000

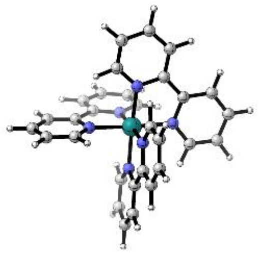

Ru^{II}–T$_1$

6	-2.260500000	-1.824407000	0.515886000
6	-3.027787000	-2.847583000	1.078018000
6	-2.505672000	-3.589686000	2.136211000
6	-1.225798000	-3.296214000	2.609549000
6	-0.509024000	-2.267733000	2.005741000
7	-1.796435000	-0.060375000	-1.034605000
6	-2.705005000	-0.976257000	-0.603087000
6	-3.963056000	-1.063519000	-1.203335000
6	-4.282972000	-0.198844000	-2.248951000
6	-3.340403000	0.739746000	-2.671834000
6	-2.104393000	0.782026000	-2.034931000
6	2.258737000	-1.826206000	-0.515977000
6	3.025351000	-2.849642000	-1.078560000
6	2.502671000	-3.591044000	-2.136964000
6	1.222900000	-3.296659000	-2.610023000
6	0.506825000	-2.267953000	-2.005779000
7	1.796029000	-0.062203000	1.034968000
6	2.703860000	-0.978723000	0.603255000
6	3.961760000	-1.067301000	1.203619000
6	4.282310000	-0.203307000	2.249598000
6	3.340497000	0.735932000	2.672720000
6	2.104610000	0.779534000	2.035665000
7	-0.831987000	1.555448000	1.022309000
6	-0.455228000	2.812869000	0.542029000
6	-1.013197000	3.972012000	1.153696000
6	-1.921604000	3.848543000	2.177340000
6	-2.309883000	2.552211000	2.624718000
6	-1.746362000	1.452624000	2.014936000
7	0.833471000	1.554264000	-1.022522000
6	0.458498000	2.812216000	-0.542241000
6	1.018049000	3.970567000	-1.153960000
6	1.926139000	3.845807000	-2.177726000
6	2.312473000	2.548927000	-2.625198000
6	1.747502000	1.450130000	-2.015317000
1	4.687219000	-1.800663000	0.856175000
1	-3.094728000	-4.390928000	2.584928000
1	5.261922000	-0.262799000	2.726307000

1	0.492520000	-2.000834000	2.343440000
1	1.333524000	1.492921000	2.325025000
1	-4.689153000	-1.796348000	-0.856097000
1	-0.718192000	4.957925000	0.795486000
1	-5.262704000	-0.257280000	-2.725542000
1	-3.029511000	2.417242000	3.431722000
1	-1.332701000	1.494825000	-2.324123000
1	-2.005929000	0.440625000	2.328142000
1	0.724495000	4.956895000	-0.795704000
1	3.091199000	-4.392451000	-2.586078000
1	3.031748000	2.412941000	-3.432345000
1	-0.494584000	-2.000306000	-2.343293000
1	2.005619000	0.437780000	-2.328573000
44	-0.000117000	-0.008248000	0.000105000
7	-1.016146000	-1.550625000	0.990575000
7	1.014516000	-1.551472000	-0.990456000
1	-3.551640000	1.436385000	-3.483180000
1	3.552237000	1.432086000	3.484352000
1	-2.348378000	4.739819000	2.641180000
1	2.354126000	4.736482000	-2.641603000
1	-4.022756000	-3.065012000	0.693590000
1	-0.780023000	-3.851172000	3.435068000
1	0.776676000	-3.851066000	-3.435670000
1	4.020236000	-3.067808000	-0.694315000

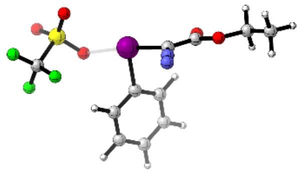

2a

6	-0.260954000	4.383454000	0.148358000
6	-1.189373000	3.736302000	-0.672689000
6	-1.172423000	2.342704000	-0.799925000
6	-0.210837000	1.643490000	-0.074069000
6	0.730844000	2.251407000	0.752434000
6	0.694151000	3.645866000	0.855380000
53	-0.180753000	-0.493779000	-0.243151000
6	-2.153578000	-0.737001000	0.475426000
8	2.103891000	0.095536000	-1.119936000
16	3.141239000	-0.980495000	-0.879792000
8	4.255822000	-0.944548000	-1.830402000
6	-3.296699000	-0.590511000	-0.437991000
8	-4.448089000	-0.875774000	0.167911000

8	-3.180747000	-0.238898000	-1.594125000
6	-5.660855000	-0.731284000	-0.609784000
6	-6.824563000	-1.087913000	0.285698000
1	-0.280824000	5.471524000	0.237440000
1	-1.932138000	4.313014000	-1.227841000
1	-1.890182000	1.825467000	-1.437635000
1	1.418191000	4.151875000	1.497386000
1	-5.718263000	0.305659000	-0.974518000
1	-5.592269000	-1.393239000	-1.486305000
1	-7.765145000	-0.987973000	-0.276481000
1	-6.742718000	-2.125717000	0.642616000
1	-6.869250000	-0.418448000	1.158032000
7	-2.333853000	-1.013262000	1.740597000
7	-2.481421000	-1.250190000	2.835313000
1	1.472550000	1.670997000	1.299607000
8	2.532362000	-2.286735000	-0.568045000
6	3.883183000	-0.423880000	0.741582000
9	4.769862000	-1.314980000	1.180971000
9	2.917931000	-0.295935000	1.666389000
9	4.488624000	0.754997000	0.604893000

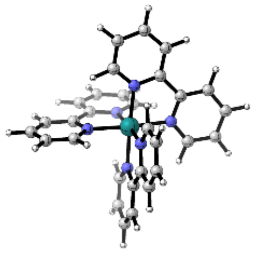

Ru^{III}

6	-2.232900000	1.816595000	-0.559667000
6	-2.981960000	2.835865000	-1.150078000
6	-2.436467000	3.558347000	-2.210376000
6	-1.150893000	3.250004000	-2.657734000
6	-0.450089000	2.226823000	-2.028374000
7	-1.805669000	0.071246000	1.020487000
6	-2.698214000	0.994673000	0.567744000
6	-3.955235000	1.116398000	1.162082000
6	-4.292122000	0.281481000	2.226579000
6	-3.366073000	-0.661626000	2.674779000
6	-2.130450000	-0.740197000	2.041469000
6	2.218908000	1.841434000	0.554288000
6	2.954495000	2.874842000	1.137037000
6	2.400658000	3.596252000	2.193716000
6	1.120948000	3.271625000	2.646414000
6	0.433574000	2.234724000	2.024807000

7	1.813471000	0.080663000	-1.014638000
6	2.695059000	1.016819000	-0.566642000
6	3.953821000	1.144664000	-1.156083000
6	4.304381000	0.301955000	-2.209933000
6	3.390504000	-0.655921000	-2.651734000
6	2.152438000	-0.739719000	-2.023992000
7	-0.843724000	-1.605670000	-1.007842000
6	-0.473955000	-2.837663000	-0.560235000
6	-0.998240000	-3.990975000	-1.146690000
6	-1.909775000	-3.871331000	-2.194743000
6	-2.282565000	-2.600489000	-2.635215000
6	-1.729285000	-1.487032000	-2.012424000
7	0.852539000	-1.605913000	1.004999000
6	0.484702000	-2.837832000	0.555589000
6	1.010535000	-3.991303000	1.140448000
6	1.920653000	-3.871991000	2.189791000
6	2.290713000	-2.601272000	2.632780000
6	1.736560000	-1.487805000	2.010776000
1	4.656806000	1.893317000	-0.795531000
1	-3.012496000	4.356723000	-2.680521000
1	5.285326000	0.393157000	-2.678689000
1	0.554062000	1.949903000	-2.348213000
1	1.407764000	-1.470288000	-2.338033000
1	-4.666549000	1.855411000	0.797970000
1	-0.700772000	-4.975045000	-0.789097000
1	-5.271364000	0.368353000	2.699683000
1	-2.993214000	-2.462115000	-3.449949000
1	-1.376591000	-1.459175000	2.360136000
1	-1.989313000	-0.476310000	-2.325445000
1	0.715004000	-4.975252000	0.780915000
1	2.965981000	4.405975000	2.657389000
1	2.999742000	-2.462924000	3.448935000
1	-0.565508000	1.945169000	2.349104000
1	1.993980000	-0.477114000	2.326022000
44	0.001976000	0.001268000	0.000293000
7	-0.981333000	1.526628000	-1.011539000
7	0.972060000	1.537235000	1.009830000
1	-3.588004000	-1.333603000	3.503733000
1	3.623805000	-1.335455000	-3.471366000
1	-2.326294000	-4.765825000	-2.660268000
1	2.338143000	-4.766499000	2.654413000
1	-3.981459000	3.066665000	-0.785494000
1	-0.686792000	3.789263000	-3.483404000
1	0.650939000	3.809054000	3.469938000

1	3.950082000	3.117422000	0.769551000

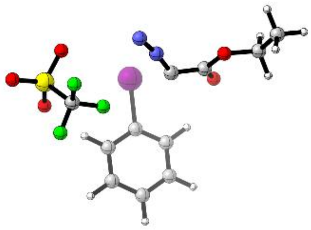

2a•–

6	0.301443000	3.955062000	1.986536000
6	-0.978025000	3.695083000	1.483880000
6	-1.156762000	2.781969000	0.439087000
6	-0.029780000	2.144292000	-0.084771000
6	1.256787000	2.372057000	0.407236000
6	1.410277000	3.292067000	1.451259000
53	-0.283004000	0.753854000	-1.672758000
6	-1.775291000	-0.787557000	0.248407000
8	3.478177000	0.292054000	-0.653080000
16	3.322648000	-1.165656000	-0.423207000
8	4.591130000	-1.885196000	-0.173297000
6	-3.177345000	-0.437702000	0.042180000
8	-3.990692000	-1.412261000	0.492701000
8	-3.579789000	0.617330000	-0.406547000
6	-5.407132000	-1.138884000	0.473837000
6	-6.114583000	-2.329044000	1.081869000
1	0.432822000	4.670151000	2.801858000
1	-1.848873000	4.201660000	1.907104000
1	-2.151535000	2.547512000	0.058219000
1	2.409395000	3.481946000	1.851352000
1	-5.597243000	-0.212364000	1.038166000
1	-5.718863000	-0.961412000	-0.567471000
1	-7.201469000	-2.156124000	1.078364000
1	-5.907835000	-3.245015000	0.507541000
1	-5.792322000	-2.488474000	2.122193000
7	-1.281404000	-1.955869000	0.231120000
7	-0.692685000	-2.946848000	0.232778000
1	2.112476000	1.824383000	0.005893000
8	2.372444000	-1.853947000	-1.321839000
6	2.477381000	-1.252480000	1.243768000
9	2.301653000	-2.526145000	1.619610000
9	1.274453000	-0.662456000	1.222575000
9	3.213552000	-0.642509000	2.183786000

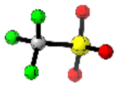

TfO⁻

8	1.243845000	-0.471218000	-1.364323000
16	0.916820000	0.000036000	0.000058000
8	1.243430000	-0.946232000	1.090189000
8	1.243503000	1.417340000	0.274165000
6	-0.953390000	-0.000033000	-0.000043000
9	-1.436976000	-1.228406000	-0.235262000
9	-1.436724000	0.410552000	1.181411000
9	-1.436856000	0.817908000	-0.946250000

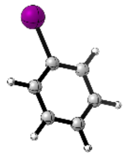

PhI

6	3.354923000	0.000006000	-0.000100000
6	2.653897000	-1.209602000	-0.000075000
6	1.254764000	-1.218580000	-0.000030000
6	0.569764000	-0.000009000	-0.000012000
6	1.254752000	1.218574000	-0.000036000
6	2.653884000	1.209609000	-0.000080000
53	-1.560572000	0.000000000	0.000044000
1	4.447040000	0.000014000	-0.000138000
1	3.194405000	-2.159110000	-0.000089000
1	0.711297000	-2.164578000	-0.000008000
1	3.194385000	2.159121000	-0.000098000
1	0.711276000	2.164566000	-0.000017000

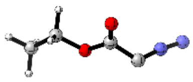

I

6	1.436338000	-0.082786000	0.712025000
6	0.211492000	0.538038000	0.182115000
8	-0.821086000	-0.292542000	0.225430000
8	0.179480000	1.697336000	-0.167029000
6	-2.103268000	0.226175000	-0.211822000
6	-3.119005000	-0.883781000	-0.077959000
1	-2.353102000	1.097165000	0.412495000
1	-2.002454000	0.573301000	-1.251147000
1	-4.105335000	-0.519046000	-0.401636000

1	-2.845669000	-1.745457000	-0.705448000
1	-3.199737000	-1.220216000	0.966629000
7	2.443775000	-0.413061000	0.030352000
7	3.425625000	-0.759793000	-0.475244000

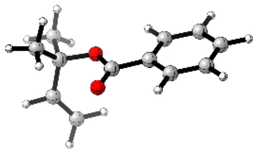

1v

6	2.338473000	0.307294000	-0.258833000
6	2.859955000	-0.411589000	0.968381000
6	0.057712000	-0.597909000	-0.261501000
6	2.207917000	-0.571901000	2.120255000
1	2.678155000	-1.072286000	2.970474000
1	1.182995000	-0.215396000	2.255965000
1	3.890105000	-0.771648000	0.869285000
6	2.768654000	-0.389542000	-1.553947000
1	2.315720000	0.118592000	-2.418615000
1	3.862528000	-0.324155000	-1.652286000
1	2.471502000	-1.443795000	-1.561781000
6	2.837629000	1.756611000	-0.246745000
1	3.936630000	1.778535000	-0.225851000
1	2.491748000	2.285681000	-1.147830000
1	2.459643000	2.278150000	0.644005000
8	0.889316000	0.452113000	-0.196298000
6	-1.370754000	-0.179917000	-0.122153000
6	-2.358295000	-1.175651000	-0.182981000
6	-1.744353000	1.160076000	0.068792000
6	-3.705081000	-0.836813000	-0.054971000
1	-2.049761000	-2.211698000	-0.331011000
6	-3.093202000	1.496043000	0.197327000
1	-0.974283000	1.929940000	0.115999000
6	-4.073921000	0.499956000	0.135479000
1	-4.470373000	-1.614714000	-0.102990000
1	-3.381510000	2.539041000	0.346330000
1	-5.128854000	0.765981000	0.236215000
8	0.406603000	-1.749886000	-0.410268000

SUPPORTING INFORMATION

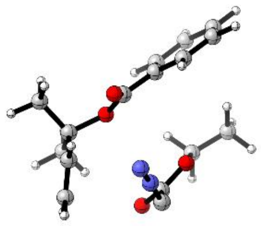

TS1

6	1.882639000	-1.645369000	-0.645843000
6	2.814477000	-0.894039000	0.284977000
6	-0.173649000	-1.172752000	0.658014000
6	3.782642000	-0.053396000	-0.086933000
1	4.428871000	0.413279000	0.661829000
1	3.967959000	0.215310000	-1.128315000
6	1.669386000	2.490667000	0.859064000
1	2.664597000	-1.109730000	1.344325000
6	1.900469000	-3.139157000	-0.311276000
1	1.172799000	-3.676350000	-0.938016000
1	2.902426000	-3.547614000	-0.508812000
1	1.657525000	-3.311015000	0.745971000
6	2.166740000	-1.403859000	-2.122800000
1	3.175477000	-1.754703000	-2.380894000
1	1.438303000	-1.957661000	-2.732117000
1	2.084228000	-0.334728000	-2.360164000
8	0.487987000	-1.164802000	-0.504184000
6	1.054387000	2.141094000	-0.430549000
8	1.695344000	2.010825000	-1.448933000
8	-0.270946000	2.087685000	-0.331793000
6	-1.025111000	1.931686000	-1.562190000
1	-0.926032000	0.884607000	-1.881075000
1	-0.568703000	2.570333000	-2.332175000
6	-2.457883000	2.310270000	-1.272453000
1	-3.067842000	2.156442000	-2.175214000
1	-2.873475000	1.687480000	-0.468643000
1	-2.531842000	3.368134000	-0.977940000
6	-1.640017000	-0.965078000	0.465337000
6	-2.401127000	-0.529780000	1.560449000
6	-2.271418000	-1.243065000	-0.756588000
6	-3.779775000	-0.360915000	1.431302000
1	-1.895749000	-0.324860000	2.505572000
6	-3.653457000	-1.088952000	-0.878267000
1	-1.675541000	-1.590051000	-1.601139000
6	-4.407809000	-0.644583000	0.212750000
1	-4.368349000	-0.010678000	2.282066000
1	-4.143861000	-1.312856000	-1.828064000

1	-5.488256000	-0.517281000	0.113389000
7	1.736891000	1.768226000	1.889902000
7	1.875823000	1.219257000	2.898243000
8	0.337052000	-1.328343000	1.748964000

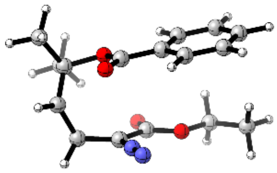

II

6	2.805504000	-0.614563000	-0.592475000
6	3.030530000	0.360571000	0.524691000
6	0.691235000	-1.364791000	0.396548000
6	2.377867000	1.706377000	0.585427000
1	2.829987000	2.298572000	1.394652000
1	2.536323000	2.263193000	-0.352731000
6	0.863734000	1.690435000	0.756795000
1	3.633949000	0.033307000	1.373274000
6	3.622504000	-1.889953000	-0.398182000
1	3.391641000	-2.613616000	-1.193597000
1	4.695278000	-1.650271000	-0.446311000
1	3.407633000	-2.346266000	0.576776000
6	3.068478000	0.013692000	-1.963743000
1	4.108649000	0.365621000	-2.022679000
1	2.903947000	-0.732984000	-2.755038000
1	2.388172000	0.858614000	-2.135197000
8	1.367715000	-0.986435000	-0.688601000
6	-0.050004000	1.908526000	-0.360898000
8	0.317093000	2.160046000	-1.494578000
8	-1.338059000	1.798997000	0.003089000
6	-2.332699000	1.992856000	-1.019340000
1	-2.173424000	1.245266000	-1.810932000
1	-2.193365000	2.989829000	-1.465893000
6	-3.690446000	1.843358000	-0.371717000
1	-4.479509000	1.980385000	-1.126556000
1	-3.801279000	0.841142000	0.067038000
1	-3.833257000	2.594359000	0.420343000
6	-0.762072000	-1.559703000	0.115114000
6	-1.614570000	-1.834237000	1.195056000
6	-1.287726000	-1.466865000	-1.182895000
6	-2.980481000	-2.015578000	0.979873000
1	-1.188590000	-1.895207000	2.197463000
6	-2.653802000	-1.655470000	-1.396123000

1	-0.621374000	-1.242334000	-2.015520000
6	-3.500924000	-1.929468000	-0.316657000
1	-3.642831000	-2.223911000	1.822954000
1	-3.061457000	-1.583247000	-2.406784000
1	-4.570736000	-2.072035000	-0.485843000
7	0.347173000	1.414909000	1.924777000
7	-0.076623000	1.166664000	2.947373000
8	1.184953000	-1.509177000	1.498927000

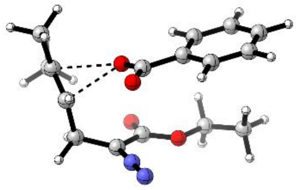

TS6

6	3.278055000	-0.492959000	-0.195424000
6	2.474366000	-0.000697000	0.815892000
6	0.029368000	-1.562409000	0.147524000
6	1.975145000	1.412342000	0.932487000
1	2.315054000	1.817152000	1.900216000
1	2.385766000	2.052610000	0.142933000
6	0.471085000	1.521534000	0.834262000
1	2.229440000	-0.667285000	1.644291000
6	3.808143000	-1.884918000	-0.129020000
1	3.477458000	-2.448343000	-1.017579000
1	4.911749000	-1.867426000	-0.164172000
1	3.483364000	-2.416022000	0.775195000
6	3.630220000	0.333093000	-1.391378000
1	4.425552000	1.055141000	-1.132649000
1	4.005693000	-0.293258000	-2.211903000
1	2.759719000	0.909002000	-1.738683000
8	0.902201000	-1.014525000	-0.631545000
6	-0.179874000	1.686953000	-0.464178000
8	0.415085000	1.988919000	-1.481596000
8	-1.501987000	1.465612000	-0.401139000
6	-2.250283000	1.574223000	-1.626561000
1	-1.773446000	0.937941000	-2.386416000
1	-2.201012000	2.616446000	-1.980614000
6	-3.667810000	1.135152000	-1.338667000
1	-4.271732000	1.206454000	-2.255889000
1	-3.687335000	0.091592000	-0.993222000
1	-4.129392000	1.772968000	-0.569196000
6	-1.242958000	-1.961794000	-0.579175000

6	-2.328656000	-2.424637000	0.176838000
6	-1.358283000	-1.867986000	-1.973574000
6	-3.519472000	-2.788794000	-0.454581000
1	-2.218997000	-2.487369000	1.260404000
6	-2.548518000	-2.235952000	-2.604754000
1	-0.510266000	-1.501926000	-2.553275000
6	-3.630689000	-2.696197000	-1.846697000
1	-4.364725000	-3.144607000	0.139377000
1	-2.634460000	-2.160163000	-3.691245000
1	-4.563051000	-2.979701000	-2.340798000
7	-0.276236000	1.258385000	1.872581000
7	-0.921606000	1.046054000	2.780918000
8	0.132855000	-1.740632000	1.360949000

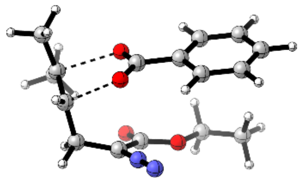

TS7

6	3.015214000	-0.508783000	-0.366664000
6	2.570603000	0.052183000	0.836686000
6	0.265136000	-1.365360000	-0.017681000
6	2.047298000	1.464419000	0.981348000
1	2.395983000	1.869894000	1.942233000
1	2.457129000	2.102914000	0.186619000
6	0.543965000	1.614396000	0.893388000
1	2.830552000	-0.471612000	1.756051000
6	3.641212000	-1.866467000	-0.340493000
1	3.307738000	-2.456451000	-1.205996000
1	4.738601000	-1.762202000	-0.421250000
1	3.405205000	-2.411771000	0.582602000
6	3.173064000	0.289644000	-1.621814000
1	4.159664000	0.788973000	-1.600559000
1	3.156752000	-0.368906000	-2.500562000
1	2.395172000	1.053840000	-1.732010000
8	0.886838000	-1.206032000	-1.089603000
6	-0.146813000	1.843946000	-0.371880000
8	0.416566000	2.149143000	-1.407486000
8	-1.472987000	1.665873000	-0.264150000
6	-2.253839000	1.761479000	-1.469488000
1	-1.883563000	1.008610000	-2.182894000
1	-2.099560000	2.755046000	-1.918401000
6	-3.699378000	1.519333000	-1.099120000

1	-4.328393000	1.580149000	-2.000159000
1	-3.824736000	0.520694000	-0.656524000
1	-4.051801000	2.273731000	-0.378964000
6	-1.209930000	-1.709158000	-0.104388000
6	-1.996657000	-1.771880000	1.054251000
6	-1.797158000	-1.941593000	-1.356144000
6	-3.358873000	-2.063983000	0.960217000
1	-1.530123000	-1.576029000	2.019903000
6	-3.158344000	-2.237211000	-1.448641000
1	-1.171103000	-1.882739000	-2.247225000
6	-3.941260000	-2.299260000	-0.290296000
1	-3.970290000	-2.106221000	1.864734000
1	-3.612334000	-2.415170000	-2.426334000
1	-5.007586000	-2.526284000	-0.362849000
7	-0.186248000	1.349152000	1.944099000
7	-0.806262000	1.126433000	2.866286000
8	0.739481000	-1.230951000	1.148806000

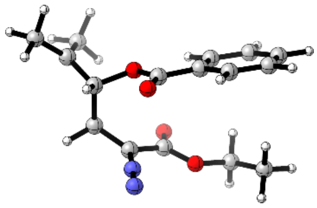

IX

6	3.724866000	-0.448028000	0.079144000
6	2.404337000	-0.122452000	0.691968000
6	0.283517000	-1.251515000	0.552345000
6	2.106139000	1.400639000	0.723199000
1	2.721903000	1.857097000	1.511752000
1	2.397234000	1.851505000	-0.234577000
6	0.646351000	1.719489000	0.901913000
1	2.337810000	-0.525979000	1.710119000
6	4.747872000	-1.203299000	0.859667000
1	4.665707000	-2.297102000	0.681745000
1	5.772591000	-0.920052000	0.565093000
1	4.641349000	-1.047089000	1.944672000
6	3.912656000	-0.255502000	-1.390107000
1	4.919757000	0.133269000	-1.622017000
1	3.820319000	-1.218407000	-1.932830000
1	3.166318000	0.426973000	-1.823526000
8	1.348924000	-0.752422000	-0.095898000
6	-0.229902000	1.901456000	-0.256715000
8	0.184647000	2.040149000	-1.392032000
8	-1.530793000	1.867922000	0.074504000

6	-2.494967000	1.990621000	-0.989908000
1	-2.139611000	1.414706000	-1.855504000
1	-2.553082000	3.050418000	-1.287721000
6	-3.819631000	1.471785000	-0.476998000
1	-4.584855000	1.569387000	-1.262031000
1	-3.737572000	0.409230000	-0.204683000
1	-4.153974000	2.039601000	0.404836000
6	-0.848152000	-1.557814000	-0.368197000
6	-1.999673000	-2.151835000	0.171680000
6	-0.805567000	-1.228899000	-1.731935000
6	-3.098495000	-2.417347000	-0.644334000
1	-2.017533000	-2.391589000	1.235796000
6	-1.909497000	-1.491872000	-2.544930000
1	0.082527000	-0.747960000	-2.139524000
6	-3.054709000	-2.085833000	-2.003852000
1	-3.993885000	-2.878067000	-0.221494000
1	-1.878752000	-1.227944000	-3.604294000
1	-3.917838000	-2.287784000	-2.642414000
7	0.092182000	1.630709000	2.084334000
7	-0.371907000	1.555315000	3.115456000
8	0.240341000	-1.417321000	1.755165000

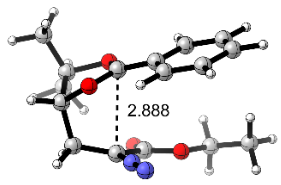

III

6	2.749138000	-0.559387000	-0.683758000
6	2.513332000	-0.409327000	0.834507000
6	0.653958000	-1.218158000	-0.093376000
6	2.133836000	0.982574000	1.402047000
1	2.379222000	0.983599000	2.471996000
1	2.761119000	1.749305000	0.928607000
6	0.684288000	1.353624000	1.220711000
1	3.332201000	-0.854728000	1.408405000
6	3.488405000	-1.845724000	-1.018656000
1	3.412266000	-2.050864000	-2.095061000
1	4.550099000	-1.721928000	-0.761155000
1	3.092454000	-2.705344000	-0.459414000
6	3.287158000	0.641313000	-1.424253000
1	4.283144000	0.884384000	-1.025617000
1	3.399128000	0.396500000	-2.489455000
1	2.628697000	1.510884000	-1.319899000

8	1.307270000	-0.773221000	-1.109345000
6	0.128776000	2.047208000	0.054076000
8	0.808806000	2.554759000	-0.816243000
8	-1.208250000	2.012417000	0.047321000
6	-1.889689000	2.590921000	-1.086758000
1	-1.451995000	2.172232000	-2.005600000
1	-1.707353000	3.676832000	-1.090901000
6	-3.358009000	2.257364000	-0.953478000
1	-3.912799000	2.683158000	-1.802925000
1	-3.510142000	1.167511000	-0.949297000
1	-3.773982000	2.673833000	-0.023602000
6	-0.733940000	-1.578803000	-0.159453000
6	-1.400341000	-2.020756000	1.003670000
6	-1.428667000	-1.429601000	-1.379531000
6	-2.758933000	-2.308006000	0.938871000
1	-0.851120000	-2.131633000	1.938565000
6	-2.787109000	-1.715572000	-1.426112000
1	-0.898332000	-1.081096000	-2.266204000
6	-3.449342000	-2.152857000	-0.270137000
1	-3.285841000	-2.649650000	1.830998000
1	-3.336906000	-1.597318000	-2.361006000
1	-4.517794000	-2.374587000	-0.312787000
7	-0.172273000	0.974790000	2.143745000
7	-0.908464000	0.645653000	2.936182000
8	1.339164000	-1.279339000	1.012595000

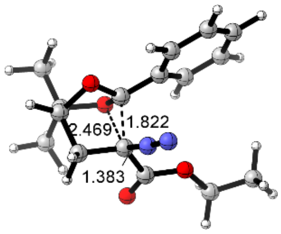

TS2

6	2.739432000	-0.406049000	-0.715593000
6	2.605032000	-0.488521000	0.819204000
6	0.620851000	-0.747780000	0.076405000
6	2.041262000	0.806533000	1.450174000
1	2.125261000	0.750375000	2.541059000
1	2.535534000	1.722643000	1.109746000
6	0.566769000	0.813925000	1.012383000
1	3.472526000	-0.906461000	1.337500000
6	3.298116000	-1.695123000	-1.304313000
1	3.123424000	-1.708387000	-2.389532000
1	4.383145000	-1.743033000	-1.127001000

1	2.825415000	-2.579103000	-0.856529000
6	3.422521000	0.824525000	-1.277813000
1	4.457726000	0.875589000	-0.909627000
1	3.452394000	0.759820000	-2.374866000
1	2.893634000	1.745488000	-1.006378000
8	1.287682000	-0.352773000	-1.041073000
6	0.042156000	1.932360000	0.114906000
8	0.766767000	2.696126000	-0.463240000
8	-1.273520000	1.867521000	0.058314000
6	-1.990020000	2.824679000	-0.773551000
1	-1.584924000	2.747241000	-1.793498000
1	-1.774595000	3.832999000	-0.390175000
6	-3.455680000	2.468254000	-0.705872000
1	-4.026907000	3.165477000	-1.338372000
1	-3.625174000	1.443377000	-1.072264000
1	-3.832934000	2.544722000	0.326224000
6	-0.708319000	-1.355048000	-0.135750000
6	-1.231135000	-2.245120000	0.813737000
6	-1.432536000	-1.042724000	-1.295779000
6	-2.481237000	-2.826151000	0.594502000
1	-0.651314000	-2.501924000	1.701585000
6	-2.681781000	-1.624314000	-1.503602000
1	-1.007998000	-0.361203000	-2.033091000
6	-3.206227000	-2.515223000	-0.559796000
1	-2.888125000	-3.526975000	1.325994000
1	-3.246935000	-1.386573000	-2.406884000
1	-4.184411000	-2.971335000	-0.726991000
7	-0.302262000	0.629536000	2.072846000
7	-1.044355000	0.395157000	2.870718000
8	1.479742000	-1.388490000	0.923837000

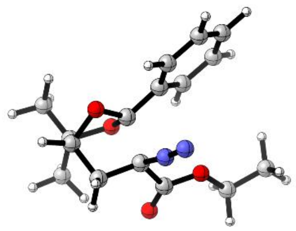

IV

6	2.718108000	-0.412769000	-0.736537000
6	2.591212000	-0.604190000	0.790903000
6	0.592030000	-0.742474000	0.065573000
6	2.045721000	0.654565000	1.504646000
1	2.105401000	0.523962000	2.590510000

1	2.546543000	1.589745000	1.233943000
6	0.578624000	0.690206000	1.026088000
1	3.454796000	-1.064130000	1.279507000
6	3.274357000	-1.658467000	-1.414731000
1	3.102656000	-1.591258000	-2.498464000
1	4.357944000	-1.728304000	-1.237262000
1	2.791217000	-2.568285000	-1.034889000
6	3.418593000	0.846898000	-1.209553000
1	4.456249000	0.850428000	-0.844077000
1	3.445088000	0.862579000	-2.308515000
1	2.910890000	1.756003000	-0.869109000
8	1.270549000	-0.323575000	-1.056602000
6	0.107651000	1.905937000	0.202805000
8	0.872928000	2.695348000	-0.276866000
8	-1.202862000	1.884052000	0.096203000
6	-1.855474000	2.920419000	-0.695888000
1	-1.402716000	2.907340000	-1.697698000
1	-1.632460000	3.888805000	-0.224804000
6	-3.331261000	2.605162000	-0.720323000
1	-3.857781000	3.374127000	-1.304702000
1	-3.514247000	1.625774000	-1.186504000
1	-3.747725000	2.596802000	0.297847000
6	-0.748823000	-1.336273000	-0.180669000
6	-1.290848000	-2.241101000	0.743108000
6	-1.477103000	-0.961299000	-1.318208000
6	-2.567542000	-2.764616000	0.527985000
1	-0.710048000	-2.544678000	1.615451000
6	-2.752259000	-1.487240000	-1.524626000
1	-1.040278000	-0.267916000	-2.037085000
6	-3.299363000	-2.385111000	-0.601028000
1	-2.989634000	-3.473394000	1.243129000
1	-3.320987000	-1.197505000	-2.410450000
1	-4.298224000	-2.795034000	-0.765700000
7	-0.329153000	0.484029000	2.079252000
7	-1.073283000	0.219789000	2.855095000
8	1.451714000	-1.487034000	0.844911000

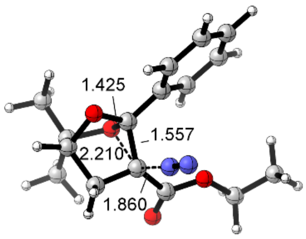

TS3

6	2.788399000	-0.379398000	-0.664995000
6	2.622013000	-0.458116000	0.869672000
6	0.624301000	-0.660383000	0.145345000
6	1.996132000	0.854855000	1.410942000
1	1.948685000	0.800018000	2.505391000
1	2.497037000	1.788896000	1.135067000
6	0.632123000	0.756683000	0.790180000
1	3.478747000	-0.837057000	1.433302000
6	3.348174000	-1.675923000	-1.237602000
1	3.206316000	-1.687257000	-2.327546000
1	4.425895000	-1.741345000	-1.026236000
1	2.844672000	-2.550393000	-0.805380000
6	3.506427000	0.843432000	-1.208225000
1	4.532461000	0.880239000	-0.813401000
1	3.564989000	0.777696000	-2.304022000
1	2.986266000	1.774791000	-0.954040000
8	1.356781000	-0.286592000	-1.018739000
6	0.007212000	1.925559000	0.036754000
8	0.703910000	2.770901000	-0.461541000
8	-1.297569000	1.813706000	-0.021381000
6	-2.044761000	2.801725000	-0.793537000
1	-1.613558000	2.825599000	-1.804582000
1	-1.884101000	3.782403000	-0.322461000
6	-3.492502000	2.376584000	-0.785908000
1	-4.085421000	3.101748000	-1.362560000
1	-3.611091000	1.384014000	-1.245012000
1	-3.887605000	2.342658000	0.240196000
6	-0.666535000	-1.353438000	-0.136587000
6	-1.188123000	-2.260064000	0.794492000
6	-1.363151000	-1.070775000	-1.318419000
6	-2.417097000	-2.874878000	0.545522000
1	-0.629800000	-2.491042000	1.702957000
6	-2.590836000	-1.688354000	-1.560601000
1	-0.941201000	-0.377225000	-2.046359000
6	-3.120664000	-2.586189000	-0.627756000
1	-2.823554000	-3.584365000	1.269087000
1	-3.134398000	-1.469192000	-2.481852000
1	-4.082560000	-3.066886000	-0.819048000
7	-0.572817000	0.608396000	2.199757000
7	-1.252407000	0.466961000	3.051469000
8	1.504140000	-1.359104000	0.965694000

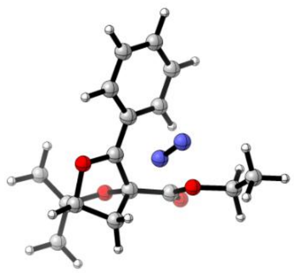

V

6	3.102458000	-0.354245000	-0.052409000
6	2.344262000	-0.241010000	1.294705000
6	0.388367000	-0.650379000	0.261050000
6	1.632119000	1.096916000	1.175740000
1	0.894494000	1.267195000	1.968060000
1	2.294532000	1.961281000	1.069065000
6	1.006960000	0.677277000	-0.168566000
1	2.878771000	-0.517585000	2.205890000
6	3.382601000	-1.781083000	-0.505598000
1	3.830029000	-1.757622000	-1.509205000
1	4.094685000	-2.262361000	0.180225000
1	2.470558000	-2.389864000	-0.549477000
6	4.372807000	0.489513000	-0.065900000
1	5.127006000	0.043314000	0.598321000
1	4.777123000	0.506726000	-1.087807000
1	4.192924000	1.525297000	0.248348000
8	2.106753000	0.238895000	-0.959962000
6	0.158571000	1.714426000	-0.881205000
8	0.432198000	2.198530000	-1.947884000
8	-0.900151000	2.000013000	-0.134164000
6	-1.844910000	2.975137000	-0.650166000
1	-2.139367000	2.662884000	-1.663268000
1	-1.324242000	3.940887000	-0.731080000
6	-3.018734000	3.029242000	0.298015000
1	-3.744970000	3.772148000	-0.063801000
1	-3.522248000	2.053067000	0.357273000
1	-2.697253000	3.322600000	1.308380000
6	-0.741393000	-1.370364000	-0.210786000
6	-1.136762000	-2.557230000	0.460975000
6	-1.477358000	-0.912544000	-1.332541000
6	-2.257966000	-3.248200000	0.030500000
1	-0.565606000	-2.901730000	1.323427000
6	-2.589184000	-1.623308000	-1.760885000
1	-1.156750000	-0.035045000	-1.890914000
6	-2.984251000	-2.780862000	-1.076209000
1	-2.575945000	-4.152778000	0.550834000

1	-3.151377000	-1.281029000	-2.630644000
1	-3.867079000	-3.329363000	-1.411610000
7	-1.629969000	0.311894000	2.476422000
7	-2.713357000	0.343577000	2.663810000
8	1.181510000	-1.161943000	1.147764000

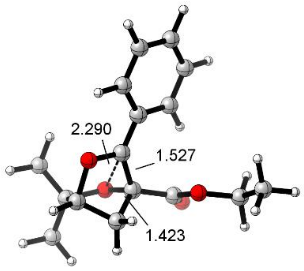

V' (no N_2)

6	2.875157000	-0.439446000	-0.210898000
6	2.310159000	-0.337903000	1.229308000
6	0.216987000	-0.636224000	0.461054000
6	1.647959000	1.030459000	1.240798000
1	1.032263000	1.209598000	2.130310000
1	2.327505000	1.870300000	1.066689000
6	0.830744000	0.677485000	-0.016894000
1	2.950113000	-0.663464000	2.052102000
6	3.027537000	-1.861790000	-0.733842000
1	3.337517000	-1.825515000	-1.787679000
1	3.802248000	-2.393418000	-0.162651000
1	2.091164000	-2.430513000	-0.669730000
6	4.168958000	0.351121000	-0.374605000
1	4.984959000	-0.147288000	0.168400000
1	4.431980000	0.385029000	-1.441250000
1	4.080371000	1.382631000	-0.011109000
8	1.794499000	0.222380000	-0.960287000
6	-0.063627000	1.768332000	-0.576876000
8	0.074228000	2.272629000	-1.660368000
8	-0.989952000	2.070579000	0.323013000
6	-1.962950000	3.091049000	-0.023699000
1	-2.426066000	2.810786000	-0.981728000
1	-1.423728000	4.038486000	-0.171805000
6	-2.968435000	3.166554000	1.100404000
1	-3.720296000	3.935311000	0.867891000
1	-3.485122000	2.203597000	1.229202000
1	-2.479764000	3.435419000	2.048883000
6	-0.995509000	-1.296479000	0.127115000
6	-1.349252000	-2.487704000	0.814708000
6	-1.845587000	-0.783991000	-0.885586000

6	-2.537758000	-3.130527000	0.506968000
1	-0.689229000	-2.875718000	1.590990000
6	-3.024928000	-1.447056000	-1.192461000
1	-1.563213000	0.096778000	-1.459429000
6	-3.374590000	-2.610394000	-0.492820000
1	-2.822074000	-4.038824000	1.040175000
1	-3.675309000	-1.063465000	-1.979598000
1	-4.309311000	-3.121800000	-0.732643000
8	1.099577000	-1.206694000	1.218510000

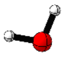

H_2O

8	0.000000000	0.121060000	0.000000000
1	0.755141000	-0.484235000	0.000000000
1	-0.755141000	-0.484241000	0.000000000

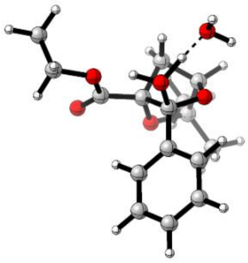

VI

6	0.620248000	2.404665000	-0.816455000
6	0.136757000	2.288946000	0.647335000
6	0.350666000	-0.006236000	0.666340000
6	-1.267905000	1.718817000	0.479791000
1	-1.739465000	1.431996000	1.425515000
1	-1.952441000	2.337832000	-0.107962000
6	-0.701418000	0.547279000	-0.351700000
1	0.282747000	3.172208000	1.276561000
6	2.132311000	2.342112000	-0.981876000
1	2.379938000	2.288778000	-2.052049000
1	2.594042000	3.250133000	-0.566106000
1	2.560690000	1.467953000	-0.479074000
6	0.043166000	3.612331000	-1.546360000
1	0.495187000	4.539698000	-1.164370000
1	0.276641000	3.531336000	-2.618011000
1	-1.047324000	3.684846000	-1.441382000
8	0.027614000	1.176256000	-1.378841000
6	-1.715337000	-0.424926000	-0.923411000
8	-2.047900000	-0.483361000	-2.073995000

8	-2.215844000	-1.201108000	0.062110000
6	-3.259146000	-2.168603000	-0.270110000
1	-3.159896000	-2.941498000	0.501700000
1	-3.014700000	-2.597632000	-1.250322000
6	-4.622985000	-1.512951000	-0.255857000
1	-5.392172000	-2.276665000	-0.447490000
1	-4.828545000	-1.052588000	0.721989000
1	-4.700603000	-0.744067000	-1.038189000
6	1.442642000	-0.906408000	0.150033000
6	2.558771000	-1.124897000	0.971668000
6	1.359793000	-1.543469000	-1.092832000
6	3.581382000	-1.974890000	0.552532000
1	2.624981000	-0.621483000	1.938007000
6	2.386100000	-2.396943000	-1.509219000
1	0.516886000	-1.364075000	-1.758900000
6	3.495946000	-2.614336000	-0.689901000
1	4.448636000	-2.139342000	1.195646000
1	2.316912000	-2.887304000	-2.482459000
1	4.297490000	-3.279302000	-1.019222000
8	0.845254000	1.167284000	1.250276000
8	-0.335474000	-0.741735000	1.761512000
1	-0.459538000	-0.126479000	2.703641000
1	-1.174666000	-1.136252000	1.399687000
8	-0.550812000	0.657191000	3.723696000
1	0.242710000	1.220849000	3.742970000
1	-0.596180000	0.201906000	4.581890000

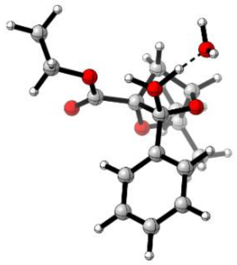

TS4

6	0.590678000	2.511558000	-0.580857000
6	0.151400000	2.233251000	0.876381000
6	0.356672000	-0.046248000	0.645416000
6	-1.259270000	1.685102000	0.684872000
1	-1.708610000	1.289740000	1.601227000
1	-1.957830000	2.365281000	0.188198000
6	-0.719332000	0.611143000	-0.283120000
1	0.315573000	3.042606000	1.594415000
6	2.097474000	2.474054000	-0.794916000

SUPPORTING INFORMATION

1	2.315323000	2.539564000	-1.870945000
1	2.569848000	3.332866000	-0.294861000
1	2.539975000	1.551619000	-0.402754000
6	-0.007630000	3.788343000	-1.160587000
1	0.454651000	4.670945000	-0.693979000
1	0.195284000	3.824378000	-2.240937000
1	-1.094727000	3.846141000	-1.017819000
8	-0.014846000	1.347436000	-1.255433000
6	-1.749495000	-0.293314000	-0.930284000
8	-2.091223000	-0.246098000	-2.079322000
8	-2.251497000	-1.149119000	-0.014548000
6	-3.299281000	-2.077809000	-0.429046000
1	-3.204139000	-2.916080000	0.271848000
1	-3.058335000	-2.421079000	-1.443402000
6	-4.660567000	-1.420324000	-0.357025000
1	-5.433180000	-2.160998000	-0.614238000
1	-4.864129000	-1.046004000	0.657336000
1	-4.734752000	-0.585835000	-1.069302000
6	1.432012000	-0.888799000	0.006534000
6	2.575971000	-1.179843000	0.764837000
6	1.309182000	-1.403733000	-1.288361000
6	3.585154000	-1.981425000	0.232620000
1	2.674175000	-0.769107000	1.771411000
6	2.322564000	-2.208065000	-1.819226000
1	0.443952000	-1.165349000	-1.905551000
6	3.459518000	-2.498776000	-1.062102000
1	4.473805000	-2.202473000	0.827916000
1	2.221944000	-2.602040000	-2.832764000
1	4.250753000	-3.124939000	-1.480456000
8	0.876961000	1.057399000	1.324121000
8	-0.306279000	-0.888727000	1.668268000
1	-0.258976000	-0.546299000	2.770794000
1	-1.170731000	-1.224651000	1.316595000
8	-0.135204000	-0.202062000	3.972247000
1	0.275983000	-0.899175000	4.512029000
1	-0.979456000	0.019562000	4.401759000

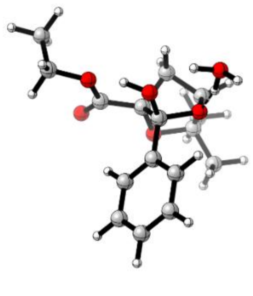

VII

6	0.435914000	2.092913000	-1.246263000
6	0.104824000	2.294936000	0.235383000
6	0.303105000	-0.140431000	0.698368000
6	-1.267918000	1.670527000	0.370829000
1	-1.607595000	1.580769000	1.397219000
1	-2.034210000	2.158494000	-0.220229000
6	-0.801004000	0.356933000	-0.286325000
1	0.286410000	3.281273000	0.644151000
6	1.918827000	2.011825000	-1.565198000
1	2.043386000	1.783256000	-2.622358000
1	2.392395000	2.972696000	-1.365118000
1	2.421286000	1.245968000	-0.984189000
6	-0.248712000	3.126596000	-2.128657000
1	0.215344000	4.102674000	-1.991531000
1	-0.129528000	2.833946000	-3.170524000
1	-1.312149000	3.213283000	-1.920466000
8	-0.183216000	0.774074000	-1.482930000
6	-1.905194000	-0.652982000	-0.558583000
8	-2.164725000	-1.108564000	-1.638012000
8	-2.536614000	-0.930568000	0.583579000
6	-3.641793000	-1.888388000	0.543655000
1	-3.659652000	-2.304518000	1.546254000
1	-3.385403000	-2.666682000	-0.169946000
6	-4.935840000	-1.192338000	0.192631000
1	-5.750242000	-1.916359000	0.227181000
1	-5.153800000	-0.397083000	0.904461000
1	-4.895741000	-0.770789000	-0.810403000
6	1.430330000	-0.977367000	0.177127000
6	2.616375000	-1.046087000	0.908109000
6	1.279850000	-1.747795000	-0.972167000
6	3.648315000	-1.869491000	0.484081000
1	2.741281000	-0.452327000	1.802272000
6	2.313751000	-2.578783000	-1.387905000
1	0.373349000	-1.701196000	-1.554145000
6	3.498678000	-2.638396000	-0.666100000
1	4.567881000	-1.910813000	1.050802000
1	2.190934000	-3.173592000	-2.282103000
1	4.303873000	-3.279441000	-0.997342000
8	0.943943000	1.324647000	0.973698000
8	-0.125606000	-0.606996000	1.897244000
1	0.839475000	1.033955000	3.957671000
1	-1.085499000	-0.505761000	1.991107000
8	0.987798000	1.821194000	3.419607000

1	0.969210000	1.522529000	2.003492000
1	1.799303000	2.229401000	3.744351000

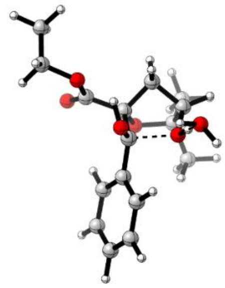

TS5

6	0.307033000	2.242813000	-1.019647000
6	0.034156000	2.270279000	0.497318000
6	0.269002000	-0.314544000	0.634953000
6	-1.307525000	1.557468000	0.601389000
1	-1.605658000	1.324226000	1.629661000
1	-2.132596000	2.078744000	0.104547000
6	-0.826833000	0.350303000	-0.238178000
1	0.130970000	3.251053000	0.977173000
6	1.782389000	2.268246000	-1.394250000
1	1.882493000	2.133161000	-2.480862000
1	2.221219000	3.240693000	-1.125632000
1	2.343848000	1.475553000	-0.888258000
6	-0.465328000	3.321054000	-1.772090000
1	-0.040674000	4.313382000	-1.560458000
1	-0.382753000	3.132507000	-2.852064000
1	-1.531789000	3.332621000	-1.510477000
8	-0.250534000	0.927706000	-1.376586000
6	-1.931078000	-0.635464000	-0.618366000
8	-2.209945000	-0.955150000	-1.741406000
8	-2.525400000	-1.069381000	0.497398000
6	-3.620811000	-2.021780000	0.374616000
1	-3.612313000	-2.563996000	1.328074000
1	-3.377308000	-2.715645000	-0.441067000
6	-4.933133000	-1.304972000	0.136503000
1	-5.750389000	-2.042047000	0.113260000
1	-5.139503000	-0.584651000	0.942276000
1	-4.923265000	-0.772722000	-0.825875000
6	1.454841000	-0.981241000	0.046278000
6	2.551328000	-1.250053000	0.883688000
6	1.468350000	-1.407715000	-1.289891000
6	3.659844000	-1.926469000	0.382086000
1	2.536966000	-0.907920000	1.919649000

6	2.577164000	-2.102452000	-1.779292000
1	0.624188000	-1.200296000	-1.944427000
6	3.672869000	-2.356172000	-0.951078000
1	4.516289000	-2.121220000	1.030604000
1	2.583623000	-2.440662000	-2.817355000
1	4.541574000	-2.889581000	-1.343200000
8	0.962013000	1.332456000	1.087930000
8	-0.117186000	-0.854755000	1.778760000
1	0.839747000	0.425270000	3.953402000
1	-1.072634000	-0.717140000	1.935352000
8	1.155550000	1.275281000	3.607217000
1	1.018896000	1.370731000	2.118515000
1	2.059496000	1.372891000	3.946425000

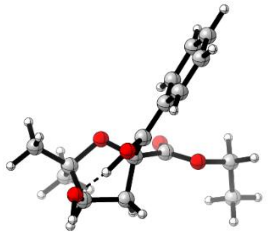

VIII

6	-2.724421000	-0.623759000	0.717288000
6	-2.854272000	-0.405651000	-0.805637000
6	0.322815000	-0.885908000	-0.633181000
6	-1.744508000	0.594347000	-1.110245000
1	-1.413048000	0.578699000	-2.155586000
1	-2.078290000	1.615688000	-0.888389000
6	-0.639148000	0.188226000	-0.097648000
1	-3.852169000	-0.042673000	-1.079122000
6	-3.112184000	-2.014471000	1.190728000
1	-2.936527000	-2.095133000	2.272982000
1	-4.179504000	-2.195545000	0.997190000
1	-2.527430000	-2.786985000	0.676590000
6	-3.467759000	0.467245000	1.486717000
1	-4.554738000	0.348768000	1.368505000
1	-3.218966000	0.388233000	2.554175000
1	-3.182517000	1.472642000	1.147413000
8	-1.288714000	-0.452867000	0.973591000
6	0.052997000	1.483873000	0.345873000
8	-0.186865000	2.053264000	1.379474000
8	0.869975000	1.895926000	-0.614741000
6	1.561144000	3.161159000	-0.434307000
1	2.453436000	3.073063000	-1.066608000

1	1.868798000	3.236967000	0.617650000
6	0.682284000	4.322907000	-0.850193000
1	1.252760000	5.260695000	-0.767299000
1	0.351278000	4.210488000	-1.893611000
1	-0.202209000	4.400461000	-0.201121000
6	1.673050000	-1.058330000	-0.152664000
6	2.565881000	-1.864600000	-0.898688000
6	2.098294000	-0.489066000	1.070198000
6	3.863837000	-2.061639000	-0.447378000
1	2.231143000	-2.305810000	-1.837597000
6	3.391872000	-0.714985000	1.524924000
1	1.409545000	0.083416000	1.689842000
6	4.277177000	-1.488018000	0.763190000
1	4.559326000	-2.664305000	-1.033739000
1	3.713035000	-0.290533000	2.477312000
1	5.297248000	-1.649575000	1.118389000
8	-2.590812000	-1.637041000	-1.511370000
8	-0.111947000	-1.728943000	-1.478214000
1	-1.154970000	-1.711043000	-1.635210000
1	-3.051648000	-1.638215000	-2.365541000

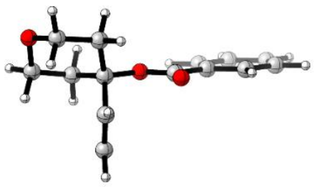

22sm

6	-1.757798000	1.161770000	1.128762000
6	0.840027000	0.667948000	-0.155886000
6	-1.621229000	1.040451000	2.450719000
1	-1.803347000	1.887991000	3.116789000
1	-1.308474000	0.103573000	2.921319000
1	-2.056908000	2.125050000	0.706044000
8	-0.066962000	-0.281741000	0.102482000
6	2.235652000	0.138208000	-0.121323000
6	3.287450000	1.028793000	-0.387919000
6	2.517673000	-1.206423000	0.168532000
6	4.607956000	0.580800000	-0.365672000
1	3.049603000	2.070097000	-0.610639000
6	3.840465000	-1.651600000	0.190150000
1	1.697911000	-1.894261000	0.375792000
6	4.885656000	-0.760309000	-0.076580000
1	5.423761000	1.276681000	-0.573659000
1	4.058047000	-2.697893000	0.416047000

1	5.920020000	-1.111801000	-0.058905000
8	0.564794000	1.827665000	-0.389196000
6	-1.494029000	0.044787000	0.129452000
6	-1.977407000	0.415629000	-1.280258000
6	-2.160193000	-1.278699000	0.530051000
6	-3.504811000	0.391374000	-1.351404000
1	-1.570700000	-0.322353000	-1.989390000
1	-1.601190000	1.407098000	-1.566332000
6	-3.675535000	-1.206587000	0.359289000
1	-1.758857000	-2.069254000	-0.122949000
1	-1.904642000	-1.541950000	1.567025000
1	-3.839217000	0.578736000	-2.382198000
1	-3.928737000	1.193037000	-0.713063000
1	-4.130685000	-2.184955000	0.570752000
1	-4.102712000	-0.473022000	1.071548000
8	-4.033724000	-0.863876000	-0.967745000

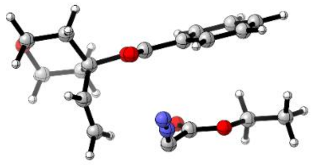

TS1(22)

6	2.385392000	1.227112000	0.494260000
6	0.322897000	-0.812166000	0.868110000
6	1.878840000	2.289941000	-0.139546000
1	1.858930000	3.270097000	0.342222000
1	1.454055000	2.223342000	-1.143298000
6	-1.015751000	2.474515000	0.434729000
1	2.771348000	1.344132000	1.510524000
8	1.128473000	-0.769159000	-0.195405000
6	-1.557263000	1.692451000	-0.689435000
8	-0.908803000	1.371082000	-1.659623000
8	-2.861778000	1.472568000	-0.524369000
6	-3.577400000	0.869205000	-1.632308000
1	-3.090647000	-0.083855000	-1.878686000
1	-3.488107000	1.536185000	-2.503947000
6	-5.012003000	0.678140000	-1.200732000
1	-5.583687000	0.218608000	-2.021142000
1	-5.068136000	0.013918000	-0.325911000
1	-5.481397000	1.640625000	-0.946827000
6	-1.006706000	-1.409182000	0.548024000
6	-2.007459000	-1.365278000	1.530988000
6	-1.273968000	-2.005711000	-0.694332000

6	-3.264132000	-1.913013000	1.275105000
1	-1.784485000	-0.893247000	2.488763000
6	-2.529143000	-2.562497000	-0.942841000
1	-0.496568000	-2.025287000	-1.457500000
6	-3.524709000	-2.516112000	0.039600000
1	-4.043317000	-1.870680000	2.039112000
1	-2.735086000	-3.029643000	-1.908284000
1	-4.508316000	-2.947090000	-0.160507000
7	-1.061578000	2.154909000	1.655189000
7	-1.029567000	1.987649000	2.797778000
8	0.625413000	-0.395921000	1.969258000
6	2.463433000	-0.176692000	-0.088498000
6	3.369210000	-1.085504000	0.754409000
6	2.978824000	-0.190278000	-1.533558000
6	4.838081000	-0.701206000	0.570314000
1	3.222753000	-2.124580000	0.420518000
1	3.091429000	-1.024909000	1.815805000
6	4.467352000	0.143535000	-1.589628000
1	2.819022000	-1.201809000	-1.938133000
1	2.401666000	0.510749000	-2.153919000
1	5.485564000	-1.405858000	1.112217000
1	5.022729000	0.309439000	0.987731000
1	4.842200000	0.046825000	-2.618799000
1	4.634273000	1.189611000	-1.264948000
8	5.229298000	-0.743325000	-0.789077000

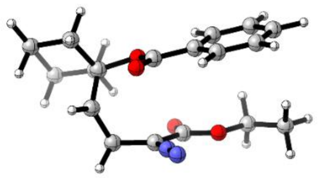

II(22)

6	2.221262000	0.634550000	1.081900000
6	0.088425000	-1.278252000	0.602573000
6	1.470591000	1.928951000	1.056744000
1	1.743086000	2.529727000	1.936694000
1	1.730844000	2.521662000	0.164793000
6	-0.044866000	1.791218000	0.986126000
1	2.680469000	0.320593000	2.020667000
8	0.874668000	-0.813579000	-0.372535000
6	-0.781472000	1.948425000	-0.264773000
8	-0.257137000	2.246170000	-1.322693000
8	-2.096919000	1.725716000	-0.114588000
6	-2.927500000	1.850458000	-1.283905000

1	-2.578971000	1.132157000	-2.041173000
1	-2.805371000	2.862750000	-1.699947000
6	-4.353155000	1.575455000	-0.863369000
1	-5.019955000	1.654930000	-1.735266000
1	-4.444731000	0.561231000	-0.448430000
1	-4.685848000	2.299925000	-0.104304000
6	-1.287337000	-1.587647000	0.115594000
6	-2.258412000	-1.949117000	1.061539000
6	-1.628277000	-1.518700000	-1.244287000
6	-3.559521000	-2.239589000	0.652154000
1	-1.976060000	-1.990212000	2.114394000
6	-2.928896000	-1.817509000	-1.651545000
1	-0.871745000	-1.226835000	-1.972463000
6	-3.894952000	-2.177400000	-0.705335000
1	-4.315373000	-2.514919000	1.390864000
1	-3.193010000	-1.763989000	-2.709912000
1	-4.913831000	-2.405761000	-1.026569000
7	-0.714891000	1.452602000	2.055476000
7	-1.273330000	1.151919000	2.996089000
8	0.438962000	-1.408804000	1.759388000
6	2.237063000	-0.330856000	-0.064937000
6	3.156077000	-1.527826000	0.211660000
6	2.677123000	0.323175000	-1.384846000
6	4.623883000	-1.101212000	0.149121000
1	2.966447000	-2.292840000	-0.557368000
1	2.928989000	-1.966438000	1.193563000
6	4.165234000	0.657204000	-1.345181000
1	2.488699000	-0.397003000	-2.196404000
1	2.070620000	1.216547000	-1.590497000
1	5.279667000	-1.977574000	0.257399000
1	4.852563000	-0.407220000	0.983084000
1	4.496081000	1.057319000	-2.314534000
1	4.362341000	1.430946000	-0.575351000
8	4.949302000	-0.495080000	-1.087771000

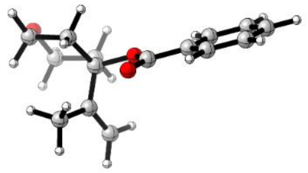

3sm

6	-1.664220000	1.137073000	0.982401000
6	0.941334000	0.536990000	-0.310291000
6	-1.348821000	1.113151000	2.284759000

1	-1.505659000	1.991156000	2.917137000
1	-0.901049000	0.238307000	2.762111000
8	0.067809000	-0.372661000	0.133438000
6	2.354189000	0.073148000	-0.170345000
6	3.376601000	0.922986000	-0.620771000
6	2.680228000	-1.170251000	0.394631000
6	4.711381000	0.534665000	-0.508698000
1	3.104681000	1.885690000	-1.056336000
6	4.017177000	-1.555846000	0.505819000
1	1.883099000	-1.826388000	0.744092000
6	5.032908000	-0.705591000	0.054884000
1	5.504209000	1.198540000	-0.860488000
1	4.268923000	-2.523271000	0.946081000
1	6.078460000	-1.010246000	0.143107000
8	0.631834000	1.616027000	-0.773939000
6	-1.375697000	-0.108032000	0.119363000
6	-1.860618000	-0.030058000	-1.339910000
6	-1.962076000	-1.391353000	0.730908000
6	-3.369507000	-0.260046000	-1.476915000
1	-1.338669000	-0.829159000	-1.889466000
1	-1.581527000	0.922122000	-1.806095000
6	-3.469823000	-1.470620000	0.524059000
1	-1.491651000	-2.248770000	0.224827000
1	-1.717260000	-1.459429000	1.799682000
1	-3.633563000	-0.342078000	-2.541488000
1	-3.935856000	0.595591000	-1.063035000
1	-3.863789000	-2.410384000	0.937168000
1	-3.974610000	-0.633692000	1.048201000
8	-3.795009000	-1.455908000	-0.853851000
6	-2.231272000	2.379114000	0.347672000
1	-1.565268000	2.735659000	-0.451254000
1	-3.221353000	2.207543000	-0.100787000
1	-2.336908000	3.177149000	1.095968000

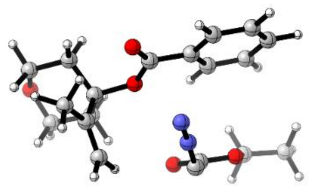

TS1(3)

6	2.461626000	0.114598000	1.242292000
6	0.353463000	-1.616544000	-0.190284000
6	1.946493000	1.124858000	1.957744000
1	2.097326000	1.170424000	3.040025000

1	1.341832000	1.915142000	1.511281000
6	-1.440244000	1.151486000	2.173955000
8	0.805923000	-0.397738000	-0.493560000
6	-1.664368000	1.836345000	0.894701000
8	-0.790061000	2.443813000	0.315744000
8	-2.939922000	1.780628000	0.533809000
6	-3.331077000	2.522532000	-0.649022000
1	-2.798802000	2.101307000	-1.515027000
1	-3.002978000	3.565634000	-0.528113000
6	-4.830548000	2.403776000	-0.787438000
1	-5.160898000	2.947801000	-1.684936000
1	-5.133867000	1.350982000	-0.888006000
1	-5.340868000	2.833548000	0.087493000
6	-1.089543000	-1.767043000	-0.549653000
6	-1.766523000	-2.915533000	-0.111469000
6	-1.773164000	-0.798940000	-1.301222000
6	-3.116241000	-3.090821000	-0.414631000
1	-1.218681000	-3.656750000	0.472005000
6	-3.121116000	-0.983229000	-1.613147000
1	-1.239106000	0.089041000	-1.639161000
6	-3.794859000	-2.124909000	-1.167303000
1	-3.642487000	-3.982039000	-0.065752000
1	-3.649649000	-0.235293000	-2.207492000
1	-4.851404000	-2.263297000	-1.408241000
7	-1.085573000	-0.035846000	2.375495000
7	-0.758757000	-1.098147000	2.703511000
8	1.010513000	-2.496541000	0.327002000
6	2.196404000	0.029099000	-0.273964000
6	3.164242000	-0.890886000	-1.036161000
6	2.231244000	1.397515000	-0.972836000
6	4.542033000	-0.240680000	-1.199161000
1	2.736911000	-1.061907000	-2.036851000
1	3.264479000	-1.868967000	-0.552523000
6	3.654382000	1.926551000	-1.096403000
1	1.819791000	1.256370000	-1.984497000
1	1.579722000	2.114694000	-0.459145000
1	5.179125000	-0.875068000	-1.832768000
1	5.043285000	-0.146532000	-0.215923000
1	3.662595000	2.879500000	-1.645211000
1	4.084089000	2.110414000	-0.091444000
8	4.473502000	1.026498000	-1.822658000
6	3.256441000	-0.974720000	1.916449000
1	2.792490000	-1.954386000	1.736683000
1	4.288935000	-1.029367000	1.537263000

1	3.306948000	-0.797316000	2.999911000

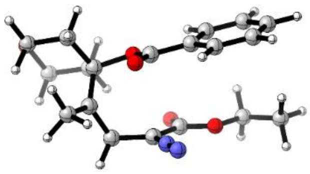

II(**3**)

6	-2.163366000	-0.693028000	0.871392000
6	-0.011843000	1.279029000	0.582394000
6	-1.391233000	-1.973778000	0.690064000
1	-1.708645000	-2.699700000	1.454373000
1	-1.585617000	-2.434280000	-0.288889000
6	0.123087000	-1.822851000	0.740001000
8	-0.746820000	0.835480000	-0.439416000
6	0.949130000	-1.892187000	-0.463093000
8	0.503508000	-2.106625000	-1.575735000
8	2.251208000	-1.696019000	-0.201015000
6	3.165850000	-1.759314000	-1.310998000
1	2.877245000	-0.996234000	-2.049170000
1	3.070743000	-2.745062000	-1.792849000
6	4.557644000	-1.521080000	-0.771488000
1	5.286779000	-1.556690000	-1.595049000
1	4.622764000	-0.532895000	-0.293433000
1	4.830115000	-2.290285000	-0.032693000
6	1.388003000	1.594319000	0.171575000
6	2.314744000	1.917800000	1.173960000
6	1.792213000	1.571881000	-1.172276000
6	3.634566000	2.216465000	0.836574000
1	1.982962000	1.924297000	2.213203000
6	3.111121000	1.880068000	-1.507580000
1	1.070063000	1.308485000	-1.944835000
6	4.032956000	2.201616000	-0.505180000
1	4.355835000	2.461736000	1.619173000
1	3.424075000	1.863965000	-2.553817000
1	5.066264000	2.437154000	-0.770214000
7	0.718702000	-1.575202000	1.875834000
7	1.219557000	-1.353010000	2.870209000
8	-0.419522000	1.393919000	1.721797000
6	-2.131310000	0.349120000	-0.223449000
6	-3.049284000	1.556225000	0.010768000
6	-2.492407000	-0.236288000	-1.600455000
6	-4.520008000	1.171188000	-0.170871000
1	-2.786592000	2.319425000	-0.737939000

1	-2.884718000	1.998307000	1.001648000
6	-3.980882000	-0.558058000	-1.671304000
1	-2.254301000	0.524229000	-2.360180000
1	-1.877743000	-1.118242000	-1.823837000
1	-5.155615000	2.065805000	-0.096600000
1	-4.835937000	0.470606000	0.626572000
1	-4.247871000	-0.931028000	-2.670763000
1	-4.237041000	-1.348126000	-0.935957000
8	-4.768494000	0.597147000	-1.440674000
6	-2.742396000	-0.469515000	2.230225000
1	-3.276004000	0.480834000	2.337560000
1	-3.438487000	-1.286698000	2.494556000
1	-1.944642000	-0.479014000	2.995630000

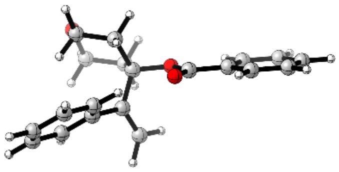

7sm

6	1.019923000	-0.045247000	0.980389000
6	-1.648517000	-0.377687000	-0.308213000
6	0.544610000	-0.172627000	2.227355000
1	0.994077000	-0.877623000	2.930542000
1	-0.323160000	0.395513000	2.572746000
8	-1.094526000	0.781550000	0.066020000
6	-3.130283000	-0.375268000	-0.132979000
6	-3.842168000	-1.526924000	-0.503947000
6	-3.818233000	0.731872000	0.389348000
6	-5.227947000	-1.572263000	-0.354700000
1	-3.291087000	-2.377824000	-0.907306000
6	-5.205244000	0.682964000	0.537575000
1	-3.261137000	1.623586000	0.676616000
6	-5.910780000	-0.467003000	0.166341000
1	-5.778793000	-2.469894000	-0.644202000
1	-5.738969000	1.544734000	0.944406000
1	-6.996475000	-0.502217000	0.283630000
8	-1.023667000	-1.325968000	-0.738808000
6	0.353510000	0.965997000	0.023649000
6	0.834659000	0.948019000	-1.438095000
6	0.536708000	2.403167000	0.546691000
6	2.215830000	1.585819000	-1.611150000
1	0.102637000	1.535906000	-2.014019000
1	0.839692000	-0.065664000	-1.851354000

6	1.955438000	2.908754000	0.307356000
1	-0.166739000	3.050867000	0.000205000
1	0.285073000	2.453309000	1.615385000
1	2.446968000	1.673765000	-2.682859000
1	2.994478000	0.950256000	-1.152425000
1	2.049442000	3.950019000	0.647958000
1	2.684509000	2.302502000	0.880429000
8	2.277728000	2.893709000	-1.070760000
6	2.216223000	-0.833520000	0.563722000
6	3.447501000	-0.619459000	1.206923000
6	2.141880000	-1.818177000	-0.437552000
6	4.582386000	-1.349757000	0.844126000
1	3.513244000	0.138209000	1.990932000
6	3.276888000	-2.547573000	-0.799231000
1	1.179309000	-2.017213000	-0.907703000
6	4.502264000	-2.312060000	-0.166554000
1	5.532186000	-1.163388000	1.351233000
1	3.201602000	-3.312006000	-1.576636000
1	5.388976000	-2.881327000	-0.455518000

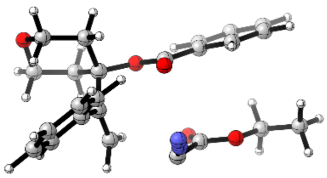

TS1(7)

6	1.724732000	-0.033718000	0.756403000
6	-0.774651000	0.781872000	-0.732184000
6	1.159996000	-0.510473000	1.889183000
1	1.489157000	-1.459053000	2.313764000
1	0.401429000	0.047659000	2.438920000
6	-0.729958000	-2.164154000	1.163314000
8	-0.092293000	1.522385000	0.145117000
6	-1.939918000	-1.375149000	1.403491000
8	-1.997168000	-0.411656000	2.138489000
8	-2.995513000	-1.906273000	0.771418000
6	-4.289397000	-1.326911000	1.063096000
1	-4.254793000	-0.255684000	0.824341000
1	-4.479076000	-1.426859000	2.143351000
6	-5.317533000	-2.059872000	0.233818000
1	-6.315269000	-1.637917000	0.427715000
1	-5.101129000	-1.951602000	-0.839211000
1	-5.338345000	-3.131539000	0.483866000
6	-2.214821000	1.156473000	-0.805678000

6	-3.024364000	0.473632000	-1.726200000
6	-2.769005000	2.158533000	0.005347000
6	-4.375727000	0.796770000	-1.843715000
1	-2.576776000	-0.310486000	-2.338072000
6	-4.123183000	2.474867000	-0.110182000
1	-2.136432000	2.676635000	0.725533000
6	-4.926229000	1.798024000	-1.035660000
1	-5.003554000	0.265525000	-2.561989000
1	-4.555888000	3.251764000	0.523756000
1	-5.985842000	2.048682000	-1.123809000
7	-0.363272000	-2.664594000	0.060731000
7	0.092017000	-3.169233000	-0.870312000
8	-0.278874000	-0.101462000	-1.404164000
6	1.355851000	1.381550000	0.266477000
6	2.022598000	1.849656000	-1.039438000
6	1.715792000	2.418107000	1.349488000
6	3.507626000	2.174923000	-0.856158000
1	1.498279000	2.769325000	-1.342753000
1	1.899470000	1.121167000	-1.846969000
6	3.221627000	2.648605000	1.422927000
1	1.224148000	3.365438000	1.078754000
1	1.325897000	2.102092000	2.326825000
1	3.899234000	2.627841000	-1.778652000
1	4.085224000	1.252200000	-0.666298000
1	3.450063000	3.421109000	2.171183000
1	3.740833000	1.719654000	1.731878000
8	3.729324000	3.108996000	0.184742000
6	2.769803000	-0.850238000	0.082371000
6	3.898199000	-1.262124000	0.816528000
6	2.651634000	-1.266676000	-1.257372000
6	4.891878000	-2.044279000	0.225530000
1	4.001955000	-0.944144000	1.856142000
6	3.644632000	-2.054175000	-1.844125000
1	1.758862000	-0.996653000	-1.817248000
6	4.770666000	-2.439630000	-1.110626000
1	5.765825000	-2.342289000	0.809570000
1	3.532012000	-2.375646000	-2.882339000
1	5.548219000	-3.050031000	-1.575847000

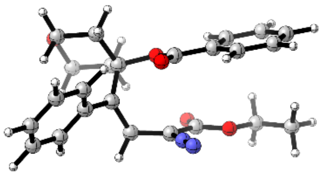

II(7)

6	-1.651587000	-0.101009000	-0.515896000
6	0.651492000	0.280896000	1.323123000
6	-0.905665000	-0.617538000	-1.724627000
1	-1.459685000	-1.457644000	-2.164325000
1	-0.801684000	0.145534000	-2.509416000
6	0.519915000	-1.061690000	-1.444667000
8	0.190552000	1.207081000	0.479713000
6	1.677440000	-0.311773000	-1.924620000
8	1.591593000	0.711864000	-2.578356000
8	2.839563000	-0.879557000	-1.565612000
6	4.051969000	-0.239419000	-2.005776000
1	4.066369000	0.791126000	-1.621289000
1	4.045130000	-0.187327000	-3.105909000
6	5.214364000	-1.050853000	-1.481146000
1	6.162343000	-0.587978000	-1.794555000
1	5.193865000	-1.088835000	-0.382322000
1	5.184304000	-2.079670000	-1.871461000
6	2.127257000	0.378550000	1.512614000
6	2.765347000	-0.621244000	2.262208000
6	2.878951000	1.430838000	0.967253000
6	4.143880000	-0.569873000	2.465668000
1	2.164534000	-1.433737000	2.673202000
6	4.256942000	1.483780000	1.179851000
1	2.378539000	2.197012000	0.375570000
6	4.890324000	0.484971000	1.927582000
1	4.639667000	-1.352191000	3.044391000
1	4.841159000	2.303798000	0.756781000
1	5.970092000	0.526533000	2.087989000
7	0.732157000	-2.139055000	-0.735858000
7	0.886717000	-3.070384000	-0.106881000
8	-0.040580000	-0.560171000	1.861958000
6	-1.223391000	1.234746000	0.073094000
6	-2.072515000	1.772001000	1.237005000
6	-1.228031000	2.336678000	-1.009175000
6	-3.452781000	2.240212000	0.767103000
1	-1.528443000	2.635314000	1.650379000
1	-2.180843000	1.039142000	2.042247000
6	-2.653472000	2.718047000	-1.391559000

1	-0.727120000	3.218668000	-0.580951000
1	-0.641941000	2.029317000	-1.884684000
1	-3.984134000	2.720373000	1.601901000
1	-4.059209000	1.376826000	0.437135000
1	-2.645899000	3.525530000	-2.137958000
1	-3.179074000	1.850930000	-1.840213000
8	-3.370893000	3.201309000	-0.270256000
6	-2.809740000	-0.873251000	-0.057777000
6	-3.764187000	-1.313563000	-1.007863000
6	-3.028414000	-1.224416000	1.294672000
6	-4.896925000	-2.028254000	-0.621757000
1	-3.632041000	-1.056247000	-2.061056000
6	-4.159243000	-1.946832000	1.675489000
1	-2.267573000	-0.972607000	2.029206000
6	-5.105334000	-2.345406000	0.725209000
1	-5.624831000	-2.334775000	-1.376888000
1	-4.295311000	-2.214017000	2.726498000
1	-5.991784000	-2.906796000	1.029293000

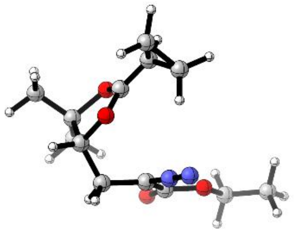

III(104)

6	-1.962971000	-1.596984000	-0.316932000
6	-1.616561000	-0.954678000	1.053008000
6	-1.738187000	0.655480000	-0.528556000
6	-0.231516000	-1.162734000	1.689036000
1	-0.333950000	-0.953425000	2.763274000
1	0.042028000	-2.222671000	1.599149000
6	0.901420000	-0.343638000	1.126274000
1	-2.391792000	-1.203541000	1.786214000
6	-3.452016000	-1.889131000	-0.429405000
1	-3.711287000	-2.139091000	-1.467080000
1	-3.693848000	-2.750186000	0.210252000
1	-4.060850000	-1.032983000	-0.103201000
6	-1.102316000	-2.745039000	-0.786660000
1	-1.263878000	-3.604387000	-0.119362000
1	-1.412596000	-3.041351000	-1.798223000
1	-0.034563000	-2.499691000	-0.784723000
8	-1.708472000	-0.418029000	-1.227664000
6	1.948833000	-0.874557000	0.247777000

8	1.973976000	-2.025802000	-0.145477000
8	2.863776000	0.048821000	-0.064226000
6	3.954924000	-0.361483000	-0.916374000
1	3.537280000	-0.747655000	-1.858975000
1	4.492118000	-1.187956000	-0.425987000
6	4.842685000	0.841794000	-1.137732000
1	5.687564000	0.563402000	-1.785360000
1	4.286017000	1.656543000	-1.625267000
1	5.245389000	1.214185000	-0.183538000
7	1.065418000	0.880481000	1.572534000
7	1.201512000	1.933820000	1.963690000
8	-1.786301000	0.480492000	0.755345000
6	-1.751395000	1.956104000	-1.136805000
6	-2.606946000	3.053429000	-0.468653000
6	-1.138956000	3.154478000	-0.399513000
1	-1.706837000	1.926392000	-2.225374000
1	-3.154311000	2.758742000	0.429304000
1	-3.158042000	3.676149000	-1.176139000
1	-0.620751000	3.857480000	-1.054826000
1	-0.642018000	2.947840000	0.549668000

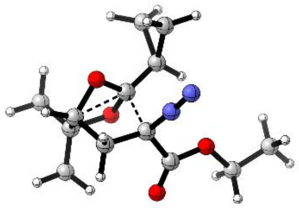

TS2(104)

6	-2.287944000	-0.939661000	-0.442570000
6	-2.158192000	-0.331531000	0.969591000
6	-0.697753000	0.687059000	-0.208985000
6	-0.948228000	-0.872903000	1.768016000
1	-1.004869000	-0.513091000	2.801338000
1	-0.877515000	-1.965733000	1.782438000
6	0.271996000	-0.279151000	1.048075000
1	-3.090525000	-0.289438000	1.539829000
6	-3.510161000	-0.405425000	-1.177871000
1	-3.426021000	-0.639869000	-2.248528000
1	-4.418328000	-0.887011000	-0.786104000
1	-3.607988000	0.682028000	-1.059498000
6	-2.176143000	-2.447342000	-0.551234000
1	-2.996473000	-2.914735000	0.013570000
1	-2.275234000	-2.747540000	-1.604170000
1	-1.217414000	-2.821048000	-0.176920000

8	-1.082328000	-0.303422000	-1.048490000
6	1.244008000	-1.201406000	0.321114000
8	1.011334000	-2.364558000	0.126448000
8	2.297305000	-0.520116000	-0.088588000
6	3.317088000	-1.233568000	-0.847105000
1	2.828365000	-1.682426000	-1.723877000
1	3.700566000	-2.044999000	-0.211495000
6	4.384833000	-0.236157000	-1.224631000
1	5.174320000	-0.748695000	-1.794069000
1	3.969940000	0.566175000	-1.853058000
1	4.839260000	0.212908000	-0.328964000
7	0.960679000	0.649886000	1.800893000
7	1.489255000	1.455203000	2.354307000
8	-1.719519000	1.004038000	0.631238000
6	0.080577000	1.791784000	-0.806160000
6	-0.684095000	2.961101000	-1.389779000
6	0.112773000	3.144058000	-0.133797000
1	0.968688000	1.446845000	-1.338695000
1	-1.772766000	2.940589000	-1.302932000
1	-0.300374000	3.370872000	-2.326433000
1	1.055519000	3.693128000	-0.179691000
1	-0.445569000	3.257730000	0.798027000

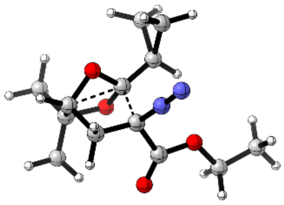

IV(104)

6	-2.279137000	-0.934611000	-0.448480000
6	-2.166528000	-0.320880000	0.964544000
6	-0.646282000	0.656634000	-0.162260000
6	-0.955595000	-0.862358000	1.758782000
1	-0.997606000	-0.496486000	2.790350000
1	-0.862239000	-1.952926000	1.773564000
6	0.235468000	-0.239798000	0.997197000
1	-3.100685000	-0.280867000	1.532113000
6	-3.489412000	-0.393449000	-1.199476000
1	-3.392807000	-0.629853000	-2.268842000
1	-4.407851000	-0.865632000	-0.819972000
1	-3.577062000	0.695162000	-1.084825000
6	-2.189214000	-2.446315000	-0.543489000
1	-3.024045000	-2.900666000	0.010766000
1	-2.274426000	-2.753132000	-1.595904000

1	-1.241763000	-2.832339000	-0.152317000
8	-1.062245000	-0.323851000	-1.038078000
6	1.238636000	-1.191899000	0.317171000
8	1.027504000	-2.366043000	0.190407000
8	2.276900000	-0.508942000	-0.115947000
6	3.321368000	-1.233671000	-0.834343000
1	2.850494000	-1.719945000	-1.700684000
1	3.705184000	-2.014986000	-0.162603000
6	4.378613000	-0.233236000	-1.231099000
1	5.184097000	-0.754011000	-1.769787000
1	3.961544000	0.539097000	-1.894509000
1	4.812538000	0.254134000	-0.345419000
7	0.948523000	0.677389000	1.791414000
7	1.485351000	1.448678000	2.375921000
8	-1.719576000	1.009028000	0.630261000
6	0.098935000	1.776535000	-0.794231000
6	-0.690513000	2.904974000	-1.408577000
6	0.092472000	3.143040000	-0.149996000
1	1.004339000	1.447612000	-1.307504000
1	-1.778519000	2.857689000	-1.325686000
1	-0.313763000	3.311134000	-2.349644000
1	1.018545000	3.719472000	-0.202636000
1	-0.476613000	3.262751000	0.774579000

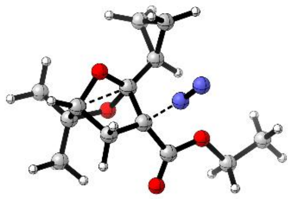

TS3(104)

6	-2.370697000	-0.878125000	-0.385703000
6	-2.186535000	-0.250659000	1.013863000
6	-0.618924000	0.641924000	-0.130047000
6	-0.942620000	-0.856545000	1.716768000
1	-0.867961000	-0.435180000	2.726729000
1	-0.902235000	-1.948040000	1.793326000
6	0.135188000	-0.325390000	0.811941000
1	-3.083220000	-0.171890000	1.634502000
6	-3.558411000	-0.270801000	-1.123008000
1	-3.501342000	-0.531924000	-2.189282000
1	-4.496290000	-0.676185000	-0.715063000
1	-3.571845000	0.822636000	-1.026526000
6	-2.378494000	-2.395772000	-0.450375000
1	-3.215350000	-2.790931000	0.144341000

1	-2.518982000	-2.717961000	-1.492051000
1	-1.439377000	-2.830308000	-0.087177000
8	-1.124900000	-0.378609000	-0.998139000
6	1.212642000	-1.243650000	0.241395000
8	1.048578000	-2.434853000	0.202422000
8	2.236887000	-0.555933000	-0.207602000
6	3.335703000	-1.291815000	-0.827121000
1	2.915803000	-1.874480000	-1.659464000
1	3.729624000	-1.992238000	-0.076508000
6	4.361697000	-0.282585000	-1.279506000
1	5.206328000	-0.811406000	-1.745410000
1	3.934368000	0.408564000	-2.021185000
1	4.744277000	0.299719000	-0.428216000
7	1.178528000	0.792617000	1.846730000
7	1.725627000	1.515148000	2.468021000
8	-1.692403000	1.051917000	0.655670000
6	0.107176000	1.730172000	-0.837781000
6	-0.686890000	2.855243000	-1.446734000
6	0.156016000	3.112135000	-0.229931000
1	0.988500000	1.373228000	-1.373364000
1	-1.770683000	2.833333000	-1.313967000
1	-0.345356000	3.232041000	-2.413190000
1	1.090328000	3.667008000	-0.339440000
1	-0.366075000	3.264683000	0.717304000

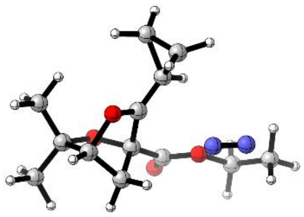

V(104)

6	-2.769084000	-0.434113000	0.198041000
6	-1.911657000	0.235053000	1.298403000
6	-0.333082000	0.795171000	-0.203309000
6	-0.763983000	-0.749421000	1.483719000
1	0.045287000	-0.358436000	2.110694000
1	-1.067620000	-1.745349000	1.818947000
6	-0.452864000	-0.711707000	-0.023941000
1	-2.425803000	0.668510000	2.158821000
6	-3.616987000	0.527834000	-0.623754000
1	-4.100641000	-0.026817000	-1.439933000
1	-4.401041000	0.972897000	0.005599000
1	-3.021499000	1.335961000	-1.065437000

6	-3.616937000	-1.579156000	0.743396000
1	-4.422075000	-1.181228000	1.377814000
1	-4.073793000	-2.115155000	-0.100436000
1	-3.029983000	-2.299090000	1.327181000
8	-1.702169000	-0.972591000	-0.660219000
6	0.645861000	-1.615216000	-0.552835000
8	0.445925000	-2.657516000	-1.116930000
8	1.829698000	-1.091367000	-0.257947000
6	3.011672000	-1.844970000	-0.639927000
1	2.994357000	-1.971097000	-1.732625000
1	2.941383000	-2.842398000	-0.181521000
6	4.224040000	-1.078190000	-0.168474000
1	5.134495000	-1.621860000	-0.461408000
1	4.258721000	-0.076032000	-0.621518000
1	4.223158000	-0.970773000	0.926065000
7	2.092930000	1.303252000	1.835666000
7	3.144445000	1.590331000	1.982806000
8	-1.191696000	1.341748000	0.593311000
6	0.434422000	1.545664000	-1.124103000
6	-0.195200000	2.826516000	-1.753811000
6	0.825453000	3.001855000	-0.726559000
1	1.146302000	0.976894000	-1.719166000
1	-1.240666000	3.006865000	-1.495923000
1	0.068122000	2.952526000	-2.805987000
1	1.847047000	3.257931000	-1.014422000
1	0.518632000	3.303459000	0.277162000

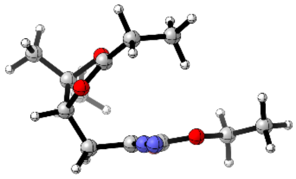

III(106)

6	-2.394256000	-0.716853000	-0.523752000
6	-2.067114000	-0.459069000	0.965902000
6	-1.398536000	1.309217000	-0.242578000
6	-0.844756000	-1.139289000	1.614411000
1	-0.980326000	-1.089886000	2.703619000
1	-0.839629000	-2.202907000	1.340878000
6	0.491437000	-0.551280000	1.236393000
1	-2.957024000	-0.596572000	1.588729000
6	-3.851051000	-0.419890000	-0.840750000
1	-3.998654000	-0.372877000	-1.928059000
1	-4.470361000	-1.233987000	-0.437658000

1	-4.185613000	0.525370000	-0.389542000
6	-1.907945000	-2.010128000	-1.129944000
1	-2.433988000	-2.843794000	-0.641662000
1	-2.161007000	-2.031314000	-2.198848000
1	-0.828561000	-2.152628000	-1.005450000
8	-1.594615000	0.424389000	-1.140260000
6	1.417709000	-1.148170000	0.269133000
8	1.212855000	-2.206838000	-0.292605000
8	2.494487000	-0.379721000	0.065760000
6	3.486109000	-0.858183000	-0.870752000
1	2.990359000	-1.055664000	-1.833158000
1	3.886249000	-1.813076000	-0.496666000
6	4.557367000	0.201521000	-0.986867000
1	5.338794000	-0.139696000	-1.682298000
1	4.139766000	1.144297000	-1.372026000
1	5.024559000	0.397289000	-0.009860000
7	0.912525000	0.515022000	1.882578000
7	1.267719000	1.433963000	2.437823000
8	-1.784916000	0.992505000	0.946048000
6	-0.755134000	2.606622000	-0.536723000
1	-1.523606000	3.222285000	-1.037636000
1	-0.520882000	3.088882000	0.421319000
6	0.478643000	2.451997000	-1.438956000
1	1.246237000	1.828649000	-0.958020000
1	0.208741000	1.996035000	-2.401335000
1	0.905927000	3.445060000	-1.629793000

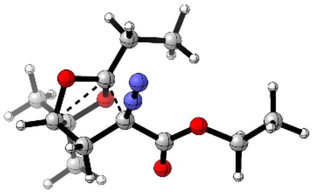

TS2(106)

6	-2.496356000	0.684439000	0.240097000
6	-2.231195000	-0.119811000	-1.049253000
6	-0.863374000	-0.911853000	0.386490000
6	-0.959267000	0.333677000	-1.801044000
1	-0.919725000	-0.148869000	-2.783910000
1	-0.886675000	1.416537000	-1.944026000
6	0.200438000	-0.156397000	-0.906028000
1	-3.106047000	-0.264300000	-1.689743000
6	-3.769638000	0.225828000	0.938801000
1	-3.783377000	0.611480000	1.968139000
1	-4.647490000	0.622186000	0.407220000

1	-3.838493000	-0.869798000	0.968918000
6	-2.420253000	2.194585000	0.133912000
1	-3.180724000	2.549207000	-0.577869000
1	-2.637355000	2.643472000	1.113758000
1	-1.430226000	2.535002000	-0.185790000
8	-1.338272000	0.179403000	1.031184000
6	1.165164000	0.888144000	-0.352626000
8	0.813229000	2.013560000	-0.120750000
8	2.366160000	0.377685000	-0.155134000
6	3.388512000	1.249308000	0.411667000
1	3.036765000	1.574727000	1.401701000
1	3.469276000	2.134679000	-0.235132000
6	4.673084000	0.460472000	0.482060000
1	5.465724000	1.098276000	0.900530000
1	4.561508000	-0.421622000	1.130113000
1	4.986948000	0.128700000	-0.518789000
7	0.901353000	-1.206176000	-1.487291000
7	1.416913000	-2.099451000	-1.896812000
8	-1.808260000	-1.377588000	-0.483879000
6	-0.201289000	-1.948890000	1.247071000
1	-1.037448000	-2.414143000	1.795284000
1	0.213120000	-2.736458000	0.601418000
6	0.837481000	-1.405441000	2.223836000
1	1.739714000	-1.056632000	1.705082000
1	0.425677000	-0.571868000	2.810101000
1	1.138255000	-2.200038000	2.920444000

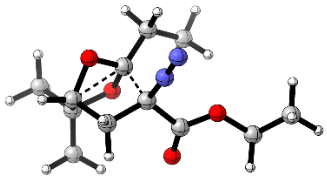

IV(106)

6	-2.541250000	0.649365000	0.186044000
6	-2.199243000	-0.112142000	-1.111555000
6	-0.835324000	-0.884384000	0.333974000
6	-0.899889000	0.406949000	-1.766455000
1	-0.784329000	-0.016179000	-2.770570000
1	-0.828799000	1.497143000	-1.837066000
6	0.196210000	-0.134448000	-0.808410000
1	-3.033442000	-0.256265000	-1.804322000
6	-3.826273000	0.129299000	0.819113000
1	-3.893535000	0.482809000	1.858057000
1	-4.697529000	0.510761000	0.265501000

1	-3.855814000	-0.968505000	0.815883000
6	-2.518333000	2.165092000	0.114337000
1	-3.248170000	2.513159000	-0.631957000
1	-2.804414000	2.584266000	1.089562000
1	-1.523478000	2.547973000	-0.138760000
8	-1.398651000	0.175716000	1.003159000
6	1.177329000	0.891470000	-0.216268000
8	0.828158000	1.998152000	0.086877000
8	2.380292000	0.365657000	-0.097520000
6	3.455664000	1.195260000	0.434674000
1	3.199984000	1.443637000	1.475206000
1	3.479485000	2.125728000	-0.150471000
6	4.733896000	0.400402000	0.322051000
1	5.569204000	1.002537000	0.709768000
1	4.674218000	-0.528228000	0.909338000
1	4.946866000	0.145454000	-0.727079000
7	0.936151000	-1.152087000	-1.449433000
7	1.480704000	-1.999597000	-1.910257000
8	-1.769382000	-1.373117000	-0.563741000
6	-0.230753000	-1.925150000	1.239708000
1	-1.097932000	-2.394449000	1.730767000
1	0.237960000	-2.709403000	0.627215000
6	0.734499000	-1.370714000	2.284908000
1	1.669127000	-1.013249000	1.831831000
1	0.274029000	-0.541381000	2.840048000
1	0.996050000	-2.161276000	3.001953000

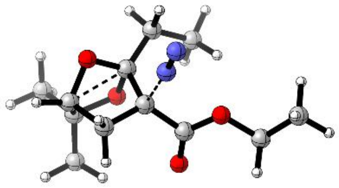

TS3(106)

6	-2.586063000	0.624655000	0.076071000
6	-2.203601000	-0.291974000	-1.107017000
6	-0.797463000	-0.825892000	0.411548000
6	-0.888032000	0.198704000	-1.762031000
1	-0.682727000	-0.398468000	-2.659020000
1	-0.847566000	1.255980000	-2.046118000
6	0.088219000	-0.107031000	-0.653153000
1	-3.006688000	-0.547310000	-1.803691000
6	-3.855482000	0.143509000	0.769348000
1	-3.946931000	0.632636000	1.749649000
1	-4.734788000	0.410561000	0.164162000

1	-3.839787000	-0.944463000	0.915999000
6	-2.621823000	2.117291000	-0.204604000
1	-3.354274000	2.333555000	-0.996422000
1	-2.933432000	2.654084000	0.702885000
1	-1.638567000	2.501956000	-0.499689000
8	-1.430562000	0.328304000	0.942811000
6	1.149981000	0.914440000	-0.273324000
8	0.820070000	2.065345000	-0.148893000
8	2.341608000	0.396563000	-0.098514000
6	3.425315000	1.299785000	0.285200000
1	3.170737000	1.716903000	1.270144000
1	3.447427000	2.120310000	-0.445912000
6	4.701905000	0.496377000	0.303564000
1	5.536074000	1.154198000	0.589038000
1	4.642486000	-0.323765000	1.034294000
1	4.916522000	0.074843000	-0.689546000
7	1.149954000	-1.495651000	-1.317526000
7	1.691599000	-2.350265000	-1.746091000
8	-1.741219000	-1.455262000	-0.401731000
6	-0.220533000	-1.726409000	1.473196000
1	-1.097934000	-2.133343000	1.998710000
1	0.273630000	-2.577939000	0.982481000
6	0.713389000	-1.028585000	2.459371000
1	1.660325000	-0.730245000	1.989933000
1	0.235283000	-0.134731000	2.884869000
1	0.955278000	-1.712163000	3.285140000

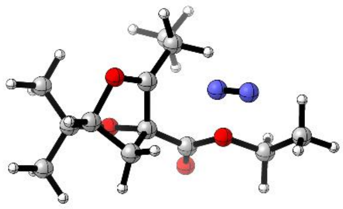

V(106)

6	2.710182000	-0.118708000	-0.374897000
6	1.861308000	1.029012000	-0.977250000
6	0.348435000	0.799274000	0.667446000
6	0.673458000	0.295730000	-1.583648000
1	-0.131038000	0.965345000	-1.907902000
1	0.934227000	-0.405238000	-2.381602000
6	0.383721000	-0.410226000	-0.250035000
1	2.373841000	1.828013000	-1.516585000
6	3.624646000	0.291176000	0.771344000
1	4.069392000	-0.611039000	1.213632000

1	4.436495000	0.927988000	0.392014000
1	3.093025000	0.837023000	1.560598000
6	3.483231000	-0.882974000	-1.445798000
1	4.272652000	-0.243221000	-1.865987000
1	3.957500000	-1.758677000	-0.981232000
1	2.840250000	-1.235597000	-2.261332000
8	1.634014000	-0.969655000	0.161156000
6	-0.746804000	-1.420314000	-0.182572000
8	-0.587716000	-2.609283000	-0.112859000
8	-1.904531000	-0.774879000	-0.236441000
6	-3.122086000	-1.569520000	-0.217464000
1	-3.107292000	-2.189801000	0.690725000
1	-3.100387000	-2.240072000	-1.089234000
6	-4.295071000	-0.619441000	-0.249021000
1	-5.230954000	-1.197573000	-0.238746000
1	-4.289657000	0.044719000	0.628319000
1	-4.279645000	-0.002461000	-1.159510000
7	-2.004701000	2.384297000	-0.644929000
7	-2.985583000	2.758975000	-0.972085000
8	1.195722000	1.644443000	0.228114000
6	-0.347488000	0.973749000	1.942760000
1	-0.127121000	1.971552000	2.344175000
1	-1.424858000	0.901716000	1.716177000
6	0.028204000	-0.146436000	2.942076000
1	-0.191999000	-1.145670000	2.541757000
1	1.093679000	-0.100331000	3.202800000
1	-0.566451000	-0.002510000	3.853631000

4. Synthetic Applications

4.1 Scaling up 27 with a circulation-flow reactor

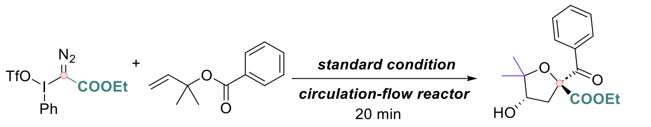

27, 65%, > 20:1 dr, 9.52g

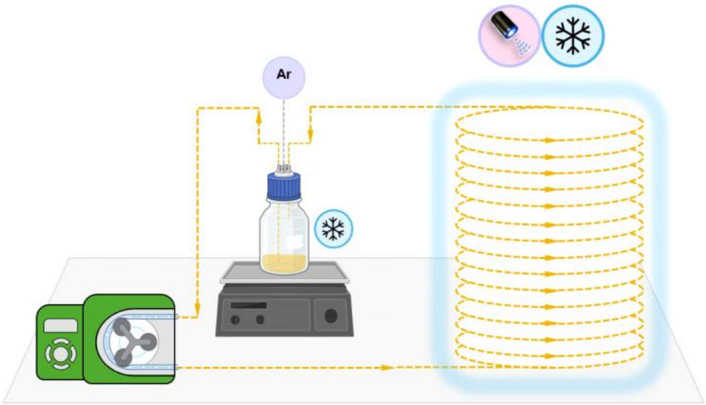

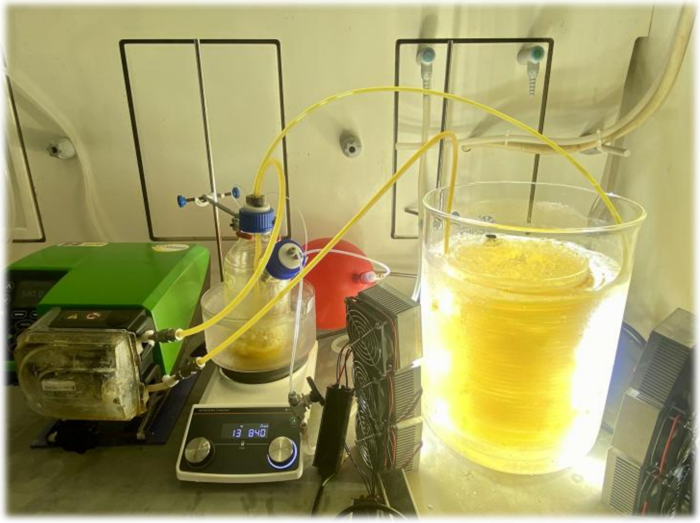

Figure S10. Schematic illustration of the high-speed circulation flow system and platform setup

General Procedure G1 (scaling up of **27** with an in-house circulation-flow reactor):

Ru(bpy)$_3$(PF$_6$)$_2$ (0.5 mmol, 0.01 equiv.), hypervalent iodine reagents **2** (75 mmol, 1.5 equiv.) and $NaHCO_3$ (100 mmol, 2.0 equiv.) were put in a 1 L reservoir. The system was filled with argon. Then, Acetonitrile (500 mL, 0.1 M), allylic benzoate (50 mmol, 1.0 equiv.) and H_2O (100 mmol, 2.0 equiv.) were introduced into reservoir under argon atmosphere. After forming a uniform reaction suspension, the reaction mixture was circulated in the system using a Watson peristaltic pump (flow rate: 600 mL/min) fitted with PharMed® BPT pump tubing (96412-15, I.D. 4.8 mm, O.D. 9.6 mm). The circulation-flow synthesis was conducted in a 1 L PFA tubing reactor (O.D 6.35 mm, I.D. 3.95 mm) under white LEDs irradiation (27W ×12) at 0℃ for 20 min. After irradiation, the crude reaction mixtures were purified by flash column chromatography to give the desired product **27**.

4.2 Further synthetic applications

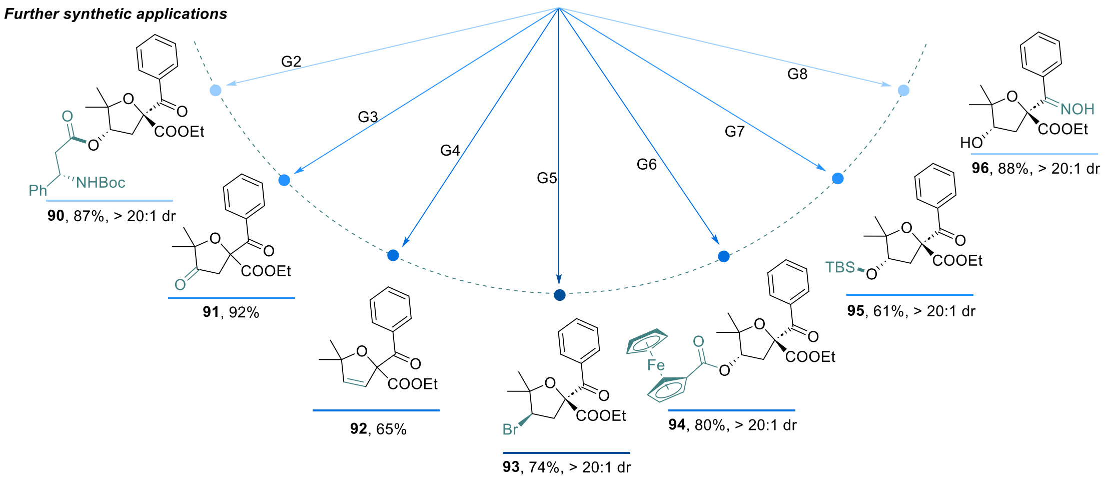

Figure S11. Synthetic applications. (**A**) Scaling up by a circulation-flow reactor. (**B**) Further diversification of product **27**.

General Procedure G2 (for the synthesis of **90**):

The corresponding (S)-*N*-Boc-3-Amino-3-phenylpropanoic acid (1.2 equiv.), **27** (1.0 equiv.), and 4-dimethylaminopyridine (5 mol %) were mixed in a flask with a magnetic stirring bar. Dry CH_2Cl_2 (1 mL) was added. Then a solution of *N*, *N*-dicyclohexylcarbodiimide (1.2 equiv.) in CH_2Cl_2 (0.5 mL) was added slowly at room temperature. The reaction mixture was stirred at room temperature for 6 h. The white precipitate was filtered off and the solution was concentrated under vacuum. **90** were purified by column chromatography on silica gel.

General Procedure G3 (for the synthesis of **91**)[14]:

To a solution of **27** (0.1 mmol, 1.0 equiv.) in dry CH_2Cl_2 (1 mL), was added 1,1,1-triacetoxy-1,1-dihydro-1,2-benziodoxol-3(1H)-one (Dess-Martin) (0.15 mmol, 1.5 equiv.) at room temperature for 2 h. It was then diluted with EtOAc (50 mL/mmol) and washed successively with saturated aq. $NaHCO_3$, water, and brine. The organic layer was dried over Na_2SO_4, filtered, and the solution was concentrated under vacuum. **91** were purified by column chromatography on silica gel.

General Procedure G4 (for the synthesis of **92**)[15]:

To a solution of **27** (0.1 mmol, 1.0 equiv.) in CH_2Cl_2 (1) were added 2,6-lutidine (0.3 mmol, 3.0 equiv.) and Tf_2O (0.15 mmol, 1.5 equiv.) at -78 °C. The mixture was stirred for 30 min at -78 °C, followed by the addition of DBU (0.1mmol, 1.0 equiv.). The resulting mixture was warmed to room temperature and stirred for 2 h. The reaction mixture was quenched by the addition of saturated $NaHCO_3$ solution. After removal of CH_2Cl_2 by evaporation, the residue was extracted with *n*-

hexane/EtOAc (5/1). The combined organic layer was washed with 1 M aqueous HCl and brine, dried over anhydrous $MgSO_4$, filtered, and concentrated, **92** were purified by column chromatography on silica gel.

General Procedure G5 (for the synthesis of **93**)[16]:

To an oven-dried 25 mL round bottom flask was added 1-oxaspiro[4.4]nonan-3-ol (0.1 mmol, 1.0 equiv), DCM (1 mL), and a stir bar. Carbon tetrabromide (0.12 mmol, 1.2 equiv.) was added. Then, the mixture was cooled to 0 °C, and triphenylphosphine (0.12 mmol, 1.2 equiv.) was added slowly. Once the addition was finished, the mixture stirred at room temperature overnight. The solvent was evaporated under reduced pressure, **93** were purified by column chromatography on silica gel.

General Procedure G6 (for the synthesis of **94**):

To a solution of **27** (0.1 mmol, 1.0 equiv.) in DCM (1 mL) added pyridine (2.0 mmol, 2.0 equiv.) and acyl chloride (1.2 mmol, 1.2 equiv) at 0 °C. The reaction mixture stirred at room temperature for 24 h, then quenched with saturated $NaHCO_3$ solution (20.0 mL) and extracted with DCM (three times). The organic layer was collected, washed with brine, dried with anhydrous $MgSO_4$ and filtered, and concentrated, **94** were purified by column chromatography on silica gel.

General Procedure G7 (for the synthesis of **95**)[17]:

To a solution of **27** (0.1 mmol, 1.0 equiv.) in DCM (1 mL) was cooled to 0 °C, and imidazole (1 mmol, 10equiv.) and TBSCl (0.1 mmol, 3.6 equiv.) were added under argon atmosphere. The resulting solution was stirred at 50 °C for 24 h. The reaction mixture was extracted with ethyl acetate (three times), and dried over anhydrous Na_2SO_4, filtered, concentrated to afford the residue and the solution was concentrated under vacuum, **95** were purified by column chromatography on silica gel.

General Procedure G8 (for the synthesis of **96**)[18]:

In an oven-dried 8 ml Schlenk tube equipped with a PTFE-coated oval stirring bar, **27** (0.1 mmol, 1.0 equiv.), hydroxylamine hydrochloride (0.2 mmol, 2.0 equiv.), sodium acetate (0.20 mmol, 2.0 equiv.), were charged under air, then the vessel was evacuated and re-filled with argon for three times. EtOH (1 mL) were added under argon atmosphere. The mixture was heated under reflux for 24 h. The solvent was evaporated under reduced pressure, **96** were purified by column chromatography on silica gel.

5. Crystal Data

Crystallographic data for compound **4** (CCDC-2427431) has been deposited with the Cambridge Crystallographic Data Centre, Copies of the data can be obtained, free of charge, on application to CCDC (Email: deposit@ccdc.cam.ac.uk).

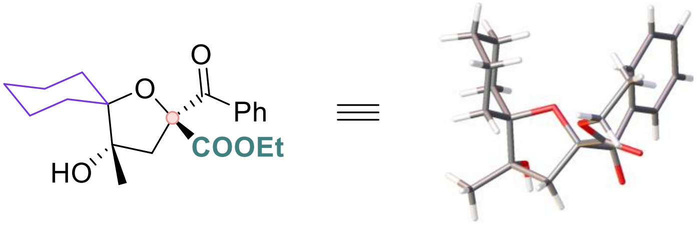

Bond precision: C-C = 0.0013 A   Wavelength=0.71073

Cell:	a=8.2780(3)	b=9.4743(3)	c=12.2832(5)
	alpha=87.1300(15)	beta=79.5100(16)	gamma=68.6600(13)
Temperature:	100 K		

	Calculated	Reported
Volume	882.17(6)	882.17(6)
Space group	P -1	P -1
Hall group	-P 1	-P 1
Moiety formula	C20 H26 O5	C20 H26 O5
Sum formula	C20 H26 O5	C20 H26 O5
Mr	346.41	346.41
Dx,g cm-3	1.304	1.304
Z	2	2
Mu (mm-1)	0.093	0.093
F000	372.0	372.0
F000'	372.20	
h,k,lmax	11,13,17	11,13,17
Nref	4949	4941
Tmin,Tmax	0.985,0.991	0.731,0.743
Tmin'	0.982	

Correction method= # Reported T Limits: Tmin=0.731 Tmax=0.743
AbsCorr = MULTI-SCAN

Data completeness= 0.998   Theta(max)= 29.594

R(reflections)= 0.0355(4505)   wR2(reflections)= 0.0968(4941)

S = 1.023   Npar= 231

Figure S12. Crystal structure of compound **4**

SUPPORTING INFORMATION

Crystallographic data for compound **43** (CCDC-2427418) has been deposited with the Cambridge Crystallographic Data Centre, Copies of the data can be obtained, free of charge, on application to CCDC (Email: deposit@ccdc.cam.ac.uk).

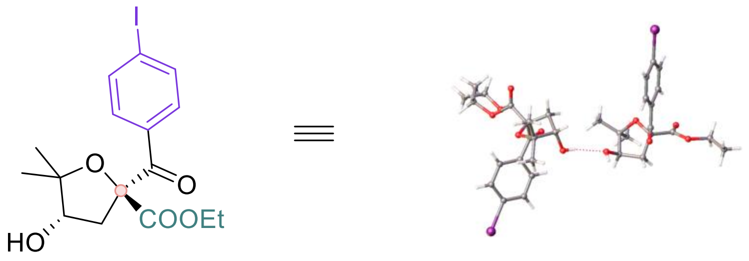

Bond precision: C-C = 0.0049 A   Wavelength=0.71073

Cell: a=12.2462(4)   b=15.0674(6)   c=36.5386(15)
alpha=90   beta=90   gamma=90

Temperature: 100 K

	Calculated	Reported
Volume	6742.0(4)	6742.0(4)
Space group	P b c a	P b c a
Hall group	-P 2ac 2ab	-P 2ac 2ab
Moiety formula	C16 H19 I O5	C16 H19 I O5
Sum formula	C16 H19 I O5	C16 H19 I O5
Mr	418.21	418.21
Dx,g cm-3	1.648	1.648
Z	16	16
Mu (mm-1)	1.920	1.920
F000	3328.0	3328.0
F000'	3321.98	
h,k,lmax	16,20,48	16,20,48
Nref	8362	8360
Tmin,Tmax	0.830,0.907	0.689,0.746
Tmin'	0.830	

Correction method= # Reported T Limits: Tmin=0.689 Tmax=0.746
AbsCorr = MULTI-SCAN

Data completeness= 1.000   Theta(max)= 28.281

R(reflections)= 0.0362(5965)   wR2(reflections)= 0.0796(8360)

S = 1.027   Npar= 438

Figure S13. Crystal structure of compound **43**

SUPPORTING INFORMATION

Crystallographic data for compound **80** (CCDC-2427423) has been deposited with the Cambridge Crystallographic Data Centre, Copies of the data can be obtained, free of charge, on application to CCDC (Email: deposit@ccdc.cam.ac.uk).

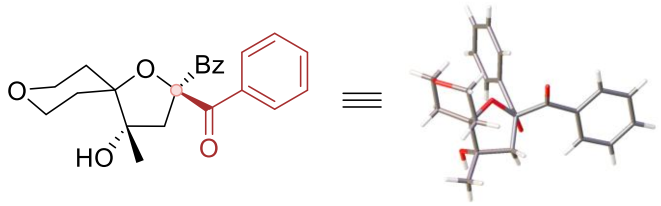

Bond precision:	C-C = 0.0035 A		Wavelength=0.71073
Cell:	a=6.8473(4)	b=14.5132(9)	c=10.1854(6)
	alpha=90	beta=109.178(2)	gamma=90
Temperature:	100 K		

	Calculated	Reported
Volume	956.01(10)	956.01(10)
Space group	P 21	P 1 21 1
Hall group	P 2yb	P 2yb
Moiety formula	C23 H24 O5	C23 H24 O5
Sum formula	C23 H24 O5	C23 H24 O5
Mr	380.42	380.42
Dx,g cm-3	1.322	1.322
Z	2	2
Mu (mm-1)	0.092	0.092
F000	404.0	404.0
F000'	404.21	
h,k,lmax	9,20,14	9,20,14
Nref	5385[2793]	5360
Tmin,Tmax	0.986,0.992	0.685,0.746
Tmin'	0.983	

Correction method= # Reported T Limits: Tmin=0.685 Tmax=0.746
AbsCorr = MULTI-SCAN

Data completeness= 1.92/1.00 Theta(max)= 29.596

R(reflections)= 0.0442(4603) wR2(reflections)= 0.0968(5360)

S = 1.087 Npar= 257

Figure S14. Crystal structure of compound **80**

SUPPORTING INFORMATION

Crystallographic data for compound **82** (CCDC-2504729) has been deposited with the Cambridge Crystallographic Data Centre, Copies of the data can be obtained, free of charge, on application to CCDC (Email: deposit@ccdc.cam.ac.uk).

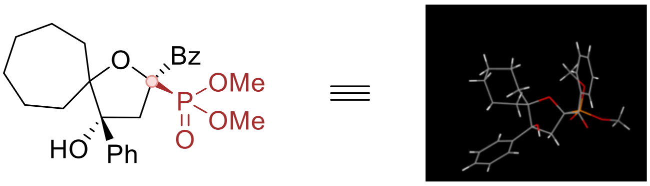

Bond precision:	C-C = 0.0038 A		Wavelength=0.71073
Cell:	a=12.9754(9)	b=8.7021(7)	c=20.3728(14)
	alpha=90	beta=90	gamma=90
Temperature:	100 K		

	Calculated	Reported
Volume	2300.4(3)	2300.4(3)
Space group	P c a 21	P c a 21
Hall group	P 2c -2ac	P 2c -2ac
Moiety formula	C25 H31 O6 P	C25 H31 O6 P
Sum formula	C25 H31 O6 P	C25 H31 O6 P
Mr	458.47	458.47
Dx,g cm-3	1.324	1.324
Z	4	4
Mu (mm-1)	0.159	0.159
F000	976.0	976.0
F000'	976.89	
h,k,lmax	18,12,28	18,12,28
Nref	6468[3323]	6438
Tmin,Tmax	0.972,0.978	0.697,0.739
Tmin'	0.939	

Correction method= # Reported T Limits: Tmin=0.697 Tmax=0.739
AbsCorr = MULTI-SCAN

Data completeness= 1.94/1.00　　Theta(max)= 29.598

R(reflections)= 0.0391(5977)　　wR2(reflections)= 0.0871(6438)

S = 1.080　　Npar= 294

Figure S15. Crystal structure of compound **82**

SUPPORTING INFORMATION

Crystallographic data for compound **89** (CCDC-2504733) has been deposited with the Cambridge Crystallographic Data Centre, Copies of the data can be obtained, free of charge, on application to CCDC (Email: deposit@ccdc.cam.ac.uk).

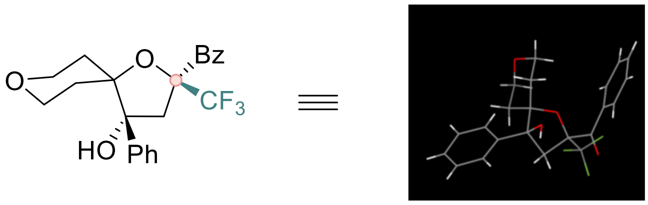

Bond precision:	C-C = 0.0026 A		Wavelength=0.71073
Cell:	a=8.6292(4)	b=21.0757(13)	c=10.7882(7)
	alpha=90	beta=101.230(2)	gamma=90
Temperature:	100 K		

	Calculated	Reported
Volume	1924.45(19)	1924.45(19)
Space group	P 21/n	P 1 21/n 1
Hall group	-P 2yn	-P 2yn
Moiety formula	C22 H21 F3 O4	C22 H21 F3 O4
Sum formula	C22 H21 F3 O4	C22 H21 F3 O4
Mr	406.39	406.39
Dx,g cm-3	1.403	1.403
Z	4	4
Mu (mm-1)	0.114	0.114
F000	848.0	848.0
F000'	848.57	
h,k,lmax	11,29,14	11,29,14
Nref	5381	5371
Tmin,Tmax	0.985,0.994	0.697,0.746
Tmin'	0.980	

Correction method= # Reported T Limits: Tmin=0.697 Tmax=0.746
AbsCorr = MULTI-SCAN

Data completeness= 0.998 Theta(max)= 29.590

R(reflections)= 0.0540(3731) wR2(reflections)= 0.1367(5371)

S = 1.051 Npar= 265

Figure S16. Crystal structure of compound **89**

6. Characterization Data for Products

4-(prop-1-en-2-yl)tetrahydro-2H-pyran-4-yl benzoate (1a)

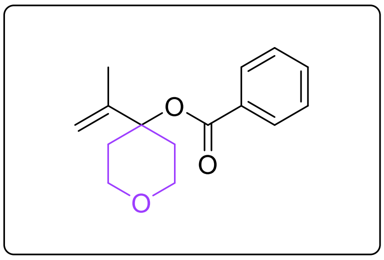

Following the **general procedure A** on 10 mmol scale, yellow oil, total yield: 44% (1.08 g). R_f = 0.7 (silica gel, hexane: EtOAc = 10:1, v/v), column chromatography (neutral alumina, hexane: EtOAc = 80: 1, v/v).

^{1}H NMR (500 MHz, Chloroform-*d*) δ 8.1 (m, 2H), 7.6 – 7.5 (m, 1H), 7.5 (m, 2H), 5.1 (s, 1H), 5.0 (s, 1H), 3.9 – 3.9 (m, 2H), 3.8 (m, 2H), 2.4 (m, 2H), 2.0 (m, 2H), 1.8 (m, 3H).

^{13}C NMR (126 MHz, Chloroform-*d*) δ 164.9, 146.6, 133.0, 130.9, 129.5, 128.5, 111.5, 81.4, 63.7, 34.5, 18.3.

HRMS (EI) m/z: $[M]^+$ Calcd. for $C_{15}H_{18}O_3^+$ 246.1256; Found: 246.1256.

1-(prop-1-en-2-yl)cyclohexyl benzoate (1b)

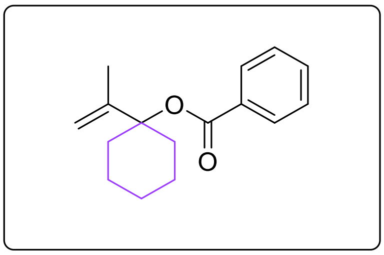

Following the **general procedure A** on 10 mmol scale, yellow oil, total yield: 41% (1.01 g). R_f = 0.8 (silica gel, hexane: EtOAc = 10:1, v/v), column chromatography (neutral alumina, hexane: EtOAc = 100: 1, v/v).

^{1}H NMR (400 MHz, Chloroform-*d*) δ 8.1 – 8.0 (m, 2H), 7.6 – 7.5 (m, 1H), 7.5 (m, 2H), 5.0 (s, 1H), 5.0 (s, 1H), 2.5 (mm, 2H), 1.8 (s, 3H), 1.8 – 1.6 (m, 7H), 1.4 – 1.3 (m, 1H).

^{13}C NMR (101 MHz, Chloroform-*d*) δ 164.9, 148.3, 132.7, 131.4, 129.5, 128.3, 110.5, 84.3, 34.3, 25.6, 22.0, 18.7.

HRMS (EI) m/z: $[M]^+$ Calcd. for $C_{16}H_{20}O_2^+$ 244.1463; Found: 244.1460.

1-(prop-1-en-2-yl)cycloheptyl benzoate (1c)

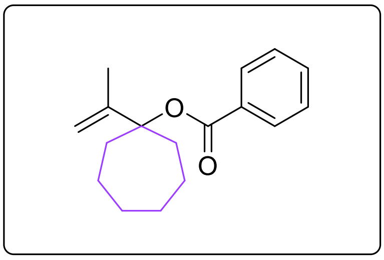

Following the **general procedure A** on 10 mmol scale, yellow oil, total yield: 49% (1.26 g). R_f = 0.8 (silica gel, hexane: EtOAc = 10:1, v/v), column chromatography (neutral alumina, hexane: EtOAc = 100: 1, v/v).

^{1}H NMR (500 MHz, Chloroform-*d*) δ 8.1 – 8.0 (m, 2H), 7.6 – 7.5 (m, 1H), 7.5 (m, 2H), 5.0 (s, 1H), 4.9 (s, 1H), 2.4 – 2.3 (m, 2H), 2.1 (m, 2H), 1.8 (m, 3H), 1.8 – 1.7 (m, 2H), 1.7 – 1.6 (m, 6H).

^{13}C NMR (126 MHz, Chloroform-*d*) δ 165.1, 149.2, 132.6, 131.4, 129.5, 128.3, 109.9, 88.6, 38.2, 29.3, 23.2, 19.2.

HRMS (EI) m/z: $[M]^+$ Calcd. for $C_{17}H_{22}O_2^+$ 258.1620; Found: 258.1626.

1-(prop-1-en-2-yl)cyclopentadecyl benzoate (1d)

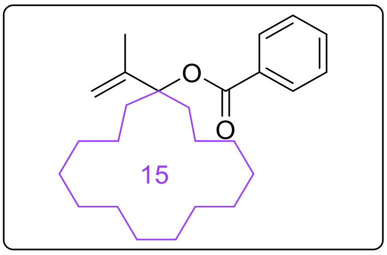

Following the **general procedure A** on 10 mmol scale, yellow oil, total yield: 39% (1.05 g). R_f = 0.8 (silica gel, hexane: EtOAc = 10:1, v/v), column chromatography (neutral alumina, hexane: EtOAc = 100: 1, v/v).

^{1}H NMR (500 MHz, Chloroform-*d*) δ 8.0 (m, 2H), 7.5 (m, 1H), 7.4 (m, 2H), 5.0 (s, 1H), 5.0 (s, 1H), 2.3 – 2.2 (m, 2H), 1.8 (m, 2H), 1.7 (s, 3H), 1.4 – 1.2 (m, 24H).

^{13}C NMR (126 MHz, Chloroform-*d*) δ 164.9, 146.5, 132.6, 129.5, 128.3, 111.9, 87.9, 33.1, 27.6, 27.0, 26.9, 26.7, 26.6, 21.5, 18.9.

HRMS (ESI) m/z: $[M+Na]^+$ Calcd. for $C_{25}H_{38}O_2^+$ 393.2770; Found: 393.2769.

4,4-dimethyl-1-(prop-1-en-2-yl)cyclohexyl benzoate (1e)

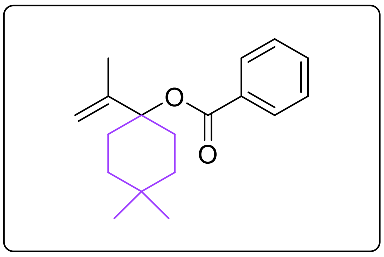

Following the **general procedure A** on 10 mmol scale, yellow oil, total yield: 41% (1.12 g). R_f = 0.8 (silica gel, hexane: EtOAc = 10:1, v/v), column chromatography (neutral alumina, hexane: EtOAc = 100: 1, v/v).

^{1}H NMR (600 MHz, Chloroform-*d*) δ 8.1 – 8.0 (m, 2H), 7.6 – 7.5 (m, 1H), 7.5 – 7.4 (m, 2H), 5.0 (s, 1H), 5.0 (s, 1H), 2.4 – 2.3 (m, 2H), 1.8 (m, 1H), 1.8 (m, 3H), 1.8 (m, 1H), 1.6 (mm, 2H), 1.4 – 1.3 (m, 2H), 1.0 (s, 3H), 0.9 (s, 3H).

^{13}C NMR (151 MHz, Chloroform-*d*) δ 164.9, 148.0, 132.7, 131.4, 129.5, 128.4, 110.6, 84.1, 34.9, 32.5, 30.3, 29.5, 24.3, 18.8.

HRMS (EI) m/z: $[M]^+$ Calcd. for $C_{18}H_{24}O_2^+$ 272.1776; Found: 272.1773.

4-(1-phenylvinyl)tetrahydro-2H-pyran-4-yl benzoate (1f)

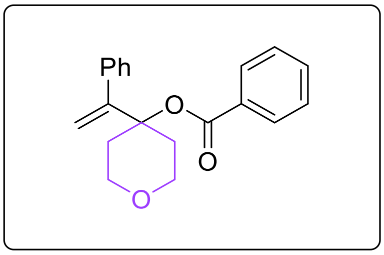

Following the **general procedure A** on 10 mmol scale, yellow oil, total yield: 44% (1.36 g). R_f = 0.8 (silica gel, hexane: EtOAc = 10:1, v/v), column chromatography (neutral alumina, hexane: EtOAc = 100: 1, v/v).

^{1}H NMR (400 MHz, Chloroform-*d*) δ 8.1 – 8.0 (m, 2H), 7.6 – 7.5 (m, 1H), 7.5 (m, 2H), 7.3 – 7.2 (m, 5H), 5.5 (s, 1H), 5.3 (s, 1H), 3.9 (m, 2H), 3.8 (m, 2H), 2.5 – 2.4 (m, 2H), 2.1 (m, 2H).

^{13}C NMR (126 MHz, Chloroform-*d*) δ 165.1, 151.5, 140.2, 133.0, 131.1, 129.5, 128.8, 128.5, 127.9, 127.3, 116.2, 80.7, 63.7, 35.5.

HRMS (EI) m/z: $[M]^+$ Calcd. for $C_{22}H_{24}O_2^+$ 308.1412; Found: 308.1418.

1-(1-phenylvinyl)cycloheptyl benzoate (1g)

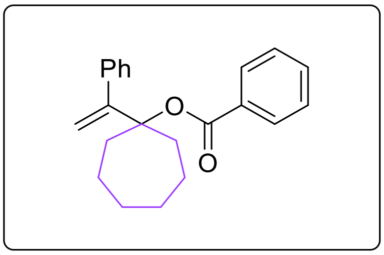

Following the **general procedure A** on 10 mmol scale, yellow oil, total yield: 37% (1.18 g). R_f = 0.8 (silica gel, hexane: EtOAc = 10:1, v/v), column chromatography (neutral alumina, hexane: EtOAc = 100: 1, v/v).

^{1}H NMR (400 MHz, Chloroform-*d*) δ 8.1 – 7.9 (m, 2H), 7.6 – 7.5 (m, 1H), 7.4 (m, 2H), 7.3 – 7.3 (m, 2H), 7.3 – 7.2 (m, 3H), 5.4 (s, 1H), 5.1 (s, 1H), 2.5 – 2.4 (m, 2H), 2.2 (m, 2H), 1.7 – 1.5 (m, 8H).

^{13}C NMR (126 MHz, Chloroform-*d*) δ 165.3, 153.8, 141.1, 132.6, 131.6, 129.5, 129.5, 128.9, 128.3, 128.1, 127.6, 126.9, 114.3, 87.8, 39.4, 29.1, 23.0.

HRMS (EI) m/z: $[M]^+$ Calcd. for $C_{22}H_{24}O_2^+$ 320.1776; Found: 320.1778.

1-vinylcyclobutyl benzoate (1h)

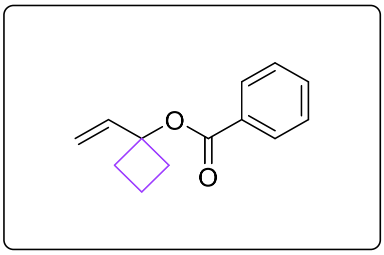

Following the **general procedure B** on 10 mmol scale, yellow oil, total yield: 45% (0.91 g). R_f = 0.8 (silica gel, hexane: EtOAc = 10:1, v/v), column chromatography (neutral alumina, hexane: EtOAc = 100: 1, v/v).

^{1}H NMR (400 MHz, Chloroform-*d*) δ 8.0 (m, 2H), 7.6 – 7.5 (m, 1H), 7.5 – 7.4 (m, 2H), 6.3 (m, 1H), 5.3 (dd, *J* = 17.4, 0.9 Hz, 1H), 5.2 (dd, *J* = 10.7, 0.9 Hz, 1H), 2.6 – 2.5 (m, 4H), 2.0 – 1.8 (m, 1H), 1.8 – 1.7 (m, 1H).

^{13}C NMR (126 MHz, Chloroform-*d*) δ 165.1, 138.4, 132.8, 130.9, 129.6, 128.3, 113.6, 81.4, 34.2, 14.0.

HRMS (EI) m/z: $[M]^+$ Calcd. for $C_{13}H_{14}O_2^+$ 202.0994; Found: 202.0983.

1-vinylcyclohexyl benzoate (1i)

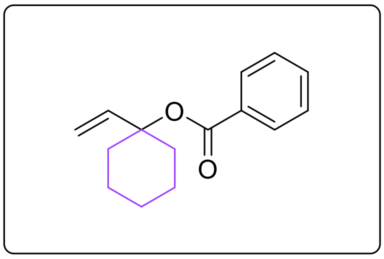

Following the **general procedure B** on 10 mmol scale, yellow oil, yield: 39% (0.90 g). R_f = 0.8 (silica gel, hexane: EtOAc = 10:1, v/v), column chromatography (neutral alumina, hexane: EtOAc = 100: 1, v/v).

^{1}H NMR (500 MHz, Chloroform-*d*) δ 8.1 – 8.0 (m, 2H), 7.6 – 7.5 (m, 1H), 7.5 – 7.4 (m, 2H), 6.2 (dd, *J* = 17.6, 11.0 Hz, 1H), 5.2 (dd, *J* = 17.7, 0.8 Hz, 1H), 5.2 (dd, *J* = 11.0, 0.8 Hz, 1H), 2.4 – 2.4 (m, 2H), 1.6 (m, 7H), 1.3 (m, 1H).

^{13}C NMR (126 MHz, Chloroform-*d*) δ 165.1, 142.0, 132.6, 131.6, 129.5, 128.3, 113.7, 82.4, 35.0, 25.4, 22.0.

HRMS (EI) m/z: $[M]^+$ Calcd. for $C_{15}H_{18}O_2^+$ 230.1307; Found: 230.1310.

1-vinylcyclododecyl benzoate (1j)

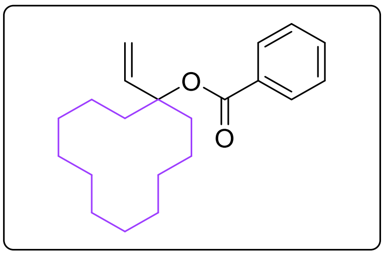

Following the **general procedure B** on 10 mmol scale, yellow oil, yield: 39% (1.24 g). R_f = 0.8 (silica gel, hexane: EtOAc = 10:1, v/v), column chromatography (neutral alumina, hexane: EtOAc = 100: 1, v/v).

^{1}H NMR (400 MHz, Chloroform-*d*) δ 8.0 – 8.0 (m, 2H), 7.6 – 7.5 (m, 1H), 7.4 (dd, *J* = 8.3, 7.0 Hz, 2H), 6.1 (dd, *J* = 17.9, 10.8 Hz, 1H), 5.2 – 5.2 (m, 2H), 2.3 (m, 2H), 1.8 (m, 2H), 1.4 (m, 18H).

^{13}C NMR (101 MHz, Chloroform-*d*) δ 165.1, 141.4, 132.6, 131.4, 129.5, 128.3, 114.0, 86.1, 31.1, 26.1, 26.1, 22.3, 22.0, 19.1.

HRMS (EI) m/z: $[M]^+$ Calcd. for $C_{21}H_{30}O_2^+$ 314.2246; Found: 314.2240.

1-vinylcyclopentadecyl benzoate (1k)

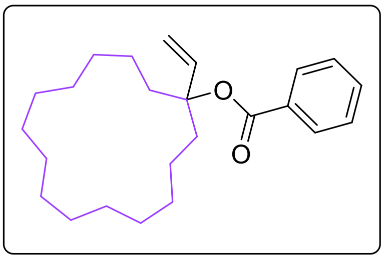

Following the **general procedure B** on 10 mmol scale, yellow oil, yield: 41% (1.45 g). R_f = 0.8 (silica gel, hexane: EtOAc = 10:1, v/v), column chromatography (neutral alumina, hexane: EtOAc = 100: 1, v/v).

^{1}H NMR (400 MHz, Chloroform-*d*) δ 8.0 (m, 2H), 7.5 (m, 1H), 7.4 (m, 2H), 6.1 (dd, *J* = 17.7, 10.9 Hz, 1H), 5.2 (d, *J* = 4.7 Hz, 1H), 5.2 (s, 1H), 2.2 (m, 2H), 1.8 (m, 2H), 1.4 – 1.3 (m, 24H).

^{13}C NMR (101 MHz, Chloroform-*d*) δ 165.1, 141.5, 132.6, 131.6, 129.5, 128.3, 114.0, 86.0, 34.6, 27.6, 27.0, 26.7, 26.7, 26.3, 21.4.

HRMS (EI) m/z: $[M]^+$ Calcd. for $C_{24}H_{36}O_2^+$ 356.2715; Found: 356.2719.

2,6-dimethyl-1-vinylcyclohexyl benzoate (1l)

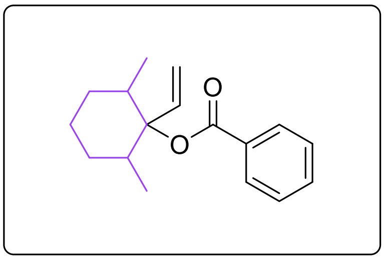

Following the **general procedure B** on 10 mmol scale, yellow oil, yield: 54% (1.39 g). R_f = 0.8 (silica gel, hexane: EtOAc = 10:1, v/v), column chromatography (neutral alumina, hexane: EtOAc = 100: 1, v/v).

^{1}H NMR (400 MHz, Chloroform-*d*) δ 8.1 – 8.0 (m, 2H), 7.6 – 7.5 (m, 1H), 7.5 – 7.4 (m, 2H), 6.6 (dd, *J* = 18.0, 11.6 Hz, 1H), 5.2 (dd, *J* = 11.6, 0.8 Hz, 1H), 5.0 (dd, *J* = 18.0, 0.8 Hz, 1H), 1.9 – 1.8 (m, 3H), 1.6 – 1.6 (m, 4H), 1.5 – 1.4 (m, 1H), 1.0 (s, 3H), 1.0 (s, 3H).

^{13}C NMR (101 MHz, Chloroform-*d*) δ 165.5, 141.6, 132.6, 131.8, 129.5, 128.4, 110.2, 88.8, 42.6, 30.8, 25.7, 17.4.

HRMS (EI) m/z: $[M]^+$ Calcd. for $C_{17}H_{22}O_2^+$ 258.1620; Found: 258.1612.

4,4-difluoro-1-vinylcyclohexyl benzoate (1m)

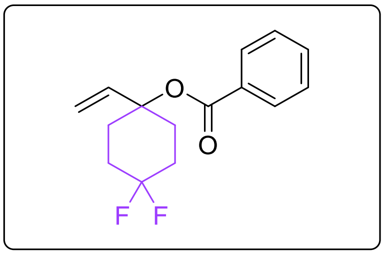

Following the **general procedure B** on 10 mmol scale, yellow oil, yield: 42% (1.12 g). R_f = 0.8 (silica gel, hexane: EtOAc = 10:1, v/v), column chromatography (neutral alumina, hexane: EtOAc = 100: 1, v/v).

^{1}H NMR (400 MHz, Chloroform-*d*) δ 8.0 – 7.9 (m, 2H), 7.6 – 7.4 (m, 1H), 7.4 (m, 2H), 6.1 (dd, *J* = 17.6, 11.0 Hz, 1H), 5.2 (d, *J* = 17.7 Hz, 1H), 5.2 (d, *J* = 11.0 Hz, 1H), 2.6 – 2.5 (m, 2H), 2.1 – 1.9 (m, 4H), 1.9 – 1.8 (m, 2H).

^{13}C NMR (126 MHz, Chloroform-*d*) δ 165.0, 139.9, 139.9, 133.1, 130.8, 129.5, 128.5, 124.6, 122.7, 120.8, 115.0, 80.3, 31.5, 31.4, 30.0, 29.8, 29.6.

^{19}F NMR (377 MHz, Chloroform-d) δ -93.4 (d, *J* = 237.9 Hz), -102.7 (d, *J* = 237.7 Hz).

HRMS (EI) m/z: $[M]^+$ Calcd. for $C_{15}H_{16}F_2O_2^+$ 266.1118; Found: 266.1113.

7-vinyl-6,7,8,9-tetrahydro-5H-benzo[7]annulen-7-yl benzoate (1n)

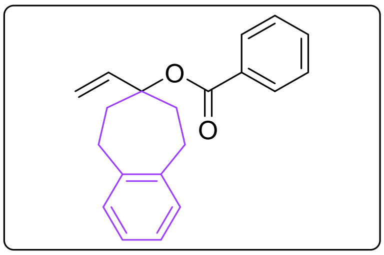

Following the **general procedure B** on 10 mmol scale, yellow oil, yield: 38% (0.77 g). R_f = 0.8 (silica gel, hexane: EtOAc = 10:1, v/v), column chromatography (neutral alumina, hexane: EtOAc = 100: 1, v/v).

^{1}H NMR (500 MHz, Chloroform-*d*) δ 8.2 – 8.1 (m, 2H), 7.6 – 7.5 (m, 1H), 7.5 (m, 2H), 7.2 (s, 4H), 6.3 (dd, *J* = 17.7, 11.1 Hz, 1H), 5.2 – 5.1 (m, 2H), 3.3 (m, 2H), 2.8 – 2.7 (m, 2H), 2.7 (m, 2H), 1.8 (s, 2H).

^{13}C NMR (126 MHz, Chloroform-*d*) δ 165.1, 142.7, 142.3, 132.9, 131.5, 129.5, 129.0, 128.4, 126.4, 113.6, 85.0, 36.7, 29.6.

HRMS (EI) m/z: $[M]^+$ Calcd. for $C_{15}H_{18}O_2^+$ 292.1463; Found: 292.1460.

8-vinyl-1,4-dioxaspiro[4.5]decan-8-yl benzoate (1o)

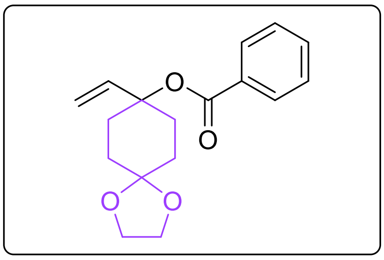

Following the **general procedure B** on 10 mmol scale, yellow oil, yield: 47% (1.35 g). R_f = 0.8 (silica gel, hexane: EtOAc = 10:1, v/v), column chromatography (neutral alumina, hexane: EtOAc = 100: 1, v/v).

^{1}H NMR (400 MHz, Chloroform-*d*) δ 8.0 (m, 2H), 7.6 – 7.5 (m, 1H), 7.5 – 7.4 (m, 2H), 6.2 (dd, *J* = 17.6, 11.0 Hz, 1H), 5.3 (dd, *J* = 17.6, 0.7 Hz, 1H), 5.2 (dd, *J* = 11.0, 0.8 Hz, 1H), 4.0 (s, 4H), 2.6 – 2.5 (m, 2H), 2.0 – 1.9 (m, 4H), 1.7 (m, 2H).

^{13}C NMR (126 MHz, Chloroform-*d*) δ 165.2, 140.9, 132.8, 131.2, 129.5, 128.3, 114.2, 108.1, 81.3, 64.4, 64.3, 32.5, 30.6.

HRMS (EI) m/z: $[M]^+$ Calcd. for $C_{17}H_{20}O_4^+$ 288.1362; Found: 288.1361.

3,3-dimethyl-9-vinyl-1,5-dioxaspiro[5.5]undecan-9-yl benzoate (1p)

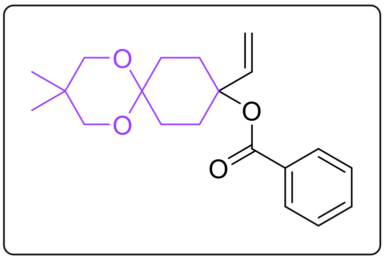

Following the **general procedure B** on 10 mmol scale, yellow oil, yield: 41% (1.37 g). R_f = 0.8 (silica gel, hexane: EtOAc = 10:1, v/v), column chromatography (neutral alumina, hexane: EtOAc = 100: 1, v/v).

^{1}H NMR (400 MHz, Chloroform-*d*) δ 8.0 – 8.0 (m, 2H), 7.5 (m, 1H), 7.5 – 7.4 (m, 2H), 6.2 (dd, *J* = 17.6, 11.0 Hz, 1H), 5.3 – 5.1 (m, 2H), 3.5 – 3.4 (m, 4H), 2.5 – 2.3 (m, 2H), 2.2 – 2.1 (m, 2H), 1.9 – 1.7 (m, 4H), 1.0 (s, 6H).

^{13}C NMR (126 MHz, Chloroform-*d*) δ 165.2, 140.9, 132.8, 131.3, 129.5, 128.3, 114.1, 96.9, 81.8, 70.1, 31.2, 30.3, 28.1, 22.7.

HRMS (EI) m/z: $[M]^+$ Calcd. for $C_{20}H_{26}O_4^+$ 330.1831; Found: 330.1830.

3-vinyloxetan-3-yl benzoate (1q)

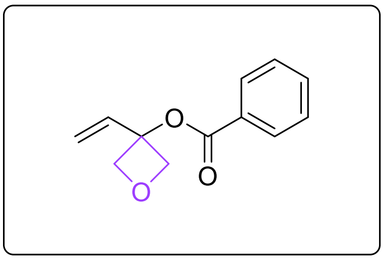

Following the **general procedure B** on 10 mmol scale, yellow oil, yield: 38% (0.78 g). R_f = 0.7 (silica gel, hexane: EtOAc = 10:1, v/v), column chromatography (neutral alumina, hexane: EtOAc = 80: 1, v/v).

^{1}H NMR (500 MHz, Chloroform-*d*) δ 8.1 (m, 2H), 7.6 – 7.6 (m, 1H), 7.5 (m, 2H), 6.4 (dd, *J* = 17.4, 10.9 Hz, 1H), 5.4 (d, *J* = 17.4 Hz, 1H), 5.3 (d, *J* = 10.9 Hz, 1H), 5.0 (m, 2H), 4.8 (m, 2H).

^{13}C NMR (126 MHz, Chloroform-*d*) δ 165.0, 135.7, 133.5, 129.8, 129.6, 128.5, 115.7, 80.9.

HRMS (EI) m/z: $[M]^+$ Calcd. for $C_{20}H_{26}O_4^+$ 204.0786; Found: 204.07813.

3-vinyltetrahydro-2H-pyran-3-yl benzoate (1r)

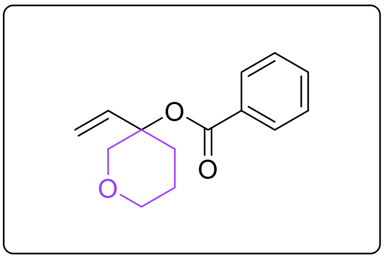

Following the **general procedure B** on 10 mmol scale, yellow oil, yield: 61% (1.42 g). R_f = 0.7 (silica gel, hexane: EtOAc = 10:1, v/v), column chromatography (neutral alumina, hexane: EtOAc = 80: 1, v/v).

^{1}H NMR (500 MHz, Chloroform-*d*) δ 8.1 – 8.0 (m, 2H), 7.6 – 7.5 (m, 1H), 7.4 (m, 2H), 6.1 (dd, *J* = 17.7, 11.1 Hz, 1H), 5.3 – 5.2 (m, 2H), 4.2 (m, 1H), 3.9 – 3.8 (m, 1H), 3.6 (m, 1H), 3.6 – 3.5 (m, 1H), 2.6 – 2.5 (m, 1H), 1.9 (m, 2H), 1.6 (m, 1H).

^{13}C NMR (126 MHz, Chloroform-*d*) δ 165.2, 138.2, 132.8, 131.1, 129.7, 128.3, 115.9, 79.1, 73.0, 67.9, 32.0, 22.1.

HRMS (EI) m/z: $[M]^+$ Calcd. for $C_{14}H_{16}O_3^+$ 232.1099; Found: 232.1089.

2,2-dimethyl-4-vinyltetrahydro-2H-pyran-4-yl benzoate (1s)

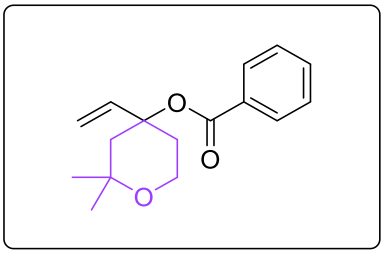

Following the **general procedure B** on 10 mmol scale, yellow oil, yield: 53% (1.38 g). R_f = 0.7 (silica gel, hexane: EtOAc = 10:1, v/v), column chromatography (neutral alumina, hexane: EtOAc = 80: 1, v/v).

^{1}H NMR (500 MHz, Chloroform-*d*) δ 8.1 – 8.0 (m, 2H), 7.6 – 7.5 (m, 1H), 7.5 (m, 2H), 6.2 (dd, *J* = 17.5, 11.0 Hz, 1H), 5.2 (d, *J* = 17.7 Hz, 1H), 5.2 (d, *J* = 11.0 Hz, 1H), 4.0 (m, 1H), 3.8 (m, 1H), 2.6 (m, 1H), 2.3 m, 1H), 1.9 (m, 1H), 1.8 (m, 1H), 1.3 (s, 3H), 1.3 (s, 3H).

^{13}C NMR (126 MHz, Chloroform-*d*) δ 165.2, 141.6, 132.9, 131.2, 129.5, 128.5, 114.1, 80.2, 71.3, 57.8, 43.3, 35.1, 31.9, 24.1.

HRMS (EI) m/z: $[M]^+$ Calcd. for $C_{16}H_{20}O_3^+$ 260.1412; Found: 260.1412.

tert-butyl 4-(benzoyloxy)-4-vinylpiperidine-1-carboxylate (1t)

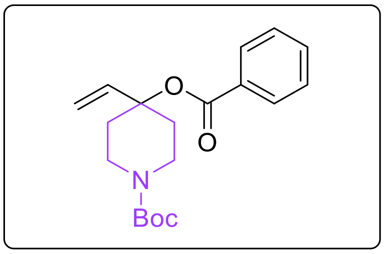

Following the **general procedure B** on 10 mmol scale, yellow oil, yield: 31% (1.03 g). R_f = 0.4 (silica gel, hexane: EtOAc = 10:1, v/v), column chromatography (neutral alumina, hexane: EtOAc = 50: 1, v/v).

^{1}H NMR (500 MHz, Chloroform-*d*) δ 8.0 – 8.0 (m, 2H), 7.6 – 7.5 (m, 1H), 7.4 (m, 2H), 6.2 (dd, *J* = 17.6, 11.0 Hz, 1H), 5.3 – 5.2 (m, 2H), 3.9 (s, 2H), 3.2 – 3.1 (m, 2H), 2.4 (m, 2H), 1.8 (m, 2H), 1.5 (s, 9H).

^{13}C NMR (126 MHz, Chloroform-*d*) δ 165.0, 154.8, 140.5, 133.0, 131.0, 129.5, 128.4, 114.8, 80.2, 79.7, 34.4, 28.4.

HRMS (EI) m/z: $[M]^+$ Calcd. for $C_{19}H_{25}NO_4^+$ 331.1784; Found: 331.1790.

1,1-dioxido-4-vinyltetrahydro-2H-thiopyran-4-yl benzoate (1u)

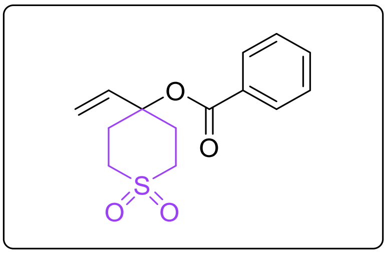

Following the **general procedure B** on 10 mmol scale, yellow oil, yield: 44% (1.23 g). R_f = 0.8 (silica gel, hexane: EtOAc = 10:1, v/v), column chromatography (neutral alumina, hexane: EtOAc = 90: 1, v/v).

^{1}H NMR (400 MHz, Chloroform-*d*) δ 7.9 (m, 2H), 7.6 – 7.5 (m, 1H), 7.4 – 7.4 (m, 2H), 6.1 (dd, *J* = 17.6, 11.1 Hz, 1H), 5.3 (d, *J* = 11.9 Hz, 1H), 5.2 (d, *J* = 5.4 Hz, 1H), 3.2 (m, 2H), 3.0 – 2.8 (m, 4H), 2.5 – 2.4 (m, 2H).

^{13}C NMR (126 MHz, Chloroform-*d*) δ 164.9, 138.6, 133.6, 130.0, 129.5, 128.7, 116.2, 78.3, 47.2, 33.1.

HRMS (EI) m/z: $[M]^+$ Calcd. for $C_{14}H_{16}O_4S^+$ 280.0769; Found: 280.0767.

2-methylbut-3-en-2-yl benzoate (1v)

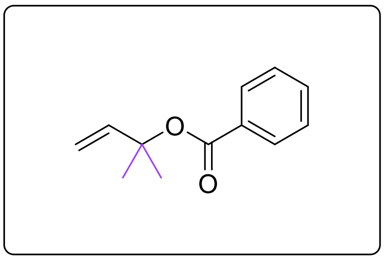

Following the **general procedure C** on 10 mmol scale, yellow oil, yield: 69% (1.31 g). R_f = 0.8 (silica gel, hexane: EtOAc = 10:1, v/v), column chromatography (neutral alumina, hexane: EtOAc = 100: 1, v/v).

^{1}H NMR (400 MHz, Chloroform-*d*) δ 8.0 – 8.0 (m, 2H), 7.6 – 7.5 (m, 1H), 7.5 – 7.4 (m, 2H), 6.2 (dd, *J* = 17.5, 10.9 Hz, 1H), 5.3 (dd, *J* = 17.6, 0.9 Hz, 1H), 5.1 (dd, *J* = 10.9, 0.8 Hz, 1H), 1.7 (s, 6H).

^{13}C NMR (101 MHz, Chloroform-*d*) δ 165.4, 142.6, 132.6, 131.6, 129.5, 128.2, 112.8, 81.3, 26.6.

HRMS (EI) m/z: $[M]^+$ Calcd. for $C_{12}H_{14}O_2^+$ 190.0994; Found: 190.0993.

3-ethylpent-1-en-3-yl benzoate (1w)

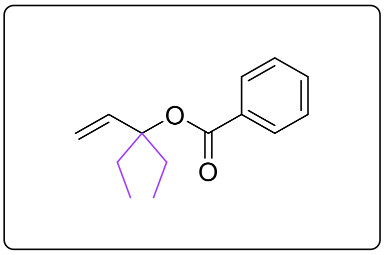

Following the **general procedure B** on 10 mmol scale, yellow oil, yield: 67% (1.27g). R_f = 0.8 (silica gel, hexane: EtOAc = 10:1, v/v), column chromatography (neutral alumina, hexane: EtOAc = 100: 1, v/v).

^{1}H NMR (500 MHz, Chloroform-*d*) δ 8.1 – 8.0 (m, 2H), 7.6 – 7.5 (m, 1H), 7.4 (m, 2H), 5.9 (dd, *J* = 17.5, 11.1 Hz, 1H), 5.3 (dd, *J* = 17.5, 1.2 Hz, 1H), 5.3 (dd, *J* = 11.0, 1.2 Hz, 1H), 2.3 (m, 2H), 1.9 (m, 7.5 Hz, 2H), 0.9 (t, *J* = 7.5 Hz, 6H).

^{13}C NMR (126 MHz, Chloroform-*d*) δ 165.0, 140.4, 132.6, 131.6, 129.5, 128.3, 114.2, 87.1, 29.0, 7.6.

HRMS (EI) m/z: $[M]^+$ Calcd. for $C_{14}H_{18}O_2^+$ 218.1307; Found: 218.1305.

2,6-dimethyl-4-vinylheptan-4-yl benzoate (1x)

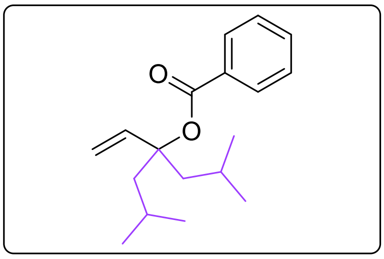

Following the **general procedure B** on 10 mmol scale, yellow oil, yield: 61% (1.67 g). R_f = 0.8 (silica gel, hexane: EtOAc = 10:1, v/v), column chromatography (neutral alumina, hexane: EtOAc = 100: 1, v/v).

^{1}H NMR (500 MHz, Chloroform-*d*) δ 8.0 (m, 2H), 7.6 – 7.5 (m, 1H), 7.4 (m, 2H), 5.9 (dd, *J* = 17.4, 11.1 Hz, 1H), 5.4 (dd, *J* = 17.4, 1.2 Hz, 1H), 5.2 (dd, *J* = 11.0, 1.3 Hz, 1H), 2.4 (m, 2H), 1.8 (m, 2H), 1.7 (m, 2H), 0.9 (m, 12H).

^{13}C NMR (126 MHz, Chloroform-*d*) δ 165.4, 142.0, 132.5, 132.0, 129.4, 128.3, 113.0, 87.1, 45.9, 24.5, 24.0, 23.7.

HRMS (EI) m/z: $[M]^+$ Calcd. for $C_{18}H_{26}O_2^+$ 274.1933; Found: 274.1931.

3-methylpent-1-en-3-yl benzoate (1y)

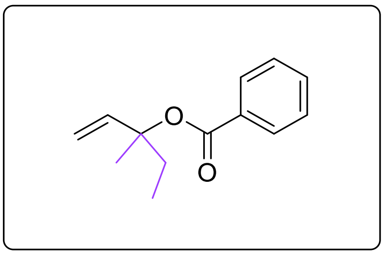

Following the **general procedure B** on 10 mmol scale, yellow oil, yield: 58% (1.18 g). R_f = 0.8 (silica gel, hexane: EtOAc = 10:1, v/v), column chromatography (neutral alumina, hexane: EtOAc = 100: 1, v/v).

^{1}H NMR (400 MHz, Chloroform-*d*) δ 8.0 – 7.9 (m, 2H), 7.5 – 7.4 (m, 1H), 7.4 – 7.3 (m, 2H), 6.0 (dd, J = 17.5, 11.0 Hz, 1H), 5.2 (dd, J = 17.5, 0.9 Hz, 1H), 5.1 (dd, J = 11.0, 0.9 Hz, 1H), 2.0 – 1.8 (m, 2H), 1.6 (s, 3H), 0.9 (t, J = 7.5 Hz, 3H).

^{13}C NMR (126 MHz, Chloroform-*d*) δ 165.3, 141.7, 132.6, 131.7, 129.5, 128.3, 113.4, 84.0, 32.9, 23.3, 8.0.

HRMS (EI) m/z: $[M]^+$ Calcd. for $C_{13}H_{16}O_2^+$ 204.1150; Found: 204.1146.

5-vinyloctahydro-1H-4,7-methanoinden-5-yl benzoate (1z)

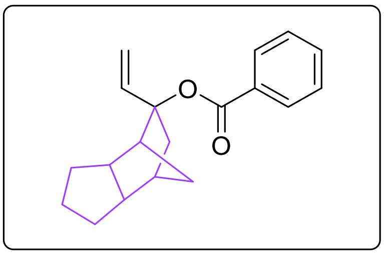

Following the **general procedure B** on 10 mmol scale, yellow oil, yield: 55% (1.55 g). R_f = 0.8 (silica gel, hexane: EtOAc = 10:1, v/v), column chromatography (neutral alumina, hexane: EtOAc = 100: 1, v/v).

^{1}H NMR (400 MHz, Chloroform-*d*) δ 8.1 – 8.0 (m, 2H), 7.6 – 7.5 (m, 1H), 7.5 – 7.4 (m, 2H), 6.2 (dd, J = 17.4, 10.8 Hz, 1H), 5.2 (dd, J = 17.5, 0.8 Hz, 1H), 5.2 (dd, J = 10.9, 0.8 Hz, 1H), 2.6 (m, 1H), 2.5 – 2.4 (m, 1H), 2.2 (m, 1H), 2.1 – 2.0 (m, 1H), 2.0 – 1.8 (m, 3H), 1.7 (m, 1H), 1.6 (m, 1H), 1.5 – 1.4 (m, 1H), 1.3 (m, 1H), 1.3 – 1.1 (m, 1H), 1.1 – 0.9 (m, 2H).

^{13}C NMR (101 MHz, Chloroform-*d*) δ 165.6, 141.8, 132.7, 131.2, 129.5, 128.3, 113.7, 87.6, 50.7, 47.5, 43.0, 40.6, 39.8, 32.5, 31.8, 30.8, 27.2.

HRMS (EI) m/z: $[M]^+$ Calcd. for $C_{19}H_{22}O_2^+$ 282.1620; Found: 282.1612.

2-vinyladamantan-2-yl benzoate (1aa)

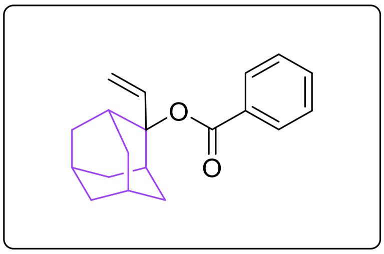

Following the **general procedure B** on 10 mmol scale, yellow oil, yield: 57% (1.61 g). R_f = 0.8 (silica gel, hexane: EtOAc = 10:1, v/v), column chromatography (neutral alumina, hexane: EtOAc = 100: 1, v/v).

^{1}H NMR (500 MHz, Chloroform-*d*) δ 8.1 – 8.0 (m, 2H), 7.6 – 7.5 (m, 1H), 7.4 (m, 2H), 6.4 (dd, J = 17.7, 11.0 Hz, 1H), 5.4 (dd, J = 17.7, 1.1 Hz, 1H), 5.3 (dd, J = 11.0, 1.1 Hz, 1H), 2.7 (m, 2H), 2.3 – 2.1 (m, 2H), 1.9 (m, 2H), 1.9 (m, 2H), 1.8 – 1.7 (m, 4H), 1.7 (m, 2H).

^{13}C NMR (126 MHz, Chloroform-*d*) δ 164.7, 140.7, 132.5, 131.8, 129.5, 128.3, 115.8, 85.7, 37.7, 35.3, 34.4, 33.1, 27.2, 26.9.

HRMS (EI) m/z: $[M]^+$ Calcd. for $C_{19}H_{22}O_2^+$ 282.1620; Found: 282.1621.

2-vinylbicyclo[2.2.1]heptan-2-yl benzoate (1ab)

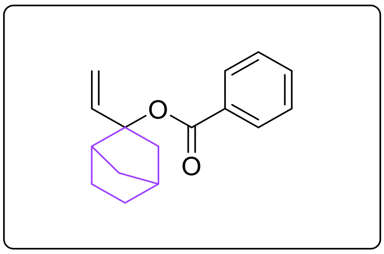

Following the **general procedure B** on 10 mmol scale, yellow oil, yield: 58% (1.41 g). R_f = 0.8 (silica gel, hexane: EtOAc = 10:1, v/v), column chromatography (neutral alumina, hexane: EtOAc = 100: 1, v/v).

^{1}H NMR (400 MHz, Chloroform-*d*) δ 8.1 – 8.0 (m, 2H), 7.6 – 7.5 (m, 1H), 7.4 (m, 2H), 6.2 (dd, *J* = 17.4, 10.9 Hz, 1H), 5.2 (d, *J* = 17.5 Hz, 1H), 5.1 (d, *J* = 10.9 Hz, 1H), 2.8 (m, 1H), 2.3 (m, 1H), 2.2 – 2.2 (m, 1H), 1.9 – 1.8 (m, 1H), 1.7 (m, 1H), 1.6 – 1.5 (m, 3H), 1.4 – 1.3 (m, 2H).

^{13}C NMR (126 MHz, Chloroform-*d*) δ 165.7, 141.6, 132.7, 131.2, 129.5, 128.3, 113.5, 88.1, 46.5, 43.3, 37.0, 36.2, 28.9, 22.5.

HRMS (EI) m/z: $[M]^+$ Calcd. for $C_{16}H_{18}O_2^+$ 242.1307; Found: 242.1302.

(2S,5R)-2-isopropyl-5-methyl-1-vinylcyclohexyl benzoate (1ac)

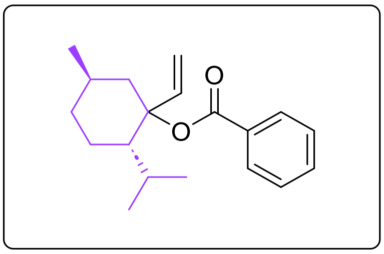

Following the **general procedure B** on 10 mmol scale, yellow oil, yield: 59% (1.69 g). R_f = 0.8 (silica gel, hexane: EtOAc = 10:1, v/v), column chromatography (neutral alumina, hexane: EtOAc = 100: 1, v/v).

^{1}H NMR (400 MHz, Chloroform-*d*) δ 8.1 – 8.0 (m, 2H), 7.6 – 7.5 (m, 1H), 7.4 (m, 2H), 6.2 (dd, *J* = 17.8, 11.3 Hz, 1H), 5.2 (dd, *J* = 11.3, 0.7 Hz, 1H), 5.1 (dd, *J* = 17.7, 0.7 Hz, 1H), 2.9 (m, 1H), 2.3 (m, 1H), 1.9 – 1.8 (m, 2H), 1.7 – 1.7 (m, 1H), 1.5 (m, 1H), 1.3 – 1.3 (m, 2H), 1.0 (m, 3H), 1.0 – 1.0 (m, 1H), 0.9 (m, 3H), 0.9 (m, 3H).

^{13}C NMR (126 MHz, Chloroform-*d*) δ 165.2, 142.4, 132.7, 131.6, 129.5, 128.4, 112.1, 87.1, 53.6, 41.6, 35.0, 27.8, 26.5, 23.8, 22.2, 21.2, 18.3.

HRMS (EI) m/z: $[M]^+$ Calcd. for $C_{19}H_{26}O_2^+$ 286.1933; Found: 286.1932.

1-vinylcyclohexyl 4-iodobenzoate (2a)

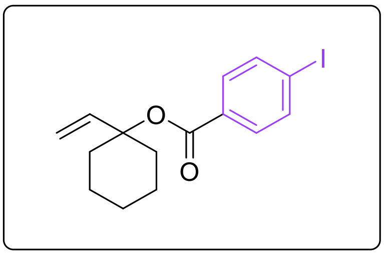

Following the **general procedure B** on 10 mmol scale, yellow oil, yield: 49% (1.74 g). R_f = 0.8 (silica gel, hexane: EtOAc = 10:1, v/v), column chromatography (neutral alumina, hexane: EtOAc = 100: 1, v/v).

^{1}H NMR (400 MHz, Chloroform-*d*) δ 7.8 (m, 2H), 7.7 (m, 2H), 6.2 (dd, *J* = 17.6, 11.0 Hz, 1H), 5.2 (d, *J* = 17.6 Hz, 1H), 5.2 (d, *J* = 11.0 Hz, 1H), 2.4 – 2.3 (m, 2H), 1.7 – 1.6 (m, 7H), 1.4 – 1.3 (m, 1H).

^{13}C NMR (101 MHz, Chloroform-*d*) δ 164.6, 141.7, 137.6, 131.1, 131.0, 114.0, 100.3, 82.9, 34.9, 25.3, 22.0.

HRMS (EI) m/z: $[M]^+$ Calcd. for $C_{12}H_{13}IO_2^+$ 356.0273; Found: 356.0272.

1-vinylcyclohexyl 4-(trifluoromethoxy)benzoate (2b)

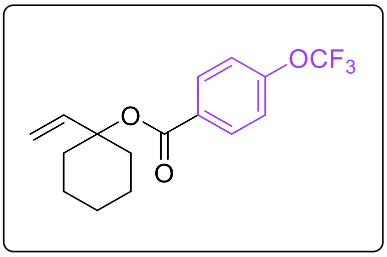

Following the **general procedure B** on 10 mmol scale, yellow oil, yield: 41% (1.29 g). R_f = 0.8 (silica gel, hexane: EtOAc = 10:1, v/v), column chromatography (neutral alumina, hexane: EtOAc = 100: 1, v/v).

^{1}H NMR (500 MHz, Chloroform-*d*) δ 8.4 (m, 2H), 7.6 – 7.6 (m, 2H), 6.5 (dd, *J* = 17.6, 11.0 Hz, 1H), 5.6 (d, *J* = 17.7 Hz, 1H), 5.5 (d, *J* = 11.0 Hz, 1H), 2.7 (m 2H), 2.0 – 1.9 (m, 7H), 1.7 – 1.6 (m, 1H).

^{13}C NMR (126 MHz, Chloroform-*d*) δ 163.9, 152.4, 141.7, 131.4, 130.0, 120.2, 120.0 (q, *J* = 250.0 Hz,), 114.0, 83.0, 34.9, 25.3, 22.0.

^{19}F NMR (471 MHz, Chloroform-*d*) δ -57.7.

HRMS (EI) m/z: $[M]^+$ Calcd. for $C_{16}H_{17}F_3O_2^+$ 314.1130; Found: 314.1129.

1-vinylcyclohexyl 4-(methylsulfonyl)benzoate (2c)

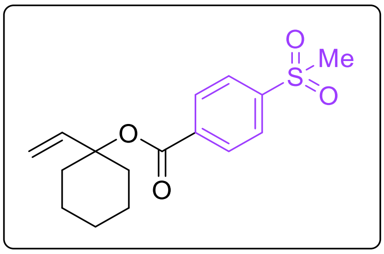

Following the **general procedure B** on 10 mmol scale, yellow oil, yield: 41% (1.26 g). R_f = 0.8 (silica gel, hexane: EtOAc = 10:1, v/v), column chromatography (neutral alumina, hexane: EtOAc = 100: 1, v/v).

^{1}H NMR (400 MHz, Chloroform-*d*) δ 8.2 (m, 2H), 8.0 (d, *J* = 8.5 Hz, 2H), 6.2 (dd, *J* = 17.6, 11.0 Hz, 1H), 5.2 (dd, *J* = 17.7, 0.7 Hz, 1H), 5.2 (dd, *J* = 11.0, 0.7 Hz, 1H), 3.1 (s, 3H), 2.4 – 2.3 (m, 2H), 1.7 – 1.6 (m, 7H), 1.4 – 1.3 (m, 1H).

^{13}C NMR (101 MHz, Chloroform-*d*) δ 163.3, 143.9, 141.2, 136.4, 130.4, 127.5, 114.4, 83.8, 44.4, 34.8, 25.3, 22.0.

HRMS (EI) m/z: $[M]^+$ Calcd. for $C_{16}H_{20}O_4S^+$ 308.1082; Found: 308.1088.

methyl (2-methylbut-3-en-2-yl) terephthalate (2d)

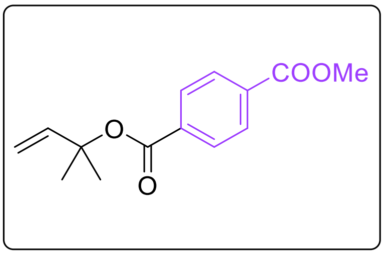

Following the **general procedure C** on 10 mmol scale, yellow oil, yield: 63% (1.56 g). R_f = 0.7 (silica gel, hexane: EtOAc = 10:1, v/v), column chromatography (neutral alumina, hexane: EtOAc = 80: 1, v/v).

^{1}H NMR (400 MHz, Chloroform-*d*) δ 8.1 – 8.0 (m, 4H), 6.2 (dd, *J* = 17.5, 10.9 Hz, 1H), 5.3 (dd, *J* = 17.5, 0.7 Hz, 1H), 5.2 (dd, *J* = 10.9, 0.7 Hz, 1H), 4.0 (s, 3H), 1.7 (s, 6H).

^{13}C NMR (126 MHz, Chloroform-*d*) δ 166.4, 164.5, 142.2, 135.4, 133.6, 129.4, 113.2, 82.0, 52.4, 26.5.

HRMS (EI) m/z: $[M]^+$ Calcd. for $C_{14}H_{16}O_4^+$ 248.1049; Found: 248.1041.

2-methylbut-3-en-2-yl 4-cyanobenzoate (2e)

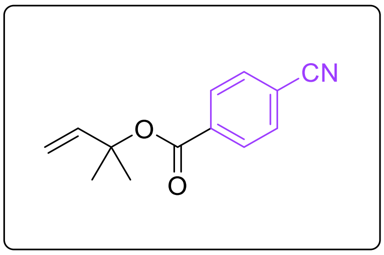

Following the **general procedure C** on 10 mmol scale, yellow oil, yield: 48% (1.03 g). R_f = 0.7 (silica gel, hexane: EtOAc = 10:1, v/v), column chromatography (neutral alumina, hexane: EtOAc = 80: 1, v/v).

^{1}H NMR (400 MHz, Chloroform-*d*) δ 8.1 (m, 2H), 7.7 – 7.7 (m, 2H), 6.2 (dd, *J* = 17.5, 10.9 Hz, 1H), 5.3 – 5.2 (m, 1H), 5.2 (dd, *J* = 10.9, 0.7 Hz, 1H), 1.7 (s, 6H).

^{13}C NMR (126 MHz, Chloroform-*d*) δ 163.6, 141.8, 135.5, 132.1, 130.0, 118.1, 116.0, 113.5, 82.6, 26.5.

HRMS (EI) m/z: $[M]^+$ Calcd. for $C_{13}H_{13}NO_2^+$ 215.0946; Found: 215.0949.

2-methylbut-3-en-2-yl 4-nitrobenzoate (2f)

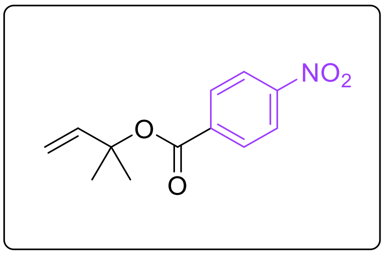

Following the **general procedure C** on 10 mmol scale, yellow oil, yield: 52% (1.22 g). R_f = 0.7 (silica gel, hexane: EtOAc = 10:1, v/v), column chromatography (neutral alumina, hexane: EtOAc = 80: 1, v/v).

^{1}H NMR (400 MHz, Chloroform-*d*) δ 8.3 (m, 2H), 8.2 (m, 2H), 6.2 (dd, *J* = 17.5, 10.9 Hz, 1H), 5.3 (dd, *J* = 17.5, 0.7 Hz, 1H), 5.2 (dd, *J* = 10.9, 0.7 Hz, 1H), 1.7 (s, 6H).

^{13}C NMR (101 MHz, Chloroform-*d*) δ 163.4, 150.3, 141.8, 137.0, 130.6, 123.4, 113.6, 82.8, 26.4.

HRMS (EI) m/z: $[M]^+$ Calcd. for $C_{12}H_{13}NO_4^+$ 235.0845; Found: 235.0842.

2-methylbut-3-en-2-yl 4-iodobenzoate (2g)

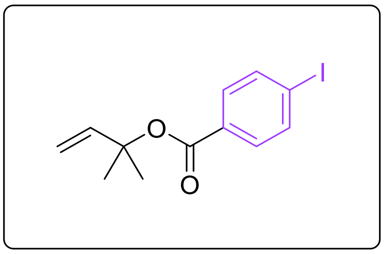

Following the **general procedure C** on 10 mmol scale, yellow oil, yield: 67% (2.11 g). R_f = 0.8 (silica gel, hexane: EtOAc = 10:1, v/v), column chromatography (neutral alumina, hexane: EtOAc = 100: 1, v/v).

^{1}H NMR (400 MHz, Chloroform-*d*) δ 7.8 (m, 2H), 7.7 (m, 2H), 6.2 (dd, *J* = 17.5, 10.9 Hz, 1H), 5.3 (dd, *J* = 17.6, 0.8 Hz, 1H), 5.1 (dd, *J* = 10.9, 0.8 Hz, 1H), 1.7 (s, 6H).

^{13}C NMR (126 MHz, Chloroform-*d*) δ 164.9, 142.3, 137.6, 131.1, 131.0, 113.1, 100.3, 81.8, 26.6.

HRMS (EI) m/z: $[M]^+$ Calcd. for $C_{12}H_{13}IO_2^+$ 315.9960; Found: 315.9961.

2-methylbut-3-en-2-yl 4-(chloromethyl)benzoate (2h)

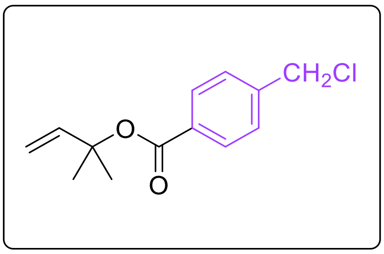

Following the **general procedure C** on 10 mmol scale, yellow oil, yield: 44% (1.05 g). R_f = 0.8 (silica gel, hexane: EtOAc = 10:1, v/v), column chromatography (neutral alumina, hexane: EtOAc = 100: 1, v/v).

^{1}H NMR (400 MHz, Chloroform-*d*) δ 7.9 – 7.8 (m, 2H), 7.3 – 7.2 (m, 2H), 6.0 (dd, *J* = 17.5, 10.9 Hz, 1H), 5.1 (dd, *J* = 17.5, 0.8 Hz, 1H), 5.0 (dd, *J* = 10.9, 0.8 Hz, 1H), 4.4 (s, 2H), 1.5 (s, 6H).

^{13}C NMR (126 MHz, Chloroform-*d*) δ 164.9, 142.4, 141.9, 131.6, 129.9, 128.4, 113.0, 45.4, 26.6, 26.6.

HRMS (EI) m/z: $[M]^+$ Calcd. for $C_{13}H_{15}ClO_2^+$ 238.0761; Found: 238.0766.

2-methylbut-3-en-2-yl 4-(trifluoromethoxy)benzoate (2i)

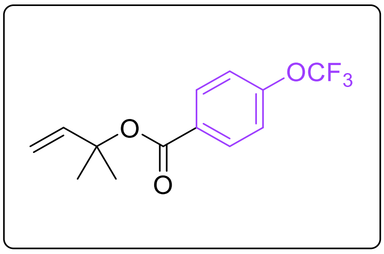

Following the **general procedure C** on 10 mmol scale, yellow oil, yield: 61% (1.67 g). R_f = 0.8 (silica gel, hexane: EtOAc = 10:1, v/v), column chromatography (neutral alumina, hexane: EtOAc = 100: 1, v/v).

^{1}H NMR (400 MHz, Chloroform-*d*) δ 8.0 (m, 2H), 7.3 – 7.2 (m, 2H), 6.2 (dd, *J* = 17.5, 10.9 Hz, 1H), 5.3 (dd, *J* = 17.5, 0.8 Hz, 1H), 5.1 (dd, *J* = 10.9, 0.8 Hz, 1H), 1.7 (s, 6H).

^{13}C NMR (126 MHz, Chloroform-*d*) δ 164.2, 152.4, 142.3, 131.4, 130.1, 120.3 (q, *J* = 258.4 Hz), 120.2, 113.1, 81.8, 26.5.

^{19}F NMR (377 MHz, Chloroform-*d*) δ -57.7.

HRMS (EI) m/z: $[M]^+$ Calcd. for $C_{13}H_{13}F_3O_3^+$ 274.0817; Found: 274.0811.

2-methylbut-3-en-2-yl 4-ethoxybenzoate (2j)

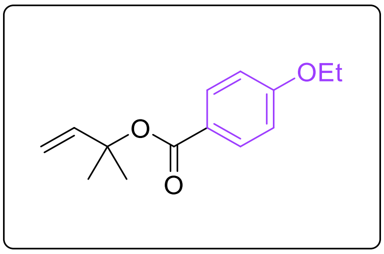

Following the **general procedure C** on 10 mmol scale, yellow oil, yield: 69% (1.61 g). R_f = 0.7 (silica gel, hexane: EtOAc = 10:1, v/v), column chromatography (neutral alumina, hexane: EtOAc = 80: 1, v/v).

^{1}H NMR (400 MHz, Chloroform-*d*) δ 7.9 (m, 2H), 6.9 (m, 2H), 6.2 (dd, *J* = 17.5, 10.9 Hz, 1H), 5.3 (dd, *J* = 17.5, 0.9 Hz, 1H), 5.1 (dd, *J* = 10.9, 0.9 Hz, 1H), 4.1 (q, *J* = 7.0 Hz, 2H), 1.6 (s, 6H), 1.4 (t, *J* = 7.0 Hz, 3H).

^{13}C NMR (126 MHz, Chloroform-*d*) δ 165.2, 162.5, 142.9, 131.5, 123.9, 113.9, 112.6, 80.8, 63.6, 26.7, 14.7.

HRMS (EI) m/z: $[M]^+$ Calcd. for $C_{14}H_{18}O_3^+$ 234.1256; Found: 234.1251.

methyl (2-methylbut-3-en-2-yl) phthalate (2k)

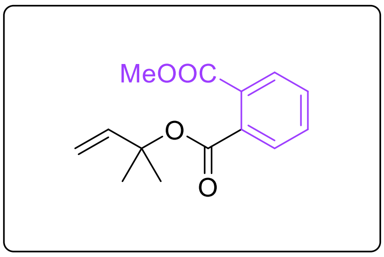

Following the **general procedure C** on 10 mmol scale, yellow oil, yield: 53% (1.31g). R_f = 0.7 (silica gel, hexane: EtOAc = 10:1, v/v), column chromatography (neutral alumina, hexane: EtOAc = 80: 1, v/v).

^{1}H NMR (400 MHz, Chloroform-*d*) δ 7.7 – 7.6 (m, 2H), 7.5 – 7.5 (m, 2H), 6.2 (dd, *J* = 17.5, 10.9 Hz, 1H), 5.2 (dd, *J* = 17.5, 0.8 Hz, 1H), 5.1 (dd, *J* = 10.9, 0.8 Hz, 1H), 3.9 (s, 3H), 1.6 (s, 6H).

^{13}C NMR (126 MHz, Chloroform-*d*) δ 168.2, 166.2, 142.2, 133.2, 131.9, 130.9, 130.7, 129.0, 128.8, 113.1, 82.5, 52.5, 26.3.

HRMS (EI) m/z: $[M]^+$ Calcd. for $C_{14}H_{16}O_4^+$ 248.1049; Found: 248.1040.

2-methylbut-3-en-2-yl 3,5-dimethylbenzoate (2l)

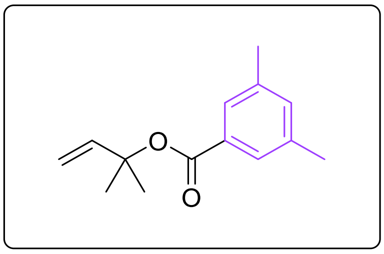

Following the **general procedure C** on 10 mmol scale, yellow oil, yield: 59% (1.29 g). R_f = 0.8 (silica gel, hexane: EtOAc = 10:1, v/v), column chromatography (neutral alumina, hexane: EtOAc = 100: 1, v/v).

^{1}H NMR (400 MHz, Chloroform-*d*) δ 7.7 – 7.6 (m, 2H), 7.2 – 7.1 (m, 1H), 6.2 (dd, *J* = 17.5, 10.9 Hz, 1H), 5.3 (dd, *J* = 17.5, 0.8 Hz, 1H), 5.1 (dd, *J* = 11.0, 0.8 Hz, 1H), 2.4 – 2.3 (m, 6H), 1.7 (s, 6H).

^{13}C NMR (126 MHz, Chloroform-*d*) δ 165.8, 142.7, 137.9, 134.2, 131.5, 127.2, 112.7, 81.1, 26.6, 21.2.

HRMS (EI) m/z: $[M]^+$ Calcd. for $C_{14}H_{18}O_2^+$ 218.1307; Found: 218.1300.

2-methylbut-3-en-2-yl 2-chloro-5-fluorobenzoate (2m)

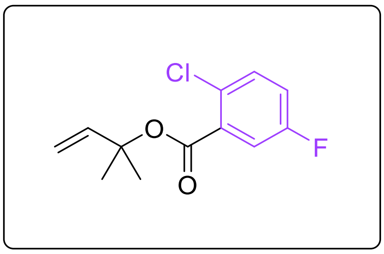

Following the **general procedure C** on 10 mmol scale, yellow oil, yield: 61% (1.47 g). R_f = 0.8 (silica gel, hexane: EtOAc = 10:1, v/v), column chromatography (neutral alumina, hexane: EtOAc = 100: 1, v/v).

^{1}H NMR (400 MHz, Chloroform-*d*) δ 7.8 (m, 1H), 7.4 (m, 1H), 7.1 (m, 1H), 6.2 (dd, *J* = 17.5, 10.9 Hz, 1H), 5.3 (dd, *J* = 17.5, 0.8 Hz, 1H), 5.1 (dd, *J* = 10.9, 0.8 Hz, 1H), 1.7 (s, 6H).

^{13}C NMR (126 MHz, Chloroform-*d*) δ 161.9 (d, *J* = 4.0 Hz), 160.4 (d, *J* = 259.7 Hz), 141.9, 133.7 (d, *J* = 9.1 Hz), 131.6, 129.1 (d, *J* = 3.7 Hz), 121.5 (d, *J* = 11.5 Hz), 118.4 (d, *J* = 24.6 Hz), 113.4, 82.8, 26.5.

^{19}F NMR (377 MHz, Chloroform-*d*) δ -112.4.

HRMS (EI) m/z: $[M]^+$ Calcd. for $C_{12}H_{12}FClO_2^+$ 242.0510; Found: 242.0506.

2-methylbut-3-en-2-yl 3,4-dichlorobenzoate (2n)

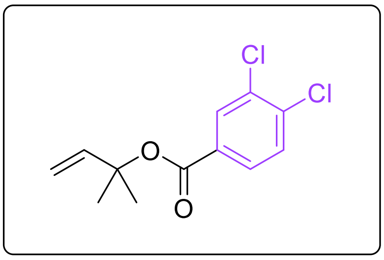

Following the **general procedure C** on 10 mmol scale, yellow oil, yield: 68% (1.75 g). R_f = 0.8 (silica gel, hexane: EtOAc = 10:1, v/v), column chromatography (neutral alumina, hexane: EtOAc = 100: 1, v/v).

^{1}H NMR (400 MHz, Chloroform-*d*) δ 8.1 (m, 1H), 7.8 (m, 1H), 7.5 (m, 1H), 6.2 (dd, *J* = 17.5, 10.9 Hz, 1H), 5.3 (dd, *J* = 17.5, 0.8 Hz, 1H), 5.2 (dd, *J* = 10.9, 0.8 Hz, 1H), 1.7 (s, 6H).

^{13}C NMR (101 MHz, Chloroform-*d*) δ 163.5, 142.0, 137.2, 132.7, 131.5, 131.4, 130.4, 128.6, 113.4, 82.3, 26.5.

HRMS (EI) m/z: $[M]^+$ Calcd. for $C_{12}H_{12}Cl_2O_2^+$ 258.0214; Found: 258.0209.

2-methylbut-3-en-2-yl 3,4-difluorobenzoate (2o)

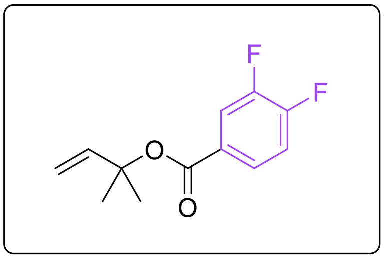

Following the **general procedure C** on 10 mmol scale, yellow oil, yield: 69% (1.56 g). R_f = 0.8 (silica gel, hexane: EtOAc = 10:1, v/v), column chromatography (neutral alumina, hexane: EtOAc = 100: 1, v/v).

^{1}H NMR (400 MHz, Chloroform-*d*) δ 7.8 – 7.7 (m, 2H), 7.2 (m, 1H), 6.2 (dd, *J* = 17.5, 10.9 Hz, 1H), 5.3 (dd, *J* = 17.5, 0.8 Hz, 1H), 5.1 (dd, *J* = 10.9, 0.7 Hz, 1H), 1.7 (s, 6H).

^{13}C NMR (126 MHz, Chloroform-*d*) δ 163.4, 153.4 (dd, *J* = 255.5, 12.8 Hz), 150.0 (dd, *J* = 249.5, 13.0 Hz), 142.1, 128.6 (d, *J* = 4.6 Hz), 126.3 (dd, *J* = 7.3, 3.7 Hz), 118.8 (d, *J* = 18.4 Hz), 117.1 (d, *J* = 17.8 Hz), 113.2, 82.1, 26.5.

^{19}F NMR (377 MHz, Chloroform-*d*) δ -131.1 (d, *J* = 20.8 Hz), -136.9 (d, *J* = 21.0 Hz).

HRMS (EI) m/z: $[M]^+$ Calcd. for $C_{12}H_{12}F_2O_2^+$ 226.0805; Found: 226.0802.

2-methylbut-3-en-2-yl 4-chloro-3-methylbenzoate (2p)

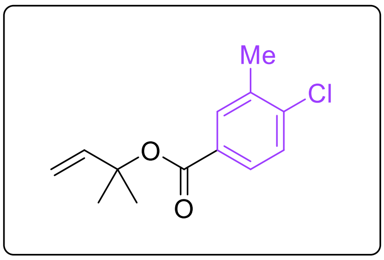

Following the **general procedure C** on 10 mmol scale, yellow oil, yield: 59% (1.40 g). R_f = 0.8 (silica gel, hexane: EtOAc = 10:1, v/v), column chromatography (neutral alumina, hexane: EtOAc = 100: 1, v/v).

^{1}H NMR (400 MHz, Chloroform-*d*) δ 7.9 (m, 1H), 7.7 (m, 1H), 7.4 (m, 1H), 6.2 (dd, *J* = 17.5, 10.9 Hz, 1H), 5.3 (dd, *J* = 17.5, 0.8 Hz, 1H), 5.1 (dd, *J* = 10.9, 0.8 Hz, 1H), 2.4 (s, 3H), 1.7 (s, 6H).

^{13}C NMR (126 MHz, Chloroform-*d*) δ 164.8, 142.4, 139.1, 136.1, 131.9, 130.0, 129.0, 128.2, 113.0, 81.6, 26.6, 20.0.

HRMS (EI) m/z: $[M]^+$ Calcd. for $C_{13}H_{15}ClO_2^+$ 238.0761; Found: 238.0756.

2-methylbut-3-en-2-yl 2-chloro-5-(trifluoromethyl)benzoate (2q)

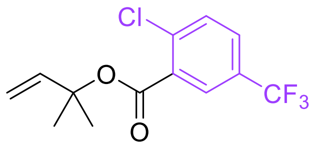

Following the **general procedure C** on 10 mmol scale, yellow oil, yield: 56% (1.64 g). R_f = 0.8 (silica gel, hexane: EtOAc = 10:1, v/v), column chromatography (neutral alumina, hexane: EtOAc = 100: 1, v/v).

^{1}H NMR (400 MHz, Chloroform-*d*) δ 8.0 (m, 1H), 7.6 – 7.6 (m, 1H), 7.5 (m, 1H), 6.2 (dd, *J* = 17.5, 10.9 Hz, 1H), 5.3 (dd, *J* = 17.5, 0.7 Hz, 1H), 5.2 (dd, *J* = 10.9, 0.7 Hz, 1H), 1.7 (s, 6H).

^{13}C NMR (126 MHz, Chloroform-*d*) δ 163.4, 141.7, 137.1, 132.4, 131.6, 129.2 (q, *J* = 33.0, 32.5 Hz), 128.5 (q, *J* = 3.7 Hz), 128.1 (q, *J* = 3.8 Hz), 123.3 (q, *J* = 272.4 Hz), 113.7, 83.5, 26.4.

^{19}F NMR (377 MHz, Chloroform-*d*) δ -62.8.

HRMS (EI) m/z: $[M]^+$ Calcd. for $C_{13}H_{12}ClF_3O_2^+$ 292.0478; Found: 292.0472.

2-methylbut-3-en-2-yl 6-bromo-2-naphthoate (2r)

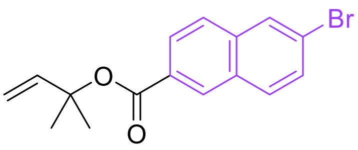

Following the **general procedure C** on 10 mmol scale, yellow oil, yield: 61% (1.94g). R_f = 0.8 (silica gel, hexane: EtOAc = 10:1, v/v), column chromatography (neutral alumina, hexane: EtOAc = 100: 1, v/v).

^{1}H NMR (500 MHz, Chloroform-*d*) δ 8.5 – 8.5 (m, 1H), 8.1 – 8.0 (m, 2H), 7.8 (m, 2H), 7.6 (dd, *J* = 8.7, 2.0 Hz, 1H), 6.3 (dd, *J* = 17.5, 10.9 Hz, 1H), 5.3 (dd, *J* = 17.5, 0.8 Hz, 1H), 5.2 (dd, *J* = 10.9, 0.8 Hz, 1H), 1.7 (s, 6H).

^{13}C NMR (126 MHz, Chloroform-*d*) δ 165.2, 142.5, 136.3, 130.9, 130.8, 130.6, 130.1, 129.9, 129.3, 127.1, 126.5, 122.4, 113.1, 81.7, 26.7.

HRMS (EI) m/z: $[M]^+$ Calcd. for $C_{16}H_{15}BrO_2^+$ 318.0255; Found: 318.0254.

2-methylbut-3-en-2-yl thiophene-2-carboxylate (2s)

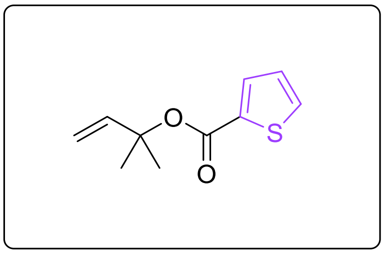

Following the **general procedure C** on 10 mmol scale, yellow oil, yield: 70% (1.37 g). R_f = 0.8 (silica gel, hexane: EtOAc = 10:1, v/v), column chromatography (neutral alumina, hexane: EtOAc = 100: 1, v/v).

^{1}H NMR (500 MHz, Chloroform-*d*) δ 7.7 (m, 1H), 7.5 (m, 1H), 7.1 (m, 1H), 6.2 (dd, *J* = 17.5, 10.9 Hz, 1H), 5.3 (dd, *J* = 17.5, 0.8 Hz, 1H), 5.1 (dd, *J* = 10.8, 0.8 Hz, 1H), 1.6 (s, 6H).

^{13}C NMR (126 MHz, Chloroform-*d*) δ 161.1, 142.3, 135.4, 132.9, 131.9, 127.6, 113.0, 82.0, 26.6.

HRMS (EI) m/z: $[M]^+$ Calcd. for $C_{10}H_{12}O_2S^+$ 195.0558; Found: 195.0552.

2-methyl 5-(2-methylbut-3-en-2-yl) thiophene-2,5-dicarboxylate (2t)

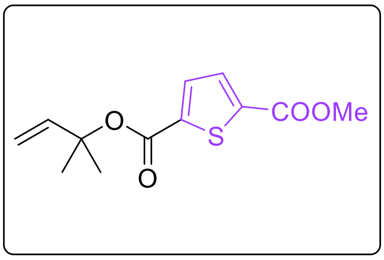

Following the **general procedure C** on 10 mmol scale, yellow oil, yield: 72% (1.83 g). R_f = 0.8 (silica gel, hexane: EtOAc = 10:1, v/v), column chromatography (neutral alumina, hexane: EtOAc = 100: 1, v/v).

^{1}H NMR (500 MHz, Chloroform-*d*) δ 7.7 (m, 1H), 7.7 (mz, 1H), 6.1 (dd, *J* = 17.5, 10.9 Hz, 1H), 5.3 (dd, *J* = 17.4, 0.8 Hz, 1H), 5.1 (dd, *J* = 10.9, 0.8 Hz, 1H), 3.9 (s, 3H), 1.6 (s, 6H).

^{13}C NMR (126 MHz, Chloroform-*d*) δ 162.1, 160.4, 141.8, 140.7, 138.2, 133.0, 132.6, 113.4, 82.9, 52.5, 26.5.

HRMS (EI) m/z: $[M]^+$ Calcd. for $C_{12}H_{14}O_4S^+$ 254.0613; Found: 254.0602.

2-methylbut-3-en-2-yl 9,10-dioxo-9,10-dihydroanthracene-2-carboxylate (2u)

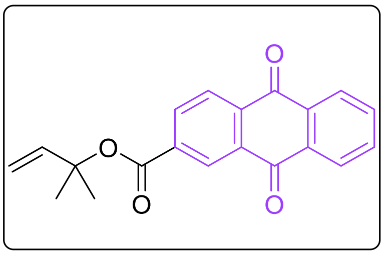

Following the **general procedure C** on 10 mmol scale, yellow oil, yield: 47% (1.50 g). $R_f = 0.8$ (silica gel, hexane: EtOAc = 10:1, v/v), column chromatography (neutral alumina, hexane: EtOAc = 100: 1, v/v).

^{1}H NMR (500 MHz, Chloroform-*d*) δ 8.8 (m, 1H), 8.4 – 8.3 (m, 4H), 7.8 – 7.8 (m, 2H), 6.2 (dd, *J* = 17.5, 10.9 Hz, 1H), 5.3 (dd, *J* = 17.5, 1.8 Hz, 1H), 5.2 (dd, *J* = 10.9, 1.8 Hz, 1H), 1.7 (s, 6H).

^{13}C NMR (126 MHz, Chloroform-*d*) δ 182.6, 182.4, 163.7, 141.9, 136.7, 135.8, 134.5, 134.4, 134.4, 133.5, 133.4, 133.4, 128.4, 127.4, 127.4, 127.4, 113.6, 82.7, 26.5.

HRMS (EI) m/z: $[M]^+$ Calcd. for $C_{12}H_{16}O_4^+$ 320.1049; Found: 320.1044.

1-vinylcyclohexyl 4-(5-(2-fluorophenyl)-1,2,4-oxadiazol-3-yl)benzoate (2v)

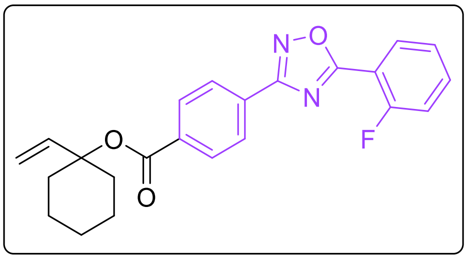

Following the **general procedure C** on 10 mmol scale, yellow oil, yield: 58% (2.27g). $R_f = 0.7$ (silica gel, hexane: EtOAc = 10:1, v/v), column chromatography (neutral alumina, hexane: EtOAc = 80: 1, v/v).

^{1}H NMR (400 MHz, Chloroform-*d*) δ 8.8 (m, 1H), 8.4 (m, 1H), 8.3 – 8.2 (m, 2H), 7.7 – 7.5 (m, 2H), 7.4 – 7.2 (m, 2H), 6.2 (dd, *J* = 17.7, 11.0 Hz, 1H), 5.3 (d, *J* = 17.7 Hz, 1H), 5.2 (d, *J* = 11.0 Hz, 1H), 2.5 – 2.3 (m, 2H), 1.7 – 1.6 (m, 7H), 1.4 – 1.3 (m, 1H).

^{13}C NMR (101 MHz, Chloroform-*d*) δ 173.0 (d, *J* = 4.5 Hz), 168.2, 164.3, 160.8 (d, *J* = 260.8 Hz), 141.7, 134.7 (d, *J* = 8.7 Hz), 132.5, 131.8 (d, *J* = 64.3 Hz), 131.0, 128.8 (d, *J* = 34.1 Hz), 127.2, 124.7 (d, *J* = 3.9 Hz), 117.2 (d, *J* = 20.8 Hz), 114.0, 112.7 (d, *J* = 11.3 Hz), 83.0, 35.0, 25.4, 22.0.

^{19}F NMR (377 MHz, Chloroform-*d*) δ -108.1.

HRMS (EI) m/z: $[M]^+$ Calcd. for $C_{23}H_{21}FN_2O_3^+$ 392.1536; Found: 392.1539.

2-methylbut-3-en-2-yl 3-methyl-4-oxo-2-phenyl-4H-chromene-6-carboxylate (2w)

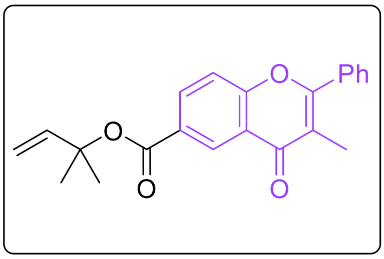

Following the **general procedure C** on 10 mmol scale, yellow oil, yield: 73% (2.54 g). $R_f = 0.8$ (silica gel, hexane: EtOAc = 10:1, v/v), column chromatography (neutral alumina, hexane: EtOAc = 100: 1, v/v).

^{1}H NMR (500 MHz, Chloroform-*d*) δ 8.4 (m, 1H), 8.1 (m, 1H), 7.8 – 7.7 (m, 2H), 7.5 – 7.5 (m, 3H), 7.4 (m, 1H), 6.2 (dd, *J* = 17.5, 10.9 Hz, 1H), 5.2 (d, *J* = 17.5 Hz, 1H), 5.1 (dd, *J* = 10.8, 0.8 Hz, 1H), 2.2 (s, 3H), 1.6 (s, 6H).

^{13}C NMR (126 MHz, Chloroform-*d*) δ 178.3, 163.7, 161.2, 154.1, 142.1, 135.7, 133.1, 130.4, 130.0, 129.3, 128.3, 124.0, 123.2, 122.6, 117.6, 113.2, 82.6, 26.5, 11.7.

HRMS (EI) m/z: $[M]^+$ Calcd. for $C_{22}H_{20}O_4^+$ 348.1362; Found: 348.1360.

2-methylbut-3-en-2-yl 4-(*N*,*N*-dipropylsulfamoyl)benzoate (2x)

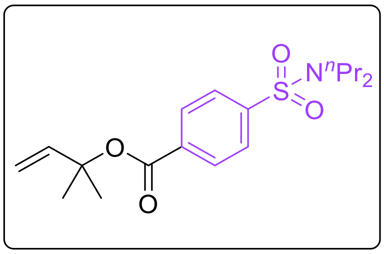

Following the **general procedure C** on 10 mmol scale, yellow oil, yield: 44% (1.67 g). R_f = 0.7 (silica gel, hexane: EtOAc = 10:1, v/v), column chromatography (neutral alumina, hexane: EtOAc = 80: 1, v/v).

^{1}H NMR (400 MHz, Chloroform-*d*) δ 8.1 (d, *J* = 8.5 Hz, 2H), 7.8 (d, *J* = 8.5 Hz, 2H), 6.2 (dd, *J* = 17.5, 10.9 Hz, 1H), 5.3 (dd, *J* = 17.5, 0.7 Hz, 1H), 5.2 (dd, *J* = 10.9, 0.7 Hz, 1H), 3.1 – 3.0 (m, 4H), 1.7 (s, 6H), 1.6 – 1.5 (m, 4H), 0.9 (t, *J* = 7.4 Hz, 6H).

^{13}C NMR (126 MHz, Chloroform-*d*) δ 164.0, 143.9, 142.0, 134.9, 130.1, 126.9, 113.4, 82.3, 49.9, 26.5, 21.9, 11.1.

HRMS (EI) m/z: $[M]^+$ Calcd. for $C_{20}H_{31}NO_4S^+$ 381.1974; Found: 381.1976.

2-methylbut-3-en-2-yl 2-(3-cyano-4-isobutylphenyl)-4-methylthiazole-5-carboxylate (2y)

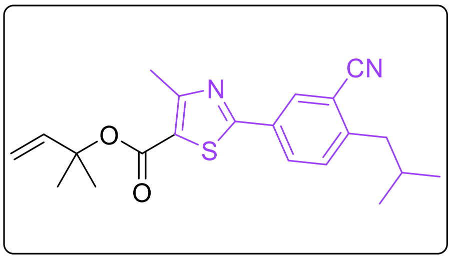

Following the **general procedure C** on 10 mmol scale, yellow oil, yield: 60% (2.21 g). R_f = 0.7 (silica gel, hexane: EtOAc = 10:1, v/v), column chromatography (neutral alumina, hexane: EtOAc = 80: 1, v/v).

^{1}H NMR (500 MHz, Chloroform-*d*) δ 8.2 (m, 1H), 8.1 (m, 1H), 7.0 (m, 1H), 6.2 (dd, *J* = 17.5, 10.9 Hz, 1H), 5.3 (d, *J* = 17.5 Hz, 1H), 5.2 (d, *J* = 10.9 Hz, 1H), 3.9 (d, *J* = 6.5 Hz, 2H), 2.7 (s, 3H), 2.2 (m, 1H), 1.7 (s, 6H), 1.1 (s, 3H), 1.1 (s, 3H).

^{13}C NMR (126 MHz, Chloroform-*d*) δ 166.7, 162.4, 160.9, 160.7, 142.1, 132.5, 132.0, 126.1, 123.0, 115.4, 113.3, 112.6, 102.9, 82.7, 75.7, 28.2, 26.6, 19.1, 17.4.

HRMS (EI) m/z: $[M]^+$ Calcd. for $C_{21}H_{21}N_2O_2S^+$ 368.1558; Found: 368.1551.

1-allylcyclohexyl 4-methylbenzoate (2z)

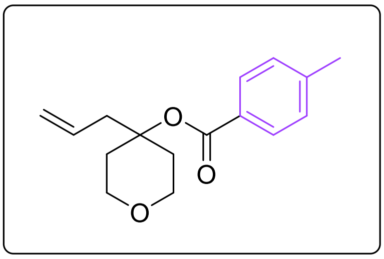

Following the **general procedure D** on 10 mmol scale, yellow oil, yield: 47% (1.22 g). R_f = 0.7 (silica gel, hexane: EtOAc = 10:1, v/v), column chromatography (neutral alumina, hexane: EtOAc = 80: 1, v/v).

^{1}H NMR (500 MHz, Chloroform-*d*) δ 7.9 (m, 2H), 7.2 (m, 2H), 5.7 – 5.8 (m, 1H), 5.2 – 5.0 (m, 2H), 3.8 (m, 2H), 3.7 (m, 2H), 2.8 (m, 2H), 2.4 (m, 3H), 2.4 (m, 2H), 1.8 (m, 2H).

^{13}C NMR (126 MHz, Chloroform-*d*) δ 165.5, 143.5, 131.9, 129.5, 129.1, 128.6, 119.0, 80.6, 63.7, 42.2, 35.0, 21.6.

HRMS (EI) m/z: $[M+Na]^+$ Calcd. for $C_{16}H_{20}O_2^+$ 283.1310; Found: 283.1305.

4-allyltetrahydro-2H-pyran-4-yl methyl terephthalate (2aa)

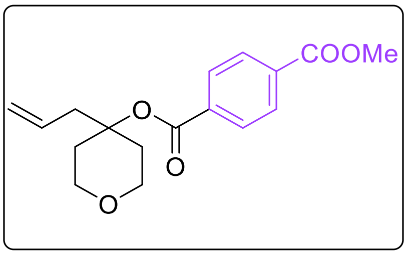

Following the **general procedure D** on 10 mmol scale, yellow oil, yield: 51% (1.48 g). R_f = 0.6 (silica gel, hexane: EtOAc = 10:1, v/v), column chromatography (neutral alumina, hexane: EtOAc = 70: 1, v/v).

^{1}H NMR (500 MHz, Chloroform-*d*) δ 8.1 (m, 2H), 8.0 (m, 2H), 5.7 (dd, *J* = 17.1, 10.0 Hz, 1H), 5.2 – 5.0 (m, 2H), 3.9 (s, 3H), 3.8 (ddd, *J* = 11.8, 4.6, 2.7 Hz, 2H), 3.7 (td, *J* = 11.6, 2.3 Hz, 2H), 2.9 – 2.8 (m, 2H), 2.4 – 2.3 (m, 2H), 1.8 (ddd, *J* = 14.2, 11.4, 4.9 Hz, 2H).

^{13}C NMR (126 MHz, Chloroform-*d*) δ 163.5, 150.5, 136.6, 131.3, 130.5, 123.6, 119.5, 82.6, 63.6, 41.8, 34.8.

HRMS (EI) m/z: $[M]^+$ Calcd. for $C_{17}H_{20}O_5^+$ 304.1311; Found: 304.1313.

1-allylcyclohexyl benzoate (2ab)

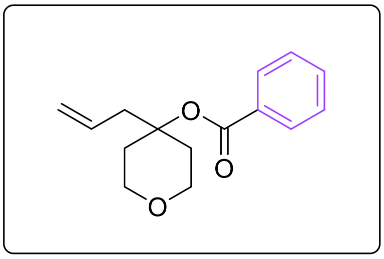

Following the **general procedure D** on 10 mmol scale, yellow oil, yield: 59% (1.45 g). R_f = 0.8 (silica gel, hexane: EtOAc = 10:1, v/v), column chromatography (neutral alumina, hexane: EtOAc = 100: 1, v/v).

^{1}H NMR (500 MHz, Chloroform-*d*) δ 8.0 – 8.0 (m, 2H), 7.6 – 7.5 (m, 1H), 7.4 (m, 2H), 5.8 (m, 1H), 5.2 – 5.0 (m, 2H), 3.8 (m, 2H), 3.7 (td, *J* = 11.6, 2.2 Hz, 2H), 2.8 (m, 2H), 2.4 (m, 2H), 1.8 (m, 2H).

^{13}C NMR (126 MHz, Chloroform-*d*) δ 165.4, 132.9, 131.8, 131.3, 129.4, 128.4, 119.1, 80.9, 63.7, 42.1, 34.9.

HRMS (EI) m/z: $[M]^+$ Calcd. for $C_{15}H_{18}O_3^+$ 246.1256; Found: 246.1258.

2-methylbut-3-en-2-yl cyclopropanecarboxylate (2ac)

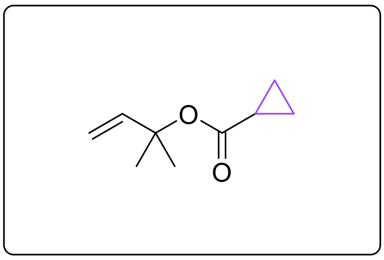

Following the **general procedure C** on 10 mmol scale, yellow oil, yield: 74% (1.14g). R_f = 0.8 (silica gel, hexane: EtOAc = 10:1, v/v), column chromatography (neutral alumina, hexane: EtOAc = 100: 1, v/v).

^{1}H NMR (400 MHz, Chloroform-*d*) δ 6.0 (dd, *J* = 17.5, 10.9 Hz, 1H), 5.1 (dd, *J* = 17.5, 0.8 Hz, 1H), 5.0 (dd, *J* = 10.9, 0.8 Hz, 1H), 1.6 – 1.5 (m, 1H), 1.5 (s, 6H), 0.9 – 0.9 (m, 2H), 0.8 – 0.7 (m, 2H).

^{13}C NMR (101 MHz, Chloroform-*d*) δ 173.7, 142.7, 112.4, 80.4, 26.5, 13.7, 8.0.

HRMS (EI) m/z: $[M]^+$ Calcd. for $C_9H_{14}O_2^+$ 154.0998; Found: 154.0987.

ethyl 2-benzoyl-4-hydroxy-4-methyl-1,8-dioxaspiro[4.5]decane-2-carboxylate (3)

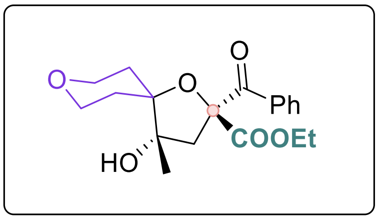

Following the **general procedure F** on 0.1 mmol scale, yellow oil, yield: 88% (30.6 mg), > 20: 1 dr. R_f = 0.3 (silica gel, hexane: EtOAc = 3:1, v/v), column chromatography (silica gel, hexane: EtOAc = 3: 1, v/v).

^{1}H NMR (400 MHz, Chloroform-*d*) δ 8.0 – 8.0 (m, 2H), 7.5 – 7.4 (m, 1H), 7.4 (m, 2H), 4.1 – 4.0 (m, 3H), 3.9 – 3.8 (m, 1H), 3.8 – 3.7 (m, 1H), 3.7 – 3.5 (m, 1H), 3.1 (d, *J* = 14.1 Hz, 1H), 2.3 (d, *J* = 14.1 Hz, 1H), 2.2 (s, 1H), 1.6 (m, 1H), 1.5 (m, 3H), 1.2 (s, 3H), 1.0 (t, *J* = 7.1 Hz, 3H).

^{13}C NMR (126 MHz, Chloroform-*d*) δ 194.8, 172.0, 134.3, 133.4, 129.8, 128.3, 88.5, 87.2, 79.6, 64.8, 63.9, 61.9, 45.5, 33.6, 30.2, 21.7, 13.8.

HRMS (EI) m/z: $[M]^+$ Calcd. for $C_{19}H_{24}O_6^+$ 348.1573; Found: 348.1568.

IR (neat, cm^{-1}) 3542, 2933, 1722, 1682, 1448, 1260, 1112, 9996, 699.

ethyl 2-benzoyl-4-hydroxy-4-methyl-1-oxaspiro[4.5]decane-2-carboxylate (4)

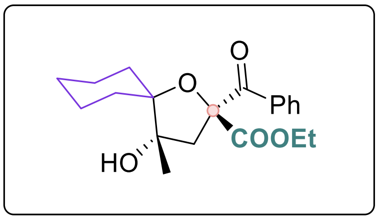

Following the **general procedure F** on 0.1 mmol scale, white solid, m.p. 140-142 ºC, yield: 83% (28.7 mg), > 20: 1 dr. R_f = 0.3 (silica gel, hexane: EtOAc = 5:1, v/v), column chromatography (silica gel, hexane: EtOAc = 5: 1, v/v).

^{1}H NMR (400 MHz, Chloroform-*d*) δ 8.1 (m, 2H), 7.6 – 7.5 (m, 1H), 7.4 (m, 2H), 4.1 (m, 2H), 3.1 (d, *J* = 14.0 Hz, 1H), 2.5 (d, *J* = 14.0 Hz, 1H), 2.2 (s, 1H), 2.0 (m, 1H), 1.8 – 1.6 (m, 4H), 1.6 (m, 2H), 1.31 – 1.26 (m, 1H), 1.26 (s, 3H), 1.17 (m, 2H), 1.02 (t, *J* = 7.1 Hz, 3H).

^{13}C NMR (126 MHz, Chloroform-*d*) δ 195.5, 172.2, 134.5, 133.1, 129.8, 128.2, 90.9, 87.1, 80.2, 61.7, 45.9, 33.6, 29.5, 25.7, 22.6, 21.9, 21.6, 13.8.

HRMS (EI) m/z: $[M]^+$ Calcd. for $C_{20}H_{26}O_5^+$ 346.1780; Found: 346.1781.

IR (neat, cm^{-1}) 3522, 2933, 1722, 1682, 1448, 1260, 1112, 956, 694.

ethyl 2-benzoyl-4-hydroxy-4-methyl-1-oxaspiro[4.6]undecane-2-carboxylate (5)

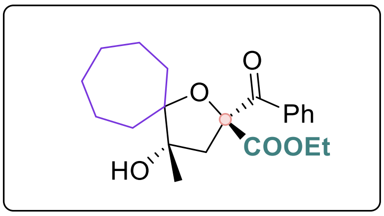

Following the **general procedure F** on 0.1 mmol scale, yellow oil, yield: 80% (28.8 mg), > 20: 1 dr. R_f = 0.3 (silica gel, hexane: EtOAc = 5:1, v/v), column chromatography (silica gel, hexane: EtOAc = 5: 1, v/v).

¹H NMR (500 MHz, Chloroform-*d*) δ 8.1 – 8.0 (m, 2H), 7.5 (m, 1H), 7.4 (m, 2H), 4.1 – 4.0 (m, 2H), 3.0 (d, *J* = 14.0 Hz, 1H), 2.6 (d, *J* = 14.0 Hz, 1H), 2.2 (s, 1H), 1.9 – 1.8 (m, 3H), 1.7 – 1.6 (m, 6H), 1.5 – 1.4 (m, 3H), 1.3 (s, 3H), 1.0 (t, *J* = 7.1 Hz, 3H).

¹³C NMR (151 MHz, Chloroform-*d*) δ 195.9, 172.0, 134.5, 133.2, 129.7, 128.3, 94.6, 87.0, 81.5, 61.8, 46.1, 36.8, 33.7, 29.8, 29.5, 23.1, 22.5, 21.3, 13.7.

HRMS (EI) m/z: $[M]^+$ Calcd. for $C_{21}H_{28}O_5^+$ 360.1937; Found: 360.1926.

IR (neat, cm^{-1}) 3526, 2919, 1724, 1682, 1445, 1261, 1109, 1025, 699.

ethyl 2-benzoyl-4-hydroxy-4-methyl-1-oxaspiro[4.14]nonadecane-2-carboxylate (6)

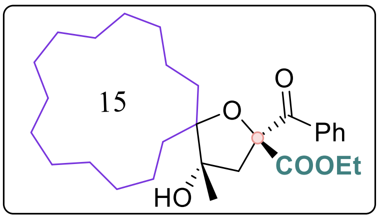

Following the **general procedure F** on 0.1 mmol scale, white solid, m.p. 133-135 ºC, yield: 84% (39.6 mg), > 20: 1 dr. R_f = 0.4 (silica gel, hexane: EtOAc = 5:1, v/v), column chromatography (silica gel, hexane: EtOAc = 6: 1, v/v).

¹H NMR (500 MHz, Chloroform-*d*) δ 8.1 – 8.0 (m, 2H), 7.6 – 7.5 (m, 1H), 7.4 (t, *J* = 7.8 Hz, 2H), 4.2 – 4.0 (m, 2H), 3.0 (d, *J* = 14.2 Hz, 1H), 2.6 (d, *J* = 14.1 Hz, 1H), 2.4 (s, 1H), 1.7 (ddd, *J* = 16.9, 8.9, 3.7 Hz, 2H), 1.7 – 1.6 (m, 2H), 1.5 – 1.4 (m, 8H), 1.4 (s, 3H), 1.4 – 1.2 (m, 16H), 1.0 (t, *J* = 7.1 Hz, 3H).

¹³C NMR (126 MHz, Chloroform-*d*) δ 195.8, 172.0, 134.4, 133.2, 129.6, 128.3, 94.0, 86.7, 80.9, 61.7, 47.4, 34.6, 31.6, 28.0, 27.0, 27.0, 26.9, 26.7, 26.6, 26.3, 26.2, 22.7, 22.1, 21.8, 13.7.

HRMS (EI) m/z: $[M]^+$ Calcd. for $C_{29}H_{44}O_5^+$ 472.3189; Found: 472.3186.

IR (neat, cm^{-1}) 3509, 2925, 1751, 1681, 1449, 1260, 1231, 1063, 936, 702.

ethyl 2-benzoyl-4-hydroxy-4,8,8-trimethyl-1-oxaspiro[4.5]decane-2-carboxylate (7)

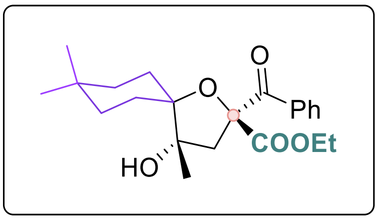

Following the **general procedure F** on 0.1 mmol scale, yellow oil, yield: 83% (31.0 mg), > 20: 1 dr. R_f = 0.3 (silica gel, hexane: EtOAc = 5:1, v/v), column chromatography (silica gel, hexane: EtOAc = 5: 1, v/v).

¹H NMR (600 MHz, Chloroform-*d*) δ 8.1 – 8.0 (m, 2H), 7.6 – 7.5 (m, 1H), 7.5 – 7.4 (m, 2H), 4.2 – 4.0 (m, 2H), 3.1 (d, *J* = 14.1 Hz, 1H), 2.5 (d, *J* = 14.1 Hz, 1H), 2.2 (s, 1H), 2.0 – 1.9 (m, 1H),

1.6 (m, 1H), 1.5 – 1.4 (m, 3H), 1.4 (m, 1H), 1.3 (s, 4H), 1.2 – 1.2 (m, 1H), 1.0 (t, *J* = 7.1 Hz, 3H), 1.0 (s, 3H), 0.9 (s, 3H).

^{13}C NMR (151 MHz, Chloroform-*d*) δ 195.4, 172.1, 134.5, 133.2, 129.8, 128.3, 90.7, 87.0, 80.3, 61.7, 45.9, 35.3, 34.7, 32.7, 29.4, 29.4, 25.8, 23.7, 21.8, 13.8.

HRMS (ESI) m/z: $[M]^+$ Calcd. for $C_{22}H_{30}O_5^+$ 374.2093; Found: 374.2096.

IR (neat, cm^{-1}) 3404, 2957, 2860, 1743, 1691, 1446, 1272, 1246, 1100, 1107, 949, 840, 695.

ethyl 2-benzoyl-4-hydroxy-4-phenyl-1-oxaspiro[4.5]decane-2-carboxylate (8)

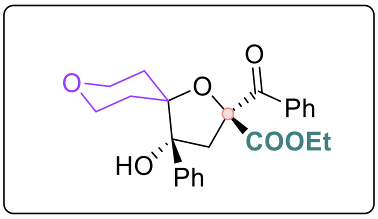

Following the **general procedure F** on 0.1 mmol scale, white solid, m.p. 168-170 ºC, yield: 93% (38.1 mg), > 20: 1 dr. R_f = 0.3 (silica gel, hexane: EtOAc = 3:1, v/v), column chromatography (silica gel, hexane: EtOAc = 3: 1, v/v).

^{1}H NMR (500 MHz, Chloroform-*d*) δ 8.1 (m, 2H), 7.6 – 7.5 (m, 1H), 7.5 – 7.4 (m, 4H), 7.4 – 7.3 (m, 3H), 4.3 – 4.1 (m, 2H), 4.0 (m, 1H), 3.8 (m, 2H), 3.7 (m, 1H), 3.4 – 3.2 (m, 2H), 2.4 (m, 1H), 1.8 (m, 1H), 1.6 (m, 1H), 1.6 – 1.5 (m, 1H), 1.1 (t, *J* = 7.1 Hz, 3H), 1.0 (m, 1H).

^{13}C NMR (126 MHz, Chloroform-*d*) δ 195.2, 171.9, 139.5, 134.4, 133.2, 130.0, 128.3, 128.3, 128.0, 126.2, 88.7, 87.8, 82.8, 64.9, 63.7, 62.2, 43.6, 34.2, 29.4, 13.9.

HRMS (ESI) m/z: $[M]^+$ Calcd. for $C_{24}H_{26}O_6^+$ 410.1729; Found: 410.1725.

IR (neat, cm^{-1}) 3522, 2863, 1753, 1691, 1444, 1279, 1241, 1099, 1066, 841, 701.

ethyl 2-benzoyl-4-hydroxy-4-phenyl-1-oxaspiro[4.6]undecane-2-carboxylate (9)

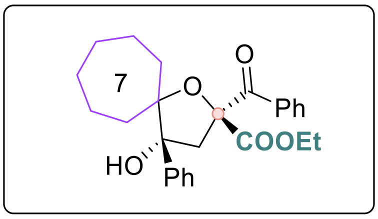

Following the **general procedure F** on 0.1 mmol scale, white solid, 143-145 ºC, yield: 84% (35.4 mg), > 20: 1 dr. R_f = 0.4 (silica gel, hexane: EtOAc = 5:1, v/v), column chromatography (silica gel, hexane: EtOAc = 6: 1, v/v).

^{1}H NMR (500 MHz, Chloroform-*d*) δ 8.1 (m, 2H), 7.6 – 7.5 (m, 3H), 7.5 – 7.4 (m, 2H), 7.4 – 7.3 (m, 2H), 7.3 – 7.3 (m, 1H), 4.3 – 4.1 (m, 2H), 3.5 (d, *J* = 13.9 Hz, 1H), 3.2 (d, *J* = 13.9 Hz, 1H), 2.4 (s, 1H), 2.1 – 2.0 (m, 1H), 1.8 – 1.8 (m, 2H), 1.6 – 1.6 (m, 3H), 1.5 (m, 1H), 1.4 – 1.3 (m, 1H), 1.3 – 1.2 (m, 3H), 1.1 (t, *J* = 7.1 Hz, 3H), 0.9 – 0.7 (m, 1H).

^{13}C NMR (126 MHz, Chloroform-*d*) δ 196.4, 171.9, 139.8, 134.6, 133.1, 129.9, 128.2, 128.1, 127.7, 126.4, 95.1, 87.7, 83.9, 62.0, 44.4, 37.3, 32.7, 29.6, 29.5, 23.1, 22.3, 13.8.

HRMS (EI) m/z: $[M]^+$ Calcd. for $C_{26}H_{33}O_5^+$ 422.2093; Found: 422.2089.

IR (neat, cm^{-1}) 3540, 2922, 1754, 1688, 1447, 1211, 1102, 1063, 978, 907, 699.

ethyl 6-benzoyl-8-hydroxy-5-oxaspiro[3.4]octane-6-carboxylate (10)

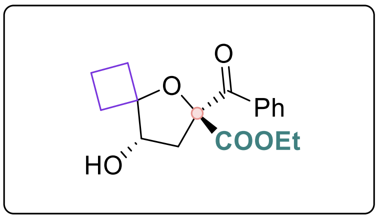

Following the **general procedure F** on 0.1 mmol scale, yellow oil, yield: 36% (11.0 mg), > 20: 1 dr. R_f = 0.3 (silica gel, hexane: EtOAc = 5:1, v/v), column chromatography (silica gel, hexane: EtOAc = 5: 1, v/v).

^{1}H NMR (500 MHz, Chloroform-*d*) δ 8.1 – 8.0 (m, 2H), 7.6 – 7.5 (m, 1H), 7.4 (m, 2H), 4.2 (d, *J* = 4.7 Hz, 1H), 4.2 – 4.0 (m, 2H), 3.1 (dd, *J* = 14.3, 0.8 Hz, 1H), 2.5 – 2.4 (m, 2H), 2.4 – 2.3 (m, 1H), 2.3 (m, 1H), 1.9 (m, 1H), 1.8 – 1.7 (m, 1H), 1.5 (m, 1H), 1.1 (t, *J* = 7.1 Hz, 3H).

^{13}C NMR (126 MHz, Chloroform-*d*) δ 194.3, 171.9, 134.2, 133.4, 129.8, 128.4, 90.4, 88.4, 75.0, 61.9, 39.5, 34.6, 28.4, 13.8, 12.0.

HRMS (EI) m/z: $[M]^+$ Calcd. for $C_{17}H_{20}O_5^+$ 304.1311; Found: 304.1307.

IR (neat, cm^{-1}) 3248, 2923, 1732, 1695, 1368, 1291, 1093, 984, 735.

ethyl 2-benzoyl-4-hydroxy-1-oxaspiro[4.4]nonane-2-carboxylate (11)

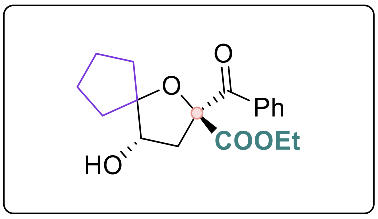

Following the **general procedure F** on 0.1 mmol scale, yellow oil, yield: 59% (18.8 mg), > 20: 1 dr. R_f = 0.3 (silica gel, hexane: EtOAc = 5:1, v/v), column chromatography (silica gel, hexane: EtOAc = 5: 1, v/v).

^{1}H NMR (400 MHz, Chloroform-*d*) δ 8.0 (dd, *J* = 8.4, 1.3 Hz, 2H), 7.6 – 7.5 (m, 1H), 7.4 (dd, *J* = 8.3, 7.0 Hz, 2H), 4.2 – 4.0 (m, 3H), 3.1 (dd, *J* = 14.2, 1.7 Hz, 1H), 2.5 (dd, *J* = 14.2, 5.0 Hz, 1H), 2.1 – 1.9 (m, 3H), 1.8 – 1.5 (m, 5H), 1.0 (t, *J* = 7.1 Hz, 3H).

^{13}C NMR (126 MHz, Chloroform-*d*) δ 195.3, 172.1, 134.4, 133.3, 129.7, 128.3, 100.2, 88.1, 76.5, 61.8, 41.2, 37.4, 32.4, 24.8, 24.1, 13.7.

HRMS (EI) m/z: $[M]^+$ Calcd. for $C_{18}H_{22}O_5^+$ 318.1467; Found: 318.1464.

IR (neat, cm^{-1}) 3251, 2913, 1742, 1677, 1351, 1277, 1077, 993, 746.

ethyl 2-benzoyl-4-hydroxy-1-oxaspiro[4.5]decane-2-carboxylate (12)

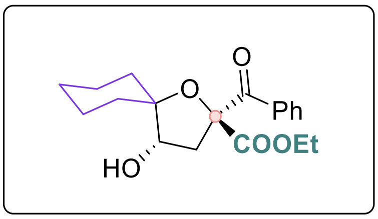

Following the **general procedure F** on 0.1 mmol scale, yellow oil, yield: 62% (20.6 mg), > 20: 1 dr. R_f = 0.3 (silica gel, hexane: EtOAc = 5:1, v/v), column chromatography (silica gel, hexane: EtOAc = 5: 1, v/v).

^{1}H NMR (500 MHz, Chloroform-*d*) δ 8.1 – 8.0 (m, 2H), 7.6 – 7.5 (m, 1H), 7.4 (m, 2H), 4.2 – 4.0 (m, 3H), 3.0 (dd, *J* = 14.4, 2.0 Hz, 1H), 2.6 (dd, *J* = 14.4, 5.7 Hz, 1H), 2.3 (s, 1H), 1.9 (m, 1H), 1.7 (m, 1H), 1.6 – 1.4 (m, 7H), 1.4 – 1.3 (m, 1H), 1.0 (t, *J* = 7.1 Hz, 3H).

^{13}C NMR (126 MHz, Chloroform-*d*) δ 195.7, 172.1, 134.4, 133.2, 129.8, 128.3, 90.8, 88.1, 76.0, 61.7, 40.6, 35.8, 31.2, 25.5, 23.2, 23.0, 13.8.

HRMS (EI) m/z: $[M]^+$ Calcd. for $C_{19}H_{24}O_5^+$ 332.1624; Found: 332.1622.

IR (neat, cm^{-1}) 3251, 2968, 1729, 1695, 1348, 1277, 1101, 1078, 771.

ethyl 2-benzoyl-4-hydroxy-1-oxaspiro[4.7]dodecane-2-carboxylate (13)

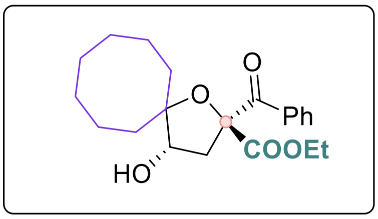

Following the **general procedure F** on 0.1 mmol scale, yellow oil, yield: 43% (15.5 mg), > 20: 1 dr. R_f = 0.3 (silica gel, hexane: EtOAc = 5:1, v/v), column chromatography (silica gel, hexane: EtOAc = 5: 1, v/v).

^{1}H NMR (500 MHz, Chloroform-*d*) δ 8.0 (m, 2H), 7.5 (m, 1H), 7.4 (m, 2H), 4.2 – 4.0 (m, 3H), 3.0 (dd, *J* = 14.4, 1.9 Hz, 1H), 2.6 (dd, *J* = 14.4, 5.8 Hz, 1H), 2.4 (s, 1H), 2.0 (m, 1H), 1.9 – 1.8 (m, 1H), 1.7 (m, 2H), 1.6 (m, 4H), 1.6 – 1.5 (m, 4H), 1.4 (m, 2H), 1.0 (t, *J* = 7.1 Hz, 3H).

^{13}C NMR (126 MHz, Chloroform-*d*) δ 196.0, 172.1, 134.4, 133.2, 129.7, 128.3, 94.7, 88.1, 76.5, 61.7, 41.1, 34.3, 29.3, 28.2, 28.1, 24.7, 22.4, 22.0, 13.7.

HRMS (EI) m/z: $[M]^+$ Calcd. for $C_{21}H_{28}O_5^+$ 360.1937; Found: 360.1931.

IR (neat, cm^{-1}) 3559, 2971, 1733, 1698, 1361, 1254, 1101, 1069, 788.

ethyl 2-benzoyl-4-hydroxy-1-oxaspiro[4.11]hexadecane-2-carboxylate (14)

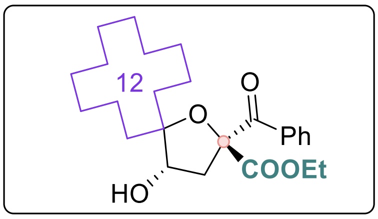

Following the **general procedure F** on 0.1 mmol scale, yellow oil, yield: 59% (24.5 mg), > 20: 1 dr. R_f = 0.4 (silica gel, hexane: EtOAc = 5:1, v/v), column chromatography (silica gel, hexane: EtOAc = 6: 1, v/v).

^{1}H NMR (500 MHz, Chloroform-*d*) δ 8.0 – 8.0 (m, 2H), 7.5 (d, *J* = 7.4 Hz, 1H), 7.4 (dd, *J* = 8.4, 7.2 Hz, 2H), 4.2 – 4.0 (m, 3H), 3.0 (dd, *J* = 14.4, 1.9 Hz, 1H), 2.6 (dd, *J* = 14.4, 5.5 Hz, 1H), 1.7 – 1.6 (m, 3H), 1.6 – 1.5 (m, 4H), 1.4 – 1.3 (m, 13H), 1.3 (d, *J* = 6.3 Hz, 2H), 1.0 (t, *J* = 7.1 Hz, 3H).

^{13}C NMR (126 MHz, Chloroform-*d*) δ 195.7, 172.1, 134.3, 133.3, 129.7, 128.4, 94.7, 88.2, 76.0, 61.7, 40.9, 32.5, 28.4, 26.6, 26.5, 26.1, 22.5, 22.2, 22.0, 19.6, 19.6, 13.7.

HRMS (EI) m/z: $[M]^+$ Calcd. for $C_{25}H_{36}O_5^+$ 416.2563; Found: 416.2561.

IR (neat, cm^{-1}) 3493, 2928, 1746, 1691, 1469, 1236, 1063, 691.

ethyl 2-benzoyl-4-hydroxy-1-oxaspiro[4.14]nonadecane-2-carboxylate (15)

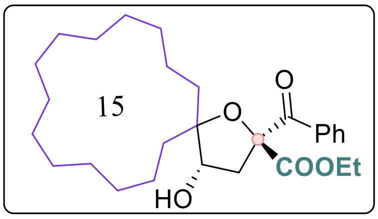

Following the **general procedure F** on 0.1 mmol scale, yellow oil, yield: 61% (28.0 mg), > 20: 1 dr. R_f = 0.4 (silica gel, hexane: EtOAc = 5:1, v/v), column chromatography (silica gel, hexane: EtOAc = 6: 1, v/v).

^{1}H NMR (400 MHz, Chloroform-*d*) δ 8.1 – 7.9 (m, 2H), 7.5 (m, 1H), 7.4 (m, 2H), 4.1 (m, 3H), 3.0 (dd, *J* = 14.4, 1.7 Hz, 1H), 2.6 (dd, *J* = 14.4, 5.6 Hz, 1H), 1.6 – 1.5 (m, 4H), 1.4 – 1.2 (m, 24H), 1.0 (t, *J* = 7.1 Hz, 3H).

^{13}C NMR (126 MHz, Chloroform-*d*) δ 195.9, 172.1, 134.4, 133.3, 129.7, 128.4, 94.5, 88.1, 76.2, 61.7, 41.0, 35.8, 31.3, 27.8, 27.8, 27.0, 27.0, 26.7, 26.7, 26.6, 26.3, 26.2, 22.2, 22.0, 13.7.

HRMS (EI) m/z: $[M]^+$ Calcd. for $C_{28}H_{42}O_5^+$ 458.3032; Found: 458.3028.

IR (neat, cm^{-1}) 3510, 2924, 1719, 1687, 1443, 1124, 1070, 692.

ethyl 2-benzoyl-4-hydroxy-6,10-dimethyl-1-oxaspiro[4.5]decane-2-carboxylate (16)

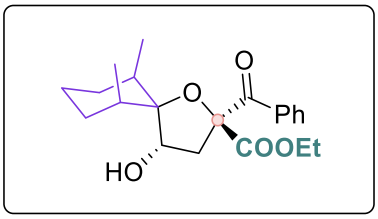

Following the **general procedure F** on 0.1 mmol scale, yellow oil, yield: 62% (22.3 mg), > 20: 1 dr. R_f = 0.3 (silica gel, hexane: EtOAc = 5:1, v/v), column chromatography (silica gel, hexane: EtOAc = 5: 1, v/v).

^{1}H NMR (500 MHz, Chloroform-*d*) δ 8.1 – 8.1 (m, 2H), 7.6 – 7.5 (m, 1H), 7.4 (m, 2H), 4.5 (m, 1H), 4.2 – 4.0 (m, 2H), 2.9 (dd, *J* = 14.2, 4.9 Hz, 1H), 2.7 (dd, *J* = 14.1, 7.1 Hz, 1H), 1.9 – 1.8 (m, 1H), 1.6 (m, 2H), 1.6 (m, 2H), 1.4 – 1.3 (m, 1H), 1.3 – 1.2 (m, 2H), 1.2 (d, *J* = 6.8 Hz, 3H), 1.0 (t, *J* = 7.1 Hz, 3H), 0.8 (d, *J* = 7.0 Hz, 3H).

^{13}C NMR (126 MHz, Chloroform-*d*) δ 196.3, 171.6, 134.6, 133.2, 130.0, 128.3, 93.2, 89.1, 74.9, 61.8, 41.3, 38.0, 34.6, 32.2, 31.5, 20.6, 17.5, 16.0, 13.8.

HRMS (EI) m/z: $[M]^+$ Calcd. for $C_{21}H_{28}O_5^+$ 360.1937; Found: 360.1931.

IR (neat, cm^{-1}) 3483, 2930, 1718, 1692, 1450, 1367, 1281, 1149, 1076, 713.

ethyl 2-benzoyl-8,8-difluoro-4-hydroxy-1-oxaspiro[4.5]decane-2-carboxylate (17)

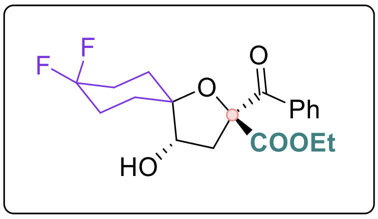

Following the **general procedure F** on 0.1 mmol scale, yellow oil, yield: 55% (20.2 mg), > 20: 1 dr. R_f = 0.3 (silica gel, hexane: EtOAc = 5:1, v/v), column chromatography (silica gel, hexane: EtOAc = 5: 1, v/v).

^{1}H NMR (400 MHz, Chloroform-*d*) δ 8.0 – 7.9 (m, 2H), 7.5 – 7.5 (m, 1H), 7.4 – 7.3 (m, 2H), 4.1 – 3.9 (m, 3H), 3.1 (dd, *J* = 14.5, 1.9 Hz, 1H), 2.5 (dd, *J* = 14.5, 5.5 Hz, 1H), 2.4 (m, 1H), 2.0 – 2.0 (m, 1H), 1.9 – 1.8 (m, 2H), 1.8 – 1.7 (m, 2H), 1.6 (m, 2H), 1.0 (t, *J* = 7.1 Hz, 3H).

^{13}C NMR (126 MHz, Chloroform-*d*) δ 194.7, 171.9, 134.1, 133.6, 129.7, 128.5, 125.4, 123.5, 88.6, 88.3, 76.2, 62.0, 40.5, 31.6, 31.6, 30.5, 30.4, 30.2, 30.2, 30.0, 29.8, 27.7, 27.7, 13.7.

^{19}F NMR (377 MHz, Chloroform-*d*) δ -92.7, -93.3, -103.0, -103.6.

HRMS (ESI) m/z: $[M+H]^+$ Calcd. for $C_{19}H_{22}F_2O_5^+$ 368.1435; Found: 368.1438.

IR (neat, cm^{-1}) 3501, 2938, 1726, 1691, 1449, 1379, 1271, 1101, 980, 691.

ethyl 5'-benzoyl-3'-hydroxy-4',5,5',6,8,9-hexahydro-3'H-spiro[benzo[7]annulene-7,2'-furan]-5'-carboxylate (18)

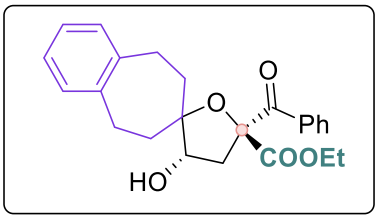

Following the **general procedure F** on 0.1 mmol scale, yellow oil, yield: 57% (22.5 mg), > 20: 1 dr. R_f = 0.3 (silica gel, hexane: EtOAc = 5:1, v/v), column chromatography (silica gel, hexane: EtOAc = 5: 1, v/v).

^{1}H NMR (500 MHz, Chloroform-*d*) δ 8.1 (m, 2H), 7.6 (m, 1H), 7.5 (m, 2H), 7.2 – 7.1 (m, 4H), 4.2 – 4.1 (m, 3H), 3.5 (s, 1H), 3.1 (dd, *J* = 14.4, 2.7 Hz, 1H), 3.0 (m, 1H), 2.7 – 2.6 (m, 2H), 2.5 (m, 1H), 2.3 (s, 1H), 2.0 (m, 1H), 1.9 (s, 1H), 1.6 (m, 2H), 1.1 (t, *J* = 7.1 Hz, 3H).

^{13}C NMR (126 MHz, Chloroform-*d*) δ 195.3, 172.1, 142.7, 142.6, 134.3, 133.4, 129.8, 128.8, 128.7, 128.4, 126.2, 126.1, 92.2, 88.1, 61.9, 40.5, 37.6, 32.9, 30.1, 30.0, 13.8.

HRMS (EI) m/z: $[M]^+$ Calcd. for $C_{24}H_{26}O_5^+$ 394.1780; Found: 394.1779.

IR (neat, cm^{-1}) 3586, 2927, 1732, 1693, 1451, 1288, 1271, 1064, 779, 693.

ethyl 10-benzoyl-12-hydroxy-1,4,9-trioxadispiro[4.2.4^8.2^5]tetradecane-10-carboxylate (19)

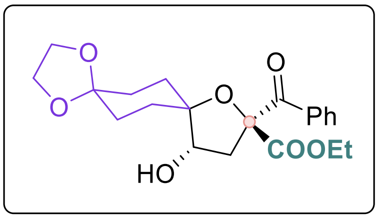

Following the **general procedure F** on 0.1 mmol scale, yellow oil, yield: 61% (23.8 mg), > 20: 1 dr. R_f = 0.3 (silica gel, hexane: EtOAc = 2:1, v/v), column chromatography (silica gel, hexane: EtOAc = 2: 1, v/v).

^{1}H NMR (500 MHz, Chloroform-*d*) δ 8.1 (m, 2H), 7.5 (m, 1H), 7.4 (m, 2H), 4.2 – 4.1 (m, 3H), 4.0 – 3.9 (m, 4H), 3.1 (dd, *J* = 14.4, 2.1 Hz, 1H), 2.6 (dd, *J* = 14.4, 5.6 Hz, 1H), 2.3 (m, 1H), 1.9 – 1.8 (m, 2H), 1.7 (m, 2H), 1.7 – 1.7 (m, 2H), 1.6 – 1.6 (m, 1H), 1.0 (t, *J* = 7.1 Hz, 3H).

^{13}C NMR (126 MHz, Chloroform-*d*) δ 195.1, 172.0, 134.3, 133.3, 129.8, 128.4, 108.7, 89.2, 88.2, 76.3, 64.3, 64.2, 61.8, 40.5, 32.8, 31.1, 30.9, 28.5, 13.8.

HRMS (EI) m/z: $[M]^+$ Calcd. for $C_{21}H_{26}O_7^+$ 390.1679; Found: 390.1683.

IR (neat, cm^{-1}) 3465, 2938, 1726, 1696, 1439, 1379, 1240, 1096, 732.

ethyl 2-benzoyl-4-hydroxy-11,11-dimethyl-1,9,13-trioxadispiro[4.2.5^8.2^5]pentadecane-2-carboxylate (20)

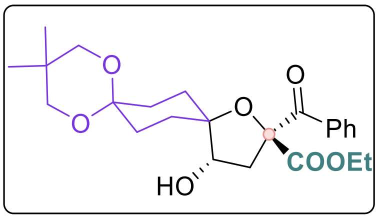

Following the **general procedure F** on 0.1 mmol scale, yellow oil, yield: 47% (19.9 mg), > 20: 1 dr. R_f = 0.3 (silica gel, hexane: EtOAc = 2:1, v/v), column chromatography (silica gel, hexane: EtOAc = 2: 1, v/v).

^{1}H NMR (400 MHz, Chloroform-*d*) δ 8.1 – 8.0 (m, 2H), 7.6 – 7.5 (m, 1H), 7.4 (m, 2H), 4.2 – 4.0 (m, 3H), 3.5 (s, 2H), 3.5 (s, 2H), 3.1 (dd, *J* = 14.4, 1.9 Hz, 1H), 2.6 (dd, *J* = 14.4, 5.5 Hz, 1H), 2.2 – 2.1 (m, 1H), 2.1 – 2.0 (m, 2H), 1.7 (s, 1H), 1.6 (s, 3H), 1.6 – 1.6 (m, 1H), 1.0 (t, *J* = 7.1 Hz, 3H), 1.0 (s, 3H), 0.9 (s, 3H).

^{13}C NMR (126 MHz, Chloroform-*d*) δ 195.3, 172.1, 134.3, 133.4, 129.8, 128.4, 97.4, 89.9, 88.2, 76.3, 70.1, 61.8, 40.5, 31.5, 30.2, 28.6, 28.3, 27.3, 22.8, 22.7, 13.8.

HRMS (EI) m/z: $[M]^+$ Calcd. for $C_{24}H_{32}O_7^+$ 423.2148; Found: 432.2142.

IR (neat, cm^{-1}) 3564, 2936, 1727, 1695, 1450, 1278, 1244, 1119, 895, 696.

ethyl 6-benzoyl-8-hydroxy-2,5-dioxaspiro[3.4]octane-6-carboxylate (21)

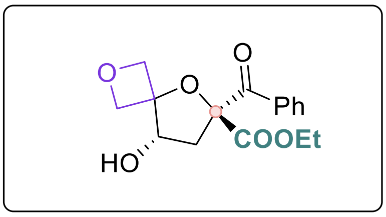

Following the **general procedure F** on 0.1 mmol scale, yellow oil, yield: 37% (11.3 mg), > 20: 1 dr. R_f = 0.3 (silica gel, hexane: EtOAc = 3:1, v/v), column chromatography (silica gel, hexane: EtOAc = 3: 1, v/v).

^{1}H NMR (400 MHz, Chloroform-*d*) δ 8.0 (m, 2H), 7.6 – 7.5 (m, 1H), 7.4 (m, 2H), 5.0 (d, *J* = 6.9 Hz, 1H), 4.8 (m, 1H), 4.7 (m, 1H), 4.6 (m, 1H), 4.4 (m, 1H), 4.2 – 4.1 (m, 2H), 3.1 (dd, *J* = 14.4, 1.7 Hz, 1H), 2.3 (dd, *J* = 14.4, 4.9 Hz, 1H), 1.1 (t, *J* = 7.1 Hz, 3H).

^{13}C NMR (126 MHz, Chloroform-*d*) δ 193.4, 171.3, 133.7, 129.8, 128.5, 88.9, 88.8, 81.3, 76.7, 74.4, 62.3, 39.7, 13.8.

HRMS (EI) m/z: $[M]^+$ Calcd. for $C_{16}H_{18}O_6^+$ 306.1103; Found: 306.1100.

IR (neat, cm^{-1}) 3522, 2921, 1728, 1685, 1455, 1269, 1232, 1107, 699.

ethyl 2-benzoyl-4-hydroxy-1,8-dioxaspiro[4.5]decane-2-carboxylate (22)

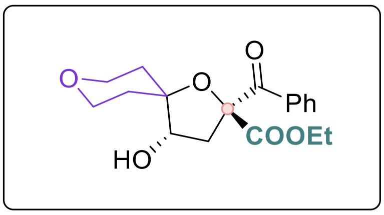

Following the **general procedure F** on 0.1 mmol scale, yellow oil, yield: 54% (18.0 mg), > 20: 1 dr. R_f = 0.3 (silica gel, hexane: EtOAc = 3:1, v/v), column chromatography (silica gel, hexane: EtOAc = 3: 1, v/v).

^{1}H NMR (500 MHz, Chloroform-*d*) δ 8.1 (m, 2H), 7.6 (m, 1H), 7.5 (mm, 2H), 4.2 – 4.0 (m, 4H), 3.8 (m, 1H), 3.7 (m, 1H), 3.7 (m, 1H), 3.1 (dd, *J* = 14.4, 2.2 Hz, 1H), 2.6 (dd, *J* = 14.4, 5.5 Hz, 1H), 1.8 – 1.7 (m, 3H), 1.6 (m, 1H), 1.0 (t, *J* = 7.1 Hz, 3H).

^{13}C NMR (126 MHz, Chloroform-*d*) δ 194.9, 171.9, 134.2, 133.5, 129.8, 128.4, 88.2, 87.6, 76.1, 64.9, 64.4, 61.9, 40.1, 35.5, 31.5, 13.8.

HRMS (EI) m/z: $[M]^+$ Calcd. for $C_{18}H_{22}O_6^+$ 334.1416; Found: 334.1407.

IR (neat, cm^{-1}) 3548, 2976, 1728, 1693, 1455, 1275, 1259, 1108, 698.

ethyl 2-benzoyl-4-hydroxy-1,7-dioxaspiro[4.5]decane-2-carboxylate (23)

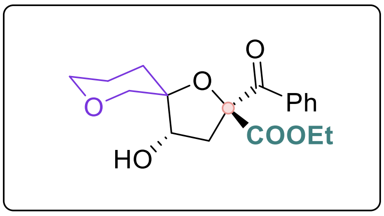

Following the **general procedure F** on 0.1 mmol scale, yellow oil, yield: 62% (20.7 mg), 2.5: 1 dr. R_f = 0.3 (silica gel, hexane: EtOAc = 3:1, v/v), column chromatography (silica gel, hexane: EtOAc = 3: 1, v/v).

^{1}H NMR (400 MHz, Chloroform-*d*) δ 8.0 (m, 2H), 7.5 (m, 1H), 7.4 (m, 2H), 4.2 (s, 1H), 4.1 – 4.0 (m, 2H), 3.7 (m, 1H), 3.6 (m, 1H), 3.5 (m, 1H), 3.3 (d, *J* = 11.7 Hz, 1H), 3.0 (dd, *J* = 14.3, 2.1 Hz, 1H), 2.5 (dd, *J* = 14.4, 5.6 Hz, 1H), 2.5 – 2.4 (m, 1H), 1.9 – 1.8 (m, 2H), 1.7 (m, 1H), 1.7 – 1.6 (m, 1H), 1.0 (t, *J* = 7.1 Hz, 3H).

^{13}C NMR (126 MHz, Chloroform-*d*) δ 194.9, 171.6, 134.2, 133.3, 129.8, 128.4, 88.9, 86.5, 75.2, 70.2, 67.8, 62.0, 40.6, 33.5, 24.0, 13.8.

HRMS (EI) m/z: $[M]^+$ Calcd. for $C_{18}H_{22}O_6^+$ 334.1416; Found: 334.1415.

IR (neat, cm^{-1}) 3452, 2923, 1725, 1694, 1446, 1265, 1246, 1091, 918, 693.

ethyl 2-benzoyl-4-hydroxy-7,7-dimethyl-1,8-dioxaspiro[4.5]decane-2-carboxylate (24)

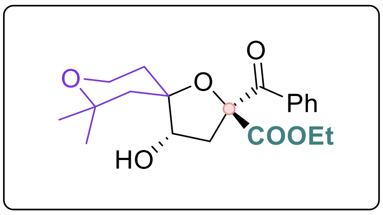

Following the **general procedure F** on 0.1 mmol scale, yellow oil, yield: 61% (22.1 mg), 1: 1 dr. R_f = 0.3 (silica gel, hexane: EtOAc = 3:1, v/v), column chromatography (silica gel, hexane: EtOAc = 3: 1, v/v).

^{1}H NMR (500 MHz, Chloroform-*d*) δ 8.1 – 8.0 (m, 2H), 7.5 (m, 1H), 7.4 (m, 2H), 4.2 – 4.0 (m, 2.6H), 4.0 – 3.9 (m, 1H), 3.8 – 3.7 (m, 0.5H), 3.1 (dd, *J* = 14.5, 1.6 Hz, 0.5H), 3.0 (dd, *J* = 14.0, 4.3 Hz, 0.5H), 2.5 (dd, *J* = 14.0, 5.9 Hz, 0.6H), 2.5 (dd, *J* = 14.5, 5.1 Hz, 0.6H), 1.8 (m, 0.5H), 1.7 (m, 1H), 1.6 (m, 1H), 1.5 – 1.6 (m, 0.6H), 1.5 (s, 1.5H), 1.4 (m, 0.7H), 1.3 – 1.2 (m, 0.3H)1.2 (d, *J* = 9.4 Hz, 3H), 1.1 (s, 1.5H), 1.0 (td, *J* = 7.1, 5.9 Hz, 3H).

^{13}C NMR (126 MHz, Chloroform-*d*) δ 195.2, 194.7, 172.2, 171.7, 134.1, 134.1, 133.4, 133.4, 129.8, 129.6, 128.4, 128.3, 88.7, 88.4, 88.2, 87.4, 77.6, 71.6, 71.6, 61.9, 58.2, 58.0, 44.4, 40.4, 39.5, 39.4, 34.5, 32.0, 31.5, 30.3, 24.6, 24.2, 13.8, 13.8.

HRMS (EI) m/z: $[M]^+$ Calcd. for $C_{20}H_{26}O_6^+$ 362.1729; Found: 362.1728.

IR (neat, cm^{-1}) 3406, 2970, 1728, 1690, 1449, 1364, 1268, 1059, 692.

8-(tert-butyl) 2-ethyl 2-benzoyl-4-hydroxy-1-oxa-8-azaspiro[4.5]decane-2,8-dicarboxylate (25)

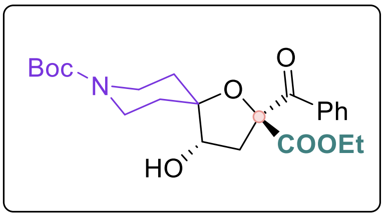

Following the **general procedure F** on 0.1 mmol scale, yellow oil, yield: 42% (18.2 mg), > 20: 1 dr. R_f = 0.3 (silica gel, hexane: EtOAc = 2:1, v/v), column chromatography (silica gel, hexane: EtOAc = 2: 1, v/v).

^{1}H NMR (400 MHz, Chloroform-*d*) δ 8.1 – 8.0 (m, 2H), 7.6 – 7.5 (m, 1H), 7.4 (m, 2H), 4.2 – 4.0 (m, 3H), 3.9 (m, 1H), 3.8 (m, 1H), 3.5 (m, 1H), 3.1 (dd, *J* = 14.3, 2.1 Hz, 1H), 3.1 – 3.0 (m, 1H), 2.5 (dd, *J* = 14.4, 5.5 Hz, 1H), 1.7 (m, 1H), 1.6 (m, 2H), 1.5 (m, 1H), 1.4 (s, 9H), 1.0 (t, *J* = 7.1 Hz, 3H).

^{13}C NMR (126 MHz, Chloroform-*d*) δ 194.9, 171.9, 154.9, 134.1, 133.5, 129.8, 128.4, 88.4, 88.2, 79.5, 76.2, 62.0, 40.3, 34.6, 30.6, 28.4, 13.8.

HRMS (ESI) m/z: $[M+Na]^+$ Calcd. for $C_{23}H_{31}NO_7^+$ 456.1999; Found: 456.1995.

IR (neat, cm^{-1}) 3447, 2974, 1733, 1694, 1423, 1367, 1244, 1160, 1074, 692.

ethyl 2-benzoyl-4-hydroxy-1-oxa-8-thiaspiro[4.5]decane-2-carboxylate 8,8-dioxide (26)

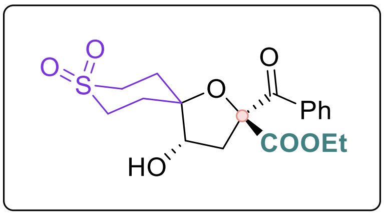

Following the **general procedure F** on 0.1 mmol scale, yellow oil, yield: 60% (22.9 mg), > 20: 1 dr. R_f = 0.3 (silica gel, hexane: EtOAc = 5:1, v/v), column chromatography (silica gel, hexane: EtOAc = 5: 1, v/v).

^{1}H NMR (400 MHz, Chloroform-*d*) δ 8.0 – 8.0 (m, 2H), 7.6 – 7.6 (m, 1H), 7.5 – 7.4 (m, 2H), 4.2 – 4.1 (m, 3H), 3.9 (m, 1H), 3.2 – 3.1 (m, 2H), 3.0 – 3.0 (m, 1H), 2.9 – 2.8 (m, 1H), 2.6 (dd, *J* = 14.6, 5.5 Hz, 1H), 2.3 – 2.1 (m, 4H), 1.0 (t, *J* = 7.1 Hz, 3H).

^{13}C NMR (126 MHz, Chloroform-*d*) δ 193.7, 171.6, 133.9, 133.8, 129.6, 128.7, 88.7, 86.4, 62.4, 48.0, 47.4, 40.4, 33.0, 29.5, 13.8.

HRMS (EI) m/z: $[M]^+$ Calcd. for $C_{18}H_{22}O_7S^+$ 382.1086; Found: 382.1083.

IR (neat, cm^{-1}) 3459, 2971, 1728, 1691, 1434, 1366, 1229, 1054, 699.

ethyl 2-benzoyl-4-hydroxy-5,5-dimethyltetrahydrofuran-2-carboxylate (27)

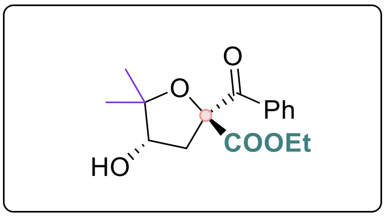

Following the **general procedure F** on 0.1 mmol scale, yellow oil, yield: 69% (20.1 mg), > 20: 1 dr. R_f = 0.3 (silica gel, hexane: EtOAc = 5:1, v/v), column chromatography (silica gel, hexane: EtOAc = 5: 1, v/v).

^{1}H NMR (500 MHz, Chloroform-*d*) δ 8.0 (m, 2H), 7.6 – 7.5 (m, 1H), 7.4 (m, 2H), 4.2 – 4.0 (m, 3H), 3.0 (dd, *J* = 14.3, 2.5 Hz, 1H), 2.6 (dd, *J* = 14.3, 5.6 Hz, 1H), 2.4 (s, 1H), 1.4 (s, 3H), 1.2 (s, 3H), 1.0 (t, *J* = 7.1 Hz, 3H).

^{13}C NMR (126 MHz, Chloroform-*d*) δ 195.4, 172.0, 134.3, 133.3, 129.7, 128.4, 89.2, 88.2, 77.3, 61.9, 40.7, 27.0, 22.3, 13.7.

HRMS (EI) m/z: $[M]^+$ Calcd. for $C_{16}H_{20}O_5^+$ 292.1311; Found: 292.1304.

IR (neat, cm^{-1}) 3501, 2976, 1727, 1692, 1450, 1336, 1248, 1063, 690.

ethyl 2-benzoyl-5,5-diethyl-4-hydroxytetrahydrofuran-2-carboxylate (28)

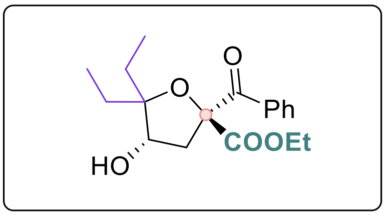

Following the **general procedure F** on 0.1 mmol scale, yellow oil, yield: 52% (16.6 mg), > 20: 1 dr. R_f = 0.3 (silica gel, hexane: EtOAc = 5:1, v/v), column chromatography (silica gel, hexane: EtOAc = 5: 1, v/v).

^{1}H NMR (500 MHz, Chloroform-*d*) δ 8.1 – 8.0 (m, 2H), 7.6 – 7.5 (m, 1H), 7.4 (m, 2H), 4.1 (m, 2H), 4.1 (m, 1H), 3.0 (dd, *J* = 14.4, 2.1 Hz, 1H), 2.6 (dd, *J* = 14.4, 5.9 Hz, 1H), 2.4 (s, 1H), 1.7 – 1.5 (m, 4H), 1.0 (t, *J* = 7.1 Hz, 6H), 0.8 (t, *J* = 7.5 Hz, 3H).

^{13}C NMR (126 MHz, Chloroform-*d*) δ 195.8, 172.0, 134.4, 133.3, 129.7, 128.4, 94.4, 88.1, 75.6, 61.7, 41.0, 28.1, 24.1, 13.7, 8.5, 8.1.

HRMS (EI) m/z: $[M]^+$ Calcd. for $C_{18}H_{24}O_5^+$ 320.1624; Found: 320.1623.

IR (neat, cm^{-1}) 3227, 2955, 1727, 1691, 1464, 1451, 1368, 1250, 1066, 690.

ethyl 2-benzoyl-4-hydroxy-5,5-diisobutyltetrahydrofuran-2-carboxylate (29)

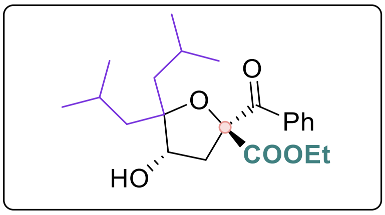

Following the **general procedure F** on 0.1 mmol scale, yellow oil, yield: 50% (18.8 mg), > 20: 1 dr. R_f = 0.3 (silica gel, hexane: EtOAc = 5:1, v/v), column chromatography (silica gel, hexane: EtOAc = 5: 1, v/v).

^{1}H NMR (500 MHz, Chloroform-*d*) δ 8.0 – 8.0 (m, 2H), 7.6 – 7.5 (m, 1H), 7.4 (m, 2H), 4.2 – 4.1 (m, 1H), 4.1 – 4.0 (m, 2H), 3.0 (dd, *J* = 14.3, 2.7 Hz, 1H), 2.6 (dd, *J* = 14.2, 5.7 Hz, 1H), 2.3 (s, 1H), 1.8 (m, 1H), 1.7 (m, 2H), 1.6 (m, 1H), 1.5 (m, 2H), 1.0 (m, 3H), 1.0 (m, 6H), 0.8 (m, 3H), 0.8 (m, 3H).

^{13}C NMR (126 MHz, Chloroform-*d*) δ 195.7, 172.0, 134.3, 133.3, 129.7, 128.3, 94.1, 87.8, 76.4, 61.7, 44.9, 40.6, 40.4, 24.6, 24.6, 24.3, 24.2, 23.9, 13.7.

HRMS (EI) m/z: $[M]^+$ Calcd. for $C_{22}H_{32}O_5^+$ 376.2250; Found: 376.2250.

IR (neat, cm^{-1}) 3427, 2949, 1728, 1693, 1459, 1367, 1255, 1059, 693.

ethyl 2-benzoyl-5-ethyl-4-hydroxy-5-methyltetrahydrofuran-2-carboxylate (30)

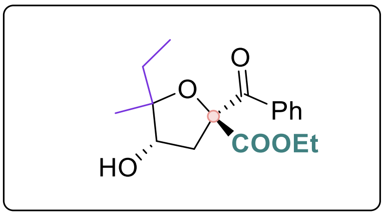

Following the **general procedure F** on 0.1 mmol scale, yellow oil, yield: 56% (17.1 mg), 1.5: 1 dr. R_f = 0.3 (silica gel, hexane: EtOAc = 5:1, v/v), column chromatography (silica gel, hexane: EtOAc = 5: 1, v/v).

^{1}H NMR (400 MHz, Chloroform-*d*) δ 8.0 – 7.9 (m, 2H), 7.5 – 7.4 (m, 1H), 7.3 (m, 2H), 4.1 – 4.0 (m, 3H), 3.0 (m, 1H), 2.6 – 2.5 (m, 1H), 1.6 – 1.5 (m, 2H), 1.2 (s, 1.8H), 1.1 (s, 1.2H), 1.0 – 0.9 (m, 4H), 0.8 (t, *J* = 7.5 Hz, 2H).

^{13}C NMR (126 MHz, Chloroform-*d*) δ 195.6, 172.1, 171.9, 134.4, 133.3, 129.7, 129.7, 128.4, 128.3, 92.0, 91.5, 88.3, 87.9, 76.8, 75.9, 61.8, 61.8, 40.8, 40.8, 32.5, 28.2, 23.4, 19.1, 13.7, 8.9, 8.6.

HRMS (EI) m/z: $[M]^+$ Calcd. for $C_{17}H_{22}O_5^+$ 306.1467; Found: 306.1461.

IR (neat, cm^{-1}) 3479, 2951, 1732, 1692, 1445, 1267, 1245, 1097, 691.

ethyl 2-benzoyl-5-cyclohexyl-4-hydroxy-5-methyltetrahydrofuran-2-carboxylate (31)

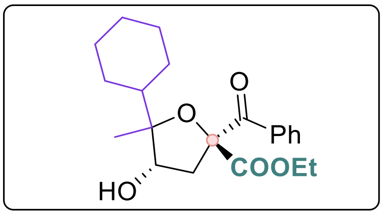

Following the **general procedure F** on 0.1 mmol scale, yellow oil, yield: 62% (22.3 mg), 2: 1 dr. R_f = 0.3 (silica gel, hexane: EtOAc = 5:1, v/v), column chromatography (silica gel, hexane: EtOAc = 5: 1, v/v).

^{1}H NMR (400 MHz, Chloroform-*d*) δ 8.0 – 7.9 (m, 2H), 7.5 (m, 1H), 7.4 (m, 2H), 4.2 (m, 1H), 4.1 – 3.9 (m, 2H), 2.9 (dd, *J* = 14.1, 3.9 Hz, 1H), 2.5 (dd, *J* = 14.1, 6.5 Hz, 1H), 2.1 (m, 1H), 1.8 – 1.7 (m, 3H), 1.7 – 1.6 (m, 1H), 1.5 – 1.4 (m, 1H), 1.3 – 1.2 (m, 3H), 1.1 – 1.0 (m, 2H), 1.0 – 0.9 (m, 6H).

^{13}C NMR (126 MHz, Chloroform-*d*) δ 195.7, 171.8, 134.4, 133.3, 129.7, 128.4, 93.0, 87.5, 74.9, 61.7, 46.9, 40.9, 28.7, 27.3, 26.7, 26.6, 26.6, 16.7, 13.8.

HRMS (EI) m/z: $[M]^+$ Calcd. for $C_{21}H_{28}O_5^+$ 360.1937; Found: 360.1932.

IR (neat, cm^{-1}) 3529, 2948, 1732, 1694, 1443, 1266, 1243, 1088, 6941.

ethyl (1R,2R,5S)-5'-benzoyl-3'-hydroxydihydro-3'H-7,8-dioxaspiro[bicyclo[3.2.1]octane-2,2'-furan]-5'-carboxylat (32)

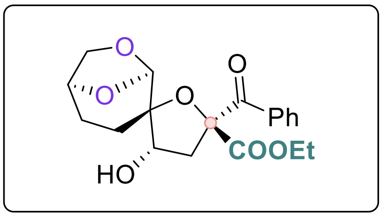

Following the **general procedure F** on 0.1 mmol scale, yellow oil, yield: 57% (20.6 mg), > 20: 1 dr. R_f = 0.3 (silica gel, hexane: EtOAc = 3:1, v/v), column chromatography (silica gel, hexane: EtOAc = 3: 1, v/v).

^{1}H NMR (400 MHz, Chloroform-*d*) δ 8.1 (m, 2H), 7.5 (m, 1H), 7.4 (m, 2H), 5.2 (s, 1H), 4.5 (s, 1H), 4.3 (m, 1H), 4.1 (m, 2H), 4.0 (m, 1H), 3.8 – 3.7 (m, 1H), 3.1 (d, *J* = 14.4 Hz, 1H), 2.5 (dd, *J* = 14.4, 5.1 Hz, 1H), 2.4 (s, 1H), 2.1 (m, 1H), 1.9 – 1.8 (m, 1H), 1.7 – 1.6 (m, 2H), 1.1 (t, *J* = 7.1 Hz, 3H).

^{13}C NMR (126 MHz, Chloroform-*d*) δ 195.6, 171.1, 134.3, 133.3, 129.9, 128.3, 102.1, 90.2, 89.6, 75.5, 73.2, 68.5, 62.1, 40.9, 28.2, 27.6, 13.8.

HRMS (EI) m/z: $[M]^+$ Calcd. for $C_{19}H_{22}O_7^+$ 362.1366; Found: 362.1351.

IR (neat, cm^{-1}) 3448, 2922, 1729, 1693, 1446, 1268, 1223, 1068, 697.

ethyl (2R,3a'R,4'S,7'S,7a'R)-5-benzoyl-3-hydroxydecahydro-3H-spiro[furan-2,5'-[4,7]methanoindene]-5-carboxylate (33)

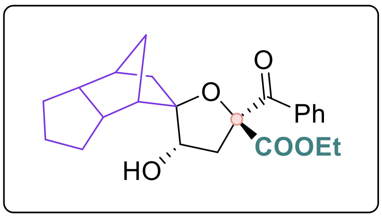

Following the **general procedure F** on 0.1 mmol scale, yellow oil, yield: 53% (20.4 mg), 1.2: 1 dr. R_f = 0.3 (silica gel, hexane: EtOAc = 5:1, v/v), column chromatography (silica gel, hexane: EtOAc = 5: 1, v/v).

^{1}H NMR (500 MHz, Chloroform-*d*) δ 8.1 – 8.0 (m, 2H), 7.6 (m, 1H), 7.5 (m, 2H), 4.2 – 4.0 (m, 2H), 4.0 (m, 1H), 3.0 (m, 1H), 2.7 (m, 0.4H), 2.6 (m, 0.6H), 2.4 (m, 0.6H), 2.3 (s, 0.6H), 2.2 – 2.1 (m, 1H), 2.0 (s, 1H), 1.9 (m, 2H), 1.8 (s, 2H), 1.7 – 1.6 (m, 1H), 1.4 (d, *J* = 4.9 Hz, 1H), 1.3 – 1.3 (m, 1H), 1.2 – 1.2 (m, 1H), 1.1 – 1.1 (m, 1H), 1.0 (dt, *J* = 16.2, 7.1 Hz, 3H), 0.9 (m, 2H).

^{13}C NMR (126 MHz, Chloroform-*d*) δ 195.6, 195.2, 172.1, 171.9, 134.5, 134.3, 133.3, 133.3, 129.8, 129.6, 128.4, 128.4, 98.1, 97.7, 88.3, 87.9, 77.1, 76.6, 61.7, 61.7, 49.8, 47.7, 47.3, 45.1, 42.5, 41.6, 41.2, 40.9, 40.1, 39.8, 39.3, 36.7, 32.8, 32.5, 31.8, 31.7, 31.4, 27.4, 27.2, 13.8, 13.7.

HRMS (EI) m/z: $[M]^+$ Calcd. for $C_{23}H_{28}O_5^+$ 384.1937; Found: 384.1931.

IR (neat, cm^{-1}) 3489, 2938, 1729, 1691, 1449, 1272, 1223, 1068, 692.

ethyl (1R,3S,5r,7r)-5'-benzoyl-3'-hydroxydihydro-3'H-spiro[adamantane-2,2'-furan]-5'-carboxylate (34)

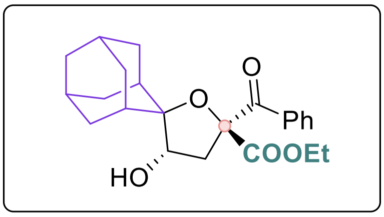

Following the **general procedure F** on 0.1 mmol scale, yellow oil, yield: 44% (16.9 mg), > 20: 1 dr. R_f = 0.3 (silica gel, hexane: EtOAc = 5:1, v/v), column chromatography (silica gel, hexane: EtOAc = 5: 1, v/v).

^{1}H NMR (500 MHz, Chloroform-*d*) δ 8.2 – 8.1 (m, 2H), 7.6 – 7.5 (m, 1H), 7.5 (m, 2H), 4.5 (d, *J* = 5.1 Hz, 1H), 4.2 – 4.0 (m, 2H), 3.1 (d, *J* = 14.5 Hz, 1H), 2.7 – 2.6 (m, 1H), 2.6 (dd, *J* = 14.5, 5.1 Hz, 1H), 2.1 – 2.1 (m, 1H), 2.0 (m, 1H), 1.9 – 1.8 (m, 4H), 1.8 – 1.8 (m, 2H), 1.7 (m, 3H), 1.7 (m, 1H), 1.6 (m, 1H), 1.0 (t, *J* = 7.1 Hz, 3H).

^{13}C NMR (126 MHz, Chloroform-*d*) δ 195.8, 172.4, 134.5, 133.3, 129.8, 128.3, 95.6, 88.3, 73.4, 61.7, 40.8, 37.6, 36.7, 35.6, 35.1, 34.1, 32.9, 32.4, 27.2, 26.9, 13.8.

HRMS (EI) m/z: $[M]^+$ Calcd. for $C_{23}H_{28}O_5^+$ 384.1937; Found: 384.1932.

IR (neat, cm^{-1}) 3346, 2938, 1715, 1694, 1417, 1284, 1105, 1021, 710.

ethyl 5'-benzoyl-3'-hydroxydihydro-3'H-spiro[bicyclo[2.2.1]heptane-2,2'-furan]-5'-carboxylate (35)

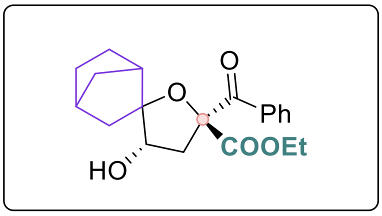

Following the **general procedure F** on 0.1 mmol scale, yellow oil, yield: 45% (15.5 mg), > 1.2: 1 dr. R_f = 0.3 (silica gel, hexane: EtOAc = 5:1, v/v), column chromatography (silica gel, hexane: EtOAc = 5: 1, v/v).

^{1}H NMR (400 MHz, Chloroform-*d*) δ 8.0 (m, 2H), 7.5 (m, 1H), 7.4 (m, 2H), 4.1 – 4.0 (m, 2H), 3.9 (d, *J* = 4.7 Hz, 1H), 3.0 – 2.9 (m, 1H), 2.6 (m, 0.65H), 2.4 (m, 0.5H), 2.3 (m, 0.7H), 2.2 – 2.3 (m, 0.5H), 2.1 – 2.2 (m, 0.7H), 2.1 (m, 0.8H), 1.9 – 1.9 (m, 0.7H), 1.4 – 1.5 (m, 0.7H), 1.6 (m, 0.7H), 1.4 – 1.5 (m, 1.7H), 1.3 (m, 4H), 1.2 – 1.1 (m, 1H), 1.2 – 1.1 (m, 0.7H), 0.9 (t, *J* = 8.5, 3H).

^{13}C NMR (126 MHz, Chloroform-*d*) δ 195.7, 195.0, 172.1, 171.8, 134.4, 134.3, 133.3, 129.8, 129.6, 128.4, 128.4, 98.4, 98.0, 88.3, 87.8, 76.5, 61.8, 61.7, 45.5, 42.8, 41.2, 40.8, 39.8, 38.9, 37.8, 37.3, 37.0, 36.4, 29.1, 28.7, 22.7, 21.9, 13.8.

HRMS (EI) m/z: $[M]^+$ Calcd. for $C_{20}H_{24}O_5^+$ 344.1624; Found: 344.1619.

IR (neat, cm^{-1}) 3480, 2943, 1732, 1694, 1447, 1268, 1247, 1070, 987, 694.

ethyl (6R,9S)-2-benzoyl-4-hydroxy-6-isopropyl-9-methyl-1-oxaspiro[4.5]decane-2-carboxylate (36)

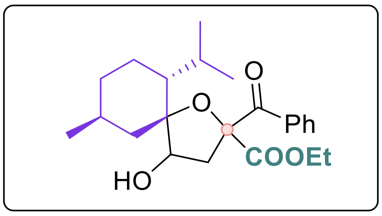

Following the **general procedure F** on 0.1 mmol scale, yellow oil, yield: 43% (16.7 mg), > 20: 1 dr. R_f = 0.3 (silica gel, hexane: EtOAc = 5:1, v/v), column chromatography (silica gel, hexane: EtOAc = 5: 1, v/v).

^{1}H NMR (500 MHz, Chloroform-*d*) δ 8.2 – 8.1 (m, 2H), 7.5 (m, 1H), 7.5 – 7.4 (m, 2H), 4.3 – 4.2 (m, 1H), 4.2 – 4.0 (m, 2H), 3.0 (dd, J = 13.7, 8.7 Hz, 1H), 2.7 (dd, J = 13.8, 7.6 Hz, 1H), 2.2 – 2.1 (m, 1H), 2.1 (m, 1H), 2.0 (m, 1H), 1.8 (m, 1H), 1.5 (m, 1H), 1.4 – 1.3 (m, 2H), 1.3 – 1.3 (m, 1H), 1.1 (m, 1H), 1.0 (m, 3H), 1.0 (m, 3H), 0.8 (m, 3H), 0.4 (m, 3H).

^{13}C NMR (126 MHz, Chloroform-*d*) δ 193.1, 172.8, 134.5, 133.3, 130.0, 128.4, 91.0, 87.9, 80.2, 61.7, 48.5, 45.9, 41.4, 34.6, 29.2, 26.9, 23.4, 22.5, 22.4, 17.9, 13.8.

HRMS (EI) m/z: $[M]^+$ Calcd. for $C_{23}H_{32}O_5^+$ 388.2250; Found: 388.2244.

IR (neat, cm^{-1}) 3479, 2926, 1727, 1687, 1450, 1260, 1089, 1070, 937, 670.

ethyl 4-hydroxy-2-(4-iodobenzoyl)-1-oxaspiro[4.5]decane-2-carboxylate (37)

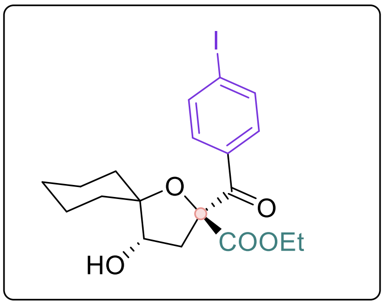

Following the **general procedure F** on 0.1 mmol scale, yellow oil, yield: 57% (26.1 mg), > 20: 1 dr. R_f = 0.3 (silica gel, hexane: EtOAc = 5:1, v/v), column chromatography (silica gel, hexane: EtOAc = 5: 1, v/v).

^{1}H NMR (500 MHz, Chloroform-*d*) δ 7.8 (s, 4H), 4.2 – 4.0 (m, 3H), 3.0 (dd, J = 14.3, 2.1 Hz, 1H), 2.6 (dd, J = 14.3, 5.5 Hz, 1H), 1.8 (m, 1H), 1.7 – 1.7 (m, 1H), 1.5 – 1.4 (m, 7H), 1.4 – 1.3 (m, 1H), 1.1 (t, J = 7.1 Hz, 3H).

^{13}C NMR (126 MHz, Chloroform-*d*) δ 194.9, 171.8, 137.6, 133.7, 131.3, 101.5, 90.7, 88.2, 75.8, 61.9, 40.6, 35.7, 31.1, 25.5, 23.1, 22.9, 13.9.

HRMS (EI) m/z: $[M]^+$ Calcd. for $C_{19}H_{23}O_5^+$ 458.0590; Found: 458.0594.

IR (neat, cm^{-1}) 3504, 2937, 1726, 1693, 1450, 1254, 1089, 1068, 937, 688.

ethyl 4-hydroxy-2-(4-(trifluoromethoxy)benzoyl)-1-oxaspiro[4.5]decane-2-carboxylate (38)

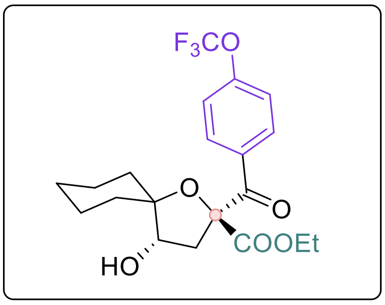

Following the **general procedure F** on 0.1 mmol scale, yellow oil, yield: 63% (26.2 mg), > 20: 1 dr. R_f = 0.3 (silica gel, hexane: EtOAc = 5:1, v/v), column chromatography (silica gel, hexane: EtOAc = 5: 1, v/v).

^{1}H NMR (400 MHz, Chloroform-*d*) δ 8.2 – 8.1 (m, 2H), 7.3 – 7.2 (m, 2H), 4.2 – 4.0 (m, 3H), 3.1 (dd, *J* = 14.3, 2.1 Hz, 1H), 2.6 (dd, *J* = 14.3, 5.5 Hz, 1H), 2.1 (s, 1H), 1.9 (m, 1H), 1.8 – 1.7 (m, 1H), 1.5 (m, 7H), 1.3 (m, 1H), 1.1 (t, *J* = 7.1 Hz, 1H).

^{13}C NMR (126 MHz, Chloroform-*d*) δ 194.1, 171.8, 152.6, 132.6, 132.0, 119.9, 119.2, 90.7, 88.3, 61.9, 40.6, 35.7, 31.1, 25.5, 23.1, 22.9, 13.8.

^{19}F NMR (377 MHz, Chloroform-*d*) δ -57.6.

HRMS (EI) m/z: $[M]^+$ Calcd. for $C_{20}H_{23}F_3O_6^+$ 416.1447; Found: 416.1444.

IR (neat, cm^{-1}) 3217, 2935, 1758, 1689, 1449, 1260, 1072, 906, 697.

ethyl 4-hydroxy-2-(4-(methylsulfonyl)benzoyl)-1-oxaspiro[4.5]decane-2-carboxylate (39)

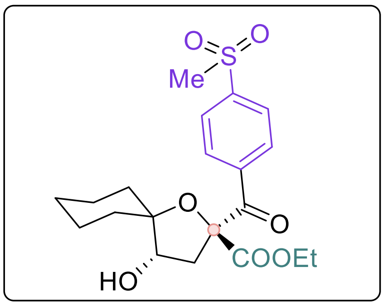

Following the **general procedure F** on 0.1 mmol scale, yellow ssolid, m.p. 130-132 °C, yield: 59% (24.2 mg), > 20: 1 dr. R_f = 0.3 (silica gel, hexane: EtOAc = 5:1, v/v), column chromatography (silica gel, hexane: EtOAc = 5: 1, v/v).

^{1}H NMR (500 MHz, Chloroform-*d*) δ 8.2 (m, 2H), 8.0 (m, 2H), 4.2 – 4.1 (m, 3H), 3.1 (s, 3H), 3.1 – 3.0 (m, 1H), 2.6 (dd, *J* = 14.3, 5.3 Hz, 1H), 2.0 (s, 1H), 1.8 (m, 1H), 1.7 – 1.7 (m, 1H), 1.5 – 1.4 (m, 7H), 1.3 – 1.3 (m, 1H), 1.1 (t, *J* = 7.1 Hz, 3H).

^{13}C NMR (126 MHz, Chloroform-*d*) δ 195.0, 171.4, 143.8, 139.0, 130.8, 127.2, 90.8, 88.6, 75.6, 62.2, 44.3, 40.9, 35.6, 30.9, 25.4, 23.0, 22.9, 13.9.

HRMS (EI) m/z: $[M]^+$ Calcd. for $C_{20}H_{26}O_7S^+$ 410.1399; Found: 410.1396.

IR (neat, cm^{-1}) 3495, 2928, 1728, 1699, 1449, 1317, 1289, 1013, 780.

ethyl 4-hydroxy-2-(4-(methoxycarbonyl)benzoyl)-5,5-dimethyltetrahydrofuran-2-carboxylate (40)

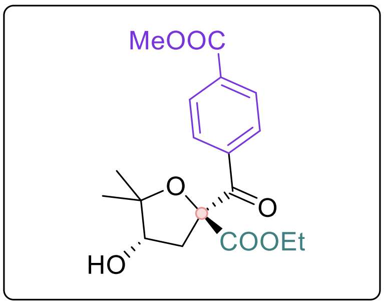

Following the **general procedure F** on 0.1 mmol scale, yellow oil, yield: 62% (21.7 mg), > 20: 1 dr. R_f = 0.3 (silica gel, hexane: EtOAc = 5:1, v/v), column chromatography (silica gel, hexane: EtOAc = 5: 1, v/v).

^{1}H NMR (400 MHz, Chloroform-*d*) δ 8.0 (s, 4H), 4.1 – 4.0 (m, 2H), 4.0 (m, 1H), 3.9 (s, 3H), 3.0 (dd, *J* = 14.2, 2.7 Hz, 1H), 2.6 (dd, *J* = 14.3, 5.5 Hz, 1H), 1.3 (s, 3H), 1.1 (s, 3H), 1.0 (t, *J* = 7.1 Hz, 3H).

^{13}C NMR (126 MHz, Chloroform-*d*) δ 195.2, 171.6, 166.3, 137.9, 133.8, 129.6, 129.4, 89.0, 88.4, 77.1, 62.1, 52.5, 40.8, 27.0, 22.1, 13.8.

HRMS (EI) m/z: $[M]^+$ Calcd. for $C_{18}H_{22}O_7^+$ 350.1366; Found: 350.1366.

IR (neat, cm^{-1}) 3498, 2972, 1733, 1690, 1447, 1256, 1071, 983, 694.

ethyl 2-(4-cyanobenzoyl)-4-hydroxy-5,5-dimethyltetrahydrofuran-2-carboxylate (41)

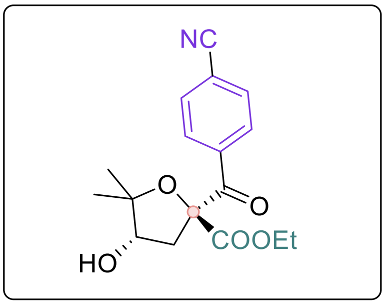

Following the **general procedure F** on 0.1 mmol scale, yellow oil, yield: 56% (17.8 mg), > 20: 1 dr. R_f = 0.3 (silica gel, hexane: EtOAc = 4:1, v/v), column chromatography (silica gel, hexane: EtOAc = 4: 1, v/v).

^{1}H NMR (400 MHz, Chloroform-*d*) δ 8.2 – 8.1 (m, 2H), 7.7 – 7.7 (m, 2H), 4.2 – 4.1 (m, 2H), 4.1 (m, 1H), 3.1 (dd, *J* = 14.3, 2.6 Hz, 1H), 2.6 (dd, *J* = 14.2, 5.3 Hz, 1H), 1.3 (s, 3H), 1.2 (s, 3H), 1.1 (t, *J* = 7.1 Hz, 3H).

^{13}C NMR (126 MHz, Chloroform-*d*) δ 194.6, 171.3, 137.9, 132.0, 130.2, 118.0, 116.1, 89.1, 88.7, 76.9, 62.3, 41.0, 26.9, 22.0, 13.8.

HRMS (EI) m/z: $[M]^+$ Calcd. for $C_{17}H_{19}NO_5^+$ 317.1263; Found: 317.1259.

IR (neat, cm^{-1}) 3512, 2975, 1731, 1702, 1458, 1274, 1241, 1071, 857, 542.

ethyl 4-hydroxy-5,5-dimethyl-2-(4-nitrobenzoyl)tetrahydrofuran-2-carboxylate (42)

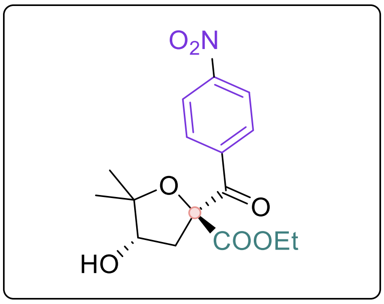

Following the **general procedure F** on 0.1 mmol scale, yellow oil, yield: 54% (18.2 mg), > 20: 1 dr. R_f = 0.3 (silica gel, hexane: EtOAc = 4:1, v/v), column chromatography (silica gel, hexane: EtOAc = 4: 1, v/v).

^{1}H NMR (400 MHz, Chloroform-*d*) δ 8.2 (m, 2H), 8.2 (m, 2H), 4.1 (m, 2H), 4.1 (mm, 1H), 3.0 (dd, *J* = 14.2, 2.6 Hz, 1H), 2.7 (dd, *J* = 14.2, 5.3 Hz, 1H), 1.3 (s, 3H), 1.1 (s, 3H), 1.1 (t, *J* = 7.1 Hz, 3H).

^{13}C NMR (126 MHz, Chloroform-*d*) δ 194.7, 171.2, 150.1, 139.5, 130.9, 123.3, 89.1, 88.8, 76.9, 62.3, 41.1, 26.9, 22.0, 13.9.

HRMS (EI) m/z: $[M]^+$ Calcd. for $C_{16}H_{19}NO_7^+$ 337.1162; Found: 337.1161.

IR (neat, cm^{-1}) 3558, 2977, 1724, 1703, 1523, 1347, 1266, 1204, 1105, 1063, 852, 719.

ethyl 4-hydroxy-2-(4-iodobenzoyl)-5,5-dimethyltetrahydrofuran-2-carboxylate (43)

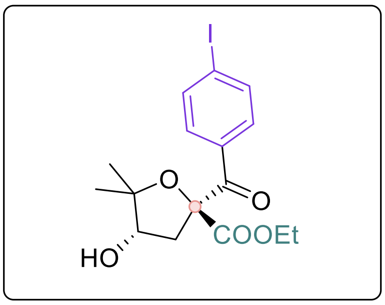

Following the **general procedure F** on 0.1 mmol scale, white solid, m.p. 154-156 ºC, yield: 63% (26.3 mg), > 20: 1 dr. R_f = 0.3 (silica gel, hexane: EtOAc = 5:1, v/v), column chromatography (silica gel, hexane: EtOAc = 5: 1, v/v).

^{1}H NMR (400 MHz, Chloroform-*d*) δ 7.8 – 7.7 (m, 4H), 4.1 (m, 2H), 4.0 (m, 1H), 3.0 (dd, *J* = 14.3, 2.5 Hz, 1H), 2.6 (dd, *J* = 14.3, 5.5 Hz, 1H), 1.3 (s, 3H), 1.2 (s, 3H), 1.1 (t, *J* = 7.1 Hz, 3H).

^{13}C NMR (126 MHz, Chloroform-*d*) δ 194.7, 171.7, 137.7, 133.6, 131.2, 101.5, 89.1, 88.4, 77.1, 62.0, 40.7, 27.0, 22.2, 13.8.

HRMS (EI) m/z: $[M]^+$ Calcd. for $C_{16}H_{19}IO_5^+$ 418.0277; Found: 418.0272.

IR (neat, cm^{-1}) 3214, 2976, 1724, 1690, 1578, 1271, 1247, 1062, 1006.

ethyl 2-(4-(chloromethyl)benzoyl)-4-hydroxy-5,5-dimethyltetrahydrofuran-2-carboxylate (44)

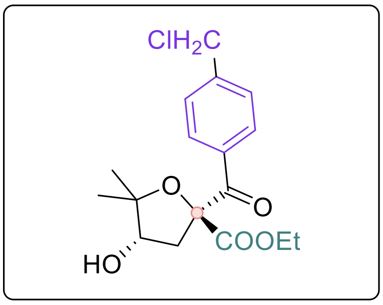

Following the **general procedure F** on 0.1 mmol scale, yellow oil, yield: 47% (16.0 mg), > 20: 1 dr. R_f = 0.3 (silica gel, hexane: EtOAc = 5:1, v/v), column chromatography (silica gel, hexane: EtOAc = 5: 1, v/v).

^{1}H NMR (400 MHz, Chloroform-*d*) δ 8.0 (mm, 2H), 7.4 (m, 2H), 4.5 (s, 2H), 4.1 – 4.0 (m, 2H), 4.0 (m, 1H), 3.0 (dd, *J* = 14.3, 2.6 Hz, 1H), 2.5 (dd, *J* = 14.3, 5.6 Hz, 1H), 1.3 (s, 3H), 1.1 (s, 3H), 1.0 (t, *J* = 7.1 Hz, 3H).

^{13}C NMR (126 MHz, Chloroform-*d*) δ 194.7, 171.9, 142.5, 134.1, 130.3, 128.4, 89.2, 88.3, 77.2, 62.0, 45.3, 40.7, 27.0, 22.2, 13.8.

HRMS (EI) m/z: $[M]^+$ Calcd. for $C_{17}H_{21}ClO_5^+$ 340.1078; Found: 340.1073.

IR (neat, cm^{-1}) 3622, 2983, 1731, 1691, 1273, 1265, 1109, 620.

ethyl 4-hydroxy-5,5-dimethyl-2-(4-(trifluoromethoxy)benzoyl)tetrahydrofuran-2-carboxylate (45)

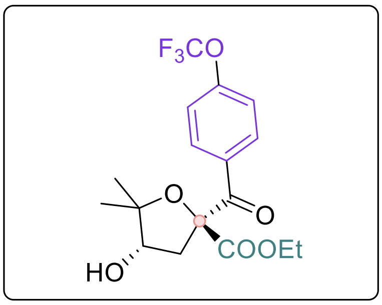

Following the **general procedure F** on 0.1 mmol scale, yellow oil, yield: 59% (22.2 mg), > 20: 1 dr. R_f = 0.3 (silica gel, hexane: EtOAc = 5:1, v/v), column chromatography (silica gel, hexane: EtOAc = 5: 1, v/v).

^{1}H NMR (400 MHz, Chloroform-*d*) δ 8.1 (m, 2H), 7.2 – 7.2 (m, 2H), 4.2 – 4.1 (m, 2H), 4.0 (m, 1H), 3.1 (dd, *J* = 14.3, 2.7 Hz, 1H), 2.6 (dd, *J* = 14.3, 5.6 Hz, 1H), 1.3 (s, 3H), 1.2 (s, 3H), 1.1 (t, *J* = 7.1 Hz, 3H).

^{13}C NMR (126 MHz, Chloroform-*d*) δ 193.9, 171.7, 152.6, 132.5, 132.0, 120.3 (d, *J* = 258.8 Hz), 120.0, 89.0, 88.4, 77.0, 62.0, 40.7, 26.9, 22.1, 13.7.

^{19}F NMR (377 MHz, Chloroform-*d*) δ -57.6.

HRMS (EI) m/z: $[M]^+$ Calcd. for $C_{17}H_{19}F_3O_6^+$ 376.1134; Found: 376.1132.

IR (neat, cm^{-1}) 3562, 2973, 1725, 1694, 1279, 1255, 1101, 690.

ethyl 2-(4-ethoxybenzoyl)-4-hydroxy-5,5-dimethyltetrahydrofuran-2-carboxylate (46)

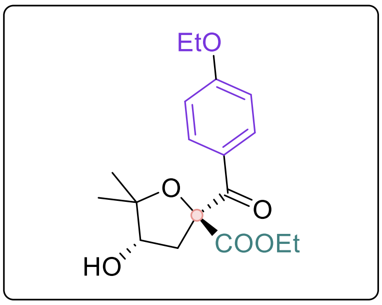

Following the **general procedure F** on 0.1 mmol scale, yellow oil, yield: 48% (16.1 mg), > 20: 1 dr. R_f = 0.3 (silica gel, hexane: EtOAc = 5:1, v/v), column chromatography (silica gel, hexane: EtOAc = 5: 1, v/v).

^{1}H NMR (400 MHz, Chloroform-*d*) δ 8.0 (m, 2H), 6.9 (m, 2H), 4.2 – 4.0 (m, 4H), 4.0 (m, 1H), 3.1 (dd, *J* = 14.3, 2.5 Hz, 1H), 2.6 (dd, *J* = 14.3, 5.6 Hz, 1H), 1.4 (t, *J* = 7.0 Hz, 3H), 1.3 (s, 3H), 1.2 (s, 3H), 1.0 (t, *J* = 7.1 Hz, 3H).

^{13}C NMR (126 MHz, Chloroform-*d*) δ 193.7, 172.3, 163.2, 132.3, 132.2, 126.8, 114.1, 114.1, 89.1, 88.2, 77.3, 63.8, 61.8, 40.6, 27.0, 22.3, 14.6, 13.8.

HRMS (EI) m/z: $[M]^+$ Calcd. for $C_{18}H_{24}O_6^+$ 336.1573; Found: 336.1568.

IR (neat, cm^{-1}) 3504, 2983, 1725, 1679, 1508, 1253, 1169, 846.

ethyl 4-hydroxy-2-(2-(methoxycarbonyl)benzoyl)-5,5-dimethyltetrahydrofuran-2-carboxylate (47)

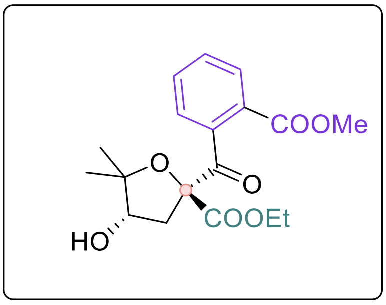

Following the **general procedure F** on 0.1 mmol scale, yellow oil, yield: 49% (18.2 mg), > 20: 1 dr. R_f = 0.3 (silica gel, hexane: EtOAc = 4:1, v/v), column chromatography (silica gel, hexane: EtOAc = 4: 1, v/v).

^{1}H NMR (400 MHz, Chloroform-*d*) δ 7.9 (m, 1H), 7.6 – 7.5 (m, 2H), 7.5 – 7.4 (m, 1H), 4.2 (m, 2H), 3.9 (s, 1H), 3.9 (s, 3H), 3.0 (dd, *J* = 14.9, 5.8 Hz, 1H), 2.9 (dd, *J* = 14.9, 1.7 Hz, 1H), 1.2 (t, *J* = 7.1 Hz, 3H), 1.2 (s, 3H), 1.1 (s, 3H).

^{13}C NMR (126 MHz, Chloroform-*d*) δ 202.3, 171.2, 167.8, 140.2, 132.0, 129.9, 129.6, 129.4, 127.6, 90.5, 88.9, 77.4, 62.2, 53.0, 43.1, 26.3, 22.0, 14.0.

HRMS (ESI) m/z: $[M+Na]^+$ Calcd. for $C_{18}H_{22}O_7^+$ 373.1264; Found: 373.1260.

IR (neat, cm^{-1}) 3524, 2964, 1729, 1698, 1456, 1341, 1279, 1255, 1159, 605.

ethyl 2-(3,5-dimethylbenzoyl)-4-hydroxy-5,5-dimethyltetrahydrofuran-2-carboxylate (48)

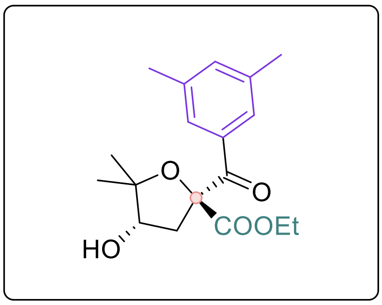

Following the **general procedure F** on 0.1 mmol scale, yellow oil, yield: 69% (22.1 mg), > 20: 1 dr. R_f = 0.3 (silica gel, hexane: EtOAc = 5:1, v/v), column chromatography (silica gel, hexane: EtOAc = 5: 1, v/v).

^{1}H NMR (500 MHz, Chloroform-*d*) δ 7.6 (m, 2H), 7.2 (m, 1H), 4.2 – 4.1 (m, 2H), 4.0 (m, 1H), 3.0 (dd, *J* = 14.3, 2.3 Hz, 1H), 2.6 (dd, *J* = 14.3, 5.6 Hz, 1H), 2.3 (s, 6H), 1.4 (s, 3H), 1.2 (s, 3H), 1.0 (t, *J* = 7.1 Hz, 3H).

^{13}C NMR (126 MHz, Chloroform-*d*) δ 196.0, 172.2, 138.0, 135.1, 134.4, 127.3, 89.3, 88.1, 77.4, 61.8, 40.9, 27.0, 22.3, 21.3, 13.7.

HRMS (EI) m/z: $[M]^+$ Calcd. for $C_{18}H_{24}O_5^+$ 320.1624; Found: 320.1621.

IR (neat, cm^{-1}) 3380, 2925, 1716, 1699, 1279, 1424, 1311, 1121, 616.

ethyl 2-(5-chloro-2-fluorobenzoyl)-4-hydroxy-5,5-dimethyltetrahydrofuran-2-carboxylate (49)

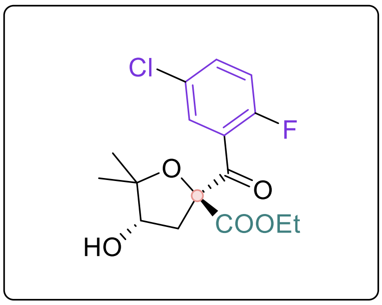

Following the **general procedure F** on 0.1 mmol scale, yellow oil, yield: 51% (17.5 mg), > 20: 1 dr. R_f = 0.3 (silica gel, hexane: EtOAc = 5:1, v/v), column chromatography (silica gel, hexane: EtOAc = 5: 1, v/v).

^{1}H NMR (400 MHz, Chloroform-*d*) δ 7.7 (m, 1H), 7.4 (m, 1H), 7.0 (m, 1H), 4.3 – 4.2 (m, 2H), 4.0 (mm, 1H), 2.9 (dd, *J* = 14.0, 2.0 Hz, 1H), 2.7 (dd, *J* = 14.0, 4.8 Hz, 1H), 1.9 (s, 1H) 1.3 (s, 3H), 1.2 (t, *J* = 7.1 Hz, 3H), 1.1 (s, 3H).

^{13}C NMR (126 MHz, Chloroform-*d*) δ 195.8, 170.2, 158.9 (d, *J* = 253.9 Hz), 133.4 (d, *J* = 8.9 Hz), 130.7 (d, *J* = 3.5 Hz), 129.4 (d, *J* = 3.3 Hz), 126.0 (d, *J* = 16.0 Hz), 117.5 (d, *J* = 24.7 Hz), 89.9, 88.4, 76.9, 62.2, 42.4, 26.9, 21.7, 13.9.

^{19}F NMR (377 MHz, Chloroform-*d*) δ -112.0.

HRMS (EI) m/z: $[M]^+$ Calcd. for $C_{16}H_{18}ClFO_5^+$ 344.0827; Found: 344.0832.

IR (neat, cm^{-1}) 3445, 2984, 1718, 1685, 1298, 1255, 1108, 760.

ethyl 2-(3,4-dichlorobenzoyl)-4-hydroxy-5,5-dimethyltetrahydrofuran-2-carboxylate (50)

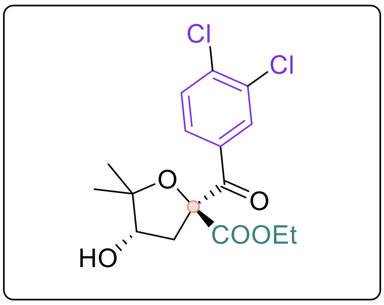

Following the **general procedure F** on 0.1 mmol scale, yellow oil, yield: 62% (22.3 mg), > 20: 1 dr. R_f = 0.3 (silica gel, hexane: EtOAc = 5:1, v/v), column chromatography (silica gel, hexane: EtOAc = 5: 1, v/v).

^{1}H NMR (400 MHz, Chloroform-*d*) δ 8.1 (m, 1H), 7.9 (m, 1H), 7.5 (m, 1H), 4.2 – 4.1 (m, 2H), 4.0 (m, 1H), 3.0 (dd, *J* = 14.3, 2.7 Hz, 1H), 2.6 (dd, *J* = 14.2, 5.4 Hz, 1H), 1.3 (s, 3H), 1.2 (s, 3H), 1.1 (t, *J* = 7.1 Hz, 3H).

^{13}C NMR (126 MHz, Chloroform-*d*) δ 193.4, 171.5, 137.8, 134.0, 132.9, 131.7, 130.3, 129.1, 89.0, 88.6, 76.9, 62.2, 40.8, 26.9, 22.1, 13.9.

HRMS (EI) m/z: $[M]^+$ Calcd. for $C_{16}H_{18}Cl_2O_5^+$ 360.0531; Found: 360.0527.

IR (neat, cm^{-1}) 3503, 2983, 1729, 1697, 1464, 1378, 1273, 1064, 1033, 797, 823.

ethyl 2-(3,4-difluorobenzoyl)-4-hydroxy-5,5-dimethyltetrahydrofuran-2-carboxylate (51)

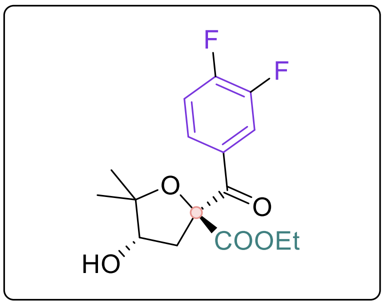

Following the **general procedure F** on 0.1 mmol scale, yellow oil, yield: 63% (20.7 mg), > 20: 1 dr. R_f = 0.3 (silica gel, hexane: EtOAc = 5:1, v/v), column chromatography (silica gel, hexane: EtOAc = 5: 1, v/v).

^{1}H NMR (400 MHz, Chloroform-*d*) δ 7.9 (m, 1H), 7.9 – 7.8 (m, 1H), 7.2 (m, 1H), 4.1 (m, 2H), 4.0 (m, 1H), 3.1 (dd, *J* = 14.3, 2.7 Hz, 1H), 2.6 (dd, *J* = 14.3, 5.5 Hz, 1H), 1.3 (s, 3H), 1.2 (s, 3H), 1.1 (t, *J* = 7.1 Hz, 3H).

^{13}C NMR (126 MHz, Chloroform-*d*) δ 192.9, 171.6, 153.6 (dd, *J* = 257.5, 12.9 Hz), 150.0 (dd, *J* = 250.1, 12.8 Hz), 131.3, 127.2 (dd, *J* = 7.4, 3.6 Hz), 119.3 (d, *J* = 18.7 Hz), 117.2 (d, *J* = 17.8 Hz), 89.0, 88.5, 77.0, 62.1, 40.7, 26.9, 22.1, 13.8.

^{19}F NMR (377 MHz, Chloroform-*d*) δ -129.3 (d, *J* = 20.9 Hz), -136.3 (d, *J* = 21.1 Hz).

HRMS (EI) m/z: $[M]^+$ Calcd. for $C_{16}H_{18}F_2O_5^+$ 328.1122; Found: 328.1120.

IR (neat, cm^{-1}) 3399, 2950, 1738, 1697, 1594, 1436, 1320, 1120, 987, 862.

ethyl 2-(3-chloro-4-methylbenzoyl)-4-hydroxy-5,5-dimethyltetrahydrofuran-2-carboxylate (52)

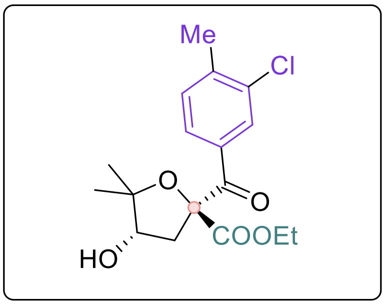

Following the **general procedure F** on 0.1 mmol scale, yellow oil, yield: 67% (22.8 mg), > 20: 1 dr. R_f = 0.3 (silica gel, hexane: EtOAc = 5:1, v/v), column chromatography (silica gel, hexane: EtOAc = 5: 1, v/v).

^{1}H NMR (400 MHz, Chloroform-*d*) δ 7.9 (m, 1H), 7.8 (m, 1H), 7.4 (m, 1H), 4.2 – 4.1 (m, 2H), 4.0 (m, 1H), 3.0 (dd, *J* = 14.3, 2.6 Hz, 1H), 2.6 (dd, *J* = 14.3, 5.6 Hz, 1H), 2.4 (s, 3H), 1.3 (s, 3H), 1.2 (s, 3H), 1.1 (t, *J* = 7.1 Hz, 3H).

^{13}C NMR (126 MHz, Chloroform-*d*) δ 194.5, 171.9, 140.0, 136.4, 132.7, 132.1, 129.1, 128.7, 89.1, 88.3, 77.2, 62.0, 40.7, 27.0, 22.2, 20.1, 13.8.

HRMS (EI) m/z: $[M]^+$ Calcd. for $C_{17}H_{21}ClO_5^+$ 340.1078; Found: 340.1070.

IR (neat, cm^{-1}) 3532, 2983, 1719, 1688, 1288, 1239, 1108, 695.

ethyl 2-(5-chloro-2-(trifluoromethyl)benzoyl)-4-hydroxy-5,5-dimethyltetrahydrofuran-2-carboxylate (53)

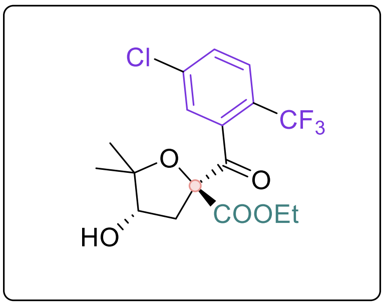

Following the **general procedure F** on 0.1 mmol scale, yellow oil, yield: 46% (18.1 mg), > 20: 1 dr. R_f = 0.3 (silica gel, hexane: EtOAc = 5:1, v/v), column chromatography (silica gel, hexane: EtOAc = 5: 1, v/v).

^{1}H NMR (400 MHz, Chloroform-*d*) δ 8.0 (m, 1H), 7.6 (m, 1H), 7.5 (m, 1H), 4.3 – 4.2 (m, 2H), 4.0 (m, 1H), 2.9 – 2.8 (m, 2H), 1.3 – 1.2 (m, 6H), 1.1 (s, 3H).

^{13}C NMR (126 MHz, Chloroform-*d*) δ 199.1, 170.1, 137.1, 135.3, 130.7, 128.3 (q, *J* = 33.8 Hz), 127.8 (q, *J* = 3.7 Hz), 127.5 (q, *J* = 3.8 Hz), 123.5 (q, *J* = 271.3 Hz), 120.2, 90.2, 88.9, 76.7, 62.5, 43.4, 26.6, 21.5, 13.9.

^{19}F NMR (377 MHz, Chloroform-*d*) δ -62.7.

HRMS (EI) m/z: $[M]^+$ Calcd. for $C_{17}H_{18}ClF_3O_5^+$ 394.0795; Found: 394.0787.

IR (neat, cm^{-1}) 3453, 2973, 1727, 1700, 1333, 1263, 1126, 1079, 830.

ethyl 2-(6-bromo-2-naphthoyl)-4-hydroxy-5,5-dimethyltetrahydrofuran-2-carboxylate (54)

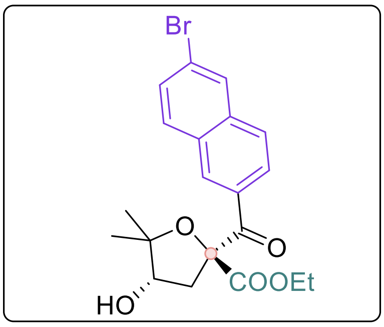

Following the **general procedure F** on 0.1 mmol scale, yellow solid, m.p. 129-131 ºC, yield: 46% (19.3 mg), > 20: 1 dr. R_f = 0.3 (silica gel, hexane: EtOAc = 5:1, v/v), column chromatography (silica gel, hexane: EtOAc = 5: 1, v/v).

^{1}H NMR (500 MHz, Chloroform-*d*) δ 8.6 – 8.6 (m, 1H), 8.1 (m, 1H), 8.0 (mm, 1H), 7.9 (m, 1H), 7.8 (m, 1H), 7.6 (m, 1H), 4.2 – 4.0 (m, 3H), 3.1 (dd, *J* = 14.3, 2.4 Hz, 1H), 2.7 (dd, *J* = 14.3, 5.6 Hz, 1H), 2.3 (s, 1H), 1.4 (s, 3H), 1.2 (s, 3H), 1.0 (t, *J* = 7.1 Hz, 3H).

^{13}C NMR (126 MHz, Chloroform-*d*) δ 195.1, 172.0, 136.5, 132.0, 131.7, 131.5, 130.8, 130.2, 129.9, 127.2, 126.3, 123.1, 89.3, 88.5, 77.3, 62.0, 40.9, 27.0, 22.3, 13.8.

HRMS (EI) m/z: $[M]^+$ Calcd. for $C_{20}H_{21}BrO_5^+$ 420.0572; Found: 420.0565.

IR (neat, cm^{-1}) 3538, 2969, 1728, 1697, 1284, 1287, 1109, 730.

ethyl 2-(5-chlorothiophene-2-carbonyl)-4-hydroxy-1-oxaspiro[4.5]decane-2-carboxylate (55)

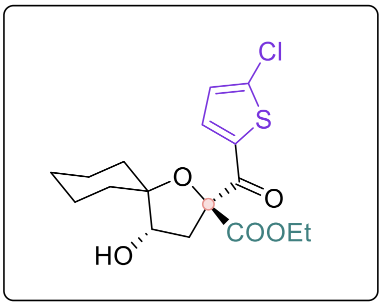

Following the **general procedure F** on 0.1 mmol scale, yellow oil, yield: 53% (19.7 mg), > 20: 1 dr. R_f = 0.3 (silica gel, hexane: EtOAc = 5:1, v/v), column chromatography (silica gel, hexane: EtOAc = 5: 1, v/v).

^{1}H NMR (500 MHz, Chloroform-*d*) δ 7.9 (d, *J* = 4.1 Hz, 1H), 7.0 (d, *J* = 4.2 Hz, 1H), 4.2 (m, 2H), 4.1 (m, 1H), 3.0 (dt, *J* = 14.3, 1.7 Hz, 1H), 2.7 (dd, *J* = 14.3, 5.1 Hz, 1H), 1.9 – 1.8 (m, 2H), 1.8 – 1.7 (m, 3H), 1.6 (m, 1H), 1.6 – 1.5 (m, 2H), 1.5 – 1.4 (m, 1H), 1.4 – 1.3 (m, 1H), 1.2 (t, *J* = 7.1 Hz, 3H).

^{13}C NMR (126 MHz, Chloroform-*d*) δ 188.2, 170.9, 140.6, 137.7, 135.2, 127.3, 90.6, 89.0, 75.5, 62.1, 40.9, 35.5, 30.9, 25.5, 23.1, 23.0, 13.9.

HRMS (EI) m/z: $[M]^+$ Calcd. for $C_{17}H_{21}ClO_5S^+$ 372.0798; Found: 372.0796.

IR (neat, cm^{-1}) 3506, 2957, 1727, 1668, 1368, 1293, 1114, 1012.

ethyl 4-hydroxy-5,5-dimethyl-2-(thiophene-2-carbonyl)tetrahydrofuran-2-carboxylate (56)

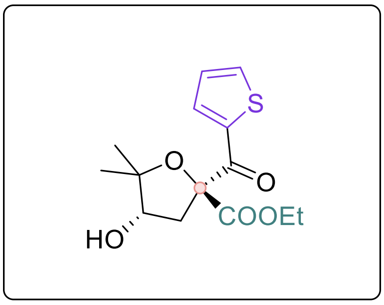

Following the **general procedure F** on 0.1 mmol scale, yellow oil, yield: 61% (18.2 mg), > 20: 1 dr. R_f = 0.3 (silica gel, hexane: EtOAc = 5:1, v/v), column chromatography (silica gel, hexane: EtOAc = 5: 1, v/v).

^{1}H NMR (400 MHz, Chloroform-*d*) δ 8.0 – 8.0 (m, 1H), 7.7 (m, 1H), 7.1 – 7.0 (m, 1H), 4.2 (m, 2H), 4.0 (m, 1H), 3.0 (dd, *J* = 14.3, 2.6 Hz, 1H), 2.7 (dd, *J* = 14.3, 5.4 Hz, 1H), 1.3 (s, 4H), 1.3 (s, 3H), 1.1 (t, *J* = 7.1 Hz, 3H).

^{13}C NMR (126 MHz, Chloroform-*d*) δ 188.8, 171.3, 140.0, 135.3, 134.8, 128.1, 88.9, 88.7, 77.1, 62.1, 40.8, 26.9, 22.1, 13.9.

HRMS (EI) m/z: $[M]^+$ Calcd. for $C_{14}H_{18}O_5S^+$ 298.0875; Found: 298.0873.

IR (neat, cm^{-1}) 3524, 2978, 1735, 1689, 1371, 1284, 1018.

ethyl 2-(5-bromothiophene-2-carbonyl)-4-hydroxy-5,5-dimethyltetrahydrofuran-2-carboxylate (57)

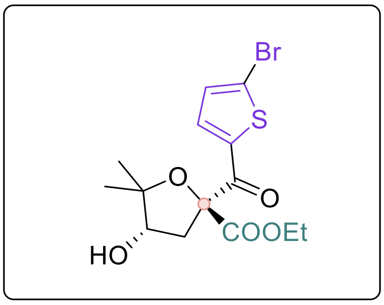

Following the **general procedure F** on 0.1 mmol scale, yellow oil, yield: 48% (18.0 mg), > 20: 1 dr. R_f = 0.3 (silica gel, hexane: EtOAc = 5:1, v/v), column chromatography (silica gel, hexane: EtOAc = 5: 1, v/v).

^{1}H NMR (400 MHz, Chloroform-*d*) δ 7.8 (d, *J* = 4.1 Hz, 1H), 7.1 (d, *J* = 4.2 Hz, 1H), 4.2 (q, *J* = 7.1 Hz, 2H), 4.0 (m, 1H), 2.9 (dd, *J* = 14.2, 2.5 Hz, 1H), 2.7 (dd, *J* = 14.2, 5.2 Hz, 1H), 1.3 (s, 3H), 1.3 (s, 3H), 1.2 (t, *J* = 7.1 Hz, 3H).

^{13}C NMR (126 MHz, Chloroform-*d*) δ 188.0, 170.7, 140.7, 135.7, 131.0, 124.1, 89.1, 88.8, 76.9, 62.2, 41.1, 26.8, 21.9, 13.9.

HRMS (EI) m/z: $[M]^+$ Calcd. for $C_{14}H_{17}BrO_5S^+$ 375.9980; Found: 375.9977.

IR (neat, cm^{-1}) 3504, 2980, 1727, 1662, 1403, 1249, 1061.

ethyl 2-(5-(ethoxycarbonyl)thiophene-2-carbonyl)-4-hydroxy-5,5-dimethyltetrahydrofuran-2-carboxylate (58)

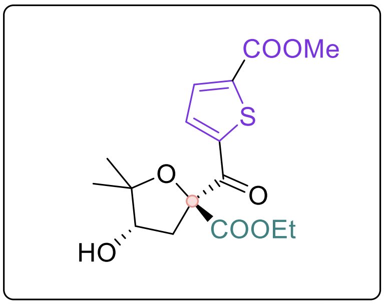

Following the **general procedure F** on 0.1 mmol scale, yellow oil, yield: 56% (20.7 mg), > 20: 1 dr. R_f = 0.3 (silica gel, hexane: EtOAc = 5:1, v/v), column chromatography (silica gel, hexane: EtOAc = 5: 1, v/v).

^{1}H NMR (400 MHz, Chloroform-*d*) δ 7.9 (d, *J* = 4.1 Hz, 1H), 7.7 (d, *J* = 4.1 Hz, 1H), 4.2 (m, 2H), 4.1 (m, 1H), 3.9 (s, 3H), 3.0 (dd, *J* = 14.2, 2.6 Hz, 1H), 2.7 (dd, *J* = 14.2, 5.2 Hz, 1H), 1.3 (s, 3H), 1.3 (s, 3H), 1.1 (t, *J* = 7.1 Hz, 3H).

^{13}C NMR (126 MHz, Chloroform-*d*) δ 189.3, 170.6, 162.3, 144.0, 140.2, 134.6, 133.0, 89.2, 88.9, 76.8, 62.3, 52.6, 41.0, 26.8, 21.9, 13.9.

HRMS (EI) m/z: $[M]^+$ Calcd. for $C_{17}H_{22}O_7S^+$ 370.1086; Found: 370.1076.

IR (neat, cm^{-1}) 3523, 2976, 1722, 1682, 1254, 1101, 753.

ethyl 2-(benzo[b]thiophene-2-carbonyl)-4-hydroxy-5,5-dimethyltetrahydrofuran-2-carboxylate (59)

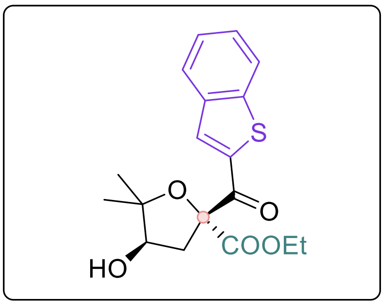

Following the **general procedure F** on 0.1 mmol scale, yellow oil, yield: 58% (20.2 mg), > 20: 1 dr. R_f = 0.3 (silica gel, hexane: EtOAc = 5:1, v/v), column chromatography (silica gel, hexane: EtOAc = 5: 1, v/v).

^{1}H NMR (400 MHz, Chloroform-*d*) δ 8.3 (m, 1H), 7.9 – 7.8 (m, 2H), 7.5 (mm, 1H), 7.4 (m, 1H), 4.2 (m, 2H), 4.1 (m, 1H), 3.1 (dd, *J* = 14.3, 2.6 Hz, 1H), 2.7 (dd, *J* = 14.3, 5.5 Hz, 1H), 1.4 (s, 3H), 1.3 (s, 4H), 1.1 (t, *J* = 7.1 Hz, 3H).

^{13}C NMR (126 MHz, Chloroform-*d*) δ 190.0, 171.3, 142.8, 139.7, 139.2, 132.6, 127.7, 126.5, 124.9, 122.7, 89.0, 88.9, 77.1, 62.2, 40.8, 26.9, 22.1, 13.9.

HRMS (EI) m/z: $[M]^+$ Calcd. for $C_{18}H_{20}O_5S^+$ 348.1031; Found: 348.1024.

IR (neat, cm^{-1}) 3504, 2980, 1739, 1666, 1509, 1239, 1106, 751.

ethyl 2-(9,10-dioxo-9,10-dihydroanthracene-2-carbonyl)-4-hydroxy-5,5-dimethyltetrahydrofuran-2-carboxylate (60)

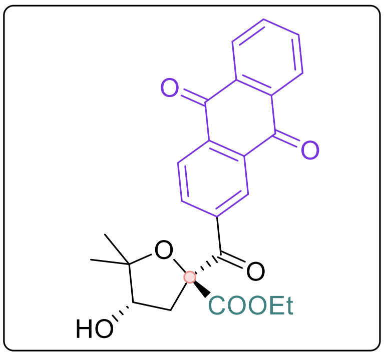

Following the **general procedure F** on 0.1 mmol scale, yellow oil, yield: 41% (17.3 mg), > 20: 1 dr. R_f = 0.3 (silica gel, hexane: EtOAc = 5:1, v/v), column chromatography (silica gel, hexane: EtOAc = 5: 1, v/v).

^{1}H NMR (400 MHz, Chloroform-*d*) δ 8.9 (m, 1H), 8.4 (m, 1H), 8.3 (m, 1H), 8.3 – 8.3 (m, 2H), 7.8 – 7.8 (m, 2H), 4.2 (m, 2H), 4.1 (m, 1H), 3.1 (dd, *J* = 14.2, 2.6 Hz, 1H), 2.7 (dd, *J* = 14.2, 5.3 Hz, 1H), 1.4 (s, 3H), 1.2 (s, 3H), 1.2 (d, *J* = 7.1 Hz, 3H).

^{13}C NMR (126 MHz, Chloroform-*d*) δ 195.4, 182.6, 182.3, 171.2, 139.4, 135.6, 134.7, 134.5, 134.4, 133.5, 133.4, 133.4, 128.9, 127.4, 127.4, 127.2, 89.1, 88.9, 77.0, 62.3, 41.3, 26.9, 22.0, 13.9.

HRMS (EI) m/z: $[M]^+$ Calcd. for $C_{24}H_{22}O_7^+$ 422.1366; Found: 422.1356.

IR (neat, cm^{-1}) 3450, 2931, 1755, 1726, 1710, 1675, 1589, 1273, 1126, 708.

ethyl 2-(4-(*N,N*-dipropylsulfamoyl)benzoyl)-4-hydroxy-1-oxaspiro[4.5]decane-2-carboxylate (61)

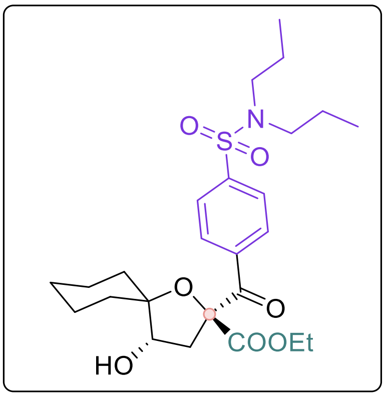

Following the **general procedure F** on 0.1 mmol scale, yellow oil, yield: 61% (18.5 mg), > 20: 1 dr. R_f = 0.3 (silica gel, hexane: EtOAc = 4:1, v/v), column chromatography (silica gel, hexane: EtOAc = 4: 1, v/v).

^{1}H NMR (500 MHz, Chloroform-*d*) δ 8.2 (d, *J* = 8.5 Hz, 2H), 7.9 (d, *J* = 8.6 Hz, 2H), 4.2 – 4.1 (m, 3H), 3.1 – 3.1 (m, 4H), 3.1 (dd, *J* = 14.2, 2.1 Hz, 1H), 2.6 (m, 1H), 1.9 (m, 1H), 1.8 – 1.7 (m, 1H), 1.6 – 1.4 (m, 11H), 1.3 (m, 1H), 1.1 (t, *J* = 7.1 Hz, 3H), 0.9 (t, *J* = 7.4 Hz, 6H).

^{13}C NMR (126 MHz, Chloroform-*d*) δ 195.2, 171.5, 143.9, 137.6, 130.4, 126.7, 90.6, 88.5, 75.7, 62.1, 49.9, 40.8, 35.7, 30.9, 25.4, 23.0, 22.9, 21.9, 13.8, 11.1.

HRMS (EI) m/z: $[M]^+$ Calcd. for $C_{18}H_{26}NO_3^+$ 304.1907; Found: 304.1903.

IR (neat, cm^{-1}) 3518, 2931, 1730, 1698, 1509, 1336, 1155, 1074, 994, 605.

ethyl 2-(4-(5-(2-fluorophenyl)-1,2,4-oxadiazol-3-yl)benzoyl)-4-hydroxy-1-oxaspiro[4.5]decane-2-carboxylate (62)

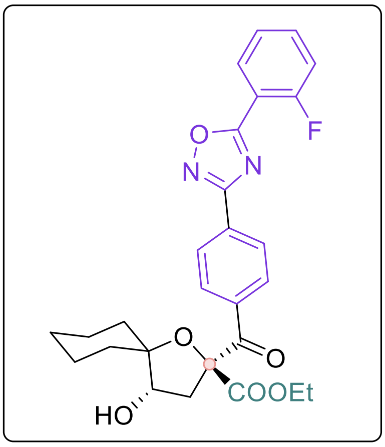

Following the **general procedure F** on 0.1 mmol scale, yellow oil, yield: 41% (20.3 mg), > 20: 1 dr. R_f = 0.3 (silica gel, hexane: EtOAc = 4:1, v/v), column chromatography (silica gel, hexane: EtOAc = 4: 1, v/v).

^{1}H NMR (500 MHz, Chloroform-*d*) δ 8.9 (m, 1H), 8.3 (m, 1H), 8.3 – 8.2 (m, 2H), 7.6 – 7.6 (m, 2H), 7.4 – 7.3 (m, 1H), 7.3 – 7.3 (m, 1H), 4.2 – 4.1 (m, 3H), 3.1 (dd, *J* = 14.4, 2.0 Hz, 1H), 2.7 (dd, *J* = 14.4, 5.5 Hz, 1H), 2.1 (s, 1H), 1.9 (m, 1H), 1.8 – 1.7 (m, 1H), 1.6 – 1.4 (m, 7H), 1.4 – 1.3 (m, 1H), 1.1 (t, *J* = 7.1 Hz, 3H).

^{13}C NMR (126 MHz, Chloroform-*d*) δ 195.2, 173.0, 173.0, 171.8, 168.1, 161.9, 159.8, 135.2, 134.8, 134.7, 132.5, 131.8, 131.0, 129.1, 128.9, 127.2, 124.8, 124.7, 117.3, 117.1, 112.8, 112.7, 90.7, 88.4, 76.0, 62.0, 40.7, 35.7, 31.1, 25.6, 23.0, 22.9.

^{19}F NMR (377 MHz, Chloroform-*d*) δ -108.7.

HRMS (ESI) m/z: $[M]^+$ Calcd. for $C_{27}H_{27}FN_2O_6^+$ 494.1853; Found: 494.1849.

IR (neat, cm^{-1}) 3536, 2976, 1731, 1687, 1511, 1248, 1157.

ethyl 4-hydroxy-5,5-dimethyl-2-(3-methyl-4-oxo-2-phenyl-4H-chromene-6-carbonyl)tetrahydrofuran-2-carboxylate (63)

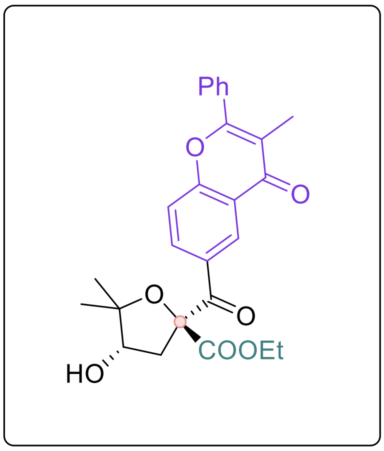

Following the **general procedure F** on 0.1 mmol scale, yellow oil, yield: 58% (26.1 mg), > 20: 1 dr. R_f = 0.3 (silica gel, hexane: EtOAc = 4:1, v/v), column chromatography (silica gel, hexane: EtOAc = 4: 1, v/v).

^{1}H NMR (500 MHz, Chloroform-*d*) δ 8.4 (m, 1H), 8.1 (m, 1H), 7.7 – 7.7 (m, 2H), 7.5 (m, 3H), 7.4 (m, 1H), 4.0 (m, 1H), 3.8 (m, 2H), 2.9 (dd, *J* = 14.2, 2.2 Hz, 1H), 2.8 (dd, *J* = 14.2, 5.0 Hz, 1H), 2.2 (s, 3H), 1.3 (s, 3H), 1.1 (s, 3H), 0.9 (t, *J* = 7.1 Hz, 3H).

^{13}C NMR (126 MHz, Chloroform-*d*) δ 198.2, 178.4, 170.7, 153.5, 134.5, 132.9, 130.3, 129.4, 129.4, 128.4, 127.0, 123.7, 122.7, 117.7, 89.9, 88.9, 76.9, 61.9, 43.0, 26.9, 21.9, 13.7, 11.7.

HRMS (EI) m/z: $[M]^+$ Calcd. for $C_{26}H_{26}O_7^+$ 450.1679; Found: 450.1676.

IR (neat, cm^{-1}) 3534, 2985, 1737, 1726, 1689, 1511, 1225, 1113, 731.

ethyl 2-(4-(*N*,*N*-dipropylsulfamoyl)benzoyl)-4-hydroxy-5,5-dimethyltetrahydrofuran-2-carboxylate (64)

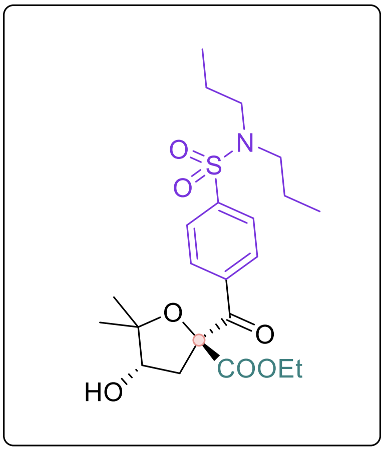

Following the **general procedure F** on 0.1 mmol scale, yellow oil, yield: 50% (22.8 mg), > 20: 1 dr. R_f = 0.3 (silica gel, hexane: EtOAc = 4:1, v/v), column chromatography (silica gel, hexane: EtOAc = 4: 1, v/v).

^{1}H NMR (400 MHz, Chloroform-*d*) δ 8.1 – 8.1 (m, 2H), 7.8 – 7.8 (m, 2H), 4.1 (m, 2H), 4.0 (m, 1H), 3.1 – 3.0 (m, 5H), 2.6 (dd, *J* = 14.2, 5.4 Hz, 1H), 1.5 – 1.4 (m, 5H), 1.3 (s, 3H), 1.1 (s, 3H), 1.0 (t, *J* = 7.1 Hz, 3H), 0.8 (t, *J* = 7.4 Hz, 6H).

^{13}C NMR (126 MHz, Chloroform-*d*) δ 194.9, 171.4, 144.0, 137.5, 130.3, 126.8, 89.1, 88.6, 77.0, 62.2, 49.9, 40.9, 26.9, 22.1, 21.9, 13.8, 11.1.

HRMS (EI) m/z: $[M]^+$ Calcd. for $C_{22}H_{33}NO_7S^+$ 455.1978; Found: 455.1974.

IR (neat, cm^{-1}) 3528, 2936, 1731, 1696, 1509, 1263, 1072, 752.

ethyl 2-(2-(3-cyano-4-isobutoxyphenyl)-4-methylthiazole-5-carbonyl)-4-hydroxy-5,5-dimethyltetrahydrofuran-2-carboxylate (65)

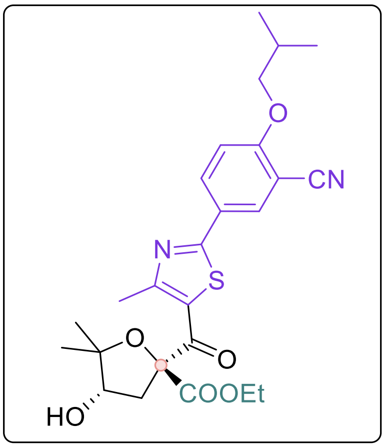

Following the **general procedure F** on 0.1 mmol scale, yellow oil, yield: 61% (31.5 mg), > 20: 1 dr. R_f = 0.3 (silica gel, hexane: EtOAc = 3:1, v/v), column chromatography (silica gel, hexane: EtOAc = 3: 1, v/v).

^{1}H NMR (400 MHz, Chloroform-*d*) δ 8.2 (m, 1H), 8.2 (m, 1H), 7.0 (m, 1H), 4.2 (m, 2H), 4.1 (m, 1H), 3.9 (m, 2H), 3.0 – 3.0 (m, 1H), 2.8 – 2.7 (m, 4H), 2.2 (m, 1H), 1.8 (s, 1H), 1.4 (s, 6H), 1.2 (t, *J* = 7.1 Hz, 3H), 1.1 (s, 3H), 1.1 (s, 3H).

^{13}C NMR (126 MHz, Chloroform-*d*) δ 190.2, 170.3, 169.0, 164.4, 162.5, 132.8, 132.3, 126.0, 123.7, 115.4, 112.6, 102.9, 89.8, 89.2, 76.8, 62.3, 41.1, 28.2, 26.9, 21.8, 19.1, 19.0, 13.9.

HRMS (EI) m/z: [M]$^+$ Calcd. for $C_{25}H_{30}N_2O_6S^+$ 486.1825; Found: 486.1821.

IR (neat, cm^{-1}) 3471, 2922, 1742, 1667, 1603, 1368, 1288, 1115, 1009.

ethyl 4-hydroxy-2-(4-methylbenzoyl)-1,9-dioxaspiro[5.5]undecane-2-carboxylate (66)

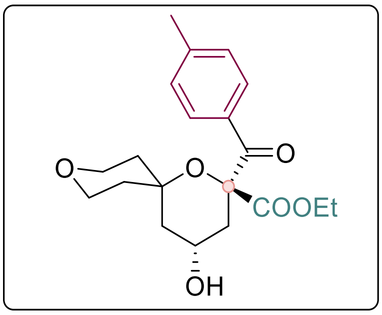

Following the **general procedure F** on 0.1 mmol scale, yellow oil, yield: 48% (17.4 mg), > 20: 1 dr. R_f = 0.3 (silica gel, hexane: EtOAc = 3:1, v/v), column chromatography (silica gel, hexane: EtOAc = 3: 1, v/v).

^{1}H NMR (500 MHz, Chloroform-*d*) δ 8.1 (d, *J* = 8.2 Hz, 2H), 7.2 (m, 2H), 4.3 – 4.2 (m, 1H), 4.2 – 4.1 (m, 2H), 4.0 (m, 1H), 3.7 – 3.6 (m, 2H), 3.6 – 3.5 (m, 1H), 2.6 (m, 1H), 2.4 (s, 3H), 2.1 – 2.1 (m, 1H), 2.0 (m, 1H), 1.8 (m, 1H), 1.7 (m, 1H), 1.6 – 1.6 (m, 2H), 1.5 (m, 1H), 1.1 (t, *J* = 7.1 Hz, 3H).

^{13}C NMR (126 MHz, Chloroform-*d*) δ 196.7, 171.5, 144.3, 131.6, 130.5, 128.9, 84.6, 74.7, 64.0, 63.7, 62.0, 61.6, 43.3, 39.7, 37.8, 36.6, 21.7, 13.8.

HRMS (EI) m/z: [M]$^+$ Calcd. for $C_{20}H_{26}O_6^+$ 362.1729; Found: 362.17726.

IR (neat, cm^{-1}) 3409, 2951, 1735, 1675, 1257, 1235, 1040, 830, 613.

ethyl 4-hydroxy-2-(4-(methoxycarbonyl)benzoyl)-1,9-dioxaspiro[5.5]undecane-2-carboxylate (67)

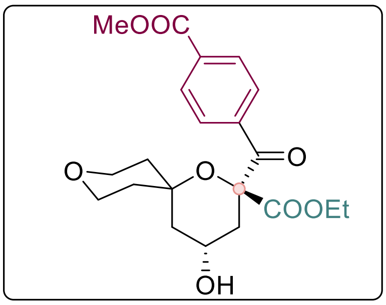

Following the **general procedure F** on 0.1 mmol scale, yellow oil, yield: 42% (17.1 mg), > 20: 1 dr. R_f = 0.3 (silica gel, hexane: EtOAc = 3:1, v/v), column chromatography (silica gel, hexane: EtOAc = 3: 1, v/v).

^{1}H NMR (500 MHz, Chloroform-*d*) δ 8.2 (d, *J* = 8.5 Hz, 2H), 8.1 (d, *J* = 8.6 Hz, 2H), 4.3 – 4.2 (m, 2H), 4.1 (m, 1H), 3.9 (m, 4H), 3.7 (m, 2H), 3.6 (s, 1H), 2.7 (m, 1H), 2.0 – 2.0 (m, 1H), 2.0 (m, 1H), 1.8 (m, 1H), 1.7 (s, 1H), 1.6 – 1.5 (m, 2H), 1.4 (m, 1H), 1.2 (*t*, *J* = 7.1 Hz, 3H).

^{13}C NMR (126 MHz, Chloroform-*d*) δ 197.1, 171.0, 166.2, 138.0, 133.7, 130.0, 129.2, 84.9, 75.3, 63.9, 63.7, 62.3, 61.4, 52.5, 43.7, 40.0, 38.0, 35.9, 13.8.

HRMS (ESI) m/z: [M+Na]$^+$ Calcd. for $C_{21}H_{26}O_8^+$ 429.1526; Found: 429.1518.

IR (neat, cm^{-1}) 3450, 2958, 1727, 1700, 1695, 1282, 1106, 1015.

ethyl 4-hydroxy-2-(4-nitrobenzoyl)-1,9-dioxaspiro[5.5]undecane-2-carboxylate (68)

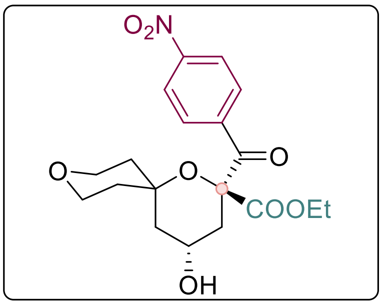

Following the **general procedure F** on 0.1 mmol scale, yellow oil, yield: 44% (17.3 mg), > 20: 1 dr. R_f = 0.3 (silica gel, hexane: EtOAc = 3:1, v/v), column chromatography (silica gel, hexane: EtOAc = 3: 1, v/v).

^{1}H NMR (500 MHz, Chloroform-*d*) δ 8.3 (s, 4H), 4.3 – 4.2 (m, 2H), 4.1 (m, 1H), 3.9 (m, 1H), 3.7 – 3.6 (m, 3H), 2.8 (m, 1H), 2.1 (m, 1H), 1.9 – 1.8 (m, 2H), 1.7 (m, 1H), 1.6 (m, 1H), 1.6 – 1.5 (m, 1H), 1.4 – 1.4 (m, 1H), 1.2 (t, *J* = 7.1 Hz, 3H).

^{13}C NMR (126 MHz, Chloroform-*d*) δ 196.4, 170.6, 150.1, 139.6, 131.1, 123.1, 85.1, 75.8, 64.0, 63.7, 62.5, 61.3, 43.7, 40.1, 38.2, 35.6, 13.8.

HRMS (ESI) m/z: [M]$^+$ Calcd. for $C_{19}H_{23}NO_8^+$ 393.1424; Found: 393.1427.

IR (neat, cm^{-1}) 3412, 2962, 1731, 1695, 1526, 1349, 1230, 854, 718.

ethyl 4-hydroxy-2-(3-methylbenzoyl)-1,9-dioxaspiro[5.5]undecane-2-carboxylate (69)

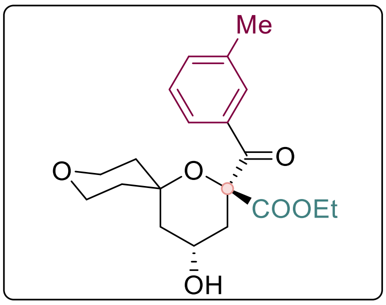

Following the **general procedure F** on 0.1 mmol scale, yellow oil, yield: 43% (15.6 mg), > 20: 1 dr. R_f = 0.3 (silica gel, hexane: EtOAc = 3:1, v/v), column chromatography (silica gel, hexane: EtOAc = 3: 1, v/v).

^{1}H NMR (500 MHz, Chloroform-*d*) δ 8.0 (m, 2H), 7.4 (m, 1H), 7.3 (m, 1H), 4.3 – 4.2 (m, 2H), 4.2 – 4.1 (m, 1H), 4.0 – 3.9 (m, 1H), 3.7 (m, 2H), 3.6 – 3.6 (m, 1H), 2.6 (m, 1H), 2.4 (s, 3H), 2.1 – 2.0 (m, 2H), 1.9 – 1.8 (m, 1H), 1.7 (m, 1H), 1.6 (m, 2H), 1.5 – 1.5 (m, 1H), 1.2 (t, *J* = 7.1 Hz, 3H).

^{13}C NMR (126 MHz, Chloroform-*d*) δ 197.4, 171.4, 137.9, 134.3, 134.0, 130.7, 128.0, 127.5, 84.8, 74.9, 64.0, 63.7, 62.0, 61.5, 43.5, 39.9, 38.0, 36.3, 21.4, 13.8.

HRMS (EI) m/z: $[M]^+$ Calcd. for $C_{20}H_{26}O_6^+$ 362.1729; Found: 362.1726.

IR (neat, cm^{-1}) 3420, 2951, 1733, 1675, 1267, 1153, 1106.

ethyl 2-(3,5-difluorobenzoyl)-4-hydroxy-1,9-dioxaspiro[5.5]undecane-2-carboxylate (70)

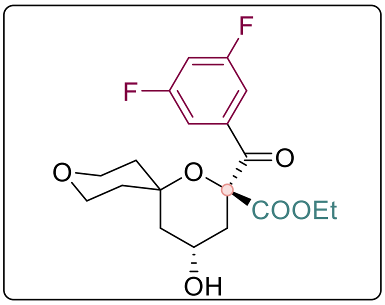

Following the **general procedure F** on 0.1 mmol scale, yellow oil, yield: 47% (18.0 mg), > 20: 1 dr. R_f = 0.3 (silica gel, hexane: EtOAc = 3:1, v/v), column chromatography (silica gel, hexane: EtOAc = 3: 1, v/v).

^{1}H NMR (500 MHz, Chloroform-*d*) δ 7.7 (m, 2H), 7.0 (m, 1H), 4.3 – 4.2 (m, 2H), 4.2 – 4.1 (m, 1H), 4.0 – 3.9 (m, 1H), 3.8 – 3.6 (m, 4H), 2.7 – 2.7 (m, 1H), 2.4 (s, 1H), 2.1 – 2.0 (m, 1H), 1.9 – 1.8 (m, 2H), 1.7 (m, 1H), 1.6 (m, 2H), 1.4 (m, 1H), 1.2 (t, *J* = 7.1 Hz, 3H).

^{13}C NMR (126 MHz, Chloroform-*d*) δ 194.8, 170.7, 162.4 (dd, *J* = 250.0, 11.7 Hz), 137.0 (d, *J* = 8.5 Hz), 114.3, 108.5 (t, *J* = 25.3 Hz), 85.1, 75.7, 64.0, 63.7, 62.4, 61.3, 43.6, 40.0, 38.1, 35.8, 13.8.

^{19}F NMR (471 MHz, Chloroform-*d*) δ -108.4.

HRMS (EI) m/z: $[M]^+$ Calcd. for $C_{19}H_{22}F_2O_6^+$ 384.1384; Found: 384.1383.

IR (neat, cm^{-1}) 3523, 2977, 1728, 1694, 1521, 1234, 1123.

phenyl 2-benzoyl-4-hydroxy-4-methyl-1,8-dioxaspiro[4.5]decane-2-carboxylate (71)

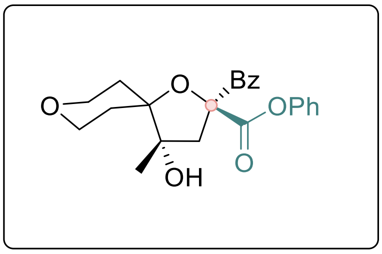

Following the **general procedure F** on 0.1 mmol scale, yellow oil, yield: 86% (34.1 mg), > 20: 1 dr. R_f = 0.3 (silica gel, hexane: EtOAc = 3:1, v/v), column chromatography (silica gel, hexane: EtOAc = 3: 1, v/v).

^{1}H NMR (500 MHz, Chloroform-*d*) δ 8.2 – 8.2 (m, 2H), 7.7 – 7.6 (m, 1H), 7.5 (m, 2H), 7.3 (m, 2H), 7.2 – 7.1 (m, 1H), 6.7 – 6.6 (m, 2H), 4.1 (m, 1H), 4.0 – 3.9 (m, 2H), 3.7 (m, 1H), 3.4 (d, *J* = 14.2 Hz, 1H), 2.6 (d, *J* = 14.2 Hz, 1H), 2.4 (s, 1H), 1.8 – 1.6 (m, 4H), 1.4 (s, 3H).

^{13}C NMR (126 MHz, Chloroform-*d*) δ 194.0, 171.0, 149.9, 134.3, 133.7, 129.8, 129.5, 128.7, 126.4, 120.8, 89.2, 87.0, 79.8, 64.9, 63.8, 45.6, 33.6, 30.2, 21.7.

HRMS (EI) m/z: $[M]^+$ Calcd. for $C_{23}H_{24}O_6^+$ 396.1573; Found: 396.1569.

IR (neat, cm^{-1}) 3426, 2957, 1764, 1690, 1596, 1490, 1188, 1099, 688.

(2S,5R)-2-isopropyl-5-methylcyclohexyl 2-benzoyl-4-hydroxy-4-methyl-1,8-dioxaspiro[4.5]decane-2-carboxylate (72)

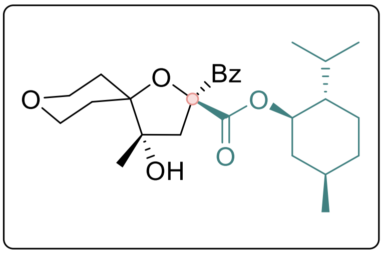

Following the **general procedure F** on 0.1 mmol scale, yellow oil, yield: 89% (40.8 mg) , 1: 1 dr. R_f = 0.3 (silica gel, hexane: EtOAc = 3:1, v/v), column chromatography (silica gel, hexane: EtOAc = 3: 1, v/v).

^{1}H NMR (500 MHz, Chloroform-*d*) δ 8.2 – 8.0 (m, 2H), 7.5 (m, 1H), 7.4 (m, 2H), 4.6 (m, 1H), 4.1 (m, 1H), 4.0 – 3.8 (m, 2H), 3.7 (m, 1H), 3.2 (dd, *J* = 45.1, 14.2 Hz, 1H), 2.4 (dd, *J* = 62.4, 14.1 Hz, 1H), 1.9 – 1.7 (m, 2H), 1.6 (m, 6H), 1.5 (m, 1H), 1.3 (s, 3H), 1.3 – 1.1 (m, 2H), 0.9 (m, 1H), 0.8 – 0.9 (m, 1.5H), 0.8 (m, 1.5H), 0.7 – 0.8 (m, 1.5H), 0.6 (m, 1.5H), 0.6 (m, 1.5H), 0.5 (m, 1.5H), 0.3 – 0.2 (m, 1.5H).

^{13}C NMR (126 MHz, Chloroform-*d*) δ 195.1, 194.1, 171.7, 171.6, 134.3, 133.5, 133.4, 129.9, 129.6, 128.5, 128.4, 88.8, 88.6, 87.0, 87.0, 79.8, 79.7, 76.3, 76.1, 64.9, 63.9, 63.8, 46.5, 46.4, 45.3, 40.2, 39.6, 33.9, 33.9, 33.7, 33.7, 31.3, 31.2, 30.3, 30.2, 26.0, 25.2, 23.0, 22.6, 21.9, 21.8, 21.7, 21.6, 20.7, 20.6, 15.9, 15.2.

HRMS (EI) m/z: $[M]^+$ Calcd. for $C_{27}H_{38}O_6^+$ 458.2668; Found: 458.2674.

IR (neat, cm^{-1}) 3442, 2953, 1743, 1692, 1450, 1103, 1106, 1078, 691.

tert-butyl 2-benzoyl-4-hydroxy-4-methyl-1,8-dioxaspiro[4.5]decane-2-carboxylate (73)

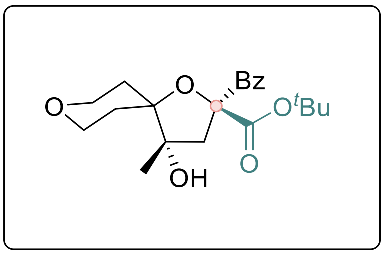

Following the **general procedure F** on 0.1 mmol scale, yellow oil, yield: 90% (33.9 mg), > 20: 1 dr. R_f = 0.3 (silica gel, hexane: EtOAc = 3:1, v/v), column chromatography (silica gel, hexane: EtOAc = 3: 1, v/v).

^{1}H NMR (400 MHz, Chloroform-*d*) δ 8.1 – 8.0 (m, 2H), 7.5 (m, 1H), 7.4 (m, 2H), 4.1 (m, 1H), 4.0 – 3.8 (m, 2H), 3.8 – 3.6 (m, 1H), 3.1 (d, *J* = 14.1 Hz, 1H), 2.4 (s, 1H), 2.4 (d, *J* = 14.1 Hz, 1H), 1.7 (m, 1H), 1.6 – 1.6 (m, 3H), 1.3 (s, 3H), 1.2 (s, 9H).

^{13}C NMR (126 MHz, Chloroform-*d*) δ 195.3, 171.0, 134.6, 133.2, 129.6, 128.3, 88.6, 87.1, 83.0, 79.7, 64.9, 64.0, 45.3, 33.7, 30.3, 27.5, 21.6.

HRMS (ESI) m/z: $[M]^+$ Calcd. for $C_{21}H_{28}O_6^+$ 376.1886; Found: 376.1882.

IR (neat, cm^{-1}) 3469, 2965, 1745, 1694, 1368, 1249, 1142, 1078, 690.

(1R,2R,4R)-1,7,7-trimethylbicyclo[2.2.1]heptan-2-yl 2-benzoyl-4-hydroxy-4-methyl-1,8-dioxaspiro[4.5]decane-2-carboxylate (74)

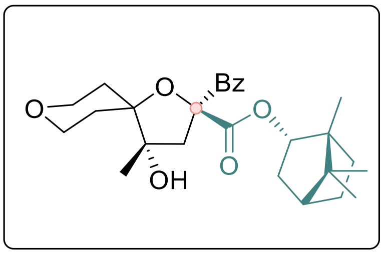

Following the **general procedure F** on 0.1 mmol scale, yellow oil, yield: 87% (39.7 mg) , 1: 1 dr. R_f = 0.3 (silica gel, hexane: EtOAc = 3:1, v/v), column chromatography (silica gel, hexane: EtOAc = 3: 1, v/v).

^{1}H NMR (500 MHz, Chloroform-*d*) δ 8.1 – 8.1 (m, 2H), 7.5 (m, 1H), 7.4 (m, 2H), 4.8 (m, 1H), 4.2 – 4.1 (m, 1H), 3.9 (m, 1H), 3.9 (m, 1H), 3.7 (m, 1H), 3.2 (d, *J* = 14.1 Hz, 1H), 2.4 (t, *J* = 14.5 Hz, 1H), 2.2 (m, 1H), 1.7 (m, 1H), 1.6 (m, 4H), 1.5 – 1.4 (m, 2H), 1.3 (s, 3H), 1.2 (m, 1H), 1.2 – 1.0 (m, 2H), 0.8 (s, 3H), 0.8 (d, *J* = 7.0 Hz, 3H), 0.7 (s, 1.5H), 0.5 (s, 1.5H).

^{13}C NMR (126 MHz, Chloroform-*d*) δ 194.7, 194.6, 172.4, 172.3, 134.5, 134.3, 133.5, 129.8, 129.7, 128.5, 128.5, 88.9, 88.7, 87.1, 86.9, 82.2, 81.9, 79.7, 79.7, 64.9, 64.0, 64.0, 48.8, 48.6, 47.9, 47.6, 45.4, 44.7, 44.5, 36.3, 35.8, 33.7, 30.3, 30.3, 27.8, 27.5, 26.8, 26.6, 21.7, 21.7, 19.5, 18.7, 13.3, 12.7.

HRMS (EI) m/z: $[M]^+$ Calcd. for $C_{27}H_{36}O_6^+$ 456.2512; Found: 456.2519.

IR (neat, cm^{-1}) 3493, 2932, 1745, 1691, 1447, 1268, 1244, 1075, 691.

tert-butyl 2-benzoyl-4-hydroxy-1-oxaspiro[4.5]decane-2-carboxylate (75)

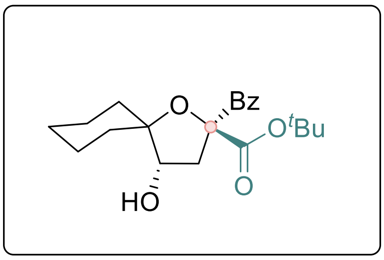

Following the **general procedure F** on 0.1 mmol scale, yellow oil, yield: 68% (16.6 mg), > 20: 1 dr. R_f = 0.3 (silica gel, hexane: EtOAc = 5:1, v/v), column chromatography (silica gel, hexane: EtOAc = 5: 1, v/v).

^{1}H NMR (500 MHz, Chloroform-*d*) δ 8.1 – 8.0 (m, 2H), 7.6 – 7.5 (m, 1H), 7.5 – 7.4 (m, 2H), 4.1 (m, 1H), 3.0 (dd, *J* = 14.3, 2.0 Hz, 1H), 2.5 (dd, *J* = 14.4, 5.7 Hz, 1H), 1.9 (m, 1H), 1.8 – 1.7 (m, 1H), 1.6 – 1.4 (m, 8H), 1.2 (s, 9H).

^{13}C NMR (126 MHz, Chloroform-*d*) δ 196.2, 171.2, 134.8, 133.1, 129.6, 128.3, 90.8, 88.1, 82.7, 76.1, 40.3, 35.9, 31.3, 27.5, 25.6, 23.2, 23.0.

HRMS (EI) m/z: $[M]^+$ Calcd. for $C_{21}H_{28}O_5^+$ 360.1937; Found: 360.1931.

IR (neat, cm^{-1}) 3527, 2946, 1738, 1693, 1456, 1077, 694.

benzyl 2-benzoyl-4-hydroxy-1-oxaspiro[4.5]decane-2-carboxylate (76)

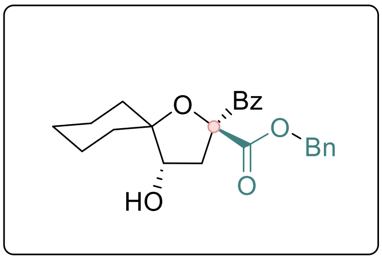

Following the **general procedure F** on 0.1 mmol scale, yellow oil, yield: 65% (25.6 mg), > 20: 1 dr. R_f = 0.3 (silica gel, hexane: EtOAc = 5:1, v/v), column chromatography (silica gel, hexane: EtOAc = 5: 1, v/v).

^{1}H NMR (500 MHz, Chloroform-*d*) δ 8.0 (m, 2H), 7.5 (m, 1H), 7.4 (m, 2H), 7.3 – 7.2 (m, 3H), 7.1 – 7.0 (m, 2H), 5.1 (m, 1H), 5.0 (m, 1H), 4.1 (m, 1H), 3.1 (dd, *J* = 14.4, 2.0 Hz, 1H), 2.6 (dd, *J* = 14.4, 5.6 Hz, 1H), 1.9 (m, 1H), 1.7 (m, 1H), 1.6 – 1.4 (m, 7H), 1.4 – 1.3 (m, 1H).

^{13}C NMR (126 MHz, Chloroform-*d*) δ 195.3, 172.0, 134.8, 134.3, 133.3, 129.8, 128.4, 128.4, 128.3, 128.1, 91.0, 88.0, 76.0, 67.3, 40.5, 35.8, 31.2, 25.5, 23.2, 23.0.

HRMS (EI) m/z: $[M]^+$ Calcd. for $C_{24}H_{26}O_5^+$ 394.1780; Found: 394.1781.

IR (neat, cm^{-1}) 3523, 2916, 1731, 1693, 1459, 1267, 695.

benzyl 10-benzoyl-12-hydroxy-1,4,9-trioxadispiro[4.2.4^8.2^5]tetradecane-10-carboxylate (77)

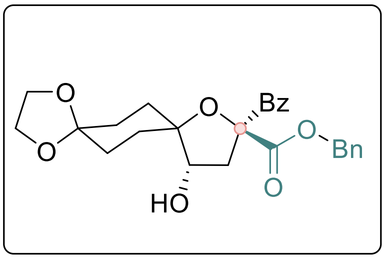

Following the **general procedure F** on 0.1 mmol scale, yellow oil, yield: 51% (26.9 mg), > 20: 1 dr. R_f = 0.3 (silica gel, hexane: EtOAc = 2:1, v/v), column chromatography (silica gel, hexane: EtOAc = 2: 1, v/v).

^{1}H NMR (500 MHz, Chloroform-*d*) δ 8.0 – 8.0 (m, 2H), 7.5 (m, 1H), 7.4 (m, 2H), 7.2 (m, 3H), 7.0 (m, 2H), 5.1 (m, 1H), 5.0 (m, 1H), 4.1 (m, 1H), 3.9 (m, 5H), 3.1 (dd, *J* = 14.4, 2.1 Hz, 1H), 2.5 (dd, *J* = 14.4, 5.6 Hz, 1H), 2.3 – 2.2 (m, 1H), 1.8 – 1.7 (m, 4H), 1.7 – 1.6 (m, 2H), 1.6 (m, 1H).

^{13}C NMR (126 MHz, Chloroform-*d*) δ 194.8, 171.8, 134.7, 134.1, 133.4, 129.8, 128.5, 128.4, 128.4, 128.1, 108.6, 89.4, 88.1, 76.3, 67.4, 64.3, 64.2, 40.4, 32.8, 31.1, 30.9, 28.6.

HRMS (EI) m/z: $[M]^+$ Calcd. for $C_{26}H_{28}O_7^+$ 452.1835; Found: 452.1839.

IR (neat, cm^{-1}) 3523, 2931, 1731, 1691, 1450, 1268, 1250, 1078, 689.

(1R,4R)-1,7,7-trimethylbicyclo[2.2.1]heptan-2-yl 2-benzoyl-4-hydroxy-1-oxaspiro[4.5]decane-2-carboxylate (78)

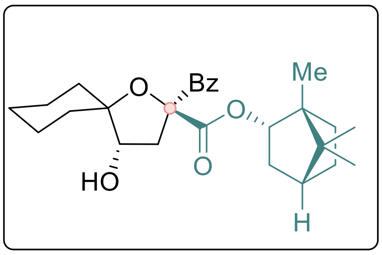

Following the **general procedure F** on 0.1 mmol scale, yellow oil, yield: 61% (52.8 mg) , 1: 1 dr. R_f = 0.3 (silica gel, hexane: EtOAc = 5:1, v/v), column chromatography (silica gel, hexane: EtOAc = 5: 1, v/v).

^{1}H NMR (500 MHz, Chloroform-*d*) δ 8.1 – 8.1 (m, 2H), 7.5 (m, 1H), 7.5 – 7.4 (m, 2H), 4.8 (m, 1H), 4.1 – 4.1 (m, 1H), 3.1 (m, 1H), 2.5 (m, 1H), 2.3 – 2.2 (m, 0.7H), 2.1 – 2.1 (m, 0.6H), 1.9 – 1.9 (m, 1.2H), 1.8 – 1.7 (m, 1.3H), 1.7 – 1.4 (m, 12H), 1.4 – 1.3 (m, 1H), 1.2 – 1.0 (m, 2H), 0.8 (m, 2H), 0.8 (d, *J* = 1.6 Hz, 3H), 0.8 (d, *J* = 5.8 Hz, 3H), 0.7 (s, 1.5H), 0.5 – 0.4 (m, 0.5H), 0.3 (s, 1.5H), 0.3 (m, 0.6H).

^{13}C NMR (126 MHz, Chloroform-*d*) δ 195.5, 172.5, 134.7, 134.5, 133.3, 133.3, 129.8, 129.7, 128.5, 128.4, 91.0, 90.9, 88.0, 87.8, 82.0, 81.6, 76.1, 48.8, 48.6, 47.9, 47.6, 44.7, 44.5, 40.4, 40.4, 36.3, 35.9, 35.9, 35.7, 31.3, 31.3, 27.9, 27.5, 26.8, 26.6, 25.6, 23.2, 23.0, 19.5, 18.7, 13.3, 12.7.

HRMS (EI) m/z: $[M]^+$ Calcd. for $C_{27}H_{36}O_5^+$ 440.2563; Found: 440.2562.

IR (neat, cm^{-1}) 3527, 2945, 1721, 1693, 1441, 1271, 1254, 1035, 692.

1-(2-benzoyl-4-hydroxy-4-methyl-1,8-dioxaspiro[4.5]decan-2-yl)ethan-1-one (79)

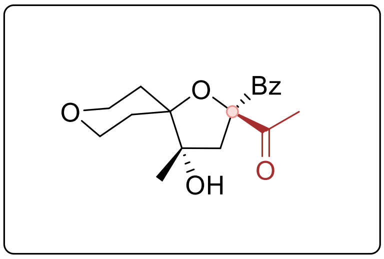

Following the **general procedure F** on 0.1 mmol scale, yellow oil, yield: 70% (22.3 mg), > 20: 1 dr. R_f = 0.3 (silica gel, hexane: EtOAc = 3:1, v/v), column chromatography (silica gel, hexane: EtOAc = 3: 1, v/v).

^{1}H NMR (400 MHz, Chloroform-*d*) δ 8.0 (m, 2H), 7.6 – 7.5 (m, 1H), 7.4 – 7.4 (m, 2H), 4.0 (mm, 1H), 3.9 (m, 2H), 3.7 (m, 1H), 3.1 (d, *J* = 13.7 Hz, 1H), 2.4 (d, *J* = 13.7 Hz, 1H), 2.2 (s, 3H), 1.7 – 1.6 (m, 1H), 1.6 – 1.5 (m, 2H), 1.3 (m, 1H), 1.3 (s, 3H).

^{13}C NMR (126 MHz, Chloroform-*d*) δ 204.7, 197.4, 134.4, 133.5, 130.0, 128.4, 94.5, 87.6, 80.0, 64.8, 63.8, 45.0, 33.8, 30.1, 25.8, 21.8.

HRMS (EI) m/z: $[M]^+$ Calcd. for $C_{18}H_{22}O_5^+$ 318.1467; Found: 318.1460.

IR (neat, cm^{-1}) 3427, 2965, 1718, 1677, 1099, 695.

(4-hydroxy-4-methyl-1,8-dioxaspiro[4.5]decane-2,2-diyl)bis(phenylmethanone) (80)

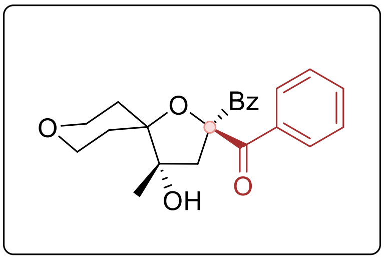

Following the **general procedure F** on 0.1 mmol scale, white solid, m.p. 198-200 ºC, yield: 64% (24.3 mg), > 20: 1 dr. R_f = 0.3 (silica gel, hexane: EtOAc = 3:1, v/v), column chromatography (silica gel, hexane: EtOAc = 3: 1, v/v).

^{1}H NMR (400 MHz, Chloroform-*d*) δ 7.9 – 7.9 (m, 2H), 7.9 (m, 2H), 7.5 – 7.4 (m, 2H), 7.3 (m, 4H), 4.0 – 3.9 (m, 1H), 3.8 – 3.7 (m, 2H), 3.6 (m, 1H), 3.2 (d, *J* = 14.2 Hz, 1H), 2.8 (d, *J* = 14.1 Hz, 1H), 1.9 – 1.8 (m, 1H), 1.8 – 1.6 (m, 2H), 1.3 (m, 1H), 1.3 (s, 3H).

^{13}C NMR (126 MHz, Chloroform-*d*) δ 198.5, 195.9, 134.4, 134.1, 133.8, 133.6, 129.8, 129.6, 128.6, 128.6, 92.0, 87.8, 81.1, 64.9, 63.6, 45.4, 33.1, 30.3, 20.9.

HRMS (EI) m/z: $[M]^+$ Calcd. for $C_{23}H_{24}O_5^+$ 380.1624; Found: 380.1621.

IR (neat, cm^{-1}) 3521, 2921, 1735, 1711, 1448 691, 695.

1-(2-benzoyl-4-hydroxy-4-phenyl-1,8-dioxaspiro[4.5]decan-2-yl)ethan-1-one (81)

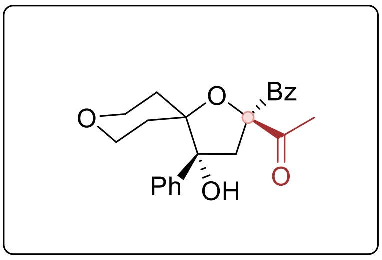

Following the **general procedure F** on 0.1 mmol scale, yellow solid, m.p. 201-203 ºC, yield: 74% (28.1 mg), > 20: 1 dr. R_f = 0.3 (silica gel, hexane: EtOAc = 3:1, v/v), column chromatography (silica gel, hexane: EtOAc = 3: 1, v/v).

^{1}H NMR (400 MHz, Chloroform-*d*) δ 8.0 (m, 2H), 7.6 – 7.5 (m, 1H), 7.4 (m, 4H), 7.4 – 7.3 (m, 3H), 3.9 (m, 1H), 3.8 (m, 1H), 3.7 – 3.7 (m, 2H), 3.2 (m, 2H), 2.3 (s, 1H), 2.3 (s, 3H), 1.7 (m, 1H), 1.7 – 1.6 (m, 1H), 1.3 – 1.2 (m, 1H), 1.0 (m, 1H).

^{13}C NMR (101 MHz, Chloroform-*d*) δ 204.7, 197.8, 139.3, 134.7, 133.4, 130.0, 128.4, 128.3, 128.2, 126.1, 95.0, 88.0, 83.2, 64.9, 63.6, 43.4, 34.6, 29.5, 26.0.

HRMS (EI) m/z: $[M]^+$ Calcd. for $C_{23}H_{24}O_5^+$ 380.1624; Found: 380.1623.

IR (neat, cm^{-1}) 3459, 2968, 1722, 1680, 1098, 1062, 1244, 693.

dimethyl (2-benzoyl-4-hydroxy-4-phenyl-1-oxaspiro[4.6]undecan-2-yl)phosphonate (82)

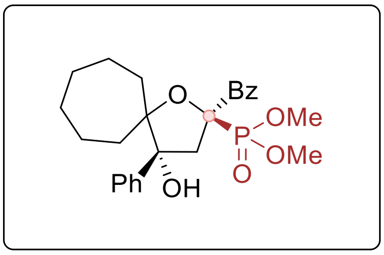

Following the **general procedure F** on 0.1 mmol scale, white solid, m.p. 188-190 ºC, yield: 68% (31.1 mg), > 20: 1 dr. R_f = 0.3 (silica gel, hexane: EtOAc = 3:1, v/v), column chromatography (silica gel, hexane: EtOAc = 3: 1, v/v).

^{1}H NMR (600 MHz, Chloroform-*d*) δ 8.2 (m, 2H), 7.5 (m, 3H), 7.4 (m, 2H), 7.3 – 7.3 (m, 3H), 3.9 (m, 6H), 3.6 (m, 1H), 3.1 (d, *J* = 12.9 Hz, 1H), 1.8 – 1.7 (m, 3H), 1.6 – 1.5 (m, 3H), 1.3 (m, 4H), 1.2 – 1.1 (m, 1H), 0.6 (m, 1H).

^{13}C NMR (126 MHz, Chloroform-*d*) δ 201.9 (d, *J* = 10.1 Hz), 94.3 (d, *J* = 5.6 Hz), 88.8 (d, *J* = 160.7 Hz), 83.3 (d, *J* = 8.0 Hz), 54.4 (d, *J* = 7.2 Hz), 54.0 (d, *J* = 6.9 Hz), 46.5, 38.2, 32.6, 29.7, 29.6, 22.8, 22.0.

^{31}P NMR (243 MHz, Chloroform-*d*) δ 21.8.

HRMS (EI) m/z: $[M]^+$ Calcd. for $C_{25}H_{31}O_6P^+$ 458.1858; Found: 458.1864.

IR (neat, cm^{-1}) 3495, 2948, 1671, 1084, 1023, 693, 557.

diethyl (2-benzoyl-4-hydroxy-4-methyl-1,8-dioxaspiro[4.5]decan-2-yl)phosphonate (83)

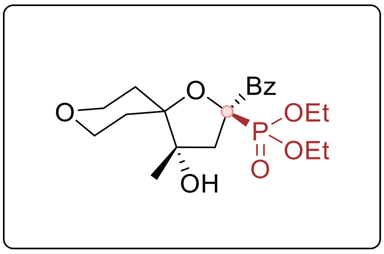

Following the **general procedure F** on 0.1 mmol scale, white solid, m.p. 185-187 ºC, yield: 91% (37.5 mg), > 20: 1 dr. R_f = 0.3 (silica gel, hexane: EtOAc = 2:1, v/v), column chromatography (silica gel, hexane: EtOAc = 2: 1, v/v).

^{1}H NMR (500 MHz, Chloroform-*d*) δ 8.1 (m, 2H), 7.4 (m, 1H), 7.3 (mm, 2H), 4.2 – 4.0 (m, 4H), 3.9 – 3.8 (m, 2H), 3.7 (m, 1H), 3.6 (m, 1H), 3.0 (dd, *J* = 13.1, 1.4 Hz, 1H), 2.7 (dd, *J* = 18.1, 13.1 Hz, 1H), 2.3 (s, 1H), 1.6 (m, 1H), 1.5 (m, 1H), 1.4 – 1.3 (m, 1H), 1.3 (t, *J* = 7.1 Hz, 6H), 1.2 (s, 3H), 1.1 (m, 1H).

^{13}C NMR (126 MHz, Chloroform-*d*) δ 201.7 (d, *J* = 9.3 Hz), 136.6, 131.9, 130.3, 127.2, 88.8 (d, *J* = 161.8 Hz), 87.4 (d, *J* = 4.9 Hz), 78.8 (d, *J* = 7.7 Hz), 64.7, 63.8 (d, *J* = 7.1 Hz), 63.7, 63.3 (d, *J* = 6.9 Hz), 47.7, 34.0, 29.6, 21.6, 16.4 (d, *J* = 3.0 Hz), 16.4 (d, *J* = 2.7 Hz).

^{31}P NMR (202 MHz, Chloroform-*d*) δ 19.1.

HRMS (EI) m/z: $[M]^+$ Calcd. for $C_{20}H_{29}O_7P^+$ 412.1651; Found: 412.1647.

IR (neat, cm^{-1}) 3392, 2960, 1679, 1232, 1094, 1017, 1075, 692, 557.

diethyl (2-benzoyl-4-hydroxy-4-methyl-1-oxaspiro[4.14]nonadecan-2-yl)phosphonate (84)

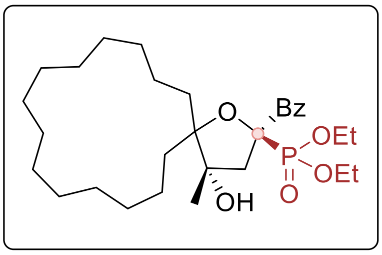

Following the **general procedure F** on 0.1 mmol scale, white solid, m.p. 166-168 ºC, yield: 84% (45.0 mg), > 20: 1 dr. R_f = 0.3 (silica gel, hexane: EtOAc = 2:1, v/v), column chromatography (silica gel, hexane: EtOAc = 2: 1, v/v).

^{1}H NMR (500 MHz, Chloroform-*d*) δ 8.1 – 8.1 (m, 2H), 7.5 – 7.4 (m, 1H), 7.3 (m, 2H), 4.2 – 4.1 (m, 4H), 2.9 (dd, *J* = 13.1, 1.8 Hz, 1H), 2.8 (dd, *J* = 18.0, 13.1 Hz, 1H), 1.6 (m, 1H), 1.5 (m, 2H), 1.5 – 1.3 (m, 9H), 1.3 (m, 23H), 1.2 – 1.1 (m, 2H), 1.1 – 1.0 (m, 1H).

^{13}C NMR (126 MHz, Chloroform-*d*) δ 201.8 (d, *J* = 9.9 Hz), 136.7, 131.9, 130.6, 127.2, 93.0 (d, *J* = 5.1 Hz), 88.8 (d, *J* = 160.8 Hz), 80.1 (d, *J* = 7.9 Hz), 63.6 (d, *J* = 7.1 Hz), 63.1 (d, *J* = 7.1 Hz), 49.2, 35.3, 31.2, 27.9 (d, *J* = 3.0 Hz), 26.9, 26.9, 26.7, 26.6, 26.1 (d, *J* = 12.6 Hz), 22.9, 22.5, 21.9, 16.4 (d, *J* = 3.6 Hz), 16.4 (d, *J* = 4.2 Hz).

^{31}P NMR (202 MHz, Chloroform-*d*) δ 19.2.

HRMS (ESI) m/z: $[M]^+$ Calcd. for $C_{30}H_{49}O_6P^+$ 536.3267; Found: 536.3269.

IR (neat, cm^{-1}) 3499, 2955, 1681, 1074, 1032, 695, 562.

2-benzoyl-4-hydroxy-N-methoxy-N,4-dimethyl-1,8-dioxaspiro[4.5]decane-2-carboxamide (85)

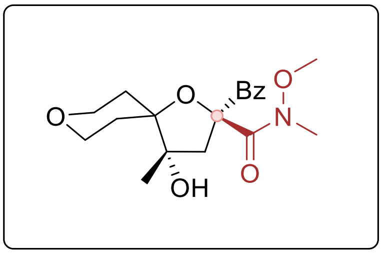

Following the **general procedure F** on 0.1 mmol scale, yellow oil, yield: 69% (25.0 mg), > 20: 1 dr. R_f = 0.3 (silica gel, hexane: EtOAc = 2:1, v/v), column chromatography (silica gel, hexane: EtOAc = 2: 1, v/v).

^{1}H NMR (500 MHz, Chloroform-*d*) δ 7.9 (m, 2H), 7.6 (m, 1H), 7.4 (m, 2H), 4.0 (m, 1H), 3.9 (m, 1H), 3.9 – 3.7 (m, 2H), 3.7 (s, 3H), 3.2 (m, 2H), 3.0 (s, 3H), 2.6 (m, 1H), 1.9 (m, 1H), 1.8 – 1.7 (m, 2H), 1.6 (m, 1H), 1.3 (s, 3H).

^{13}C NMR (126 MHz, Chloroform-*d*) δ 196.1, 172.2, 134.2, 133.3, 128.7, 128.6, 88.1, 86.3, 65.1, 63.9, 61.7, 45.0, 33.2, 30.2, 20.7.

HRMS (EI) m/z: $[M]^+$ Calcd. for $C_{19}H_{25}NO_6^+$ 363.1682; Found: 363.1681.

IR (neat, cm^{-1}) 3445, 2967, 1695, 1665, 1101, 1077, 961, 699, 613.

(4-hydroxy-5,5-dimethyl-2-(trifluoromethyl)tetrahydrofuran-2-yl)(phenyl)methanone (86)

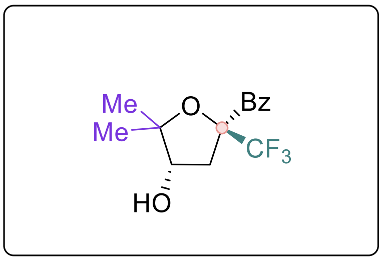

Following the **general procedure F** on 0.1 mmol scale, yellow solid, m.p. 98-101 ºC, yield: 56% (16.1 mg), > 20: 1 dr. R_f = 0.3 (silica gel, hexane: EtOAc = 5:1, v/v), column chromatography (silica gel, hexane: EtOAc = 5: 1, v/v).

^{1}H NMR (400 MHz, Chloroform-*d*) δ 8.1 (m, 2H), 7.5 (m, 1H), 7.4 (m, 2H), 4.0 (m, 1H), 2.9 (dd, *J* = 13.9, 2.6 Hz, 1H), 2.7 (dd, *J* = 13.9, 4.8 Hz, 1H), 1.8 (s, 1H), 1.3 (s, 3H), 1.0 (s, 3H).

^{13}C NMR (126 MHz, Chloroform-*d*) δ 198.1, 135.5, 132.9, 130.6, 127.7, 123.9 (q, *J* = 284.7 Hz), 90.0 (q, *J* = 28.7 Hz), 88.8, 76.1, 40.6, 26.8, 21.7.

^{19}F NMR (377 MHz, Chloroform-*d*) δ -75.3.

HRMS (EI) m/z: $[M]^+$ Calcd. for $C_{14}H_{15}F_3O_3^+$ 288.0973; Found: 288.0966.

IR (neat, cm^{-1}) 3468, 2920, 1674, 1274, 1170, 1113, 1046, 691, 649.

(4-hydroxy-2-(trifluoromethyl)-1-oxaspiro[4.11]hexadecan-2-yl)(phenyl)methanone (87)

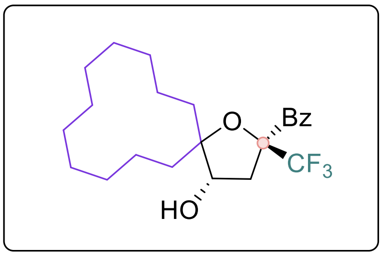

Following the **general procedure F** on 0.1 mmol scale, white solid, m.p. 102-104 °C, yield: 58% (23.9 mg), > 20: 1 dr. R_f = 0.3 (silica gel, hexane: EtOAc = 5:1, v/v), column chromatography (silica gel, hexane: EtOAc = 5: 1, v/v).

^{1}H NMR (500 MHz, Chloroform-*d*) δ 8.1 (m, 2H), 7.6 (m, 1H), 7.4 (m, 2H), 4.1 (m, 1H), 3.0 (dd, *J* = 13.9, 1.9 Hz, 1H), 2.7 (dd, *J* = 13.9, 4.4 Hz, 1H), 1.5 (m, 5H), 1.5 – 1.3 (m, 13H), 1.3 – 1.2 (m, 4H).

^{13}C NMR (126 MHz, Chloroform-*d*) δ 197.8, 135.4, 132.8, 130.7, 127.7, 124.0 (q, *J* = 285.0 Hz), 94.5, 90.1 (q, *J* = 28.7 Hz), 74.8, 40.8, 32.2, 27.8, 26.4, 26.4, 26.0, 22.5, 22.4, 22.1, 22.0, 19.7, 19.4.

HRMS (ESI) m/z: $[M]^+$ Calcd. for $C_{23}H_{31}F_3O_3^+$ 412.2225; Found: 412.2219.

IR (neat, cm^{-1}) 3531, 2952, 1677, 1118, 1046, 688, 654.

(4-hydroxy-4-methyl-2-(trifluoromethyl)-1,8-dioxaspiro[4.5]decan-2-yl)(phenyl)methanone (88)

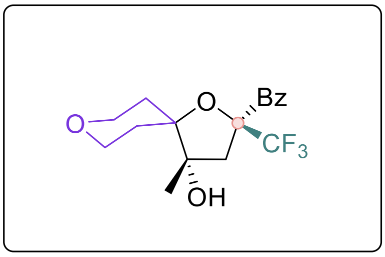

Following the **general procedure F** on 0.1 mmol scale, white solid, m.p. 100-102 °C, yield: 89% (30.6 mg), > 20: 1 dr. R_f = 0.3 (silica gel, hexane: EtOAc = 3:1, v/v), column chromatography (silica gel, hexane: EtOAc = 3: 1, v/v).

^{1}H NMR (500 MHz, Chloroform-*d*) δ 8.1 – 8.0 (m, 2H), 7.5 (m, 1H), 7.4 (m, 2H), 3.9 – 3.8 (m, 2H), 3.8 – 3.8 (m, 1H), 3.7 – 3.6 (m, 1H), 3.0 (d, *J* = 13.4 Hz, 1H), 2.5 (d, *J* = 13.5 Hz, 1H), 1.6 (m, 1H), 1.5 – 1.4 (m, 2H), 1.3 (s, 3H), 1.2 (m, 1H).

^{13}C NMR (126 MHz, Chloroform-*d*) δ 198.0, 135.6, 132.7, 130.3, 127.6, 123.9 (q, *J* = 284.4 Hz), 88.9 (q, *J* = 29.1 Hz), 88.4, 64.5, 63.3, 45.5, 34.0, 29.6, 21.6.

^{19}F NMR (377 MHz, Chloroform-*d*) δ -74.7.

HRMS (ESI) m/z: $[M+Na]^+$ Calcd. for $C_{17}H_{19}F_3NaO_4^+$ 367.1133; Found: 367.1125.

IR (neat, cm^{-1}) 3689, 2980, 1699, 1118, 1069, 700.

(4-hydroxy-4-phenyl-2-(trifluoromethyl)-1,8-dioxaspiro[4.5]decan-2-yl)(phenyl)methanone (89)

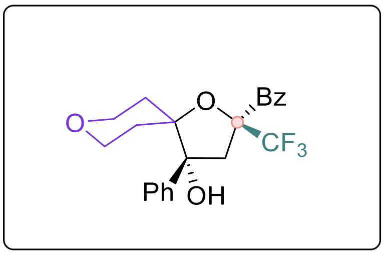

Following the **general procedure F** on 0.1 mmol scale, white solid, m.p. 105-107 ºC, yield: 92% (37.3 mg), > 20: 1 dr. R_f = 0.3 (silica gel, hexane: EtOAc = 3:1, v/v), column chromatography (silica gel, hexane: EtOAc = 3: 1, v/v).

^{1}H NMR (400 MHz, Chloroform-*d*) δ 8.1 (m, 2H), 7.5 (m, 1H), 7.4 (m, 2H), 7.3 (mm, 5H), 3.8 – 3.6 (m, 4H), 3.3 (d, *J* = 13.2 Hz, 1H), 3.1 (d, *J* = 13.3 Hz, 1H), 2.4 (s, 1H), 1.6 – 1.4 (m, 2H), 1.3 (m, 1H), 0.9 (m, 1H).

^{13}C NMR (126 MHz, Chloroform-*d*) δ 198.2, 139.0, 135.5, 132.8, 130.4, 128.6, 128.5, 127.7, 125.9, 124.0 (q, *J* = 282.5 Hz), 88.8, 88.7 (q, *J* = 28.8 Hz), 82.1, 64.5, 63.2, 43.5, 34.6, 29.2.

^{19}F NMR (377 MHz, Chloroform-*d*) δ -74.5.

HRMS (EI) m/z: $[M]^+$ Calcd. for $C_{22}H_{21}F_3O_4^+$ 406.1392; Found: 406.1386.

IR (neat, cm^{-1}) 3521, 2963, 1687, 1123, 1077, 698.

ethyl 2-benzoyl-4-(((S)-3-((tert-butoxycarbonyl)amino)-3-phenylpropanoyl)oxy)-5,5-dimethyltetrahydrofuran-2-carboxylate (90)

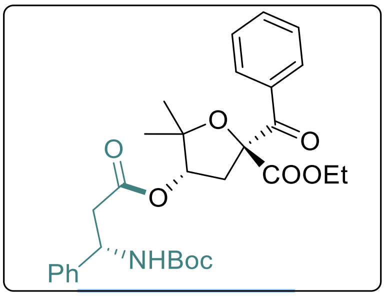

Following the **general procedure F** on 0.1 mmol scale, yellow oil, yield: 87% (46.9 mg), > 20: 1 dr. R_f = 0.3 (silica gel, hexane: EtOAc = 3:1, v/v), column chromatography (silica gel, hexane: EtOAc = 3: 1, v/v).

^{1}H NMR (400 MHz, Chloroform-*d*) δ 8.0 – 7.9 (m, 2H), 7.5 (m, 1H), 7.4 (m, 2H), 7.3 – 7.1 (m, 5H), 5.4 (s, 1H), 5.0 (s, 1H), 4.9 – 4.8 (m, 1H), 4.1 – 4.0 (m, 2H), 3.0 (dd, *J* = 14.4, 3.3 Hz, 1H), 2.8 (m, 2H), 2.6 (m, 1H), 1.3 (s, 9H), 1.3 (m, 3H), 1.0 (t, *J* = 7.1 Hz, 3H), 0.9 (m, 3H).

^{13}C NMR (126 MHz, Chloroform-*d*) δ 193.4, 193.3, 171.5, 170.3, 170.0, 155.0, 140.8, 134.3, 134.3, 133.1, 133.1, 130.0, 130.0, 128.7, 128.6, 128.3, 128.2, 127.6, 127.5, 126.2, 126.1, 88.9, 86.5, 86.4, 79.7, 78.5, 62.1, 51.2, 50.9, 41.0, 40.6, 38.1, 38.0, 28.3, 26.9, 26.8, 22.3, 13.8.

HRMS (ESI) m/z: $[M+H]^+$ Calcd. for $C_{33}H_{37}NO_8^+$ 540.2592; Found: 540.2594.

IR (neat, cm^{-1}) 3387, 2975, 1739, 1705, 1695, 1681, 1368, 1245, 1159, 1065, 696, 662.

ethyl 2-benzoyl-5,5-dimethyl-4-oxotetrahydrofuran-2-carboxylate (91)

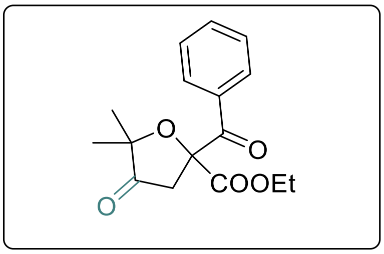

Following the **general procedure F** on 0.1 mmol scale, yellow oil, yield: 92% (26.7 mg). R_f = 0.3 (silica gel, hexane: EtOAc = 10:1, v/v), column chromatography (silica gel, hexane: EtOAc = 10: 1, v/v).

^{1}H NMR (500 MHz, Chloroform-*d*) δ 8.1 – 8.0 (m, 2H), 7.6 – 7.5 (m, 1H), 7.4 (m, 2H), 4.2 – 4.1 (m, 2H), 3.8 (d, *J* = 19.1 Hz, 1H), 2.8 (d, *J* = 19.0 Hz, 1H), 1.5 (s, 3H), 1.1 (s, 3H), 1.0 (t, *J* = 7.1 Hz, 3H).

^{13}**C NMR** (126 MHz, Chloroform-*d*) δ 213.2, 192.0, 170.8, 133.7, 133.5, 129.9, 128.5, 84.6, 83.5, 62.4, 40.3, 24.8, 24.6, 13.7.

HRMS (EI) m/z: [M]$^+$ Calcd. for $C_{16}H_{18}O_5^+$ 290.1154; Found: 290.1151.

IR (neat, cm^{-1}) 2978, 1758, 1733, 1687, 1221, 1132, 1042, 696.

ethyl 2-benzoyl-5,5-dimethyl-2,5-dihydrofuran-2-carboxylate (92)

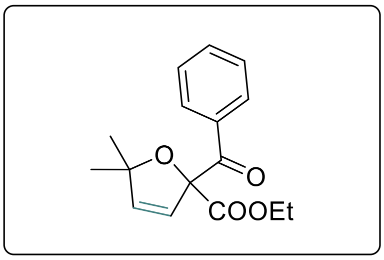

Following the **general procedure F** on 0.1 mmol scale, yellow oil, yield: 65% (17.8 mg). R_f = 0.5 (silica gel, hexane: EtOAc = 10:1, v/v), column chromatography (silica gel, hexane: EtOAc = 10: 1, v/v).

^{1}H NMR (400 MHz, Chloroform-*d*) δ 8.1 (m, 2H), 7.6 – 7.5 (m, 1H), 7.4 (m, 2H), 6.0 (m, 2H), 4.2 – 4.1 (m, 2H), 1.5 (s, 3H), 1.3 (s, 3H), 1.1 (t, *J* = 7.1 Hz, 3H).

^{13}C NMR (126 MHz, Chloroform-*d*) δ 194.7, 170.2, 138.9, 134.2, 133.2, 129.8, 128.4, 124.4, 96.5, 92.2, 61.9, 28.2, 28.1, 13.9.

HRMS (EI) m/z: [M]$^+$ Calcd. for $C_{16}H_{18}O_4^+$ 274.1205; Found: 274.1208.

IR (neat, cm^{-1}) 2978, 1758, 1687, 1215, 1117, 1042, 906, 697.

ethyl 2-benzoyl-4-bromo-5,5-dimethyltetrahydrofuran-2-carboxylate (93)

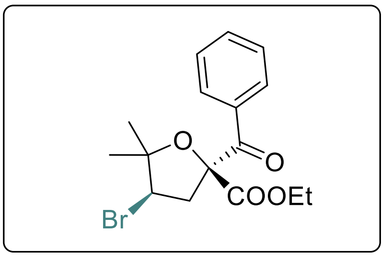

Following the **general procedure F** on 0.1 mmol scale, yellow oil, yield: 74% (26.2 mg), > 20: 1 dr. R_f = 0.5 (silica gel, hexane: EtOAc = 10:1, v/v), column chromatography (silica gel, hexane: EtOAc = 10: 1, v/v).

^{1}H NMR (400 MHz, Chloroform-*d*) δ 8.0 (dd, *J* = 8.4, 1.4 Hz, 2H), 7.6 – 7.5 (m, 1H), 7.4 (dd, *J* = 8.4, 7.0 Hz, 2H), 4.1 (qd, *J* = 7.1, 3.8 Hz, 2H), 4.0 (dd, *J* = 10.0, 6.9 Hz, 1H), 3.7 (dd, *J* = 13.4, 6.9 Hz, 1H), 2.6 (dd, *J* = 13.4, 10.1 Hz, 1H), 1.5 (s, 3H), 1.3 (s, 3H), 1.1 (t, *J* = 7.1 Hz, 3H).

^{13}C NMR (126 MHz, Chloroform-*d*) δ 192.0, 171.1, 133.8, 133.4, 129.9, 128.4, 87.3, 87.1, 62.0, 51.8, 41.4, 26.4, 25.0, 13.8.

HRMS (EI) m/z: $[M]^+$ Calcd. for $C_{16}H_{19}BrO_4^+$ 354.0467; Found: 354.0460.

IR (neat, cm^{-1}) 2977, 1757, 1693, 1449, 1231, 1075, 946, 687.

ethyl 2-benzoyl-4- (Ferrocenoyloxy)-5,5-dimethyltetrahydrofuran-2-carboxylate (94)

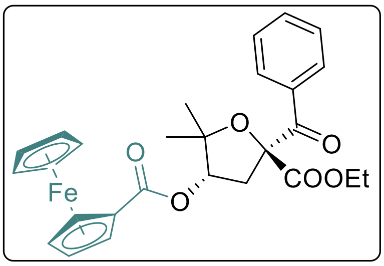

Following the **general procedure F** on 0.1 mmol scale, yellow oil, yield: 80% (40.3 mg), > 20: 1 dr. R_f = 0.4 (silica gel, hexane: EtOAc = 10:1, v/v), column chromatography (silica gel, hexane: EtOAc = 10: 1, v/v).

^{1}H NMR (400 MHz, Chloroform-*d*) δ 8.1 (m, 2H), 7.5 – 7.4 (m, 1H), 7.4 (m, 2H), 5.2 (m, 1H), 4.7 – 4.6 (m, 2H), 4.3 (s, 2H), 4.1 (s, 5H), 4.1 (m, 2H), 3.2 (dd, *J* = 14.4, 2.6 Hz, 1H), 2.8 (dd, *J* = 14.4, 5.6 Hz, 1H), 1.4 (s, 3H), 1.2 (s, 3H), 1.0 (t, *J* = 7.1 Hz, 3H).

^{13}C NMR (126 MHz, Chloroform-*d*) δ 193.2, 171.6, 171.0, 134.3, 133.1, 130.1, 128.3, 89.5, 87.0, 77.7, 71.4, 71.4, 70.7, 70.3, 70.0, 69.8, 62.2, 38.8, 27.0, 22.7, 13.9.

HRMS (EI) m/z: $[M]^+$ Calcd. for $C_{27}H_{28}FeO_6^+$ 504.1229; Found: 504.1224.

IR (neat, cm^{-1}) 2980, 1752, 1715, 1693, 1453, 1274, 1137, 1065, 691.

ethyl 2-benzoyl-4-((tert-butyldimethylsilyl)oxy)-5,5-dimethyltetrahydrofuran-2-carboxylate (95)

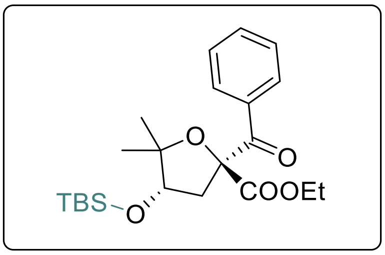

Following the **general procedure F** on 0.1 mmol scale, yellow oil, yield: 61% (24.8 mg), > 20: 1 dr. R_f = 0.5 (silica gel, hexane: EtOAc = 10:1, v/v), column chromatography (silica gel, hexane: EtOAc = 10: 1, v/v).

^{1}H NMR (500 MHz, Chloroform-*d*) δ 8.0 (m, 2H), 7.5 (m, 1H), 7.4 (m, 2H), 4.1 (m, 2H), 4.0 (m, 1H), 3.0 (dd, *J* = 13.5, 5.8 Hz, 1H), 2.5 (dd, *J* = 13.5, 5.9 Hz, 1H), 1.3 (s, 3H), 1.1 (t, *J* = 7.1 Hz, 3H), 1.0 (s, 3H), 0.8 (s, 9H), 0.1 (s, 3H), 0.0 (s, 3H).

^{13}C NMR (126 MHz, Chloroform-*d*) δ 194.7, 172.0, 134.8, 132.7, 129.9, 128.0, 88.3, 86.8, 77.2, 61.9, 40.9, 26.9, 25.7, 22.3, 18.0, 13.8, -4.7, -5.1.

HRMS (EI) m/z: $[M]^+$ Calcd. for $C_{22}H_{34}O_5Si^+$ 406.2176; Found: 406.2176.

IR (neat, cm^{-1}) 2987, 1737, 1689, 1452, 1239, 1068, 697.

ethyl4-hydroxy-2-((hydroxyimino)(phenyl)methyl)-5,5-dimethyltetrahydrofuran-2-carboxylate (96)

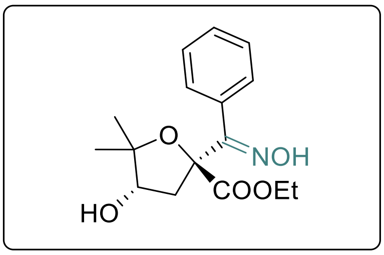

Following the **general procedure F** on 0.1 mmol scale, yellow oil, yield: 62% (22.4 mg), > 20: 1 dr. R_f = 0.3 (silica gel, hexane: EtOAc = 3:1, v/v), column chromatography (silica gel, hexane: EtOAc = 3: 1, v/v).

^{1}H NMR (400 MHz, Chloroform-*d*) δ 10.0 (s, 1H), 7.5 – 7.4 (m, 2H), 7.4 – 7.2 (m, 3H), 5.3 (s, 1H), 4.0 – 3.8 (m, 3H), 2.9 (d, *J* = 14.7 Hz, 1H), 2.6 (dd, *J* = 14.7, 5.6 Hz, 1H), 1.3 (s, 3H), 1.2 (s, 3H), 0.8 (t, *J* = 7.1 Hz, 3H).

^{13}C NMR (126 MHz, Chloroform-*d*) δ 172.2, 158.6, 131.1, 129.3, 128.3, 128.0, 89.9, 87.2, 77.6, 61.6, 41.9, 27.2, 23.2, 13.6.

HRMS (ESI) m/z: $[M+Na]^+$ Calcd. for $C_{16}H_{21}NO_5^+$ 330.1318; Found: 330.1315.

IR (neat, cm^{-1}) 3674, 2935, 1748, 1696, 1449, 1251, 1067, 833, 755, 690.

diethyl 2,3-bis((2,2,6,6-tetramethylpiperidin-1-yl)oxy)maleate (98)

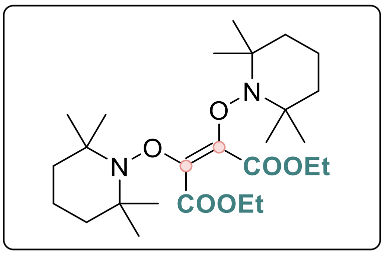

Colorless liquid, yield: 7% (3.5 mg). R_f = 0.3 (silica gel, hexane: EtOAc = 10:1, v/v), column chromatography (silica gel, hexane: EtOAc = 10: 1, v/v).

^{1}H NMR (400 MHz, Chloroform-*d*) δ 4.3 (q, *J* = 7.2 Hz, 2H), 1.7 (s, 6H), 1.5 (s, 12H), 1.3 (t, *J* = 7.1 Hz, 3H).
^{13}C NMR (101 MHz, Chloroform-*d*) δ 164.8, 164.5, 61.7, 57.1, 37.7, 29.1, 14.9, 13.8.

HRMS (ESI) m/z: $[M+Na]^+$ Calcd. for $C_{26}H_{46}N_2NaO_6^+$ 505.3248; Found: 505.3244.

IR (neat, cm^{-1}) 2937, 1732, 1636, 1252, 1204.

ethyl 2-benzoyl-4-hydroxy-1,9-dioxaspiro[5.5]undecane-2-carboxylate (102)

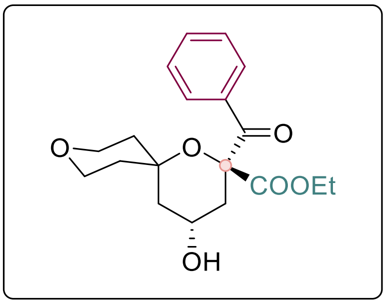

Following the **general procedure F** on 0.1 mmol scale, yellow oil, yield: 44% (15.3 mg), > 20: 1 dr. R_f = 0.3 (silica gel, hexane: EtOAc = 2:1, v/v), column chromatography (silica gel, hexane: EtOAc = 2: 1, v/v).

^{1}H NMR (400 MHz, Chloroform-*d*) δ 8.1 (m, 2H), 7.6 – 7.5 (m, 1H), 7.4 (m, 2H), 4.3 – 4.2 (m, 1H), 4.1 (m, 2H), 4.0 (m, 1H), 3.7 (m, 2H), 3.6 (m, 1H), 2.7 – 2.6 (m, 1H), 2.1 (dd, *J* = 13.6, 8.6 Hz, 1H), 2.0 (m, 1H), 1.9 – 1.8 (m, 1H), 1.7 (m, 1H), 1.6 (m, 2H), 1.5 (m, 1H), 1.1 (t, *J* = 7.1 Hz, 3H).

^{13}C NMR (126 MHz, Chloroform-*d*) δ 197.2, 171.4, 134.3, 133.3, 130.2, 128.2, 84.7, 74.9, 64.0, 63.7, 62.1, 61.5, 43.4, 39.8, 37.9, 36.4, 13.8.

HRMS (EI) m/z: $[M]^+$ Calcd. for $C_{19}H_{24}NO_6^+$ 348.1573; Found: 348.1565.

IR (neat, cm^{-1}) 3448, 2963, 1729, 1688, 1282, 1121, 1019, 692.

cyclopropyl(2-((ethylperoxy)-λ^2-methyl)-4-hydroxy-5,5-dimethyltetrahydrofuran-2-yl)methanone (104)

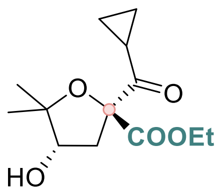

Following the **general procedure F** on 0.1 mmol scale, yellow oil, yield: 77% (19.7 mg), > 20: 1 dr. R_f = 0.3 (silica gel, hexane: EtOAc = 5:1, v/v), column chromatography (silica gel, hexane: EtOAc = 5: 1, v/v).

^{1}H NMR (400 MHz, Chloroform-*d*) δ 4.2 (m, 2H), 4.0 (mm, 1H), 2.7 (dd, *J* = 14.2, 5.4 Hz, 1H), 2.6 (dd, *J* = 14.2, 2.6 Hz, 1H), 2.4 – 2.4 (m, 1H), 1.3 (s, 3H), 1.3 – 1.2 (m, 6H), 1.1 – 1.0 (m, 2H), 1.0 – 0.9 (m, 2H).

^{13}C NMR (126 MHz, Chloroform-*d*) δ 208.1, 170.8, 89.5, 87.9, 77.2, 62.0, 40.4, 26.7, 22.0, 16.6, 14.0, 12.9, 12.2.

HRMS (EI) m/z: $[M]^+$ Calcd. for $C_{13}H_{20}O_5^+$ 256.1311; Found: 256.1316.

IR (neat, cm^{-1}) 3606, 2970, 1731, 1707, 1382, 1263, 1064, 833, 732.

(2-((ethylperoxy)-l2-methyl)-4-hydroxy-4-methyl-1,8-dioxaspiro[4.5]decan-2-yl)(phenyl)methanone oxime (108)

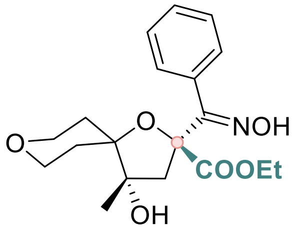

Following the **general procedure F** on 0.1 mmol scale, yellow oil, yield: 51% (18.5 mg), > 20: 1 dr. R_f = 0.3 (silica gel, hexane: EtOAc = 2:1, v/v), column chromatography (silica gel, hexane: EtOAc = 2: 1, v/v).

^{1}H NMR (500 MHz, Chloroform-*d*) δ 10.2 (s, 1H), 7.6 – 7.5 (m, 2H), 7.4 – 7.3 (m, 3H), 5.6 – 5.4 (m, 1H), 4.2 – 4.1 (m, 1H), 4.0 (m, 1H), 3.9 – 3.8 (m, 4H), 3.1 (d, *J* = 14.6 Hz, 1H), 2.5 (d, *J* = 14.5 Hz, 1H), 1.8 (mm, 1H), 1.7 – 1.6 (m, 2H), 1.5 – 1.4 (m, 1H), 1.3 (s, 3H), 0.8 (t, *J* = 7.1 Hz, 3H).

^{13}C NMR (126 MHz, Chloroform-*d*) δ 172.5, 158.3, 131.0, 129.4, 128.4, 128.0, 89.2, 85.3, 80.2, 65.0, 63.8, 61.4, 47.0, 33.8, 31.0, 20.7, 13.5.

HRMS (ESI) m/z: $[M+Na]^+$ Calcd. for $C_{16}H_{21}NO_5^+$ 386.1574; Found: 386.1579.

IR (neat, cm^{-1}) 3574, 2948, 1738, 1686, 1456, 1251, 1039, 693.

ethyl 2-diazo-3-(4,4-dimethyl-2-phenyl-4,5-dihydrooxazol-5-yl)propanoate (110)

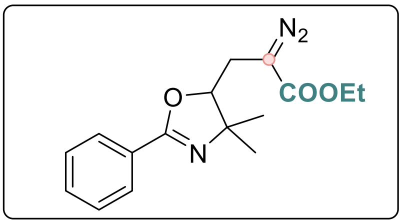

^{1}H NMR (400 MHz, Chloroform-*d*) δ 7.9 – 7.9 (m, 2H), 7.4 (d, *J* = 7.4 Hz, 1H), 7.4 – 7.3 (m, 2H), 4.4 (dd, *J* = 11.2, 2.1 Hz, 1H), 4.3 – 4.3 (m, 2H), 2.8 (dd, *J* = 14.4, 11.2 Hz, 1H), 2.5 (dd, *J* = 14.2, 2.1 Hz, 1H), 1.3 (t, *J* = 7.1 Hz, 3H), 1.3 (s, 3H), 1.1 (s, 3H).

^{13}C NMR (126 MHz, Chloroform-*d*) δ 168.0, 161.4, 136.4, 131.6, 128.4, 128.2, 86.1, 68.7, 61.6, 61.6, 30.9, 28.8, 22.4, 14.1.

HRMS (ESI) m/z: $[M-N_2]^+$ Calcd. for $C_{16}H_{20}NO_3^+$ 274.1365; Found: 274.1371.

7. Supporting References

1. Khan, I.; Reed-Berendt, B. G.; Melen, R. L.; Morrill, L. C. FLP-catalyzed transfer hydrogenation of silyl enol ethers. *Angew. Chem. Int. Ed.* **2018**, *57*, 12356–12359.
2. Zhao, G.; Lim, S.; Musaev, D. G.; Ngai, M.-Y. Visible-light-induced phosphine-catalyzed 1,3-carbobromination of allyl carboxylates via 1,2-radical migration. *J. Am. Chem. Soc.* **2023**, *145*, 8275–8284.
3. Wang, Z.; Herraiz, A.; del Hoyo; A.; Suero, M. G. Generating carbyne equivalents with photoredox catalysis. *Nature* **2018**, *554*, 86–91.
4. Cismesia, M. A.; Yoon, T. P. Characterizing chain processes in visible light photoredox catalysis. *Chem. Sci.* **2015**, *6*, 5426–5434.
5. Montalti, M.; Murov, S. L. H. o. p. Handbook of photochemistry, Taylor & Francis, Boca Raton, 2005.
6. Gaussian 16, Revision A.03, Frisch, M. J.; Trucks, G. W.; Schlegel, H. B.; Scuseria, G. E.; Robb, M. A.; Cheeseman, J. R.; Scalmani, G.; Barone, V.; Petersson, G. A.; Nakatsuji, H.; Li, X.; Caricato, M.; Marenich, A. V.; Bloino, J.; Janesko, B. G.; Gomperts, R.; Mennucci, B.; Hratchian, H. P.; Ortiz, J. V.; Izmaylov, A. F.; Sonnenberg, J. L.; Williams-Young, D.; Ding, F.; Lipparini, F.; Egidi, F.; Goings, J.; Peng, B.; Petrone, A.; Henderson, T.; Ranasinghe, D.; Zakrzewski, V. G.; Gao, J.; Rega, N.; Zheng, G.; Liang, W.; Hada, M.; Ehara, M.; Toyota, K.; Fukuda, R.; Hasegawa, J.; Ishida, M.; Nakajima, T.; Honda, Y.; Kitao, O.; Nakai, H.; Vreven, T.; Throssell, K.; Montgomery, J. A., Jr.; Peralta, J. E.; Ogliaro, F.; Bearpark, M. J.; Heyd, J. J.; Brothers, E. N.; Kudin, K. N.; Staroverov, V. N.; Keith, T. A.; Kobayashi, R.; Normand, J.; Raghavachari, K.; Rendell, A. P.; Burant, J. C.; Iyengar, S. S.; Tomasi, J.; Cossi, M.; Millam, J.

M.; Klene, M.; Adamo, C.; Cammi, R.; Ochterski, J. W.; Martin, R. L.; Morokuma, K.; Farkas, O.; Foresman, J. B.; Fox, D. J. Gaussian, Inc., Wallingford CT, 2016.

7. Becke, A. D. Density-functional thermochemistry. III. The role of exact exchange. *J. Chem. Phys.* **1993**, *98*, 5648–5652.
8. Lee, C.; Yang, W.; Parr, R. G. Development of the Colle-Salvetti correlation-energy formula into a functional of the electron density. *Phys. Rev. B* **1998**, *37*, 785–789.
9. Weigend, F.; Ahlrichs, R. Balanced basis sets of split valence, triple zeta valence and quadruple zeta valence quality for H to Rn: Design and assessment of accuracy. *Phys. Chem. Chem. Phys.* **2005**, *7*, 3297–3305.
10. Grimme, S.; Antony, J.; Ehrlich, S.; Krieg, H. A consistent and accurate *ab initio* parametrization of density functional dispersion correction (DFT-D) for the 94 elements H–Pu. *J. Chem. Phys.* **2010**, *132*, 154104.
11. Tomasi, J.; Mennucci, B.; Cammi, R. Quantum mechanical continuum solvation models. *Chem. Rev.* **2005**, *105*, 2999–3094.
12. Fukui, K. Formulation of the reaction coordinate. *J. Phys. Chem.* **1970**, *74*, 4161–4163.
13. Fukui, K. The path of chemical reactions – the IRC approach. *Acc. Chem. Res.* **1981**, *14*, 363–368.
14. Jiang, L.; Burke, S. D. A novel route to the F-ring of halichondrin B: diastereoselection in Pd(0)-mediated meso and C_2 diol desymmetrization. *Org. Lett.* **2002**, *4*, 3411–3414.
15. Kobayashi, S.; Yokoi, T.; Inoue, T.; Hori, Y.; Saka, T.; Shimomura, T.; Masuyama, A. Stereocontrolled synthesis of a possible stereoisomer of laurenidificin and a formal total synthesis of (+)-aplysiallene featuring a stereospecific ring contraction. *J. Org. Chem.* **2016**, *81*, 1484–1498.
16. Carson, W. P. II.; Sarver, P. J.; Goudy, N. S.; MacMillan, D. W. C. Photoredox catalysis-enabled sulfination of alcohols and bromides. *J. Am. Chem. Soc.* **2023**, *145*, 20767–20774.
17. Shao, Y.-P.; Liang, Y.-M. Dynamic kinetic reductive Grignard-type addition for the construction of axial and central chirality. *ACS Catal.* **2025**, *15*, 1147–1157.
18. Li, Z.; Wang, S.; Chen, S.-C.; Zhu, X.; Lian, Z.; Xing, D. Cu-catalyzed asymmetric three-component radical acylarylation of vinylarenes with aldehydes and aryl boronic acids. *J. Am. Chem. Soc.* **2024**, *146*, 32235–32242.

8. NMR Spectroscopic Data

4-(prop-1-en-2-yl)tetrahydro-2H-pyran-4-yl benzoate (1a)

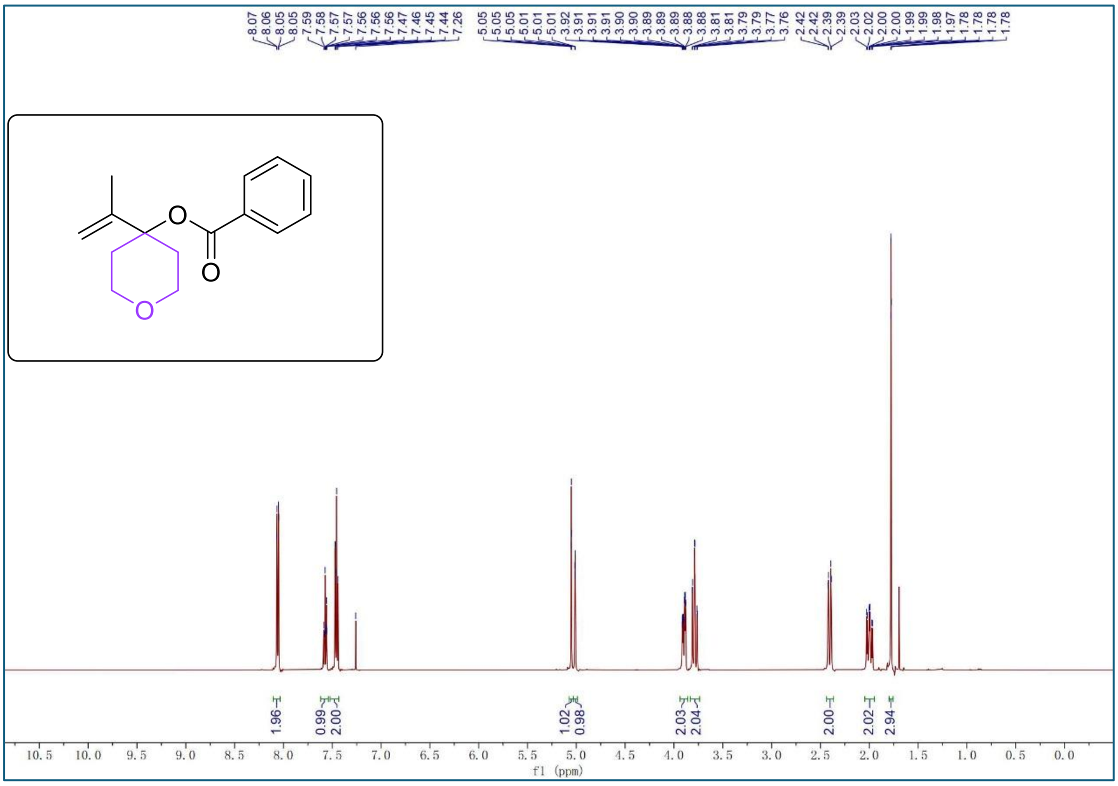

^{1}H NMR-spectrum (500 MHz, Chloroform-*d*) of **1a**

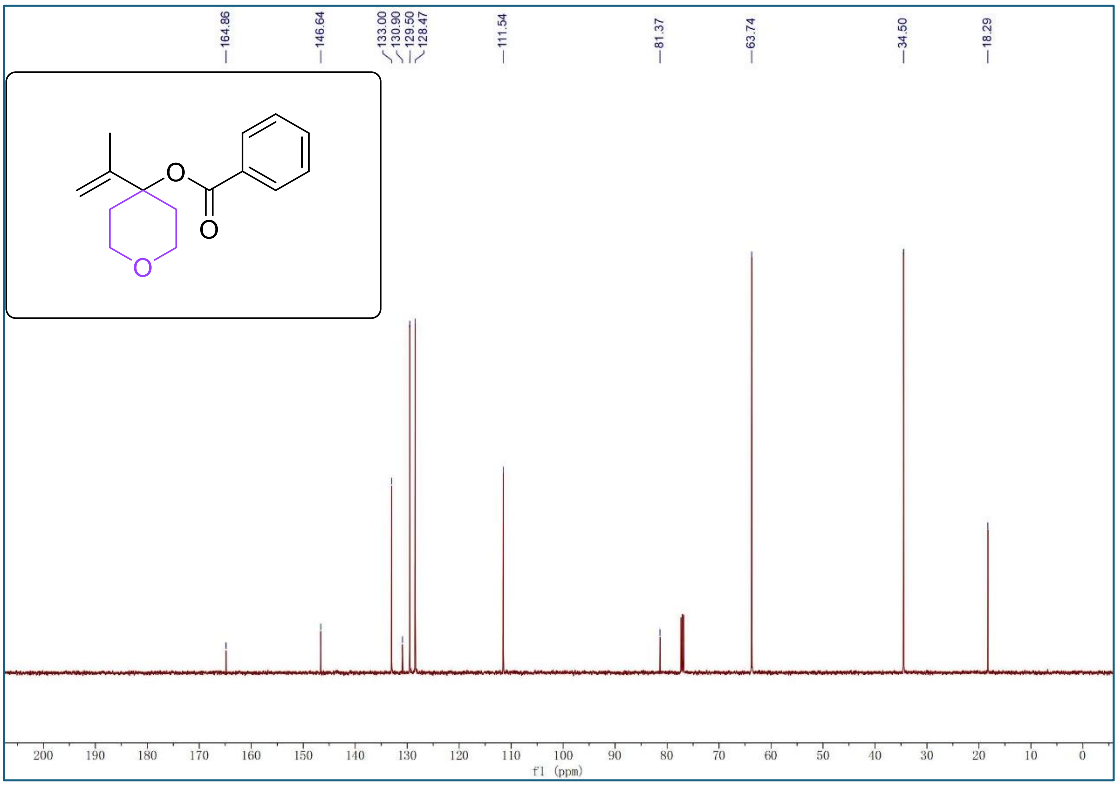

^{13}C NMR-spectrum (126 MHz, Chloroform-*d*) of **1a**

1-(prop-1-en-2-yl)cyclohexyl benzoate (1b)

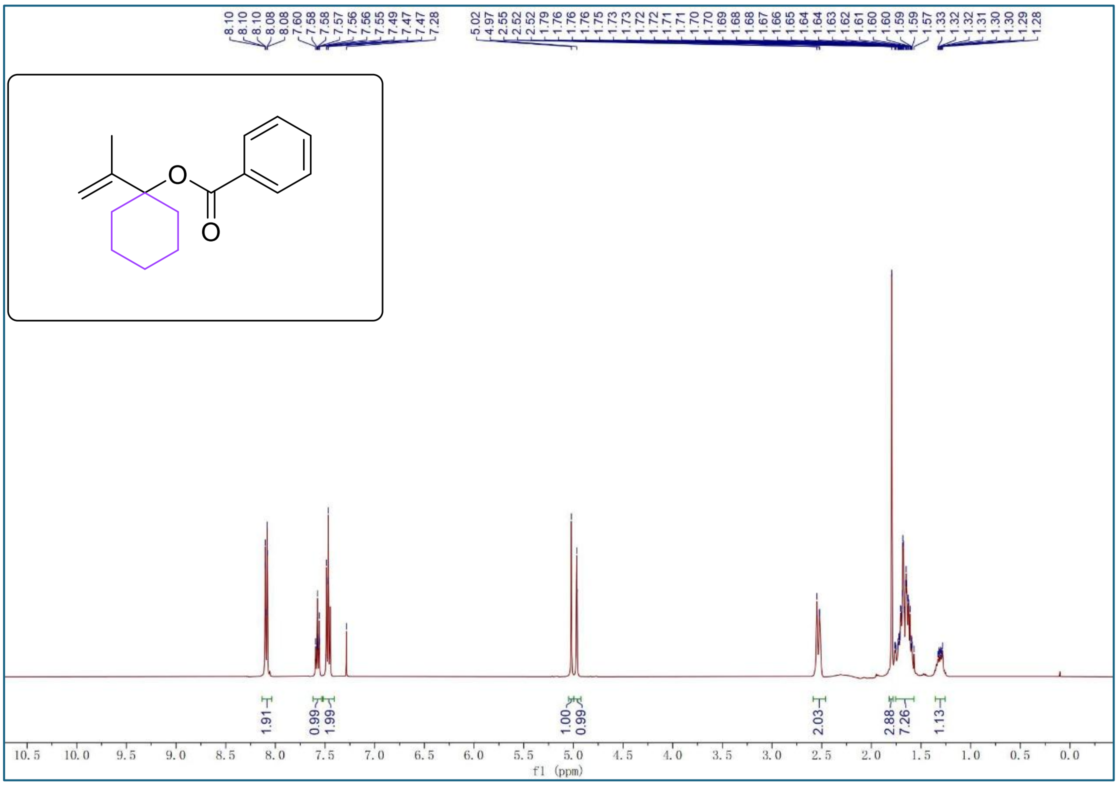

^{1}H NMR-spectrum (400 MHz, Chloroform-*d*) of **1b**

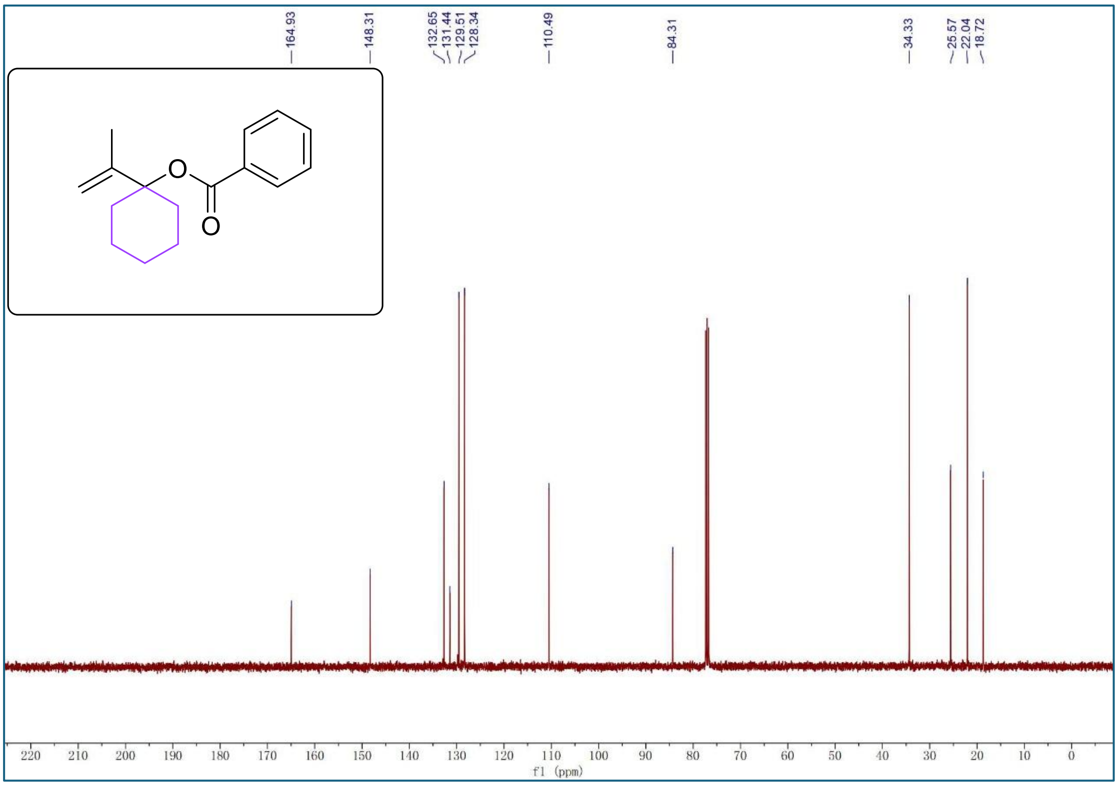

^{13}C NMR-spectrum (101 MHz, Chloroform-*d*) of **1b**

1-(prop-1-en-2-yl)cycloheptyl benzoate (1c)

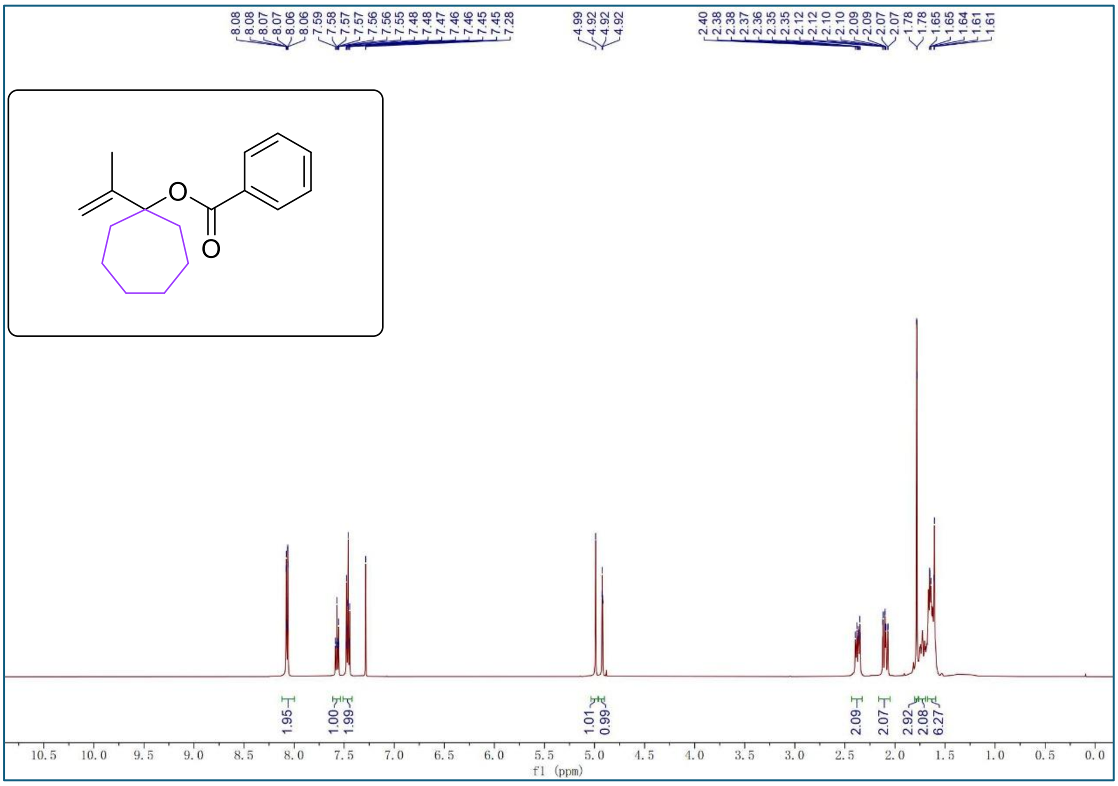

^{1}H NMR-spectrum (500 MHz, Chloroform-*d*) of **1c**

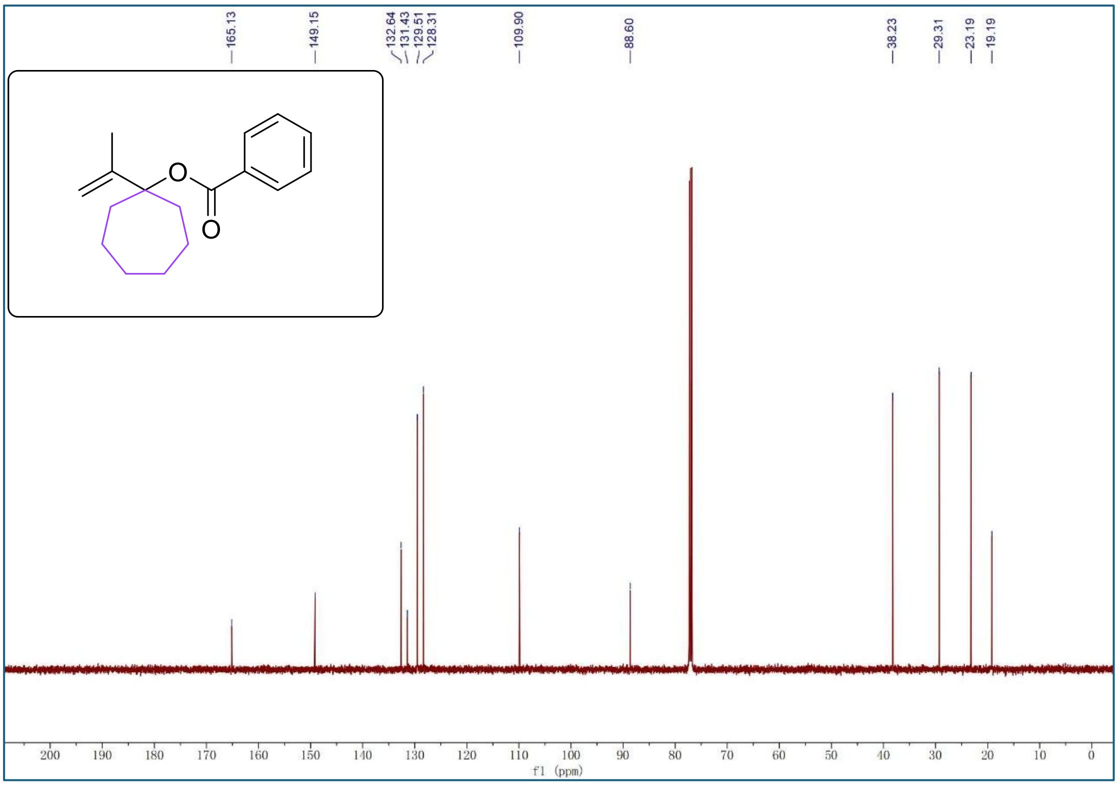

^{13}C NMR-spectrum (126 MHz, Chloroform-*d*) of **1c**

1-(prop-1-en-2-yl)cyclopentadecyl benzoate (1d)

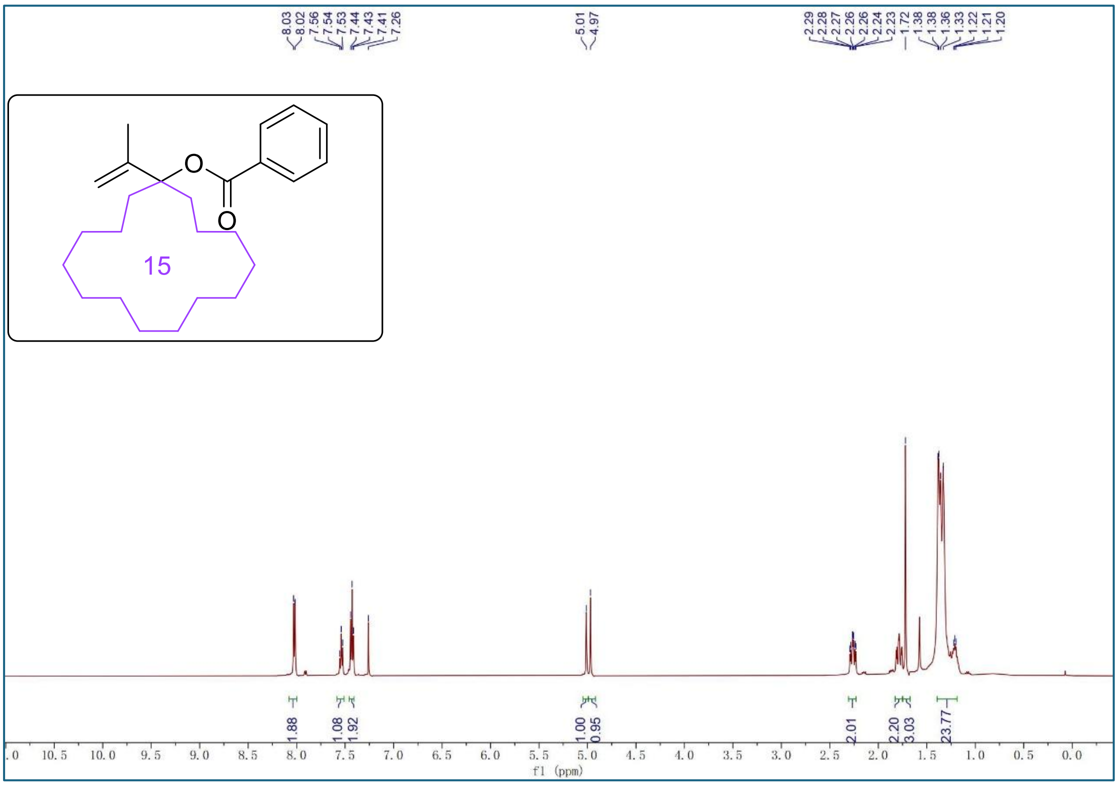

^{1}H NMR-spectrum (500 MHz, Chloroform-*d*) of **1d**

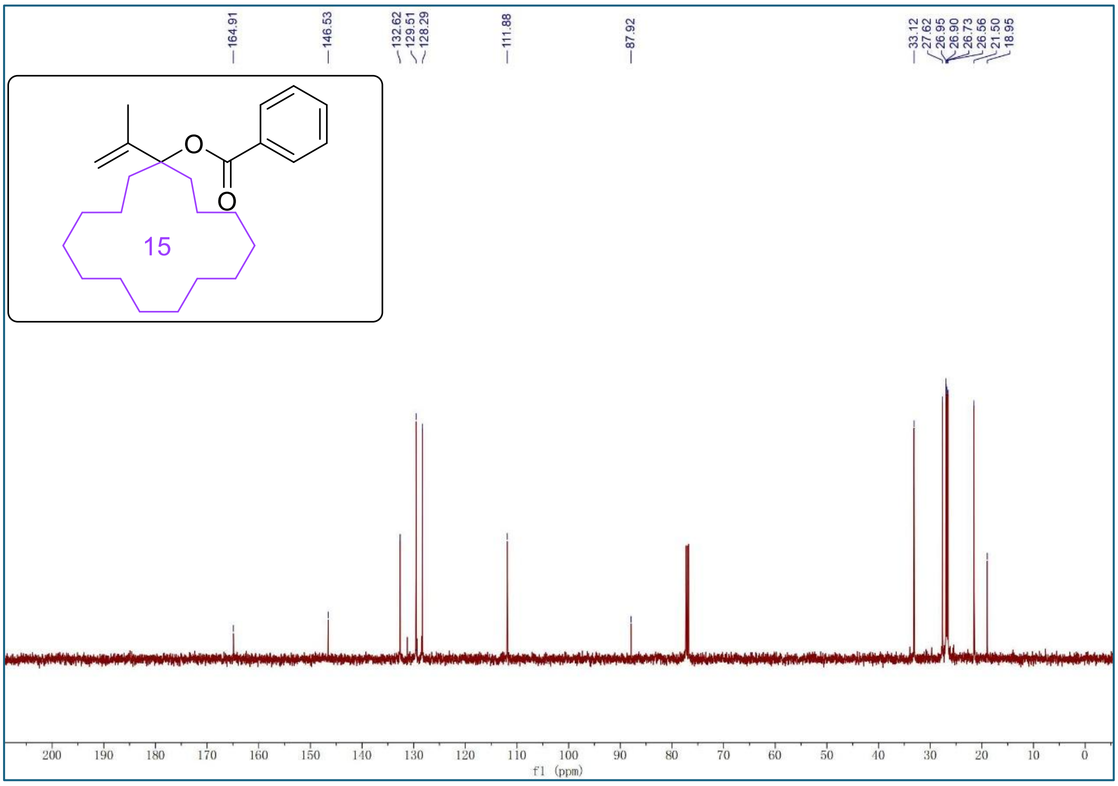

^{13}C NMR-spectrum (126 MHz, Chloroform-*d*) of **1d**

4,4-dimethyl-1-(prop-1-en-2-yl)cyclohexyl benzoate (1e)

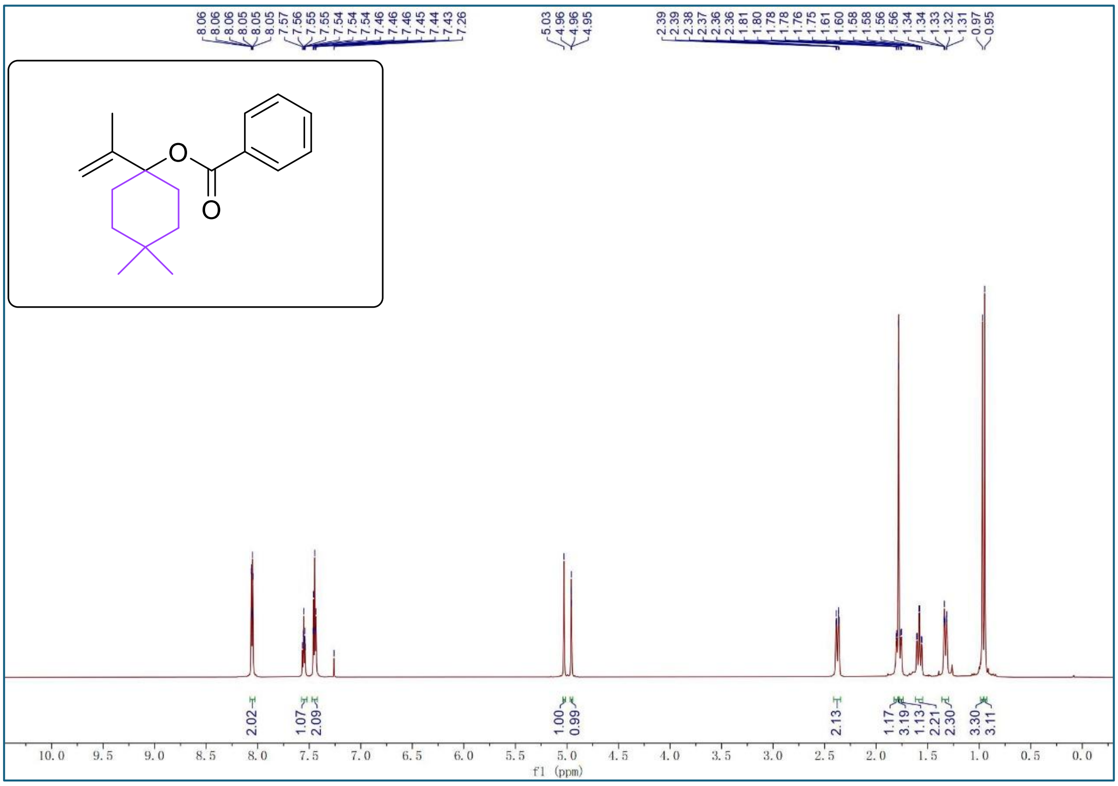

^{1}H NMR-spectrum (600 MHz, Chloroform-*d*) of **1e**

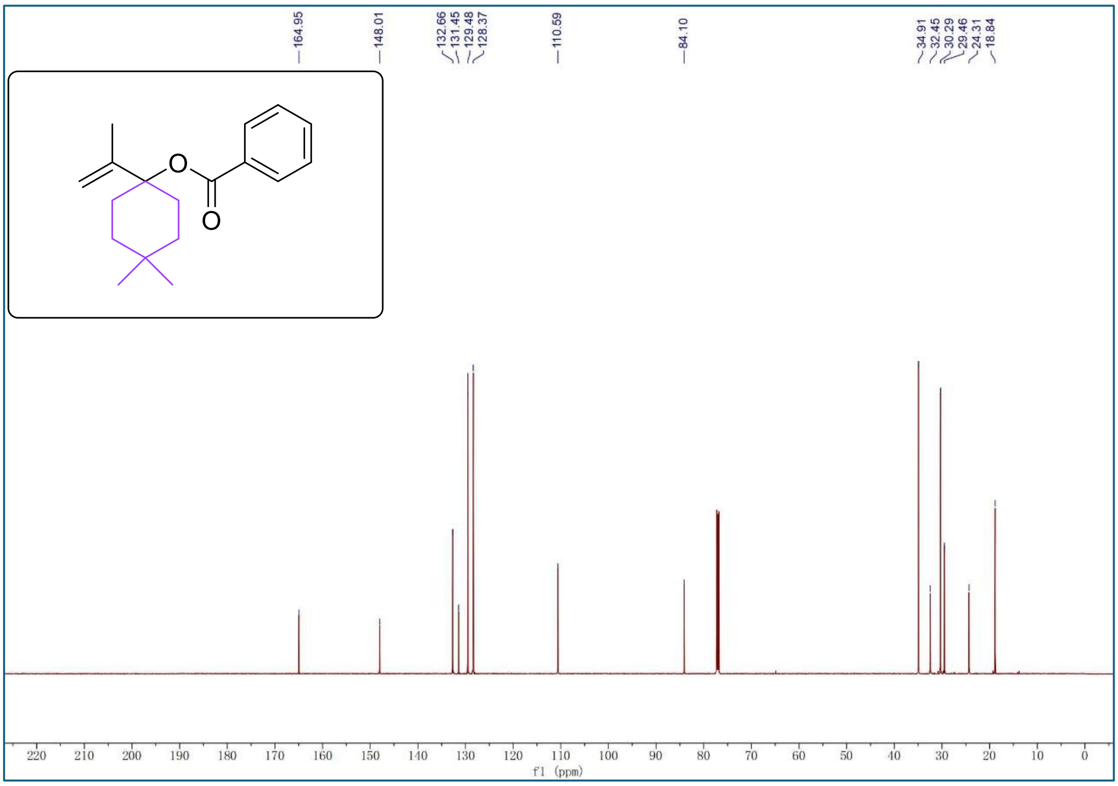

^{13}C NMR-spectrum (151 MHz, Chloroform-*d*) of **1e**

4-(1-phenylvinyl)tetrahydro-2H-pyran-4-yl benzoate (1f)

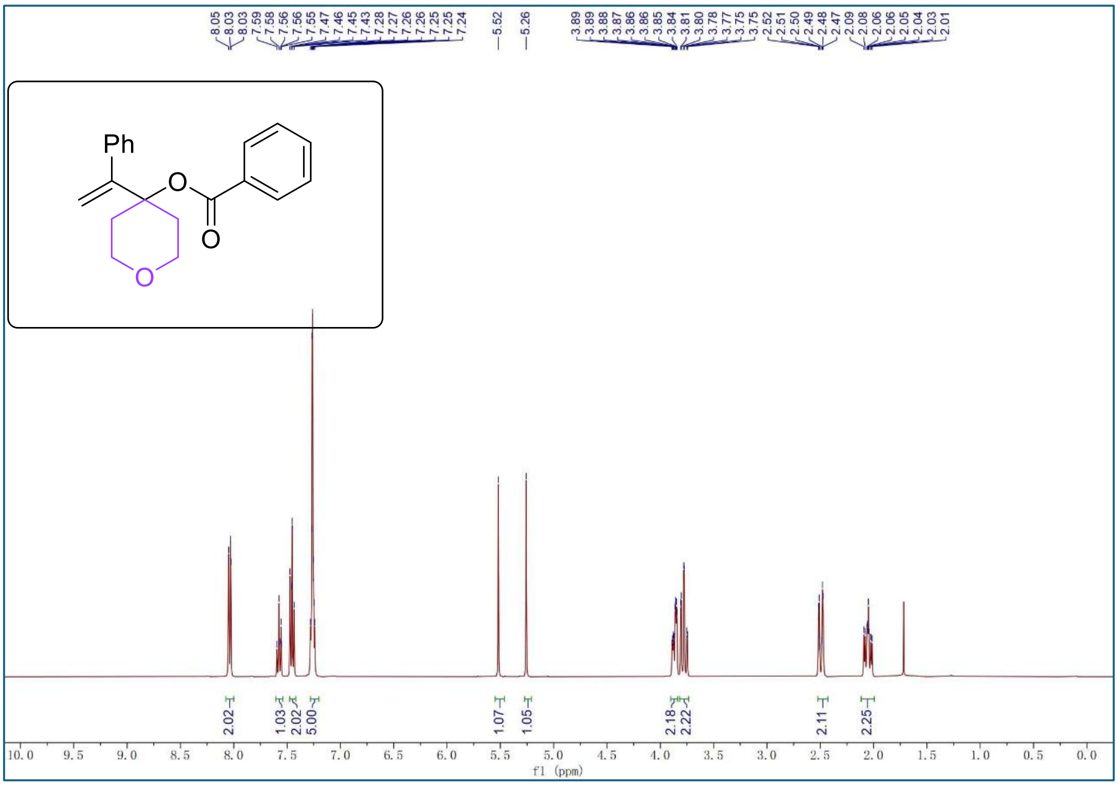

^{1}H NMR-spectrum (400 MHz, Chloroform-*d*) of **1f**

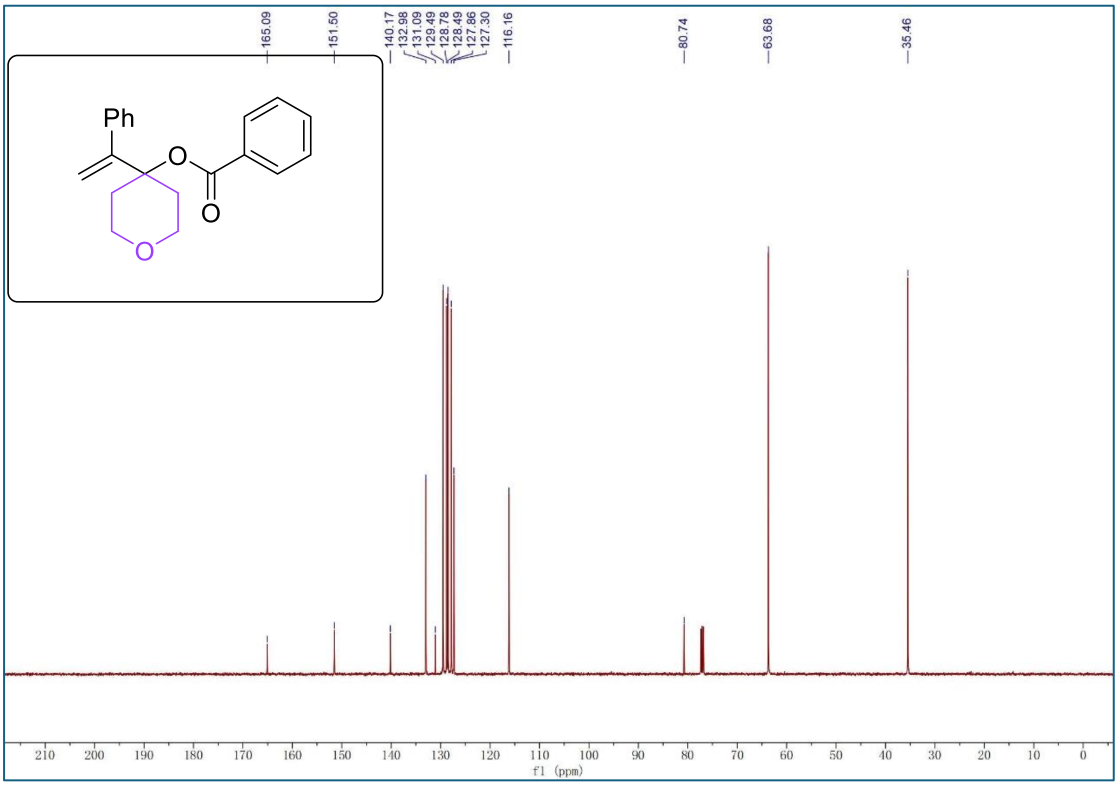

^{13}C NMR-spectrum (126 MHz, Chloroform-*d*) of **1f**

1-(1-phenylvinyl)cycloheptyl benzoate (1g)

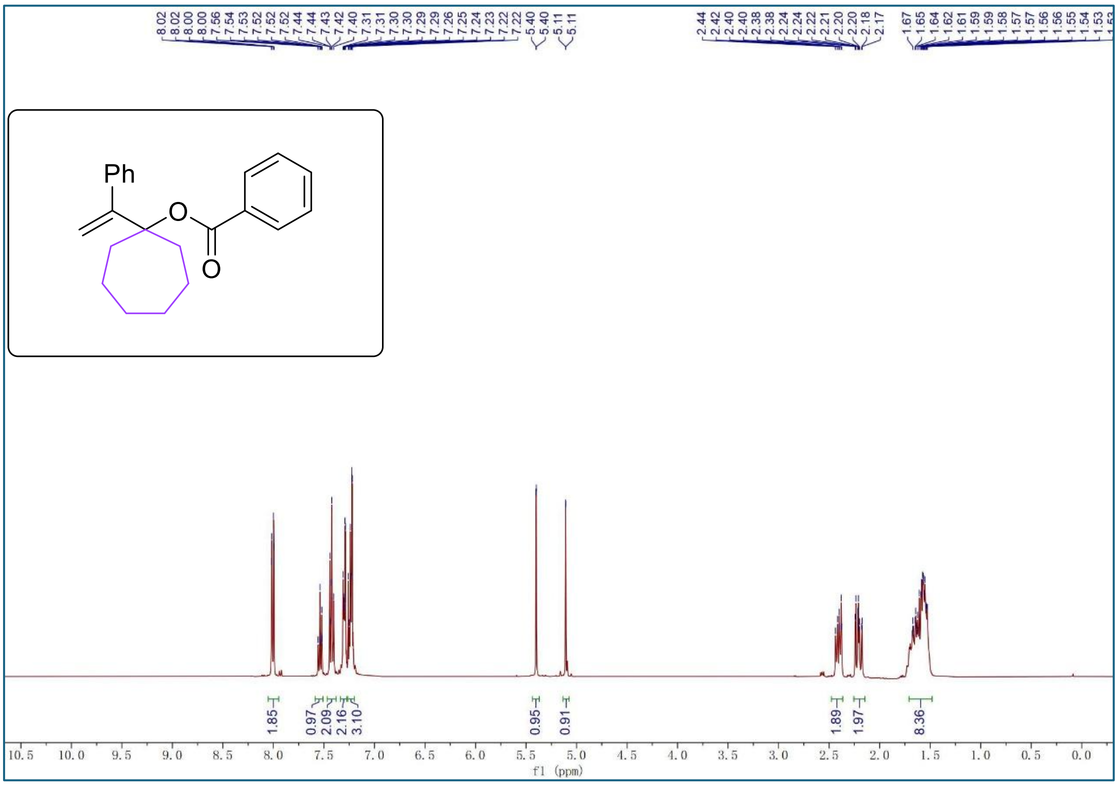

^{1}H NMR-spectrum (400 MHz, Chloroform-*d*) of **1g**

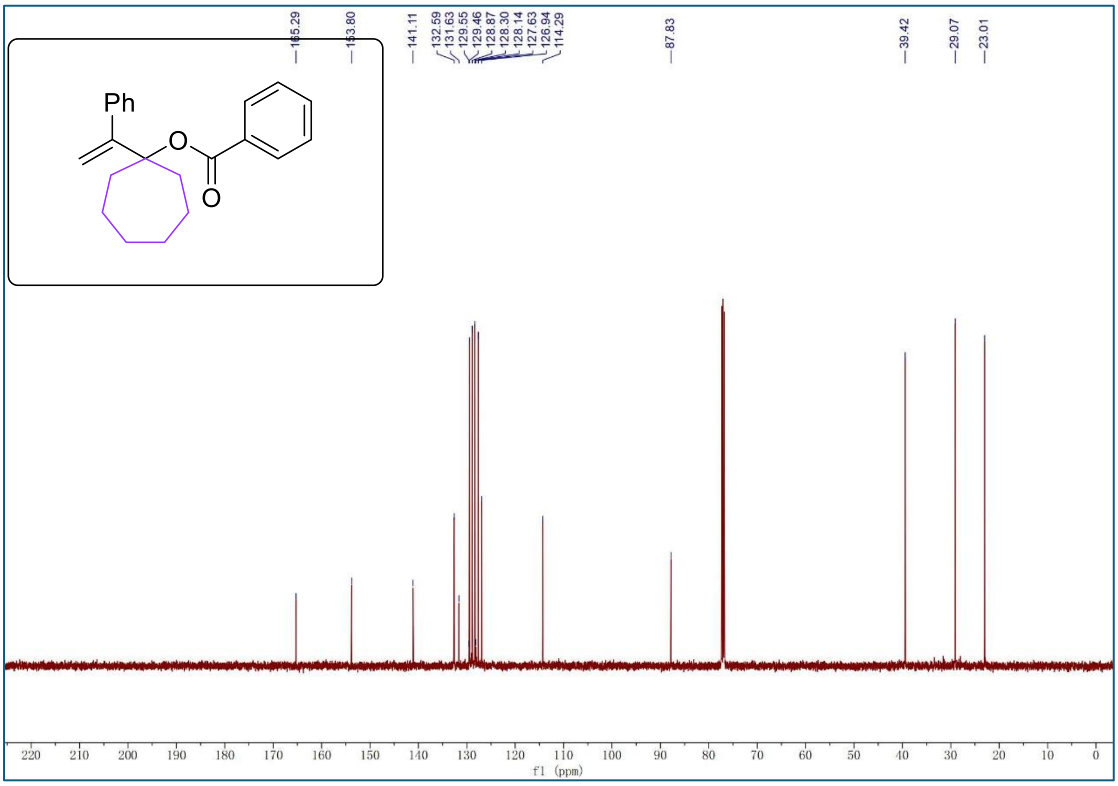

^{13}C NMR-spectrum (126 MHz, Chloroform-*d*) of **1g**

1-vinylcyclobutyl benzoate (1h)

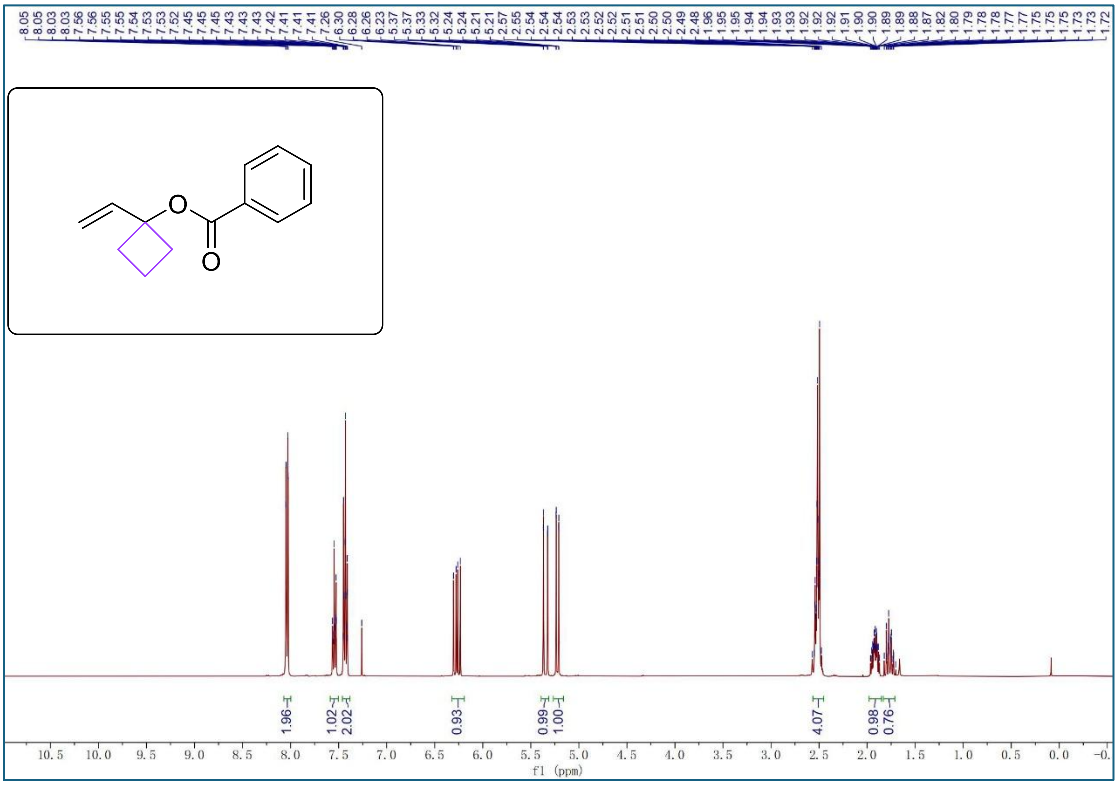

^1H NMR-spectrum (400 MHz, Chloroform-*d*) of **1h**

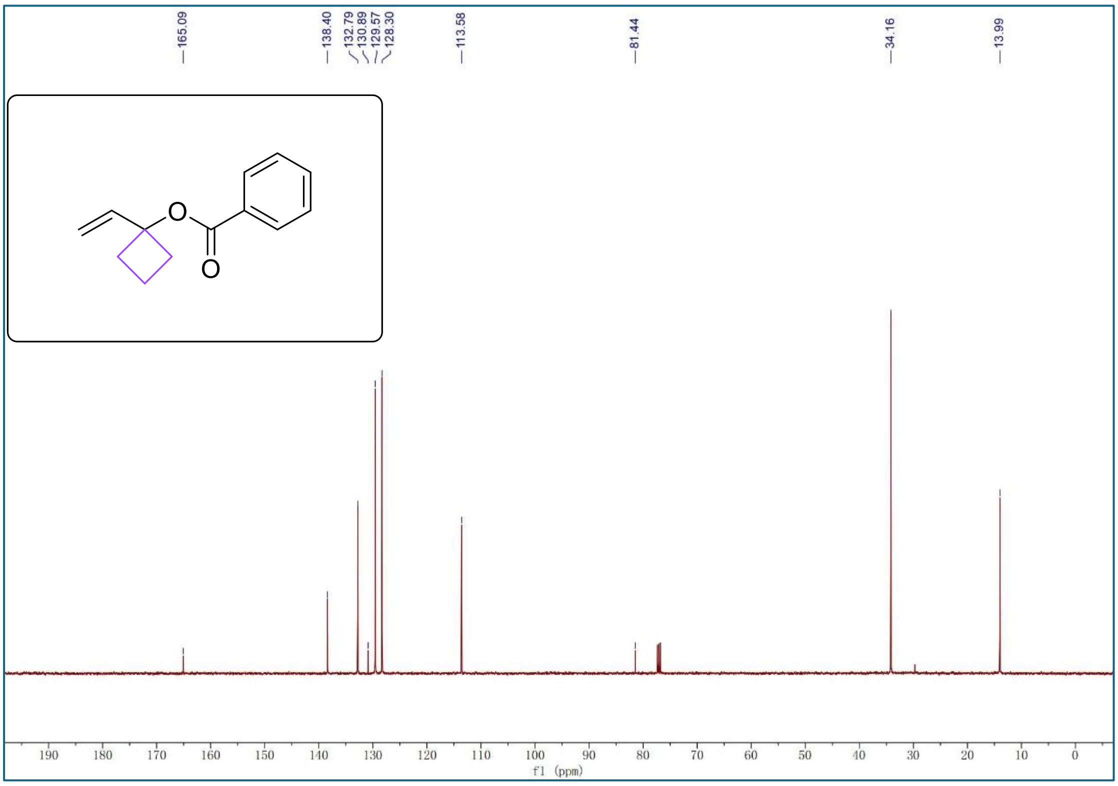

^{13}C NMR-spectrum (126 MHz, Chloroform-*d*) of **1h**

1-vinylcyclohexyl benzoate (1i)

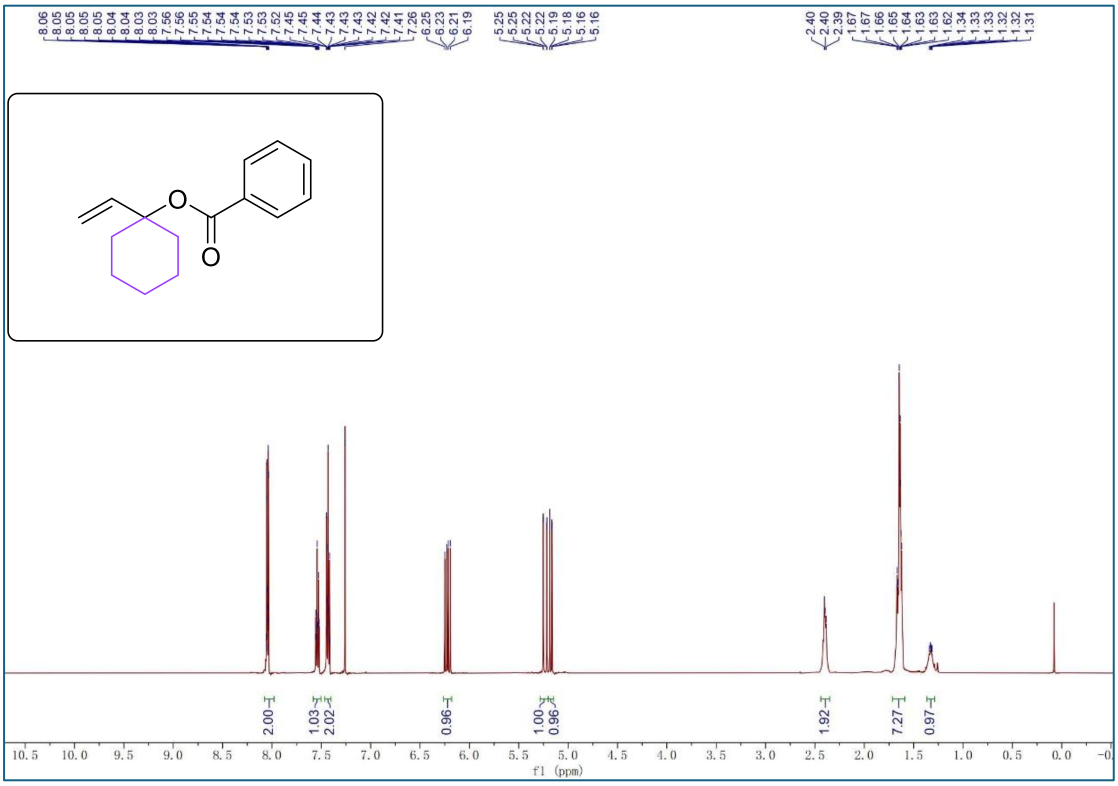

^{1}H NMR-spectrum (500 MHz, Chloroform-*d*) of **1i**

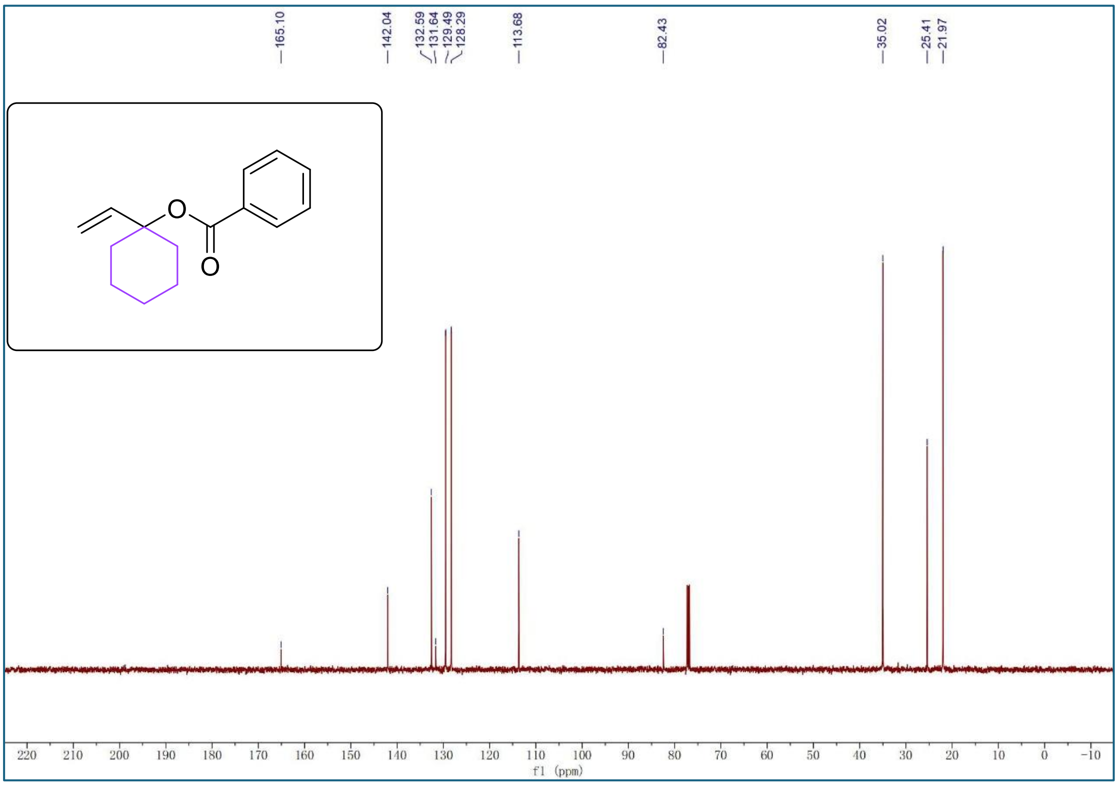

^{13}C NMR-spectrum (126 MHz, Chloroform-*d*) of **1i**

1-vinylcyclododecyl benzoate (1j)

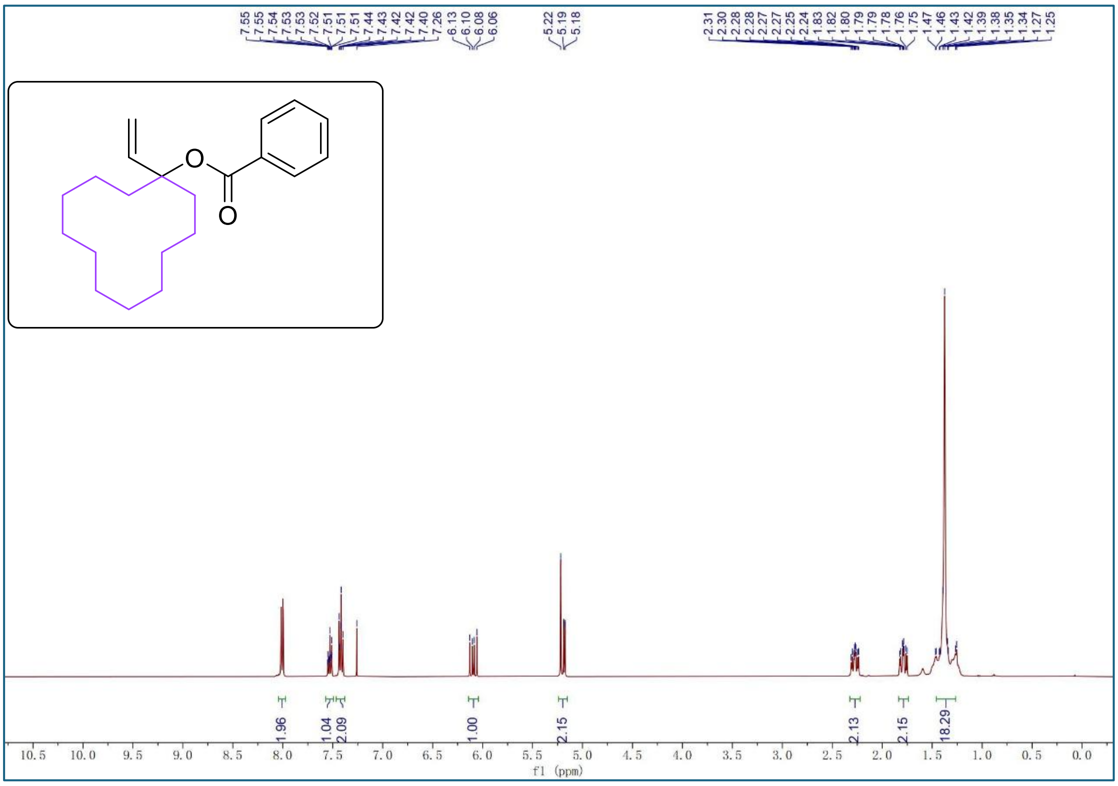

^{1}H NMR-spectrum (400 MHz, Chloroform-*d*) of **1j**

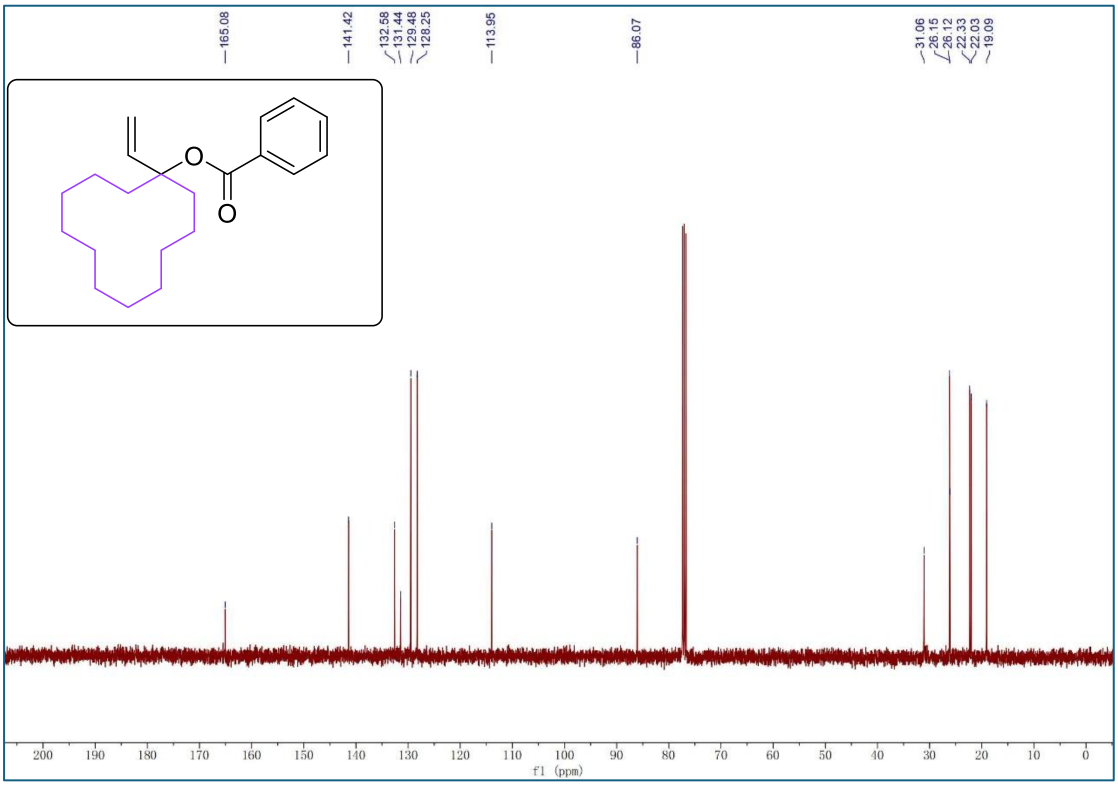

^{13}C NMR-spectrum (101 MHz, Chloroform-*d*) of **1j**

1-vinylcyclopentadecyl benzoate (1k)

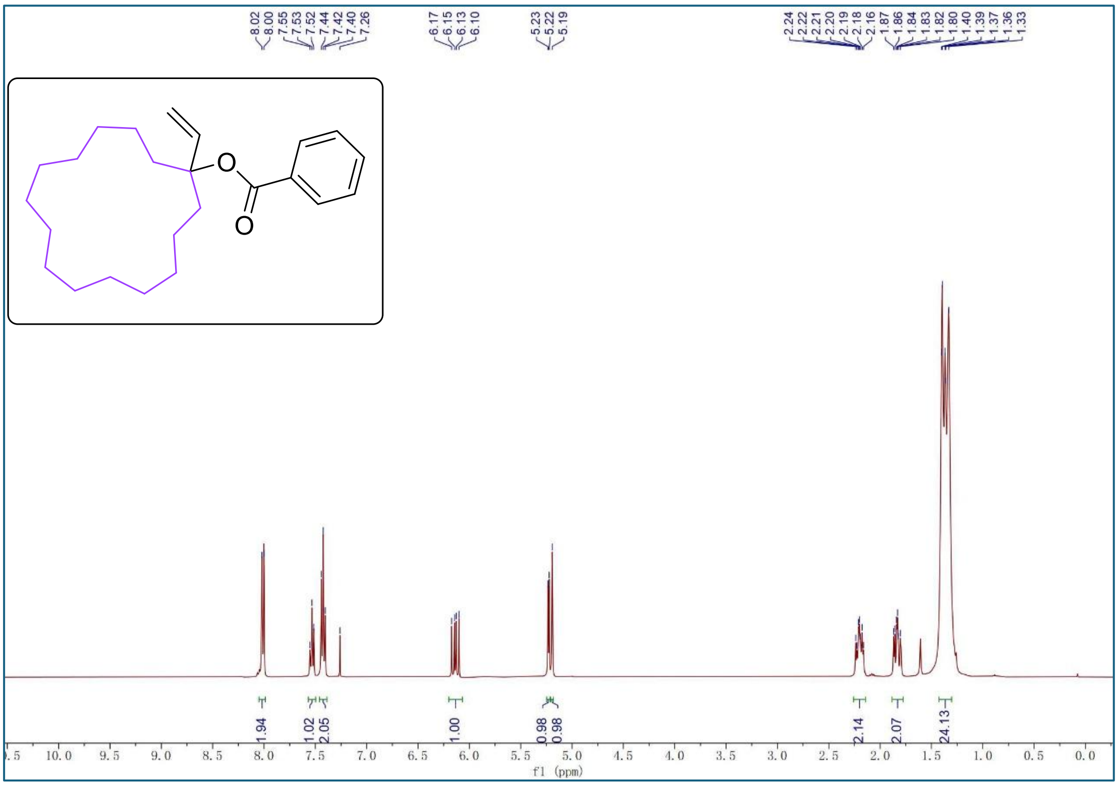

^{1}H NMR-spectrum (400 MHz, Chloroform-*d*) of **1k**

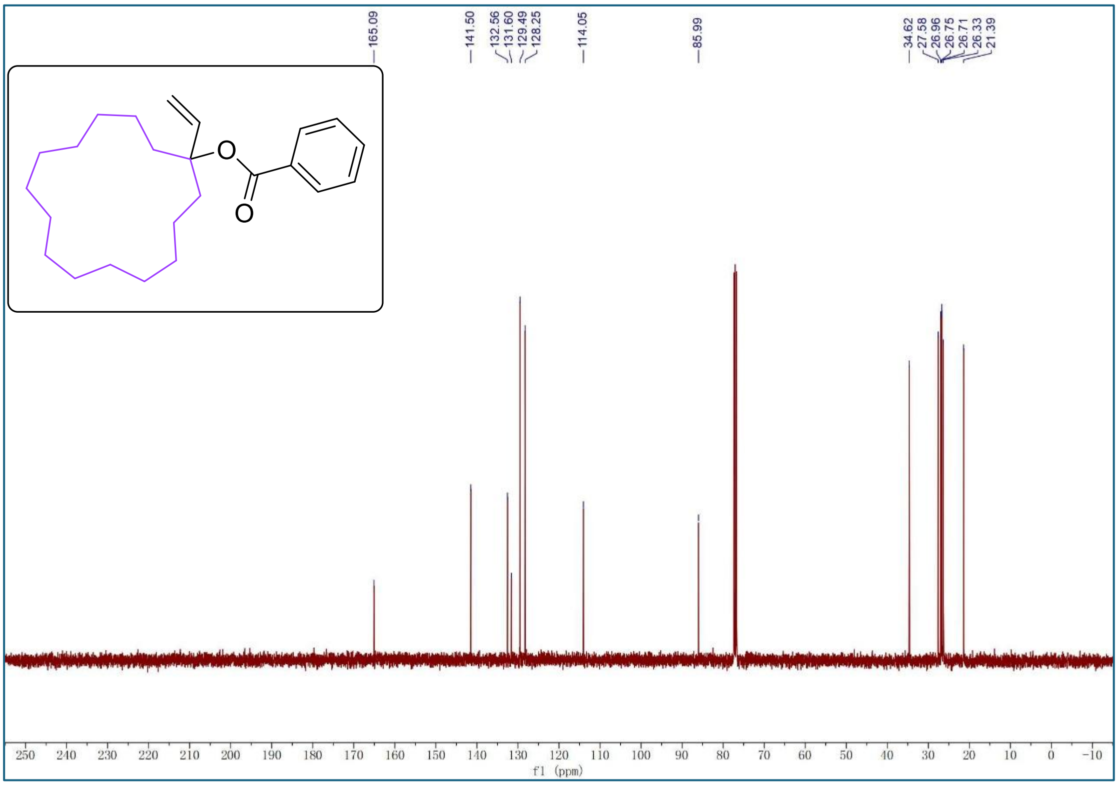

^{13}C NMR-spectrum (101 MHz, Chloroform-*d*) of **1k**

2,6-dimethyl-1-vinylcyclohexyl benzoate (1l)

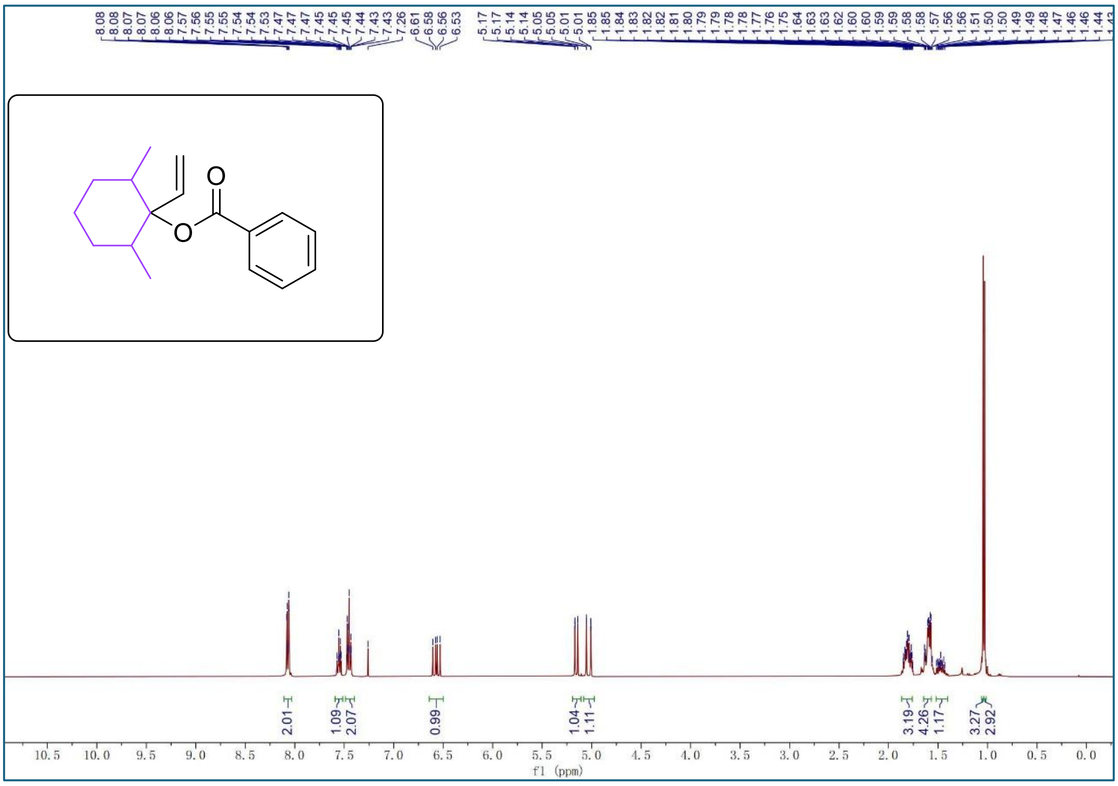

^{1}H NMR-spectrum (400 MHz, Chloroform-*d*) of **1l**

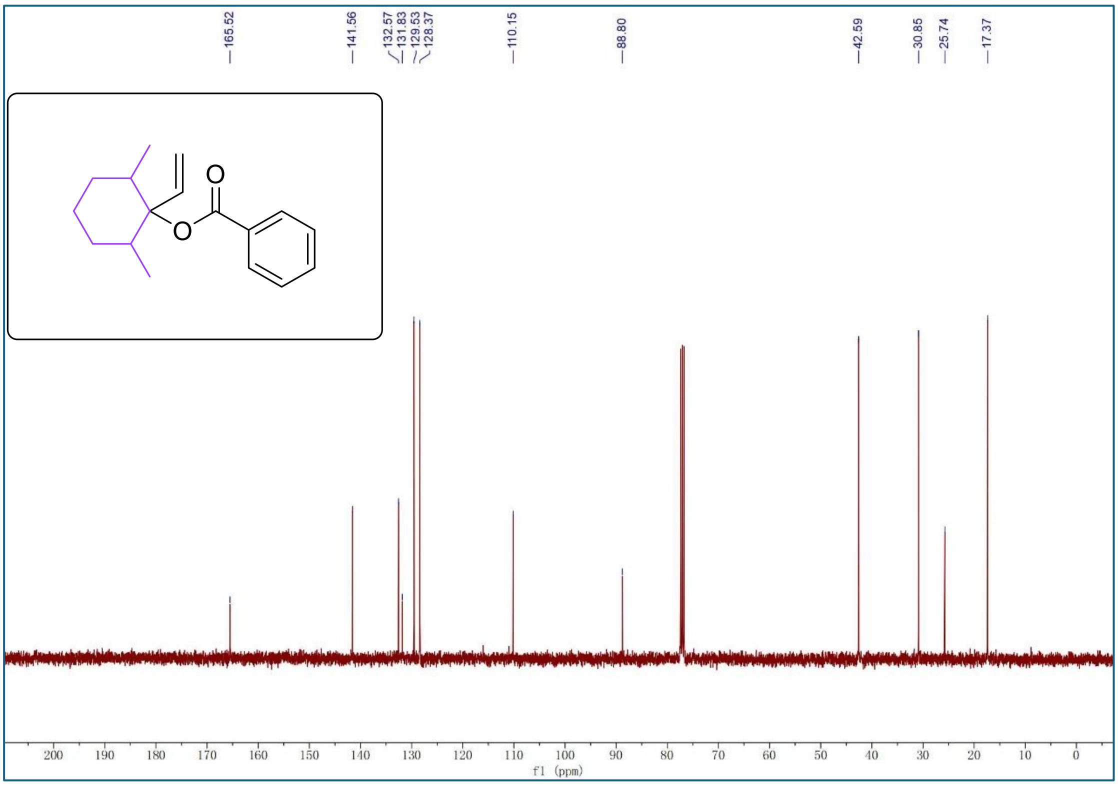

^{13}C NMR-spectrum (101 MHz, Chloroform-*d*) of **1l**

4,4-difluoro-1-vinylcyclohexyl benzoate (1m)

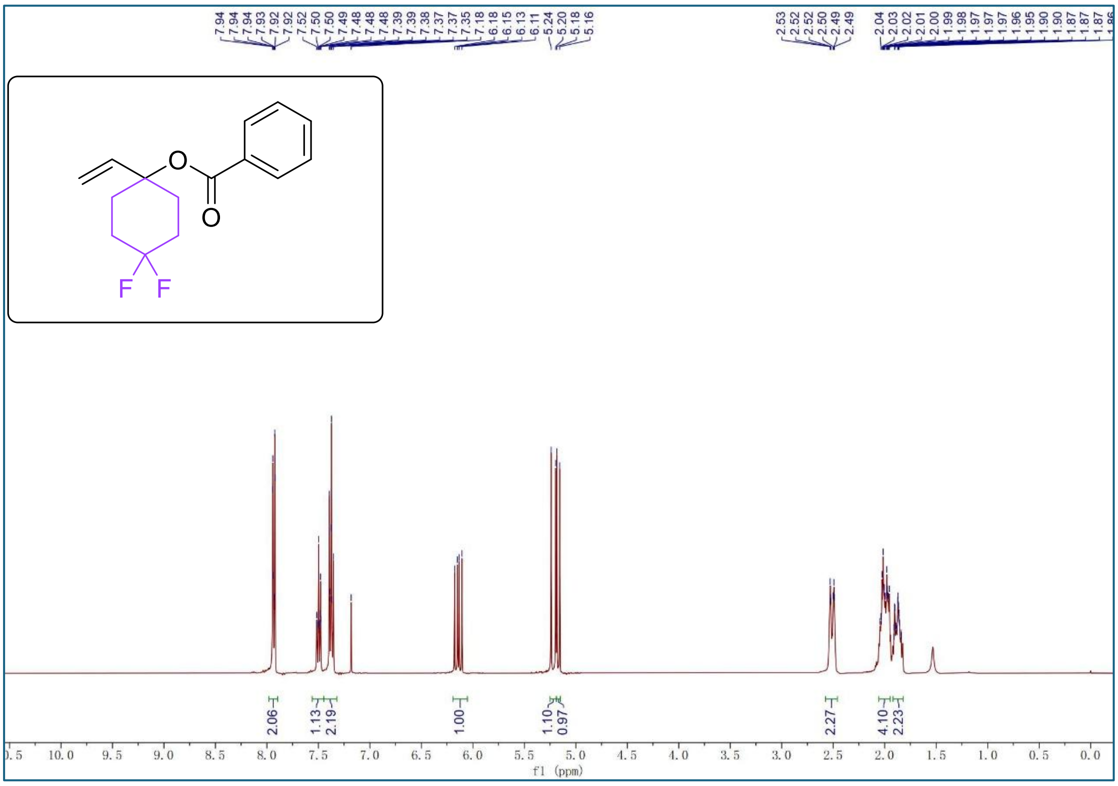

^{1}H NMR-spectrum (400 MHz, Chloroform-*d*) of **1m**

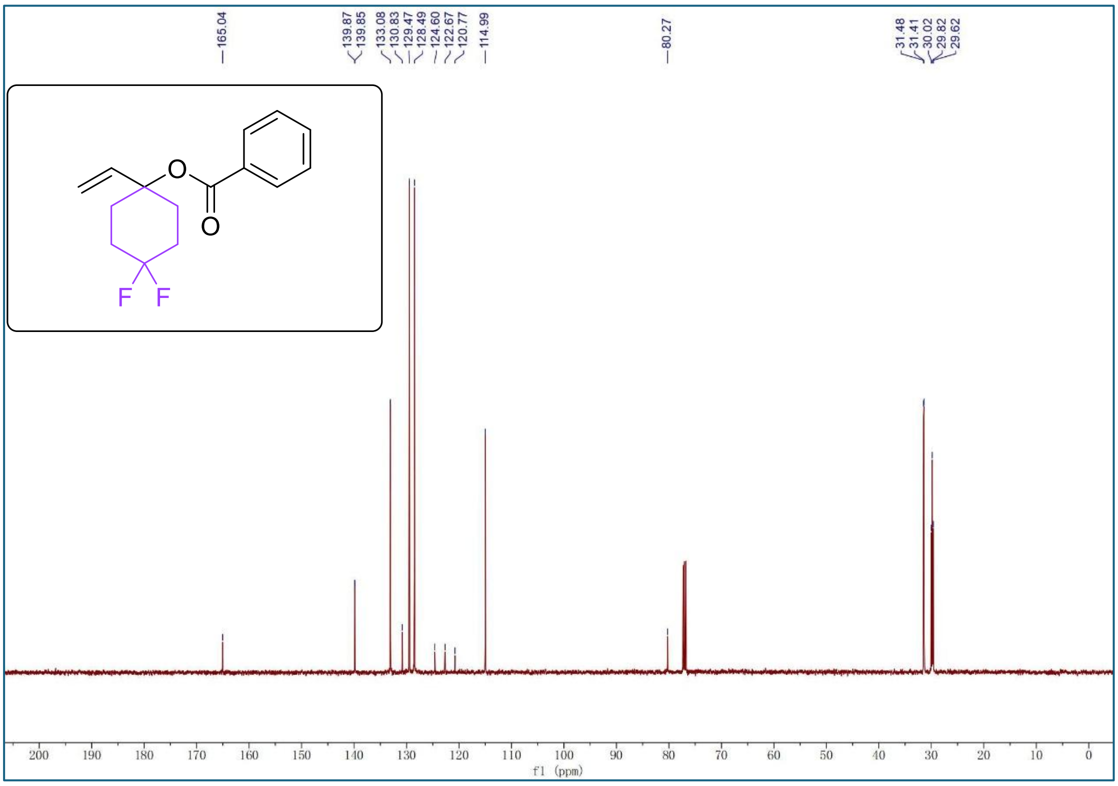

^{13}C NMR-spectrum (126 MHz, Chloroform-*d*) of **1m**

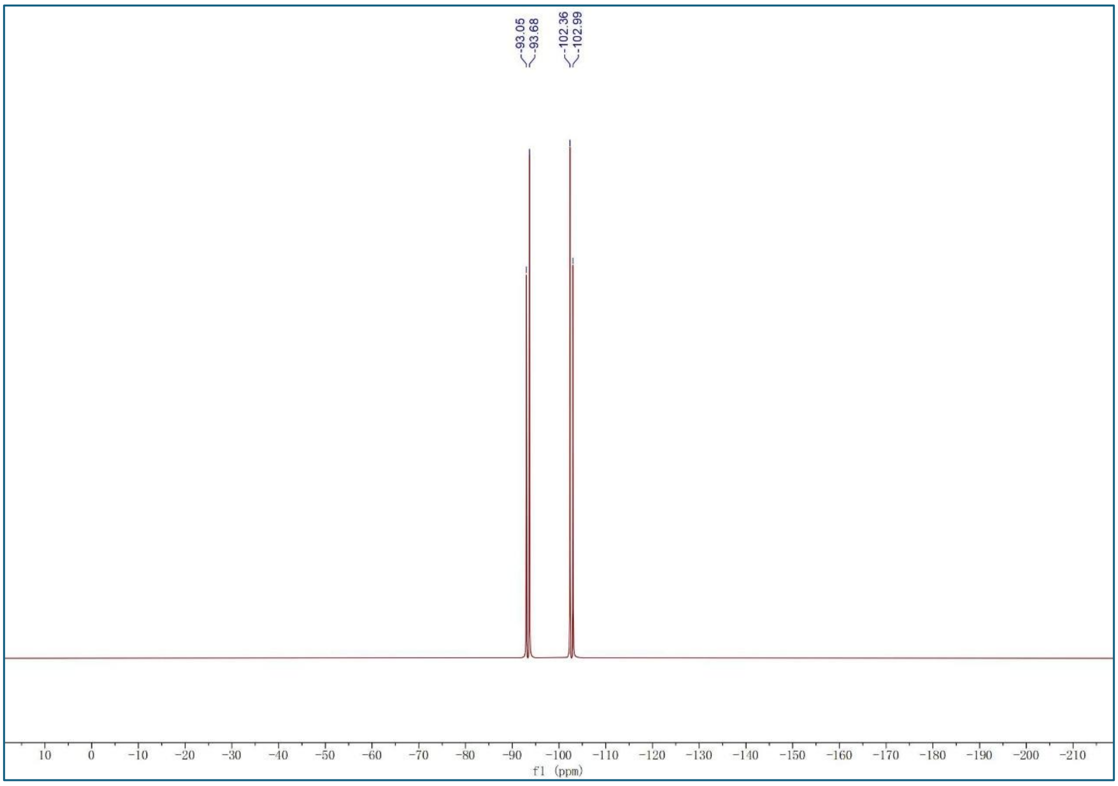

^{19}F NMR-spectrum (377 MHz, Chloroform-*d*) of **1m**

7-vinyl-6,7,8,9-tetrahydro-5H-benzo[7]annulen-7-yl benzoate (1n)

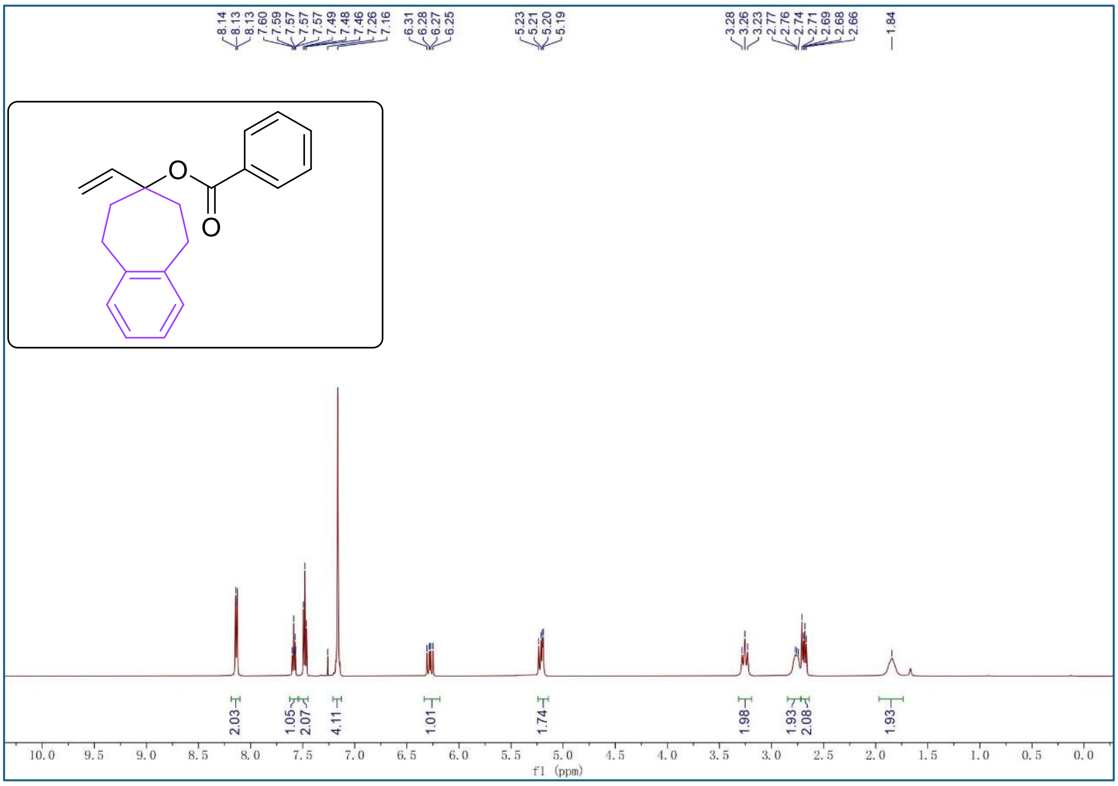

^{1}H NMR-spectrum (500 MHz, Chloroform-*d*) of **1n**

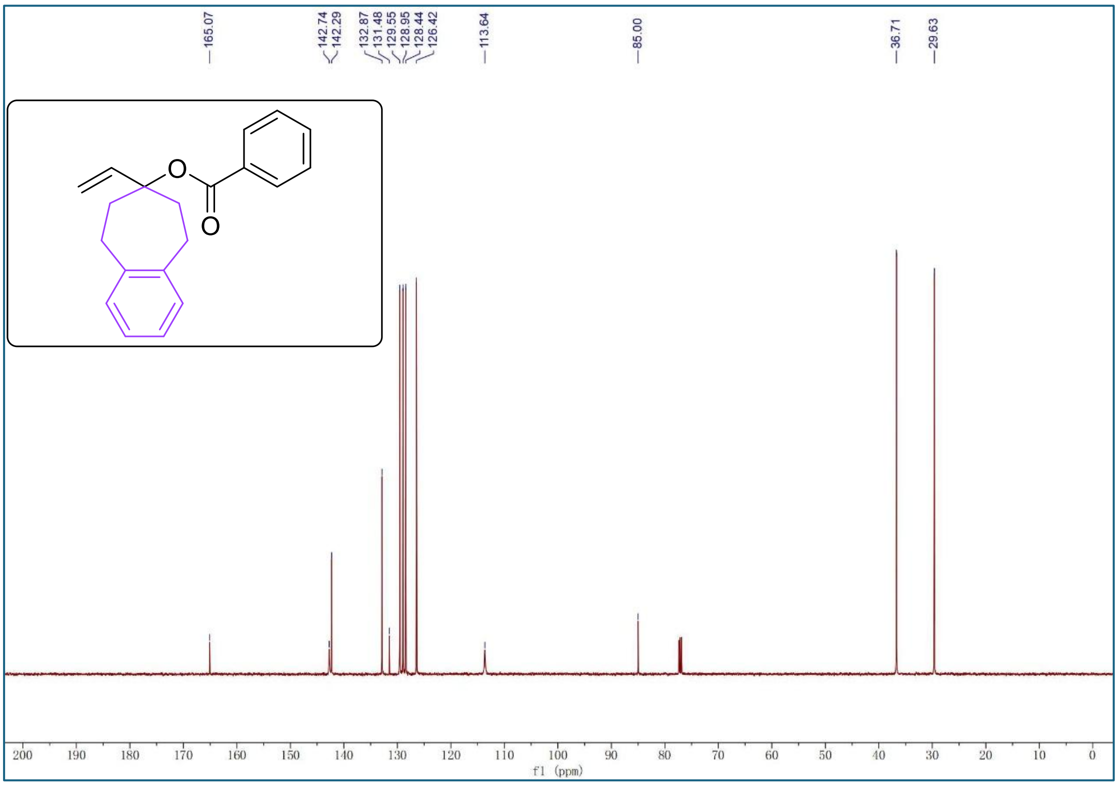

^{13}C NMR-spectrum (126 MHz, Chloroform-*d*) of **1n**

8-vinyl-1,4-dioxaspiro[4.5]decan-8-yl benzoate (1o)

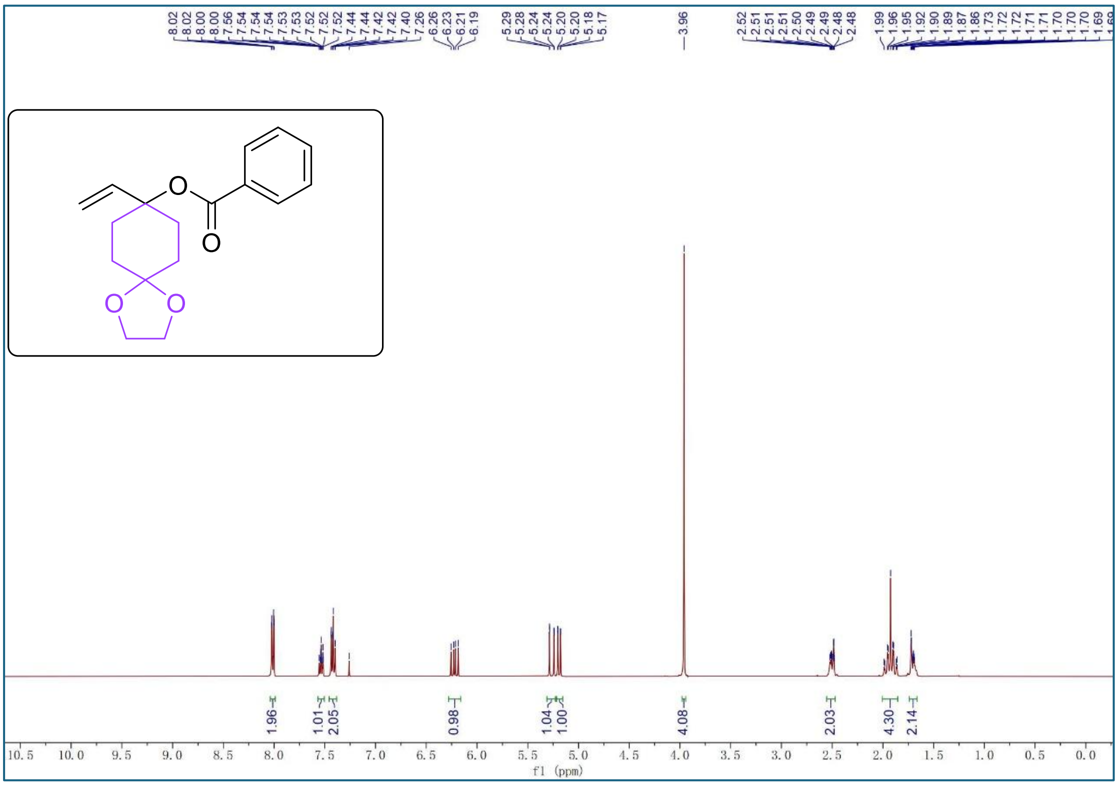

^{1}H NMR-spectrum (400 MHz, Chloroform-*d*) of **1o**

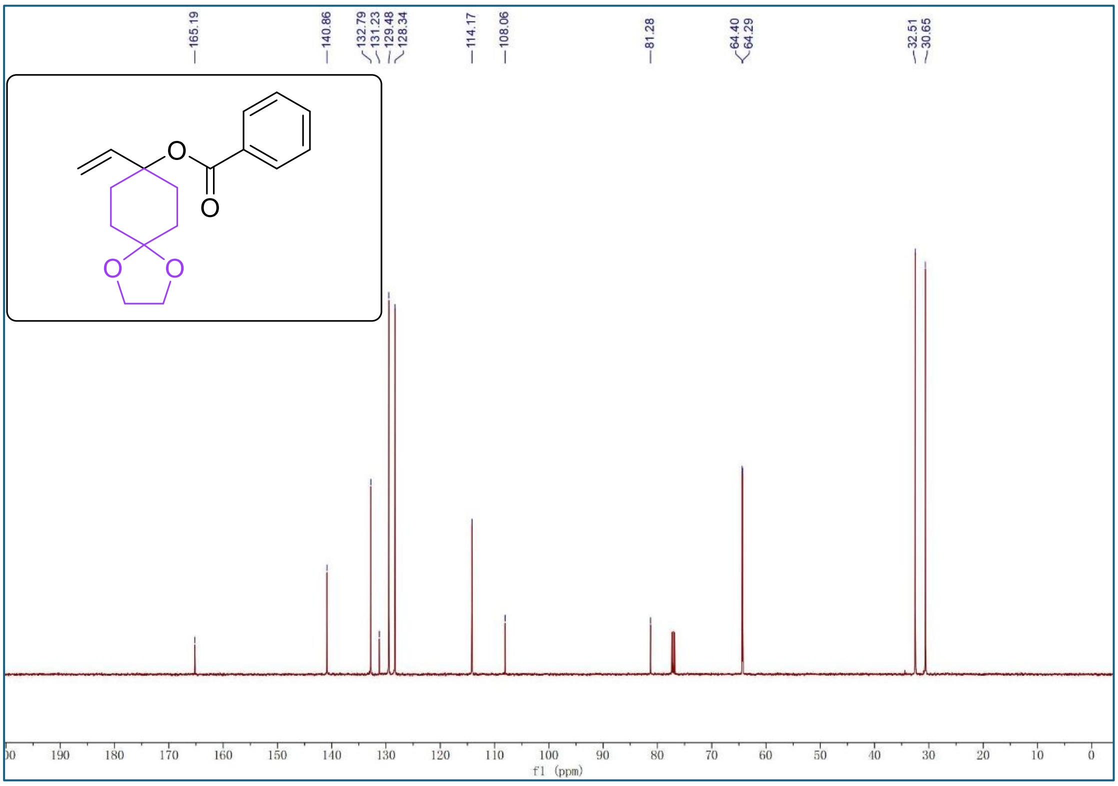

^{13}C NMR-spectrum (126 MHz, Chloroform-*d*) of **1o**

3,3-dimethyl-9-vinyl-1,5-dioxaspiro[5.5]undecan-9-yl benzoate (1p)

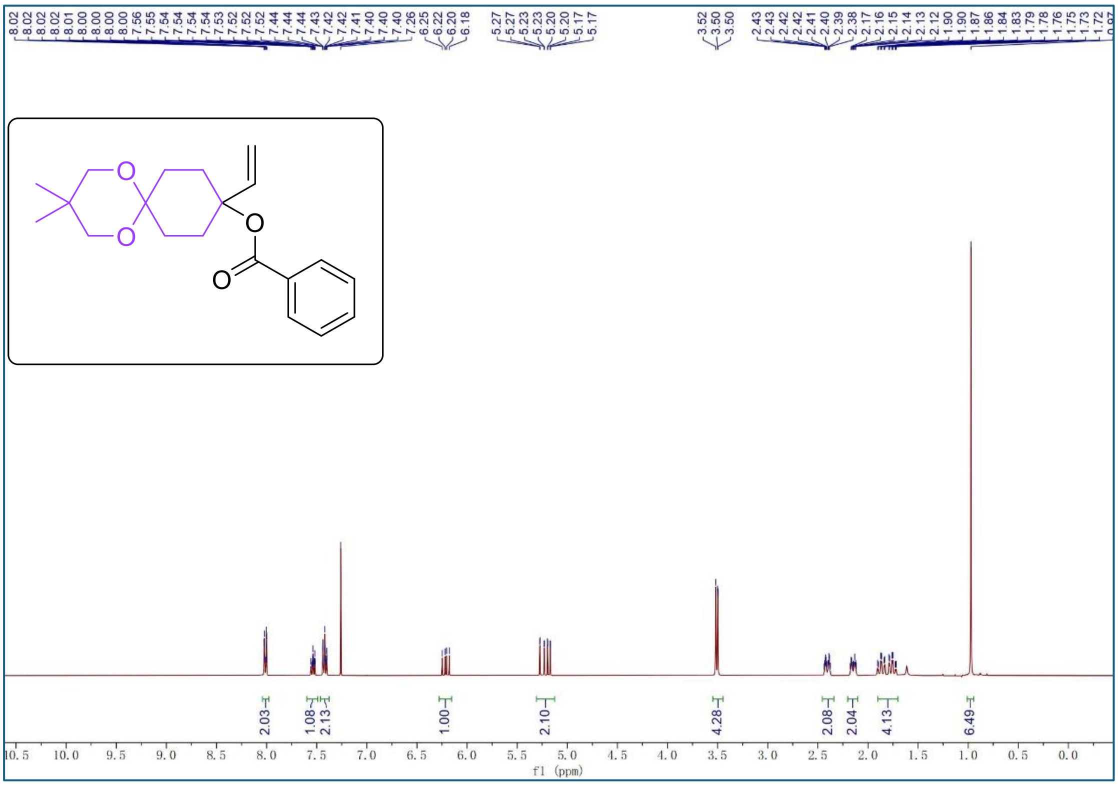

^{1}H NMR-spectrum (400 MHz, Chloroform-*d*) of **1p**

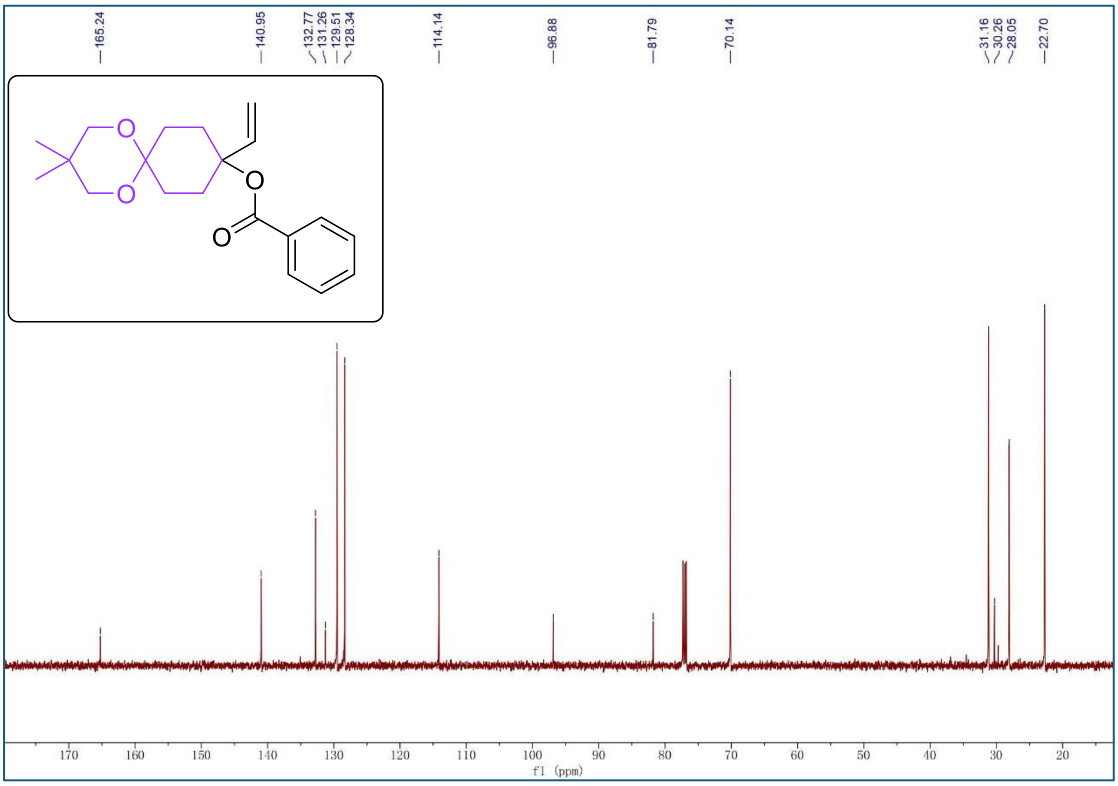

^{13}C NMR-spectrum (126 MHz, Chloroform-*d*) of **1p**

3-vinyloxetan-3-yl benzoate (1q)

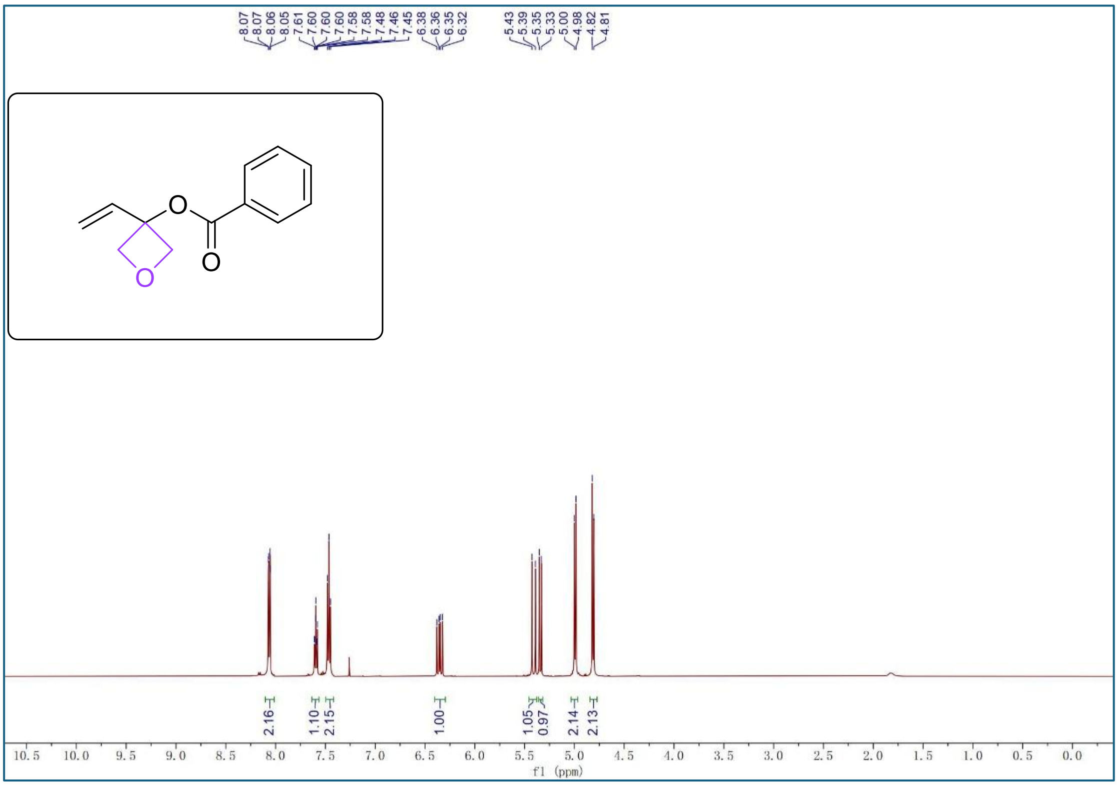

^{1}H NMR-spectrum (500 MHz, Chloroform-*d*) of **1q**

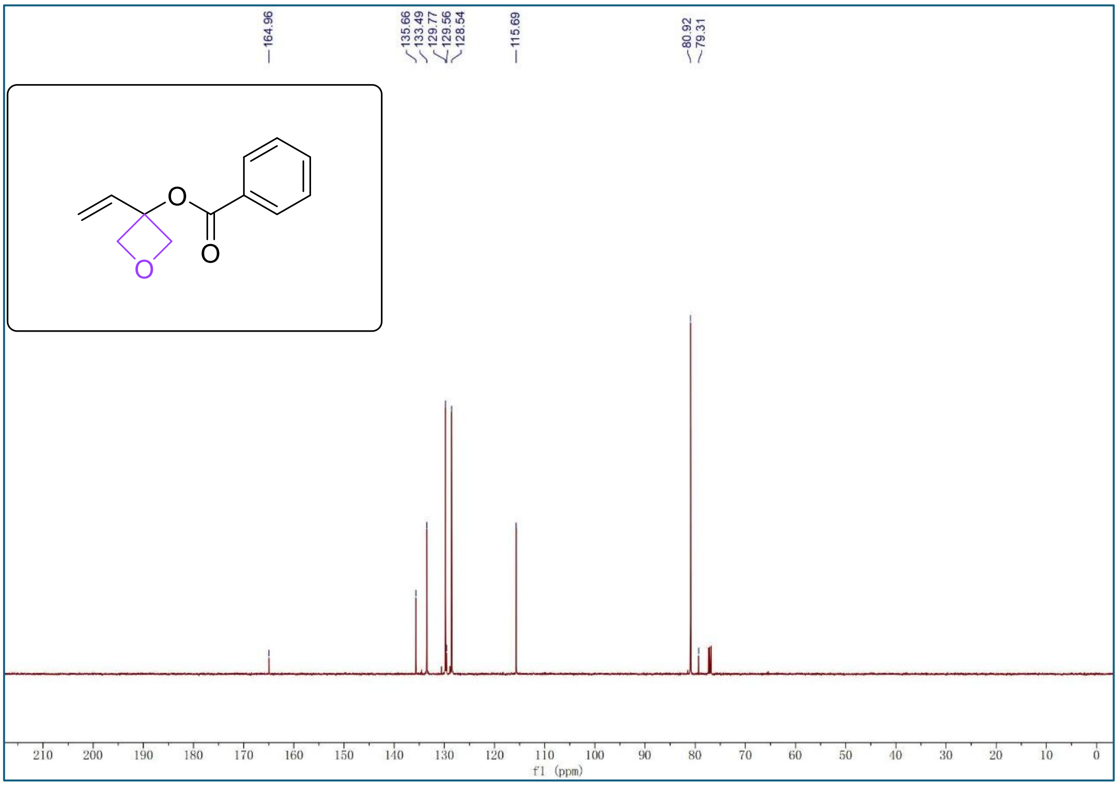

^{13}C NMR-spectrum (126 MHz, Chloroform-*d*) of **1q**

3-vinyltetrahydro-2H-pyran-3-yl benzoate (1r)

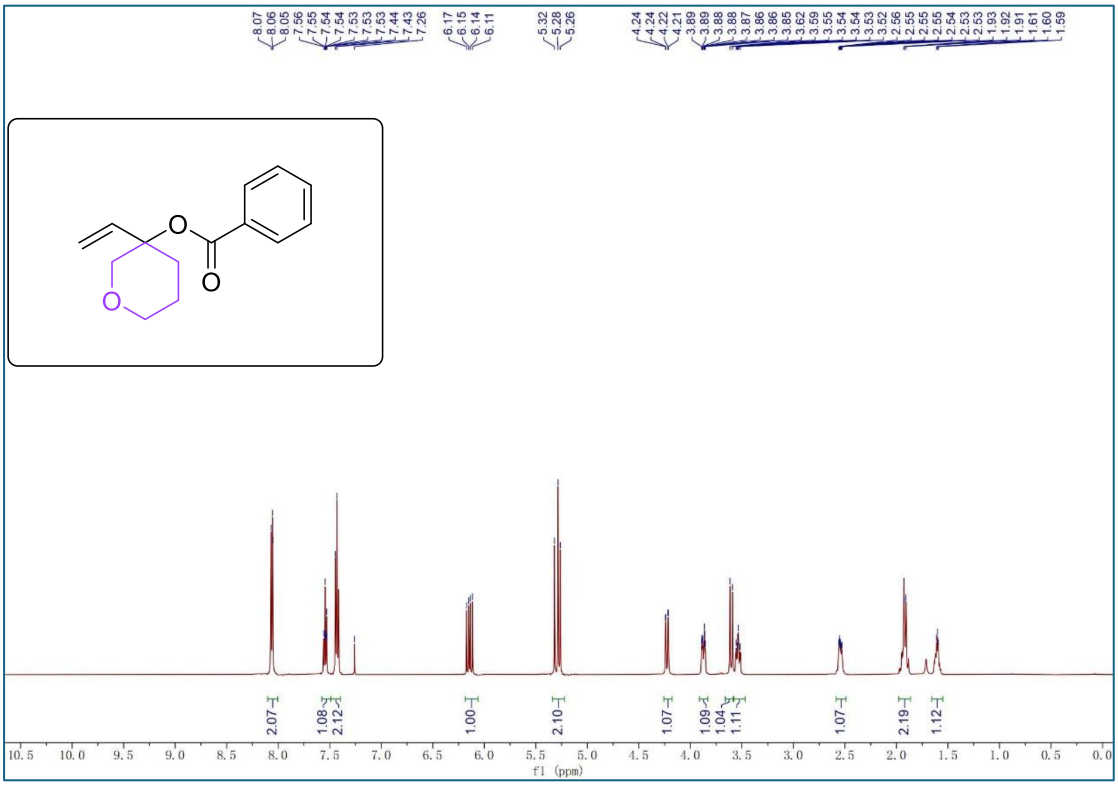

^{1}H NMR-spectrum (500 MHz, Chloroform-*d*) of **1r**

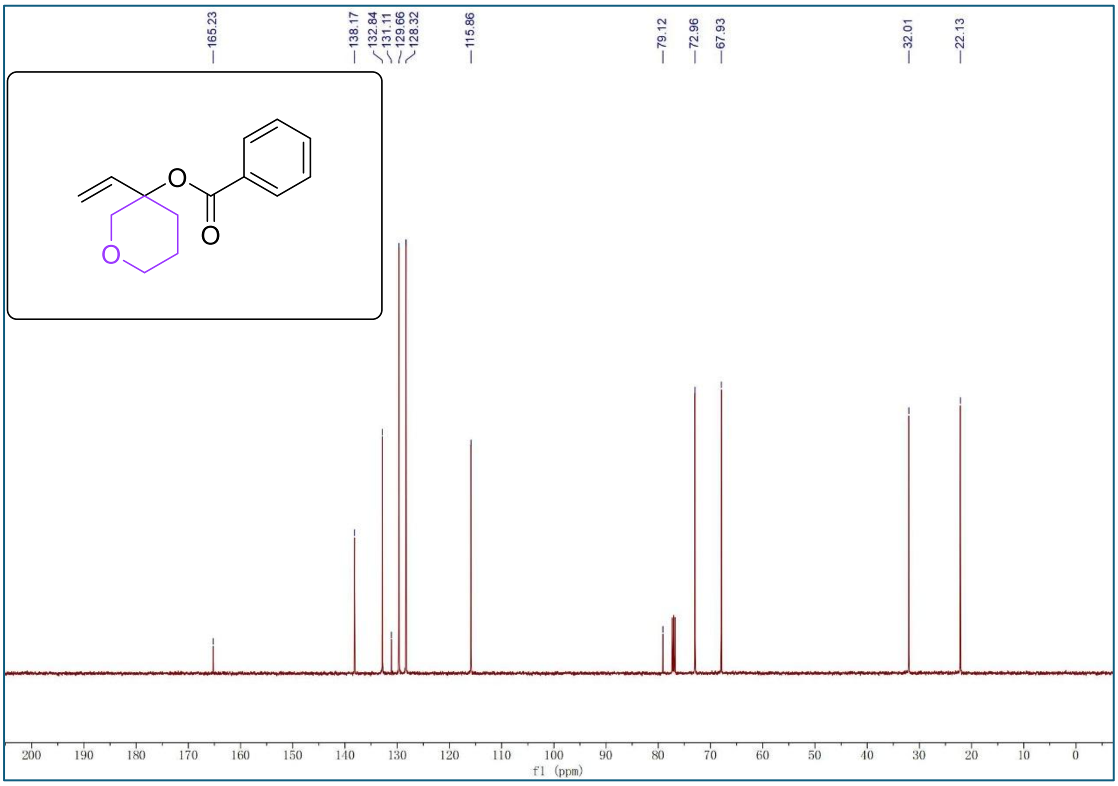

^{13}C NMR-spectrum (126 MHz, Chloroform-*d*) of **1r**

2,2-dimethyl-4-vinyltetrahydro-2H-pyran-4-yl benzoate (1s)

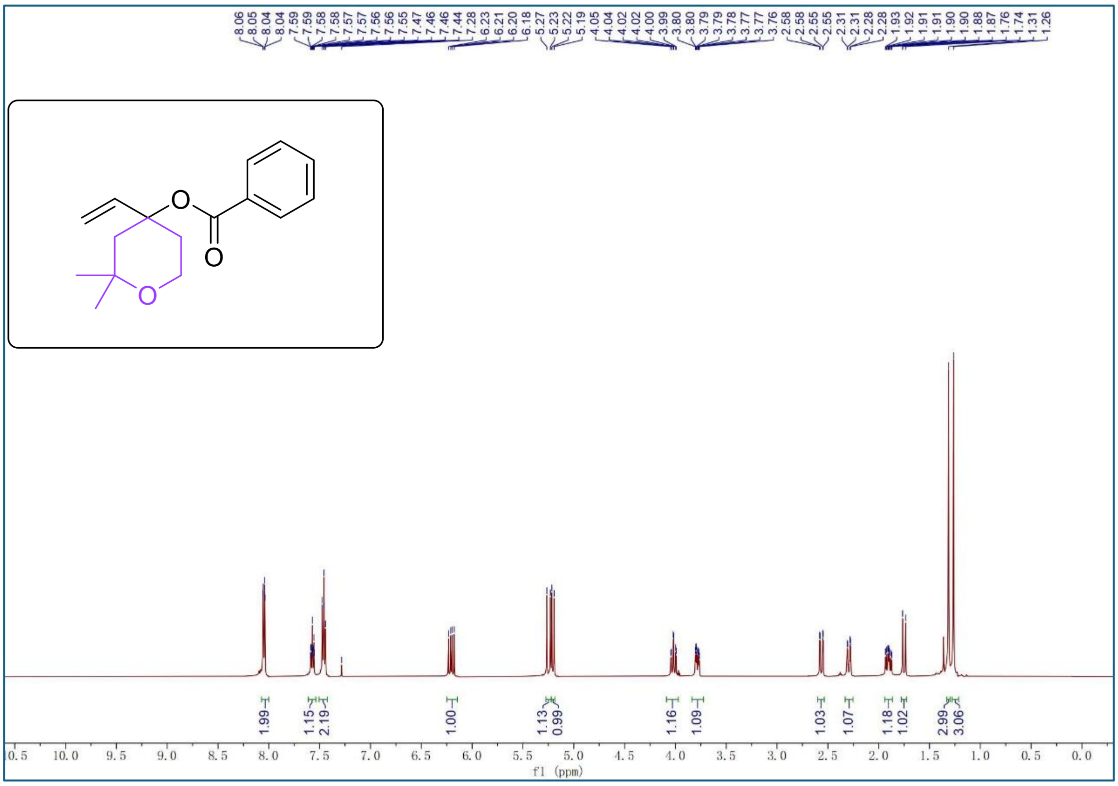

^{1}H NMR-spectrum (500 MHz, Chloroform-*d*) of **1s**

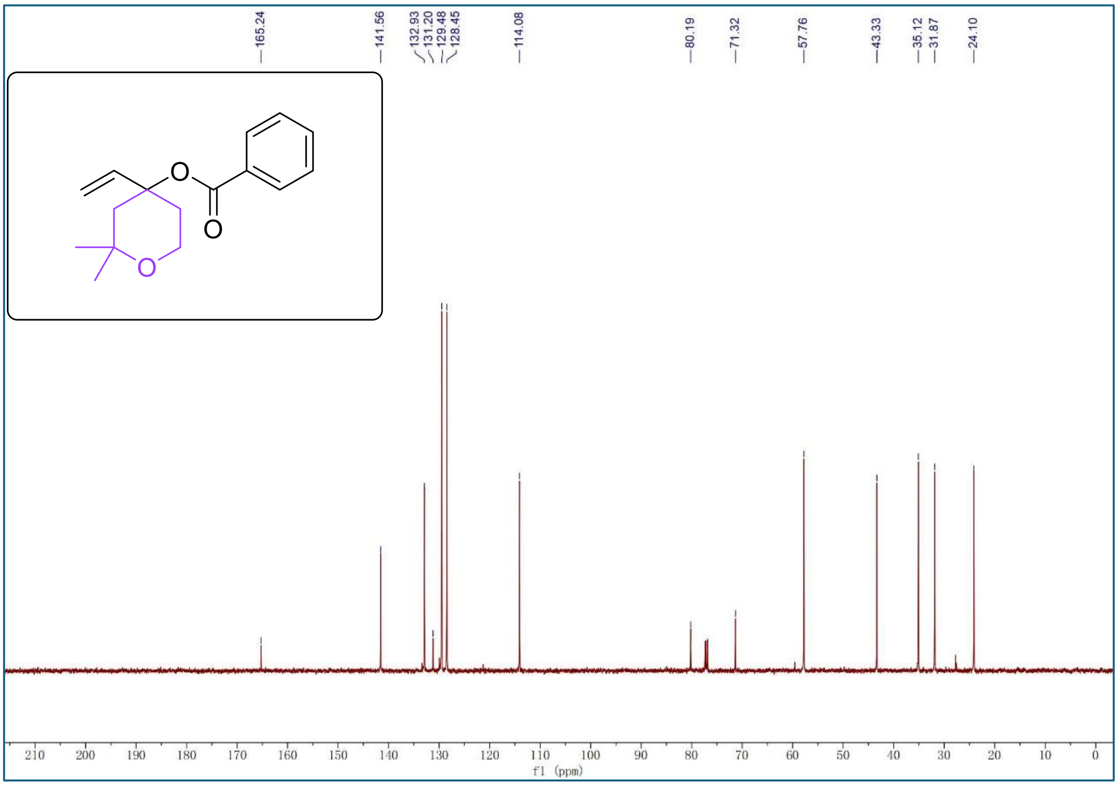

^{13}C NMR-spectrum (126 MHz, Chloroform-*d*) of **1s**

tert-butyl 4-(benzoyloxy)-4-vinylpiperidine-1-carboxylate (1t)

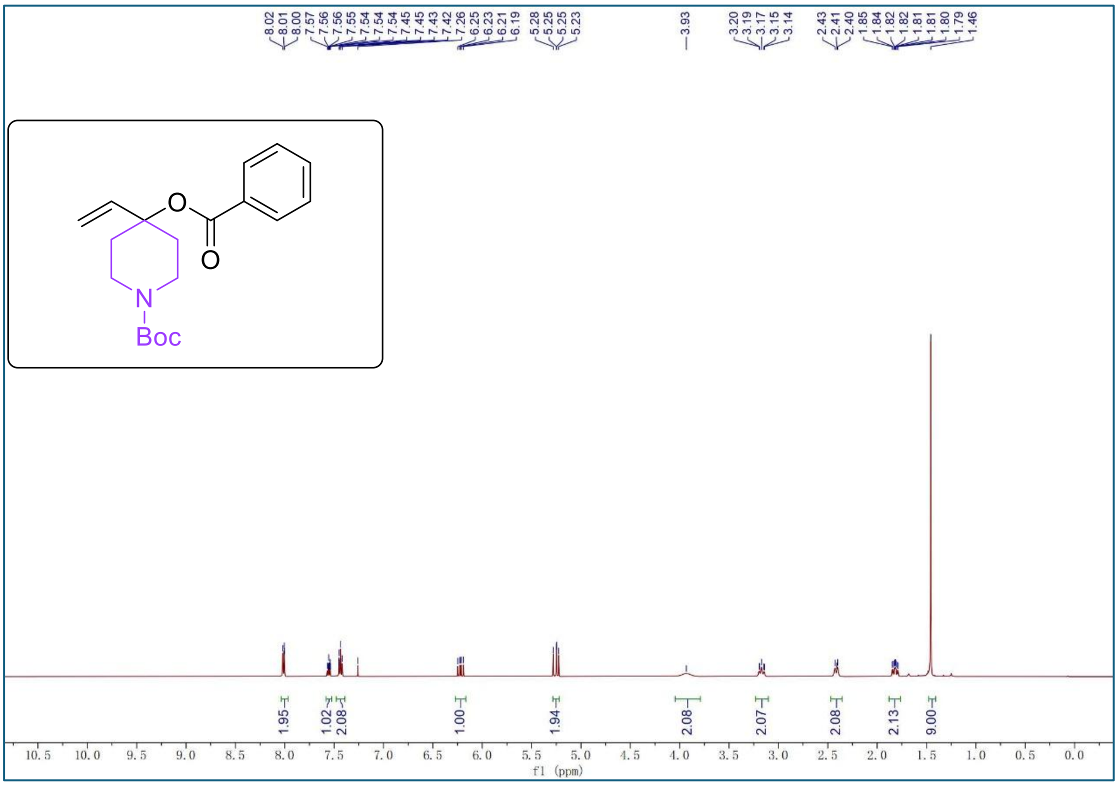

^{1}H NMR-spectrum (500 MHz, Chloroform-*d*) of **1t**

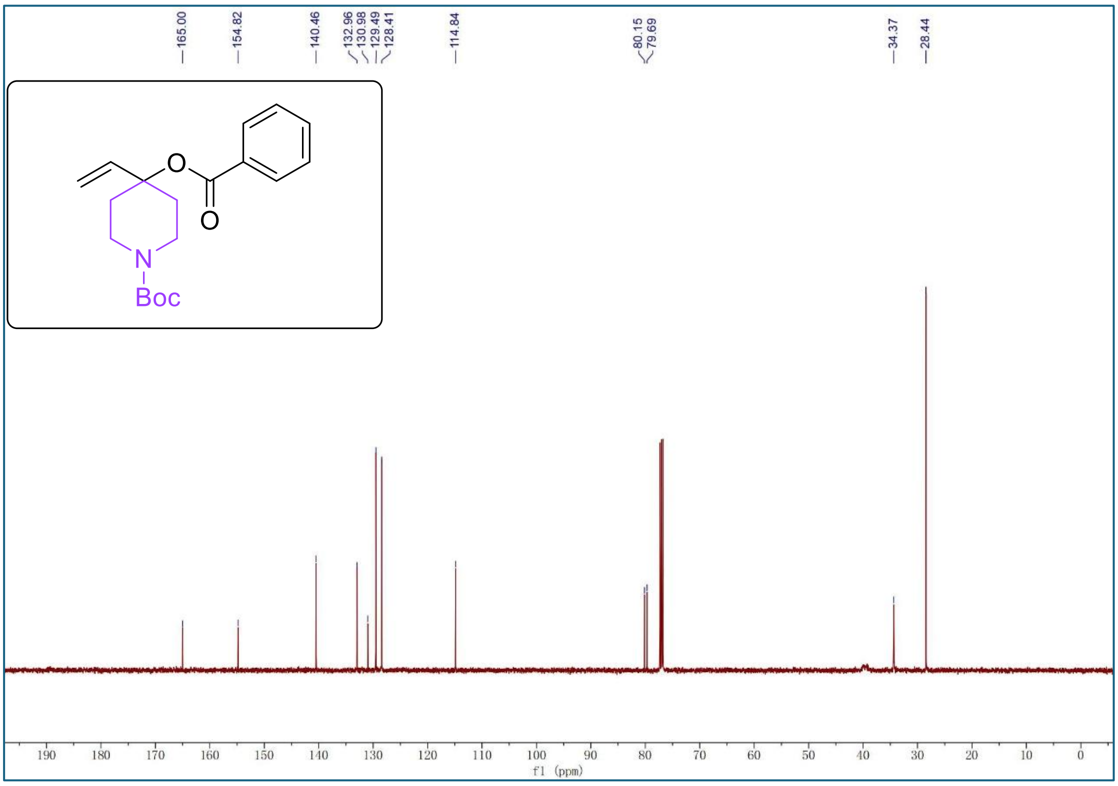

^{13}C NMR-spectrum (126 MHz, Chloroform-*d*) of **1t**

1,1-dioxido-4-vinyltetrahydro-2H-thiopyran-4-yl benzoate (1u)

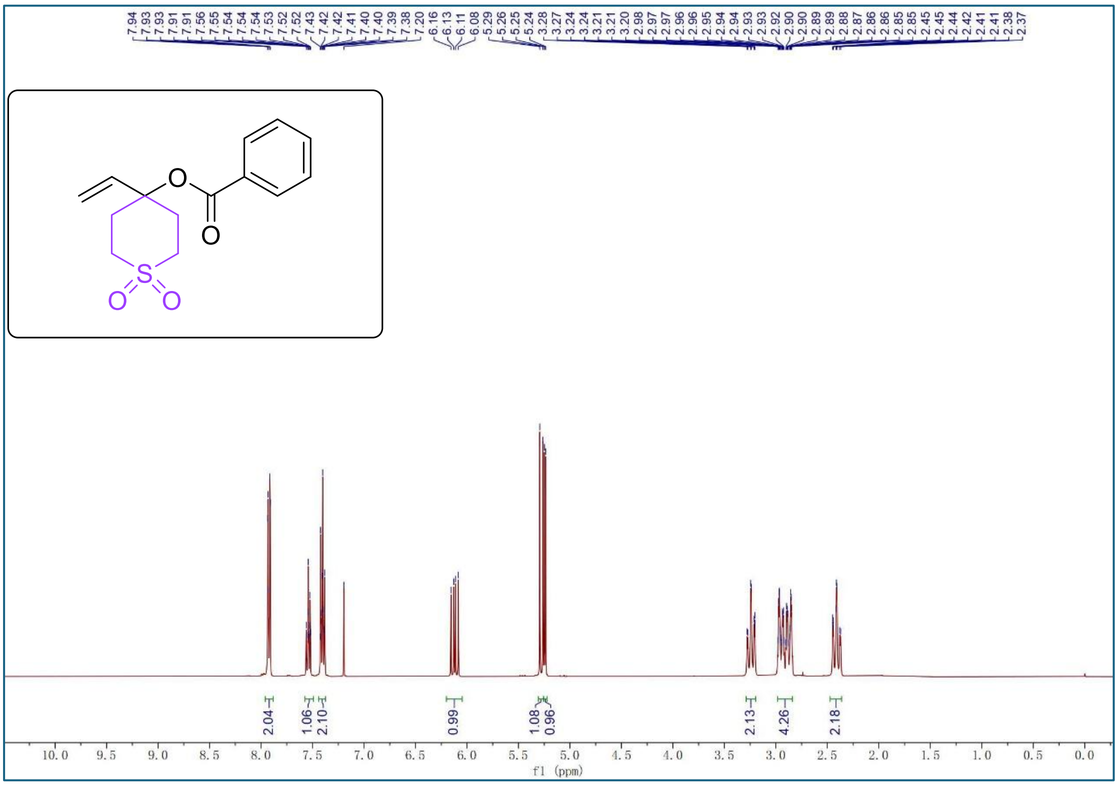

^{1}H NMR-spectrum (400 MHz, Chloroform-*d*) of **1u**

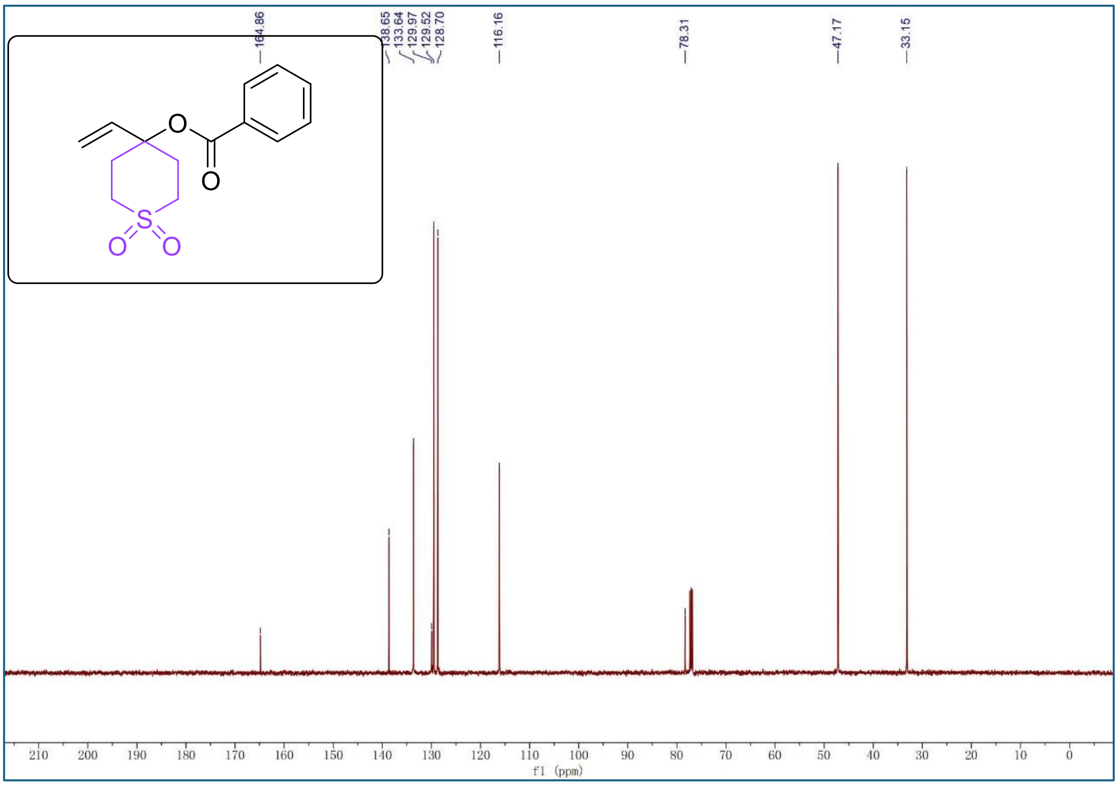

^{13}C NMR-spectrum (126 MHz, Chloroform-*d*) of **1u**

2-methylbut-3-en-2-yl benzoate (1v)

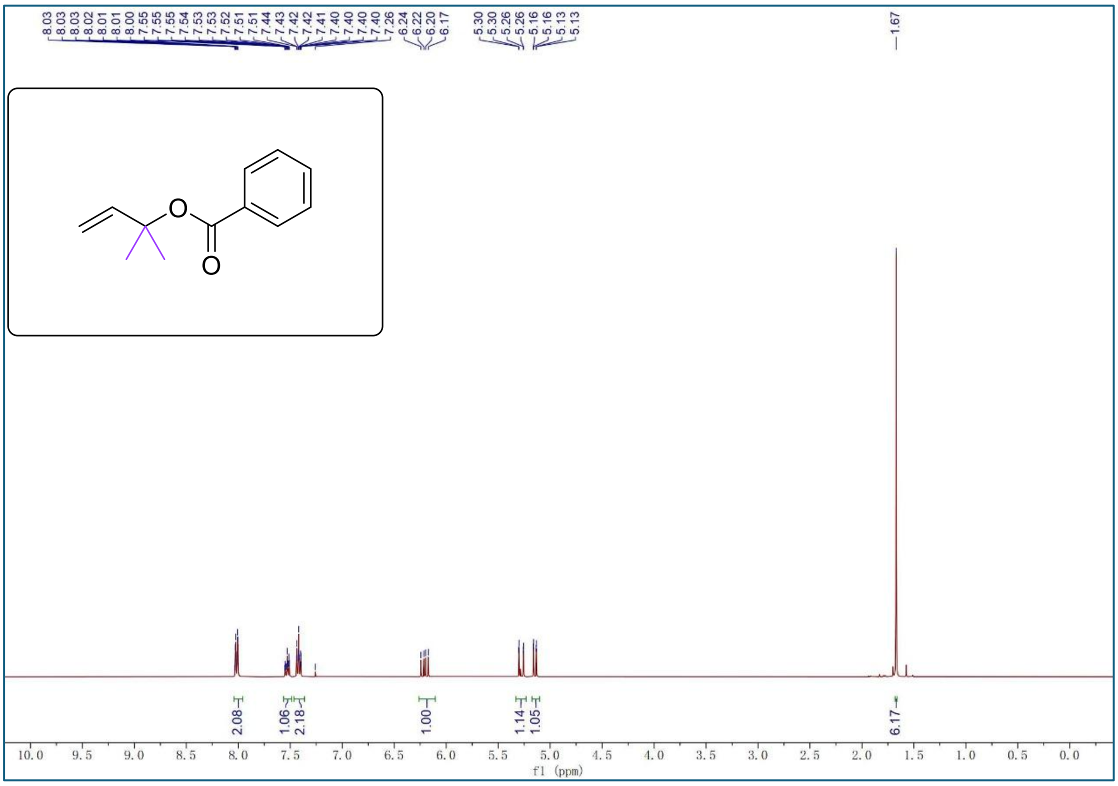

^{1}H NMR-spectrum (400 MHz, Chloroform-*d*) of **1v**

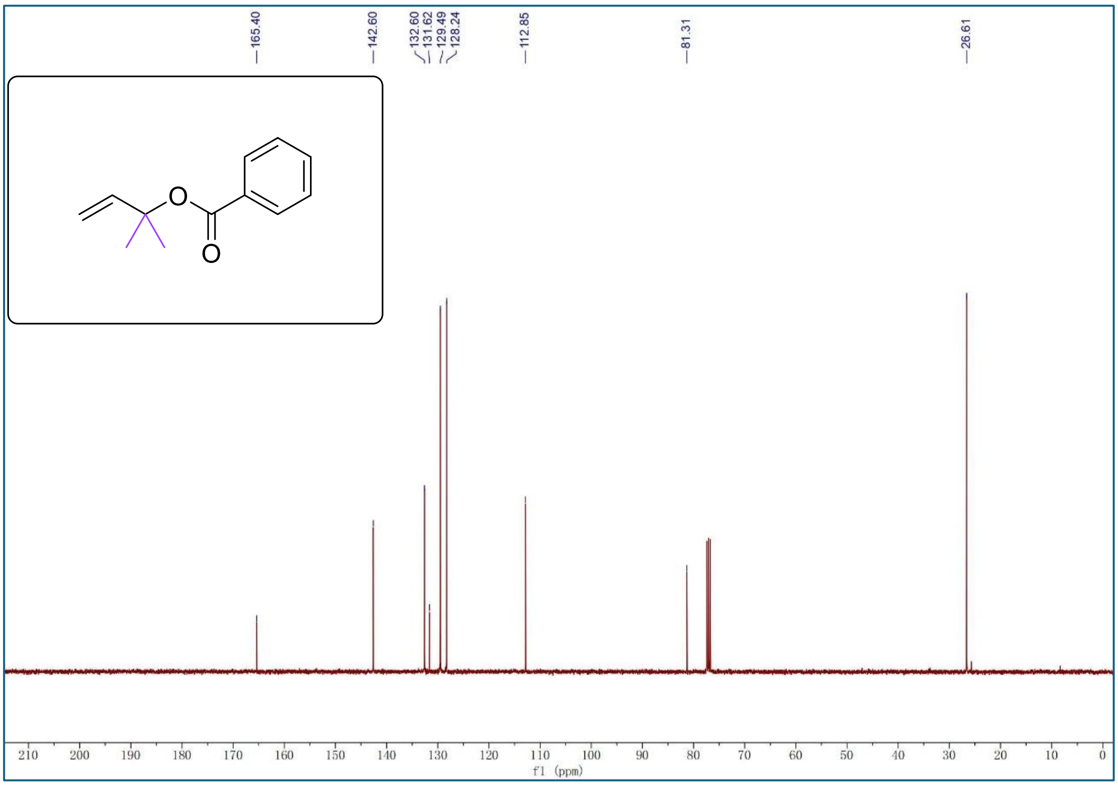

^{13}C NMR-spectrum (101 MHz, Chloroform-*d*) of **1v**

3-ethylpent-1-en-3-yl benzoate (1w)

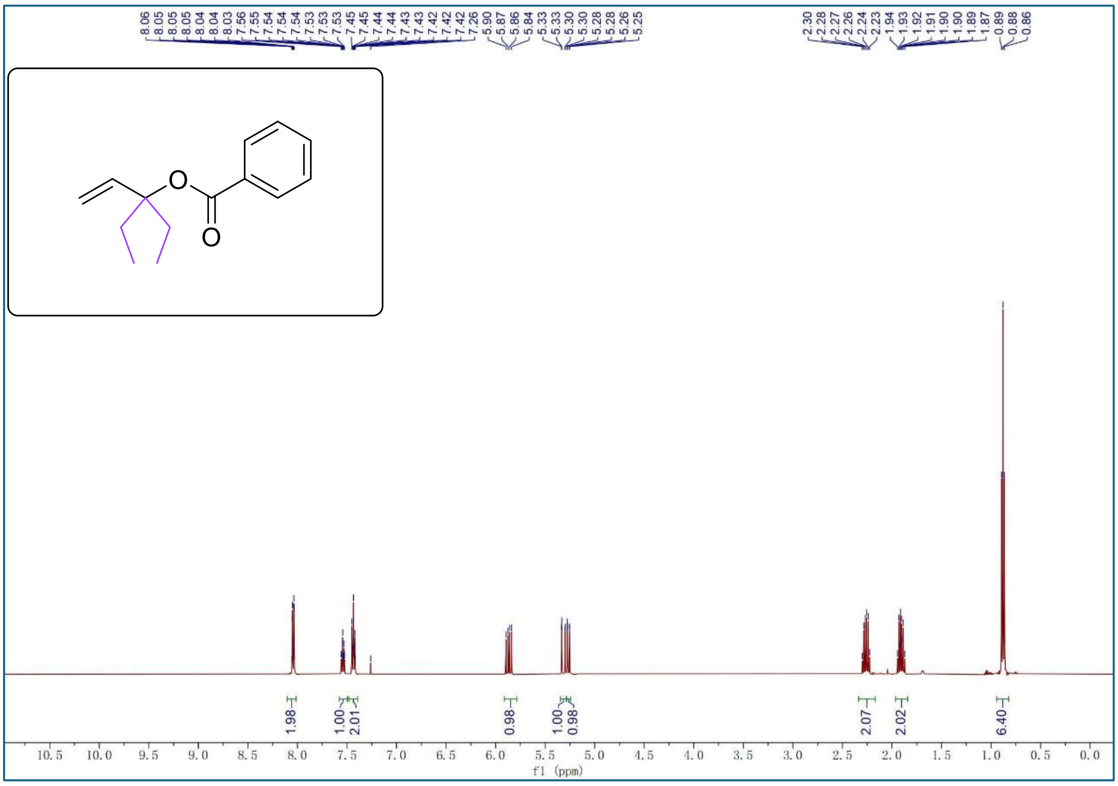

^{1}H NMR-spectrum (500 MHz, Chloroform-*d*) of **1w**

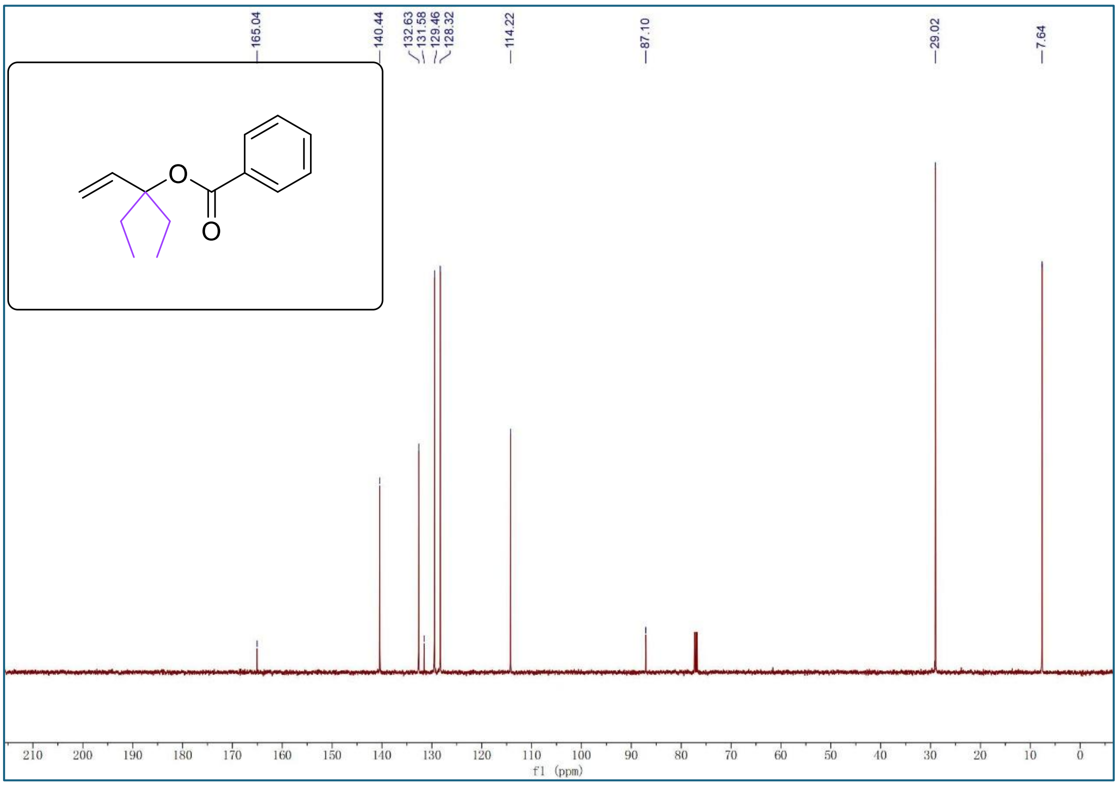

^{13}C NMR-spectrum (126 MHz, Chloroform-*d*) of **1w**

2,6-dimethyl-4-vinylheptan-4-yl benzoate (1x)

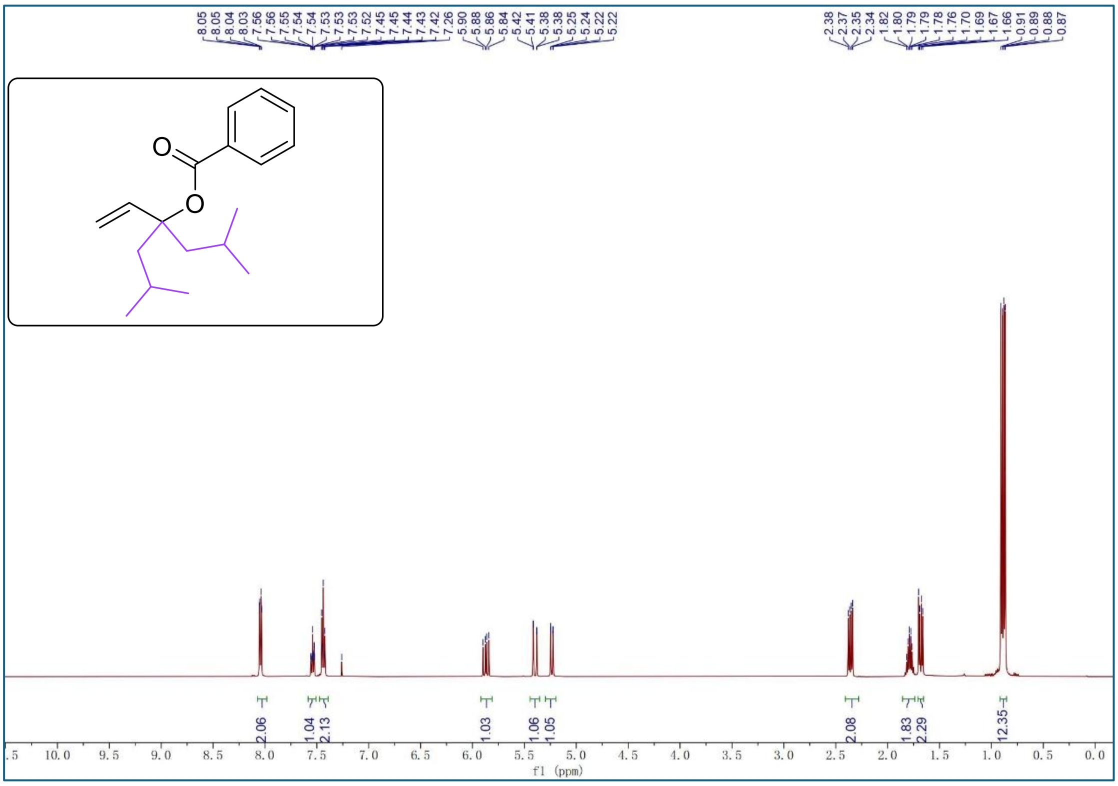

^{1}H NMR-spectrum (500 MHz, Chloroform-*d*) of **1x**

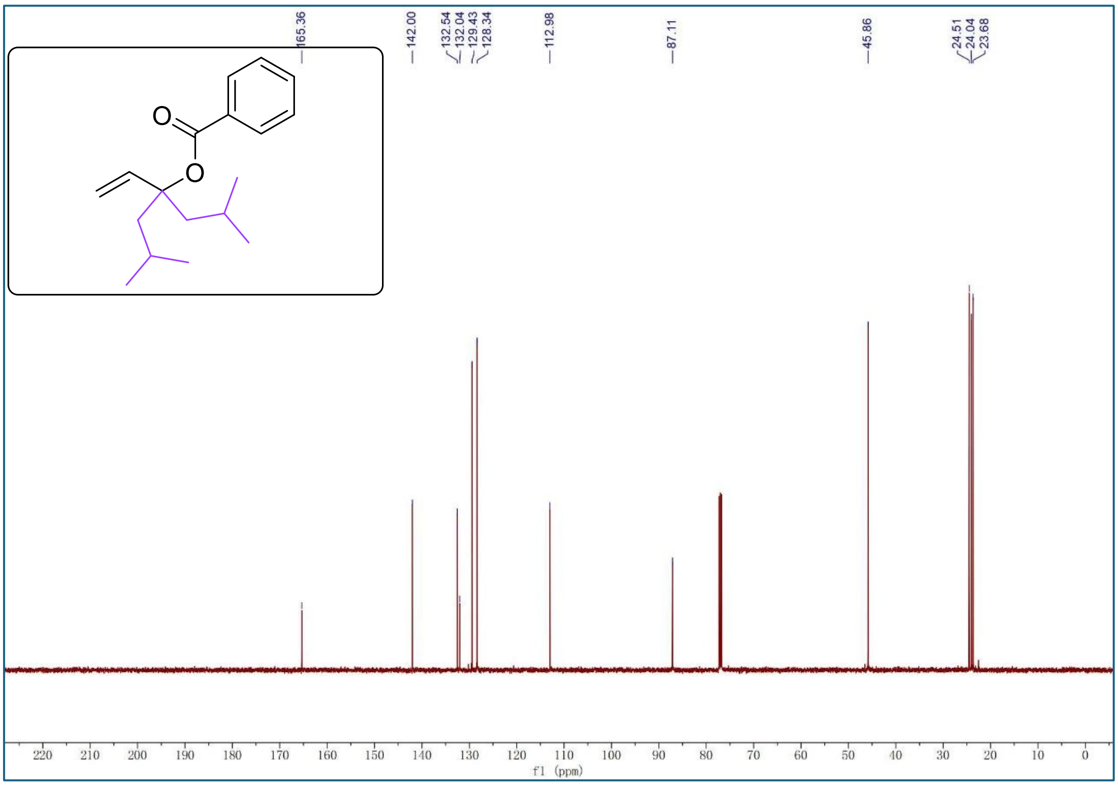

^{13}C NMR-spectrum (126 MHz, Chloroform-*d*) of **1x**

3-methylpent-1-en-3-yl benzoate (1y)

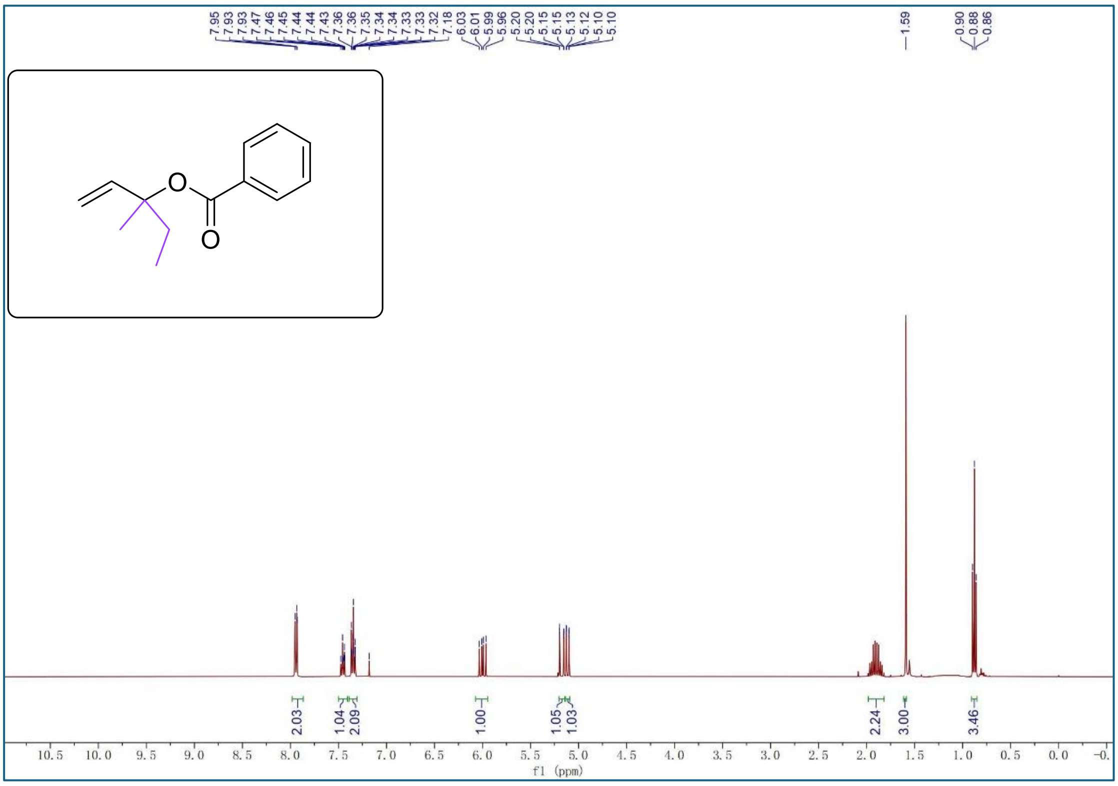

^{1}H NMR-spectrum (400 MHz, Chloroform-*d*) of **1y**

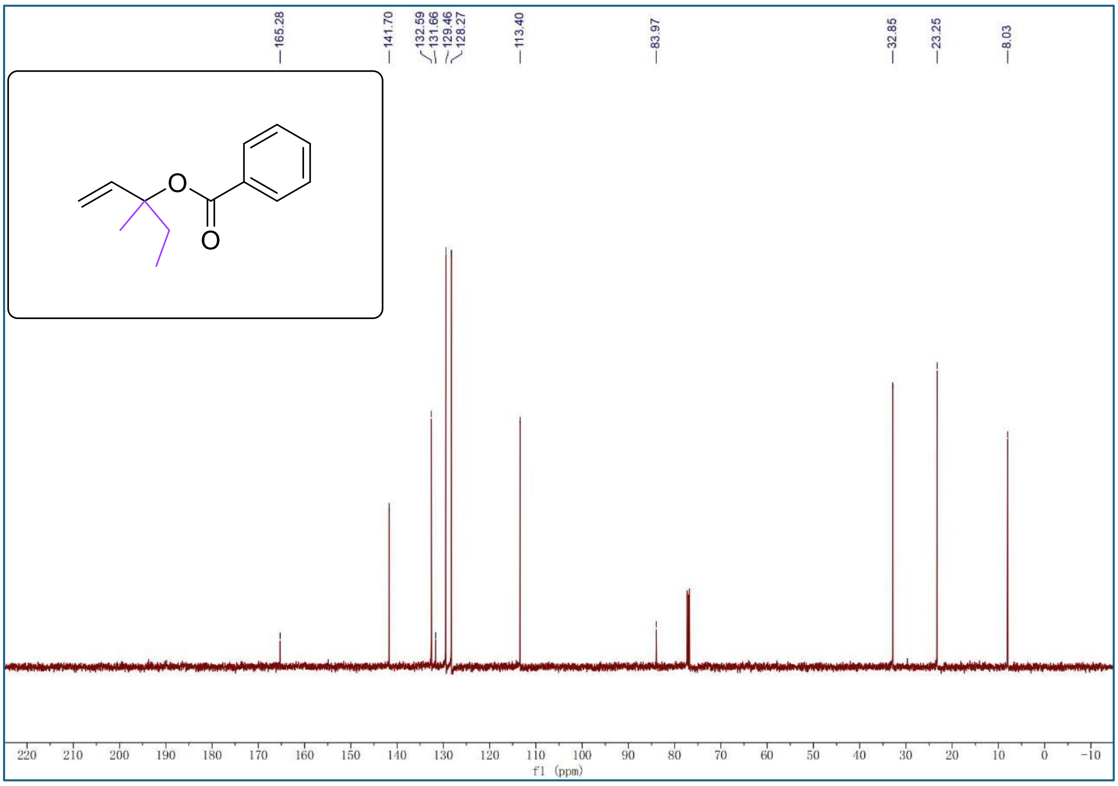

^{13}C NMR-spectrum (126 MHz, Chloroform-*d*) of **1y**

5-vinyloctahydro-1H-4,7-methanoinden-5-yl benzoate (1z)

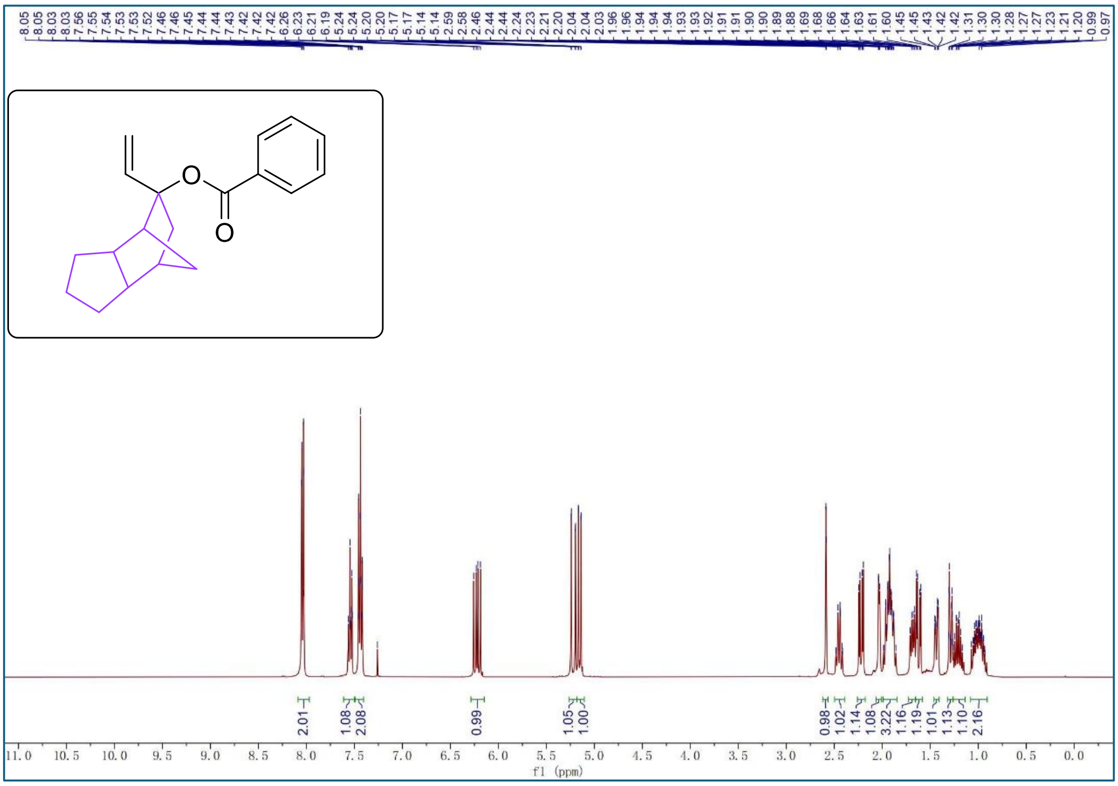

^{1}H NMR-spectrum (400 MHz, Chloroform-*d*) of **1z**

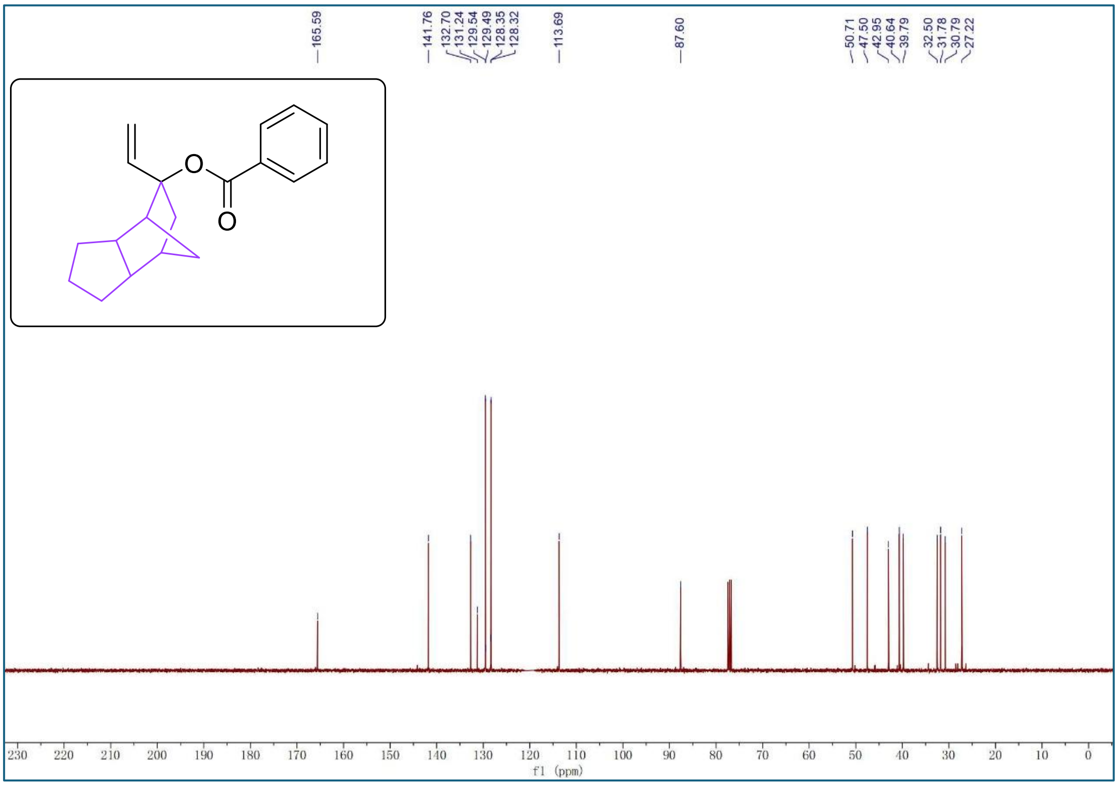

^{13}C NMR-spectrum (101 MHz, Chloroform-*d*) of **1z**

2-vinyladamantan-2-yl benzoate (1aa)

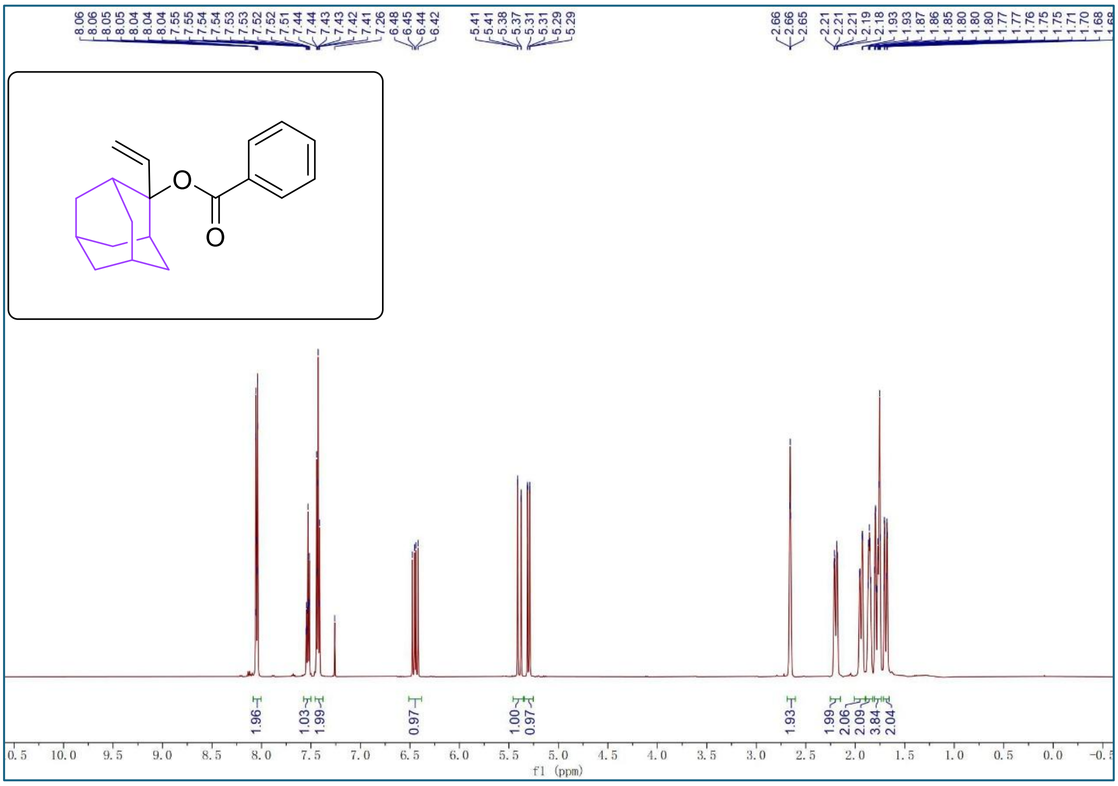

^{1}H NMR-spectrum (500 MHz, Chloroform-*d*) of **1aa**

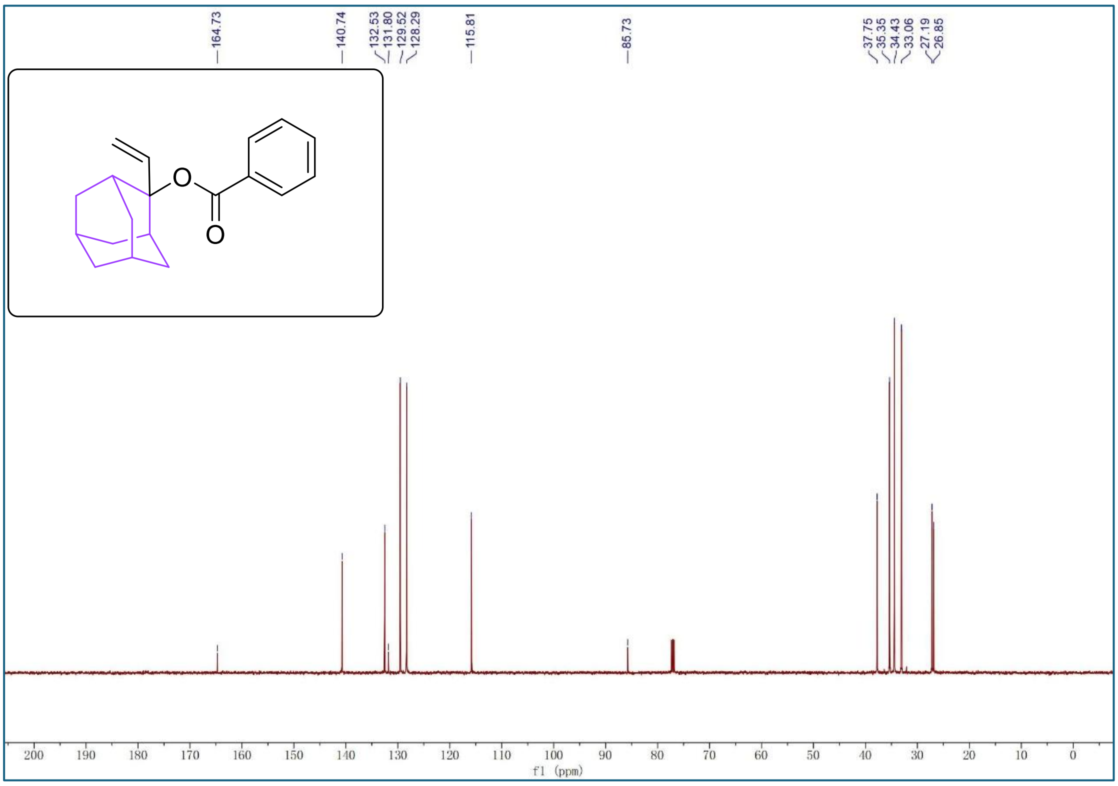

^{13}C NMR-spectrum (126 MHz, Chloroform-*d*) of **1aa**

2-vinylbicyclo[2.2.1]heptan-2-yl benzoate (1ab)

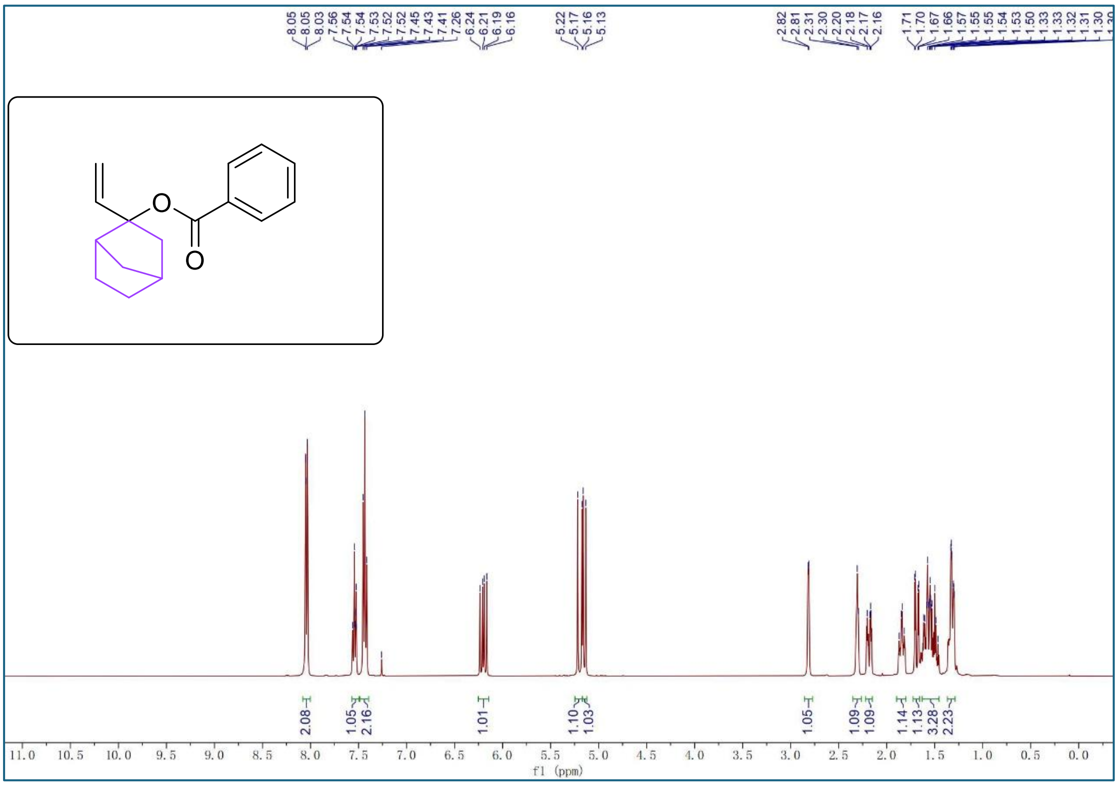

^{1}H NMR-spectrum (400 MHz, Chloroform-*d*) of **1ab**

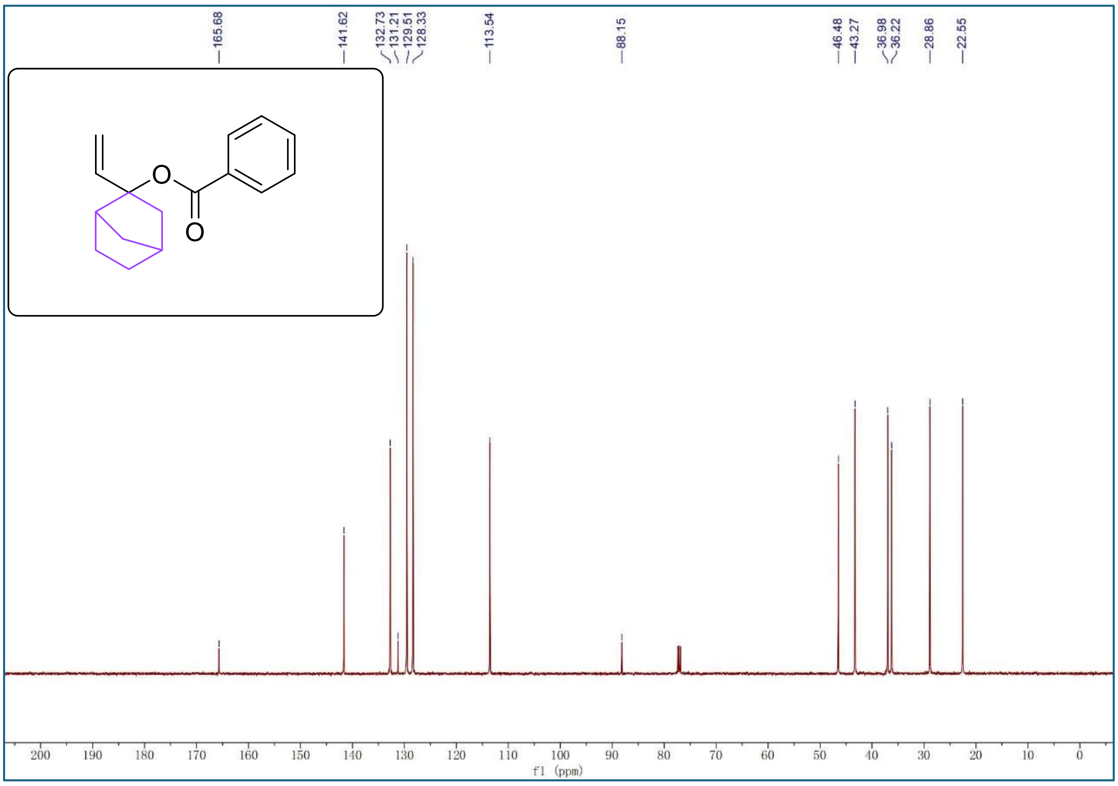

^{13}C NMR-spectrum (126 MHz, Chloroform-*d*) of **1ab**

(2S,5R)-2-isopropyl-5-methyl-1-vinylcyclohexyl benzoate (1ac)

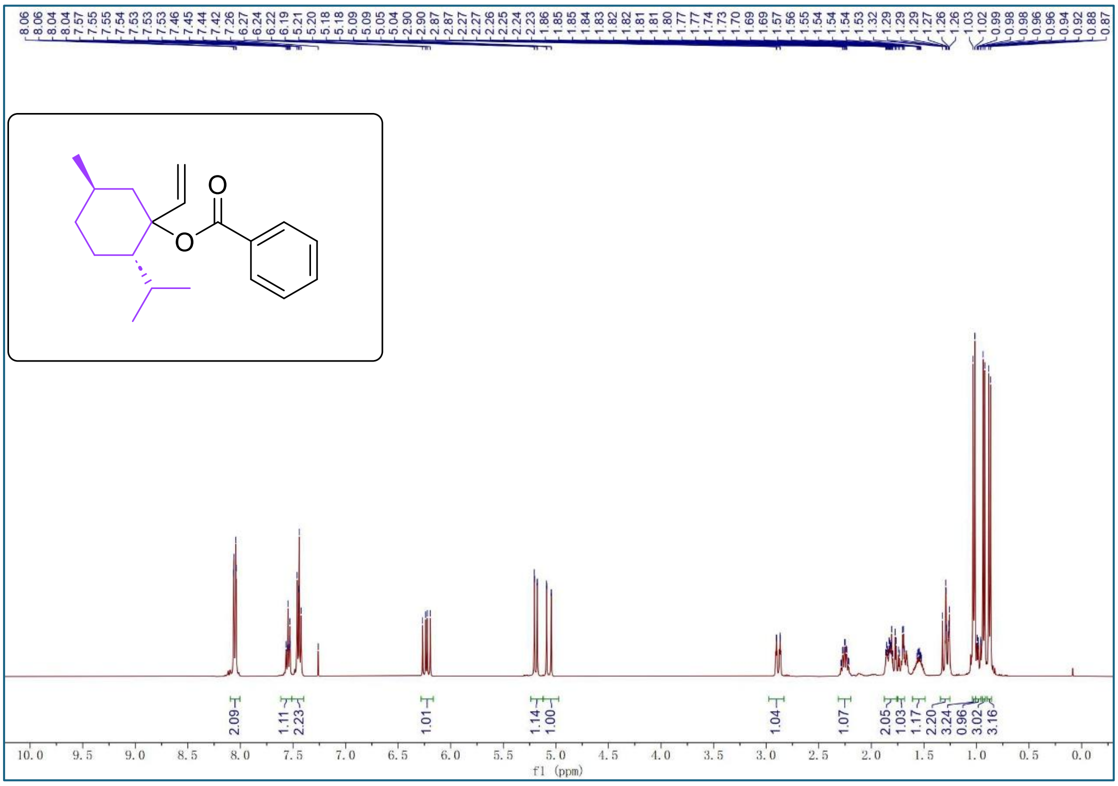

^{1}H NMR-spectrum (400 MHz, Chloroform-*d*) of **1ac**

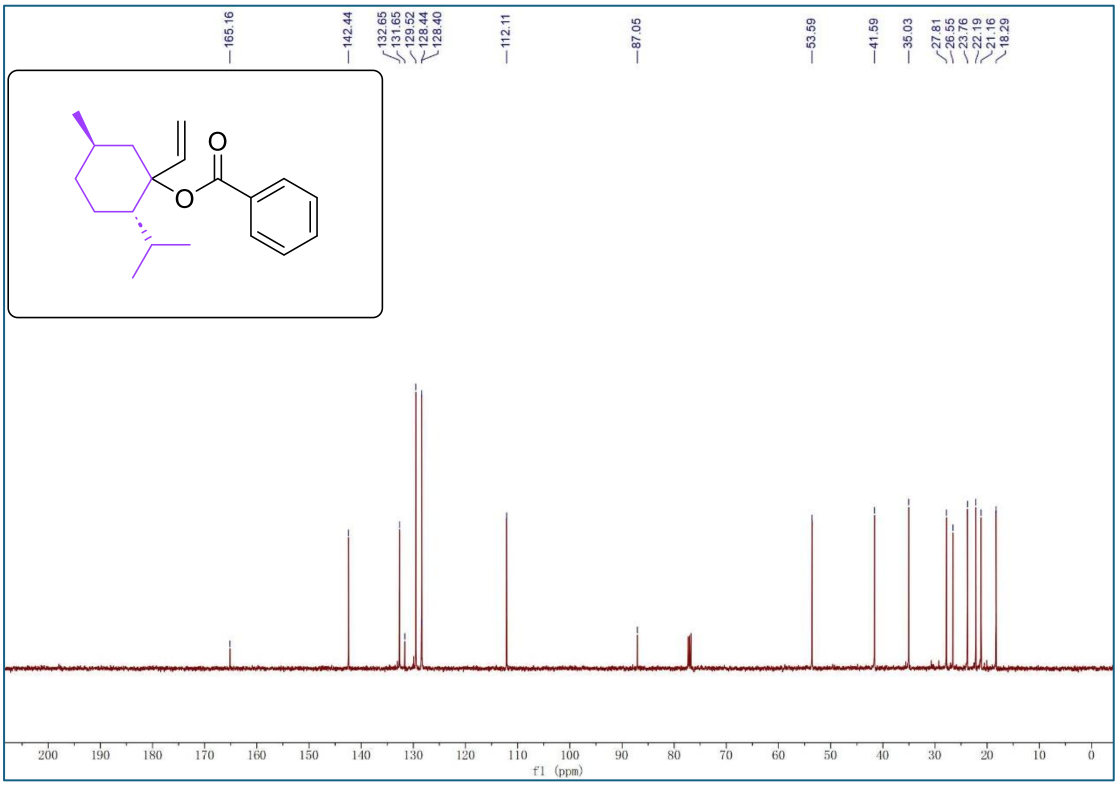

^{13}C NMR-spectrum (126 MHz, Chloroform-*d*) of **1ac**

1-vinylcyclohexyl 4-iodobenzoate (2a)

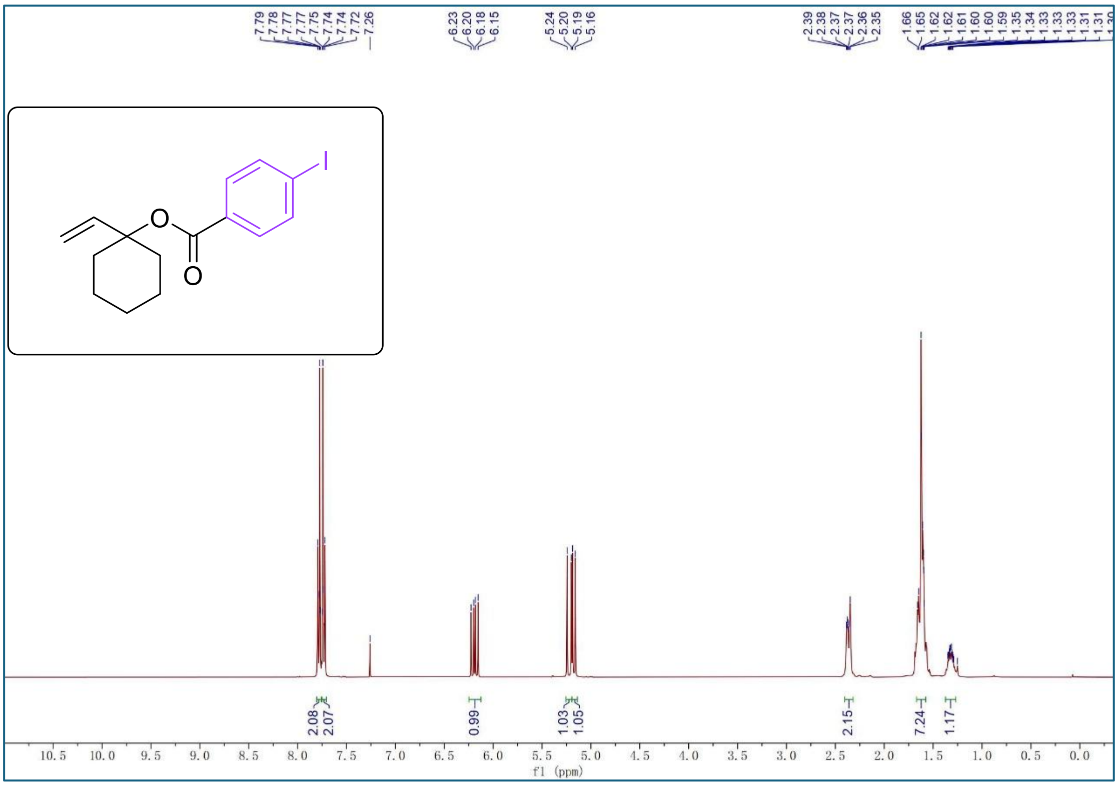

^{1}H NMR-spectrum (400 MHz, Chloroform-*d*) of **2a**

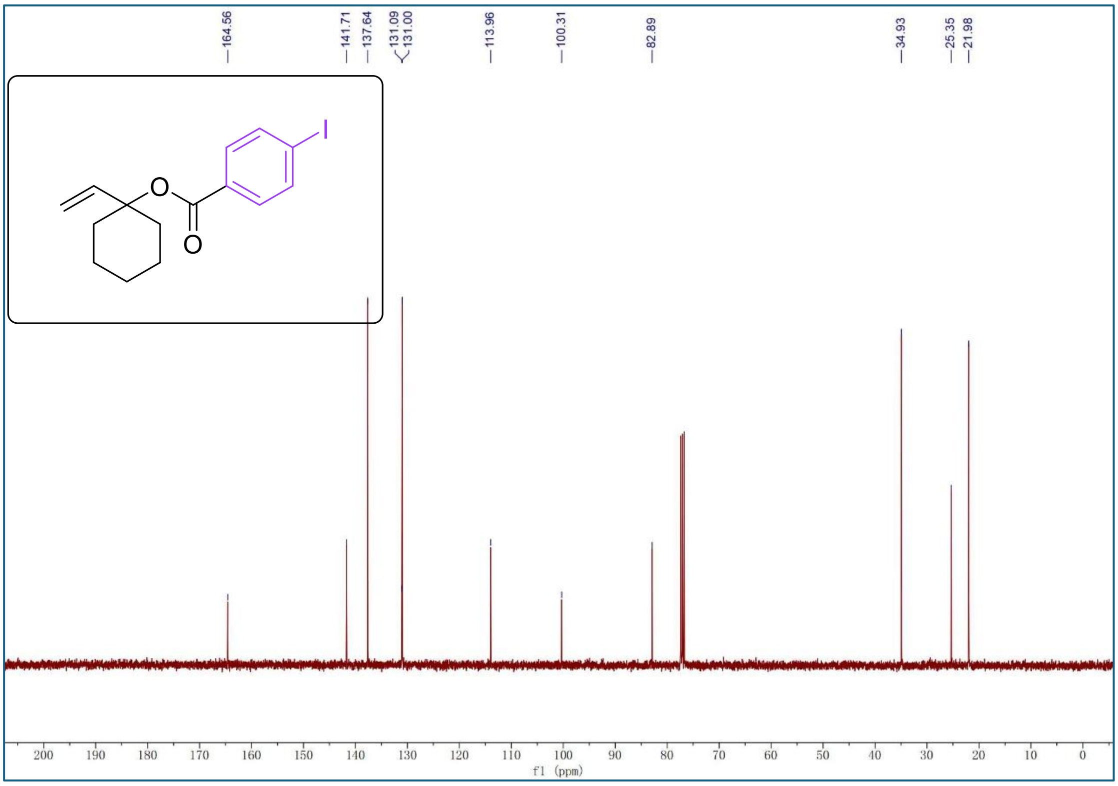

^{13}C NMR-spectrum (101 MHz, Chloroform-*d*) of **2a**

1-vinylcyclohexyl 4-(trifluoromethoxy)benzoate (2b)

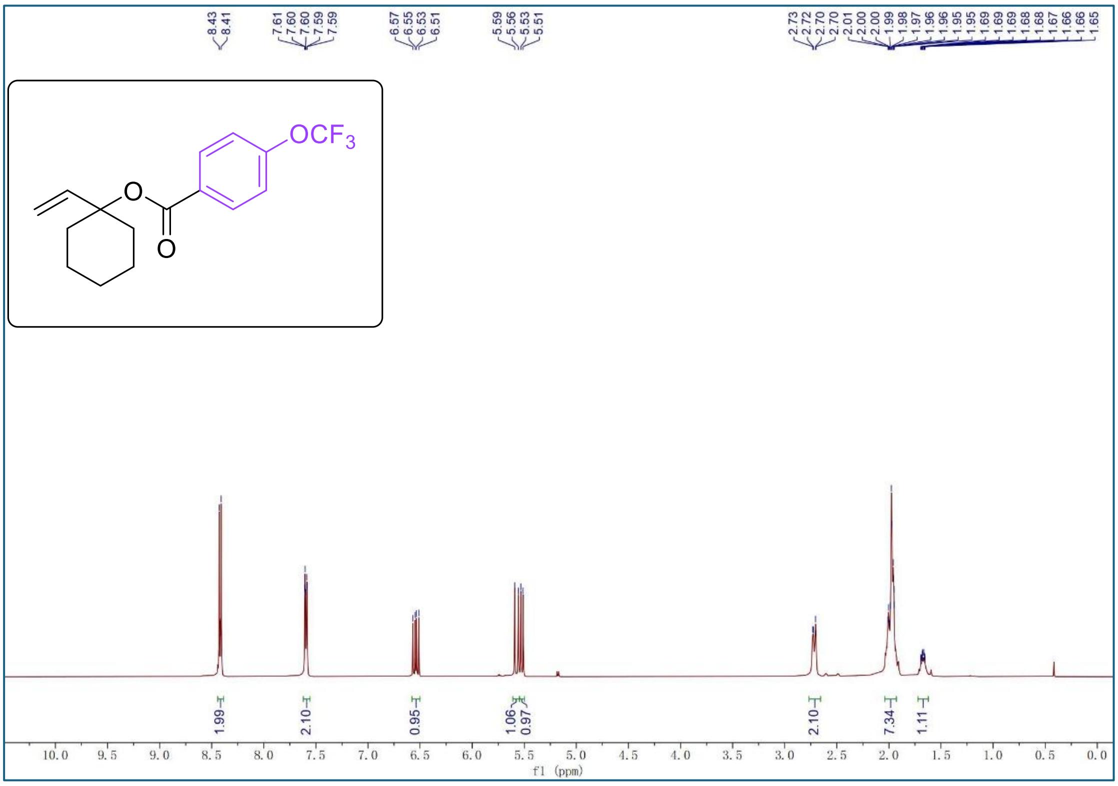

^{1}H NMR-spectrum (500 MHz, Chloroform-*d*) of **2b**

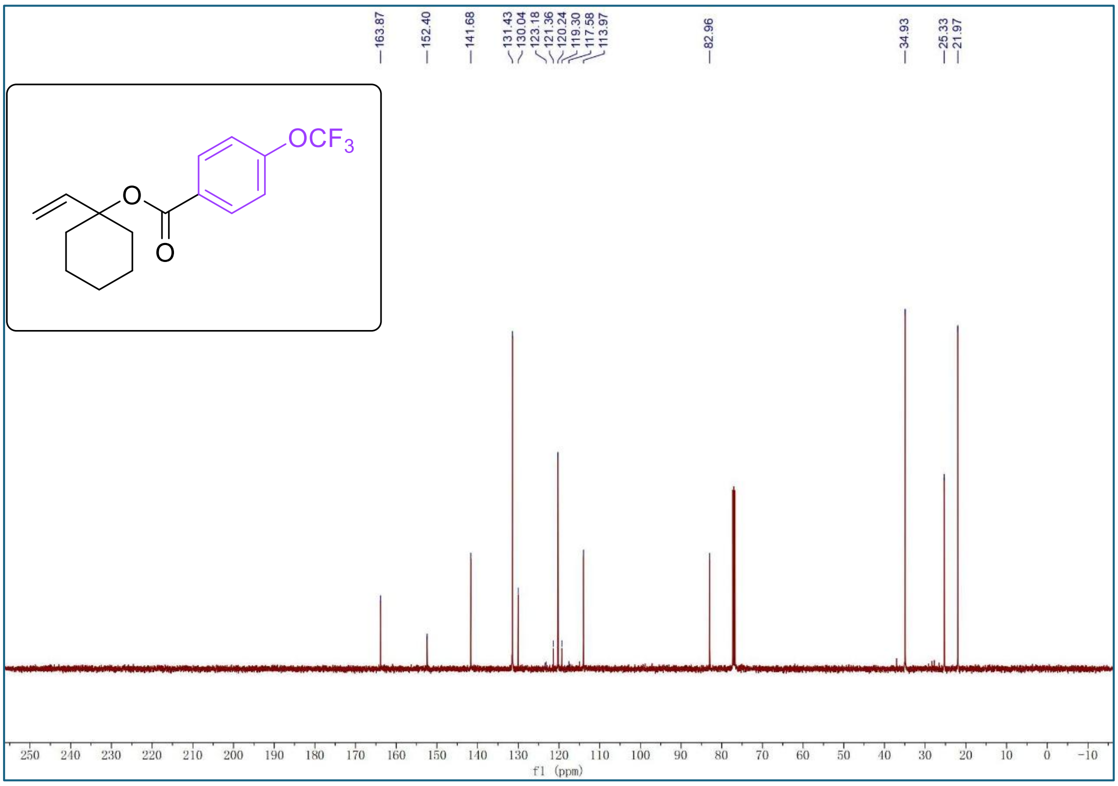

^{13}C NMR-spectrum (126 MHz, Chloroform-*d*) of **2b**

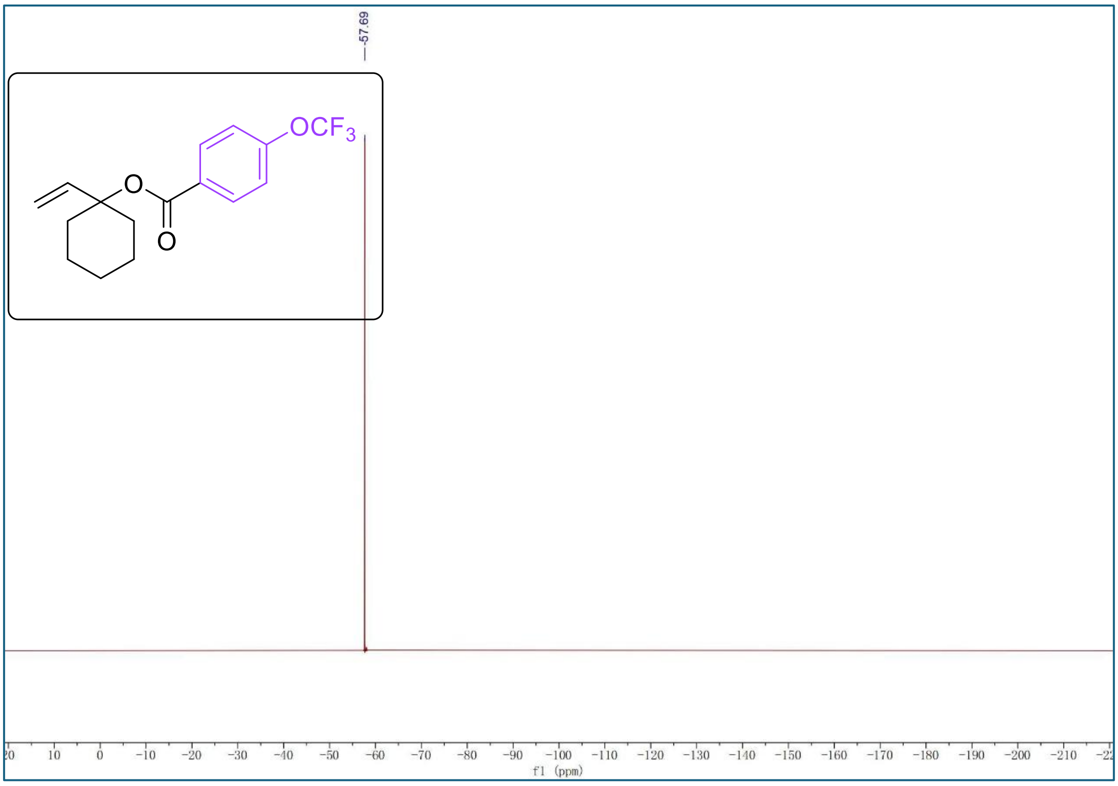

^{19}F NMR-spectrum (471 MHz, Chloroform-*d*) of **2b**

1-vinylcyclohexyl 4-(methylsulfonyl)benzoate (2c)

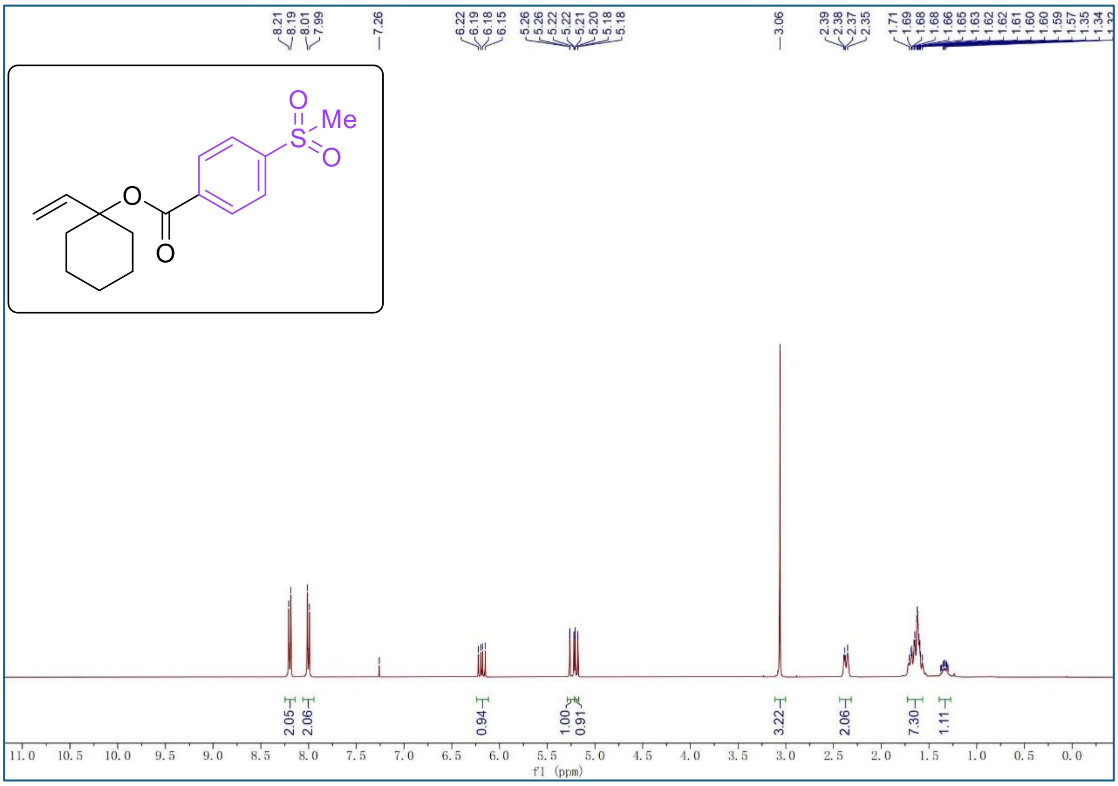

^{1}H NMR-spectrum (400 MHz, Chloroform-*d*) of **2c**

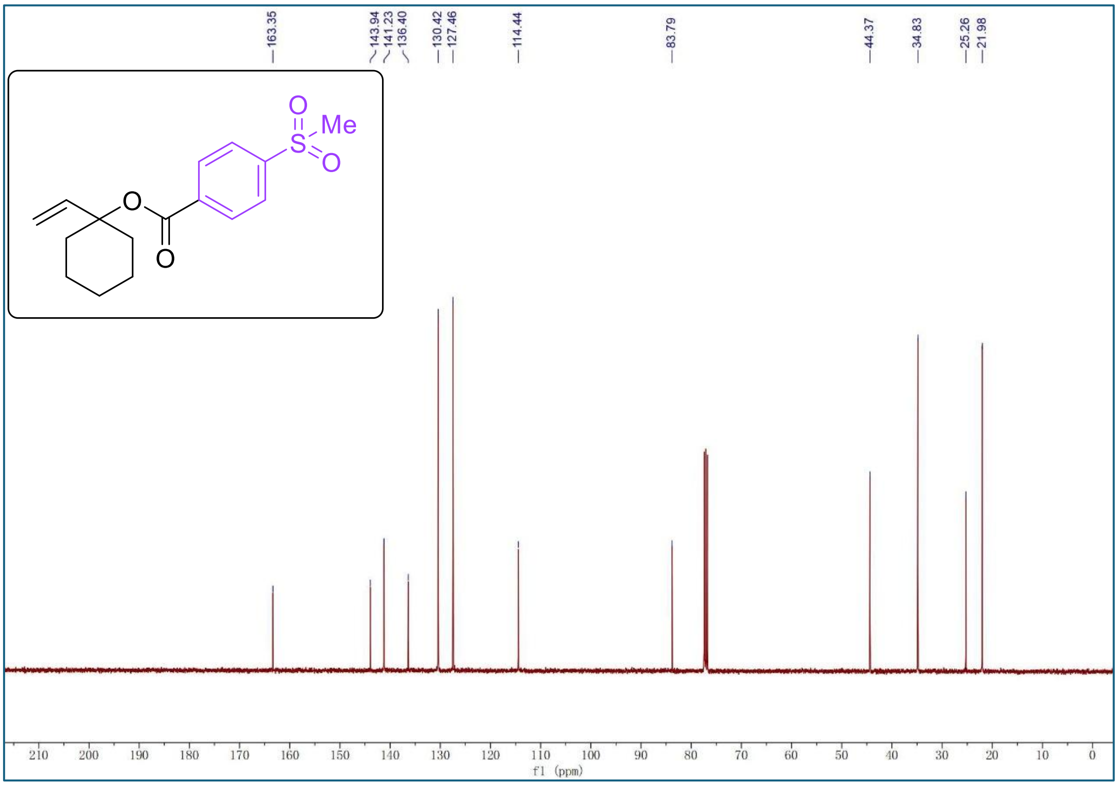

^{13}C NMR-spectrum (101 MHz, Chloroform-*d*) of **2c**

SUPPORTING INFORMATION

methyl (2-methylbut-3-en-2-yl) terephthalate (2d)

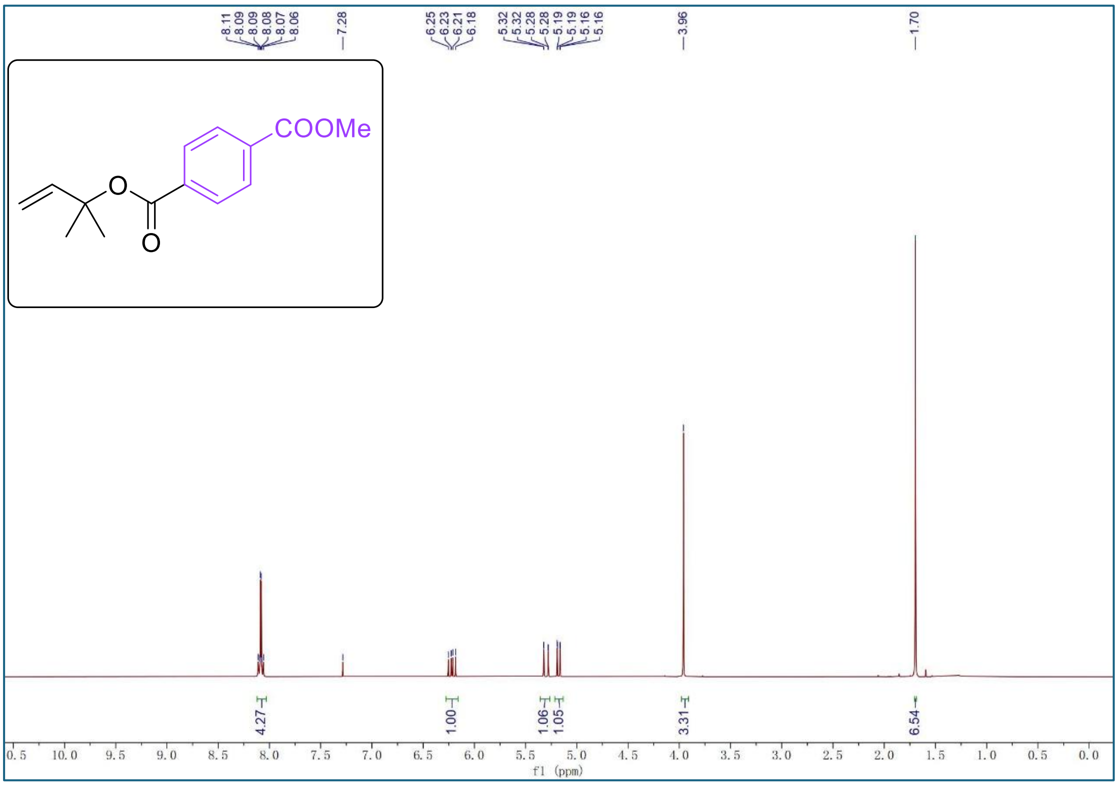

^{1}H NMR-spectrum (400 MHz, Chloroform-*d*) of **2d**

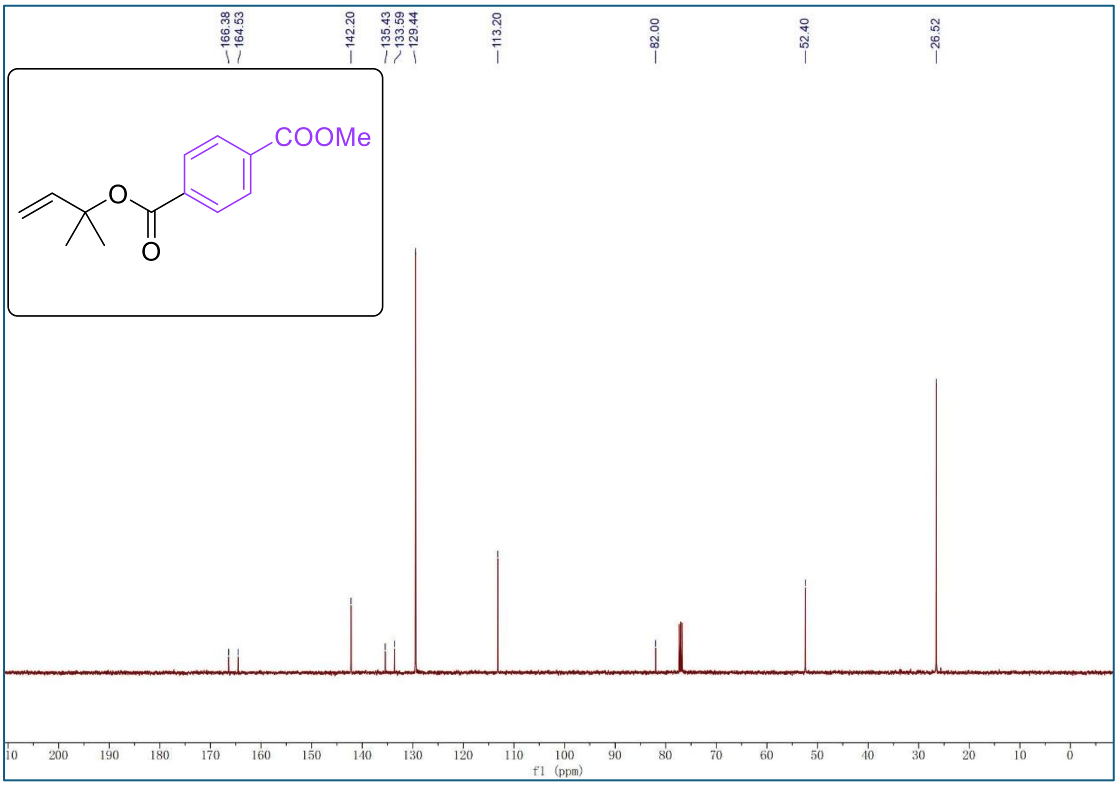

^{13}C NMR-spectrum (101 MHz, Chloroform-*d*) of **2d**

SUPPORTING INFORMATION

2-methylbut-3-en-2-yl 4-cyanobenzoate (2e)

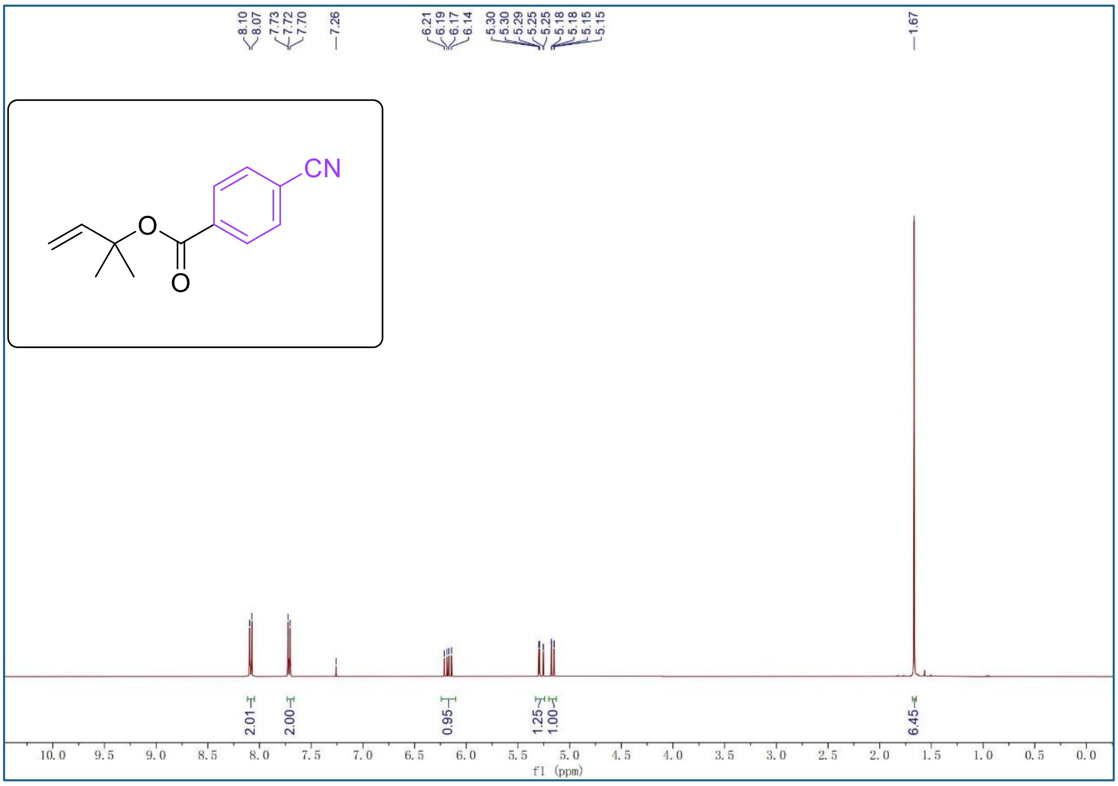

^{1}H NMR-spectrum (400 MHz, Chloroform-*d*) of **2e**

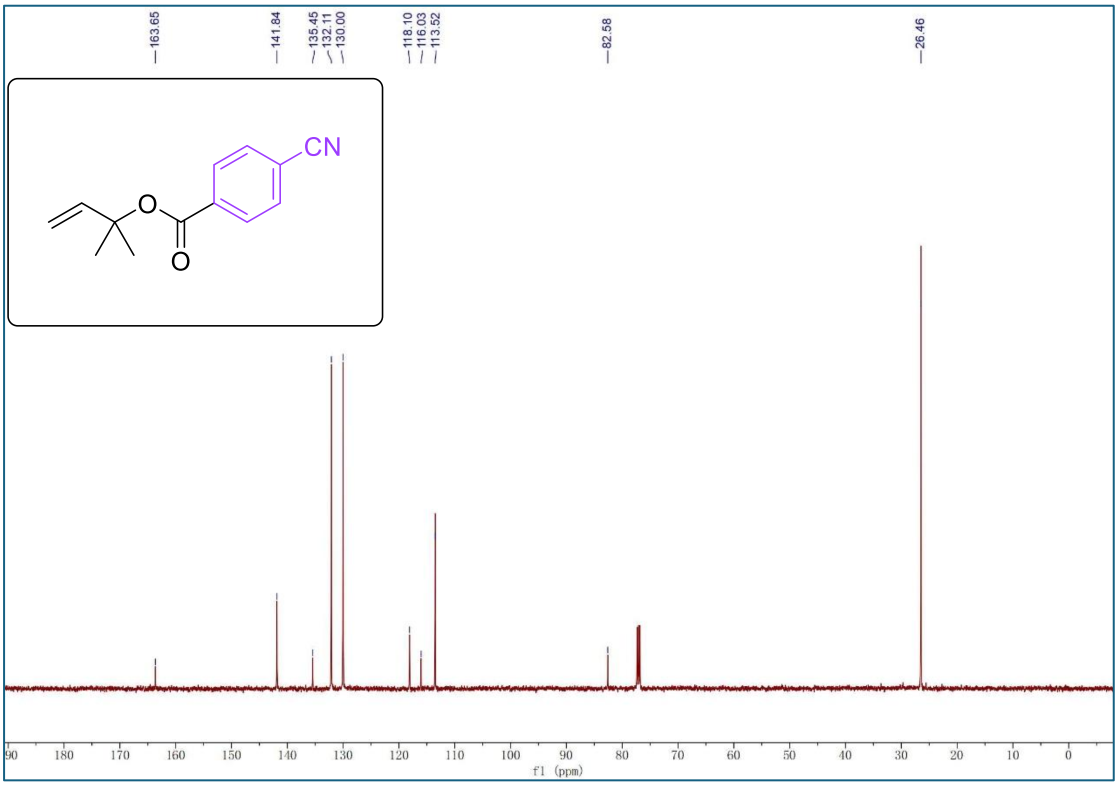

^{13}C NMR-spectrum (126 MHz, Chloroform-*d*) of **2e**

2-methylbut-3-en-2-yl 4-nitrobenzoate (2f)

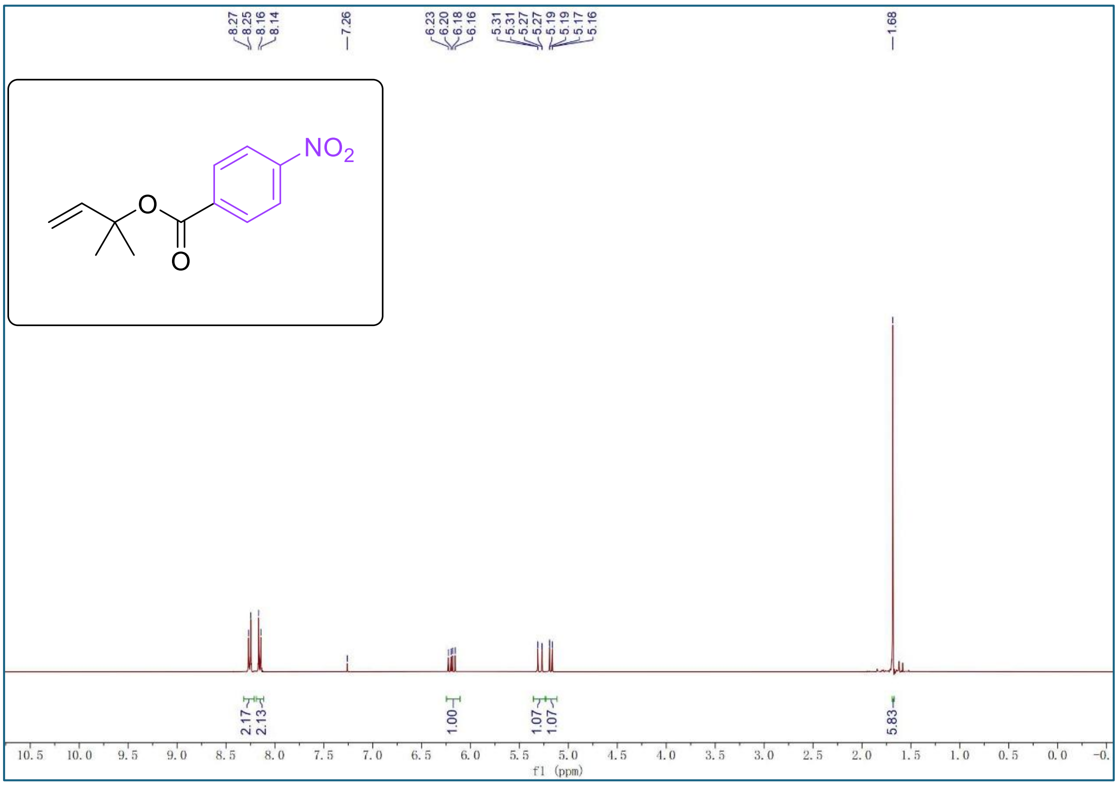

^{1}H NMR-spectrum (400 MHz, Chloroform-*d*) of **2f**

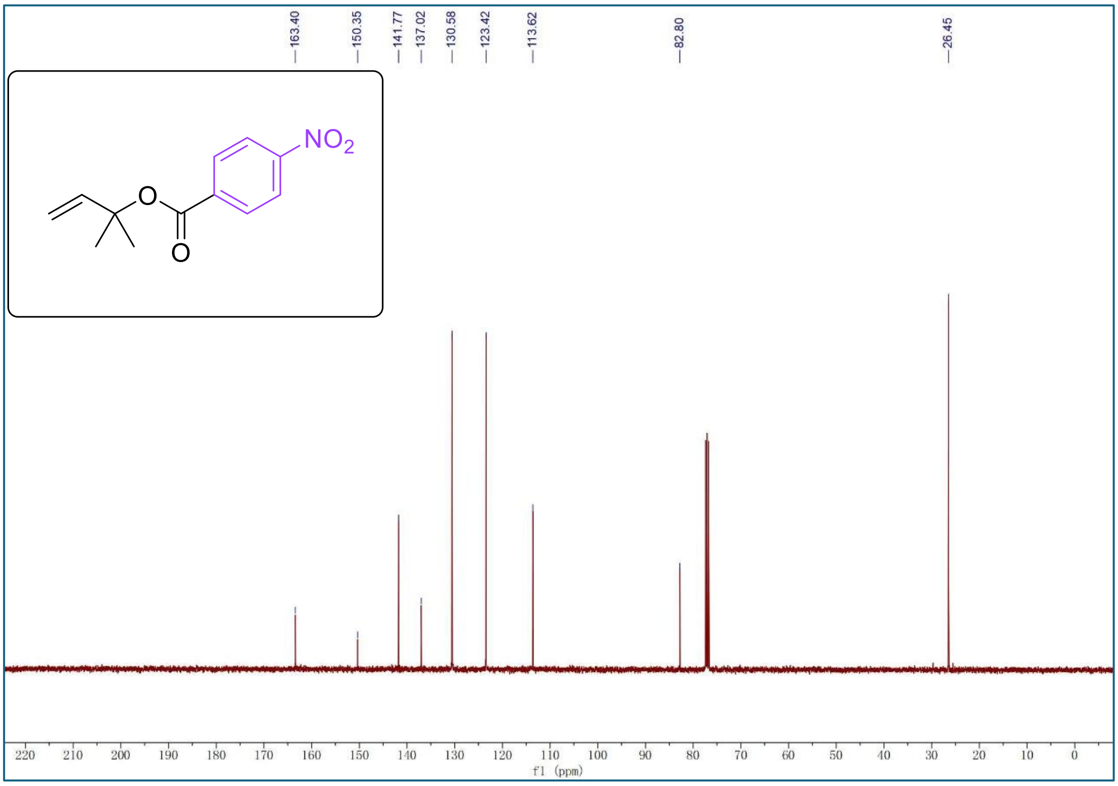

^{13}C NMR-spectrum (101 MHz, Chloroform-*d*) of **2f**

2-methylbut-3-en-2-yl 4-iodobenzoate (2g)

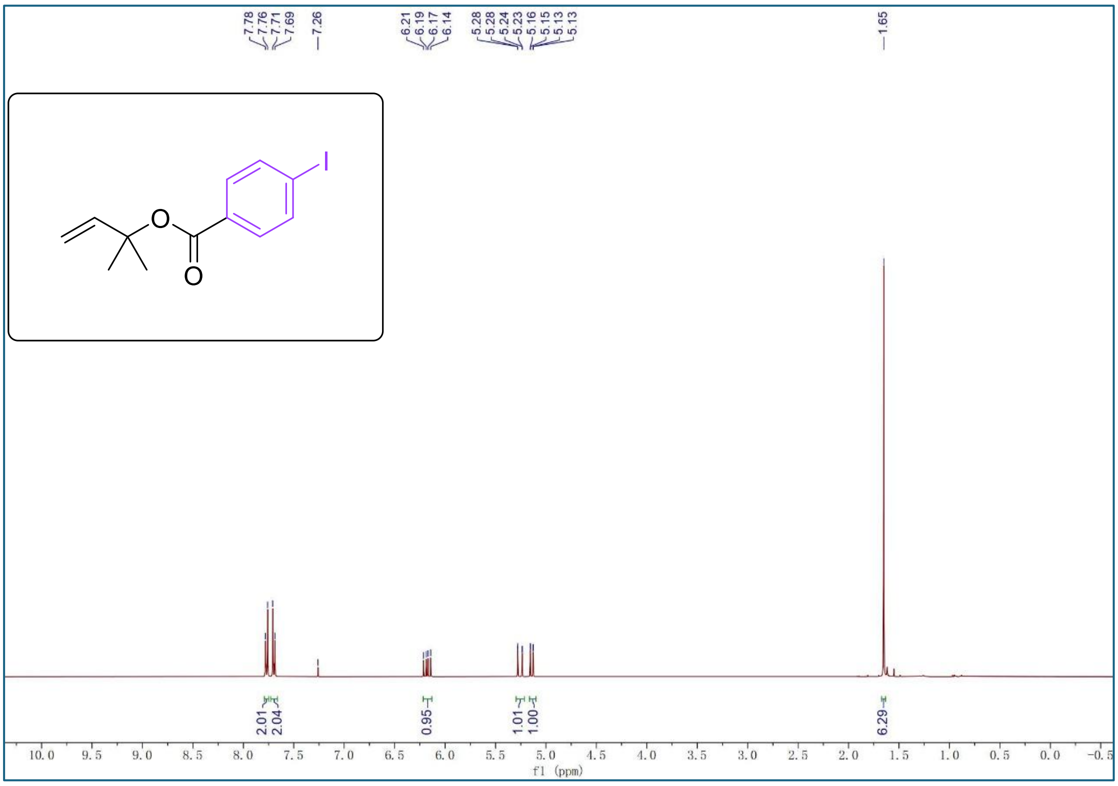

^{1}H NMR-spectrum (400 MHz, Chloroform-*d*) of **2g**

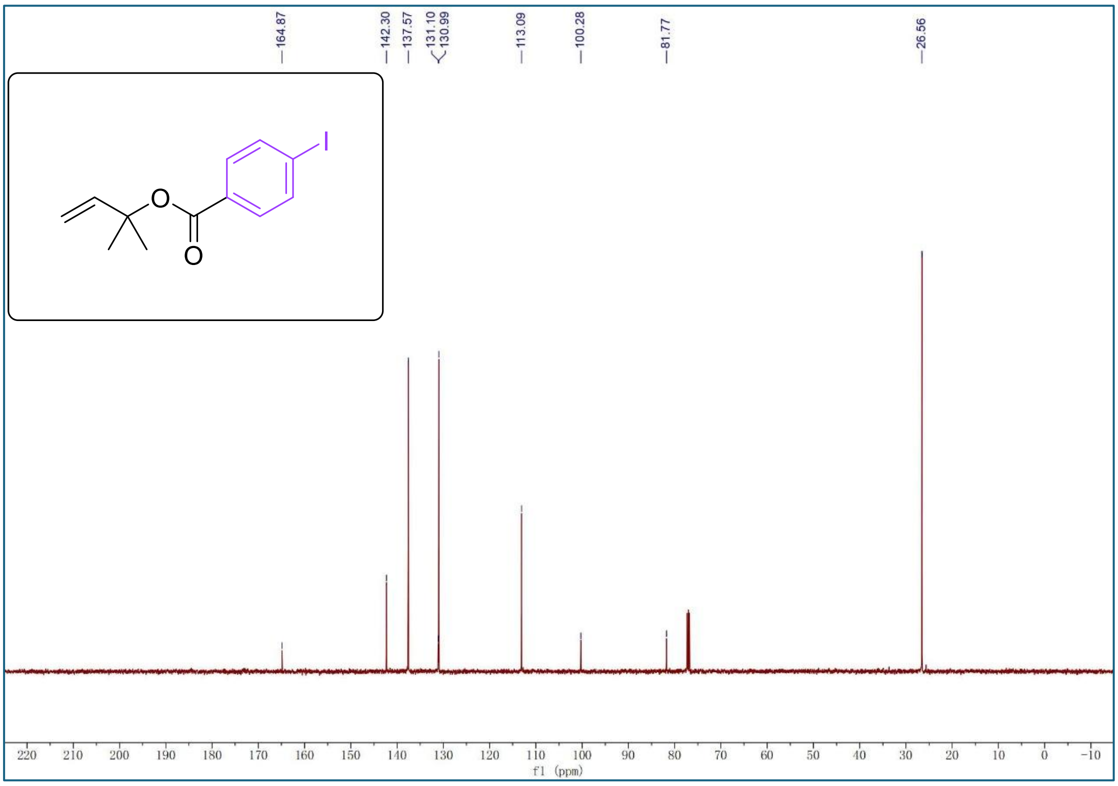

^{13}C NMR-spectrum (126 MHz, Chloroform-*d*) of **2g**

2-methylbut-3-en-2-yl 4-(chloromethyl)benzoate (2h)

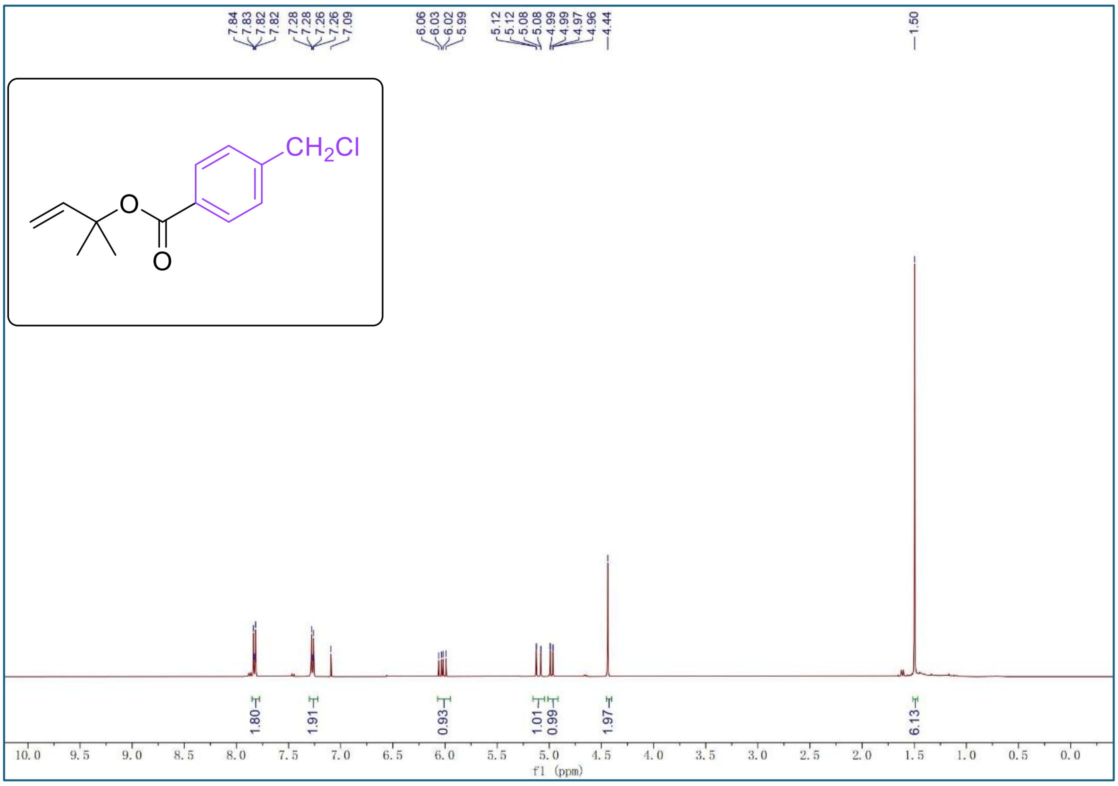

^{1}H NMR-spectrum (400 MHz, Chloroform-*d*) of **2h**

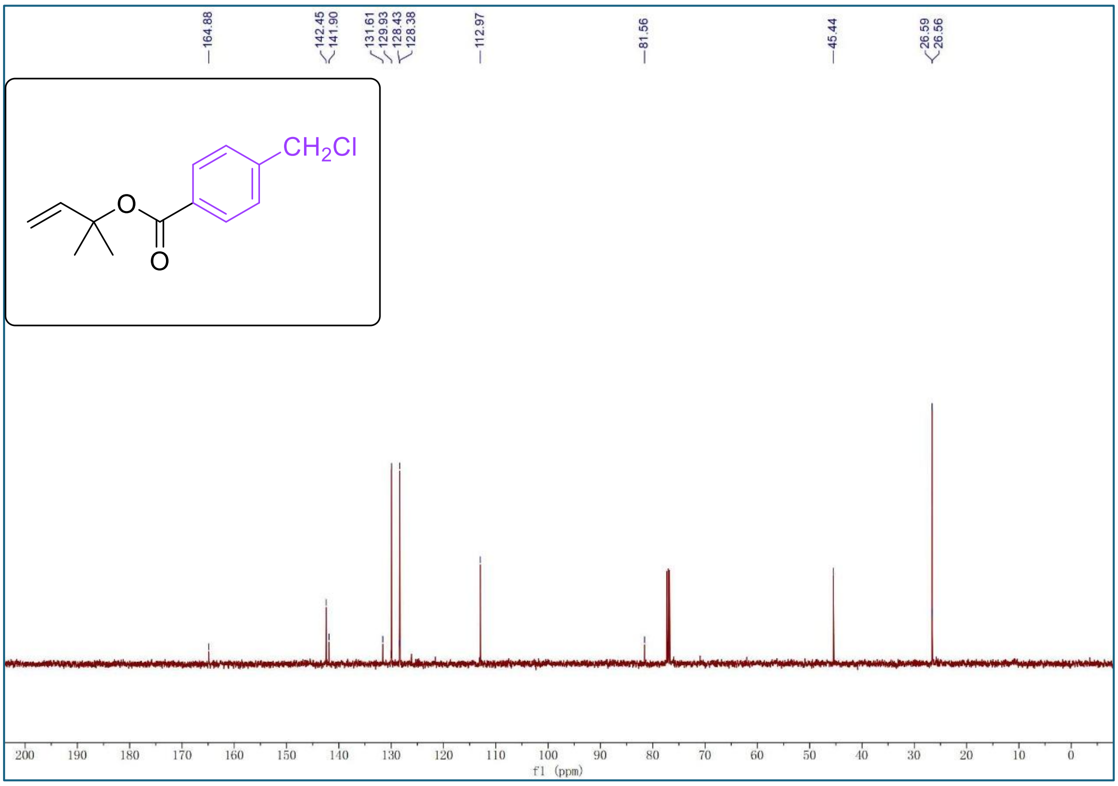

^{13}C NMR-spectrum (101 MHz, Chloroform-*d*) of **2h**

2-methylbut-3-en-2-yl 4-(trifluoromethoxy)benzoate (2i)

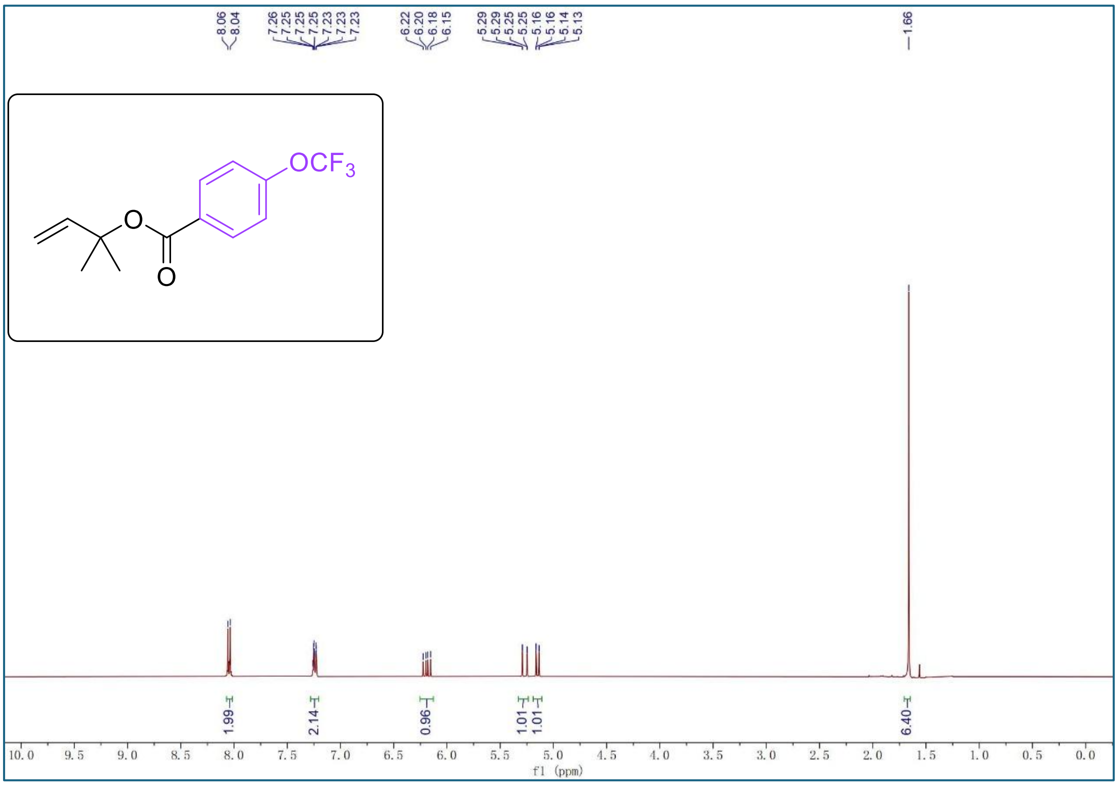

^{1}H NMR-spectrum (400 MHz, Chloroform-*d*) of **2i**

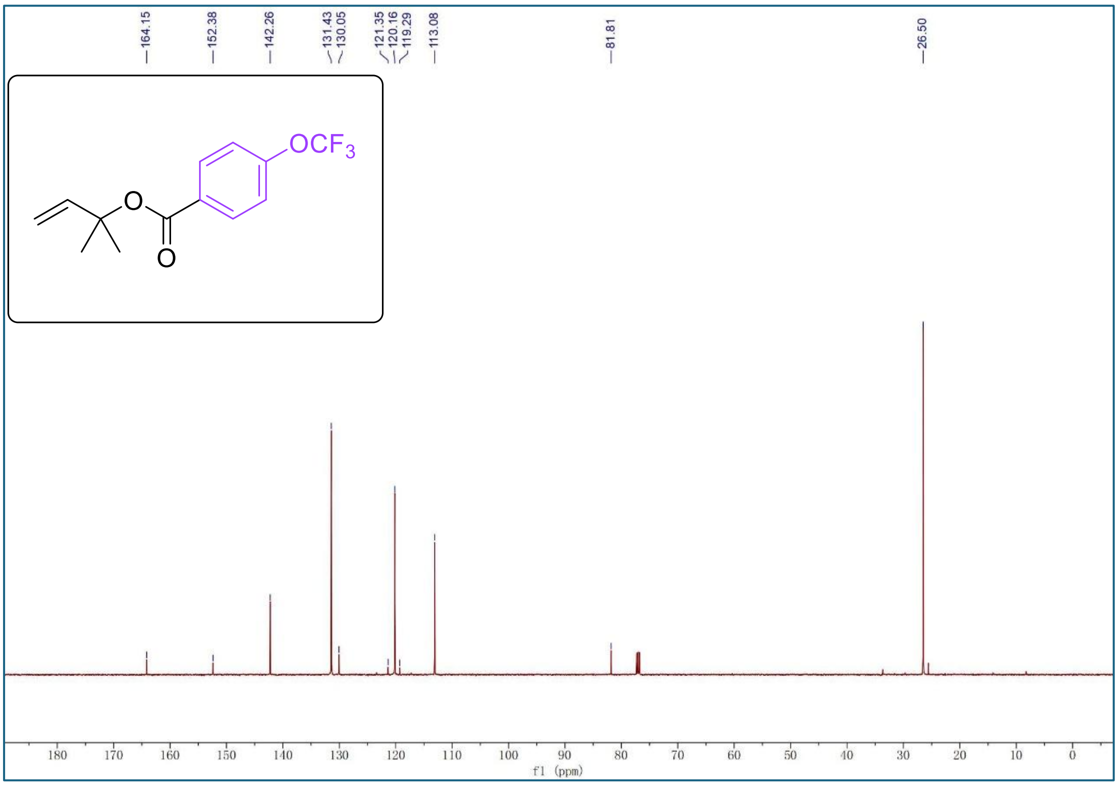

^{13}C NMR-spectrum (126 MHz, Chloroform-*d*) of **2i**

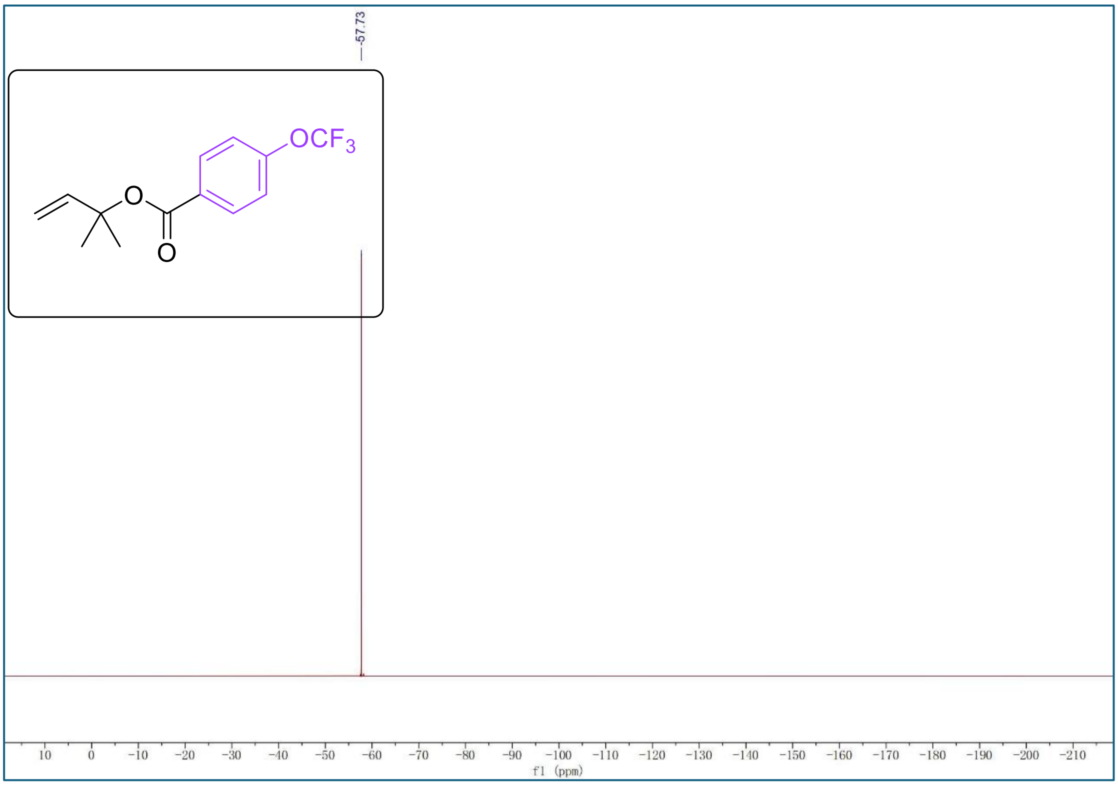

^{19}F NMR-spectrum (377 MHz, Chloroform-*d*) of **2i**

2-methylbut-3-en-2-yl 4-ethoxybenzoate (2j)

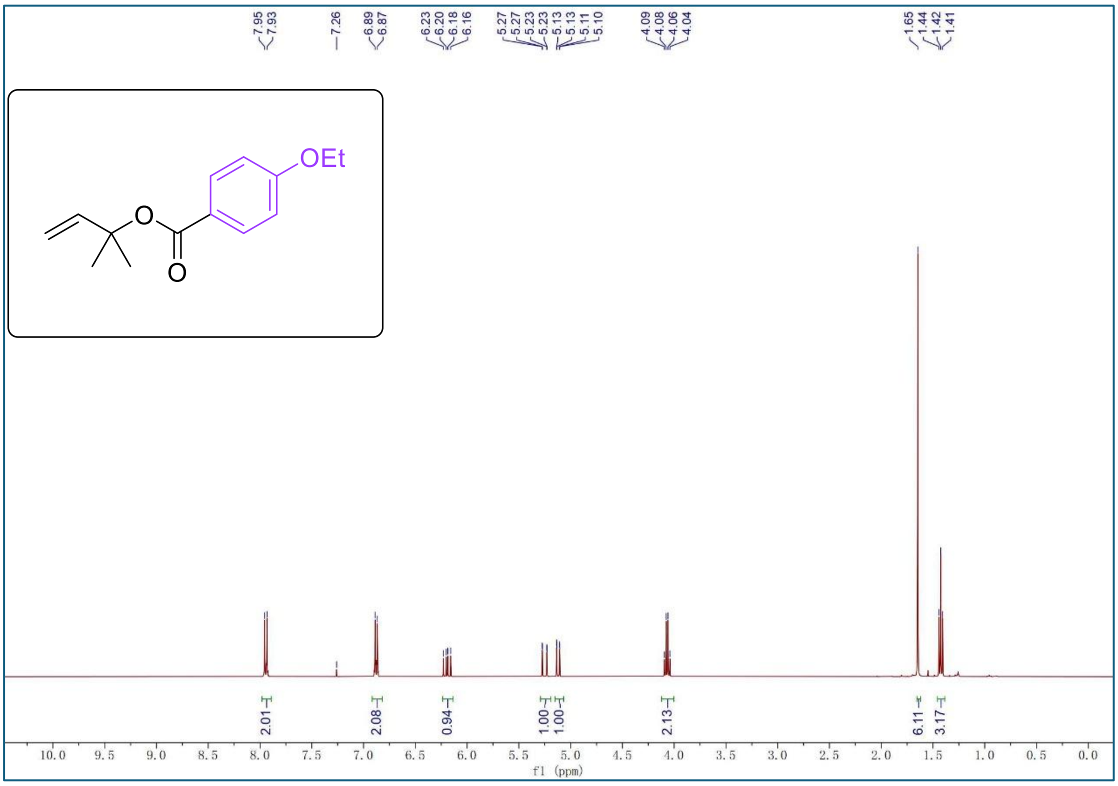

^{1}H NMR-spectrum (400 MHz, Chloroform-*d*) of **2j**

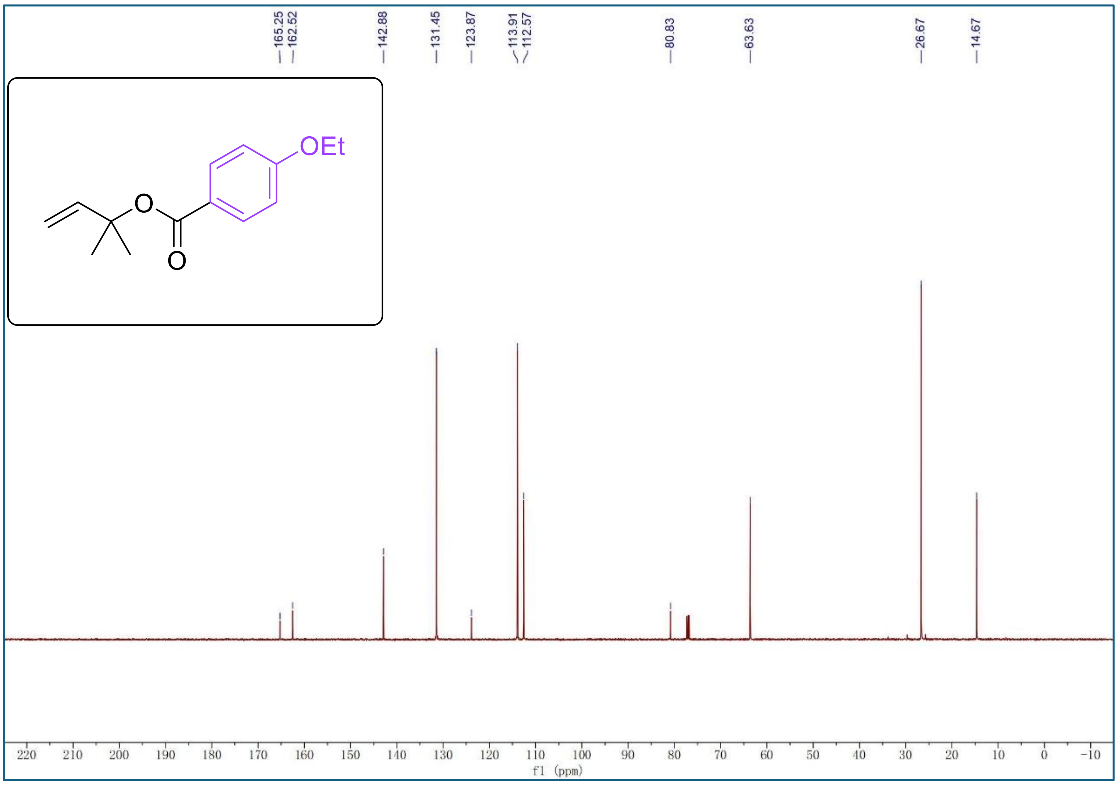

^{13}C NMR-spectrum (126 MHz, Chloroform-*d*) of **2j**

methyl (2-methylbut-3-en-2-yl) phthalate (2k)

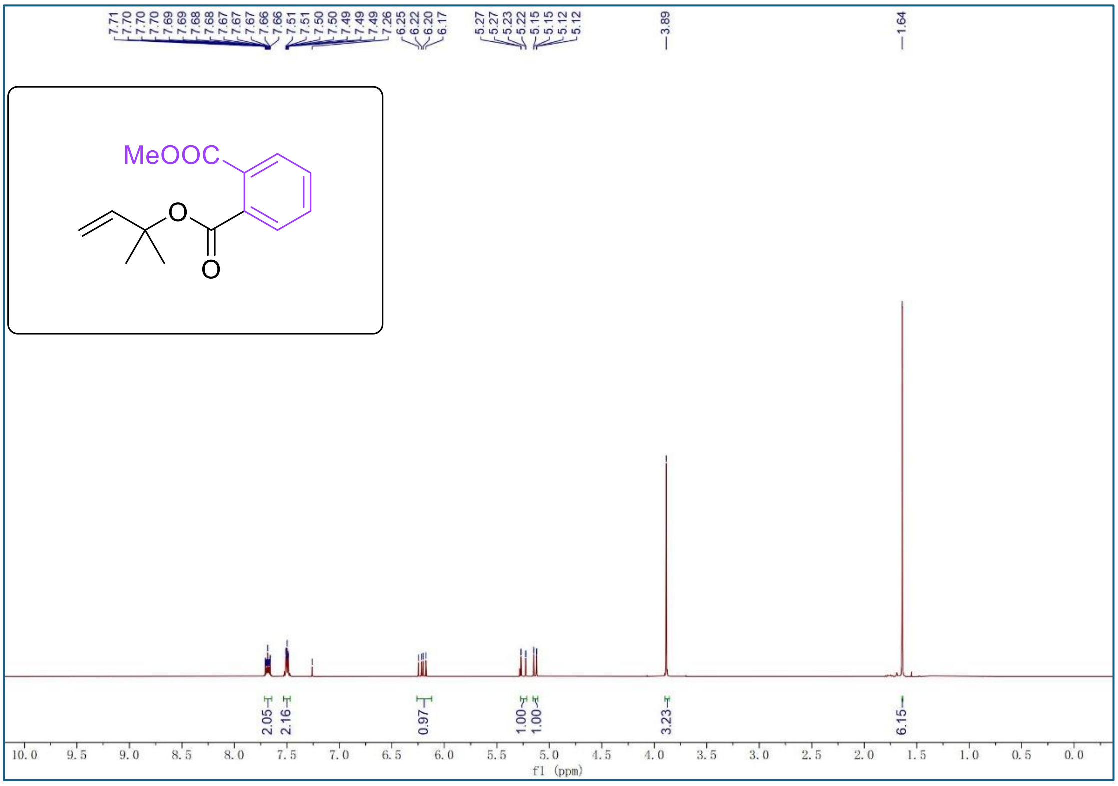

^{1}H NMR-spectrum (400 MHz, Chloroform-*d*) of **2k**

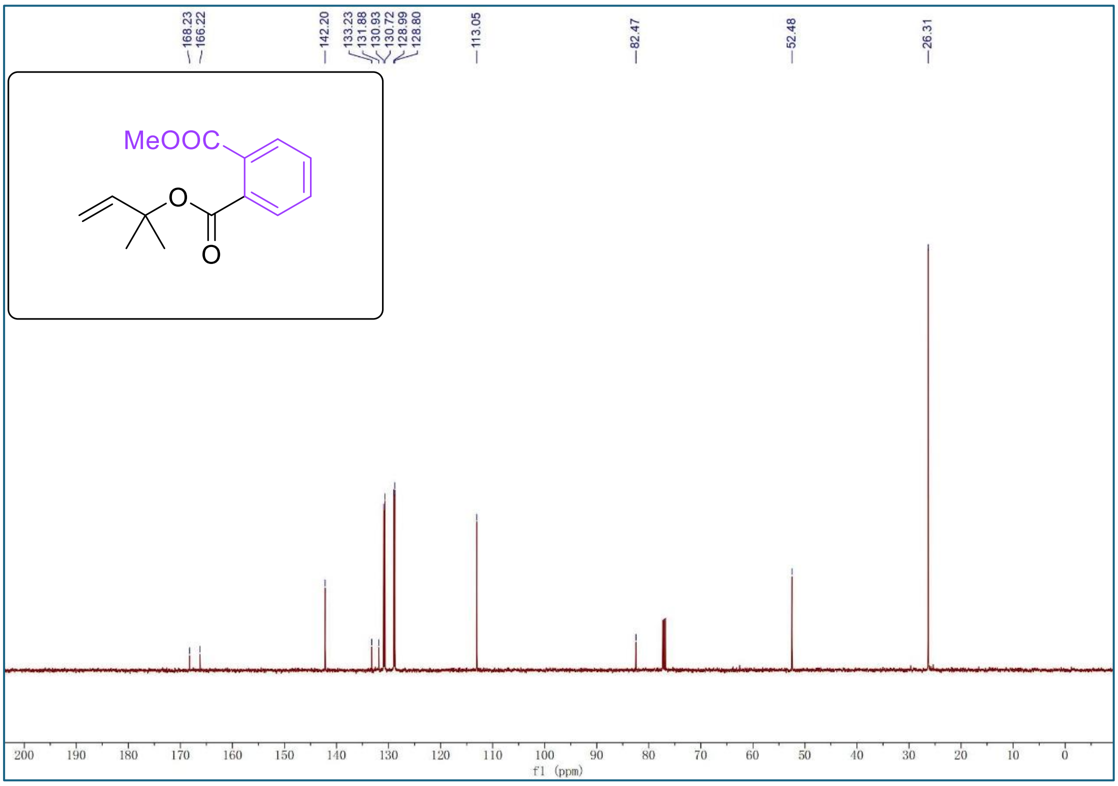

^{13}C NMR-spectrum (126 MHz, Chloroform-*d*) of **2k**

2-methylbut-3-en-2-yl 3,5-dimethylbenzoate (2l)

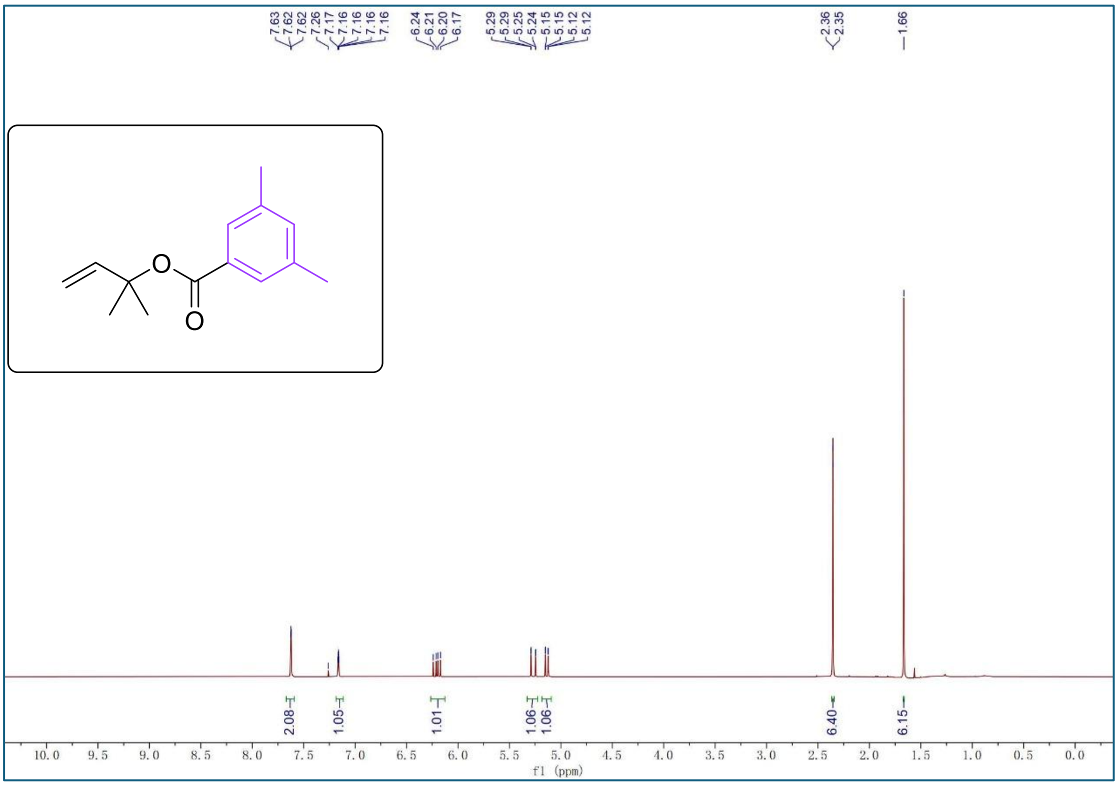

^{1}H NMR-spectrum (500 MHz, Chloroform-*d*) of **2l**

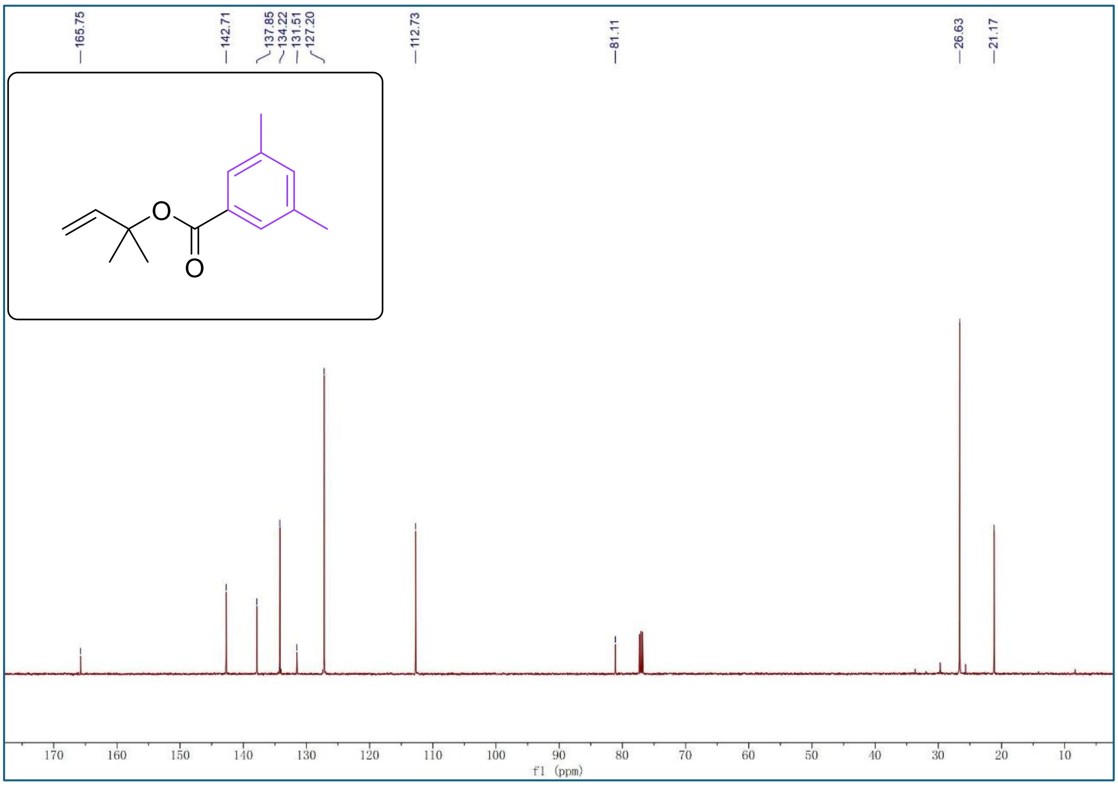

^{13}C NMR-spectrum (126 MHz, Chloroform-*d*) of **2l**

2-methylbut-3-en-2-yl 2-chloro-5-fluorobenzoate (2m)

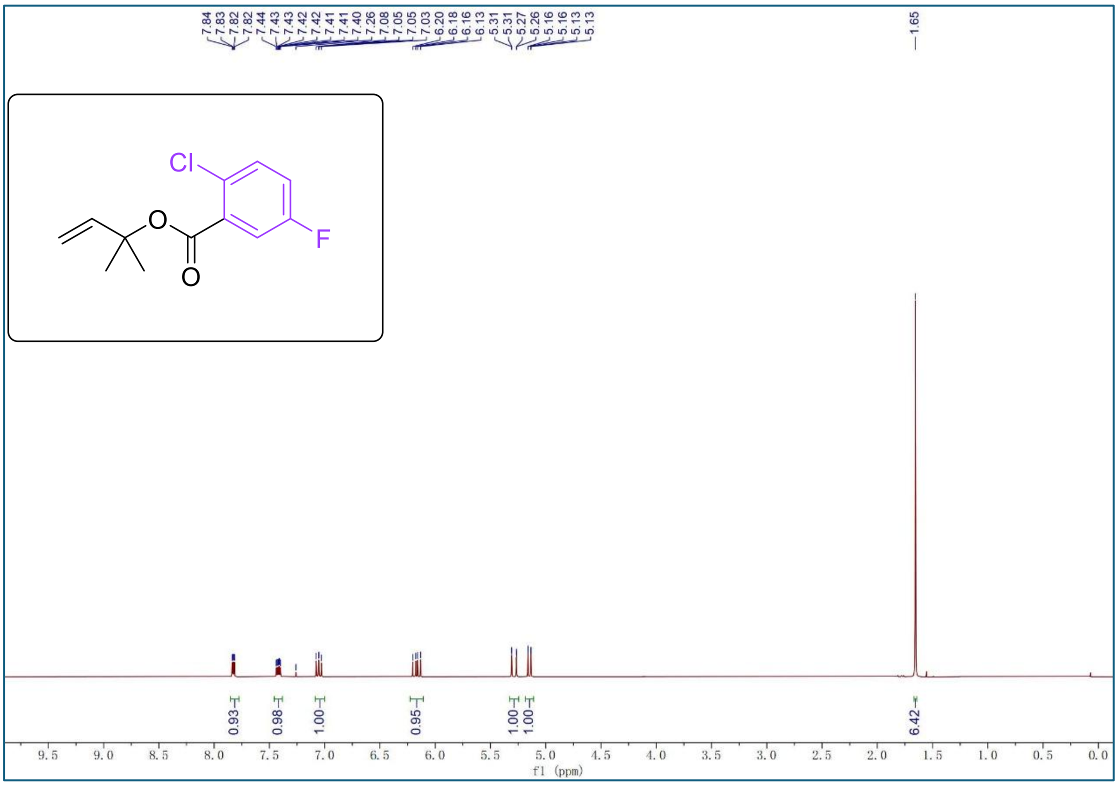

^{1}H NMR-spectrum (400 MHz, Chloroform-*d*) of **2m**

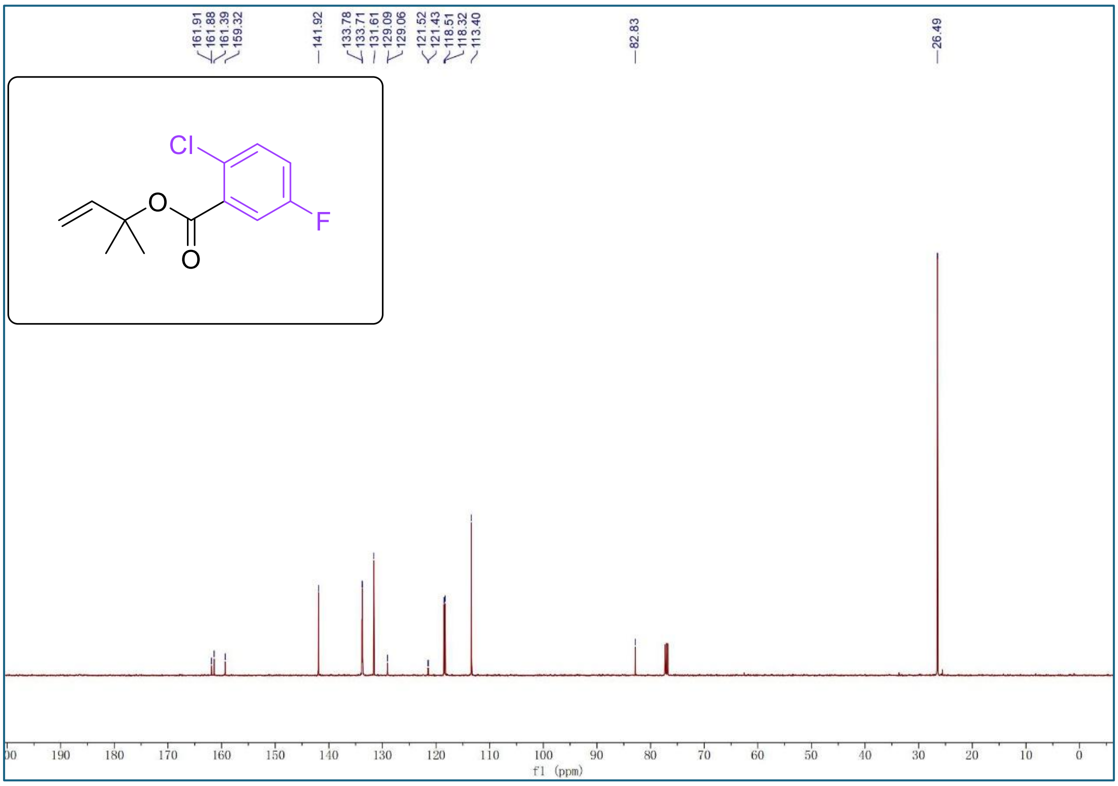

^{13}C NMR-spectrum (126 MHz, Chloroform-*d*) of **2m**

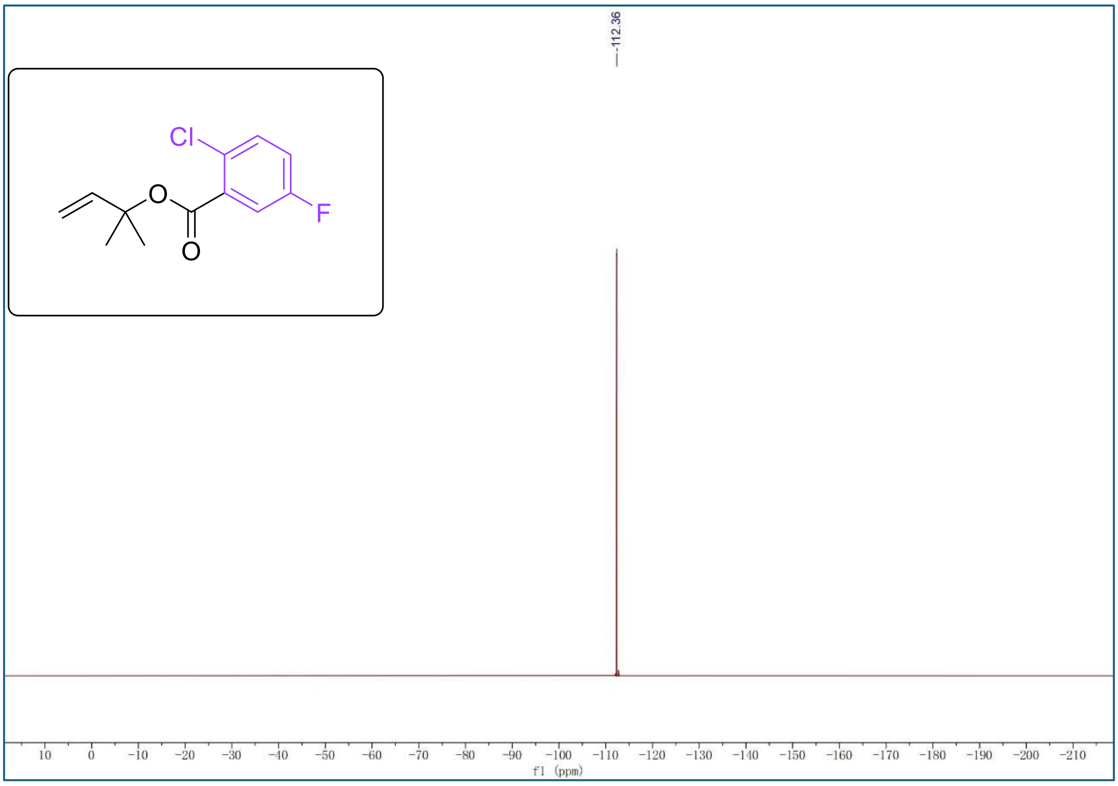

^{19}F NMR-spectrum (377 MHz, Chloroform-*d*) of **2m**

2-methylbut-3-en-2-yl 3,4-dichlorobenzoate (2n)

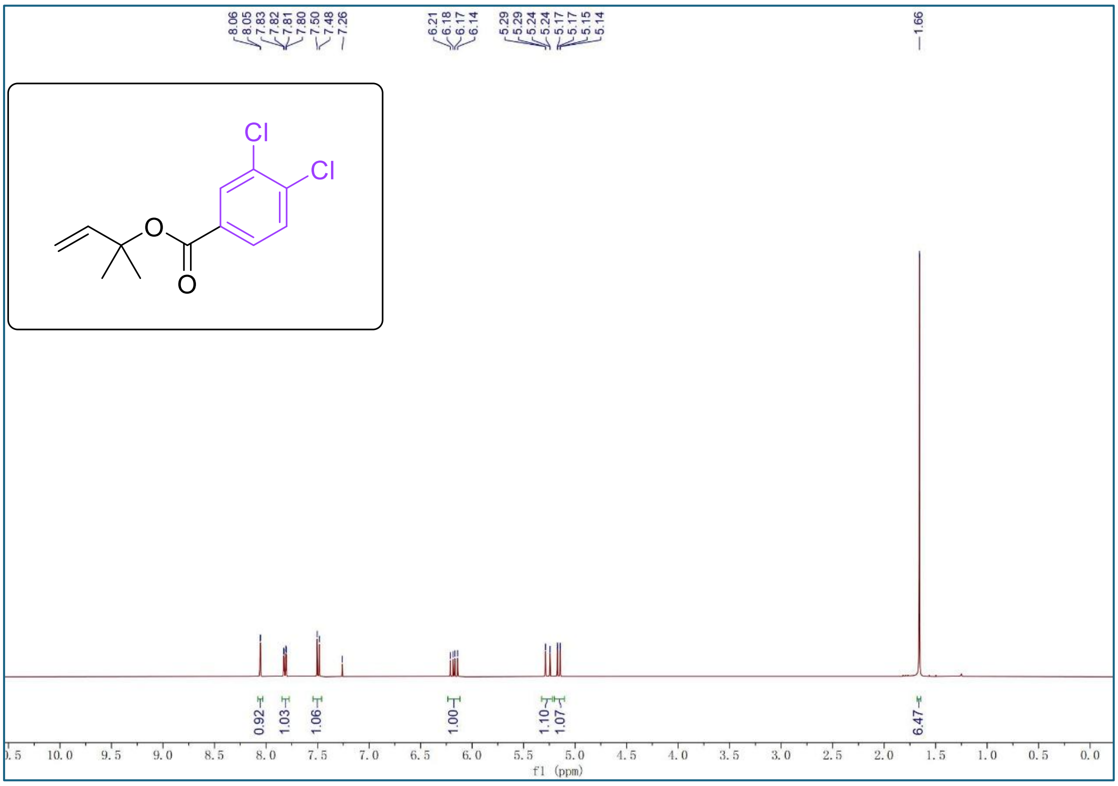

^{1}H NMR-spectrum (400 MHz, Chloroform-*d*) of **2n**

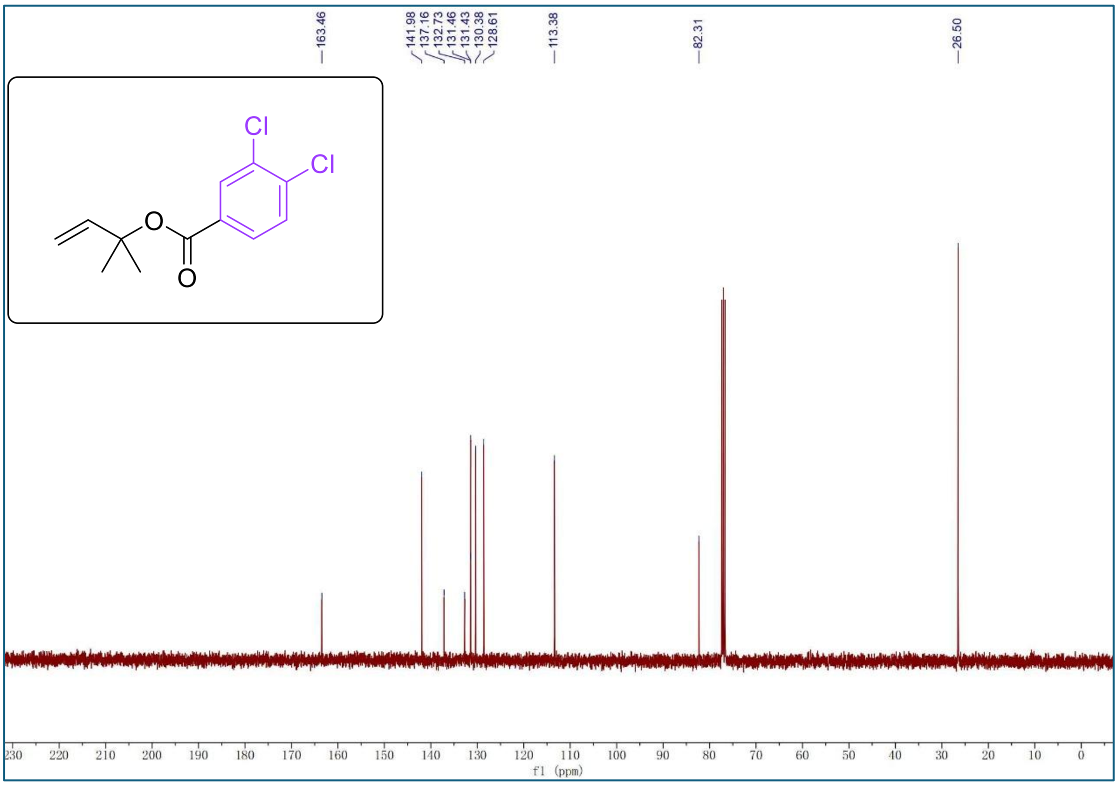

^{13}C NMR-spectrum (101 MHz, Chloroform-*d*) of **2n**

2-methylbut-3-en-2-yl 3,4-difluorobenzoate (2o)

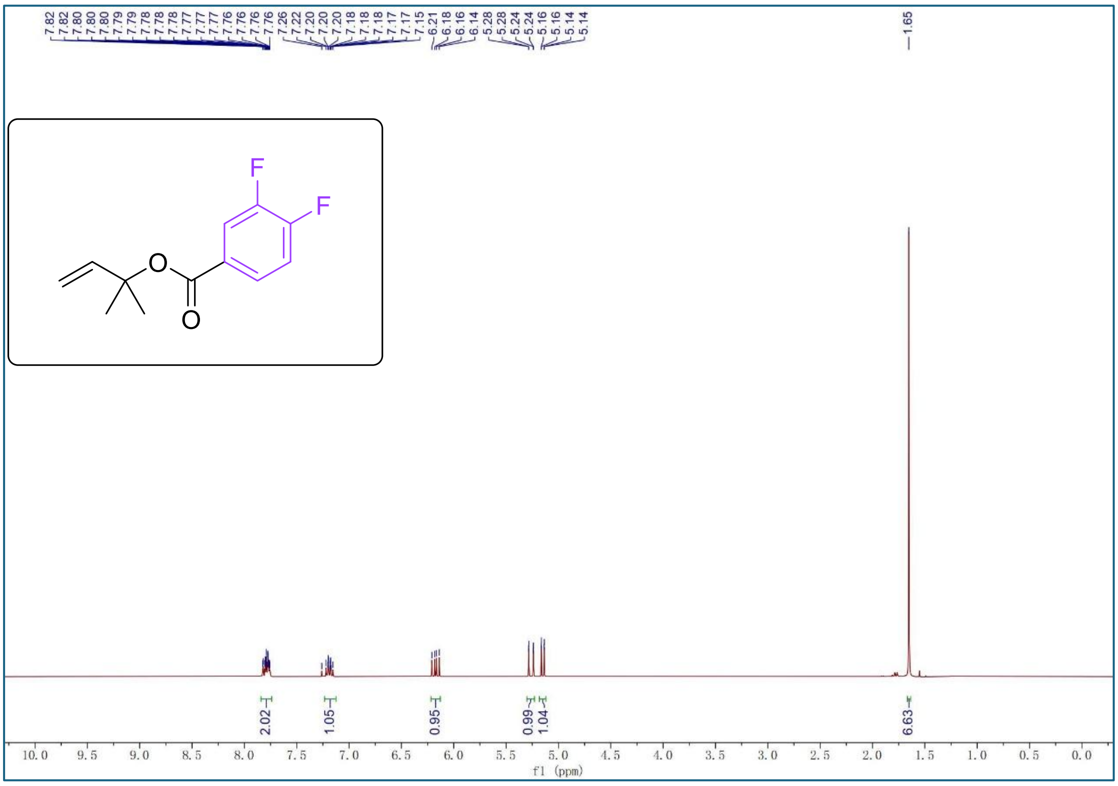

^{1}H NMR-spectrum (400 MHz, Chloroform-*d*) of **2o**

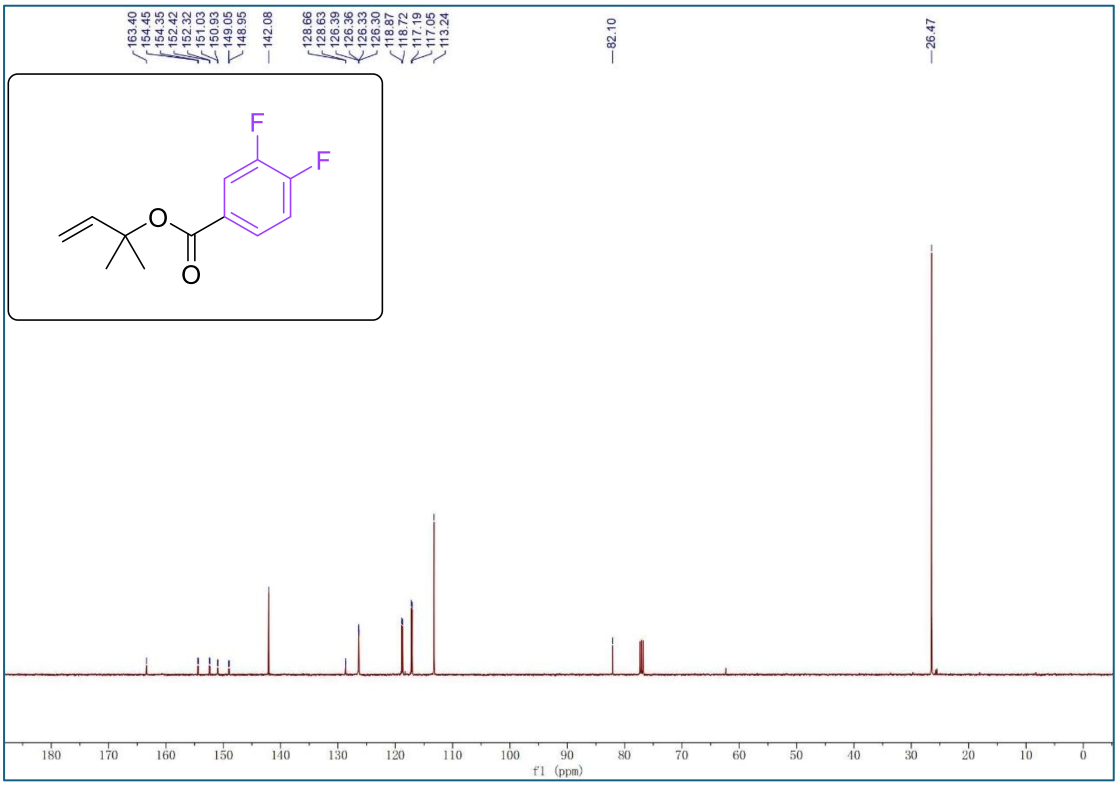

^{13}C NMR-spectrum (126 MHz, Chloroform-*d*) of **2o**

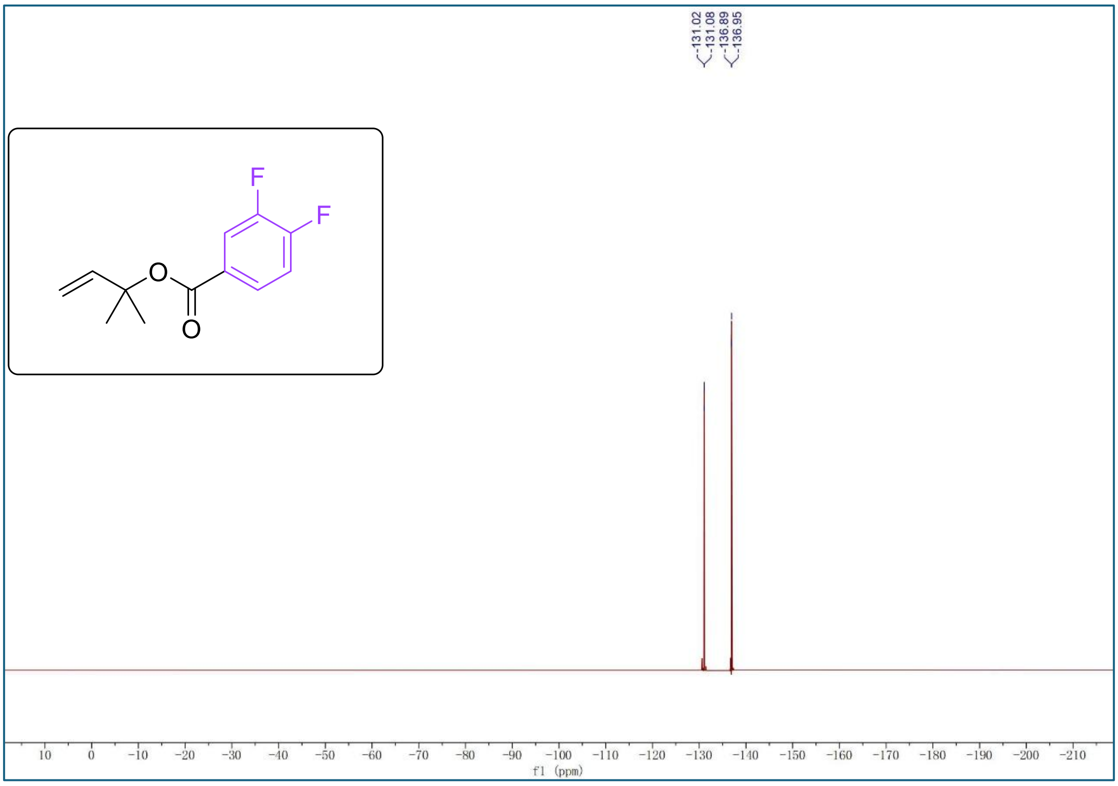

^{19}F NMR-spectrum (377 MHz, Chloroform-*d*) of **2o**

2-methylbut-3-en-2-yl 4-chloro-3-methylbenzoate (2p)

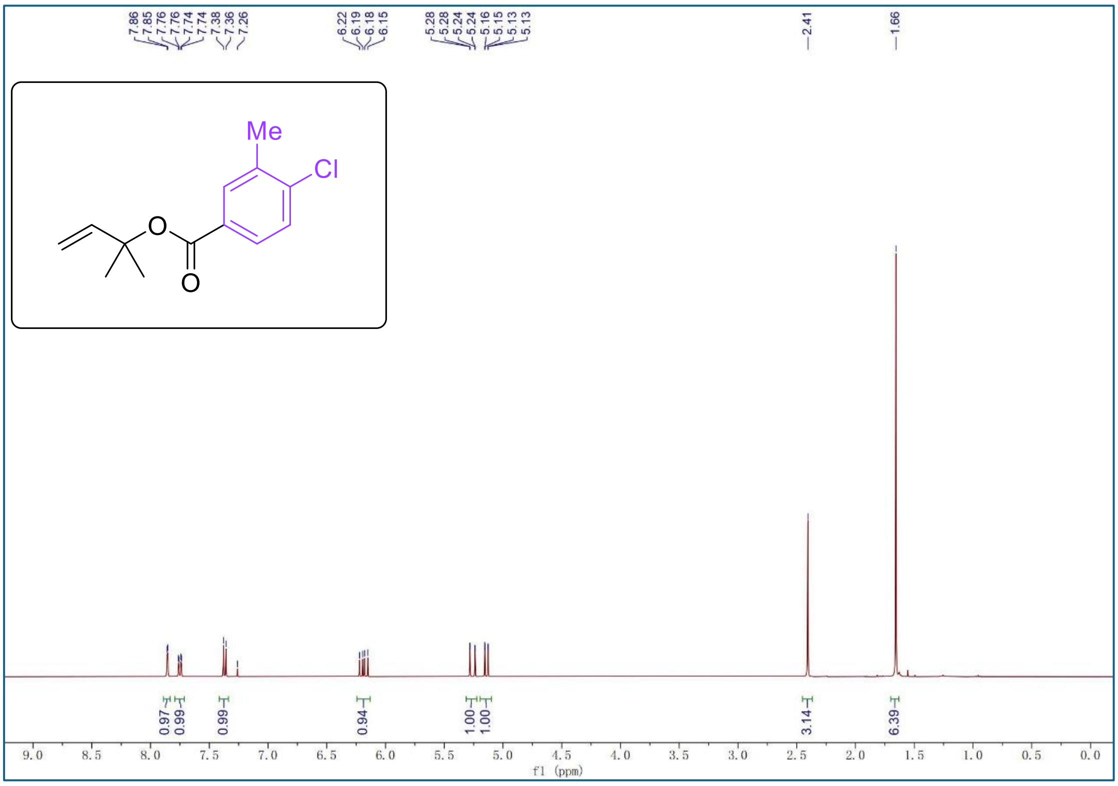

^{1}H NMR-spectrum (400 MHz, Chloroform-*d*) of **2p**

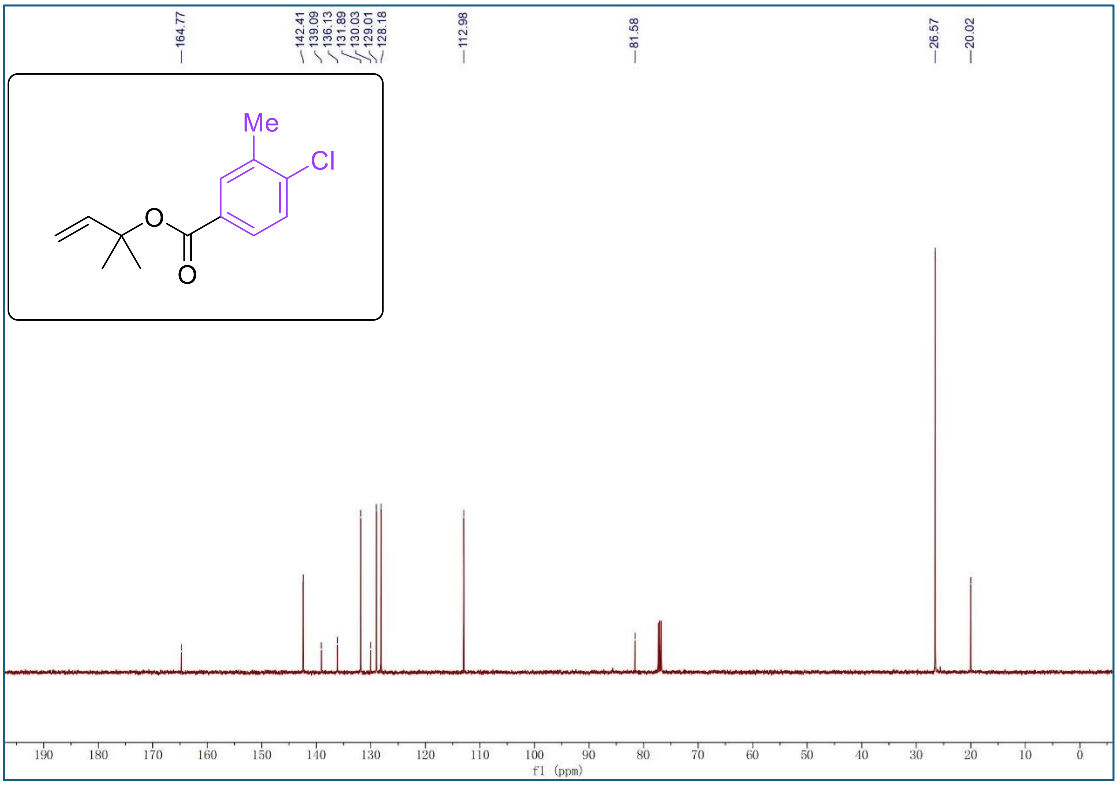

^{13}C NMR-spectrum (126 MHz, Chloroform-*d*) of **2p**

2-methylbut-3-en-2-yl 2-chloro-5-(trifluoromethyl)benzoate (2q)

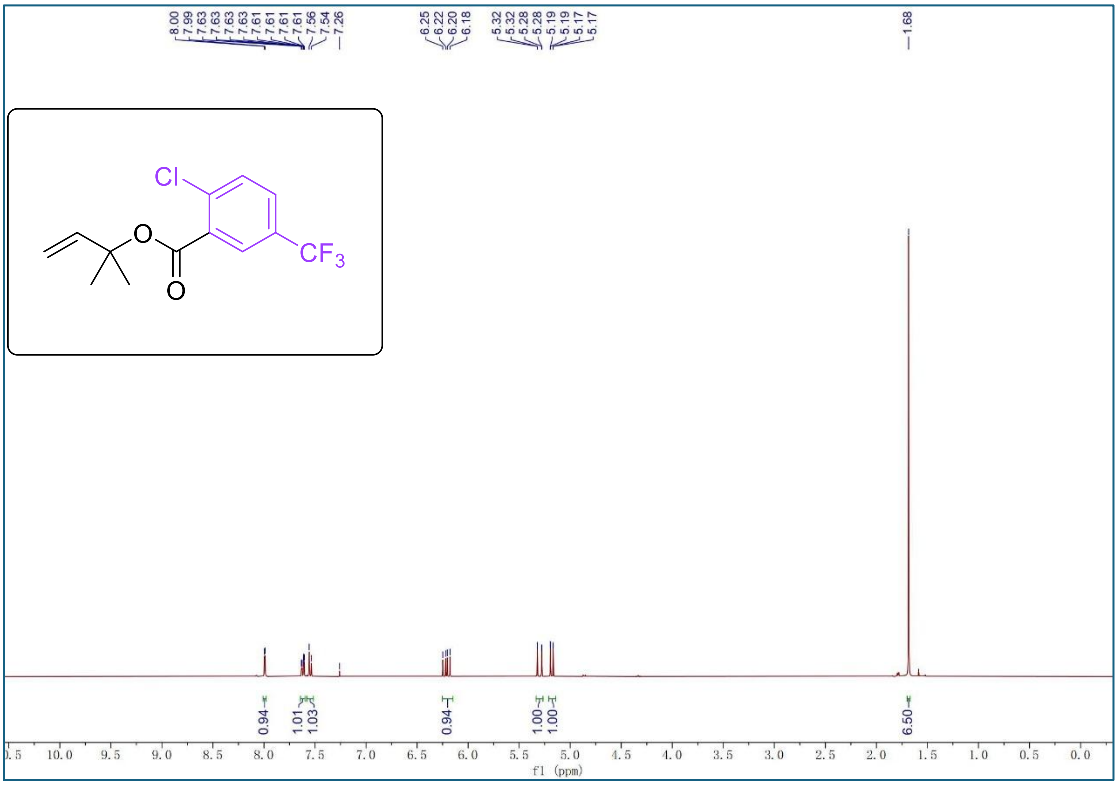

^{1}H NMR-spectrum (400 MHz, Chloroform-*d*) of **2q**

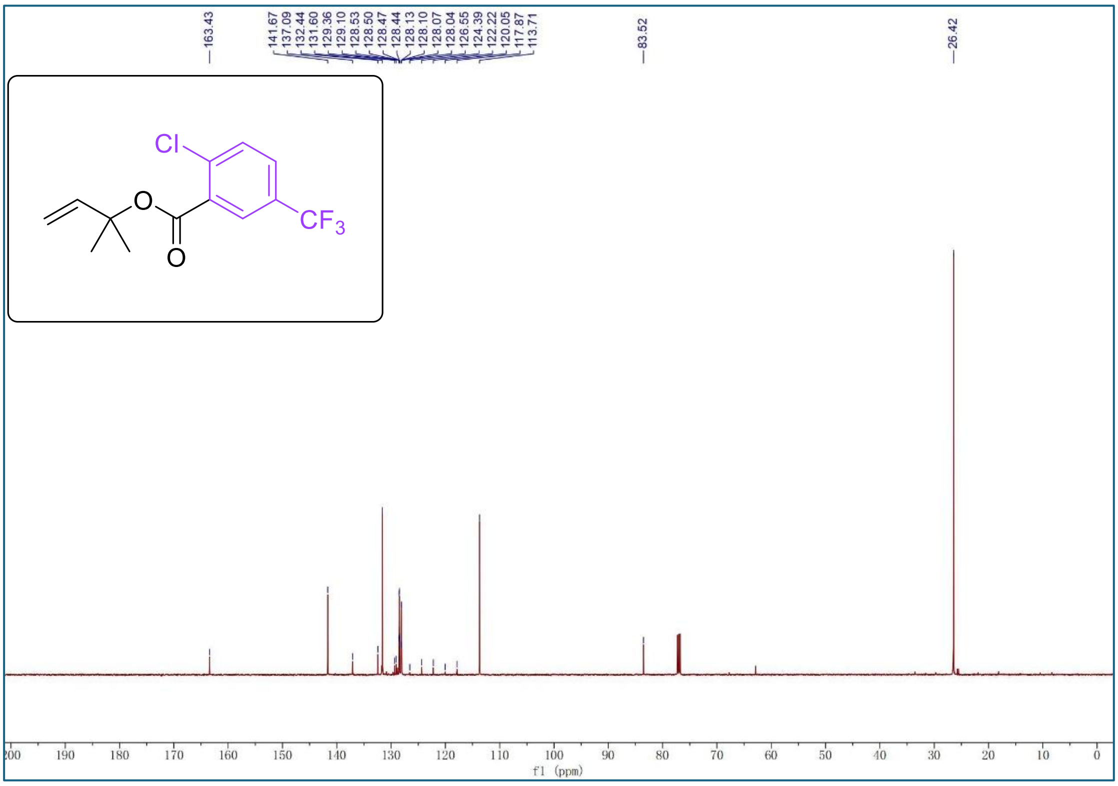

^{13}C NMR-spectrum (126 MHz, Chloroform-*d*) of **2q**

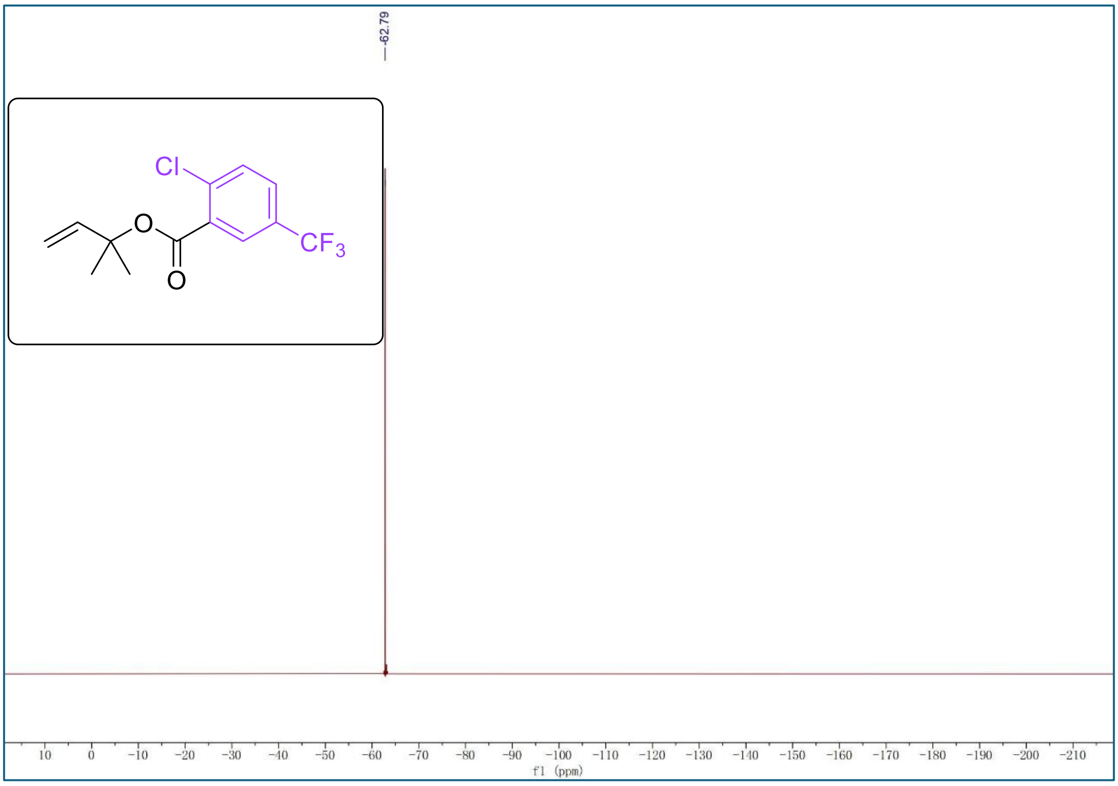

^{19}F NMR-spectrum (377 MHz, Chloroform-*d*) of **2q**

2-methylbut-3-en-2-yl 6-bromo-2-naphthoate (2r)

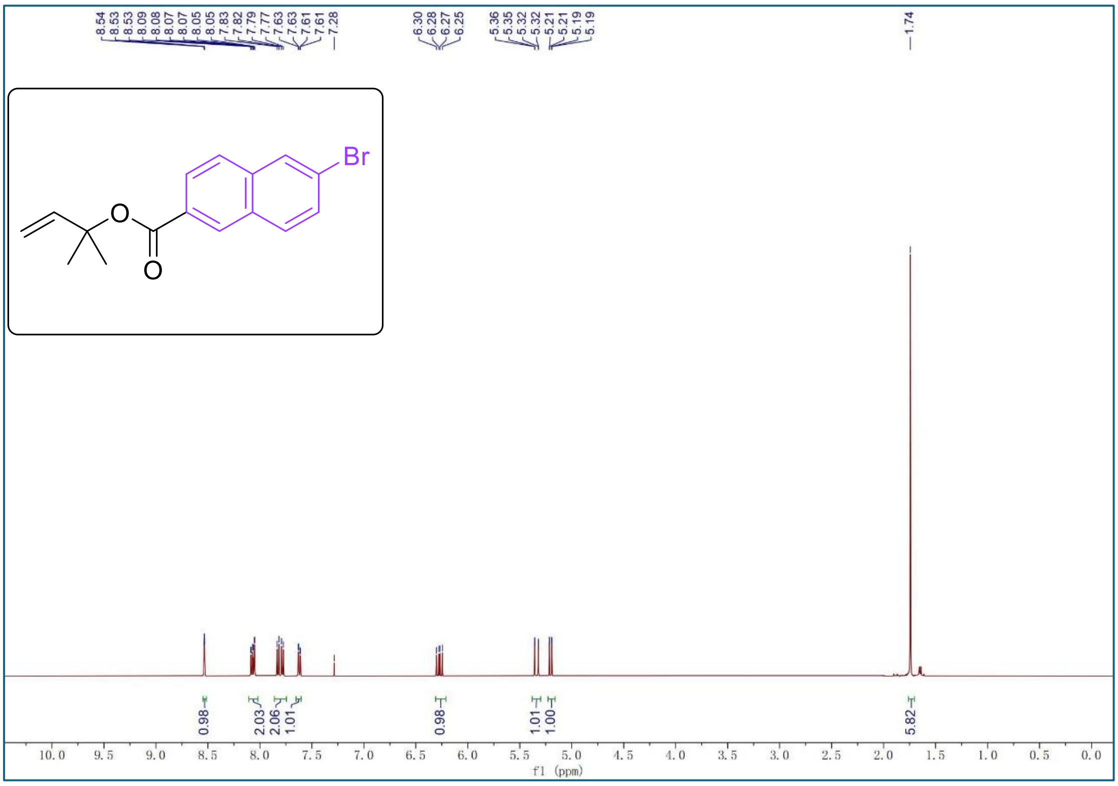

^{1}H NMR-spectrum (400 MHz, Chloroform-*d*) of **2r**

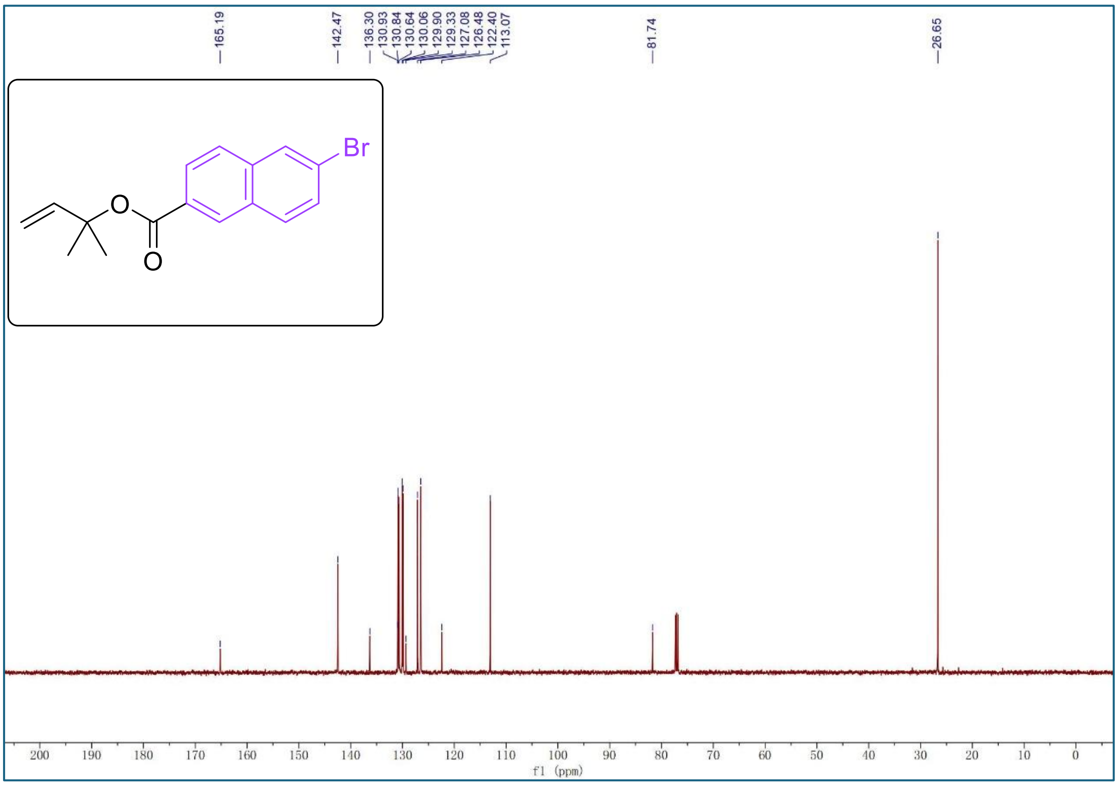

^{13}C NMR-spectrum (126 MHz, Chloroform-*d*) of **2r**

2-methylbut-3-en-2-yl thiophene-2-carboxylat (2s)

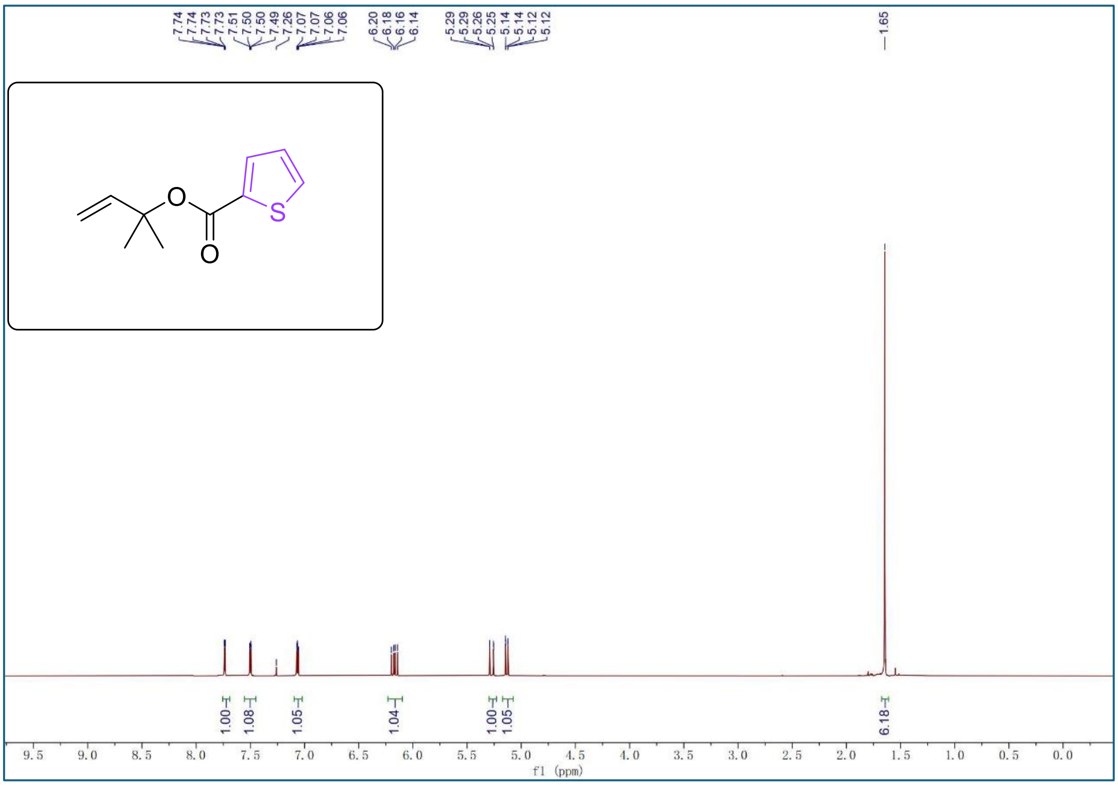

^{1}H NMR-spectrum (500 MHz, Chloroform-*d*) of **2s**

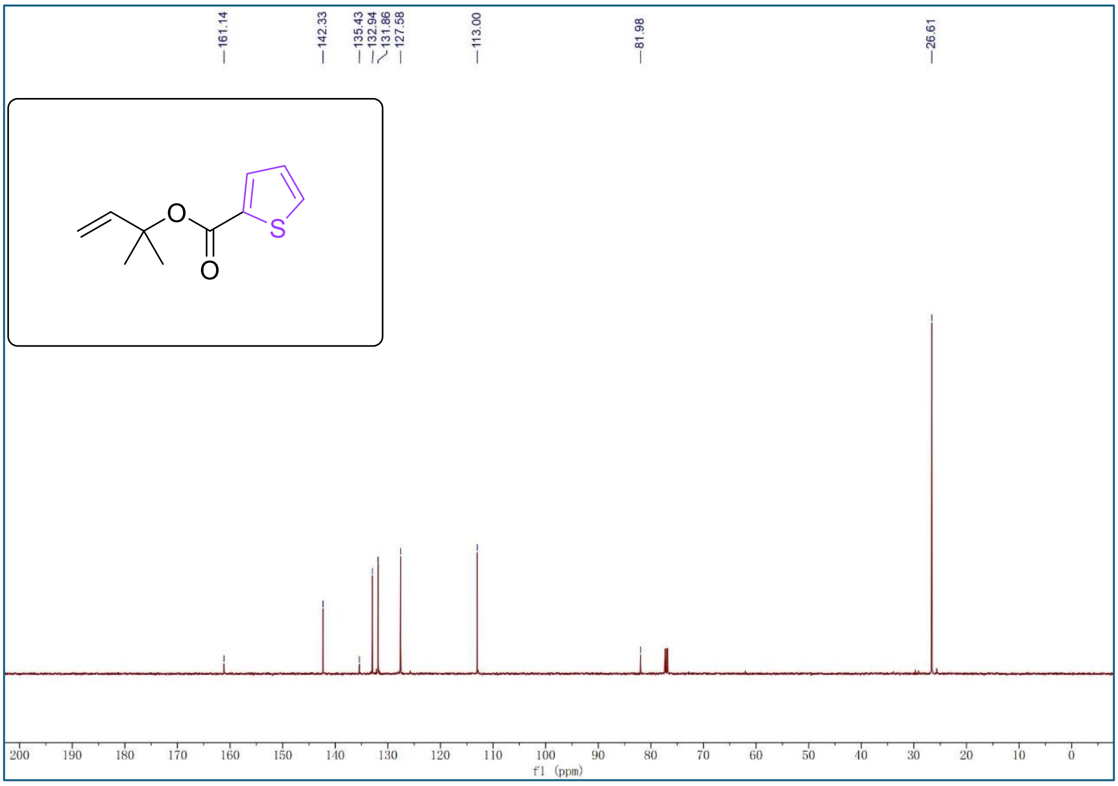

^{13}C NMR-spectrum (126 MHz, Chloroform-*d*) of **2s**

2-ethyl 4-(2-methylbut-3-en-2-yl) thiophene-2,4-dicarboxylate (2t)

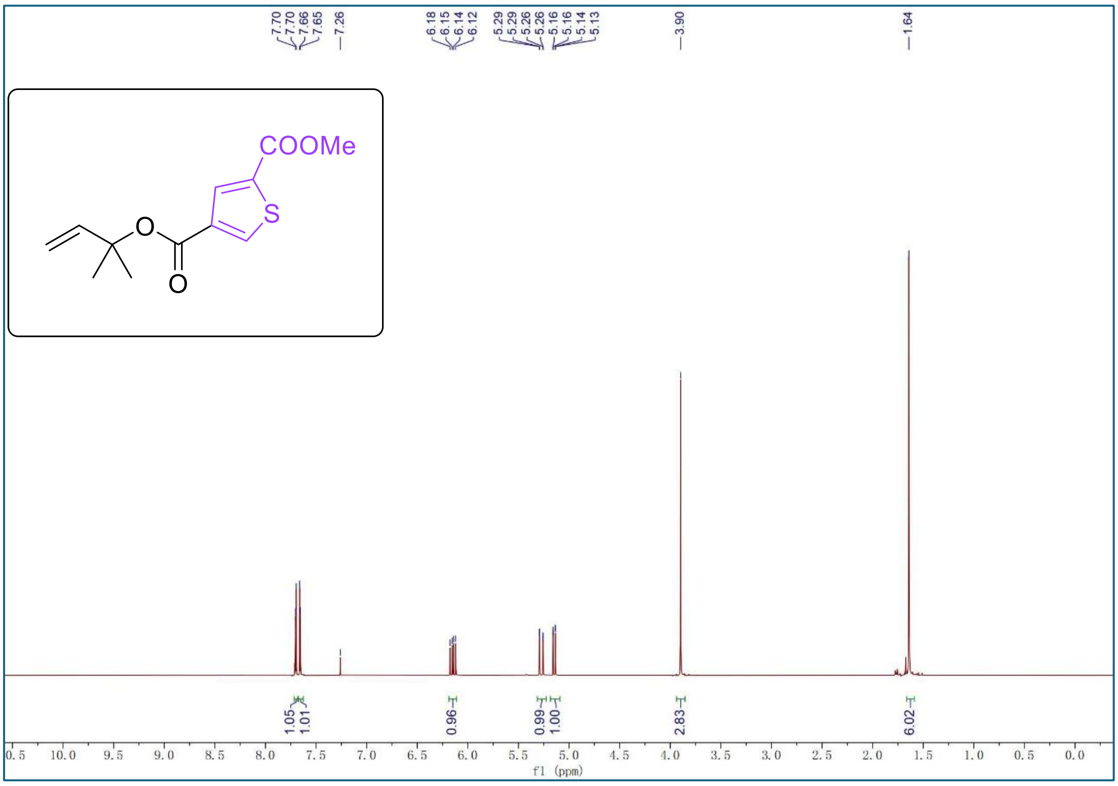

^{1}H NMR-spectrum (500 MHz, Chloroform-*d*) of **2t**

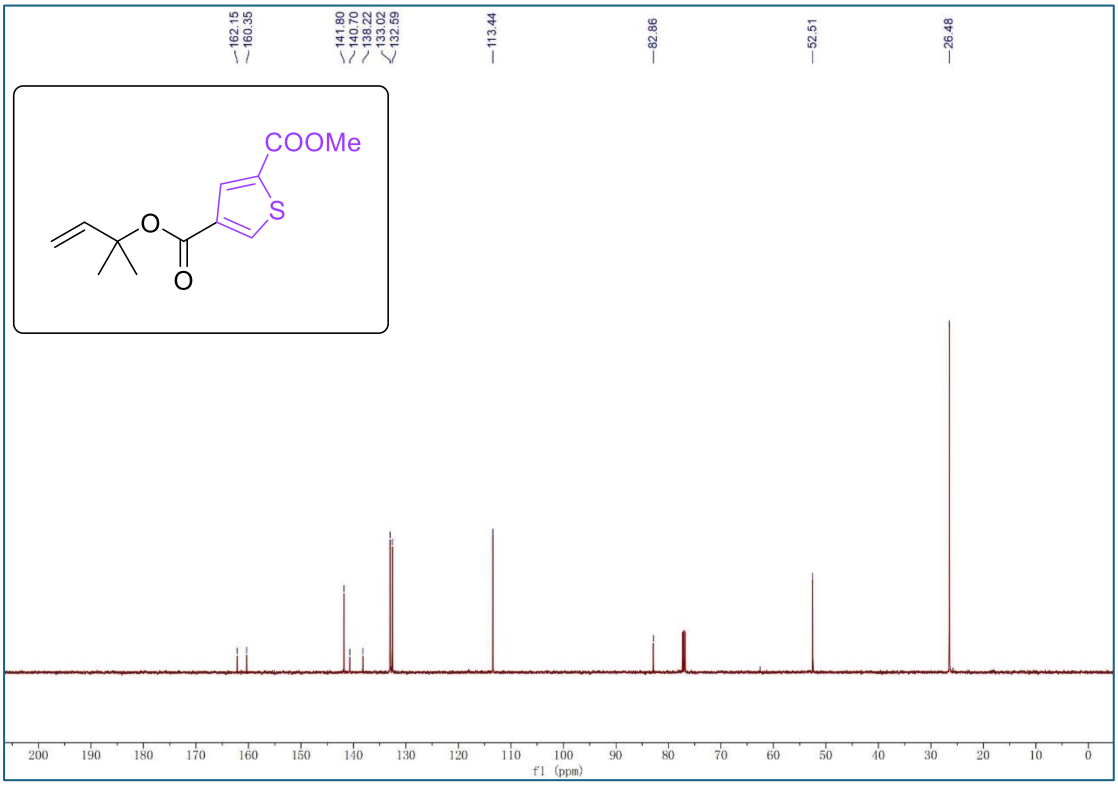

^{13}C NMR-spectrum (126 MHz, Chloroform-*d*) of **2t**

2-methylbut-3-en-2-yl 9,10-dioxo-9,10-dihydroanthracene-2-carboxylate (2u)

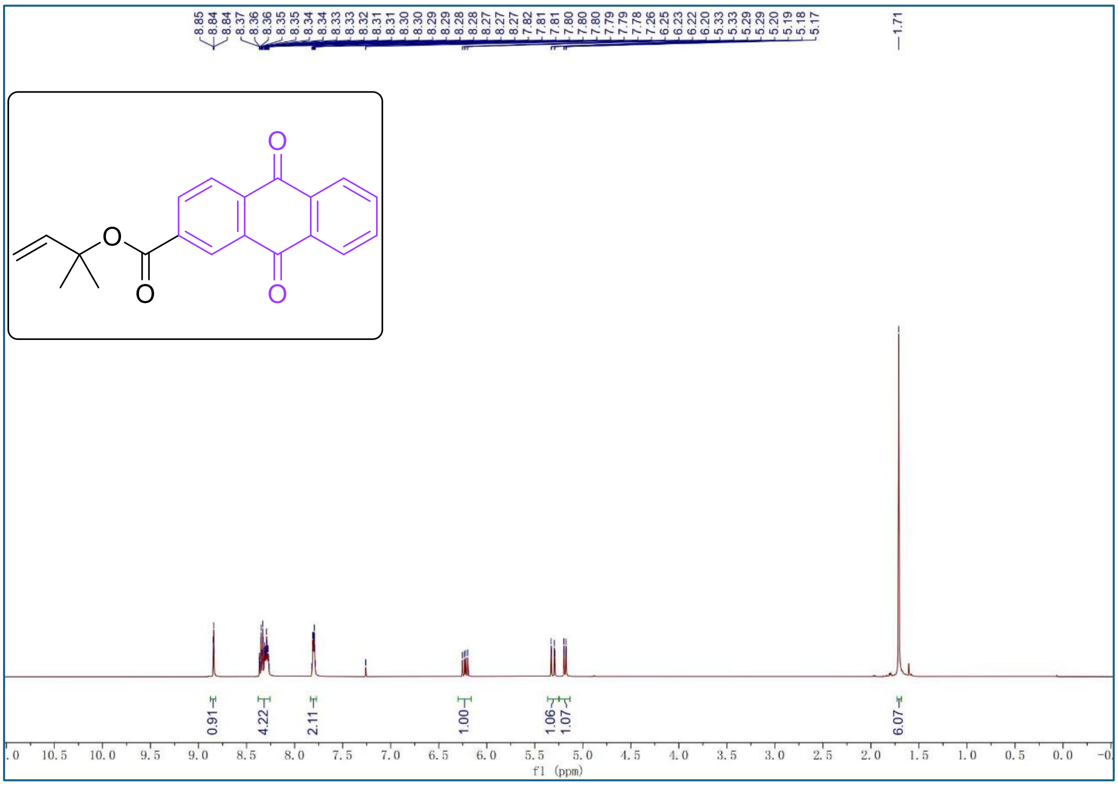

^{1}H NMR-spectrum (500 MHz, Chloroform-*d*) of **2u**

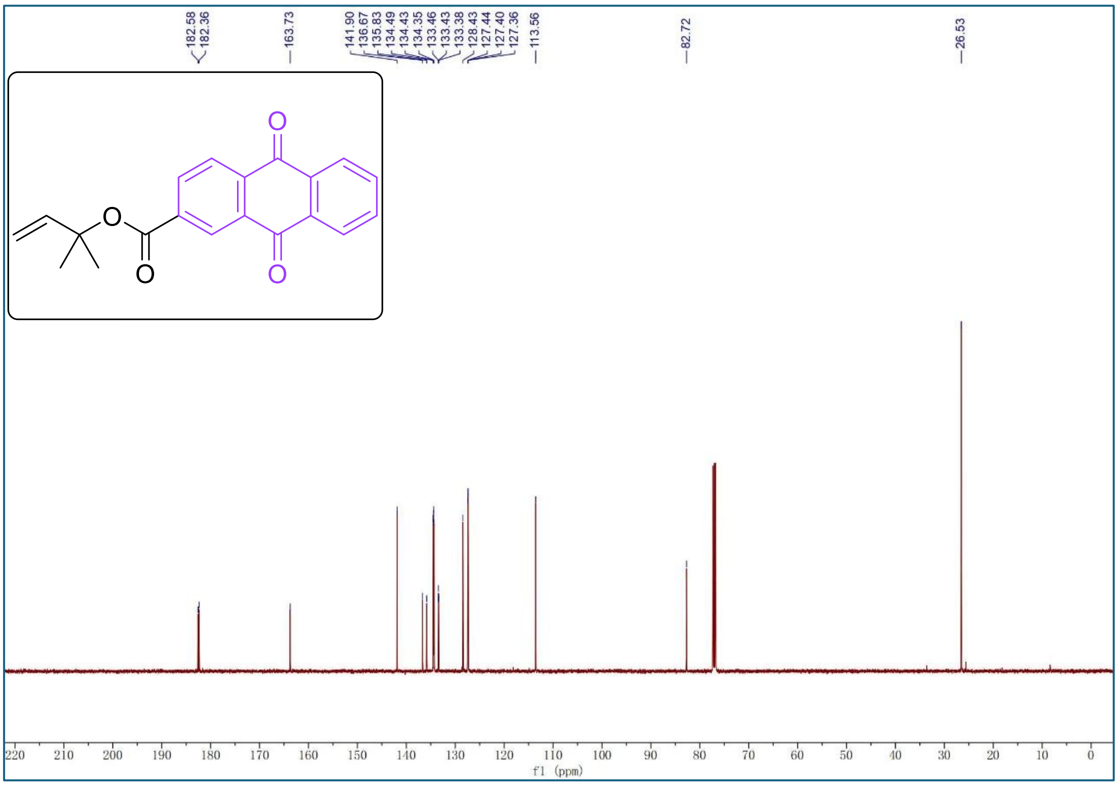

^{13}C NMR-spectrum (126 MHz, Chloroform-*d*) of **2u**

1-vinylcyclohexyl 4-(5-(2-fluorophenyl)-1,2,4-oxadiazol-3-yl)benzoate (2v)

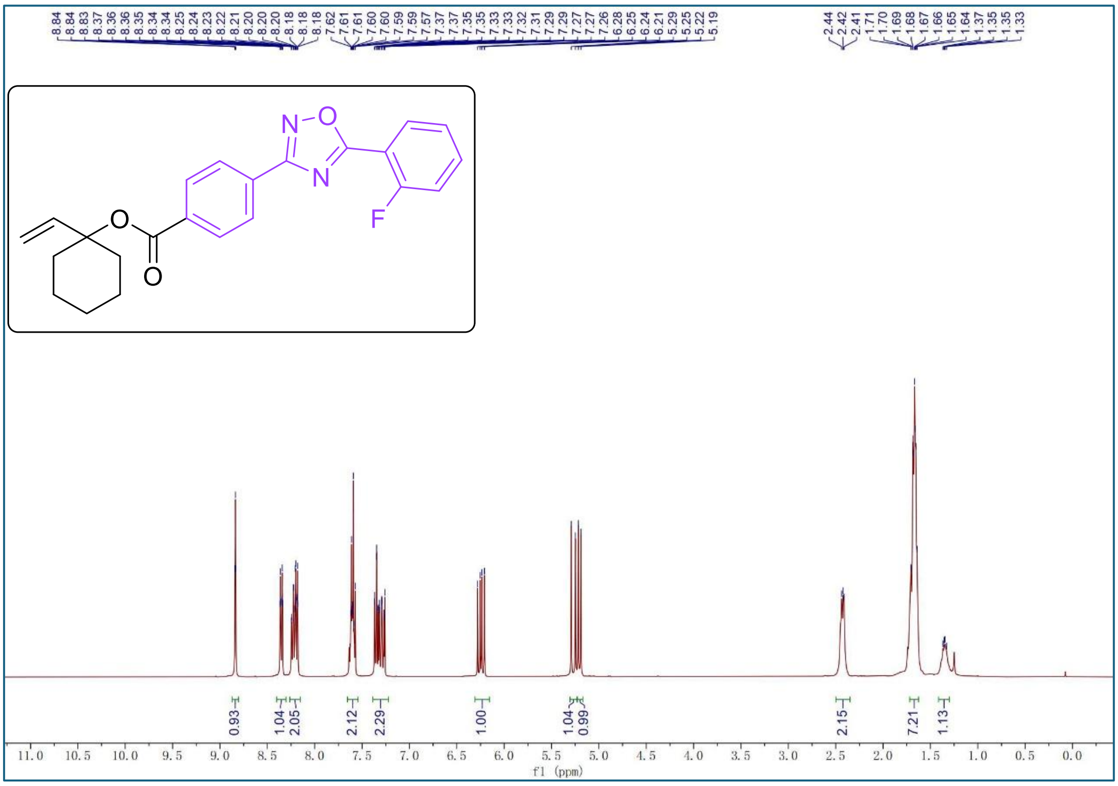

^{1}H NMR-spectrum (400 MHz, Chloroform-*d*) of **2v**

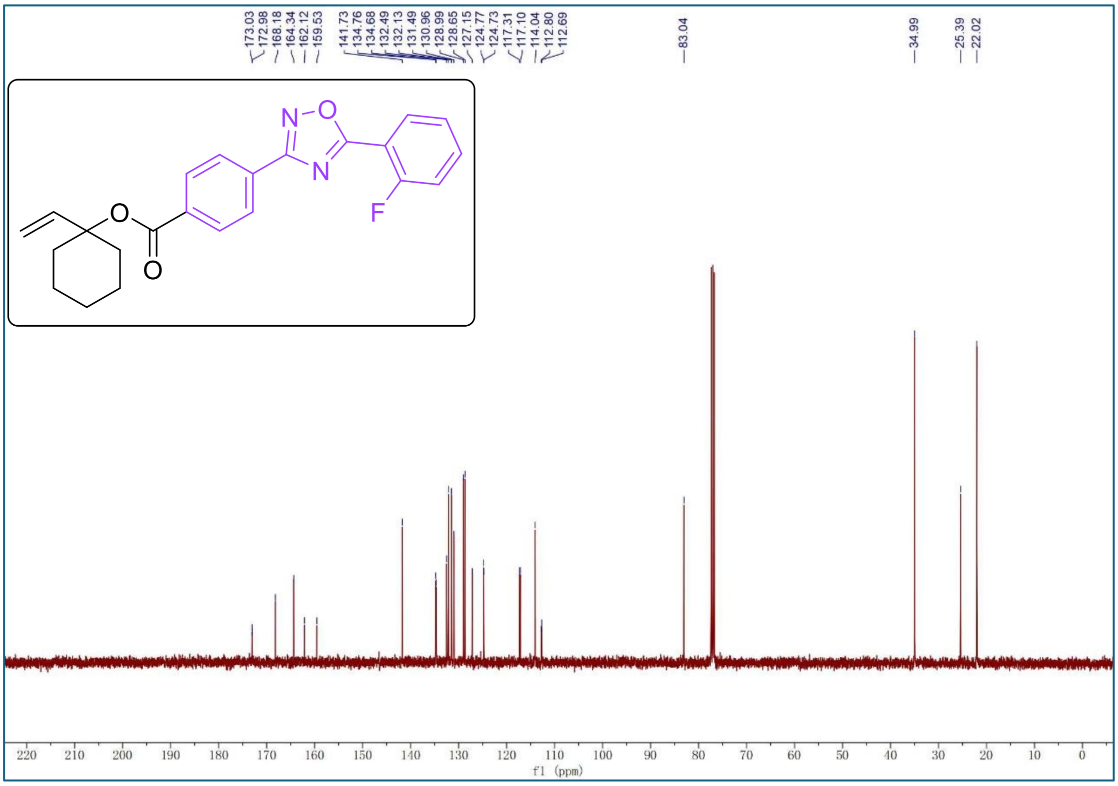

^{13}C NMR-spectrum (101 MHz, Chloroform-*d*) of **2v**

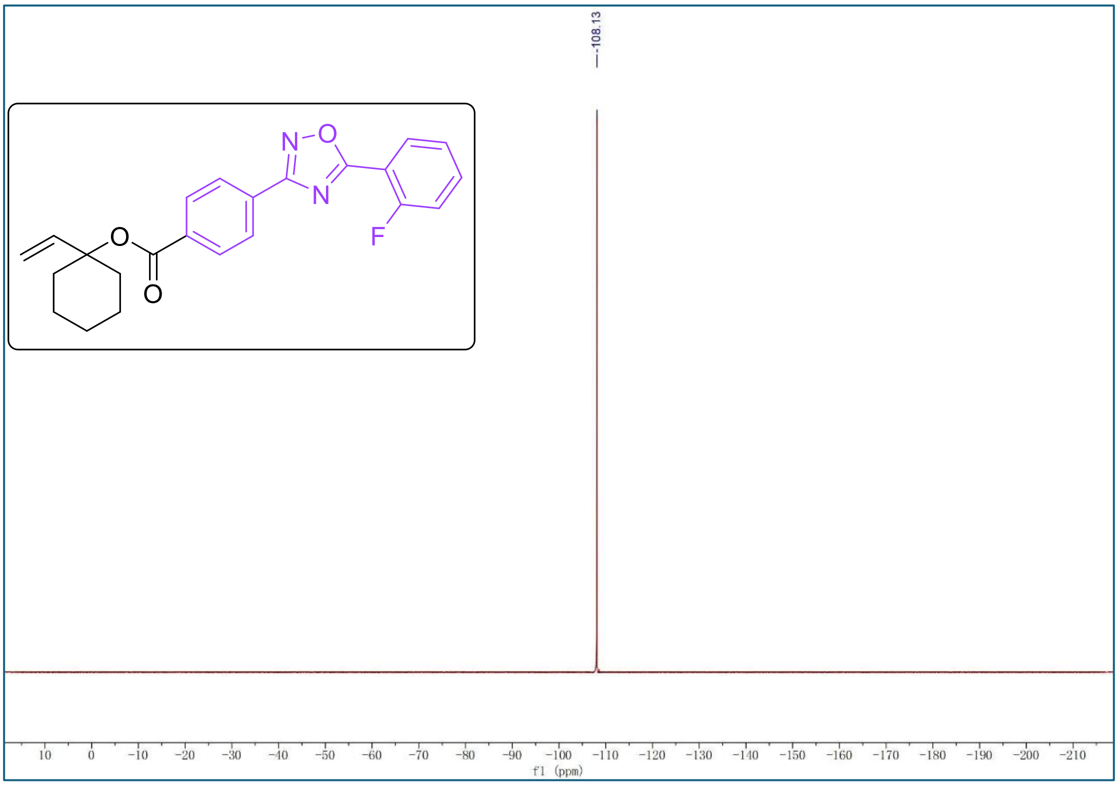

^{19}F NMR-spectrum (377 MHz, Chloroform-*d*) of **2v**

2-methylbut-3-en-2-yl 3-methyl-4-oxo-2-phenyl-4H-chromene-6-carboxylate (2w)

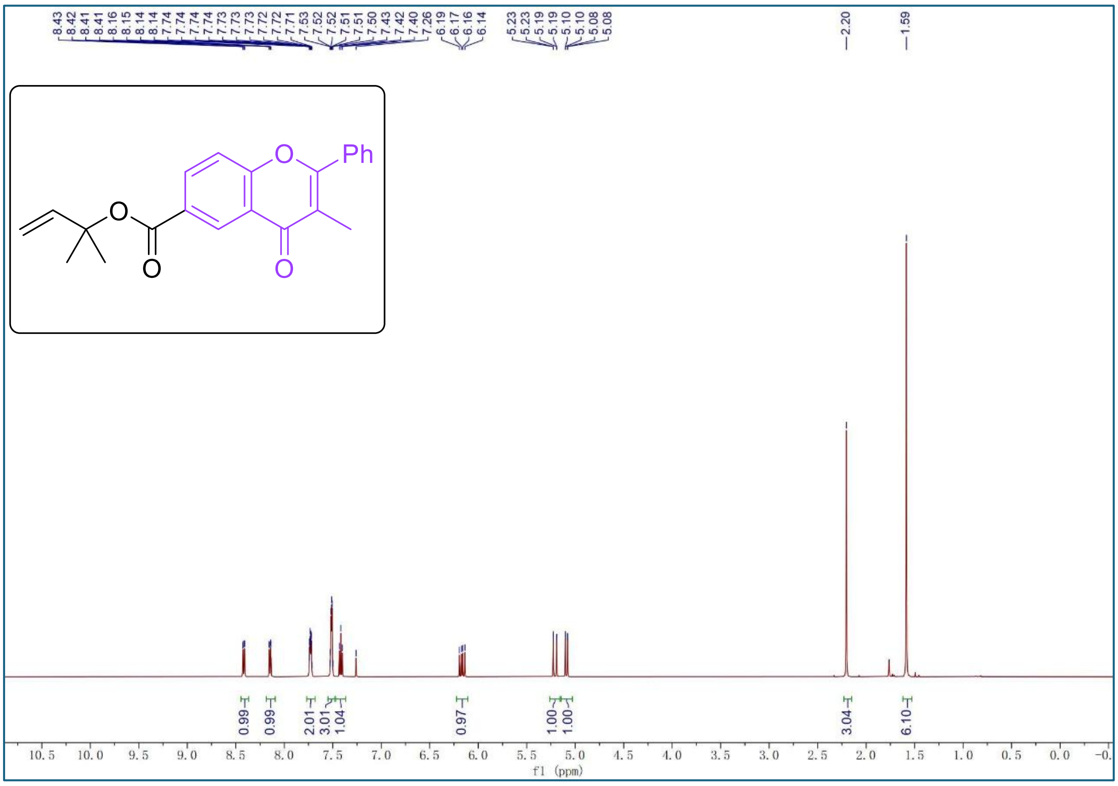

^{1}H NMR-spectrum (500 MHz, Chloroform-*d*) of **2w**

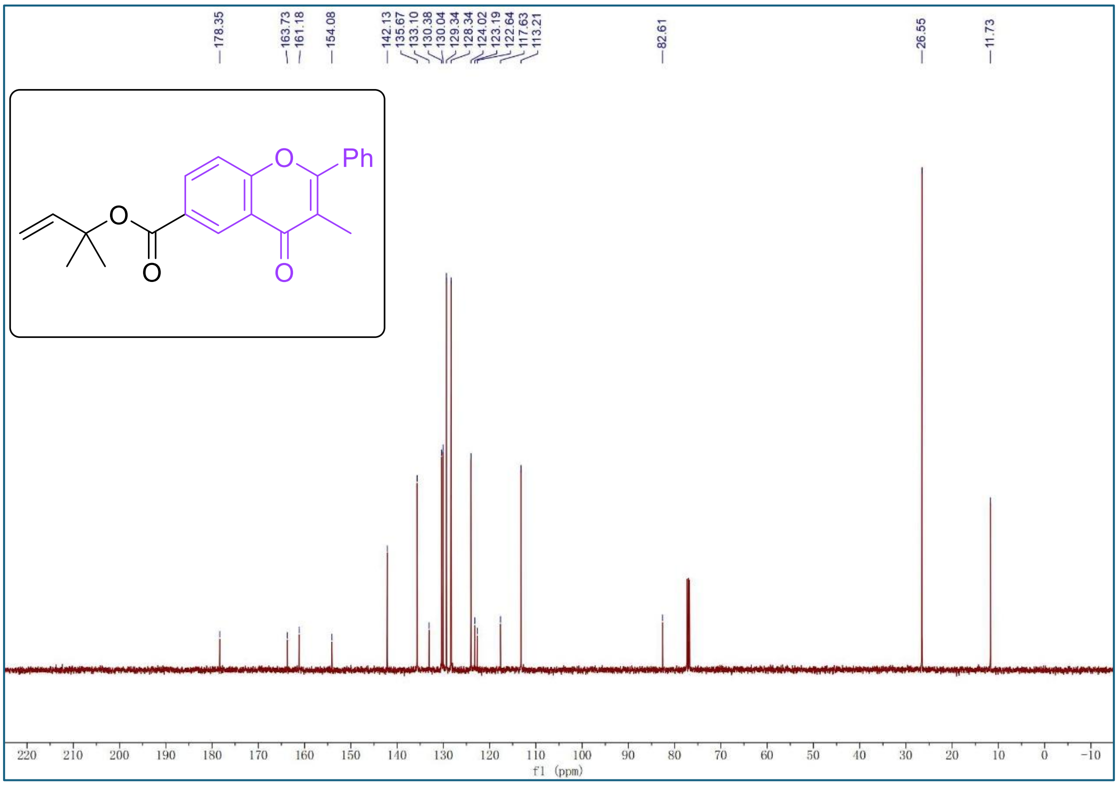

^{13}C NMR-spectrum (126 MHz, Chloroform-*d*) of **2w**

2-methylbut-3-en-2-yl 4-(*N,N*-dipropylsulfamoyl)benzoate (2x)

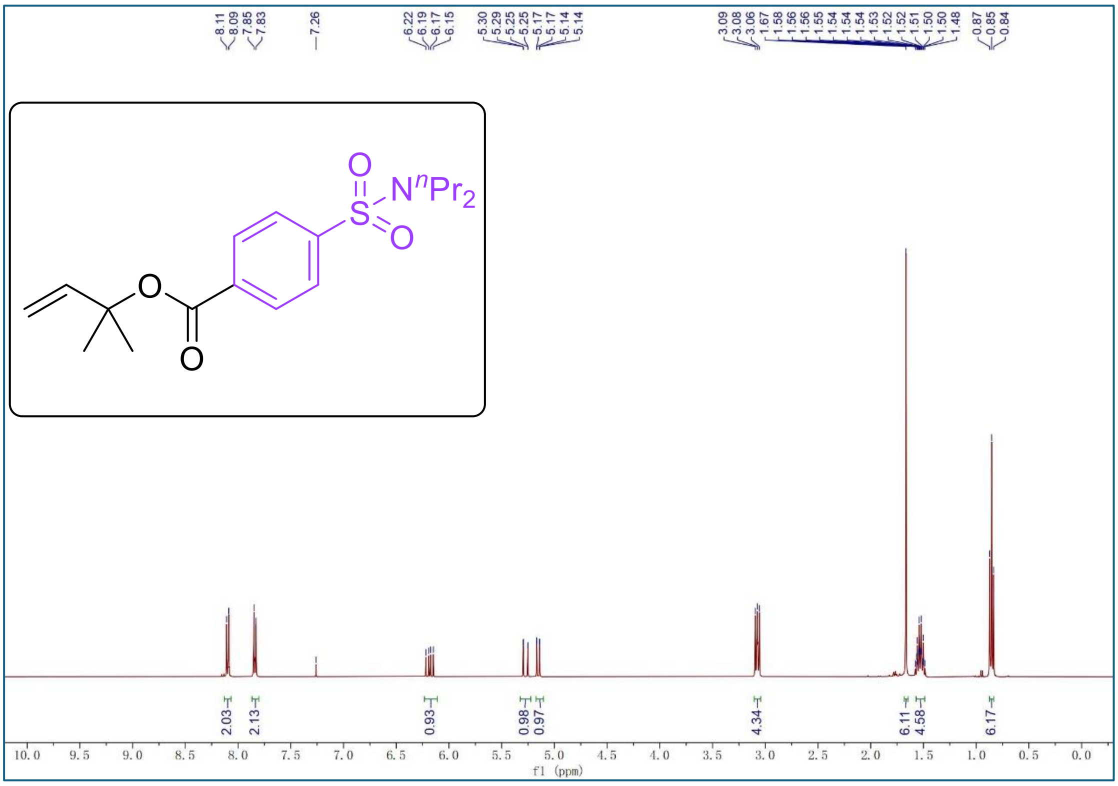

^{1}H NMR-spectrum (400 MHz, Chloroform-*d*) of **2x**

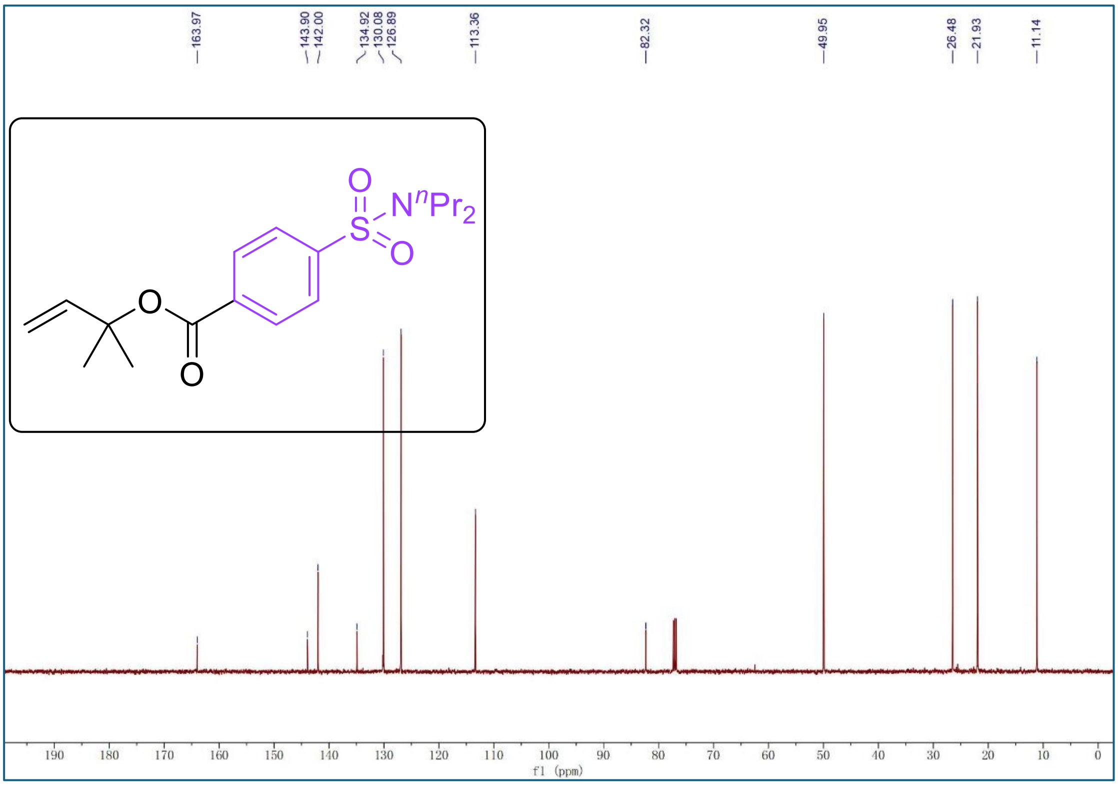

^{13}C NMR-spectrum (126 MHz, Chloroform-*d*) of **2x**

2-methylbut-3-en-2-yl 2-(3-cyano-4-isobutylphenyl)-4-methylthiazole-5-carboxylate (2y)

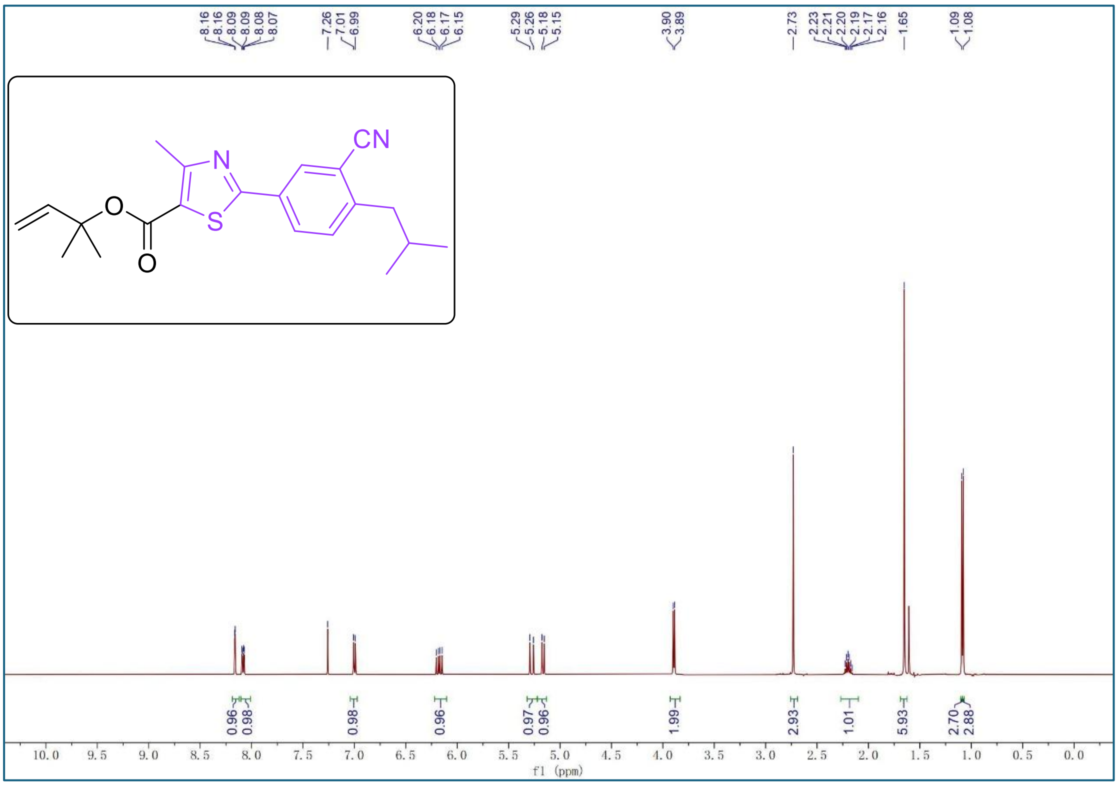

^{1}H NMR-spectrum (500 MHz, Chloroform-*d*) of **2y**

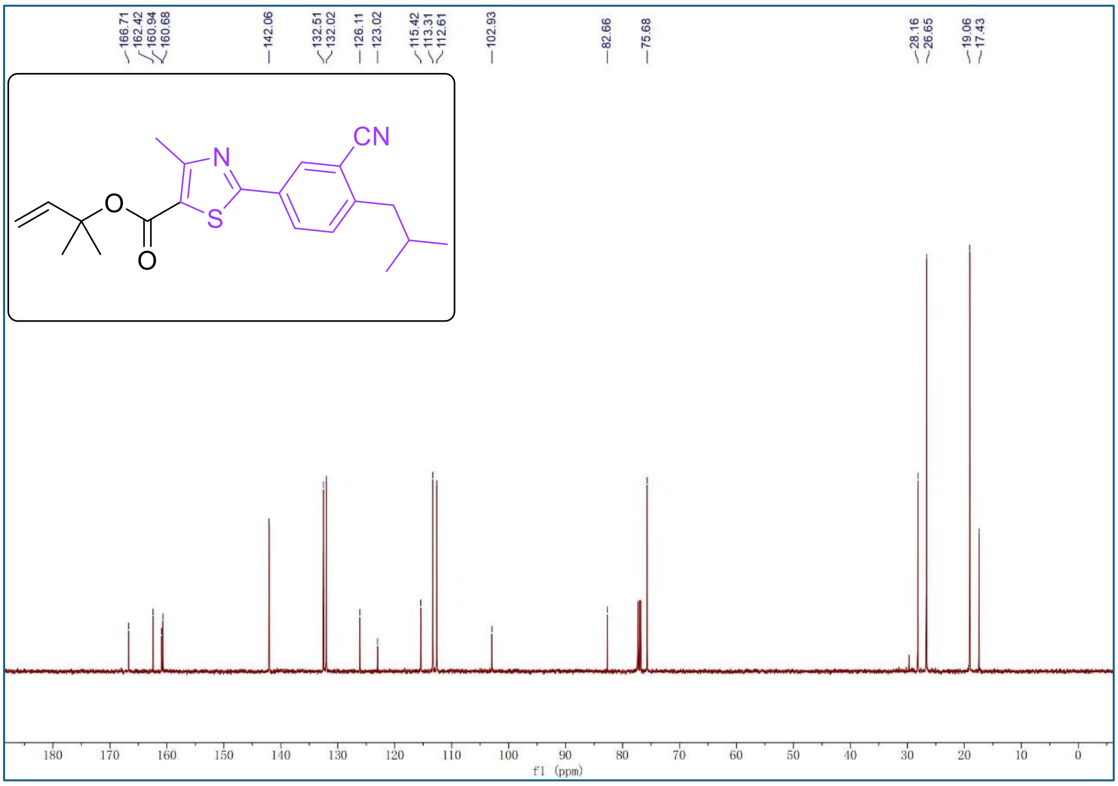

^{13}C NMR-spectrum (126 MHz, Chloroform-*d*) of **2y**

SUPPORTING INFORMATION

1-allylcyclohexyl 4-methylbenzoate (2z)

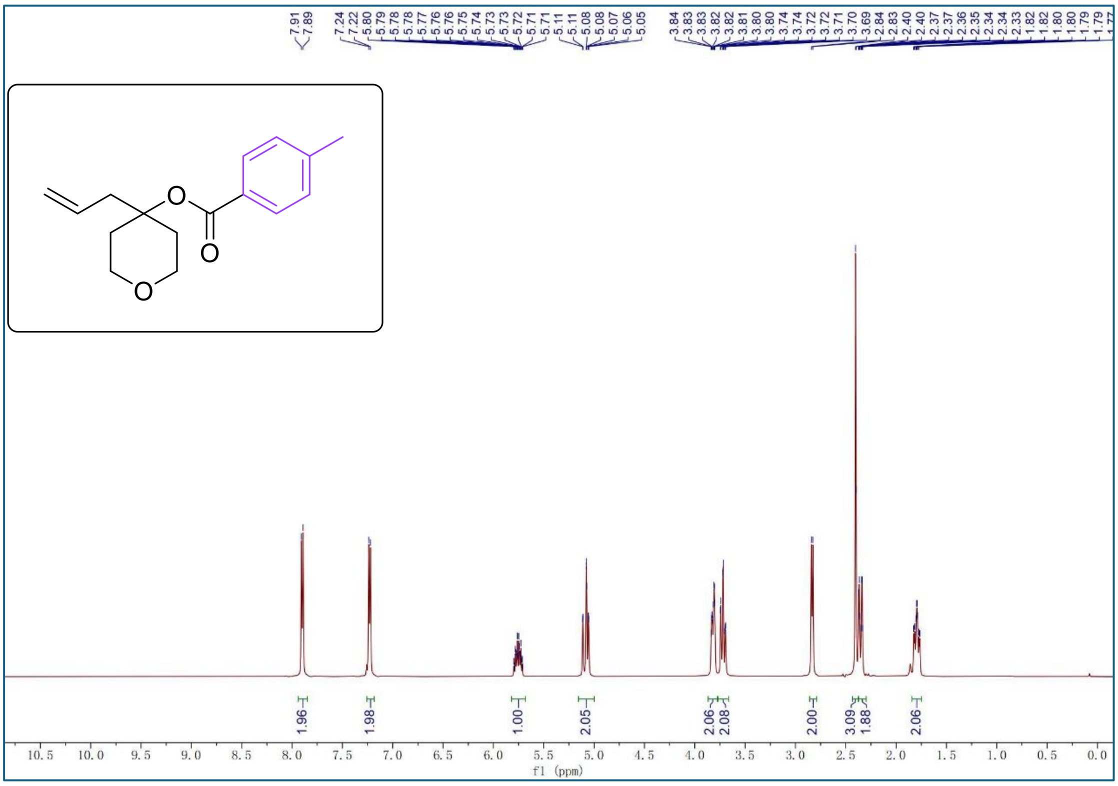

^{1}H NMR-spectrum (500 MHz, Chloroform-*d*) of **2z**

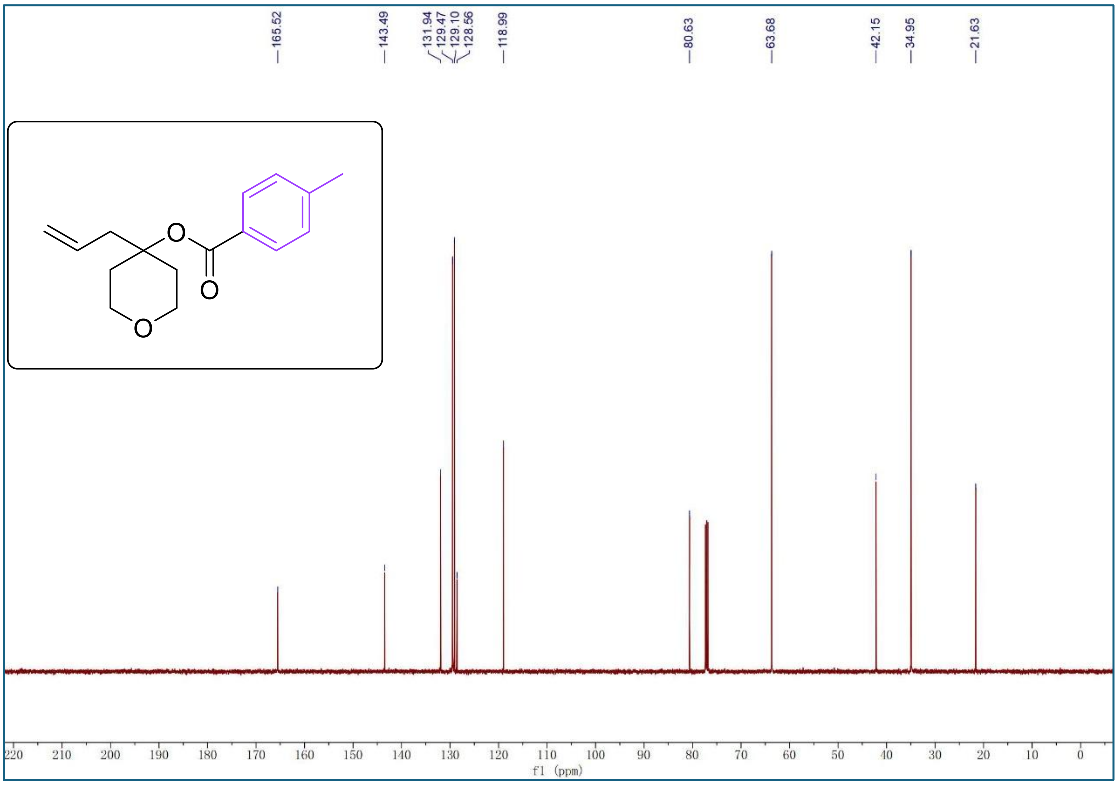

^{13}C NMR-spectrum (126 MHz, Chloroform-*d*) of **2z**

4-allyltetrahydro-2H-pyran-4-yl methyl terephthalate (2aa)

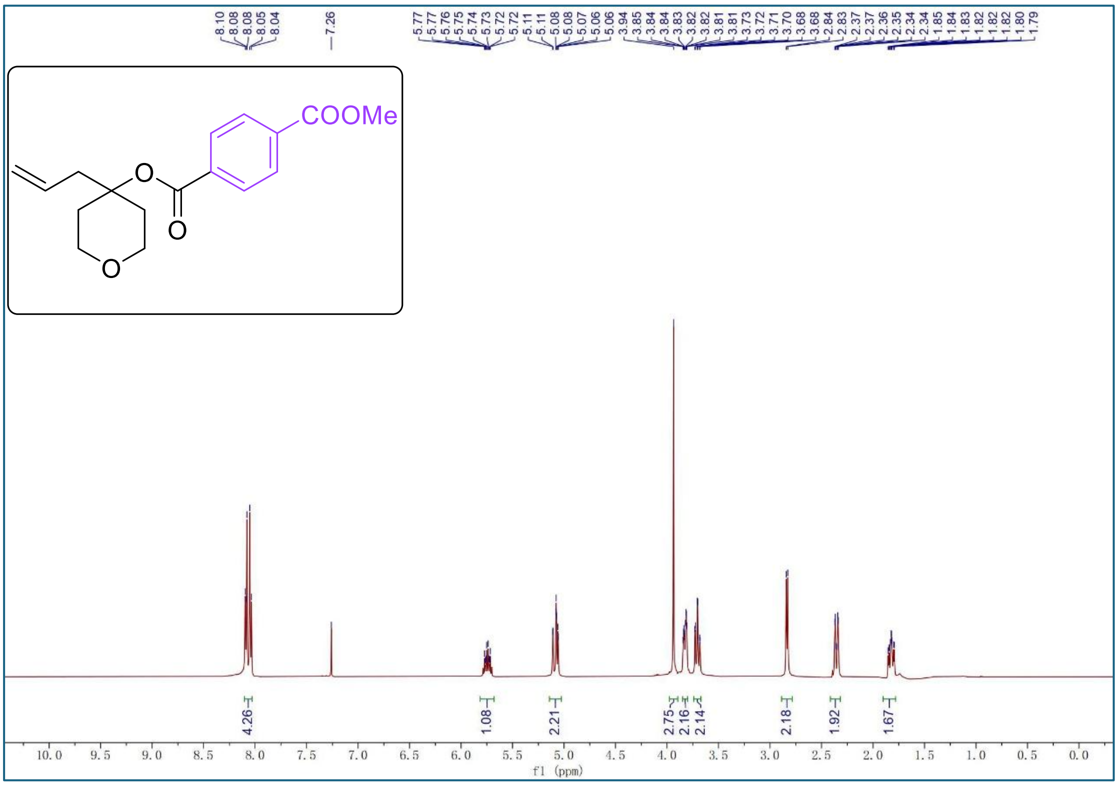

^{1}H NMR-spectrum (500 MHz, Chloroform-*d*) of **2aa**

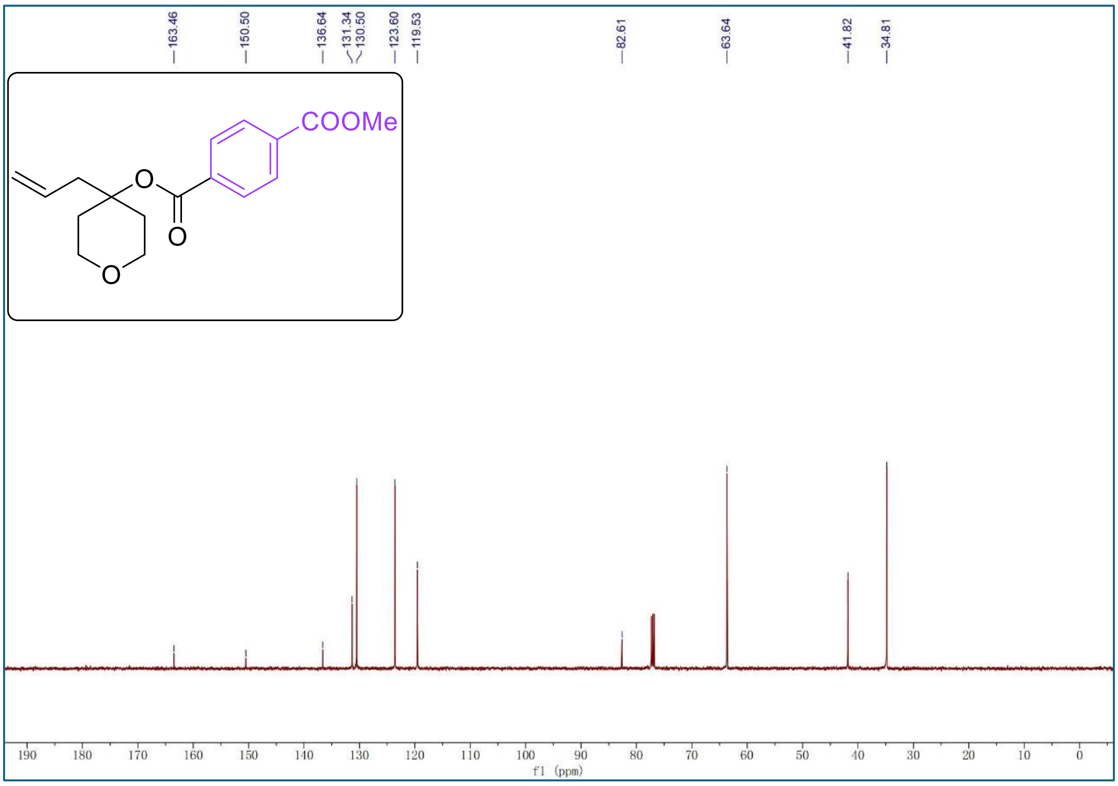

^{13}C NMR-spectrum (126 MHz, Chloroform-*d*) of **2aa**

1-allylcyclohexyl benzoate (2ab)

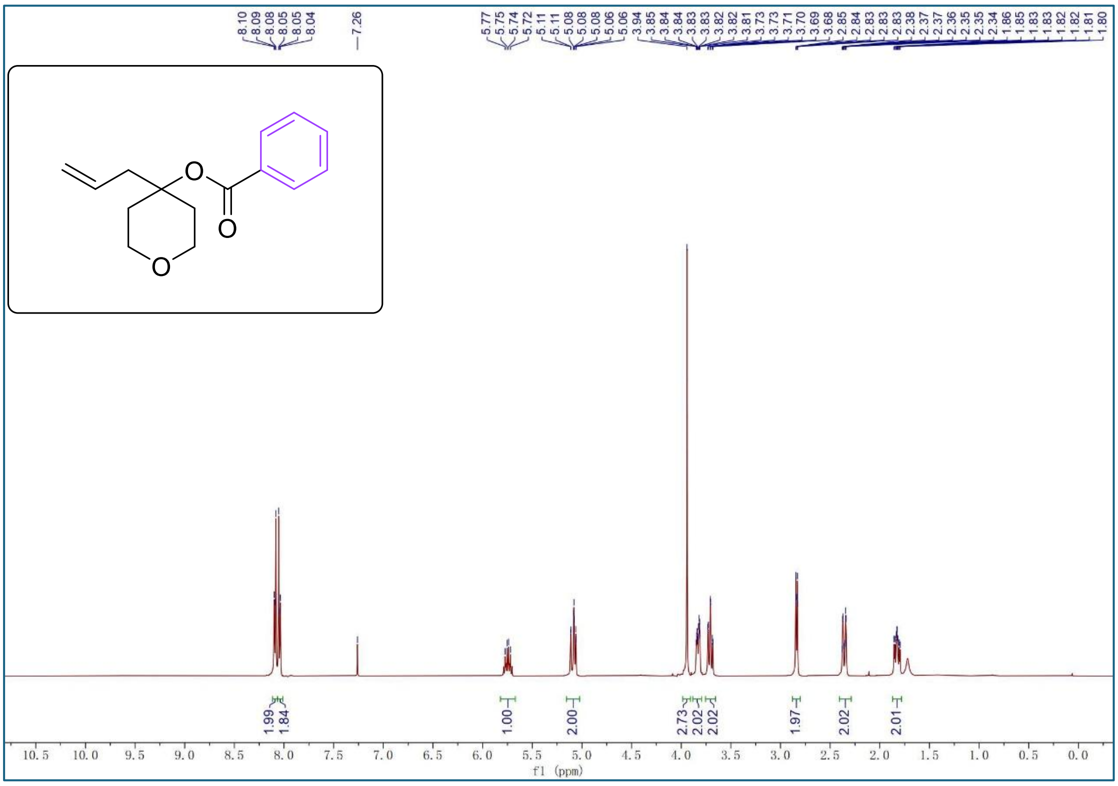

^{1}H NMR-spectrum (500 MHz, Chloroform-*d*) of **2ab**

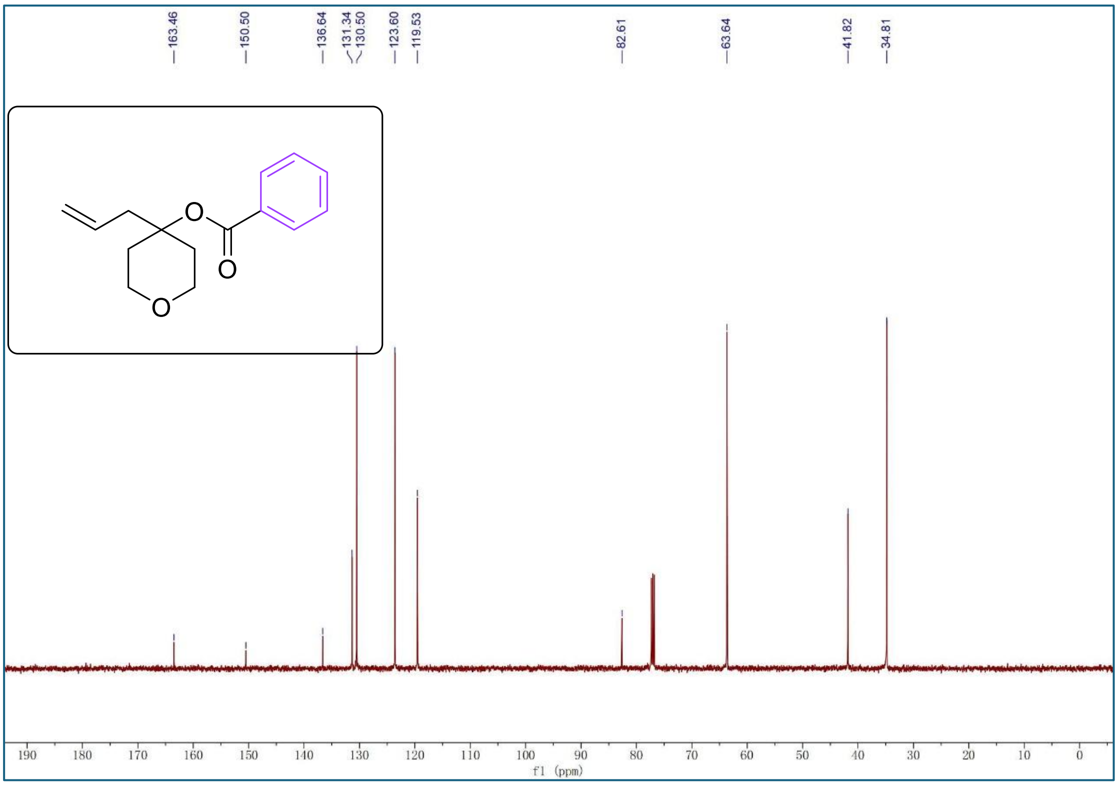

^{13}C NMR-spectrum (126 MHz, Chloroform-*d*) of **2ab**

2-methylbut-3-en-2-yl cyclopropanecarboxylate (2ac)

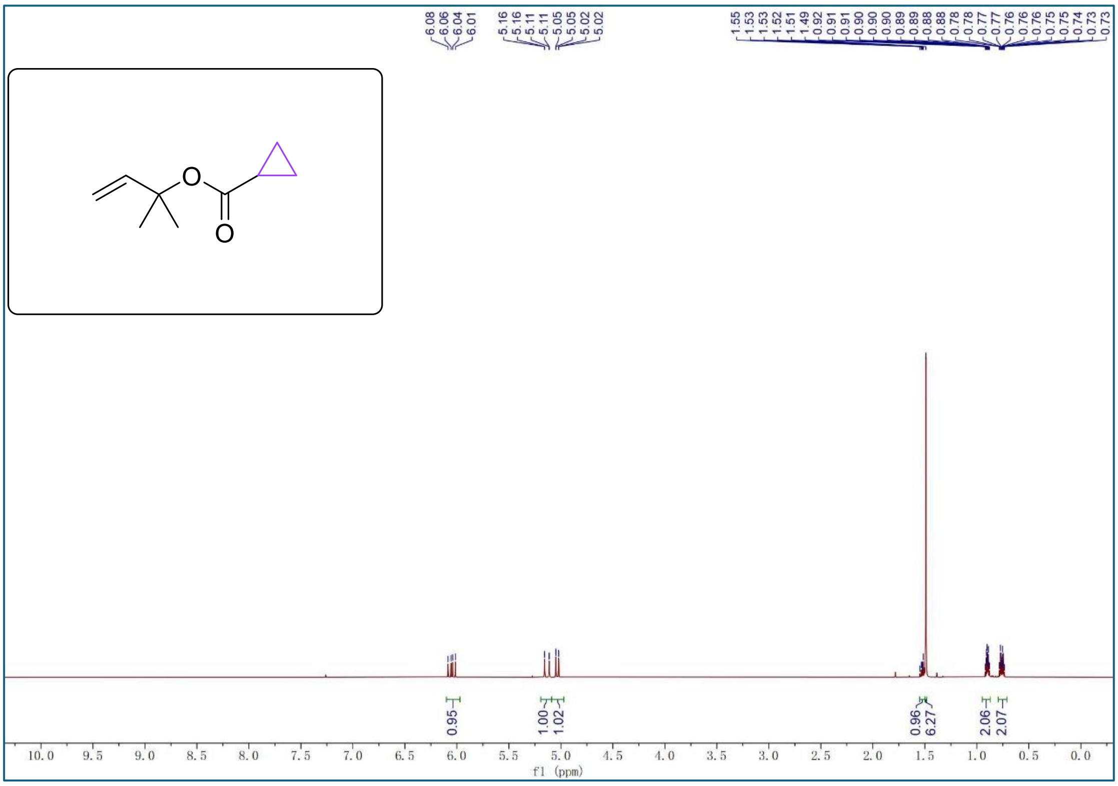

^{1}H NMR-spectrum (400 MHz, Chloroform-*d*) of **2ac**

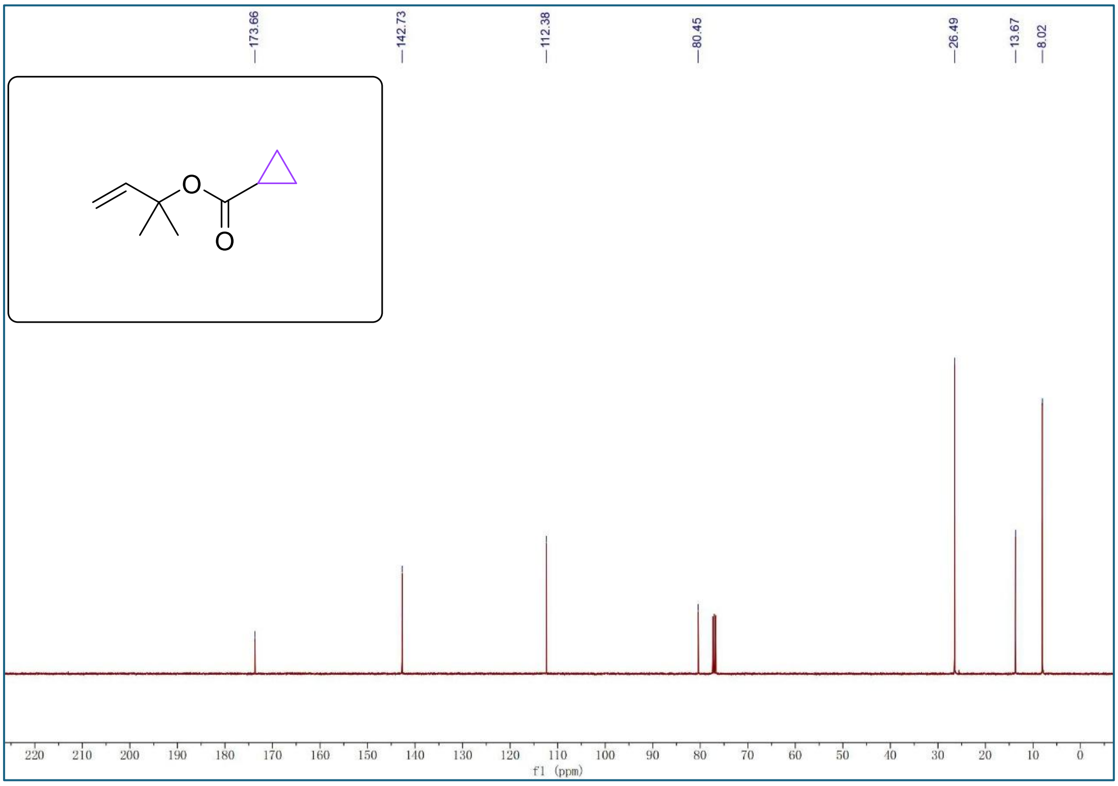

^{13}C NMR-spectrum (101 MHz, Chloroform-*d*) of **2ac**

ethyl 2-benzoyl-4-hydroxy-4-methyl-1,8-dioxaspiro[4.5]decane-2-carboxylate (3)

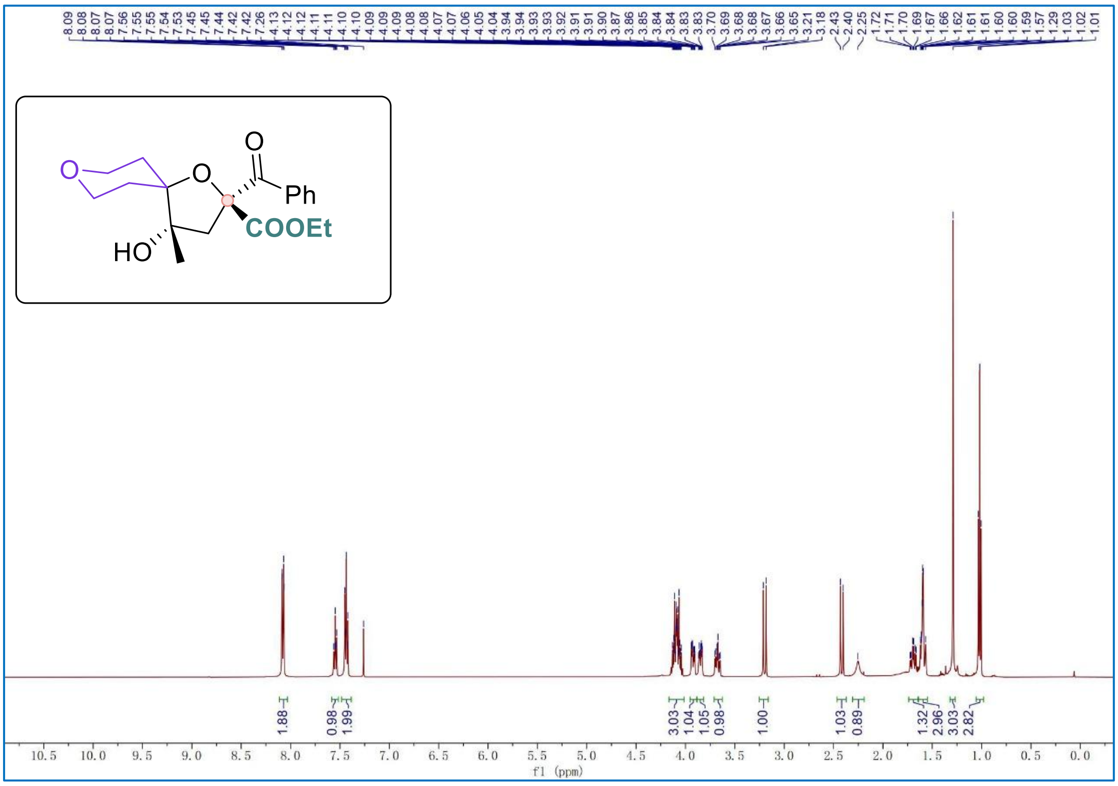

^{1}H NMR-spectrum (400 MHz, Chloroform-*d*) of **3**

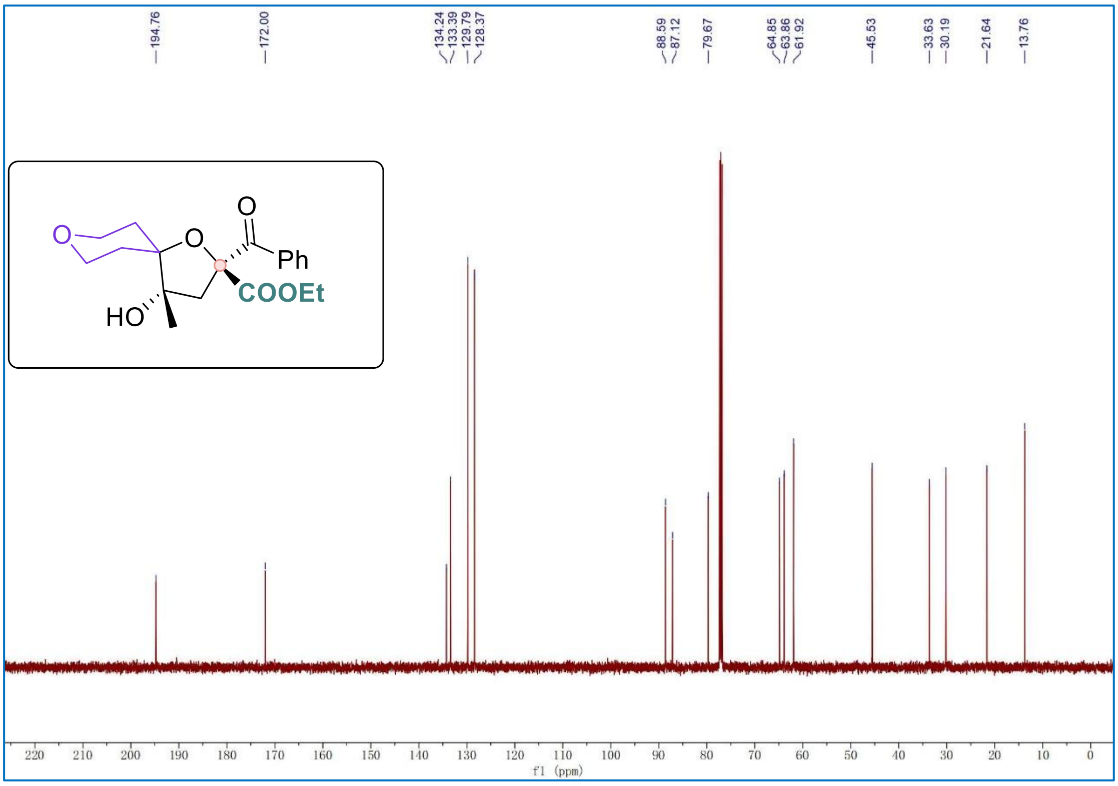

^{13}C NMR-spectrum (126 MHz, Chloroform-*d*) of **3**

ethyl 2-benzoyl-4-hydroxy-4-methyl-1-oxaspiro[4.5]decane-2-carboxylate (4)

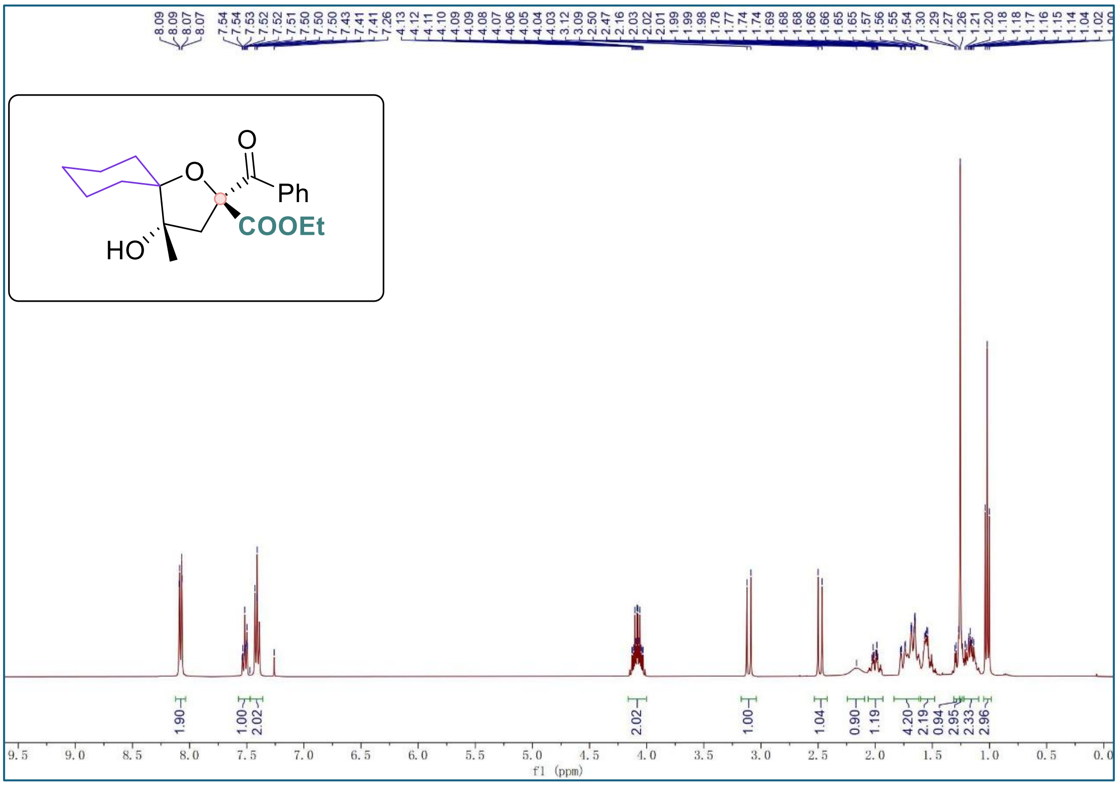

^{1}H NMR-spectrum (400 MHz, Chloroform-*d*) of **4**

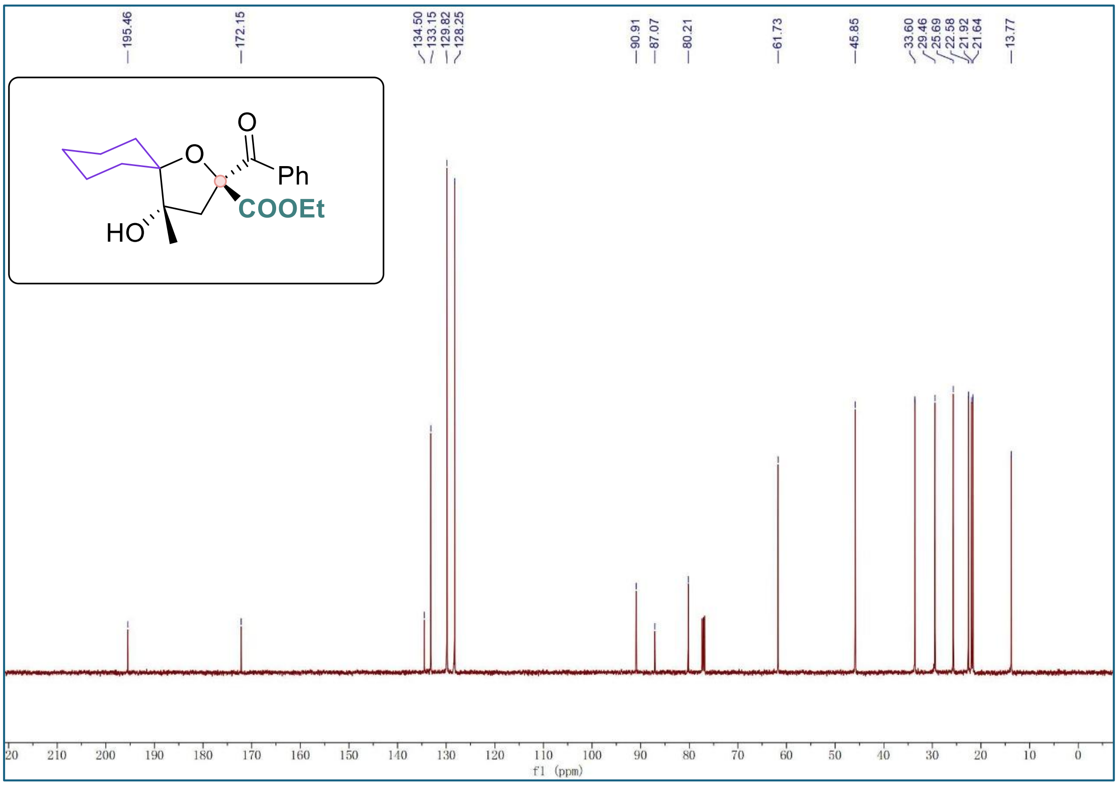

^{13}C NMR-spectrum (126 MHz, Chloroform-*d*) of **4**

SUPPORTING INFORMATION

ethyl 2-benzoyl-4-hydroxy-4-methyl-1-oxaspiro[4.6]undecane-2-carboxylate (5)

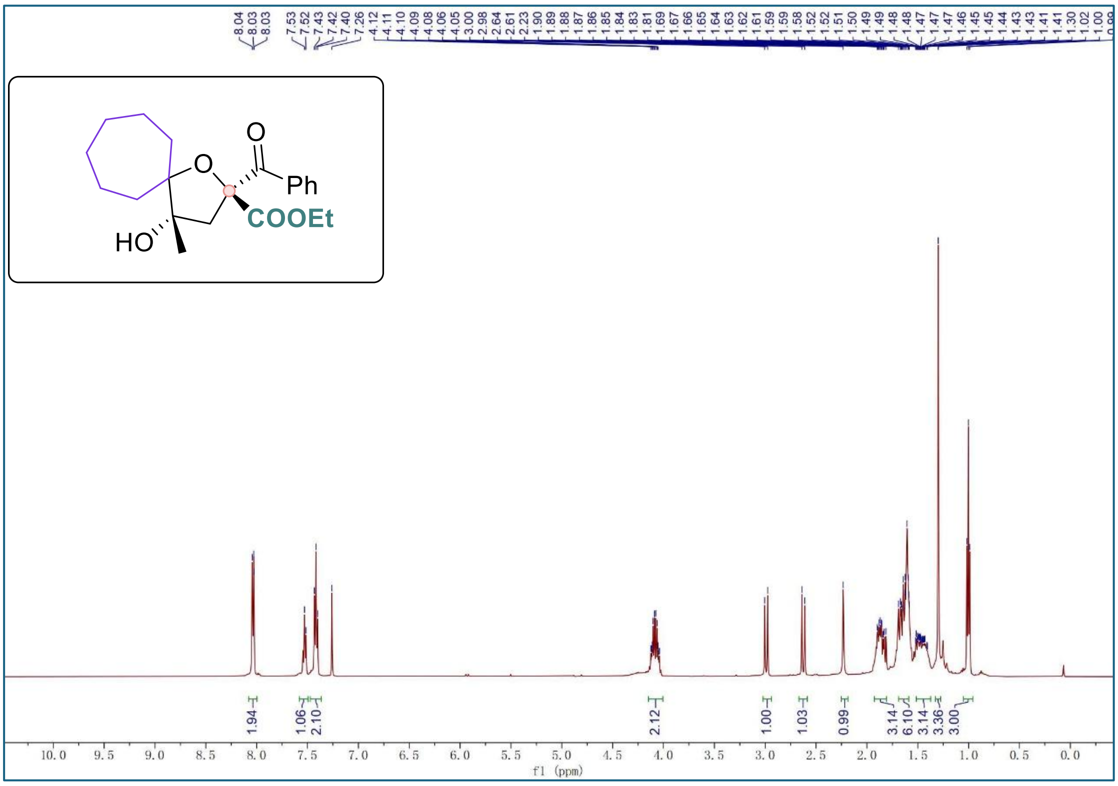

^{1}H NMR-spectrum (500 MHz, Chloroform-*d*) of **5**

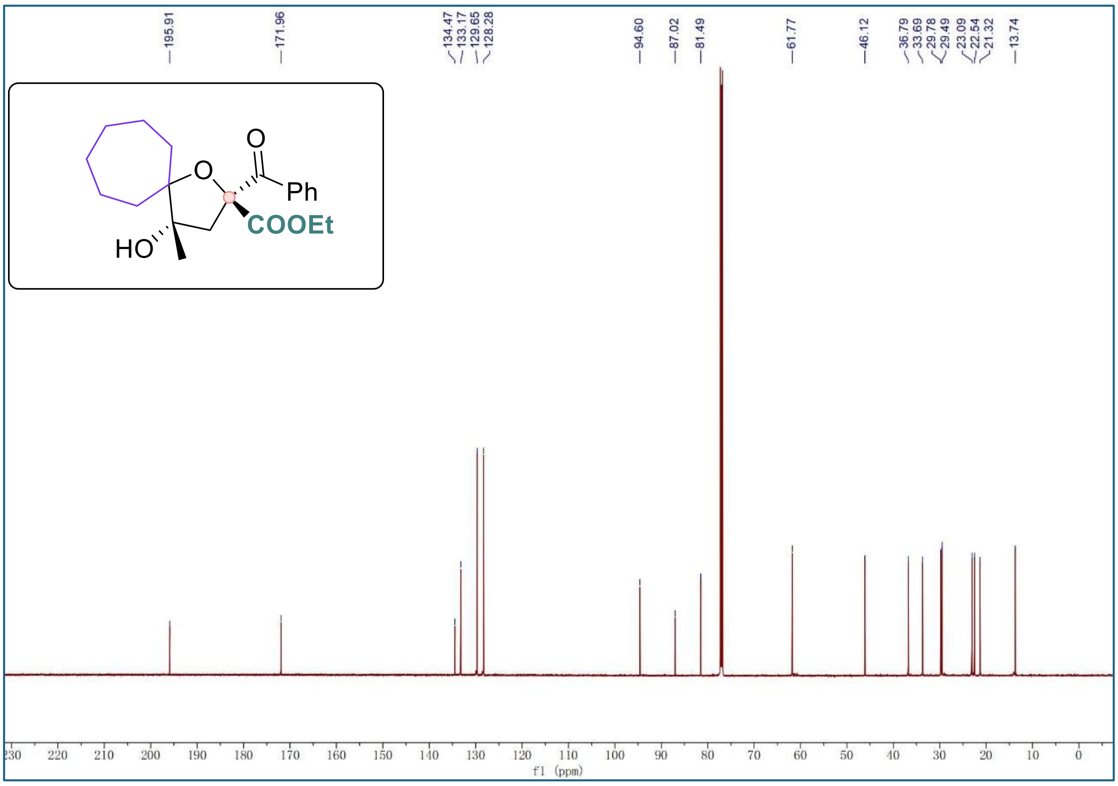

^{13}C NMR-spectrum (151 MHz, Chloroform-*d*) of **5**

ethyl 2-benzoyl-4-hydroxy-4-methyl-1-oxaspiro[4.14]nonadecane-2-carboxylate (6)

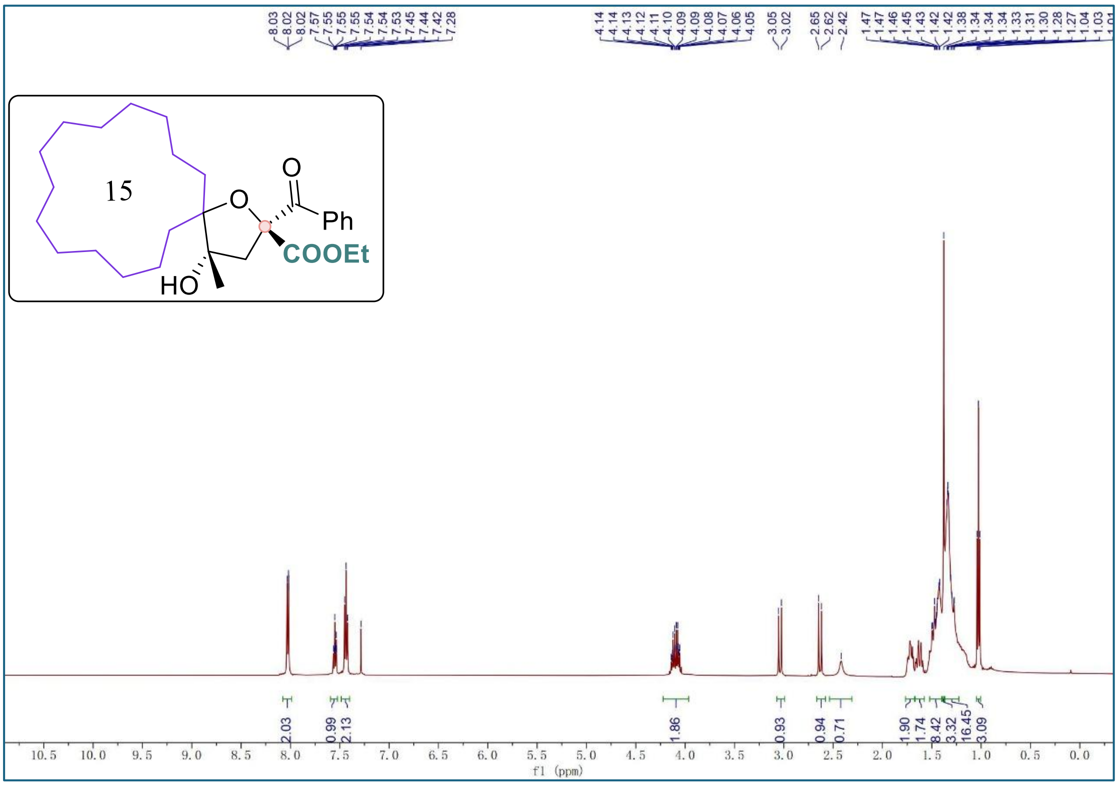

^{1}H NMR-spectrum (500 MHz, Chloroform-*d*) of **6**

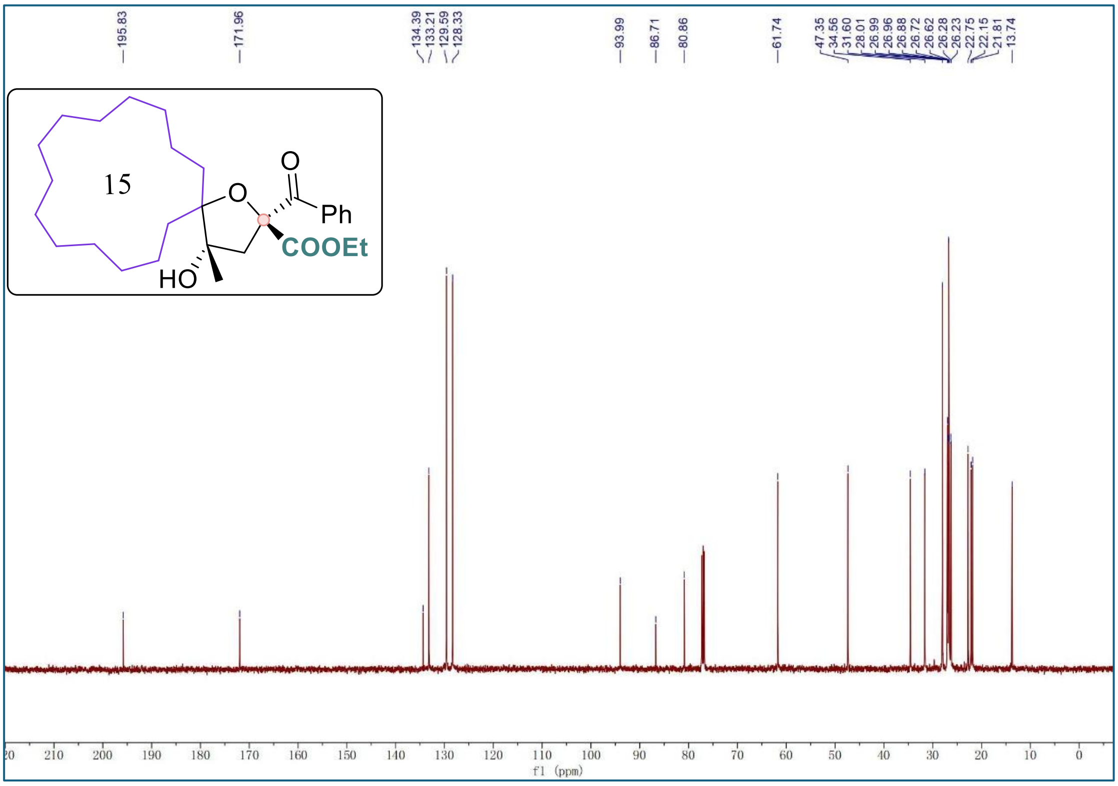

^{13}C NMR-spectrum (126 MHz, Chloroform-*d*) of **6**

ethyl 2-benzoyl-4-hydroxy-4,8,8-trimethyl-1-oxaspiro[4.5]decane-2-carboxylate (7)

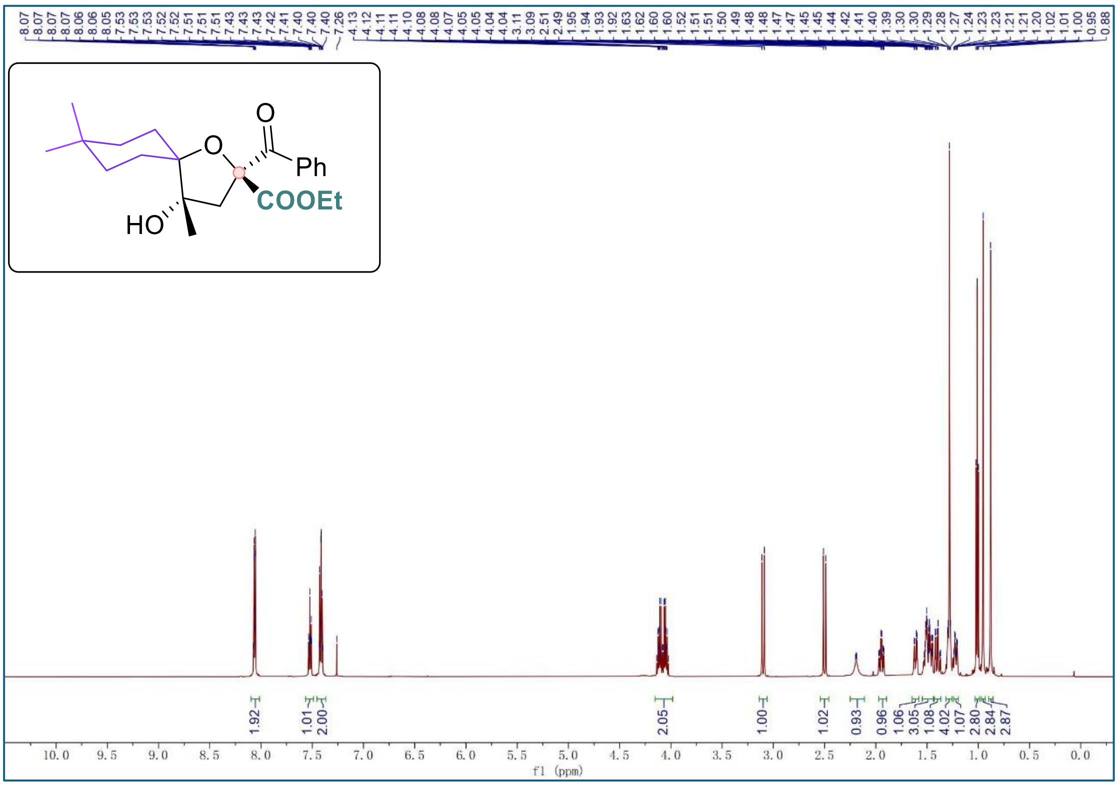

^{1}H NMR-spectrum (600 MHz, Chloroform-*d*) of **7**

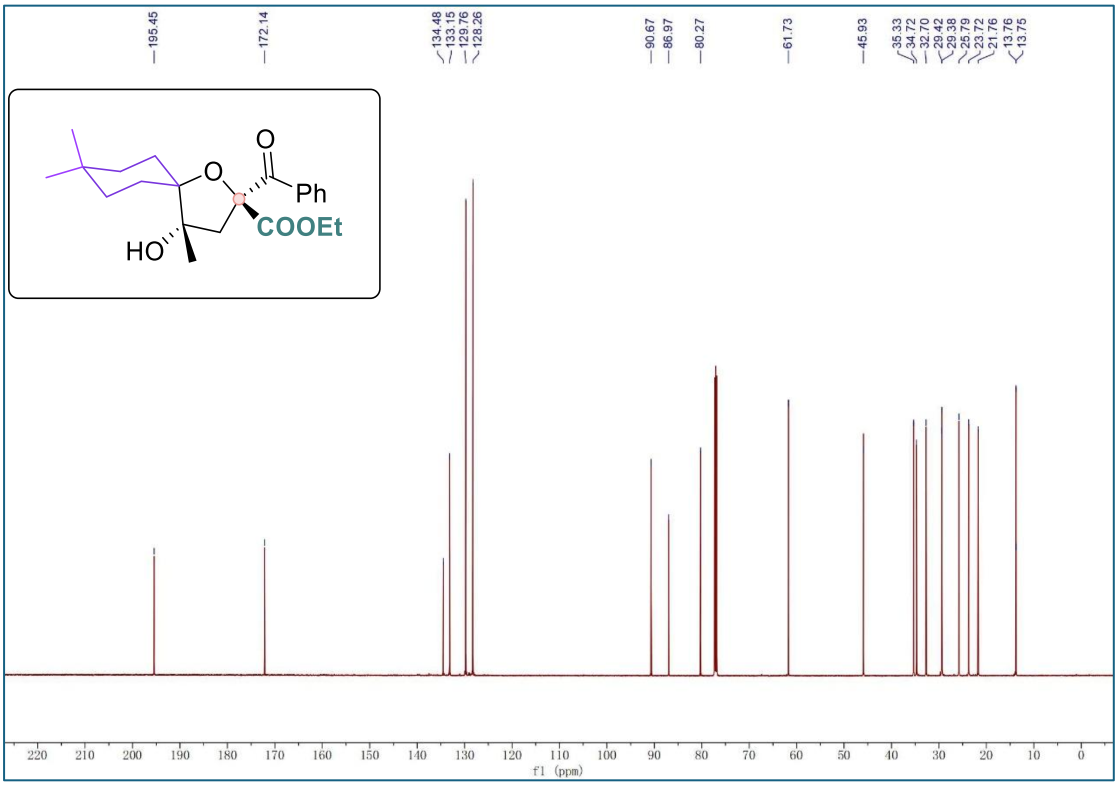

^{13}C NMR-spectrum (151 MHz, Chloroform-*d*) of **7**

ethyl 2-benzoyl-4-hydroxy-4-phenyl-1-oxaspiro[4.5]decane-2-carboxylate (8)

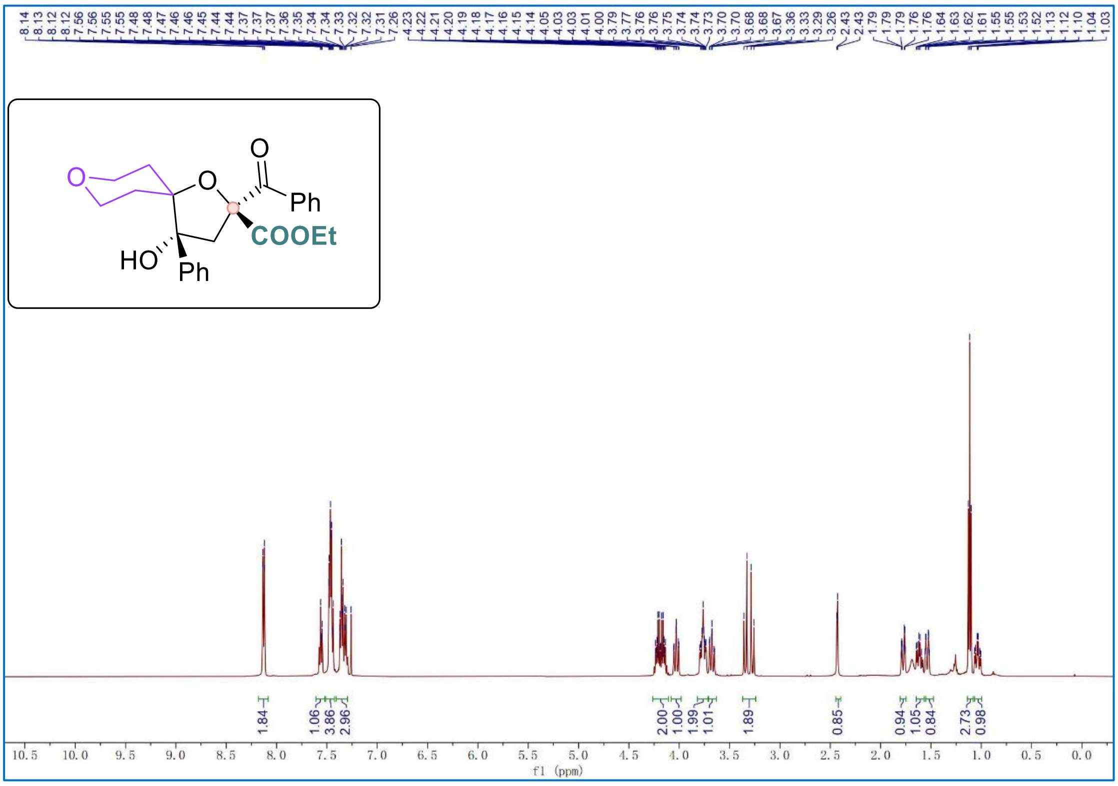

^{1}H NMR-spectrum (500 MHz, Chloroform-*d*) of **8**

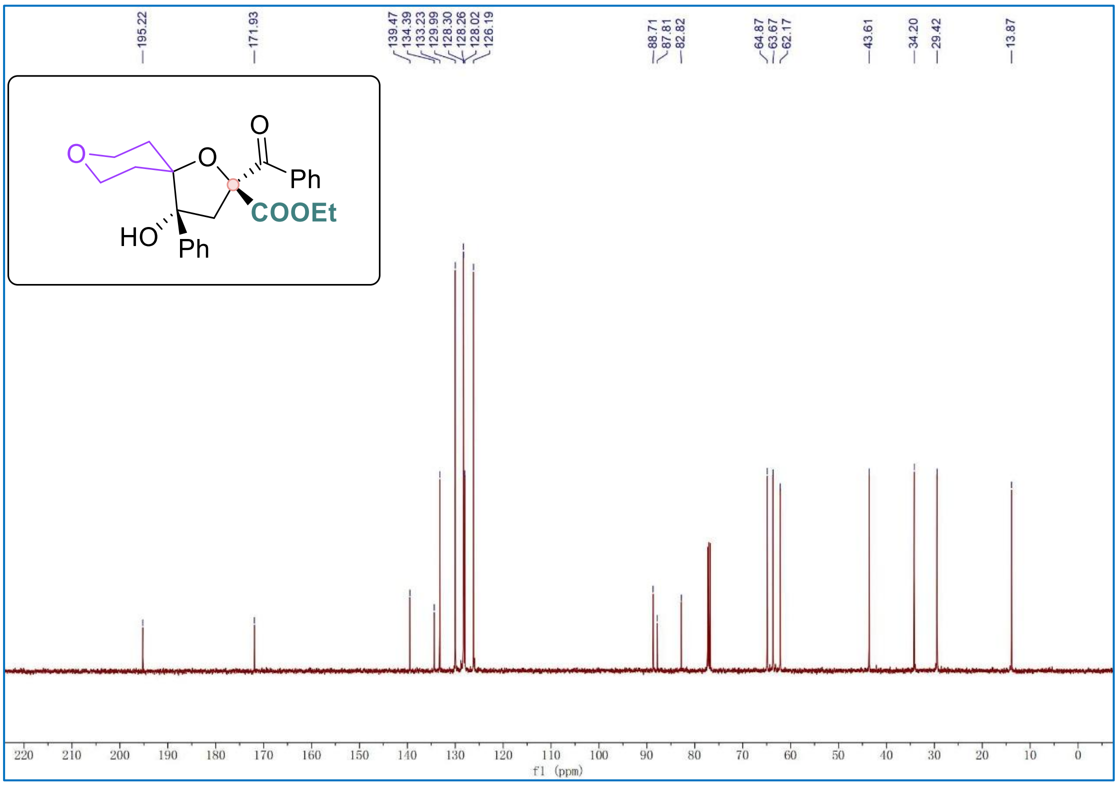

^{13}C NMR-spectrum (126 MHz, Chloroform-*d*) of **8**

ethyl 2-benzoyl-4-hydroxy-4-phenyl-1-oxaspiro[4.6]undecane-2-carboxylate (9)

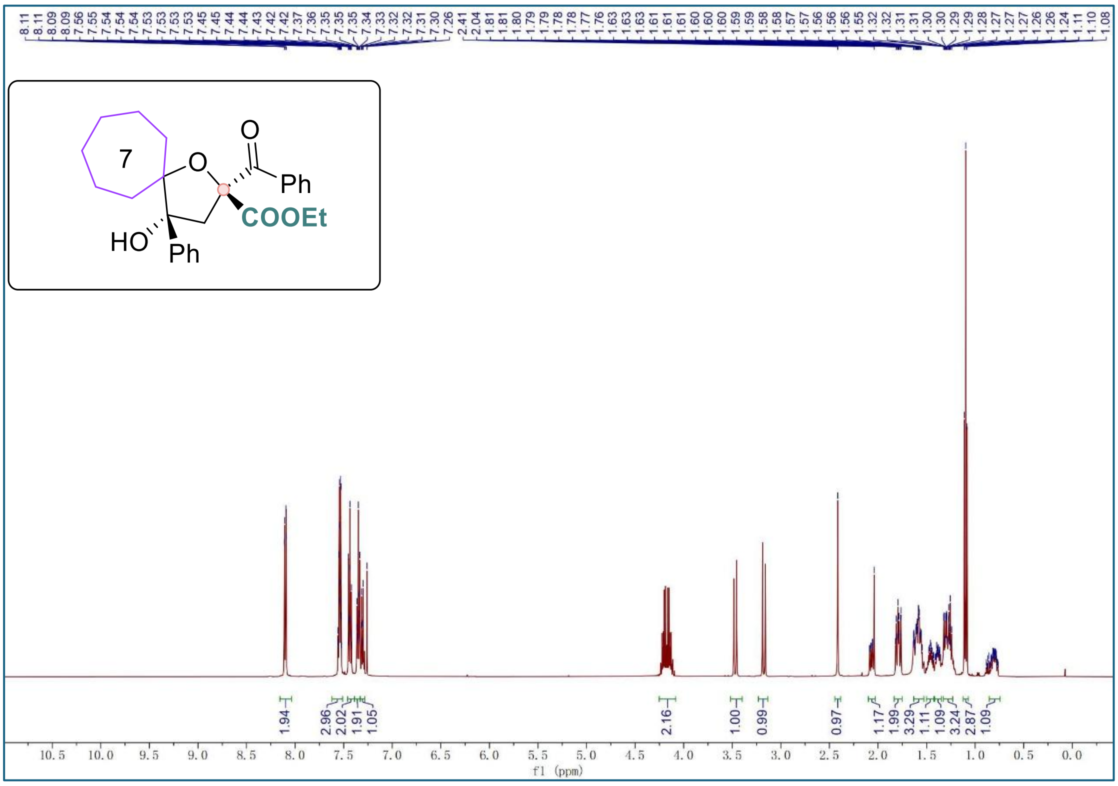

^{1}H NMR-spectrum (500 MHz, Chloroform-d) of **9**

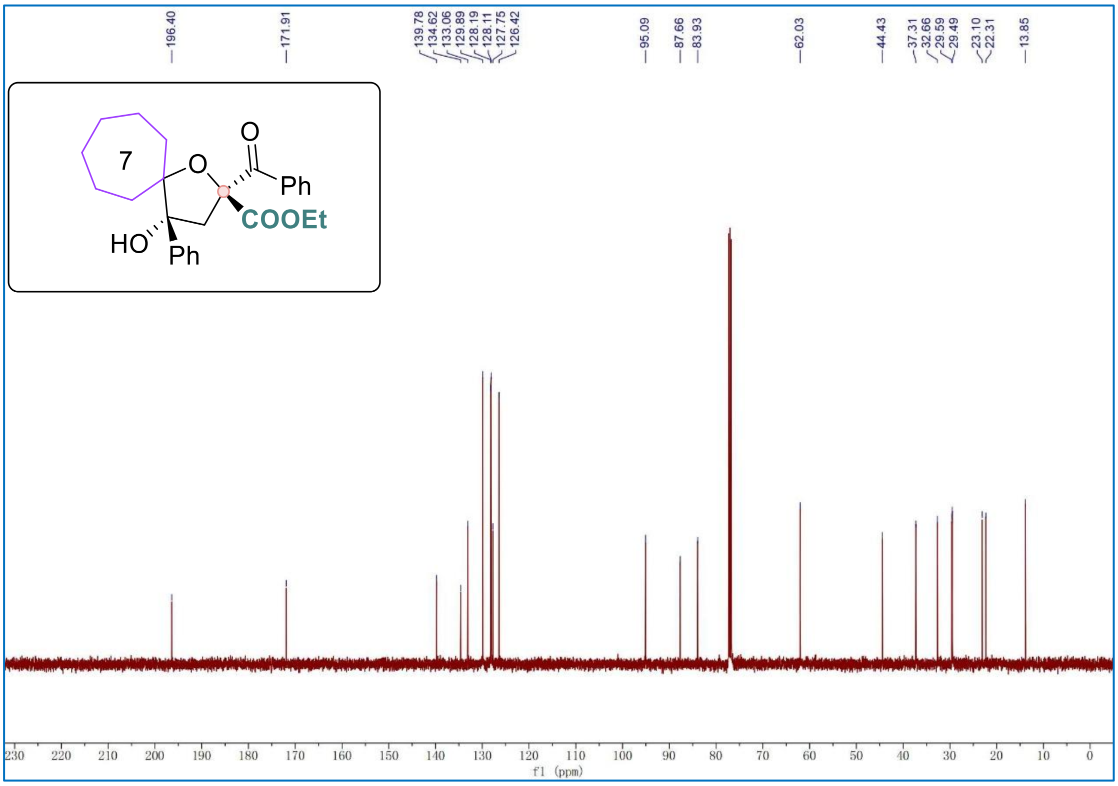

^{13}C NMR-spectrum (126 MHz, Chloroform-d) of **9**

ethyl 6-benzoyl-8-hydroxy-5-oxaspiro[3.4]octane-6-carboxylate (10)

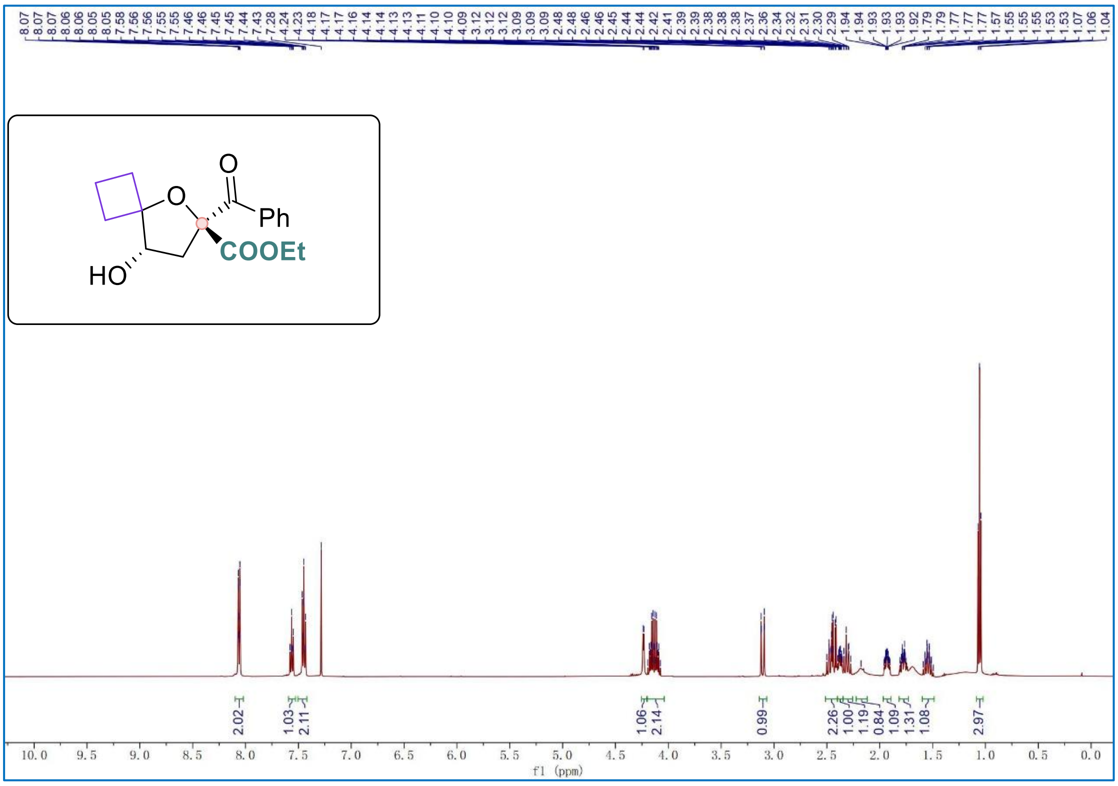

^{1}H NMR-spectrum (500 MHz, Chloroform-*d*) of **10**

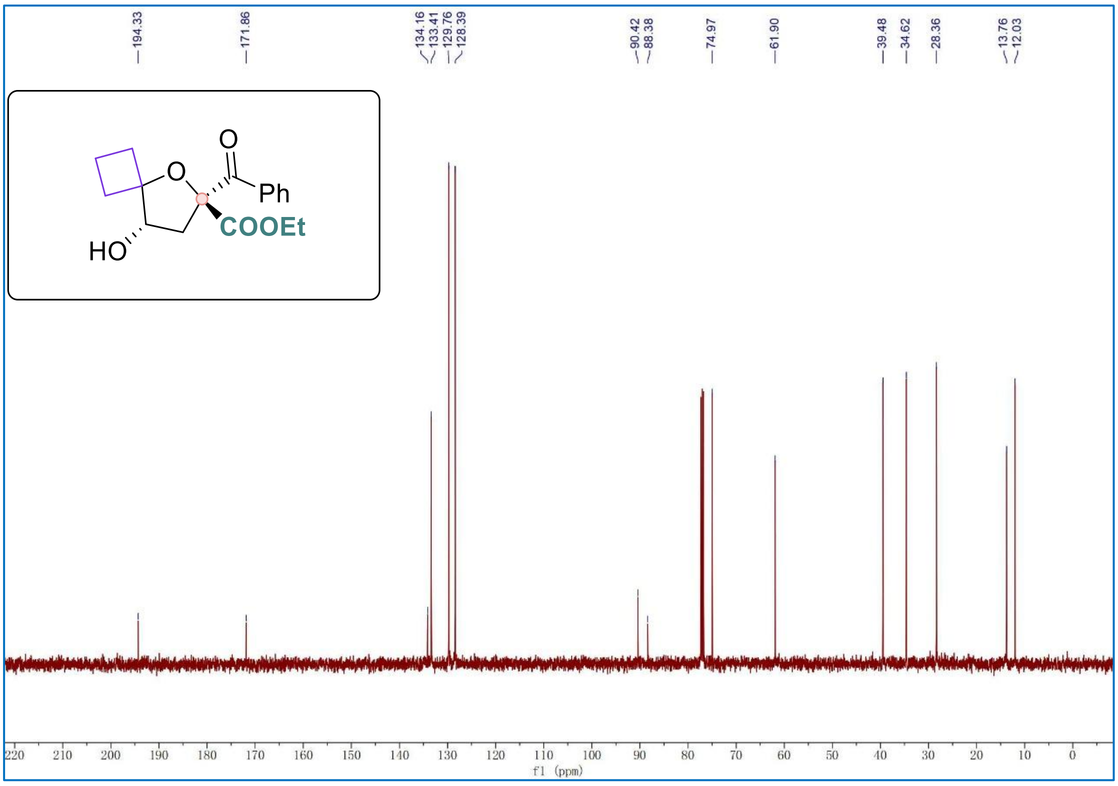

^{13}C NMR-spectrum (126 MHz, Chloroform-*d*) of **10**

ethyl 2-benzoyl-4-hydroxy-1-oxaspiro[4.4]nonane-2-carboxylate (11)

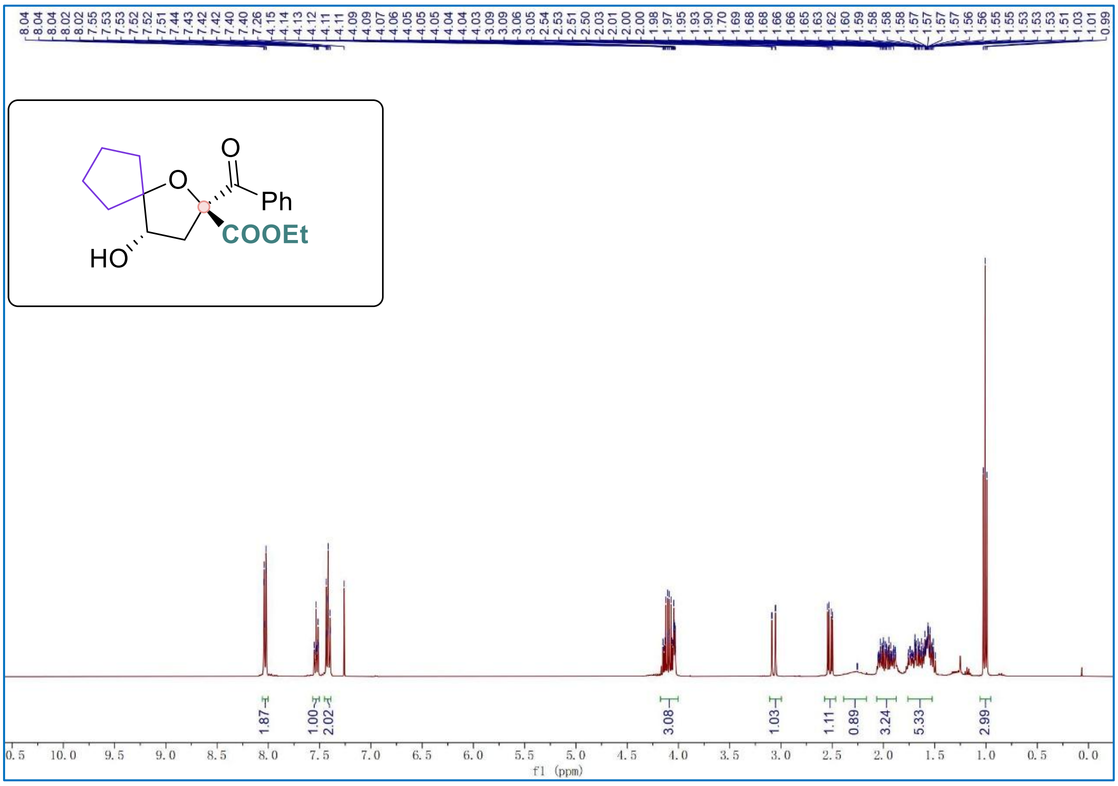

^{1}H NMR-spectrum (400 MHz, Chloroform-*d*) of **11**

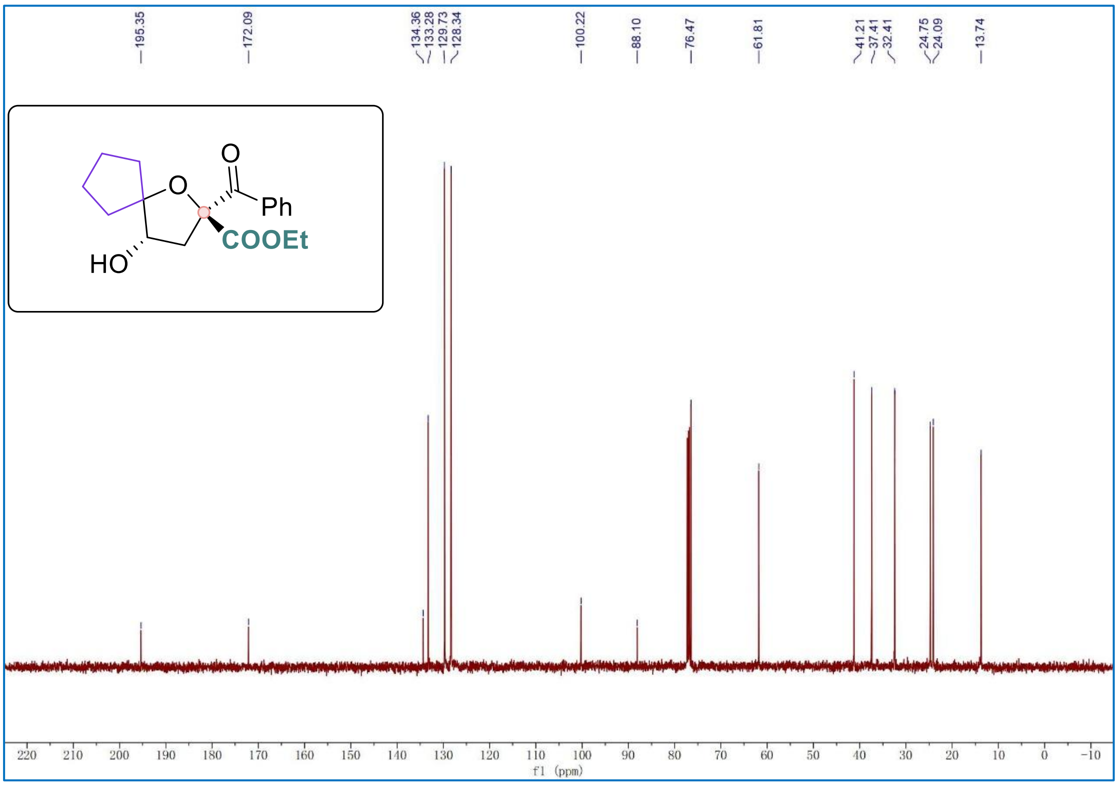

^{13}C NMR-spectrum (126 MHz, Chloroform-*d*) of **11**

ethyl 2-benzoyl-4-hydroxy-1-oxaspiro[4.5]decane-2-carboxylate (12)

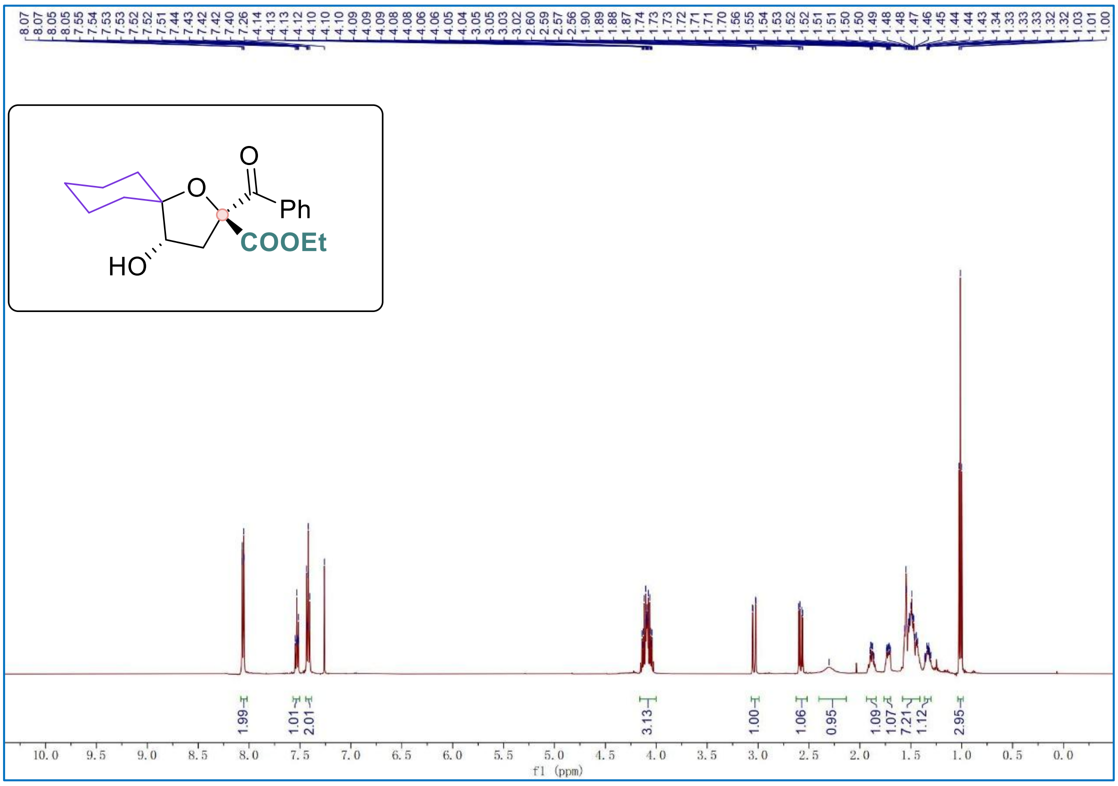

^{1}H NMR-spectrum (500 MHz, Chloroform-*d*) of **12**

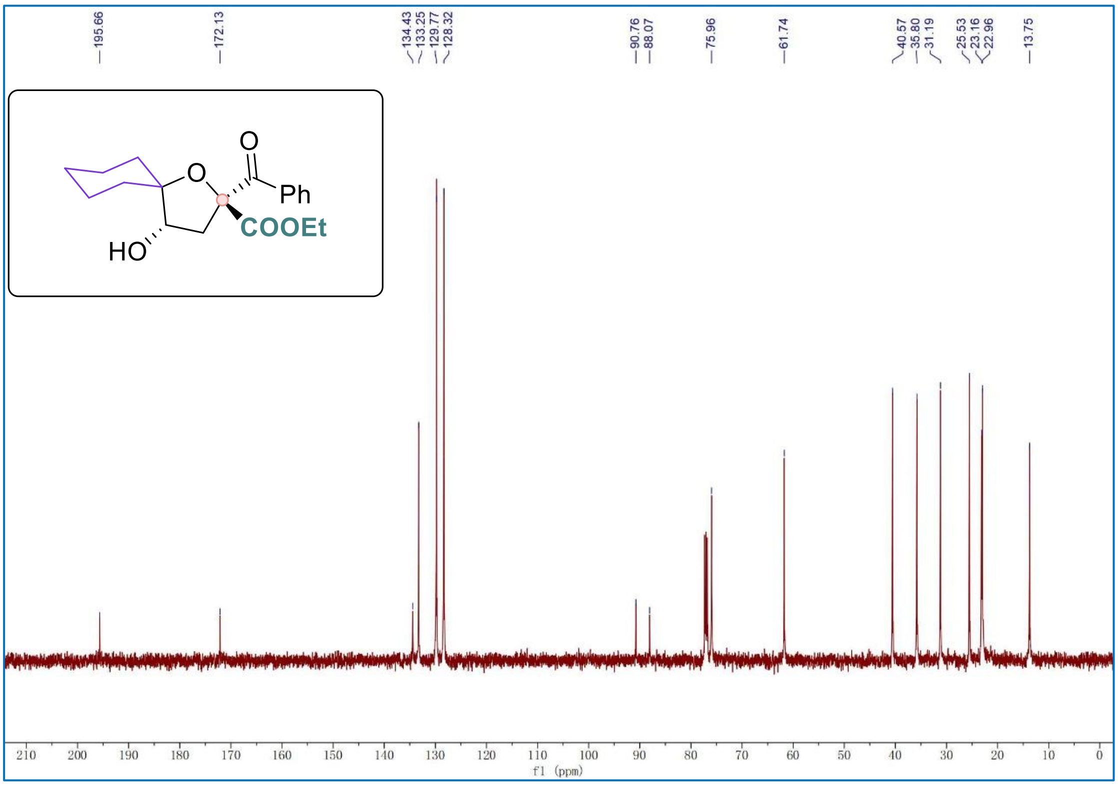

^{13}C NMR-spectrum (126 MHz, Chloroform-*d*) of **12**

SUPPORTING INFORMATION

ethyl 2-benzoyl-4-hydroxy-1-oxaspiro[4.7]dodecane-2-carboxylate (13)

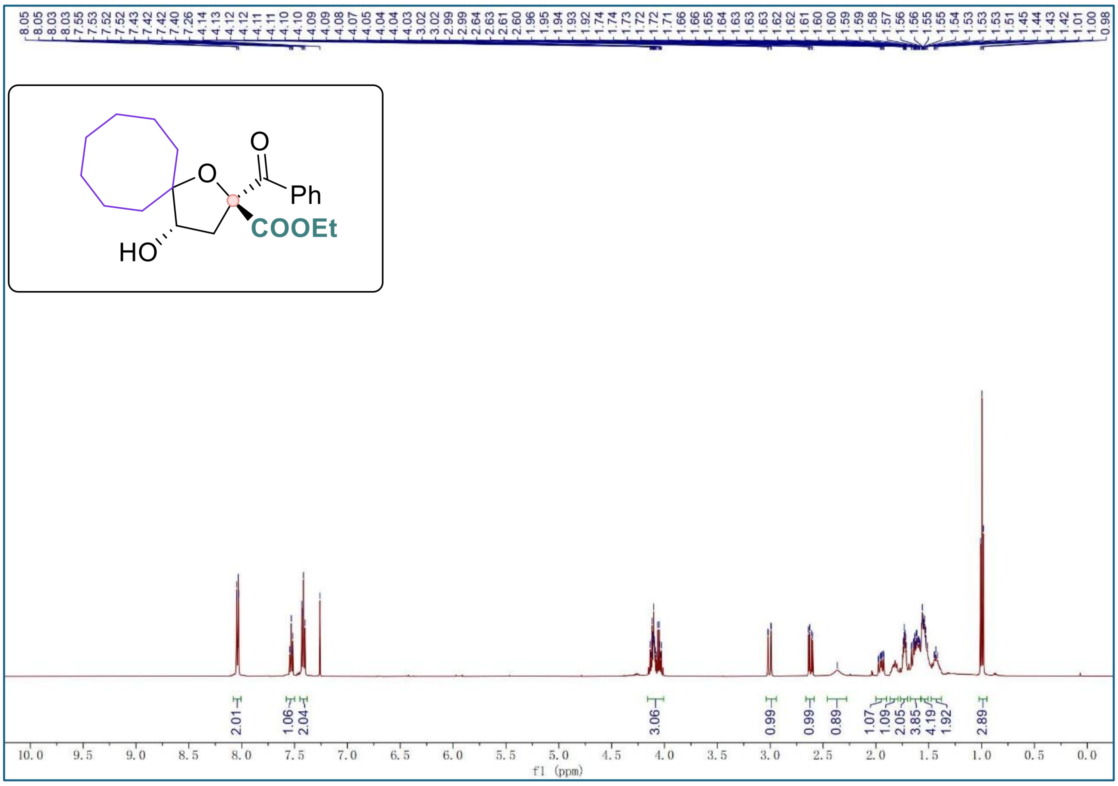

^{1}H NMR-spectrum (500 MHz, Chloroform-*d*) of **13**

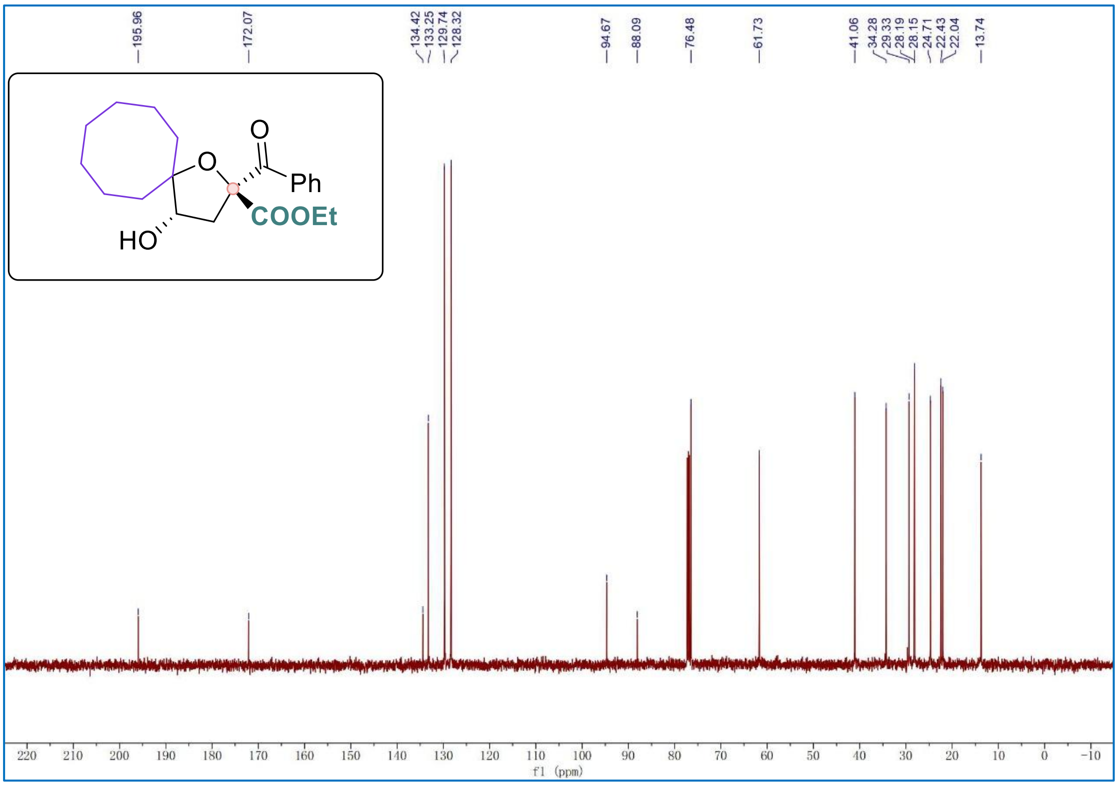

^{13}C NMR-spectrum (126 MHz, Chloroform-*d*) of **13**

ethyl 2-benzoyl-4-hydroxy-1-oxaspiro[4.11]hexadecane-2-carboxylate (14)

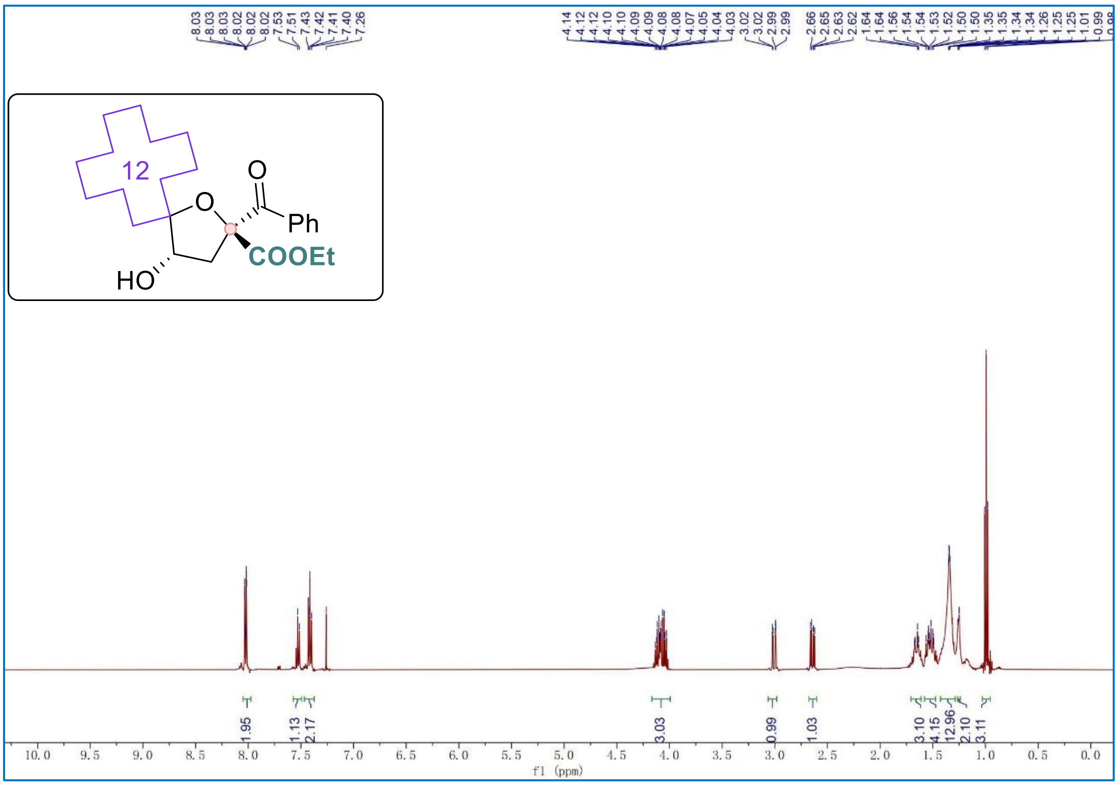

^{1}H NMR-spectrum (500 MHz, Chloroform-*d*) of **14**

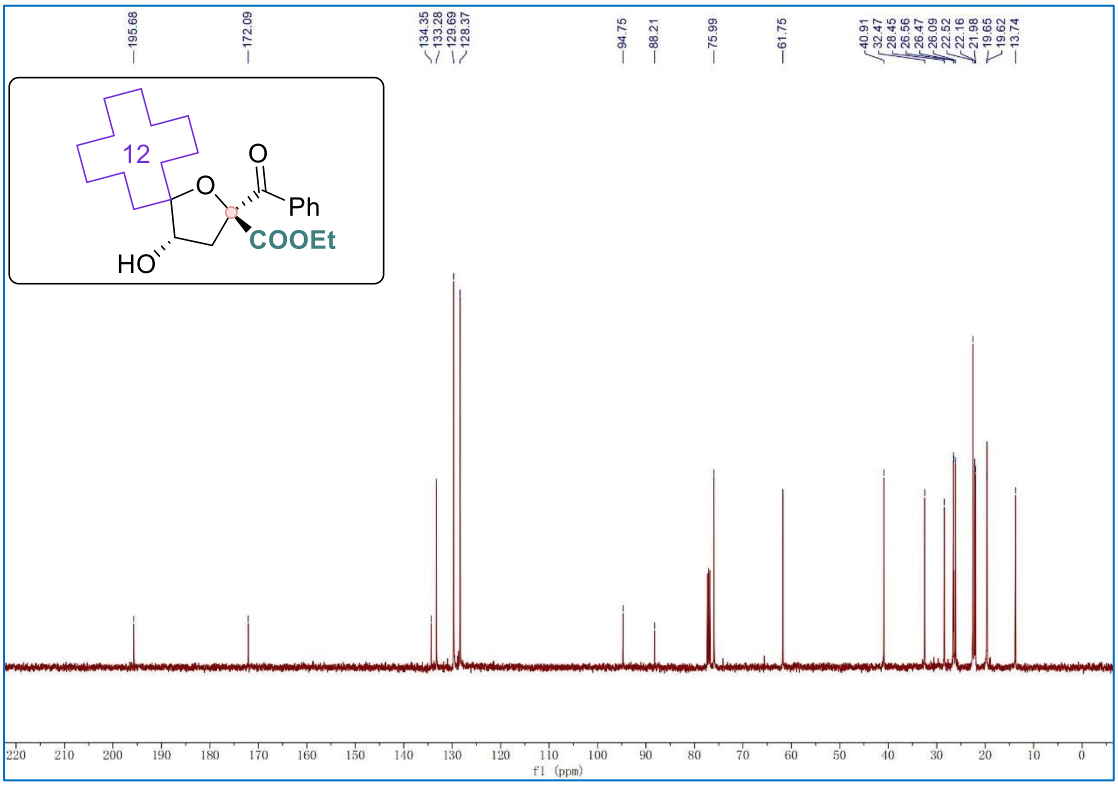

^{13}C NMR-spectrum (126 MHz, Chloroform-*d*) of **14**

ethyl 2-benzoyl-4-hydroxy-1-oxaspiro[4.14]nonadecane-2-carboxylate (15)

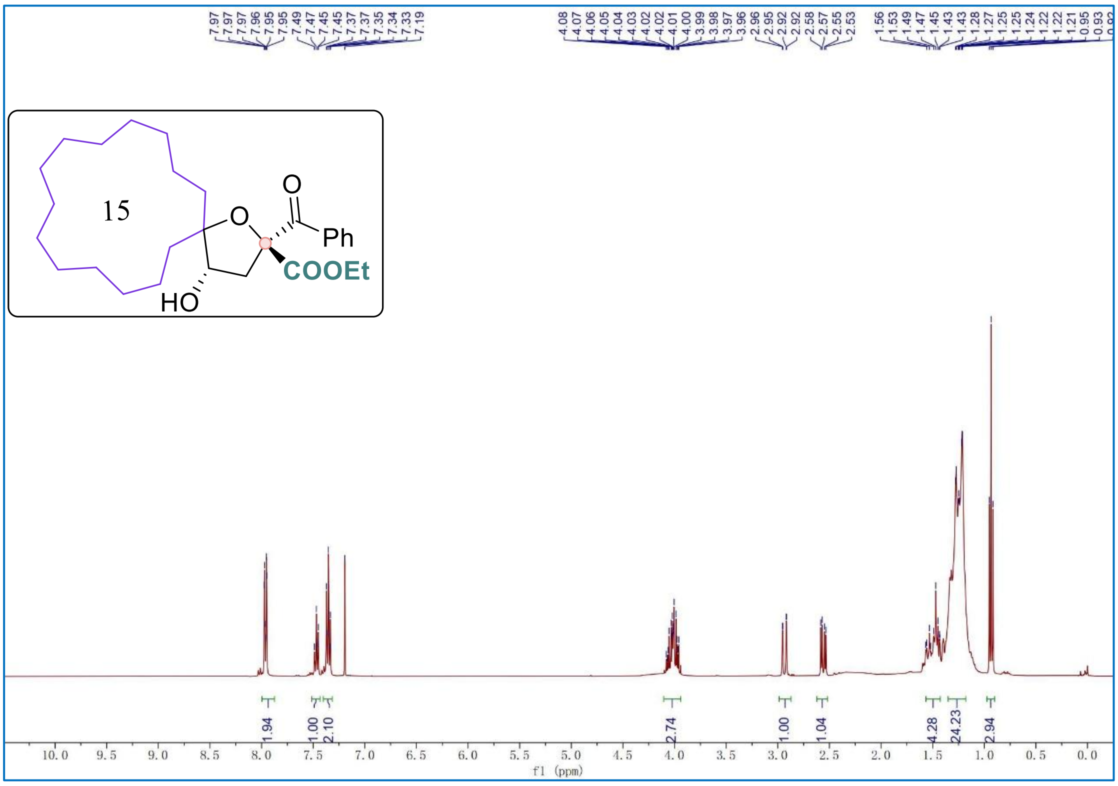

^{1}H NMR-spectrum (400 MHz, Chloroform-*d*) of **15**

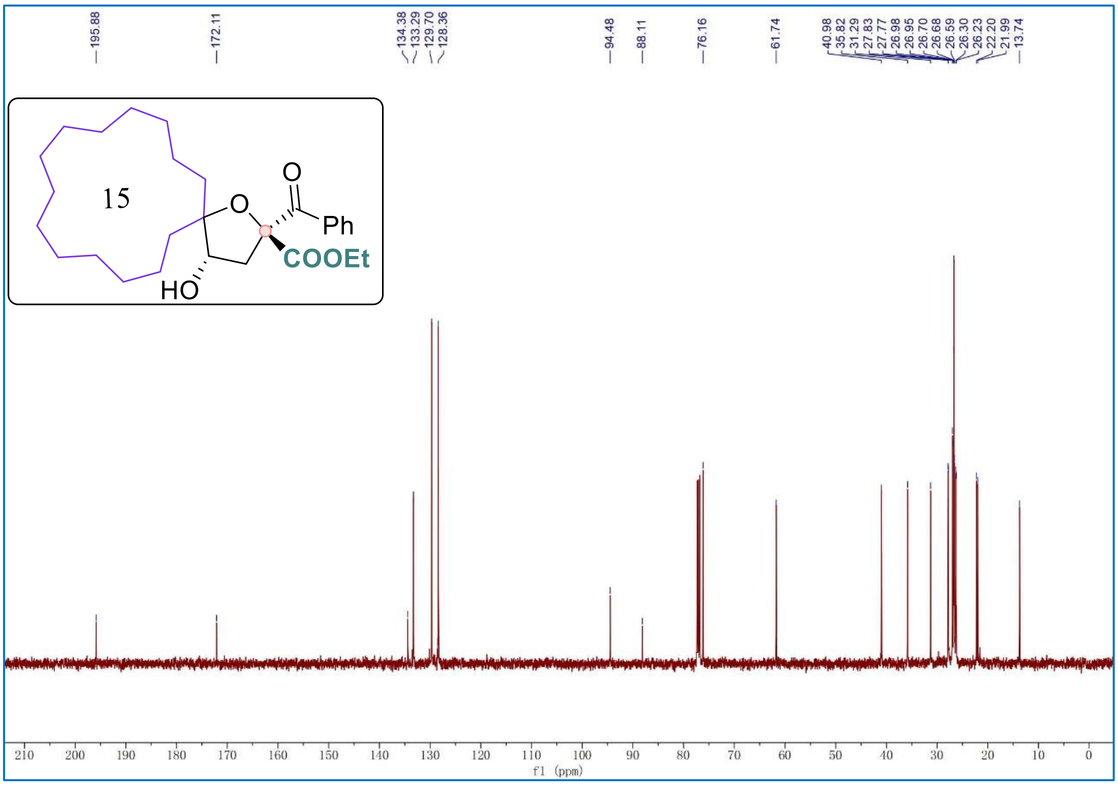

^{13}C NMR-spectrum (126 MHz, Chloroform-*d*) of **15**

ethyl 2-benzoyl-4-hydroxy-6,10-dimethyl-1-oxaspiro[4.5]decane-2-carboxylate (16)

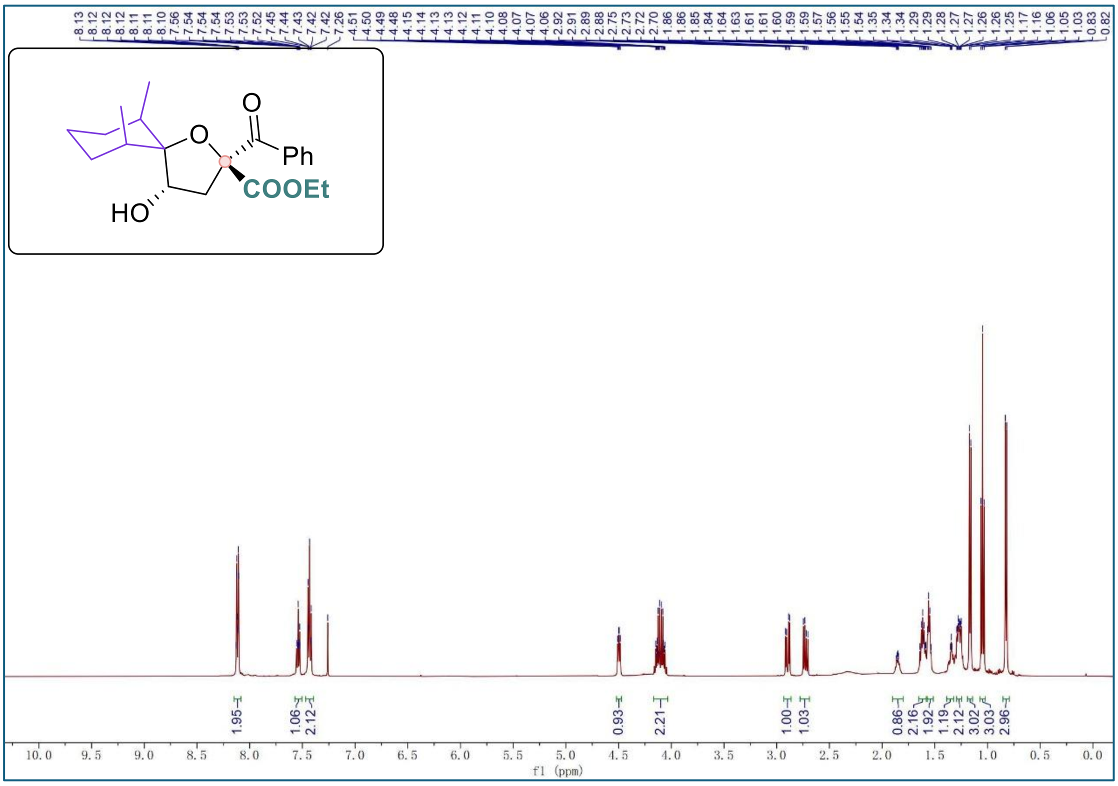

^{1}H NMR-spectrum (500 MHz, Chloroform-*d*) of **16**

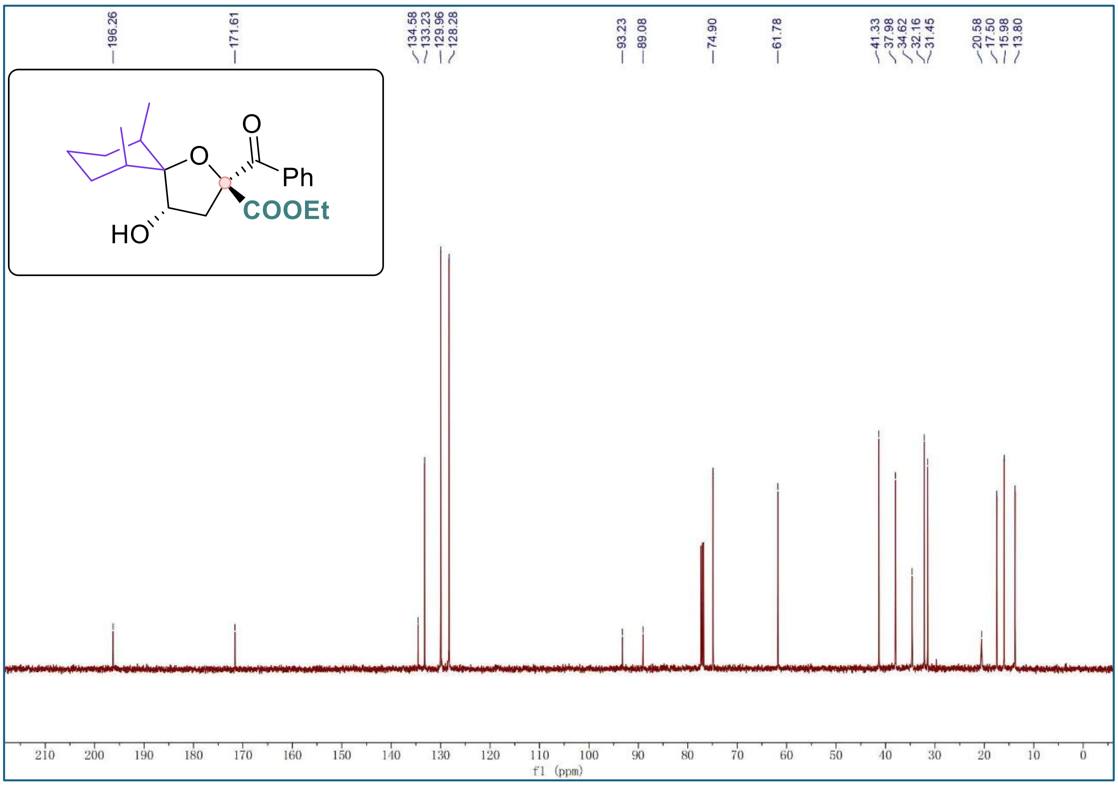

^{13}C NMR-spectrum (126 MHz, Chloroform-*d*) of **16**

ethyl 2-benzoyl-8,8-difluoro-4-hydroxy-1-oxaspiro[4.5]decane-2-carboxylate (17)

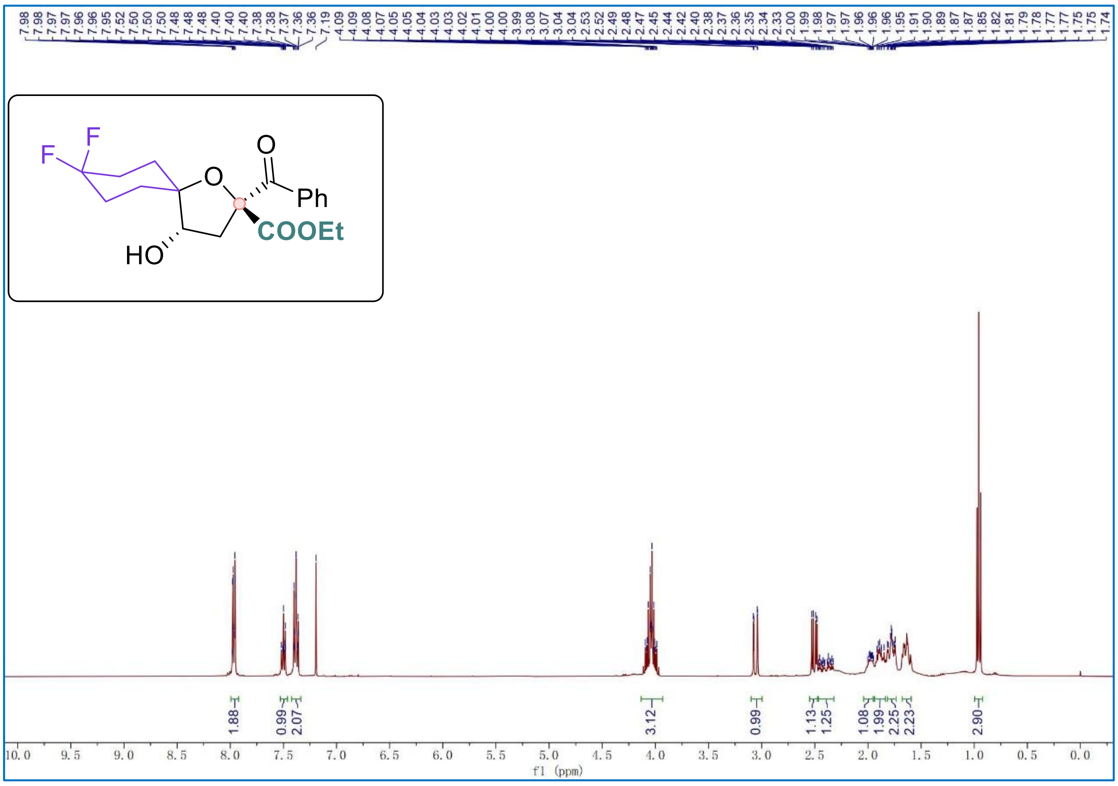

^{1}H NMR-spectrum (400 MHz, Chloroform-*d*) of **17**

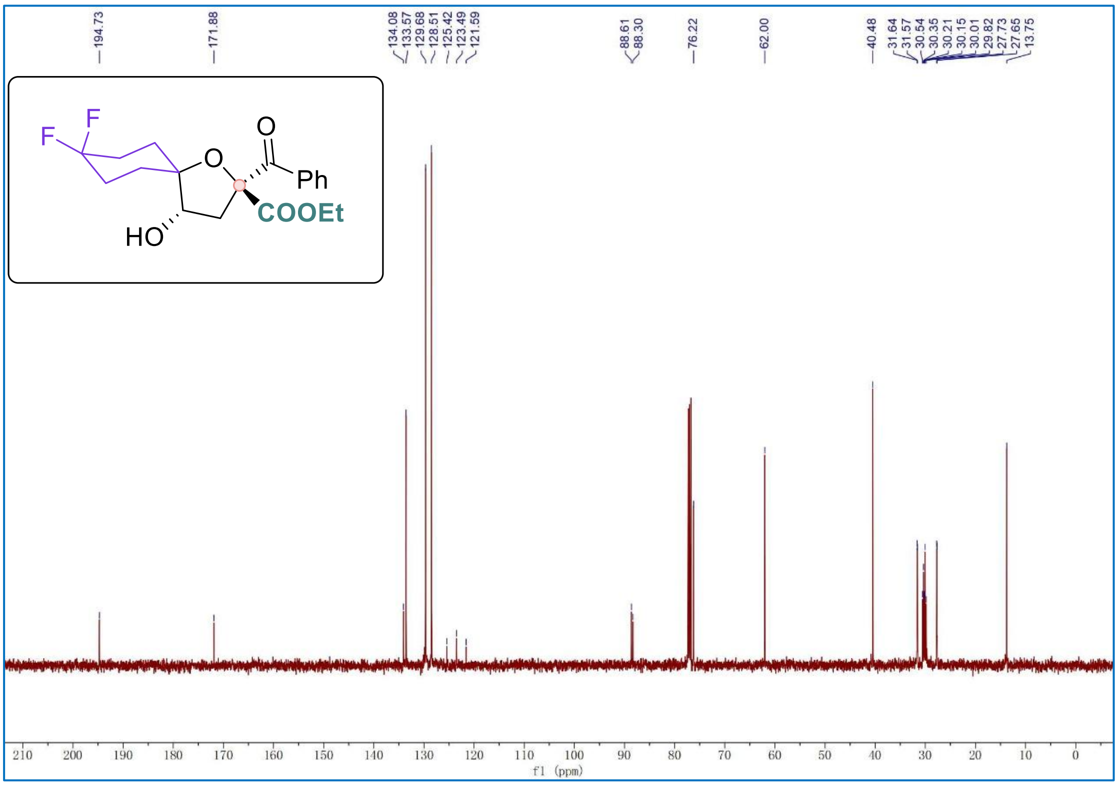

^{13}C NMR-spectrum (126 MHz, Chloroform-*d*) of **17**

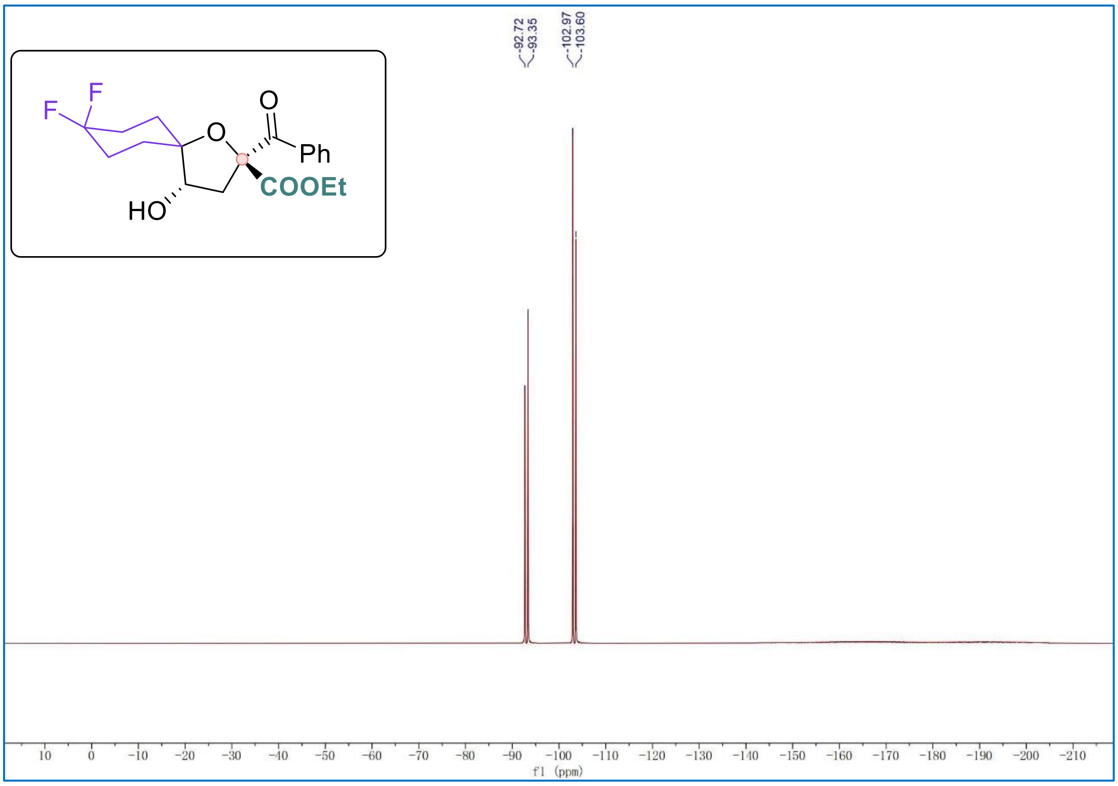

^{19}F NMR-spectrum (377 MHz, Chloroform-*d*) of **17**

SUPPORTING INFORMATION

ethyl 5'-benzoyl-3'-hydroxy-4',5,5',6,8,9-hexahydro-3'H-spiro[benzo[7]annulene-7,2'-furan]-5'-carboxylate (18)

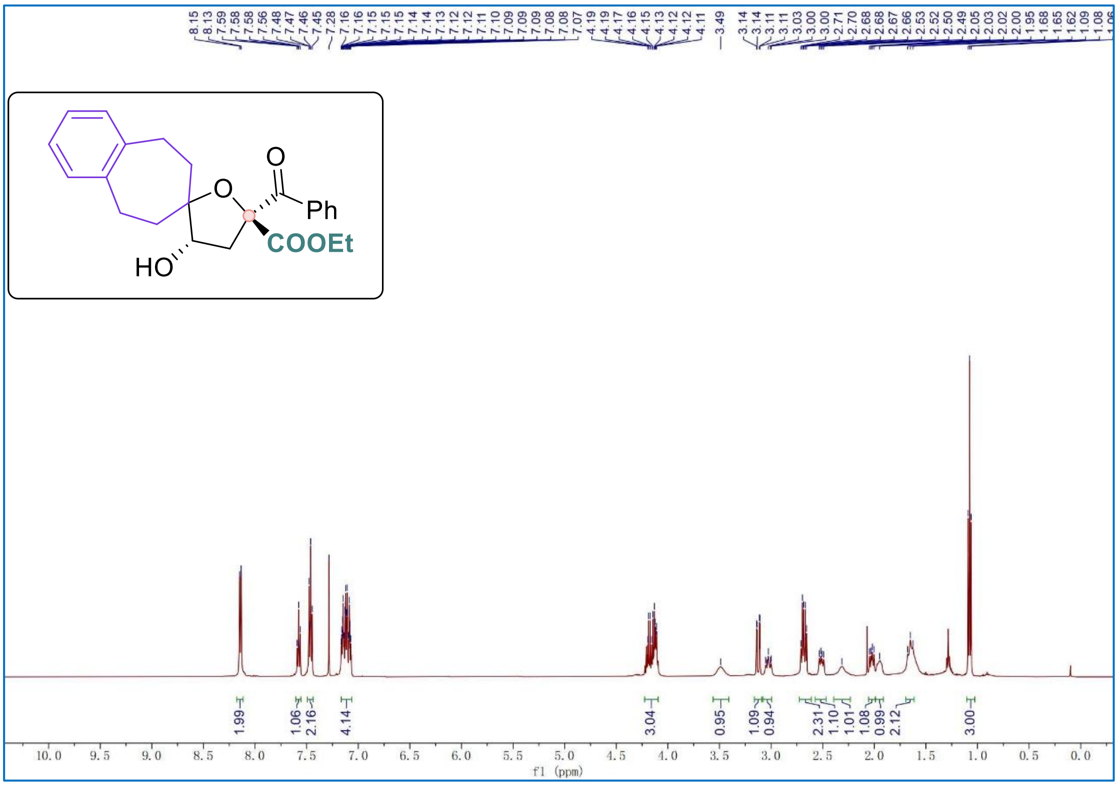

^{1}H NMR-spectrum (500 MHz, Chloroform-*d*) of **18**

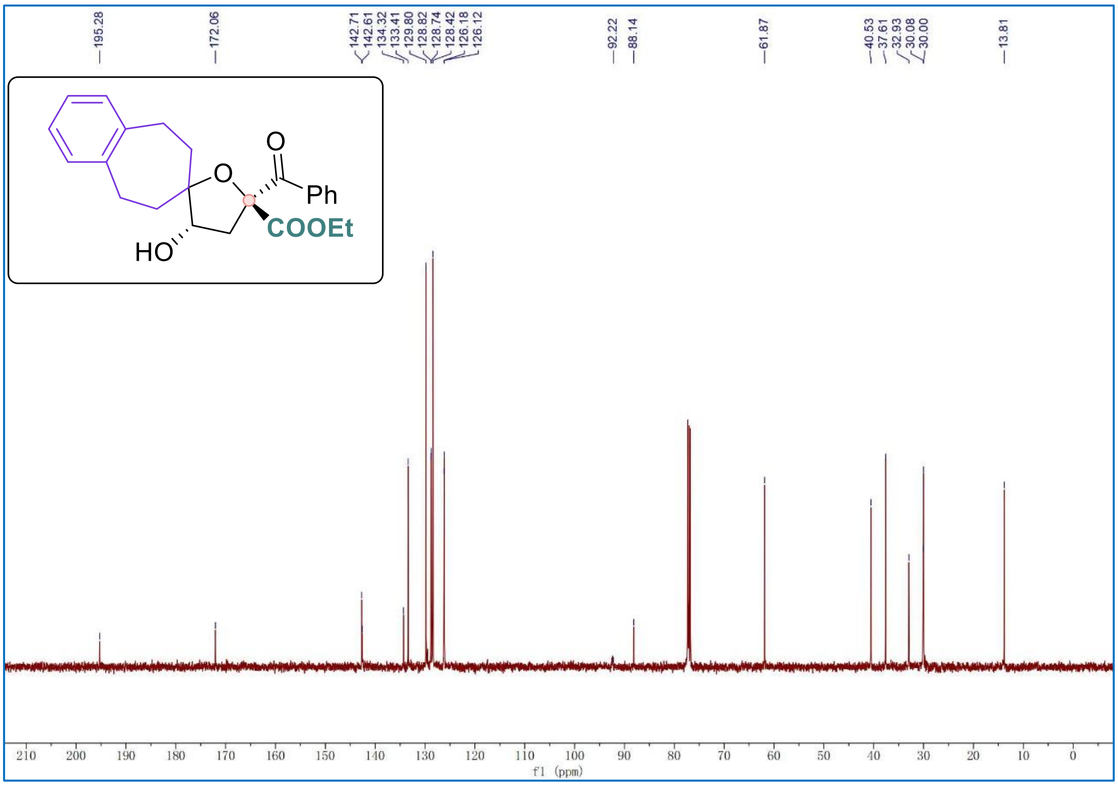

^{13}C NMR-spectrum (126 MHz, Chloroform-*d*) of **18**

ethyl 10-benzoyl-12-hydroxy-1,4,9-trioxadispiro[4.2.48.25]tetradecane-10-carboxylate (19)

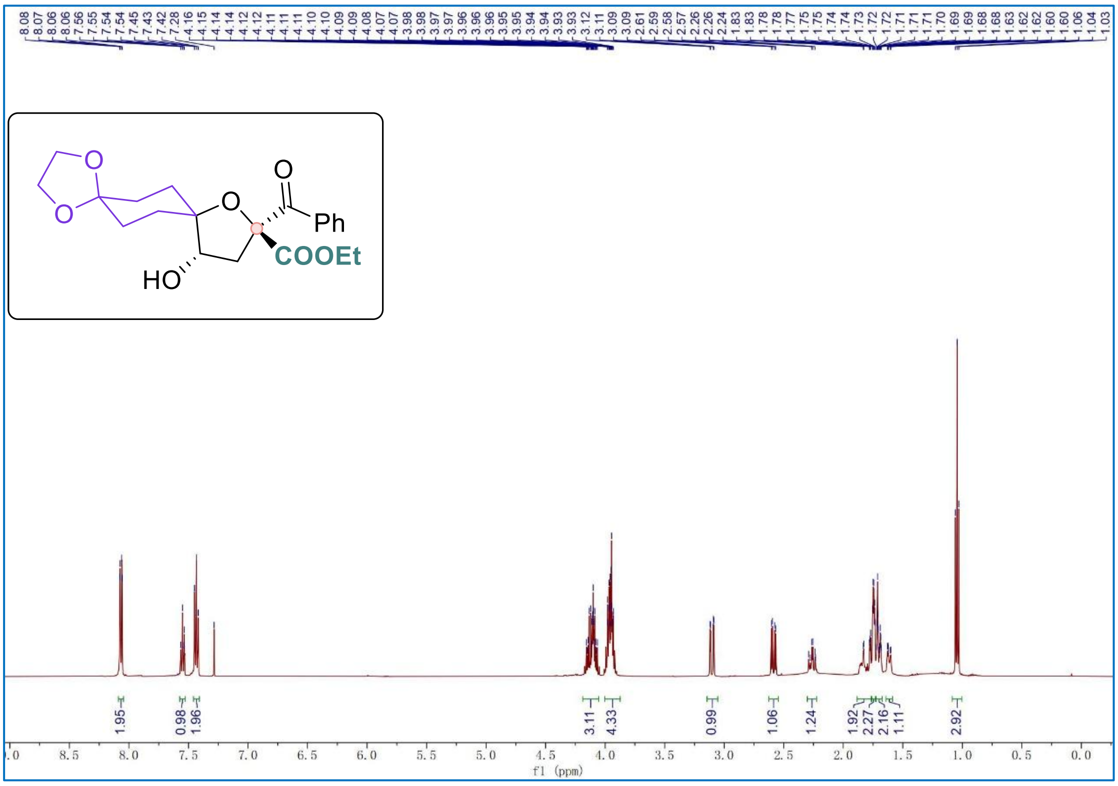

^{1}H NMR-spectrum (500 MHz, Chloroform-*d*) of **19**

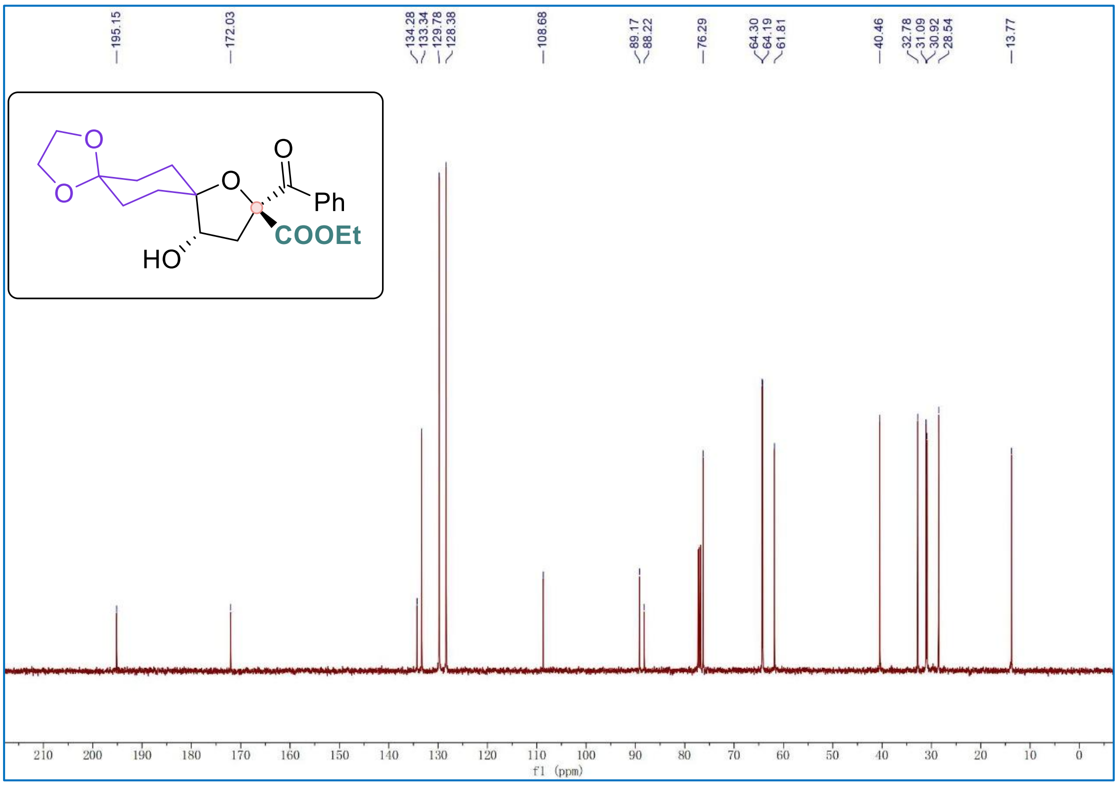

^{13}C NMR-spectrum (126 MHz, Chloroform-*d*) of **19**

ethyl 2-benzoyl-4-hydroxy-11,11-dimethyl-1,9,13-trioxadispiro[4.2.5^{8}.2^{5}]pentadecane-2-carboxylate (20)

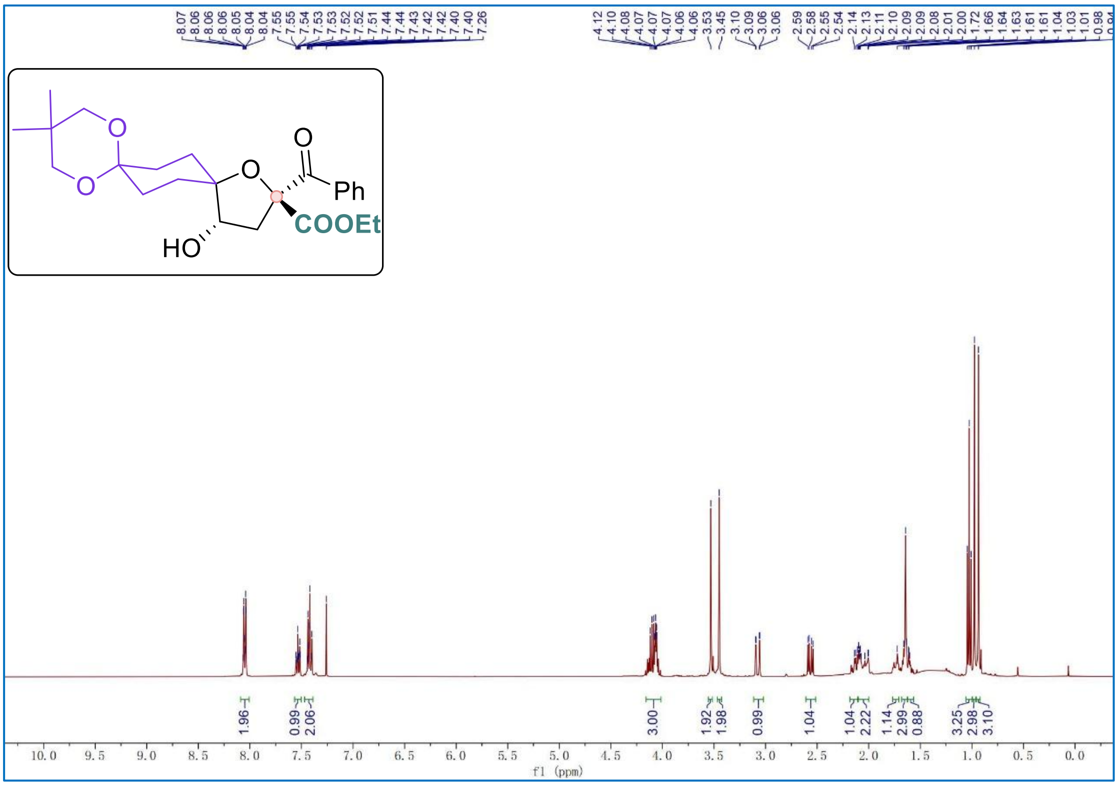

^{1}H NMR-spectrum (400 MHz, Chloroform-*d*) of **20**

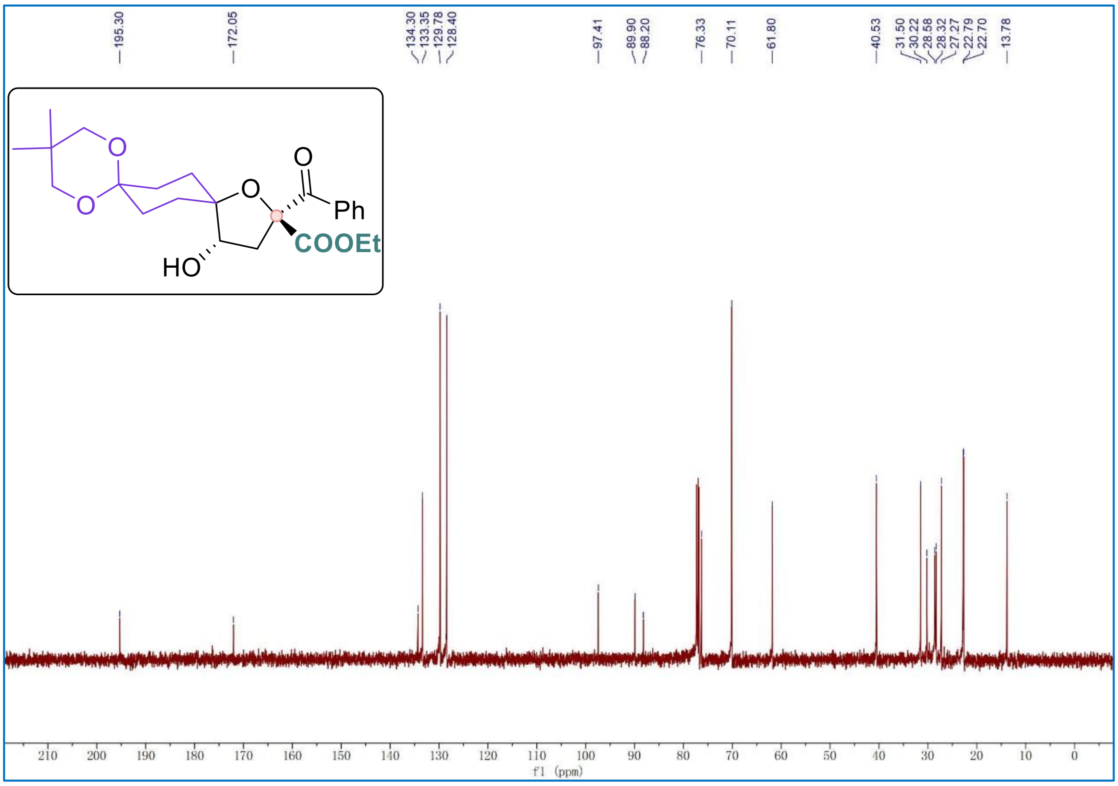

^{13}C NMR-spectrum (126 MHz, Chloroform-*d*) of **20**

ethyl 6-benzoyl-8-hydroxy-2,5-dioxaspiro[3.4]octane-6-carboxylate (21)

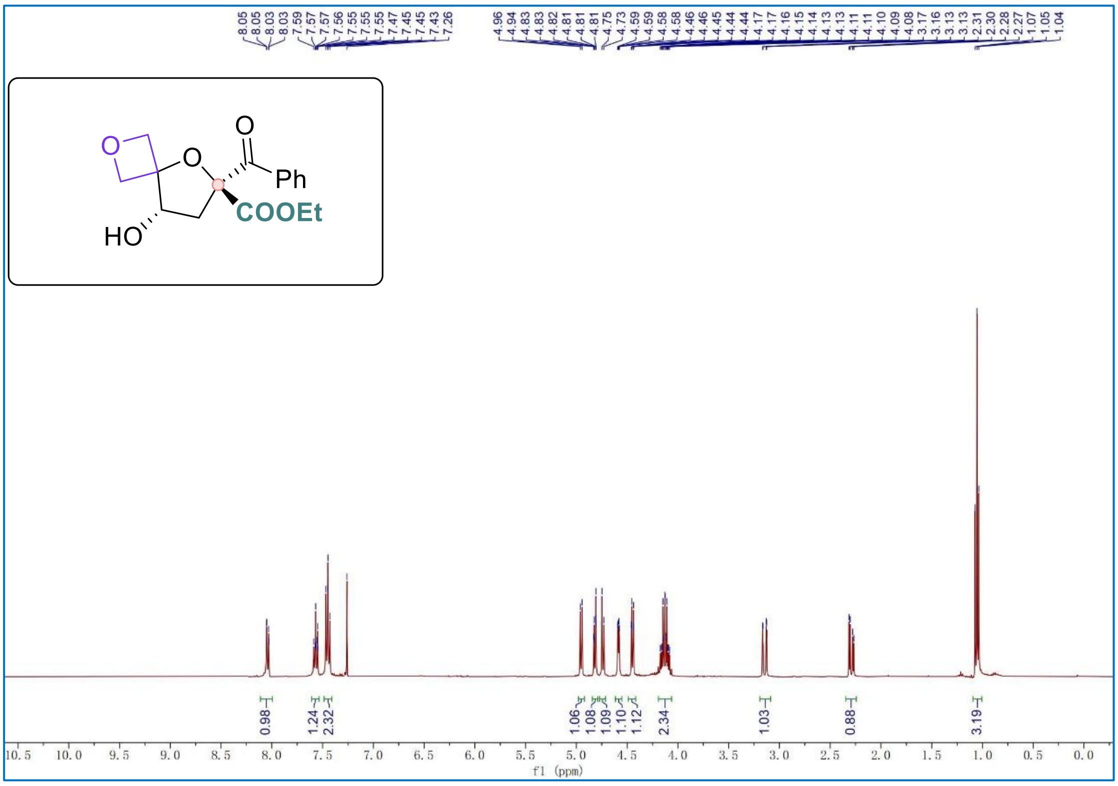

^{1}H NMR-spectrum (400 MHz, Chloroform-*d*) of **21**

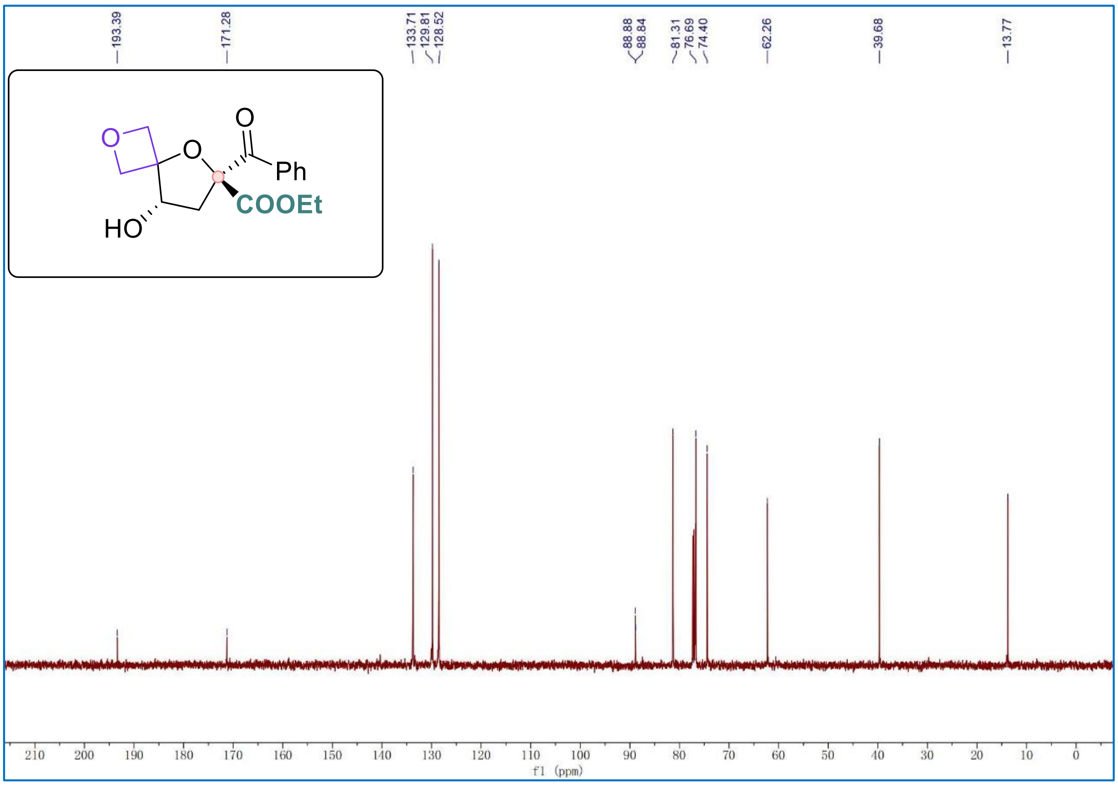

^{13}C NMR-spectrum (126 MHz, Chloroform-*d*) of **21**

ethyl 2-benzoyl-4-hydroxy-1,8-dioxaspiro[4.5]decane-2-carboxylate (22)

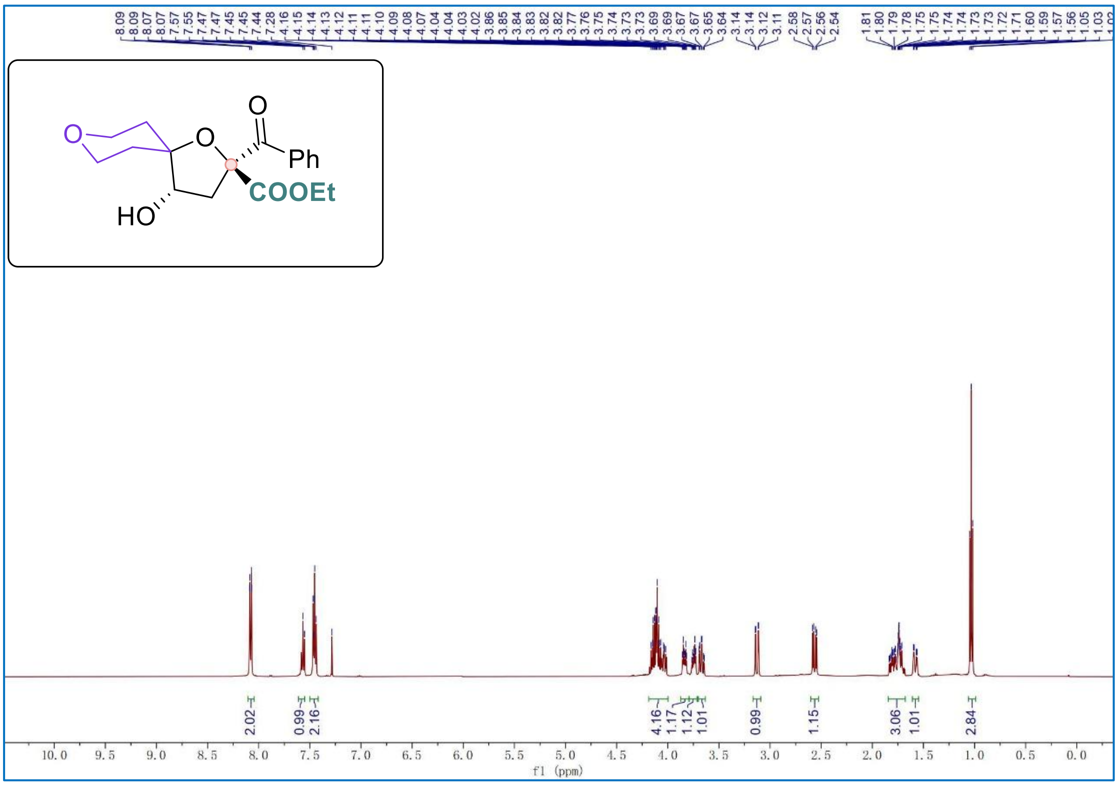

^{1}H NMR-spectrum (500 MHz, Chloroform-*d*) of **22**

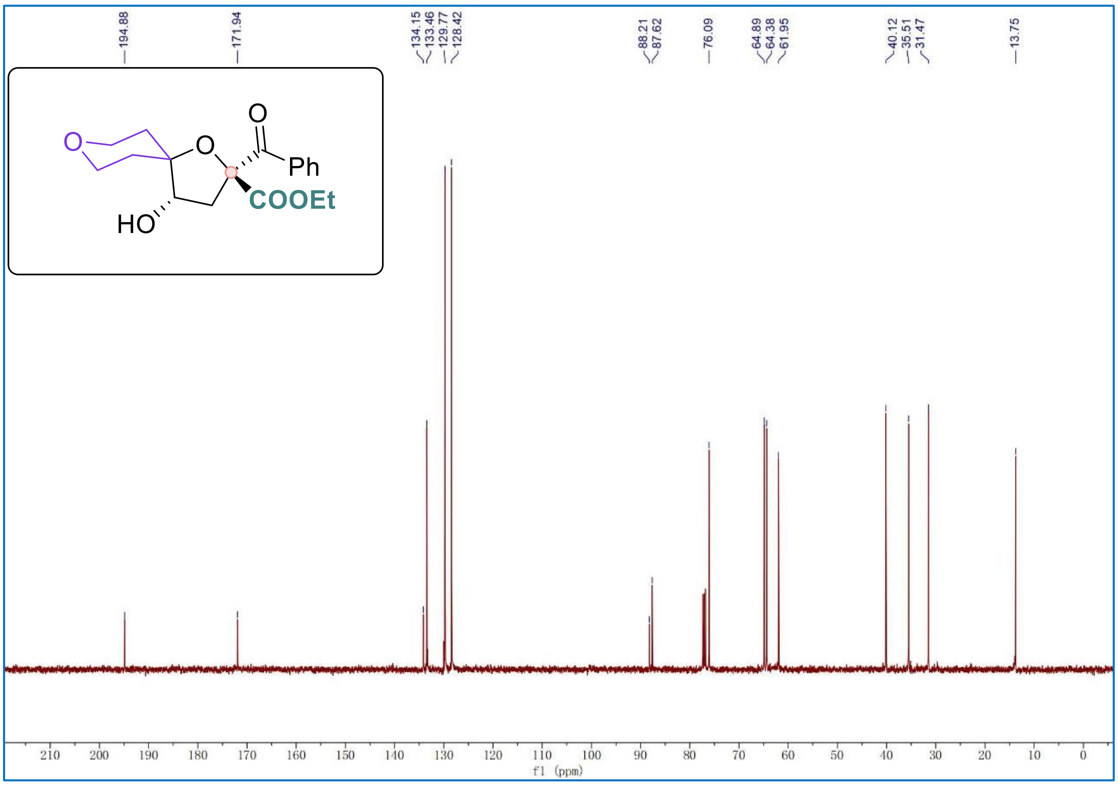

^{13}C NMR-spectrum (126 MHz, Chloroform-*d*) of **22**

ethyl 2-benzoyl-4-hydroxy-1,7-dioxaspiro[4.5]decane-2-carboxylate (23)

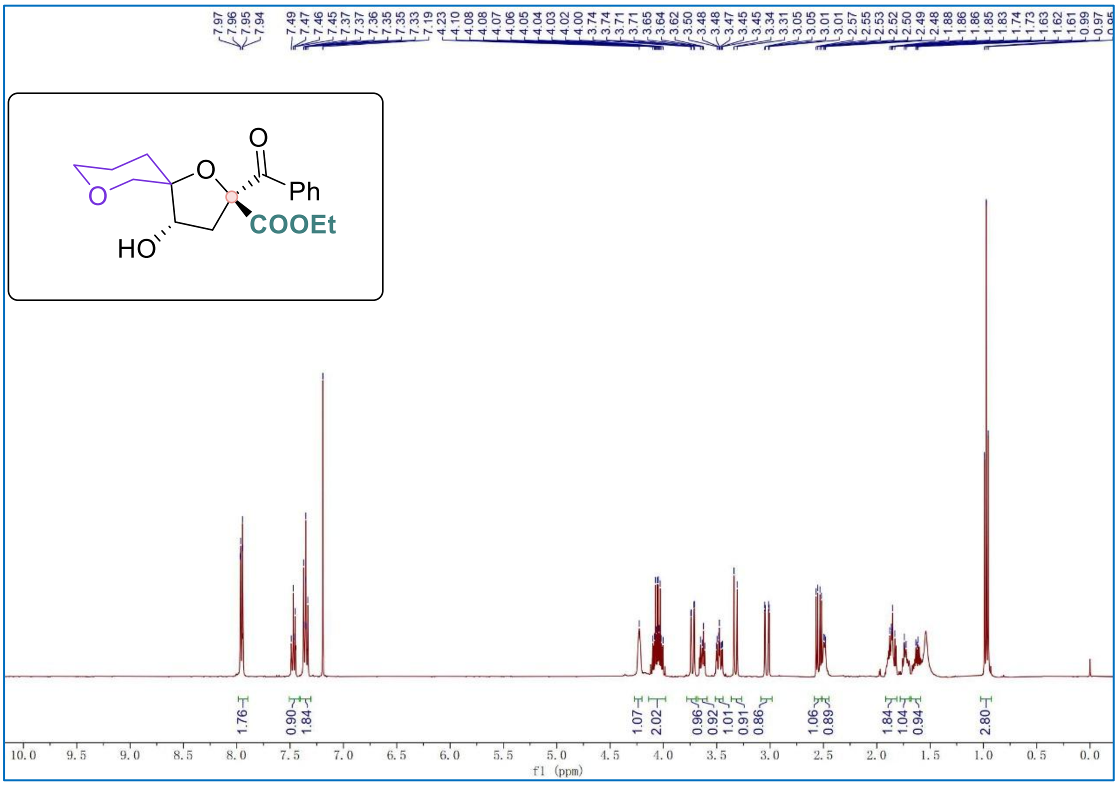

^{1}H NMR-spectrum (400 MHz, Chloroform-*d*) of **23**

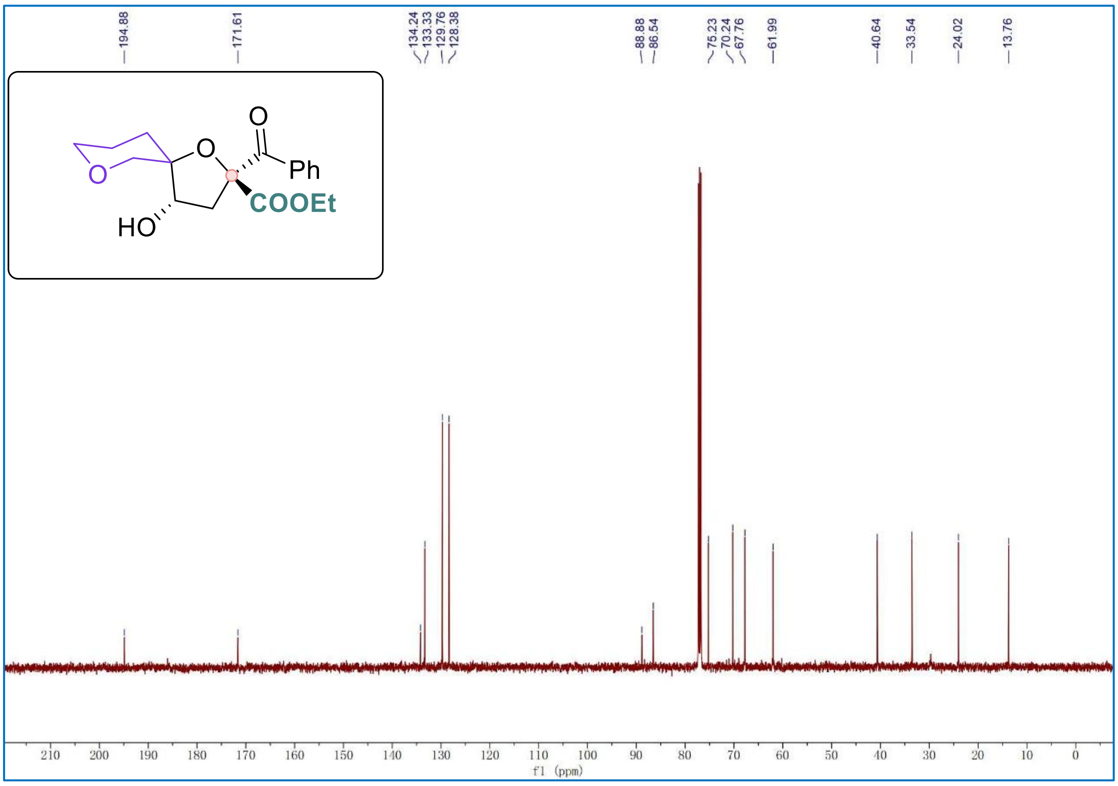

^{13}C NMR-spectrum (126 MHz, Chloroform-*d*) of **23**

ethyl 2-benzoyl-4-hydroxy-7,7-dimethyl-1,8-dioxaspiro[4.5]decane-2-carboxylate (24)

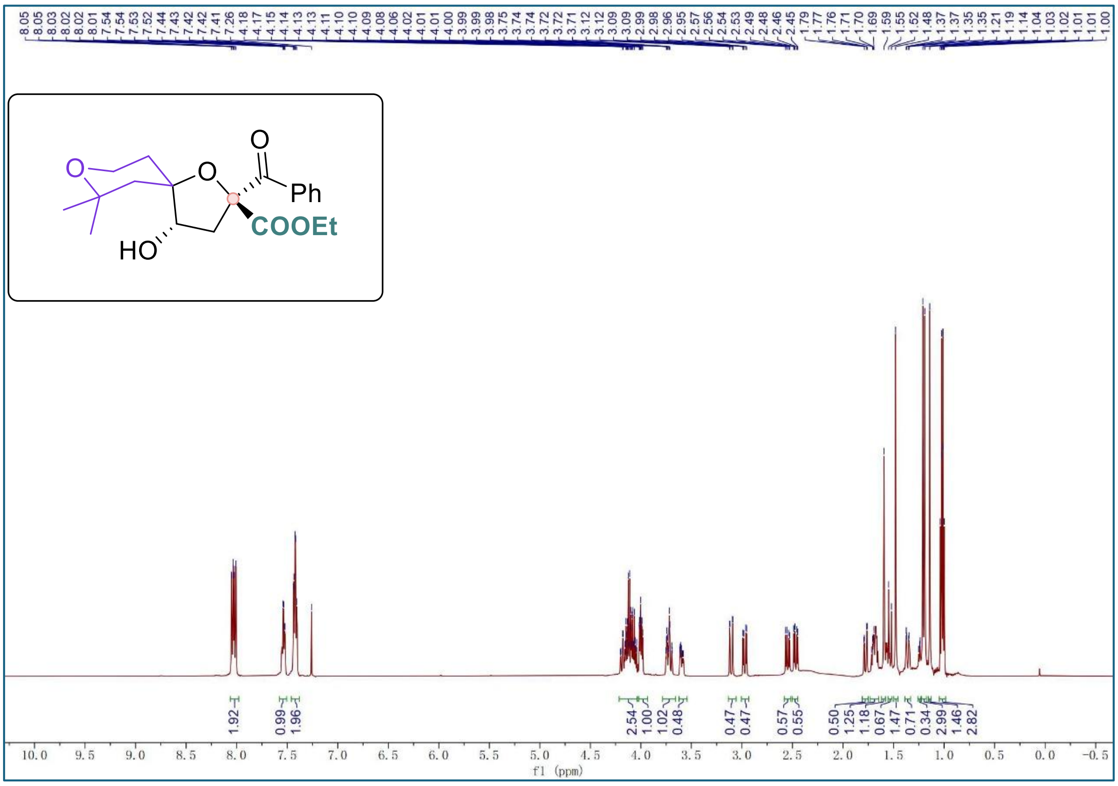

^{1}H NMR-spectrum (500 MHz, Chloroform-*d*) of **24**

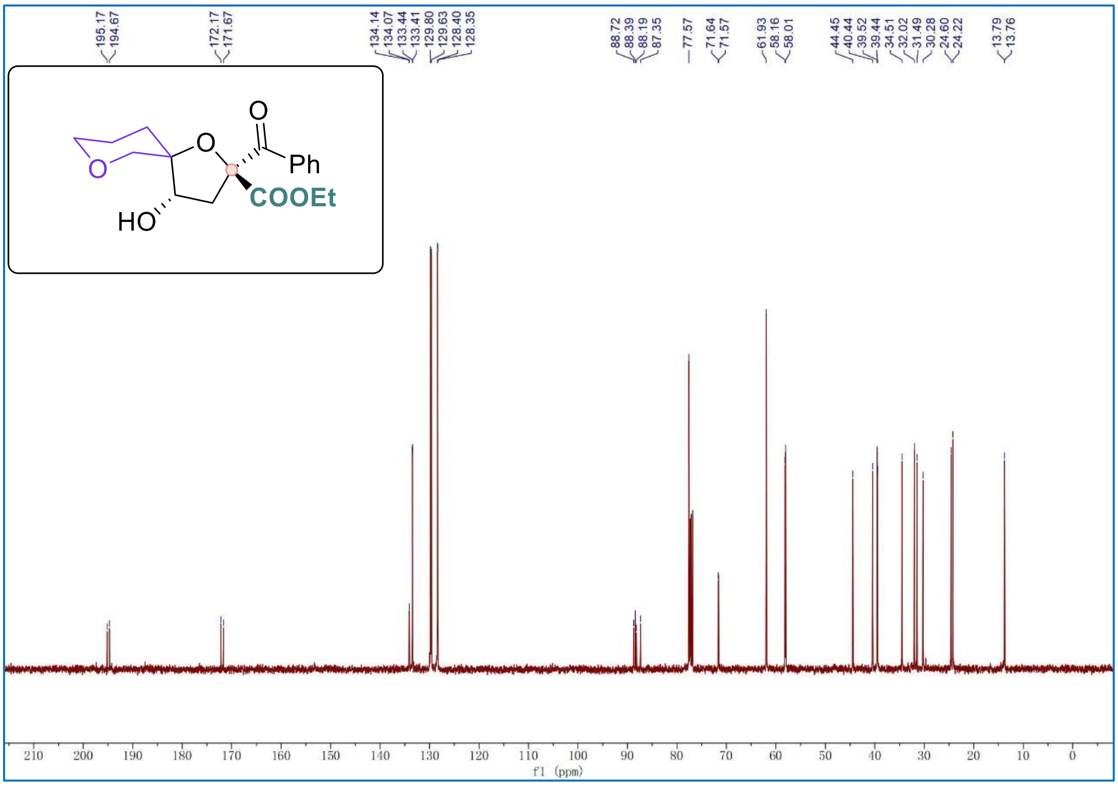

^{13}C NMR-spectrum (126 MHz, Chloroform-*d*) of **24**

8-(tert-butyl) 2-ethyl 2-benzoyl-4-hydroxy-1-oxa-8-azaspiro[4.5]decane-2,8-dicarboxylate (25)

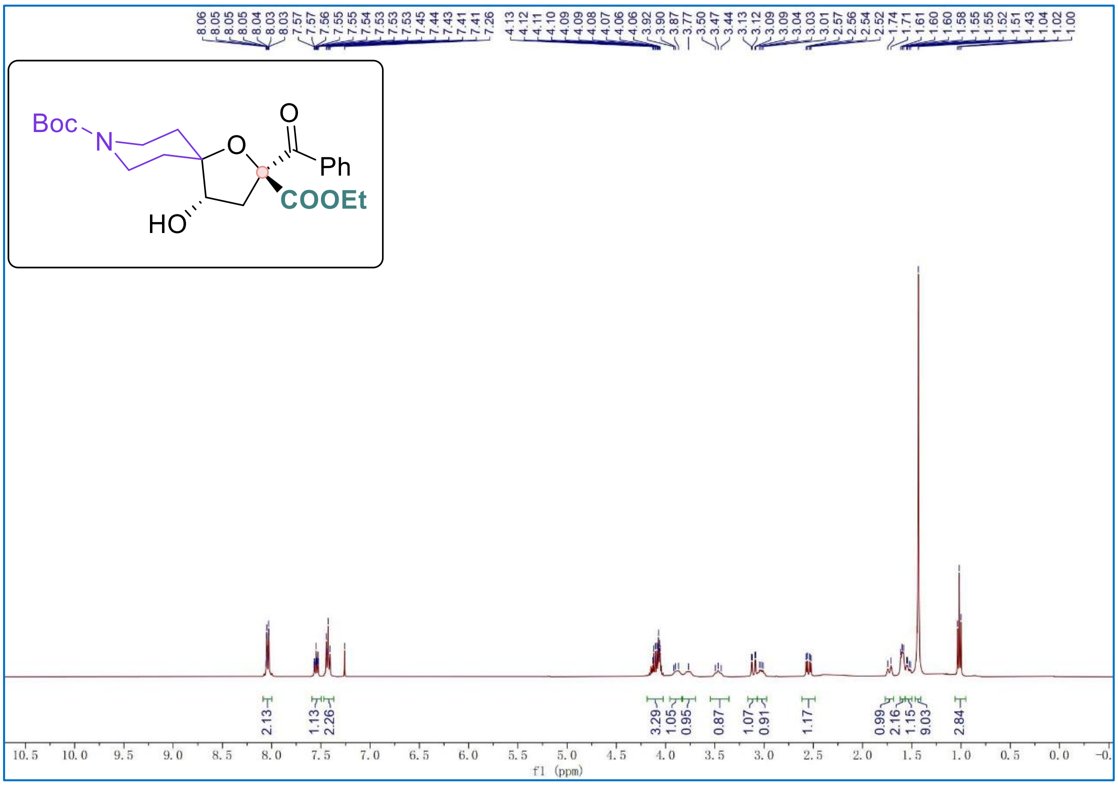

^{1}H NMR-spectrum (400 MHz, Chloroform-*d*) of **25**

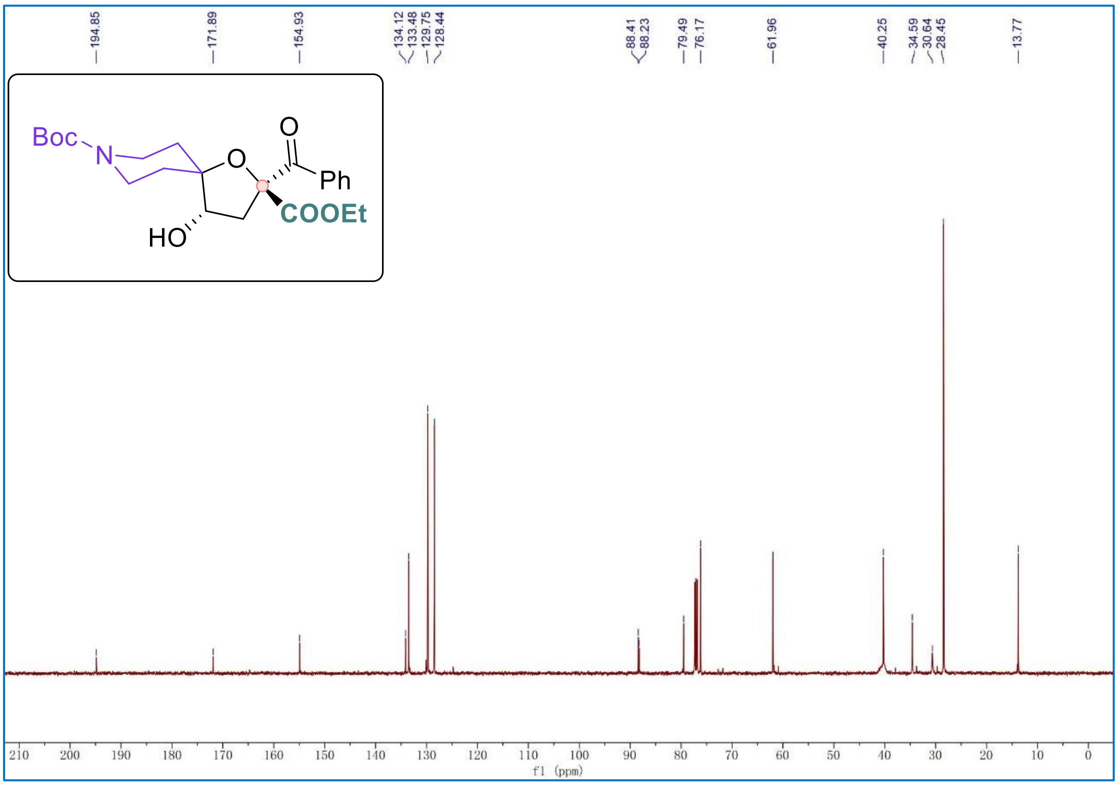

^{13}C NMR-spectrum (126 MHz, Chloroform-*d*) of **25**

ethyl 2-benzoyl-4-hydroxy-1-oxa-8-thiaspiro[4.5]decane-2-carboxylate 8,8-dioxide (26)

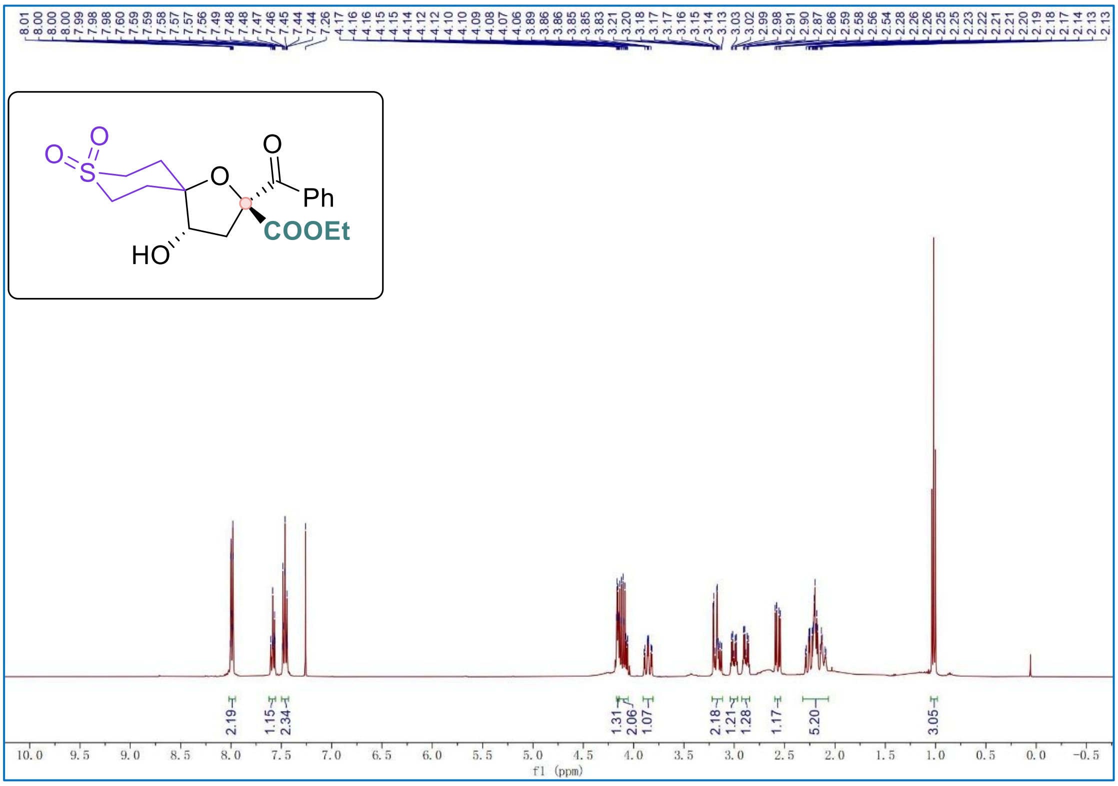

^{1}H NMR-spectrum (400 MHz, Chloroform-*d*) of **26**

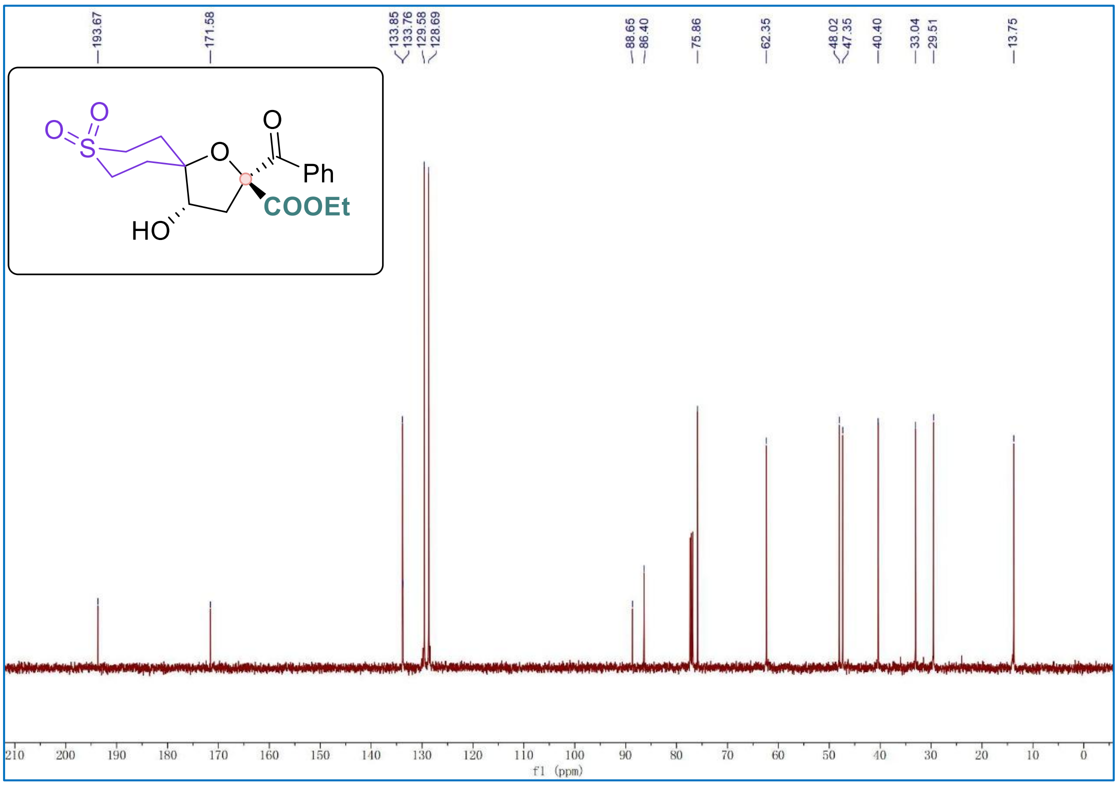

^{13}C NMR-spectrum (126 MHz, Chloroform-*d*) of **26**

ethyl 2-benzoyl-4-hydroxy-5,5-dimethyltetrahydrofuran-2-carboxylate (27)

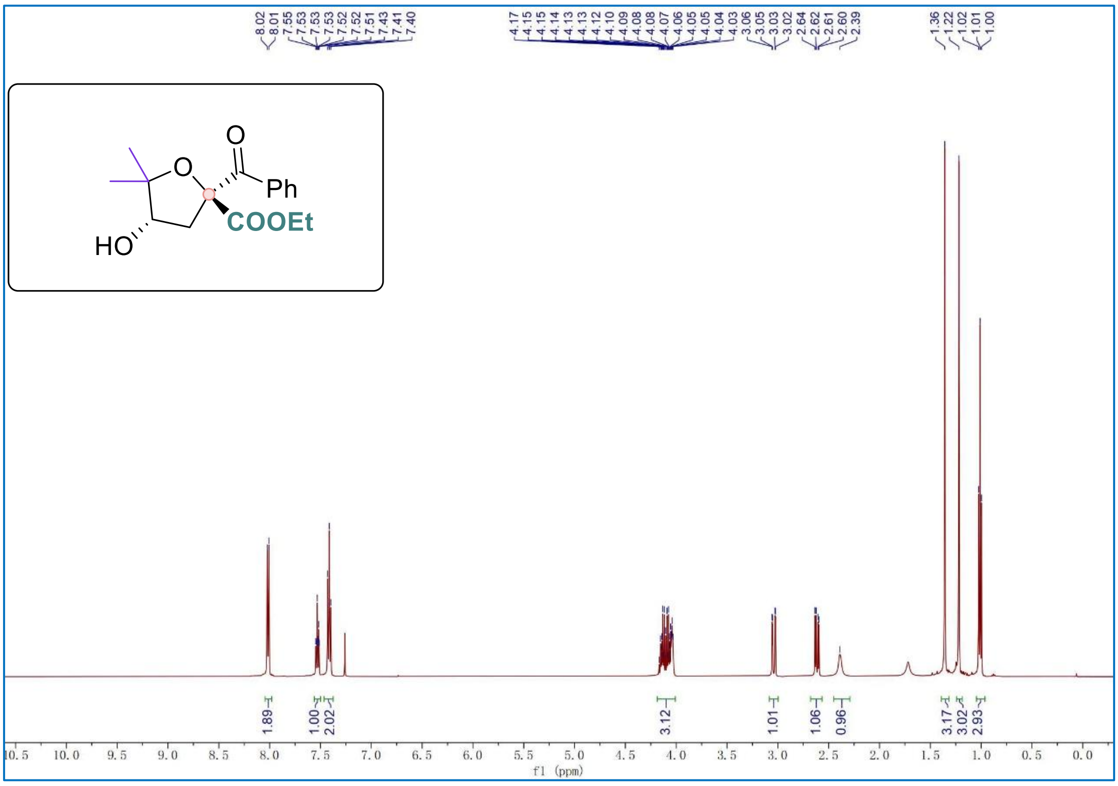

^{1}H NMR-spectrum (500 MHz, Chloroform-*d*) of **27**

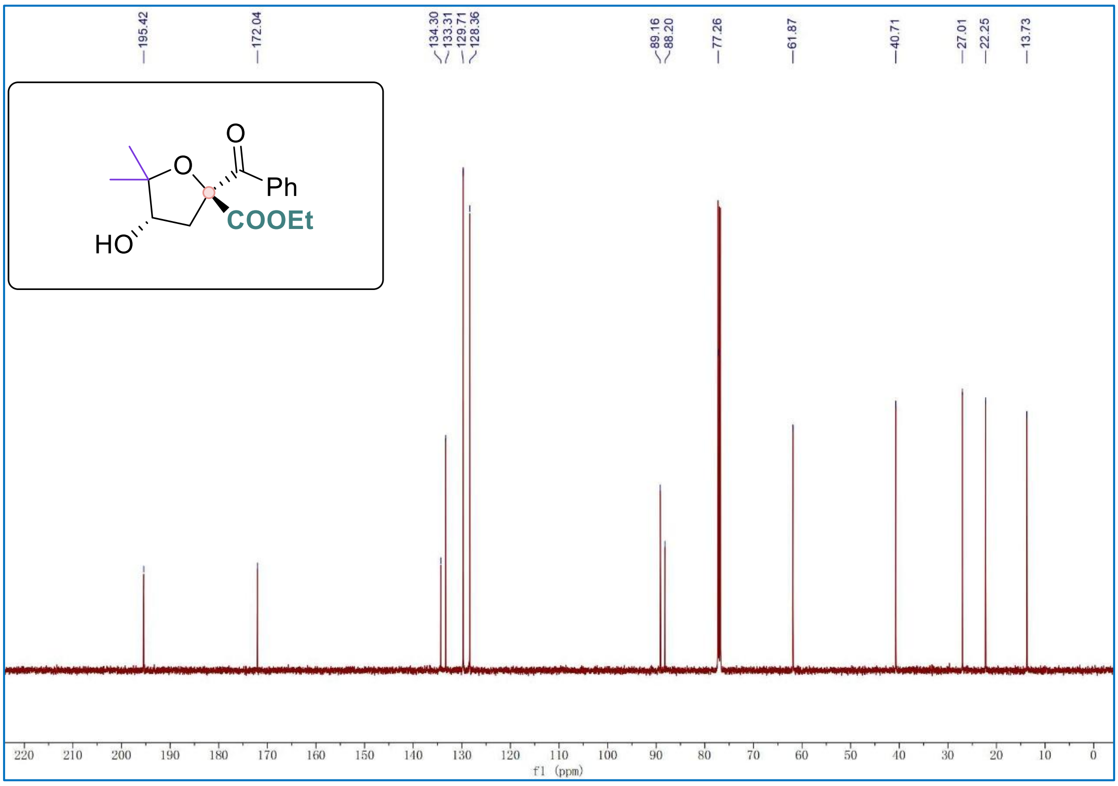

^{13}C NMR-spectrum (126 MHz, Chloroform-*d*) of **27**

ethyl 2-benzoyl-5,5-diethyl-4-hydroxytetrahydrofuran-2-carboxylate (28)

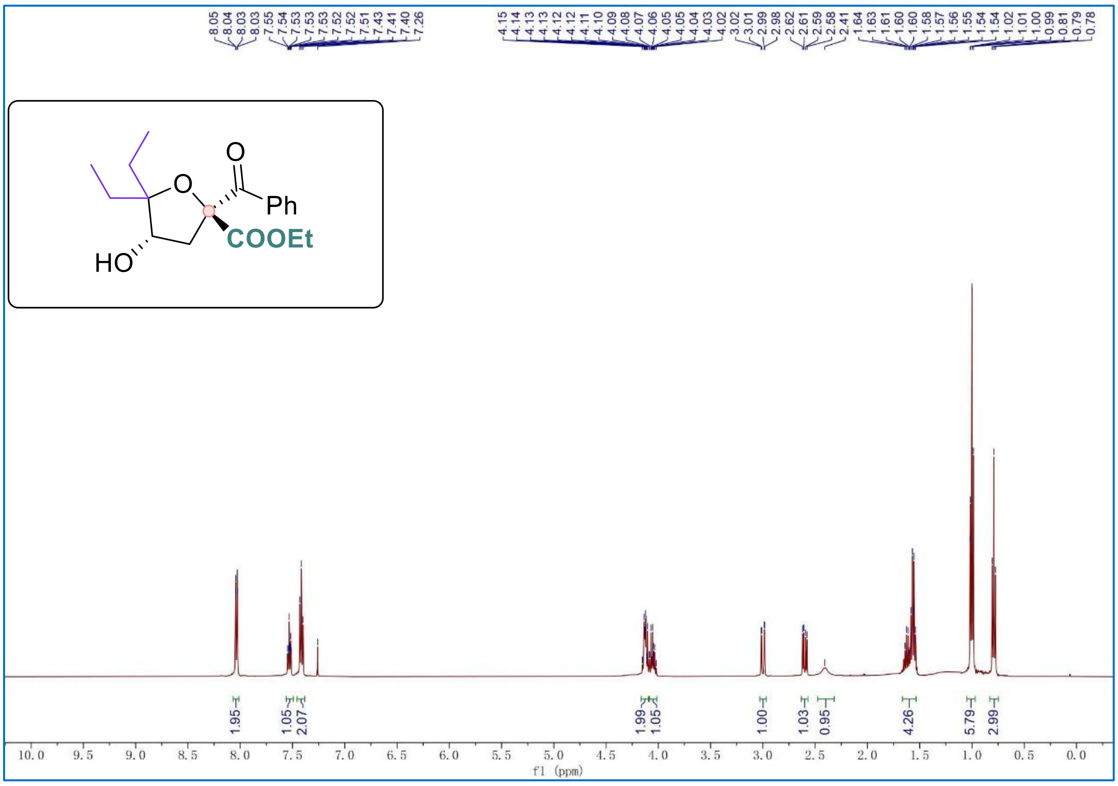

^{1}H NMR-spectrum (500 MHz, Chloroform-*d*) of **28**

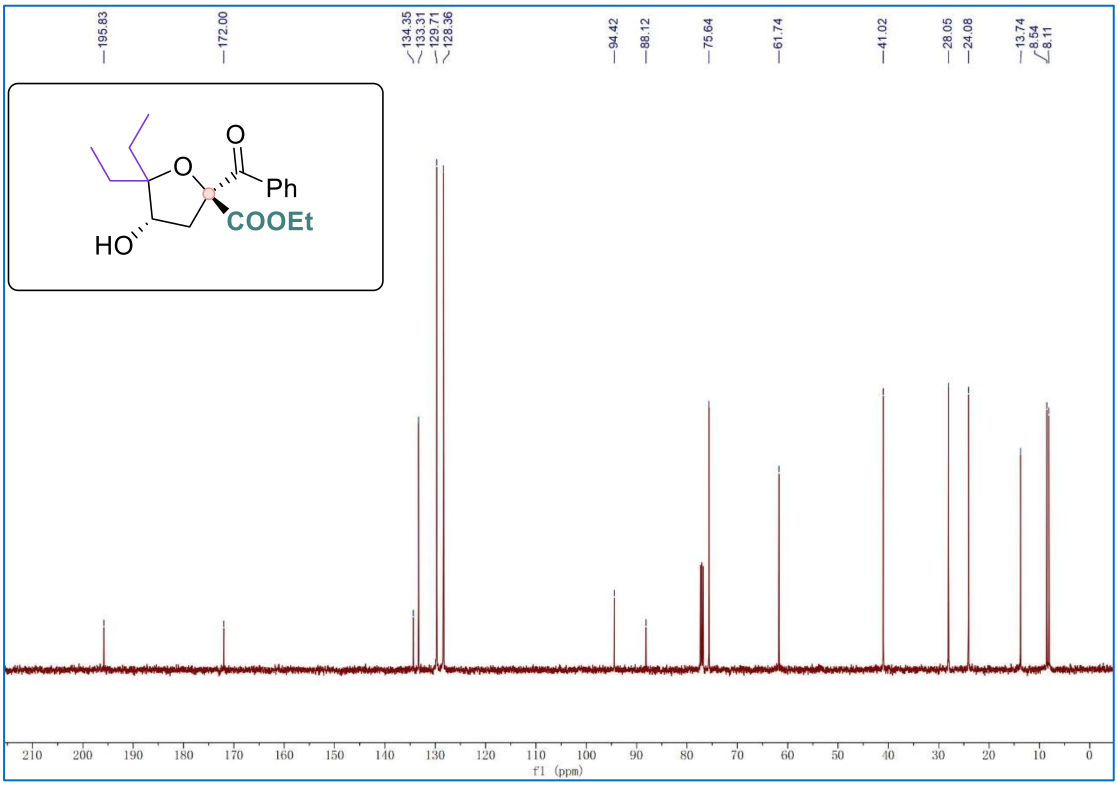

^{13}C NMR-spectrum (126 MHz, Chloroform-*d*) of **28**

ethyl 2-benzoyl-4-hydroxy-5,5-diisobutyltetrahydrofuran-2-carboxylate (29)

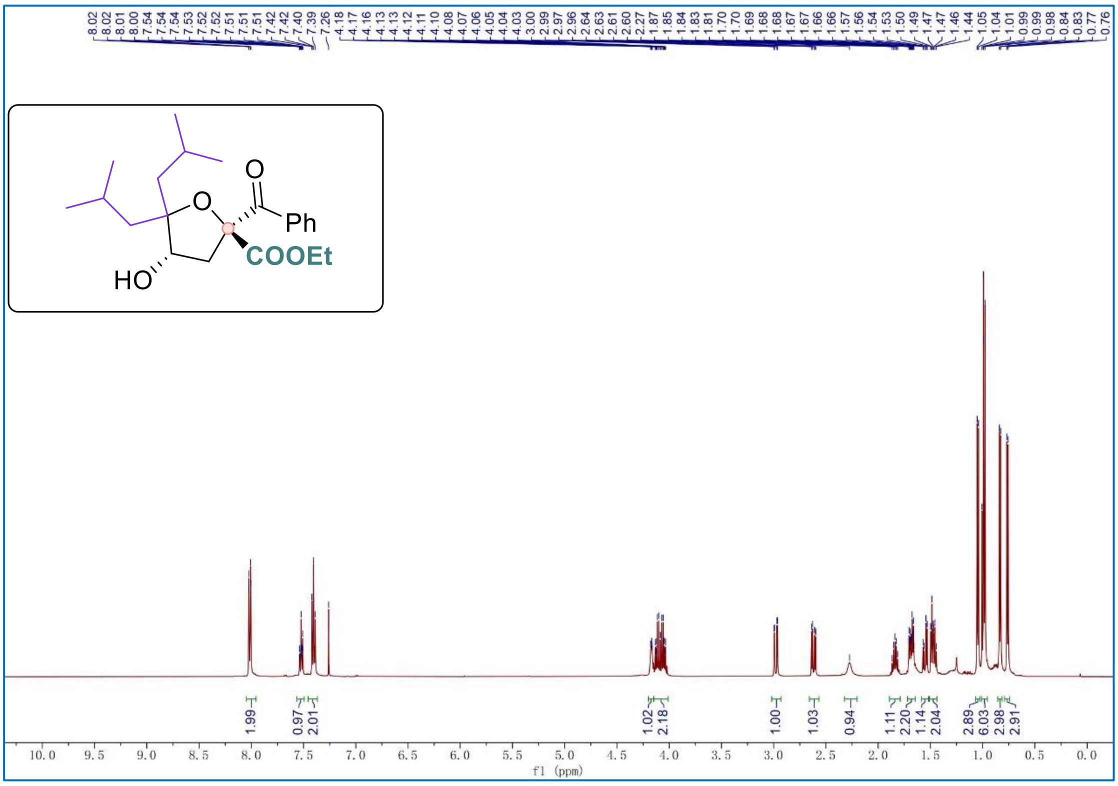

^{1}H NMR-spectrum (500 MHz, Chloroform-*d*) of **29**

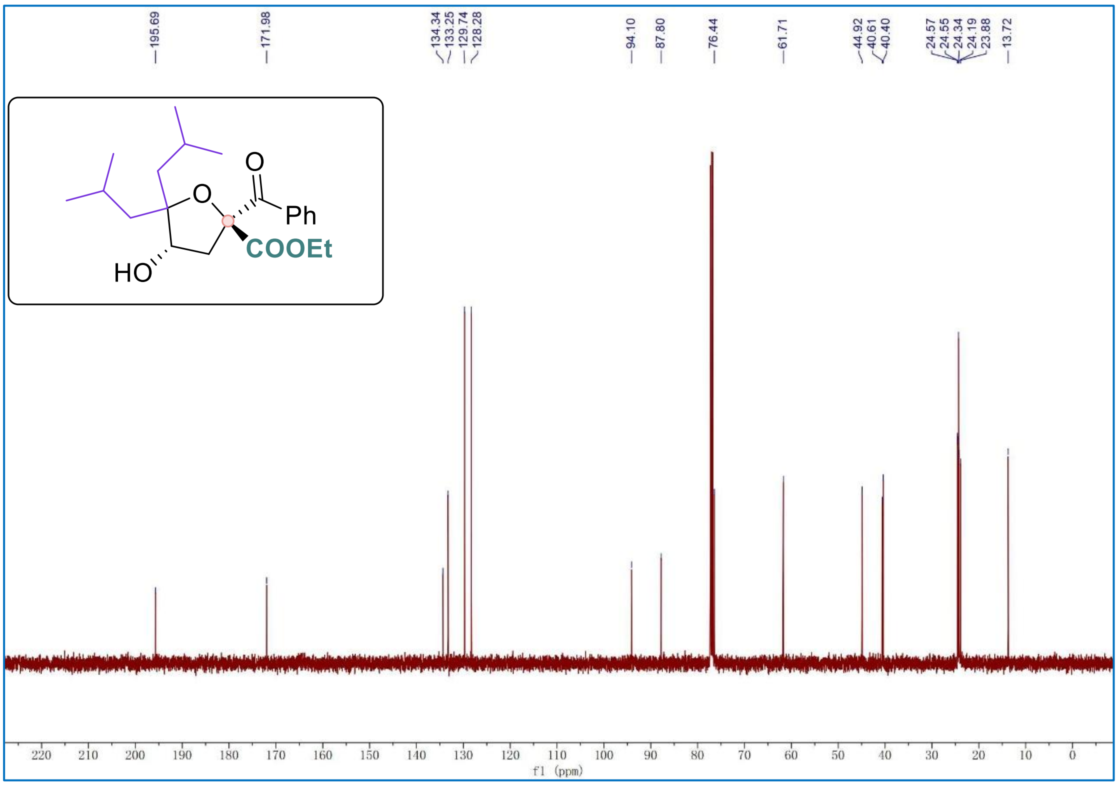

^{13}C NMR-spectrum (126 MHz, Chloroform-*d*) of **29**

ethyl 2-benzoyl-5-ethyl-4-hydroxy-5-methyltetrahydrofuran-2-carboxylate (30)

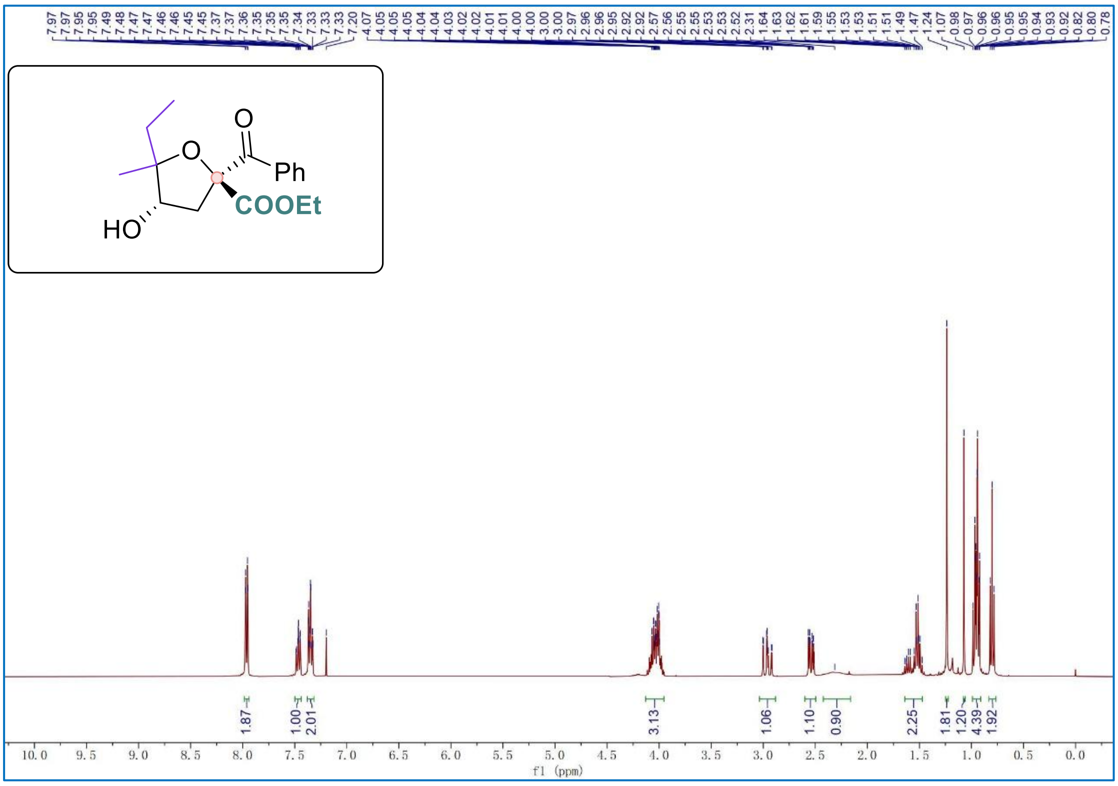

^{1}H NMR-spectrum (400 MHz, Chloroform-*d*) of **30**

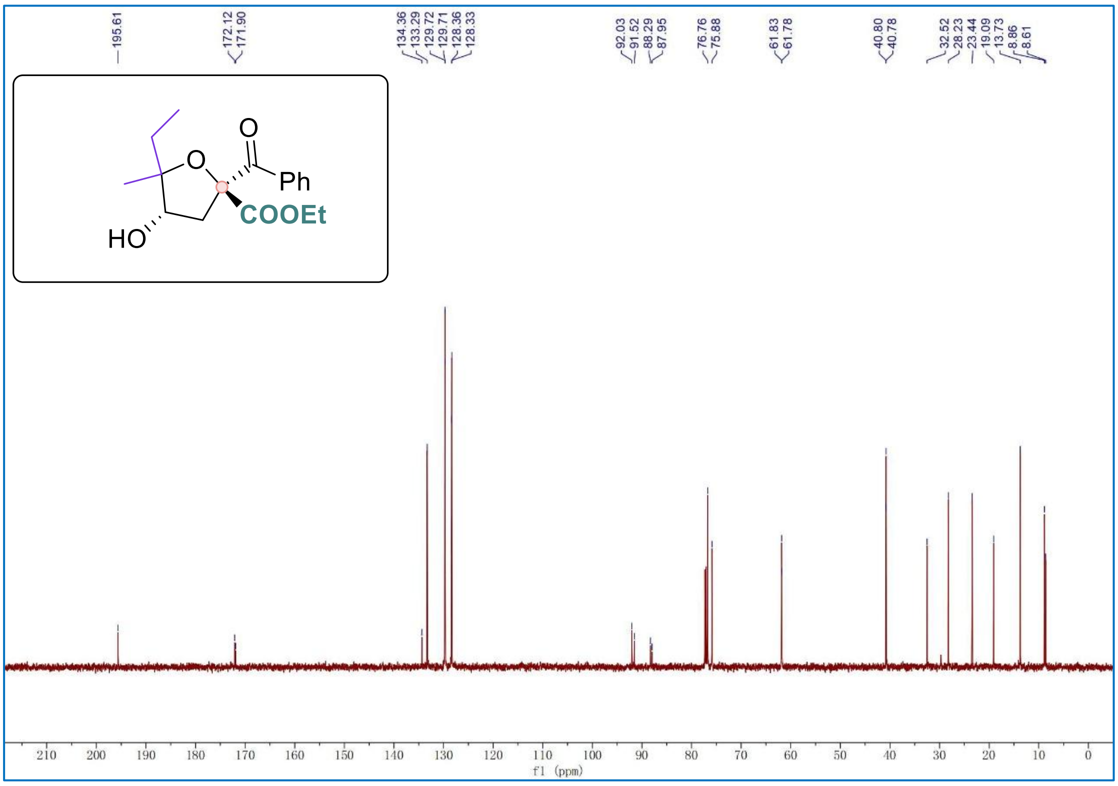

^{13}C NMR-spectrum (126 MHz, Chloroform-*d*) of **30**

ethyl 2-benzoyl-5-cyclohexyl-4-hydroxy-5-methyltetrahydrofuran-2-carboxylate (31)

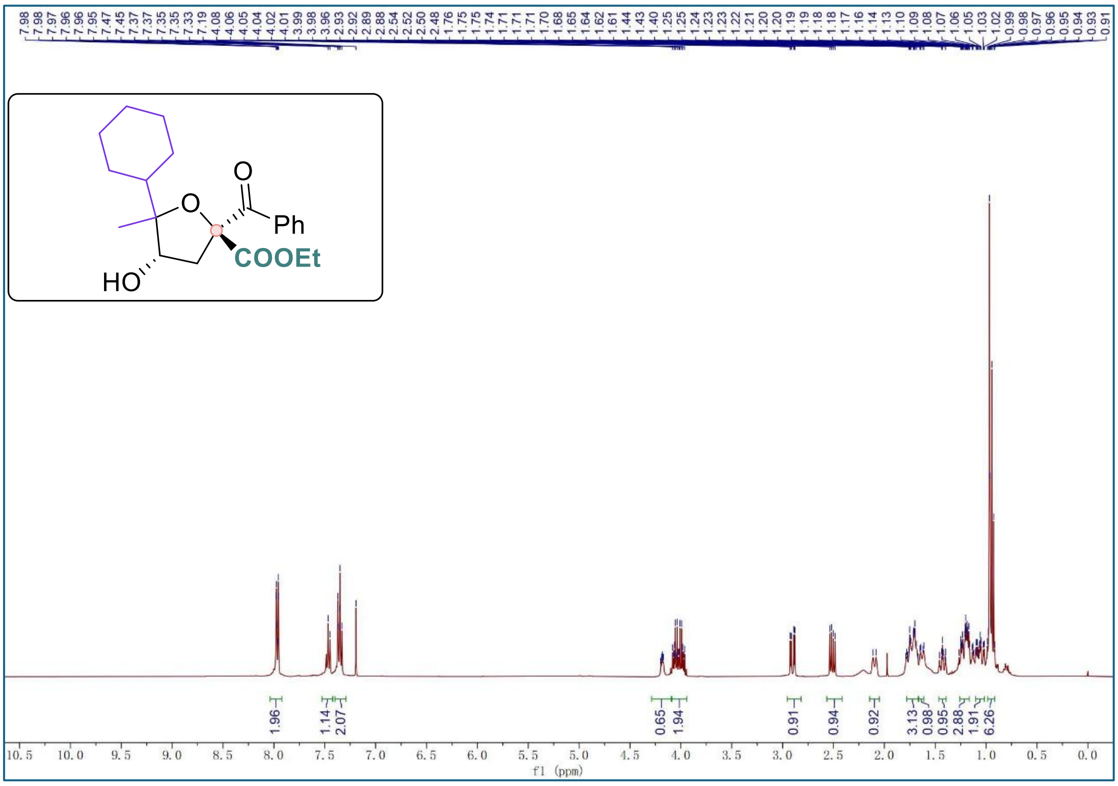

^{1}H NMR-spectrum (400 MHz, Chloroform-*d*) of **31**

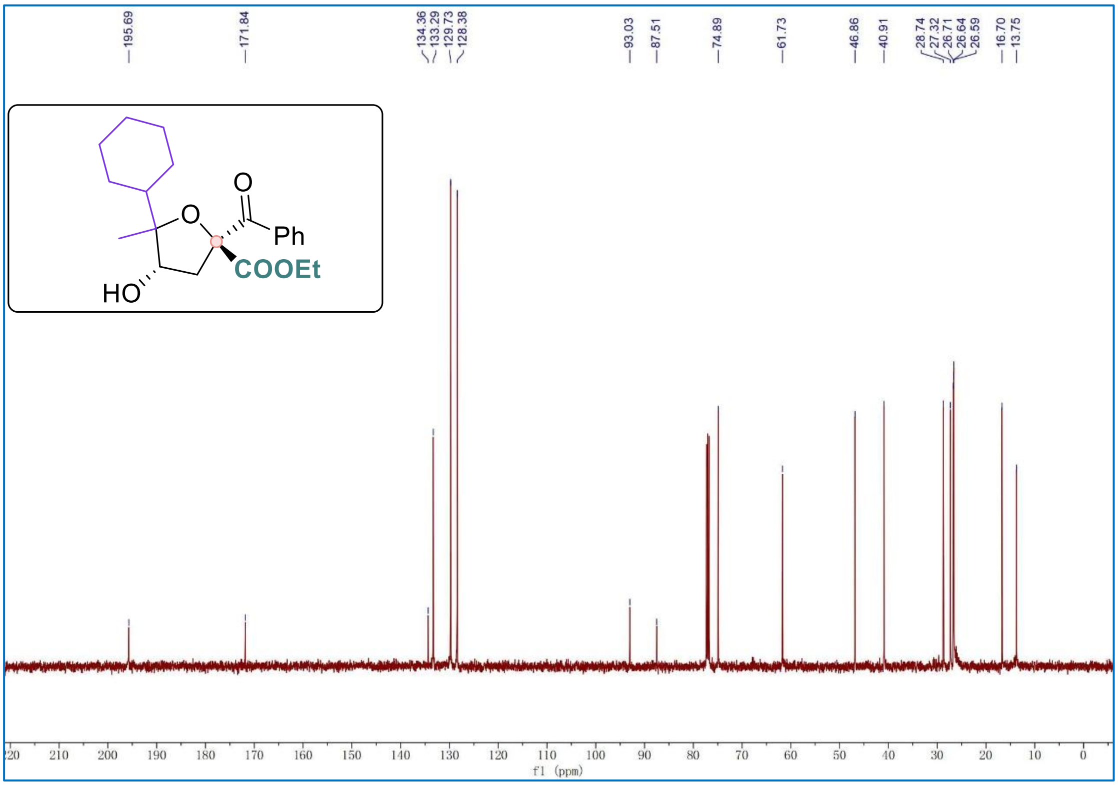

^{13}C NMR-spectrum (126 MHz, Chloroform-*d*) of **31**

SUPPORTING INFORMATION

ethyl (1S,5S,6S)-5'-benzoyl-3'-hydroxydihydro-3'H-7,8-dioxaspiro[bicyclo[3.2.1]octane-6,2'-furan]-5'-carboxylate (32)

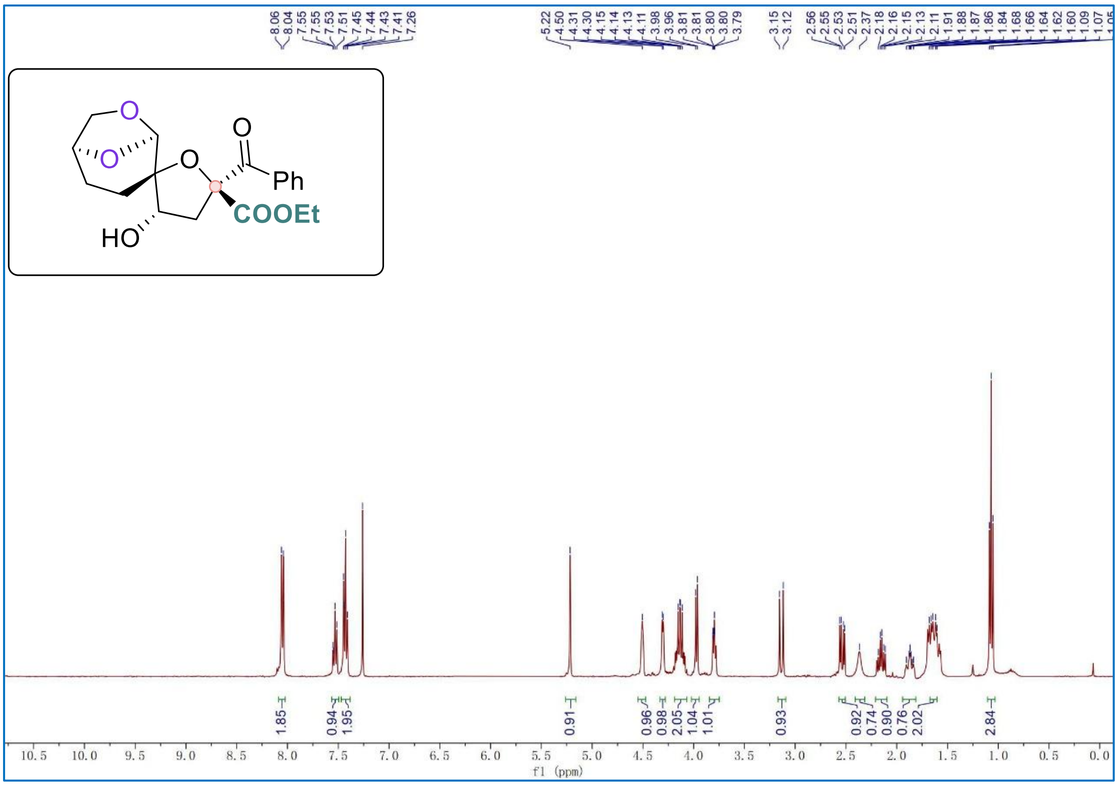

^{1}H NMR-spectrum (400 MHz, Chloroform-*d*) of **32**

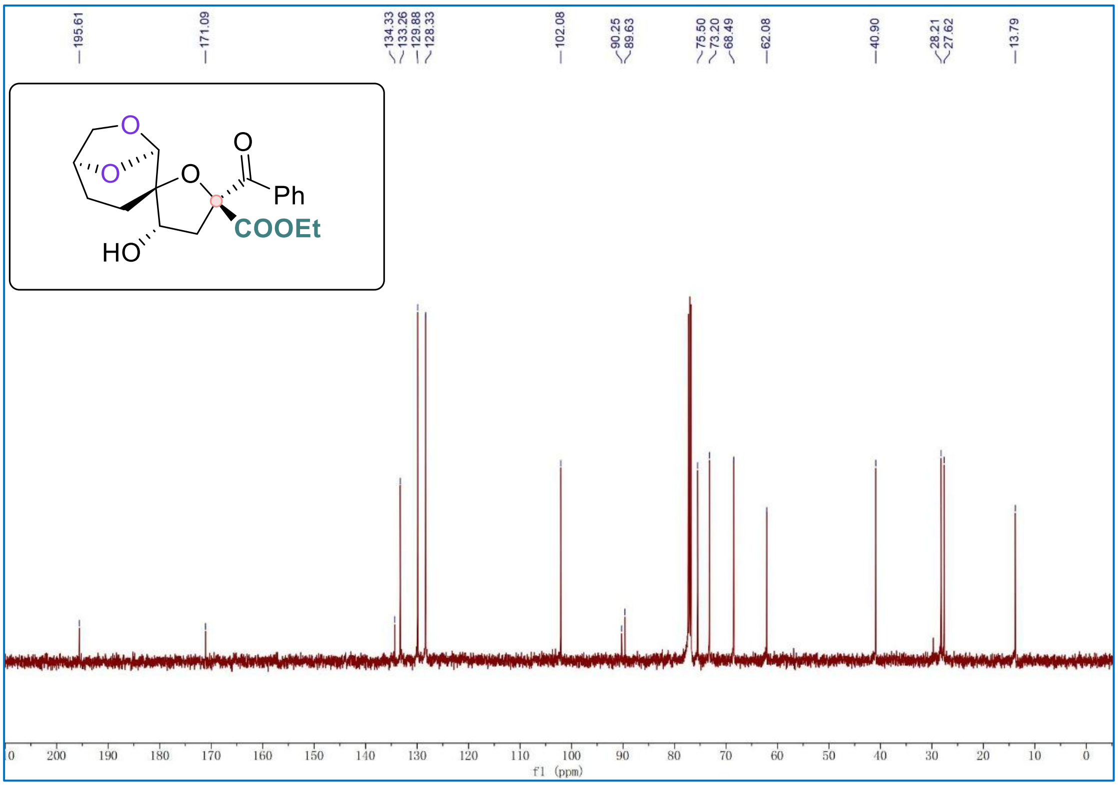

^{13}C NMR-spectrum (126 MHz, Chloroform-*d*) of **32**

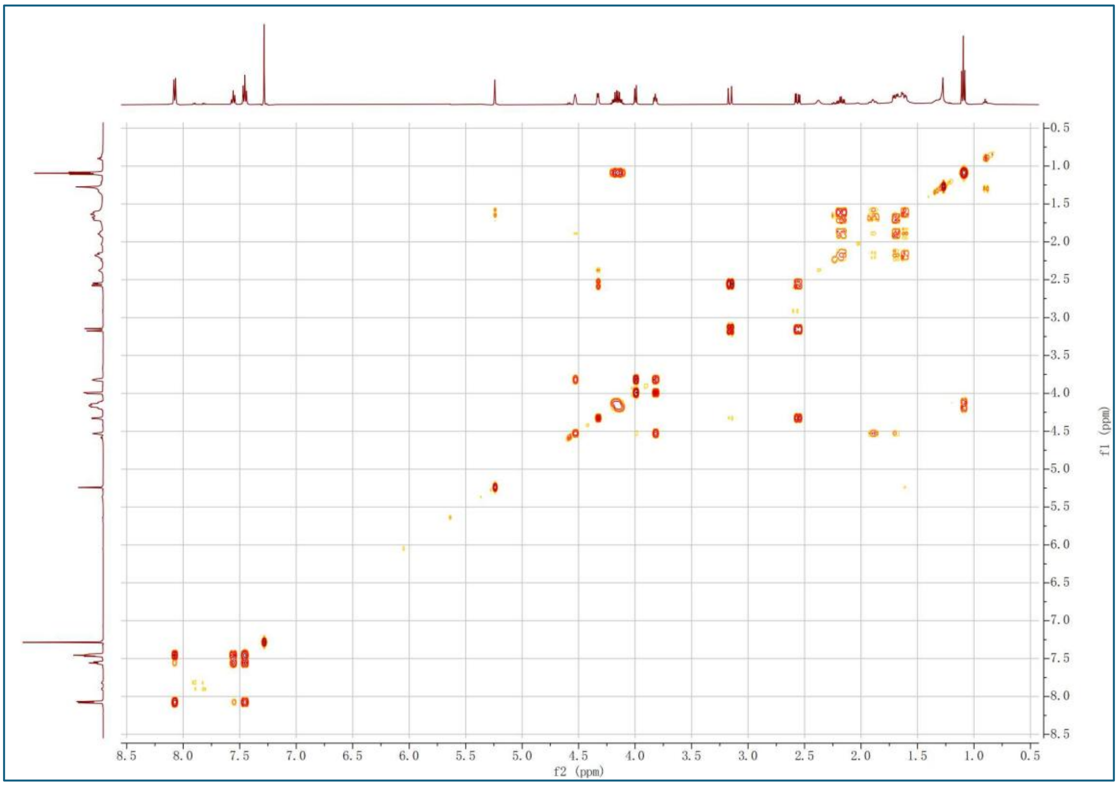

COSY of **32**

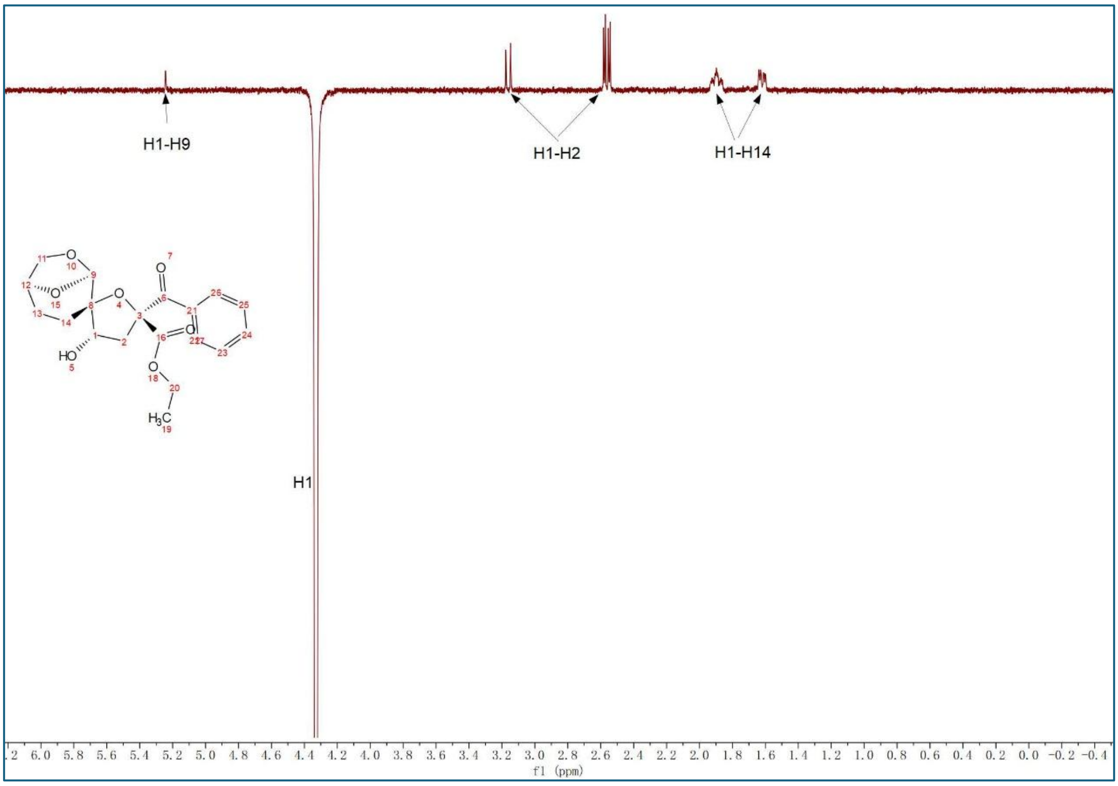

1D NOESY experiment with selective excitation of H1

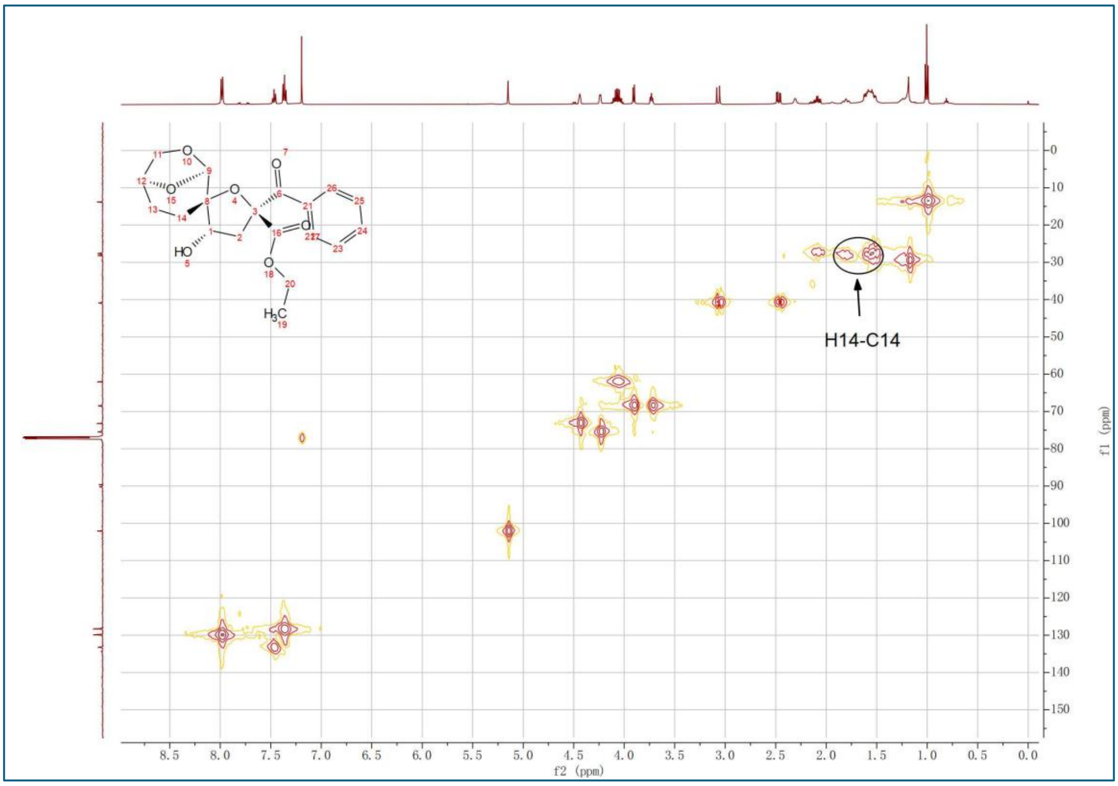

HMQC of **32**

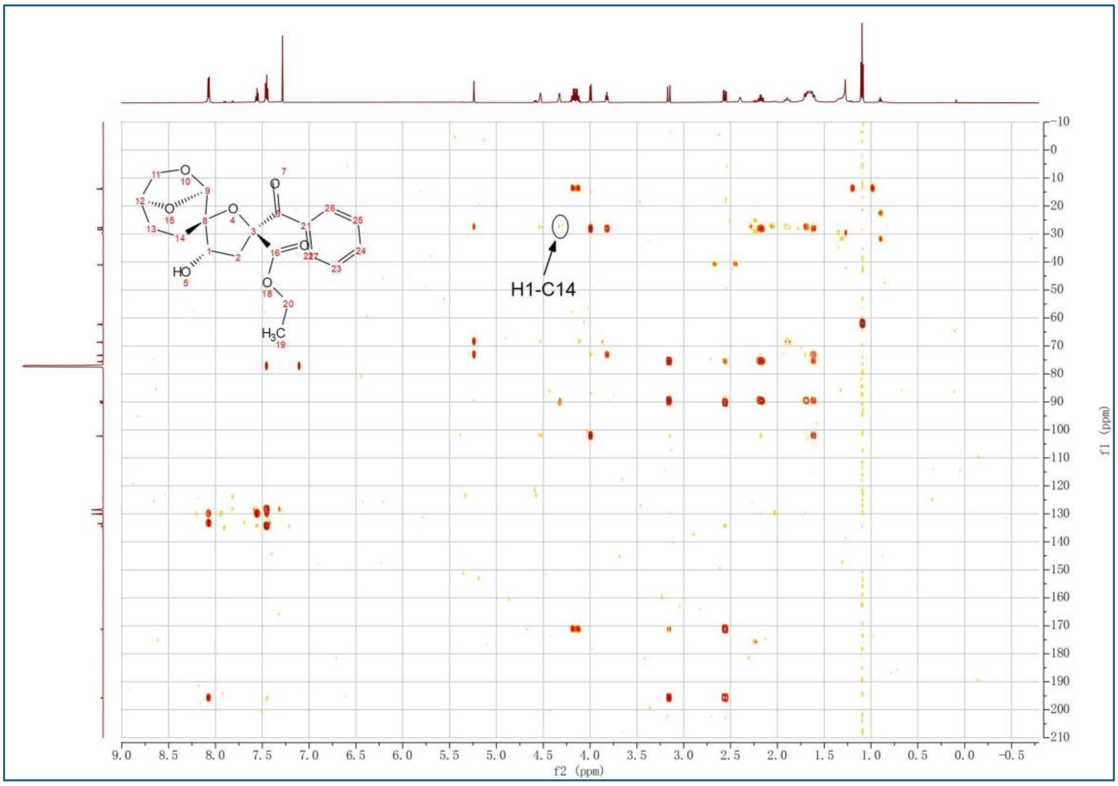

HMBC of **32**

ethyl (2R,3a'R,4'S,7'S,7a'R)-5-benzoyl-3-hydroxydecahydro-3H-spiro[furan-2,5'-[4,7]methanoindene]-5-carboxylate (33)

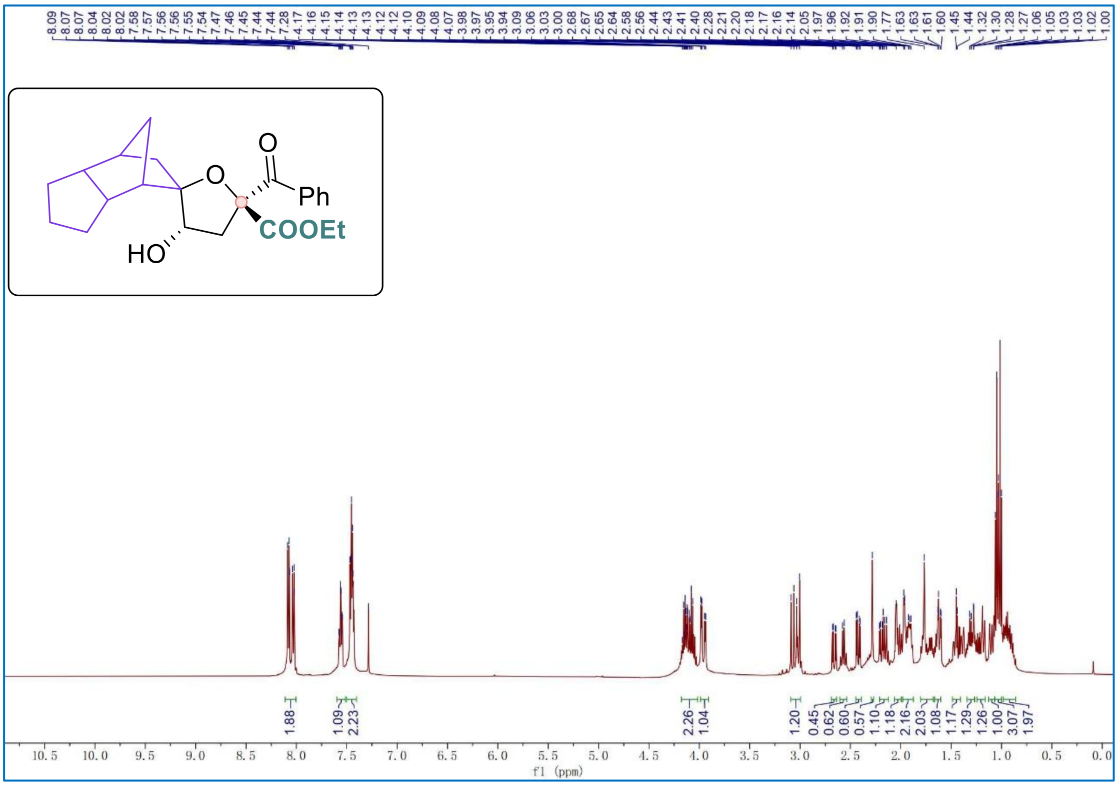

^{1}H NMR-spectrum (500 MHz, Chloroform-*d*) of **33**

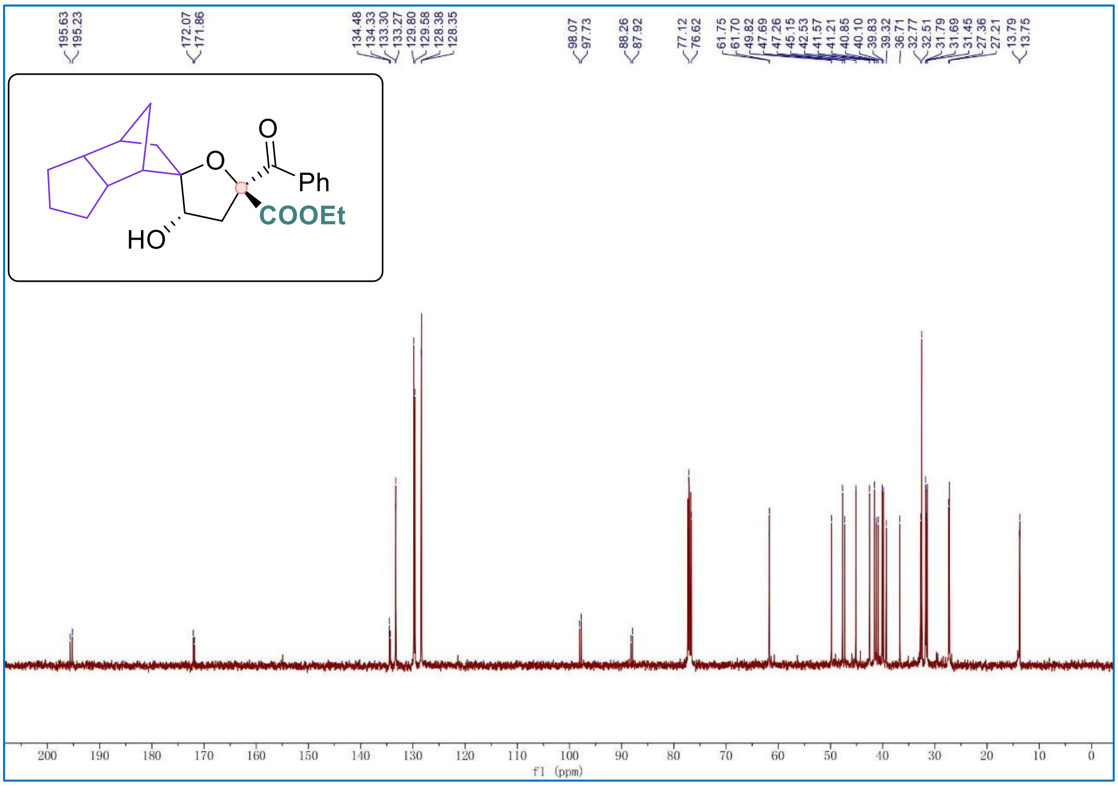

^{13}C NMR-spectrum (126 MHz, Chloroform-*d*) of **33**

SUPPORTING INFORMATION

ethyl (1R,3S,5r,7r)-5'-benzoyl-3'-hydroxydihydro-3'H-spiro[adamantane-2,2'-furan]-5'-carboxylate (34)

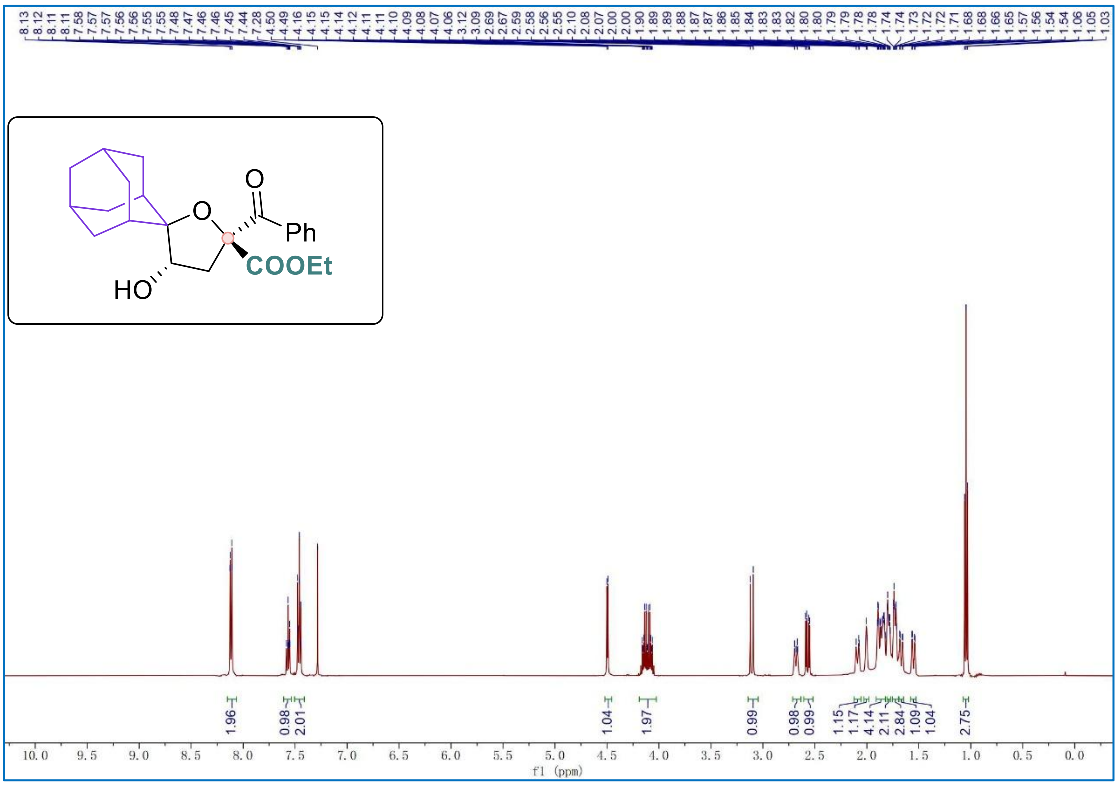

^{1}H NMR-spectrum (500 MHz, Chloroform-*d*) of **34**

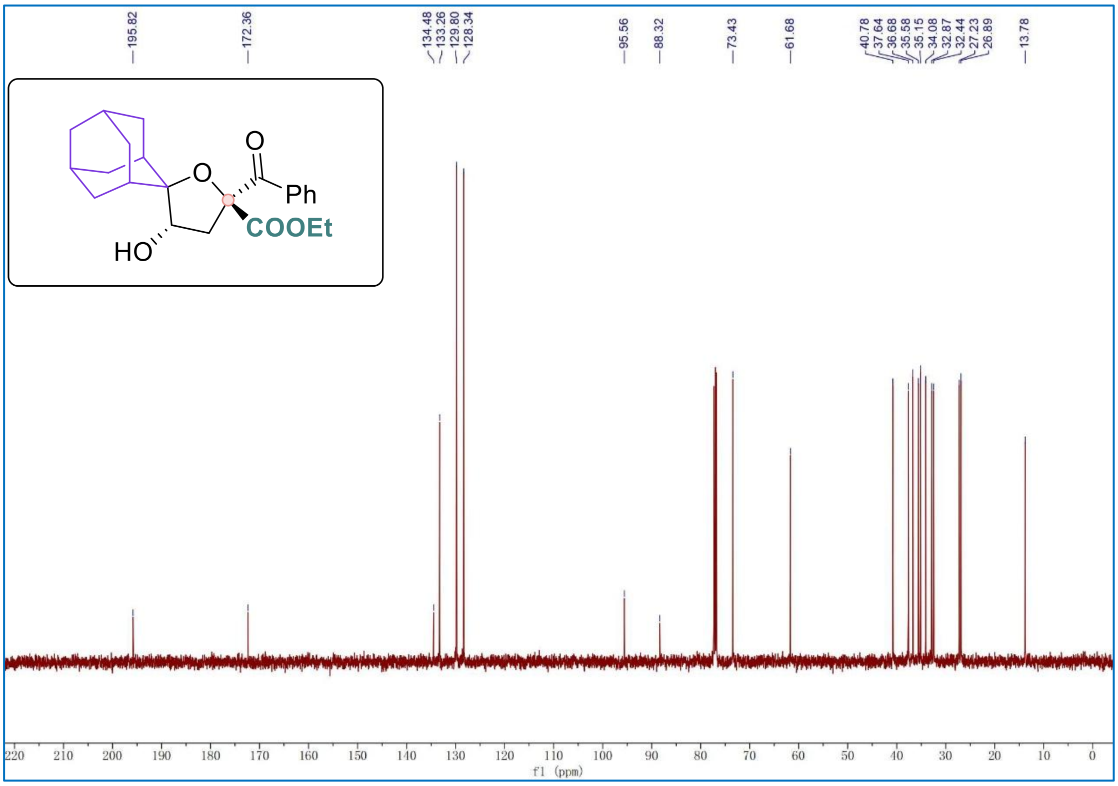

^{13}C NMR-spectrum (126 MHz, Chloroform-*d*) of **34**

ethyl 5'-benzoyl-3'-hydroxydihydro-3'H-spiro[bicyclo[2.2.1]heptane-2,2'-furan]-5'-carboxylate (35)

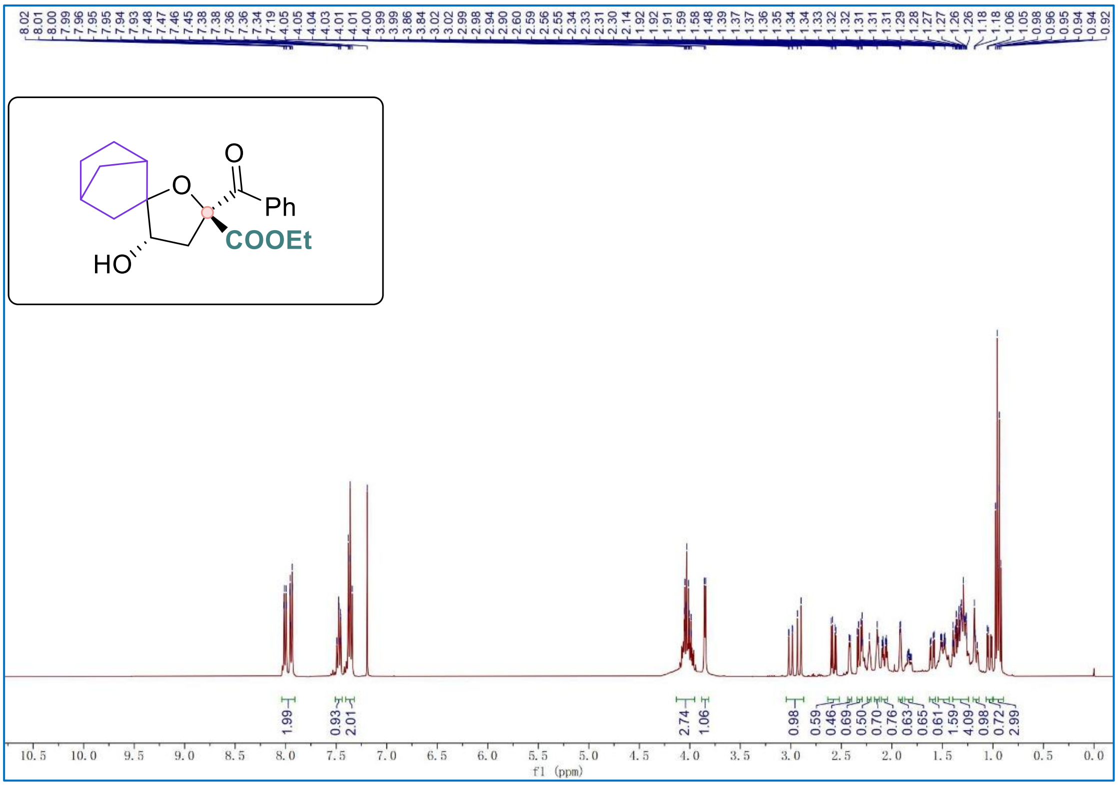

^{1}H NMR-spectrum (400 MHz, Chloroform-*d*) of **35**

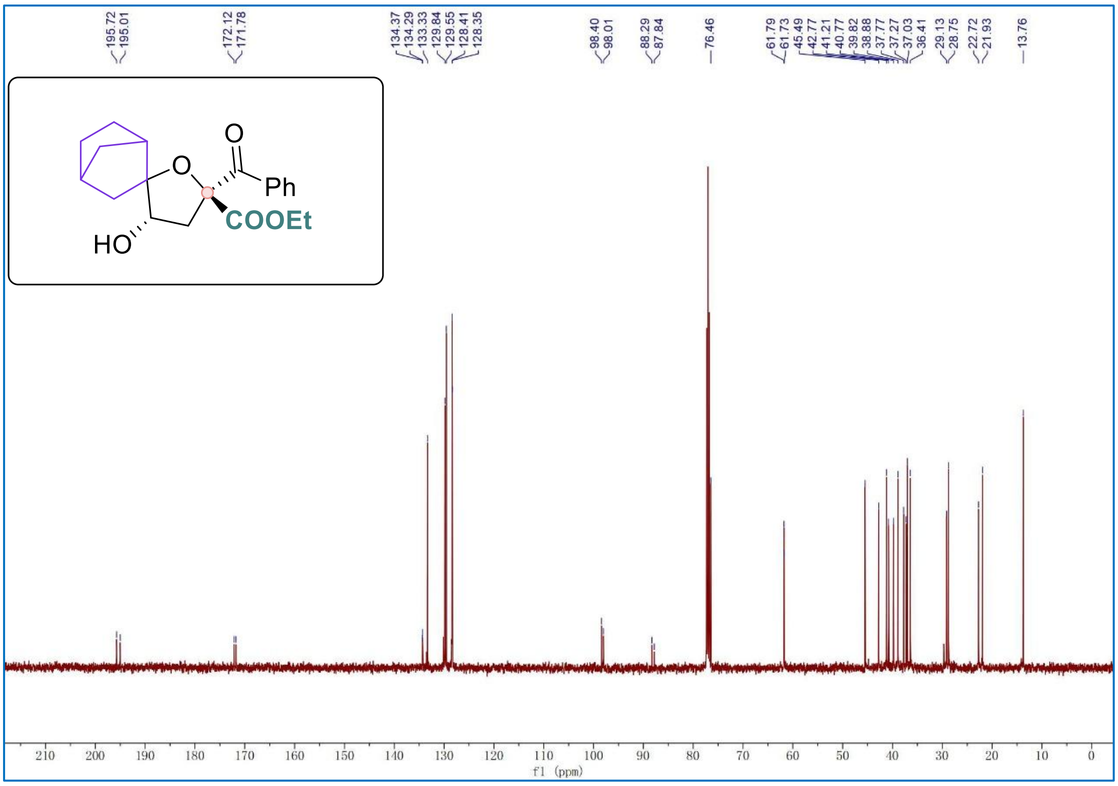

^{13}C NMR-spectrum (126 MHz, Chloroform-*d*) of **35**

ethyl (5S,6R,9S)-2-benzoyl-4-hydroxy-6-isopropyl-9-methyl-1-oxaspiro[4.5]decane-2-carboxylate (36)

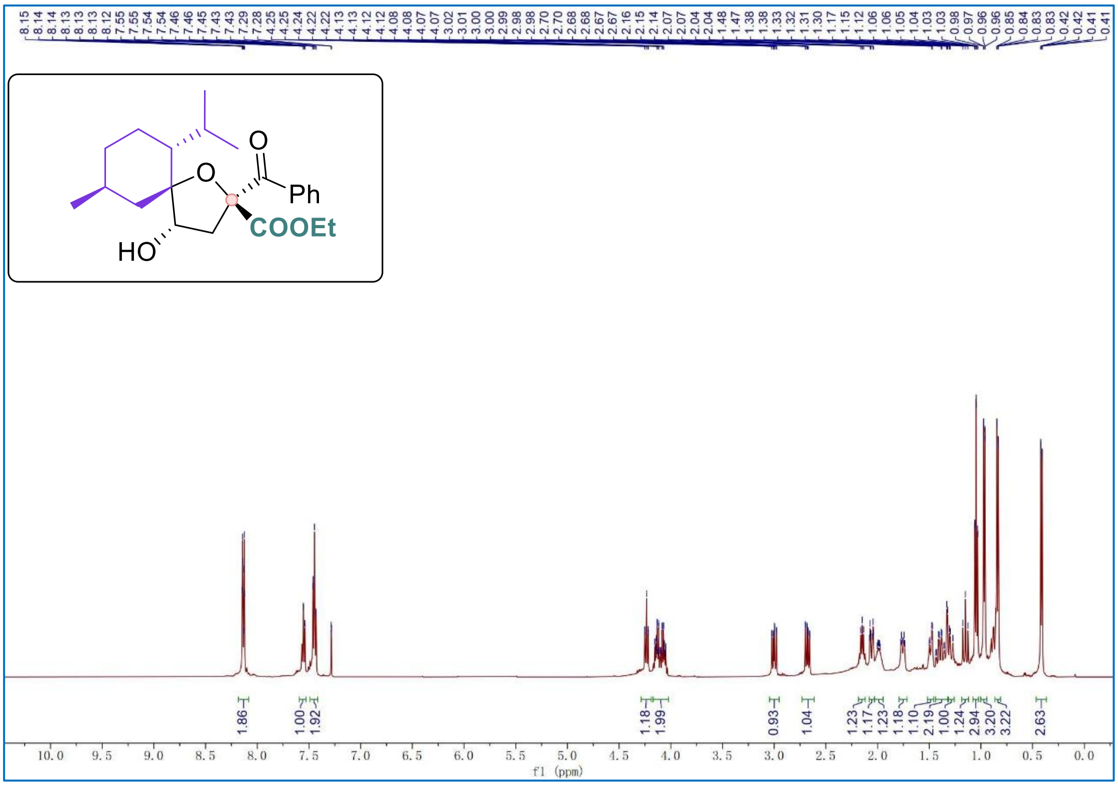

^{1}H NMR-spectrum (500 MHz, Chloroform-*d*) of **36**

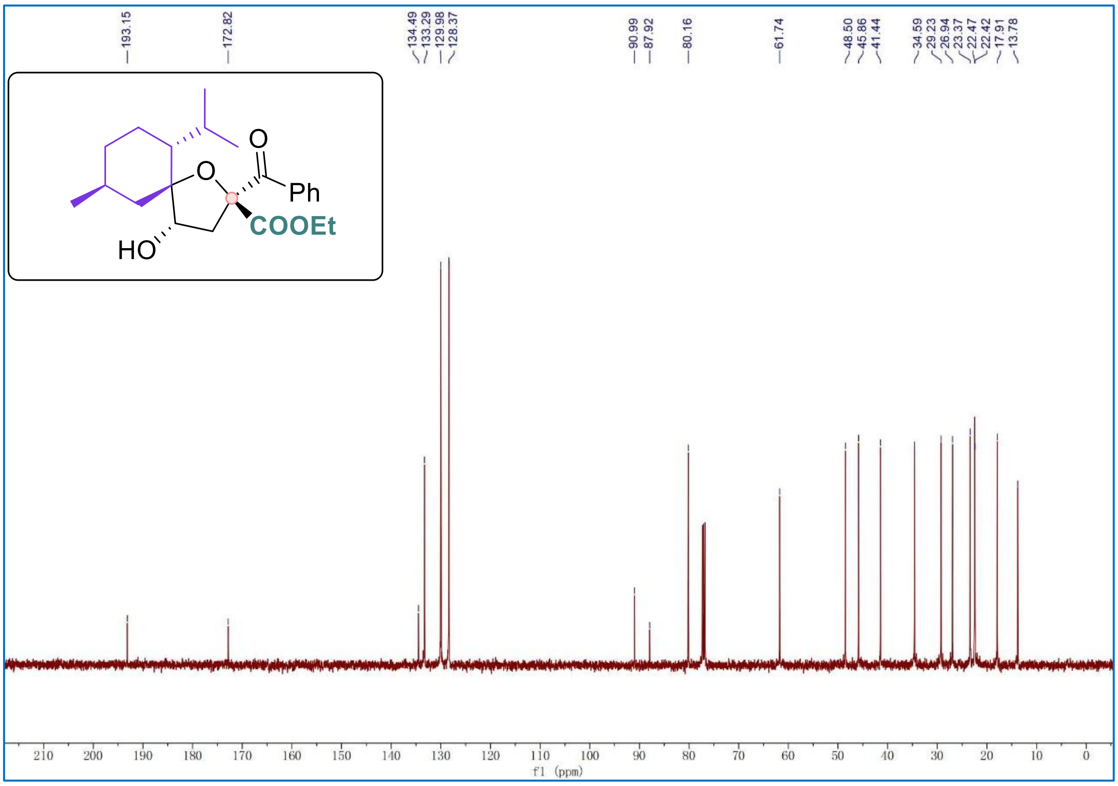

^{13}C NMR-spectrum (126 MHz, Chloroform-*d*) of **36**

SUPPORTING INFORMATION

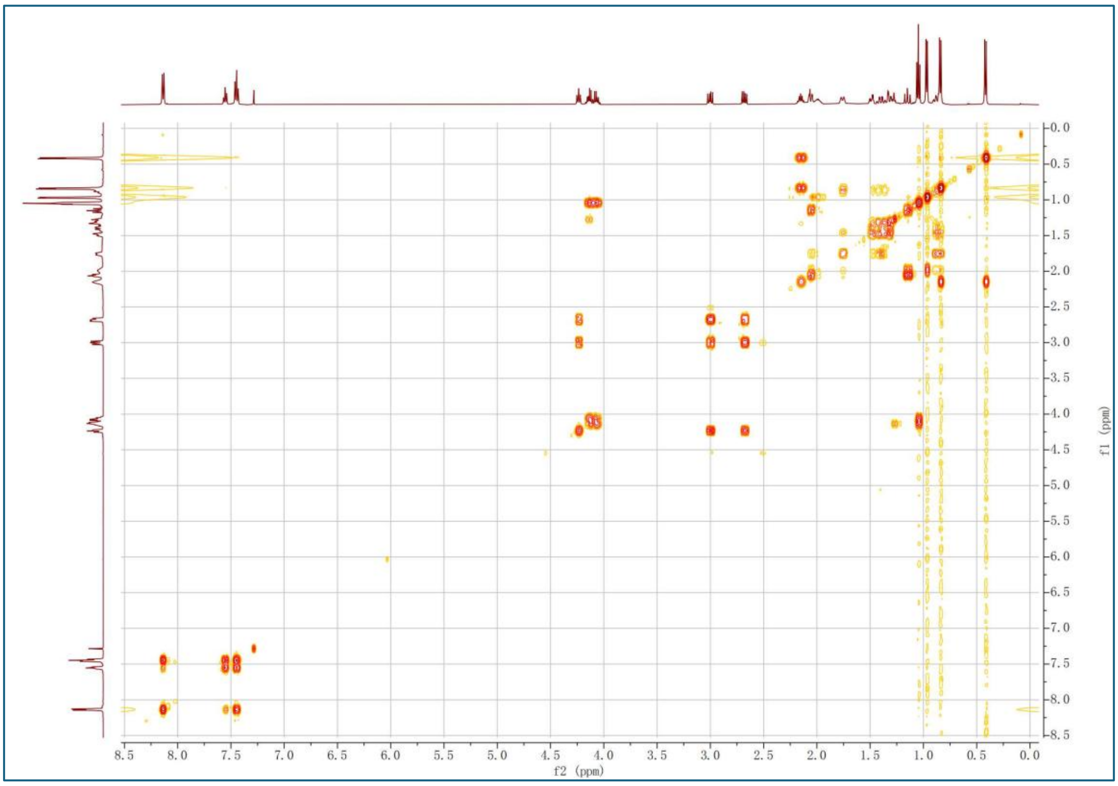

COESY of **36**

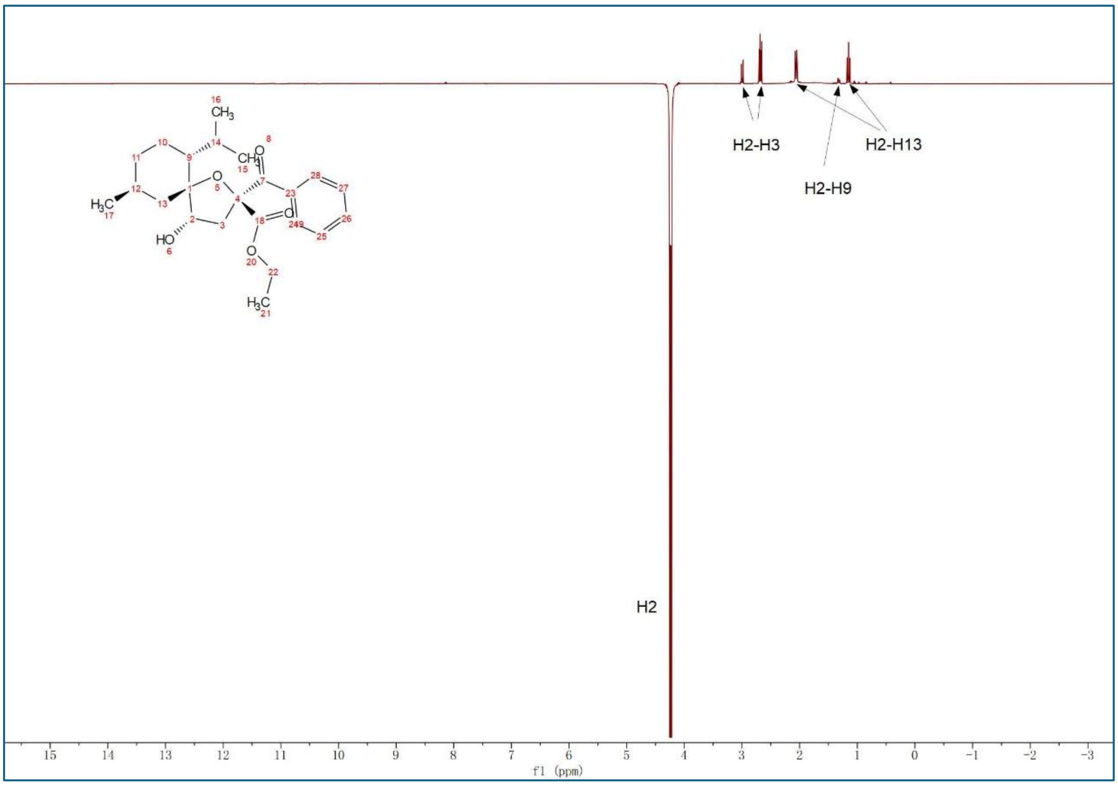

1D NOESY experiment with selective excitation of H2

ethyl 4-hydroxy-2-(4-iodobenzoyl)-1-oxaspiro[4.5]decane-2-carboxylate (37)

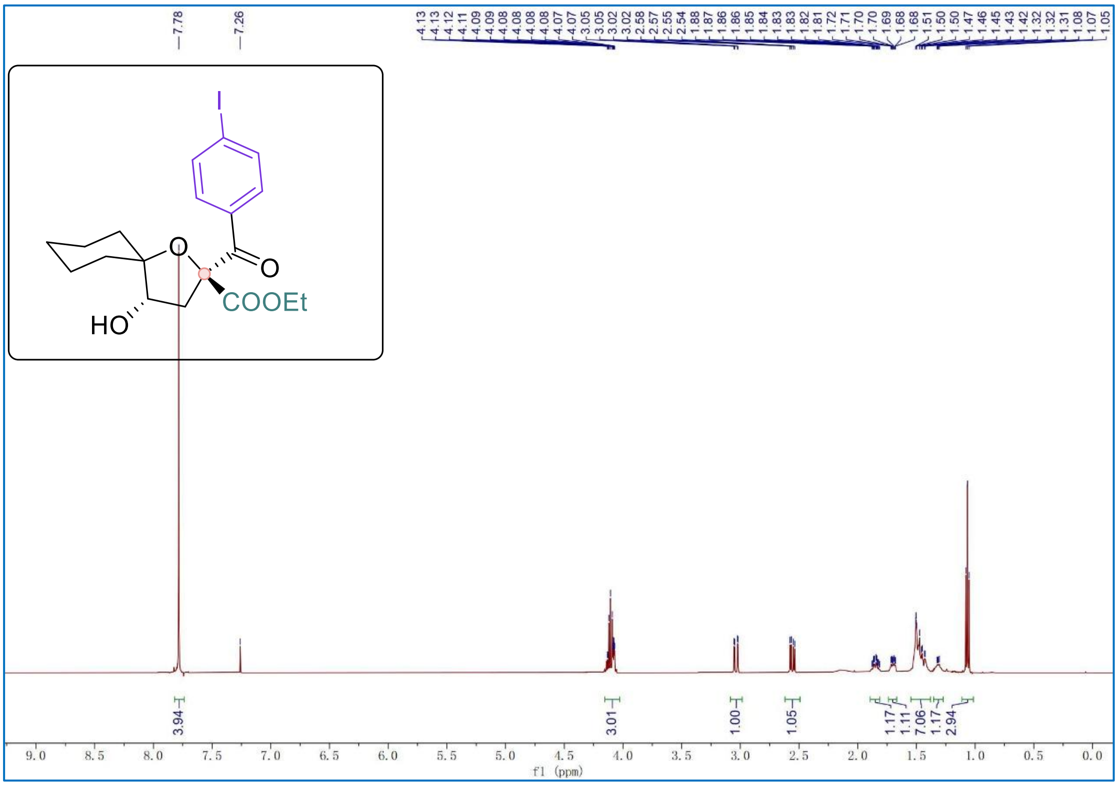

^{1}H NMR-spectrum (500 MHz, Chloroform-*d*) of **37**

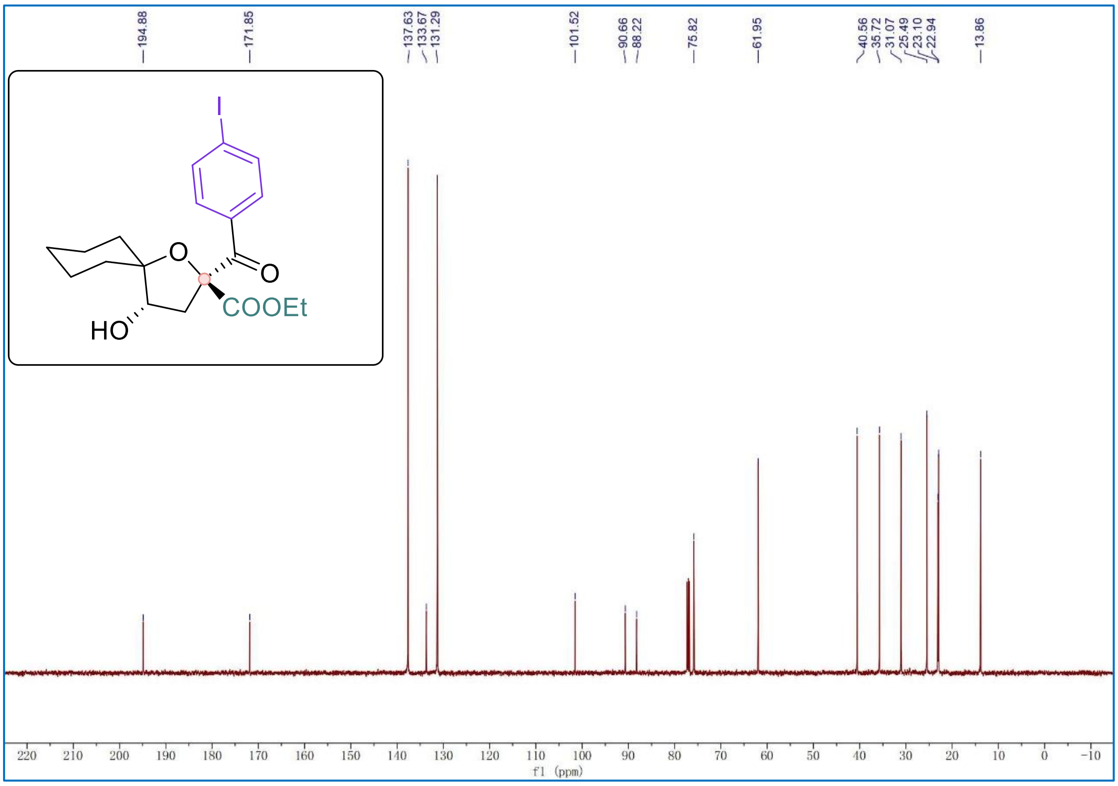

^{13}C NMR-spectrum (126 MHz, Chloroform-*d*) of **37**

SUPPORTING INFORMATION

ethyl 4-hydroxy-2-(4-(trifluoromethoxy)benzoyl)-1-oxaspiro[4.5]decane-2-carboxylate (38)

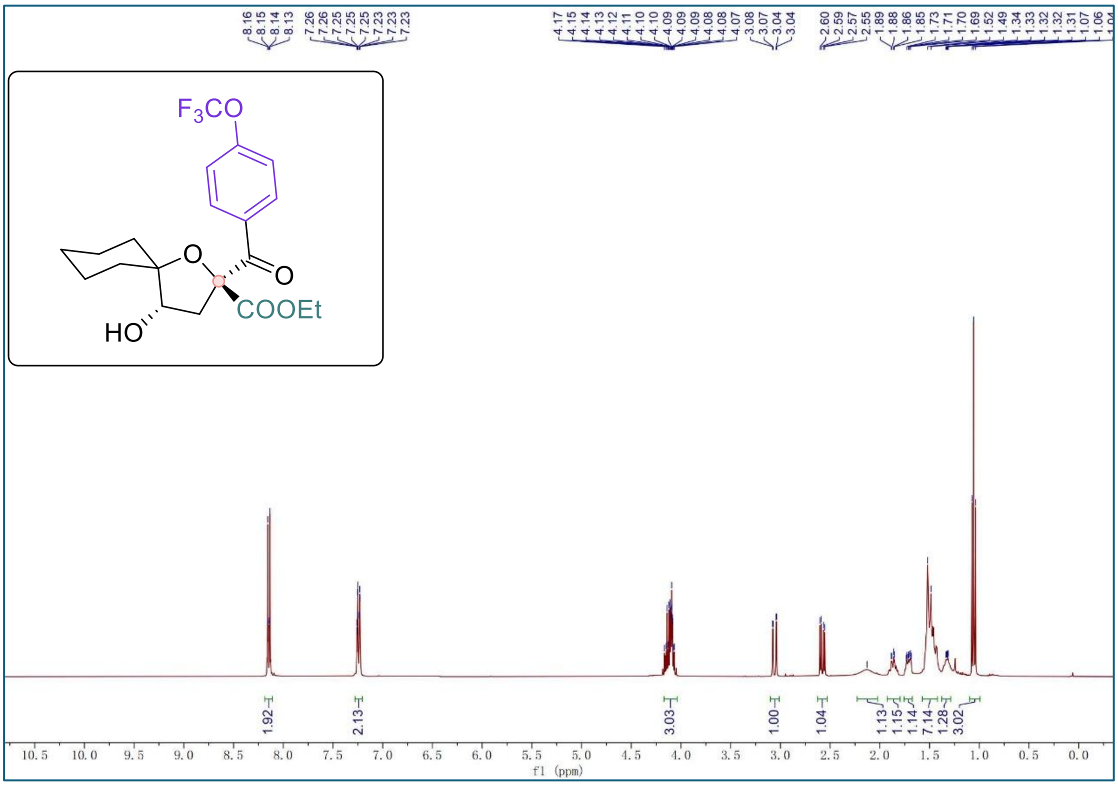

^{1}H NMR-spectrum (400 MHz, Chloroform-*d*) of **38**

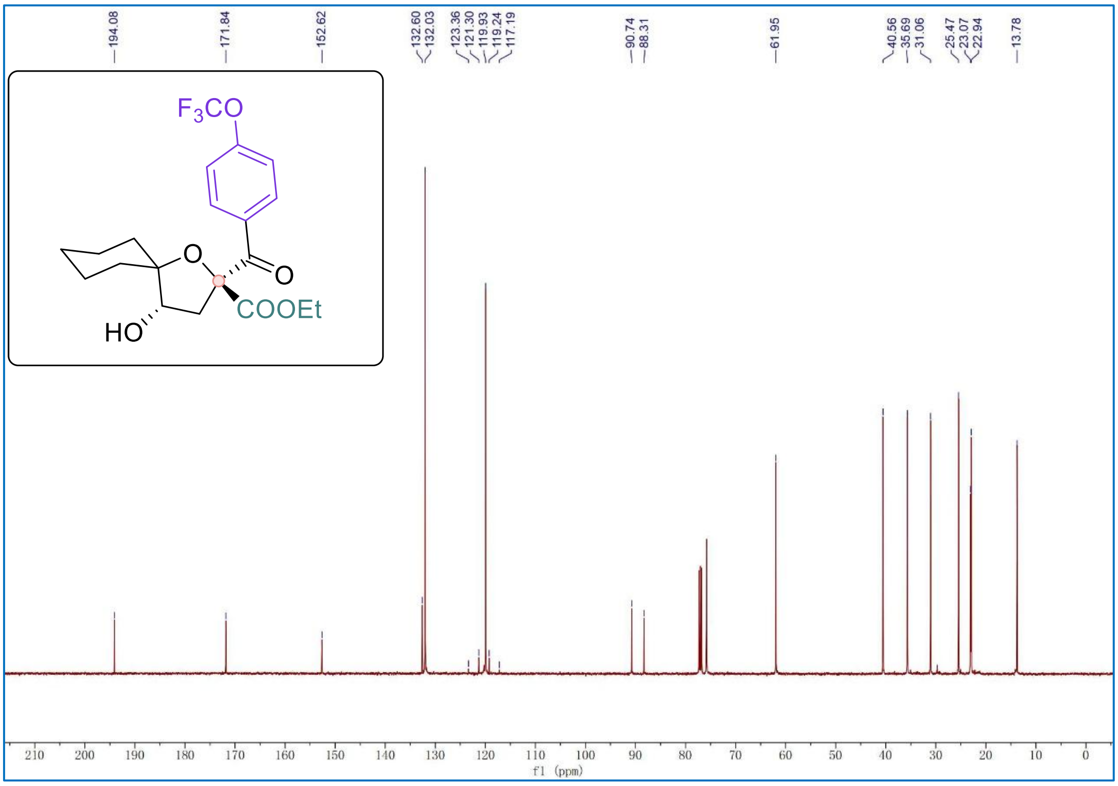

^{13}C NMR-spectrum (126 MHz, Chloroform-*d*) of **38**

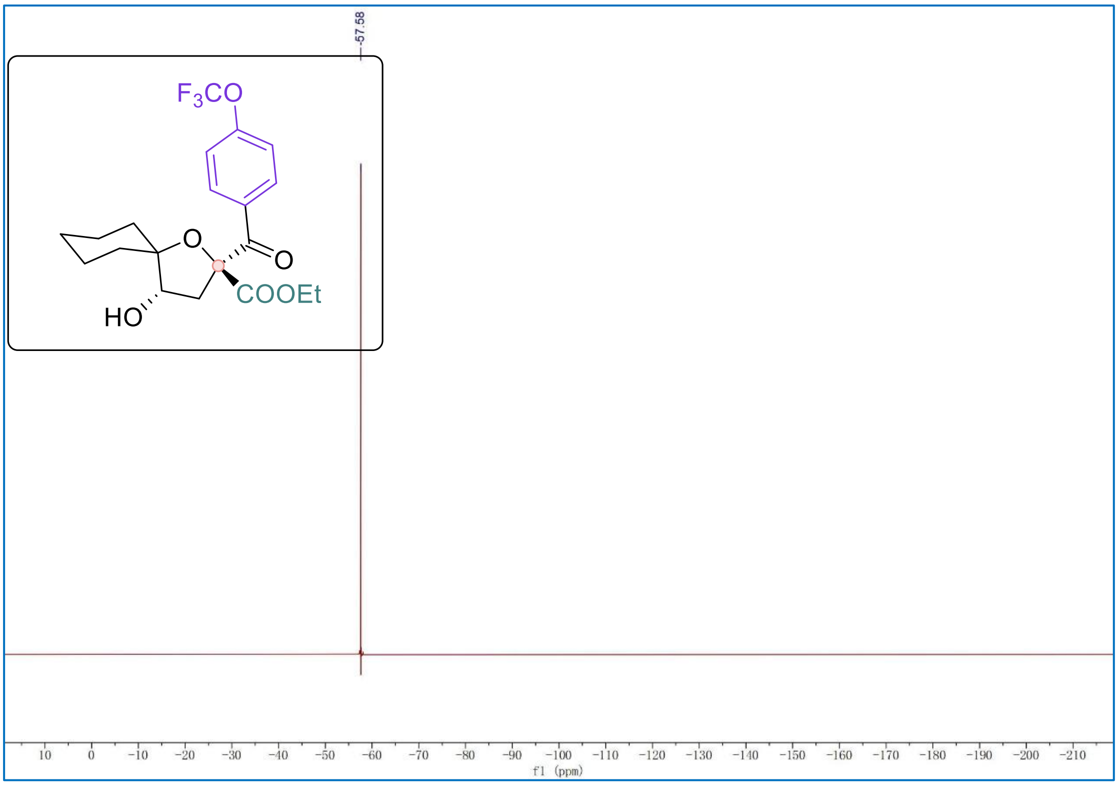

^{19}F NMR-spectrum (377 MHz, Chloroform-*d*) of **38**

ethyl 4-hydroxy-2-(4-(methylsulfonyl)benzoyl)-1-oxaspiro[4.5]decane-2-carboxylate (39)

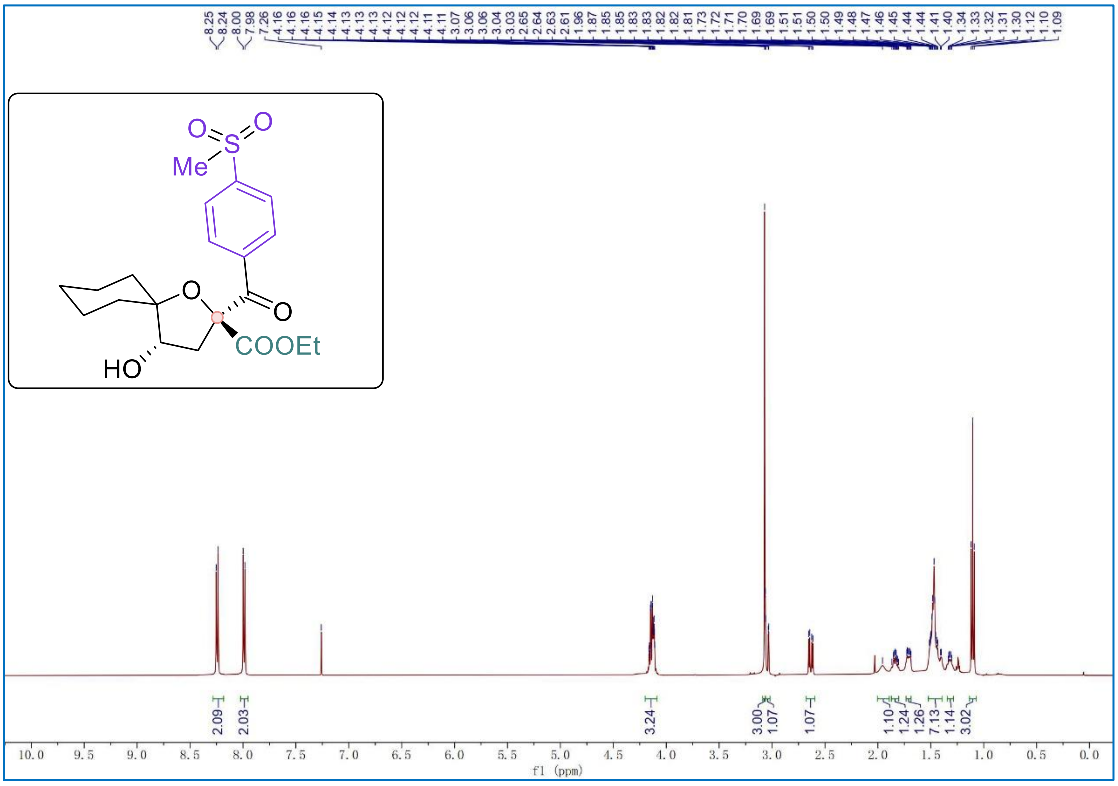

^{1}H NMR-spectrum (500 MHz, Chloroform-*d*) of **39**

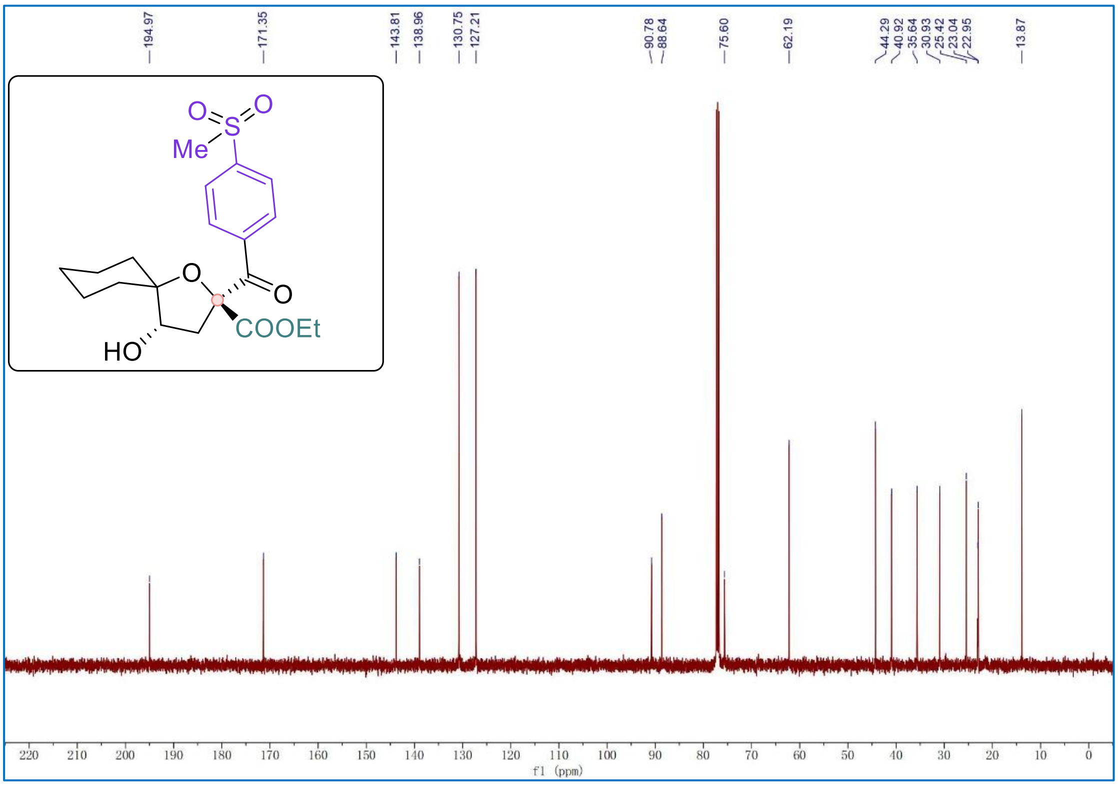

^{13}C NMR-spectrum (126 MHz, Chloroform-*d*) of **39**

SUPPORTING INFORMATION

ethyl 4-hydroxy-2-(4-(methoxycarbonyl)benzoyl)-5,5-dimethyltetrahydrofuran-2-carboxylate (40)

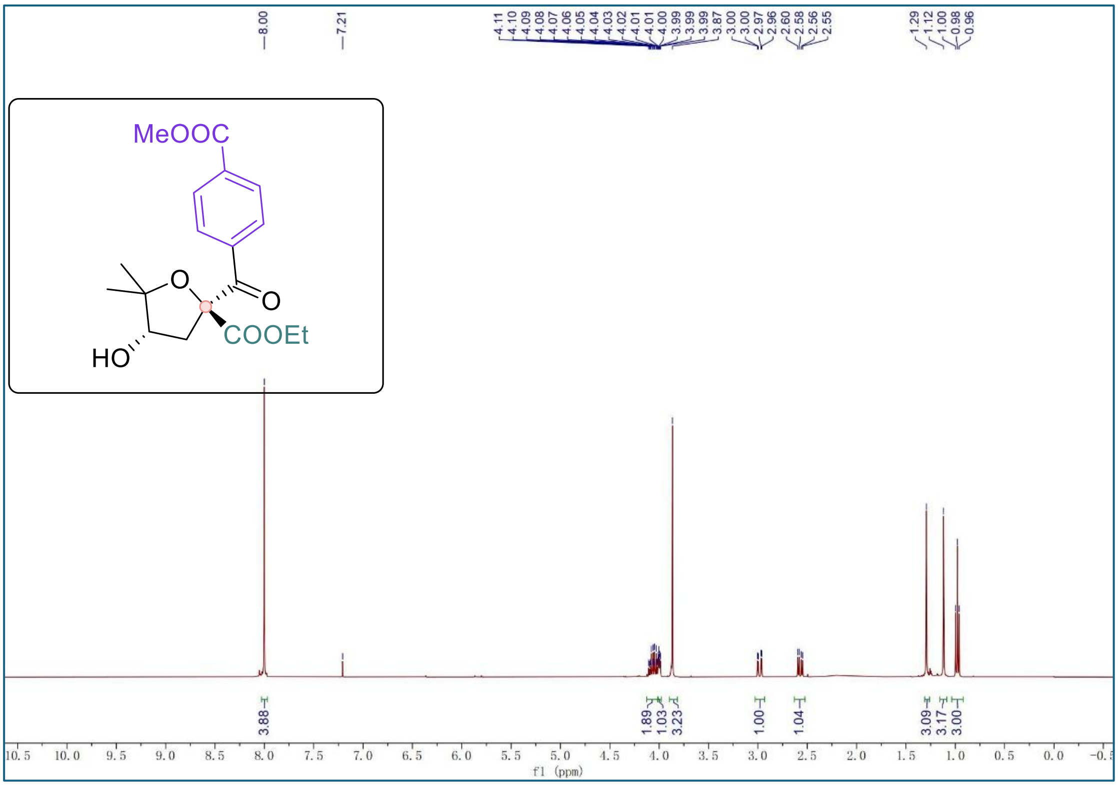

^{1}H NMR-spectrum (400 MHz, Chloroform-*d*) of **40**

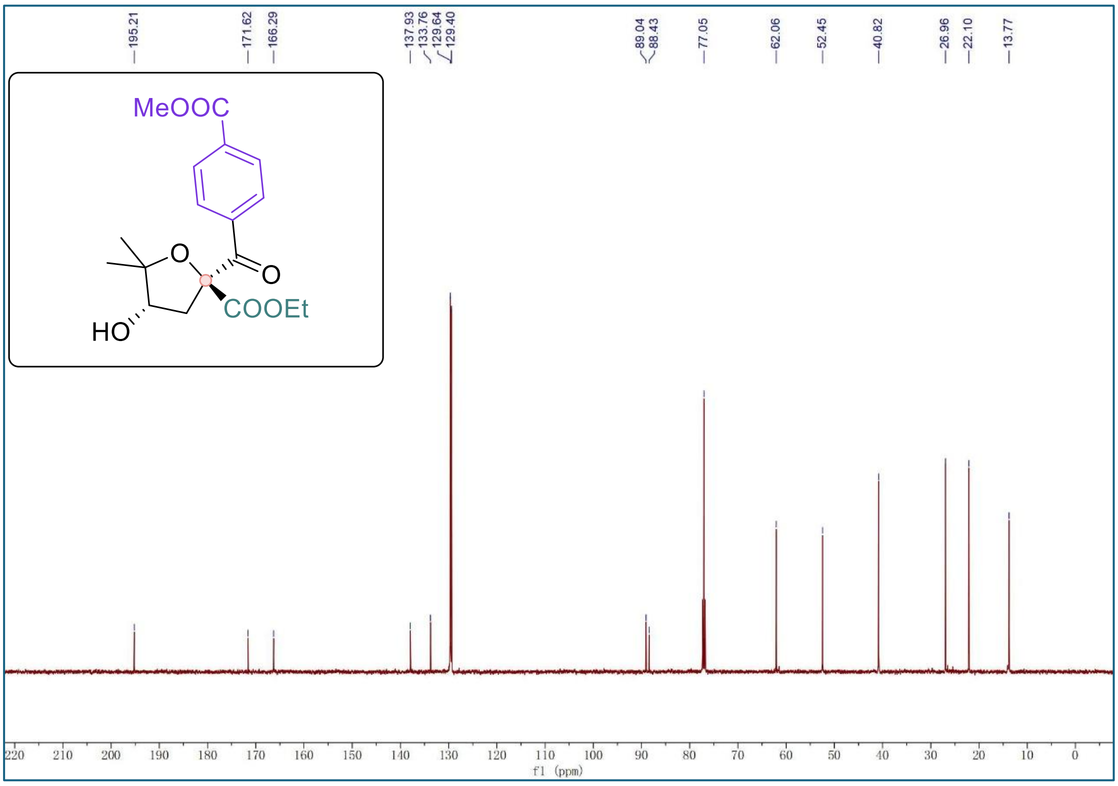

^{13}C NMR-spectrum (126 MHz, Chloroform-*d*) of **40**

ethyl 2-(4-cyanobenzoyl)-4-hydroxy-5,5-dimethyltetrahydrofuran-2-carboxylate (41)

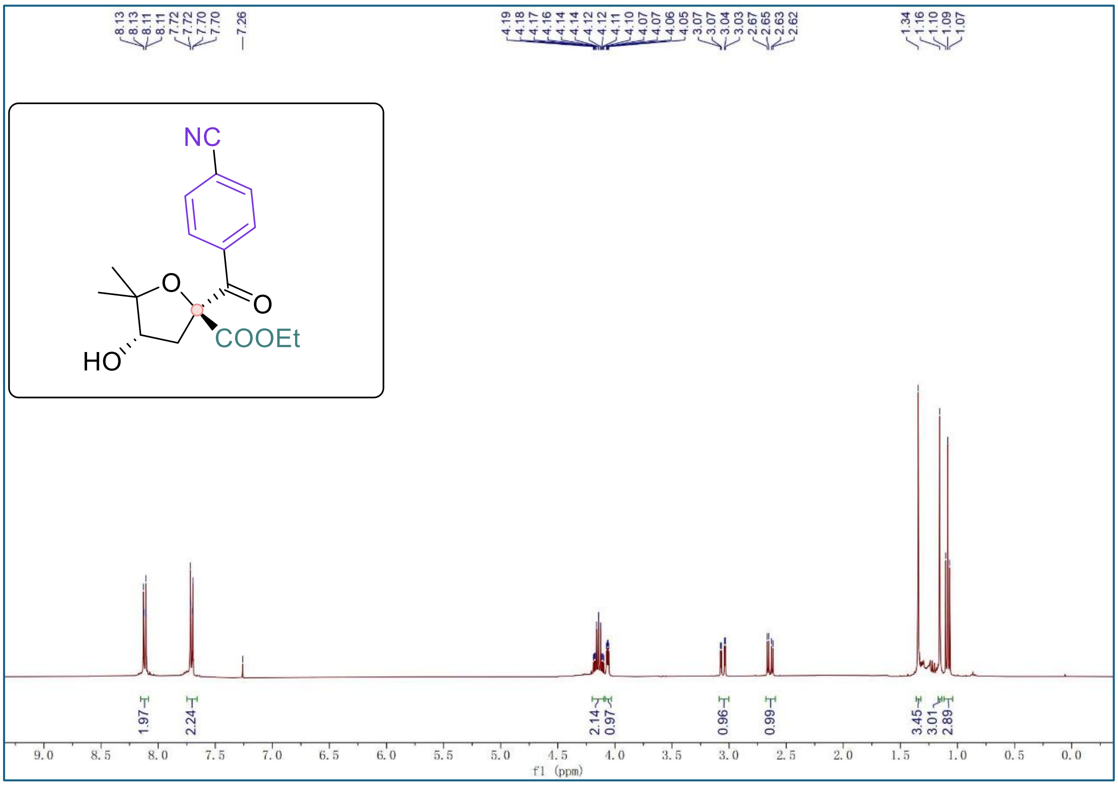

^{1}H NMR-spectrum (400 MHz, Chloroform-*d*) of **41**

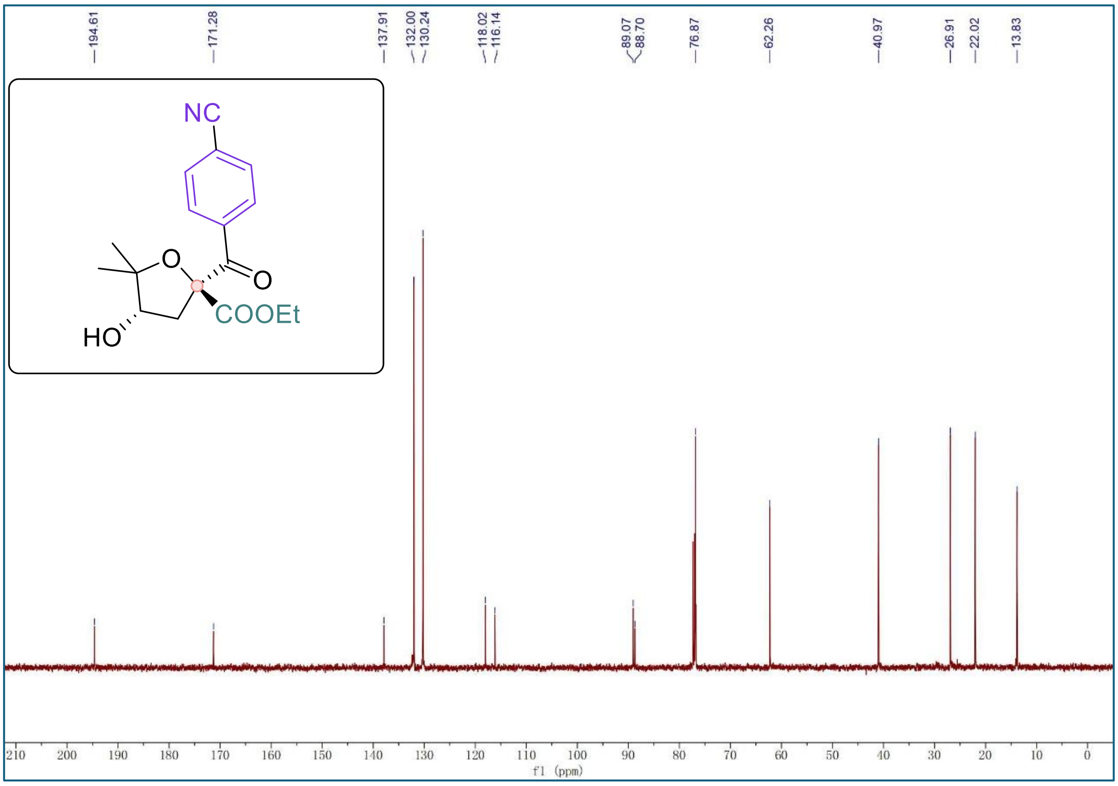

^{13}C NMR-spectrum (126 MHz, Chloroform-*d*) of **41**

ethyl 4-hydroxy-5,5-dimethyl-2-(4-nitrobenzoyl)tetrahydrofuran-2-carboxylate (42)

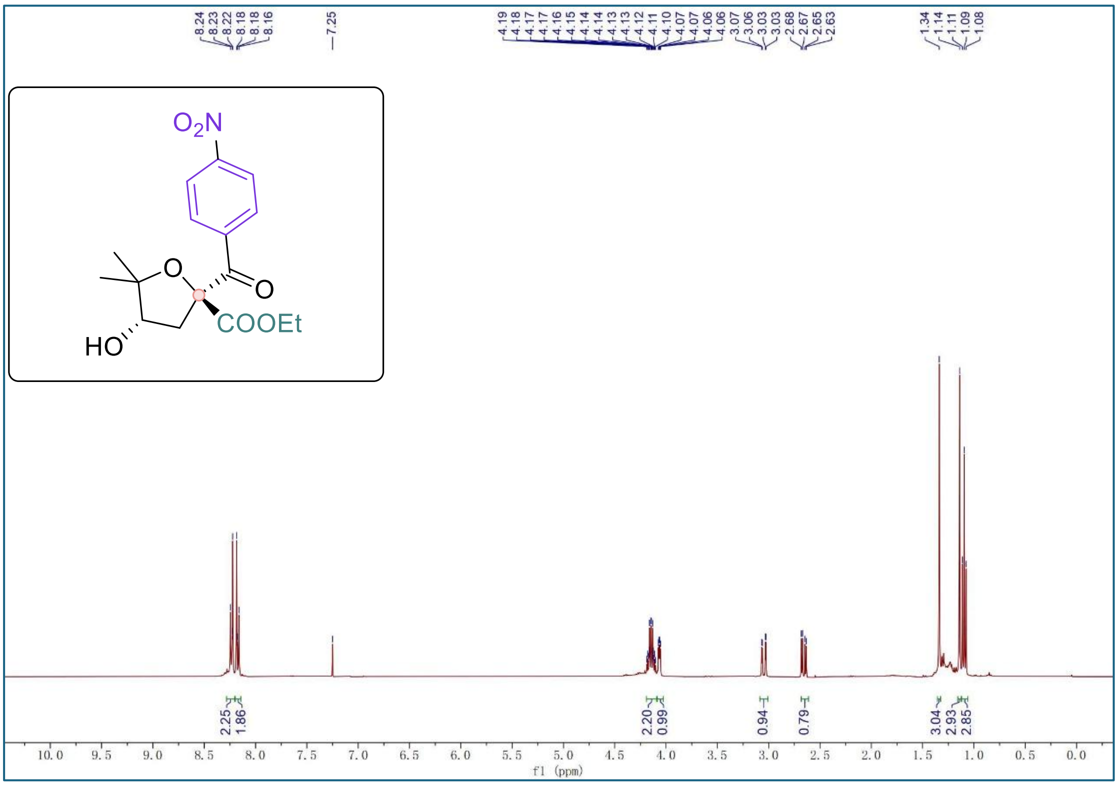

^{1}H NMR-spectrum (400 MHz, Chloroform-*d*) of **42**

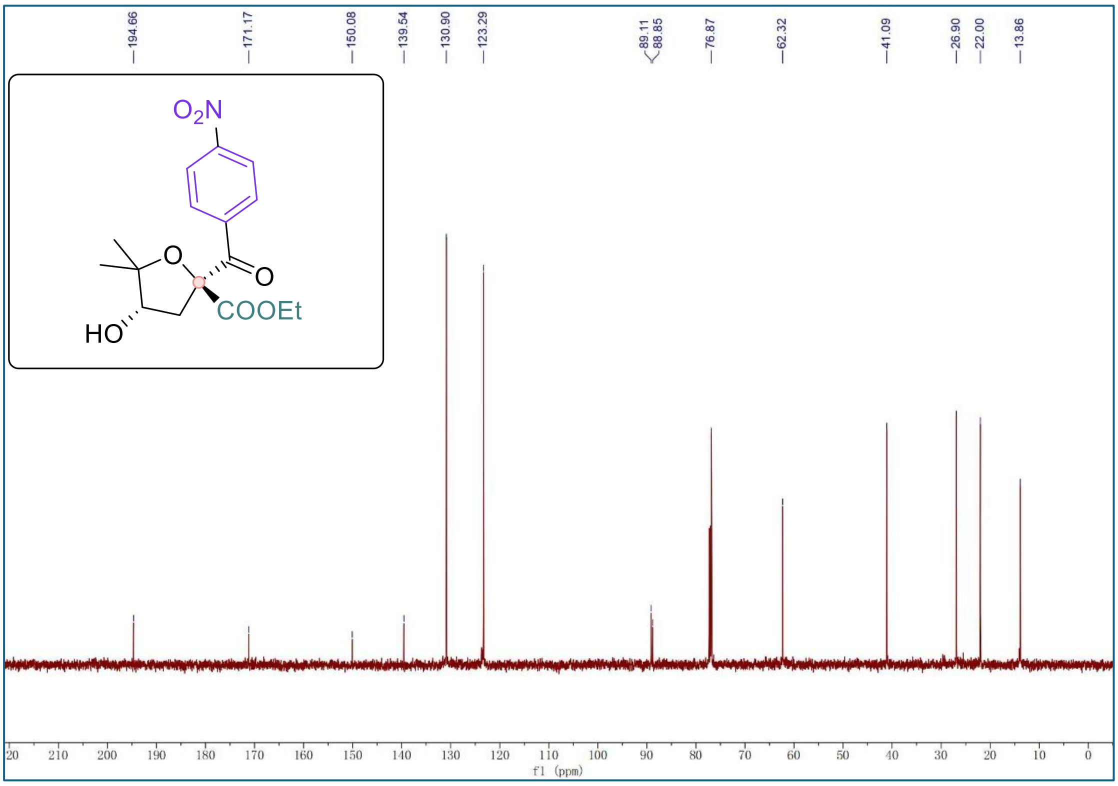

^{13}C NMR-spectrum (126 MHz, Chloroform-*d*) of **42**

ethyl 4-hydroxy-2-(4-iodobenzoyl)-5,5-dimethyltetrahydrofuran-2-carboxylate (43)

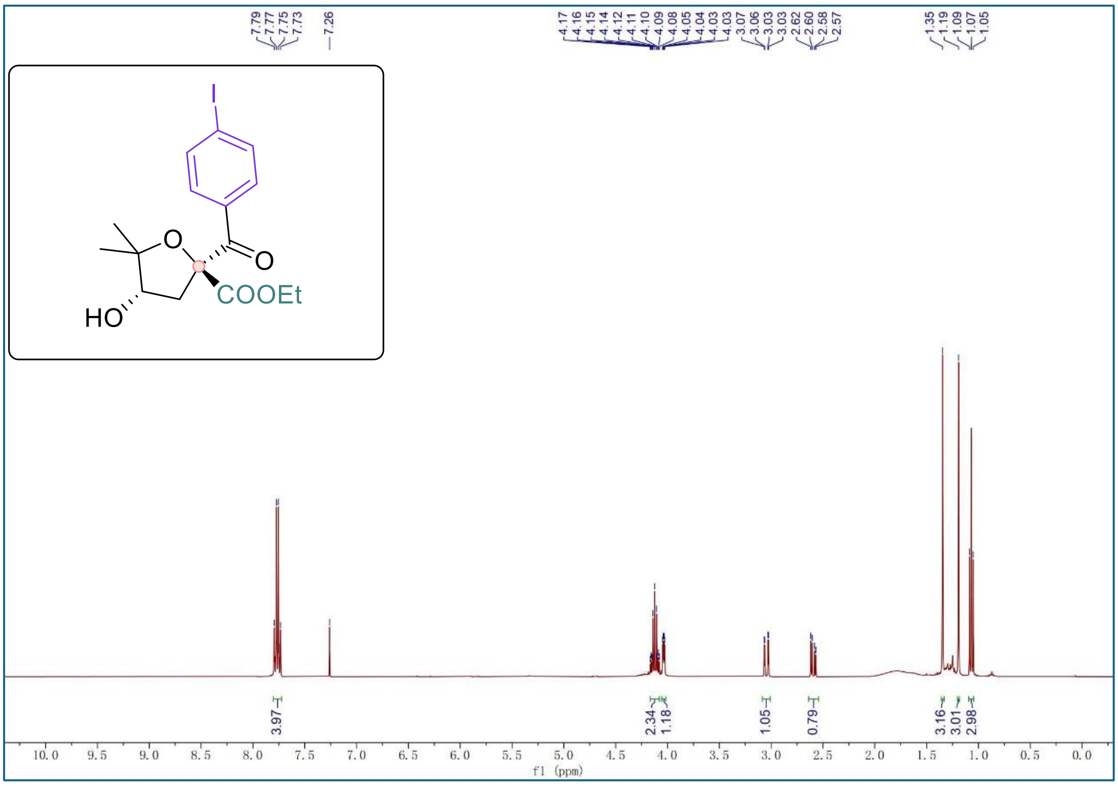

^{1}H NMR-spectrum (400 MHz, Chloroform-*d*) of **43**

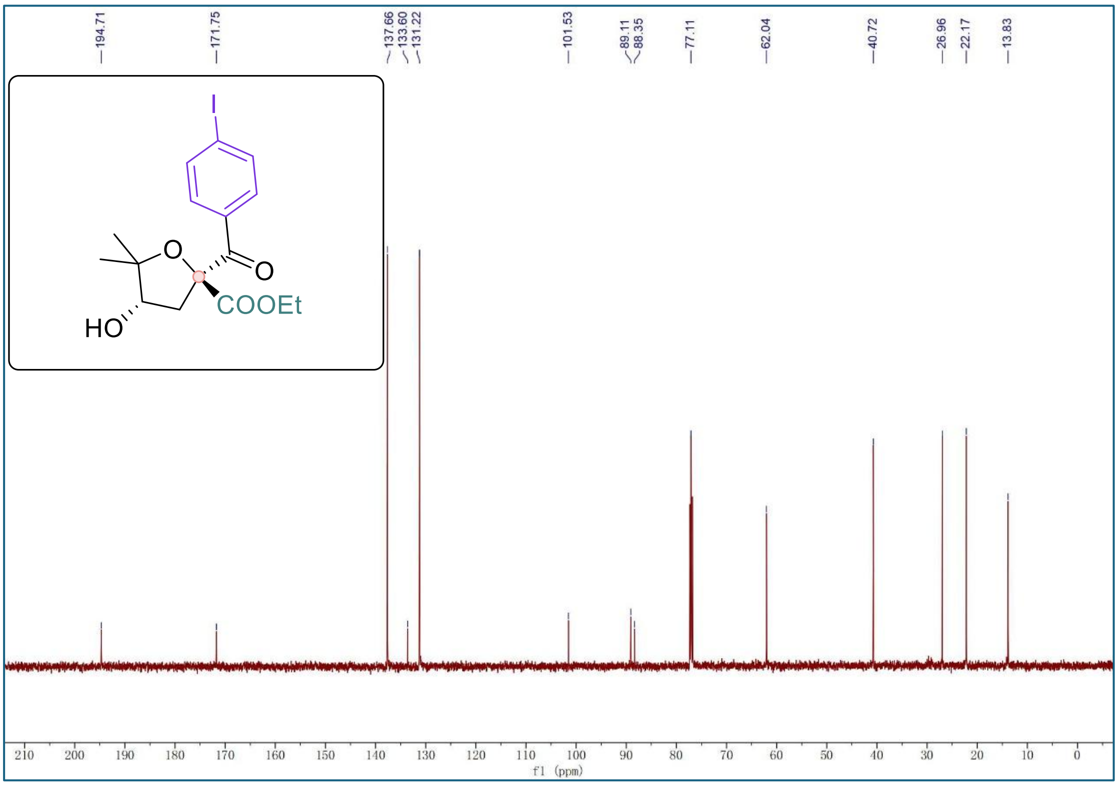

^{13}C NMR-spectrum (126 MHz, Chloroform-*d*) of **43**

ethyl 2-(4-(chloromethyl)benzoyl)-4-hydroxy-5,5-dimethyltetrahydrofuran-2-carboxylate (44)

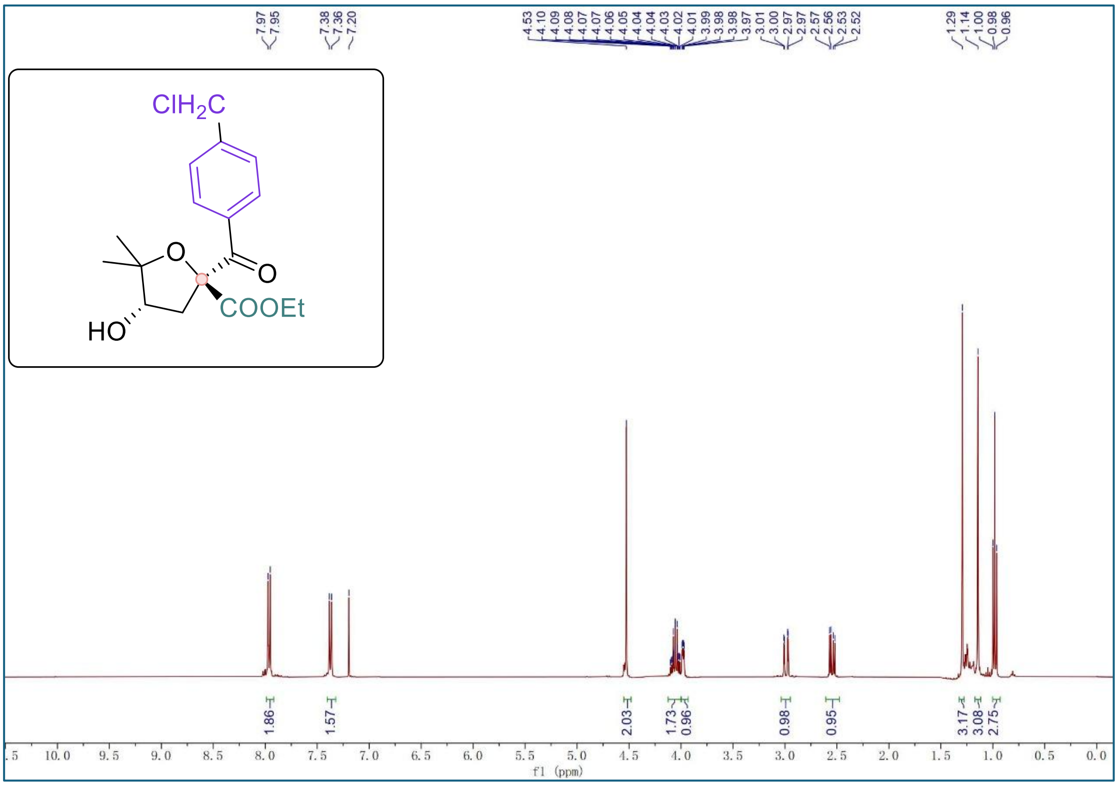

^{1}H NMR-spectrum (400 MHz, Chloroform-*d*) of **44**

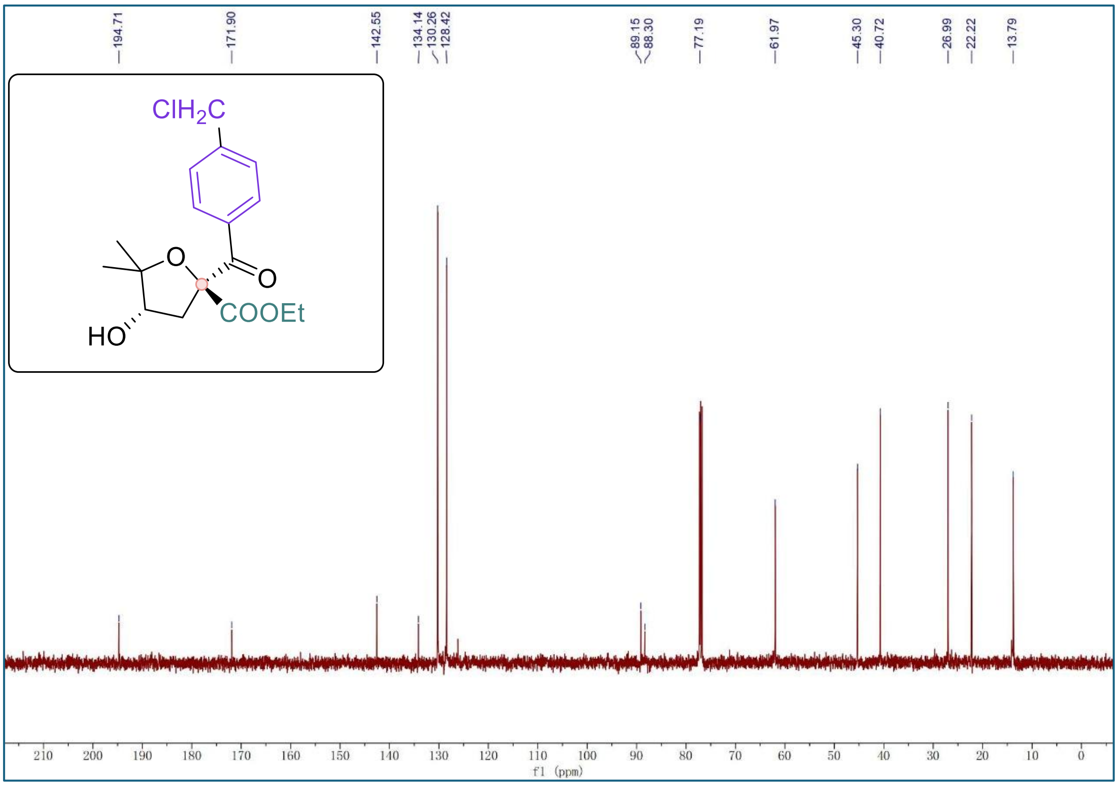

^{13}C NMR-spectrum (126 MHz, Chloroform-*d*) of **44**

ethyl 4-hydroxy-5,5-dimethyl-2-(4-(trifluoromethoxy)benzoyl)tetrahydrofuran-2-carboxylate (45)

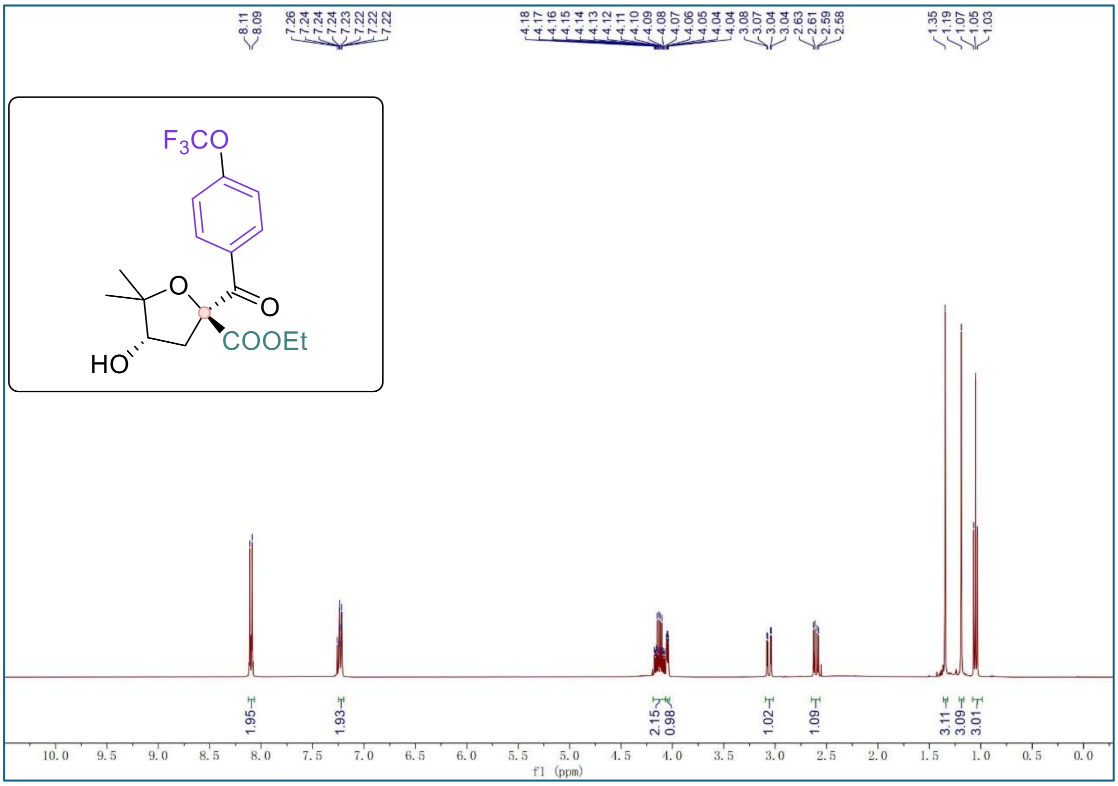

^{1}H NMR-spectrum (400 MHz, Chloroform-*d*) of **45**

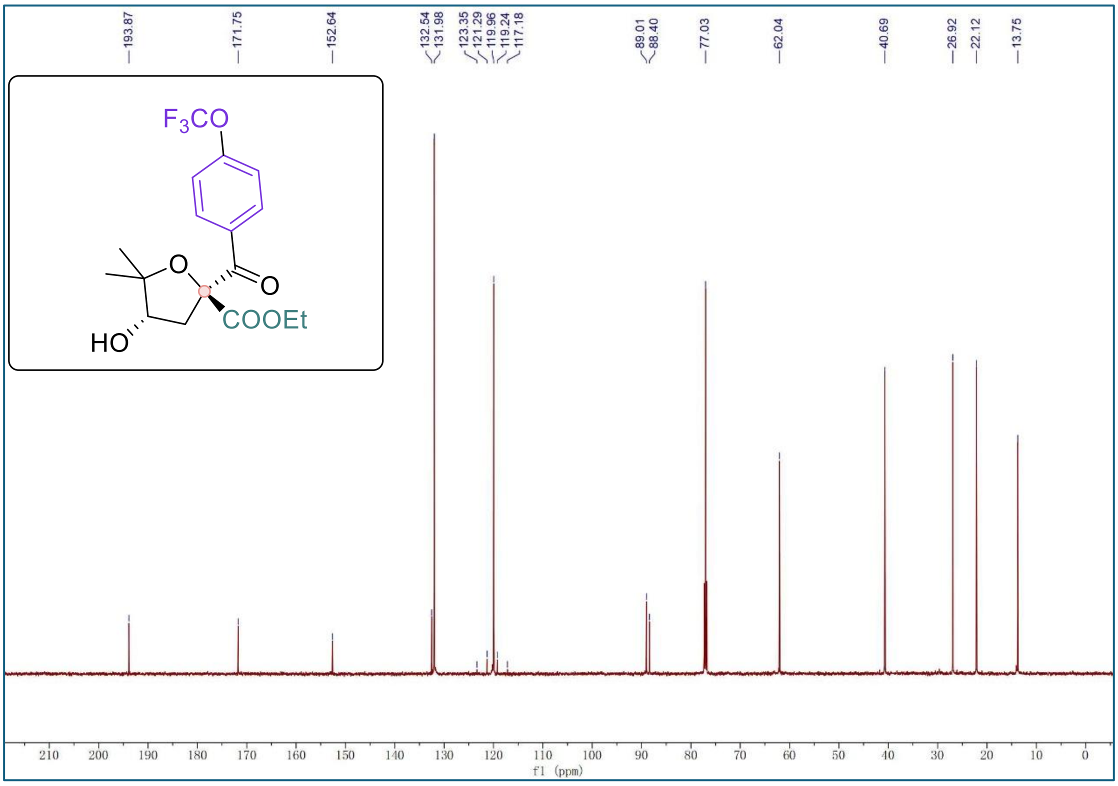

^{13}C NMR-spectrum (126 MHz, Chloroform-*d*) of **45**

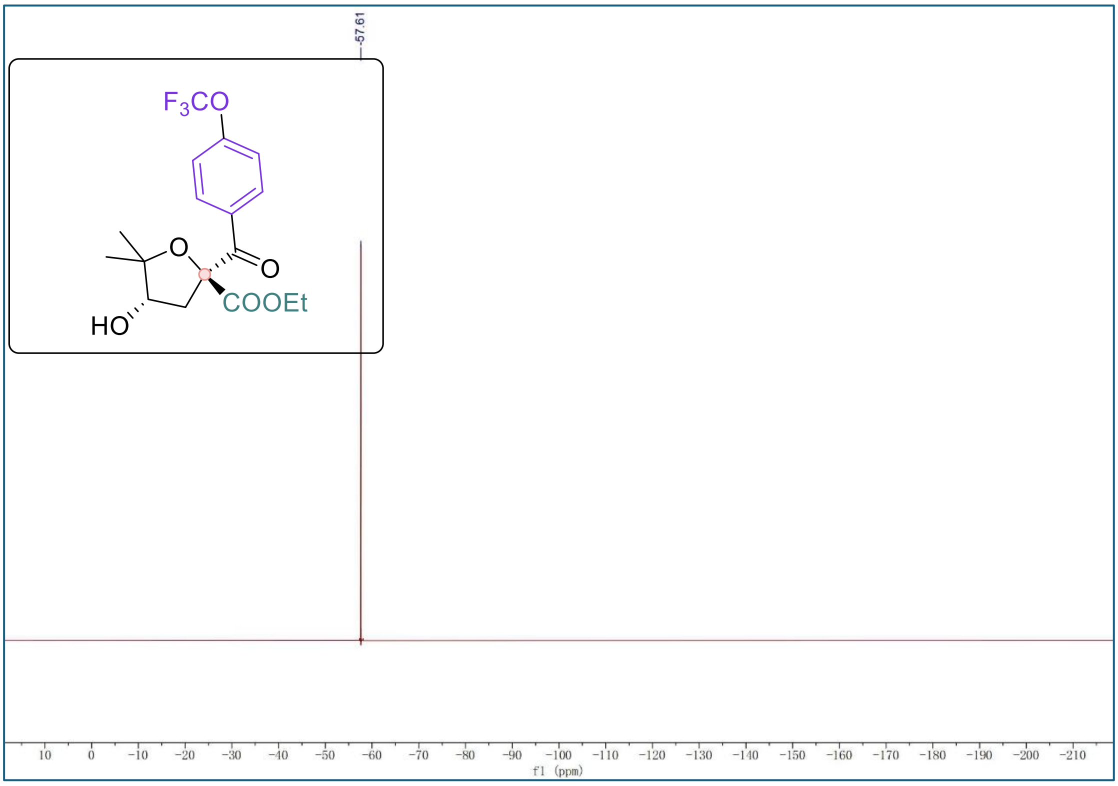

^{19}F NMR-spectrum (377 MHz, Chloroform-*d*) of **45**

ethyl 2-(4-ethoxybenzoyl)-4-hydroxy-5,5-dimethyltetrahydrofuran-2-carboxylate (46)

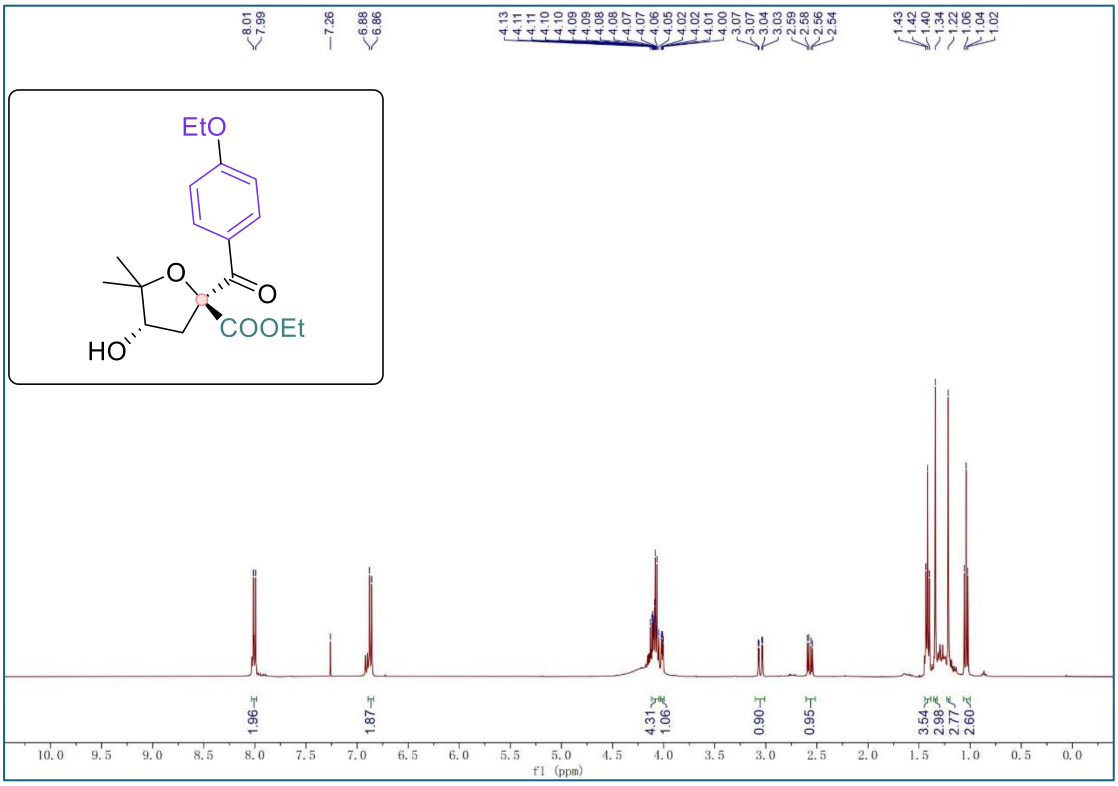

^{1}H NMR-spectrum (400 MHz, Chloroform-*d*) of **46**

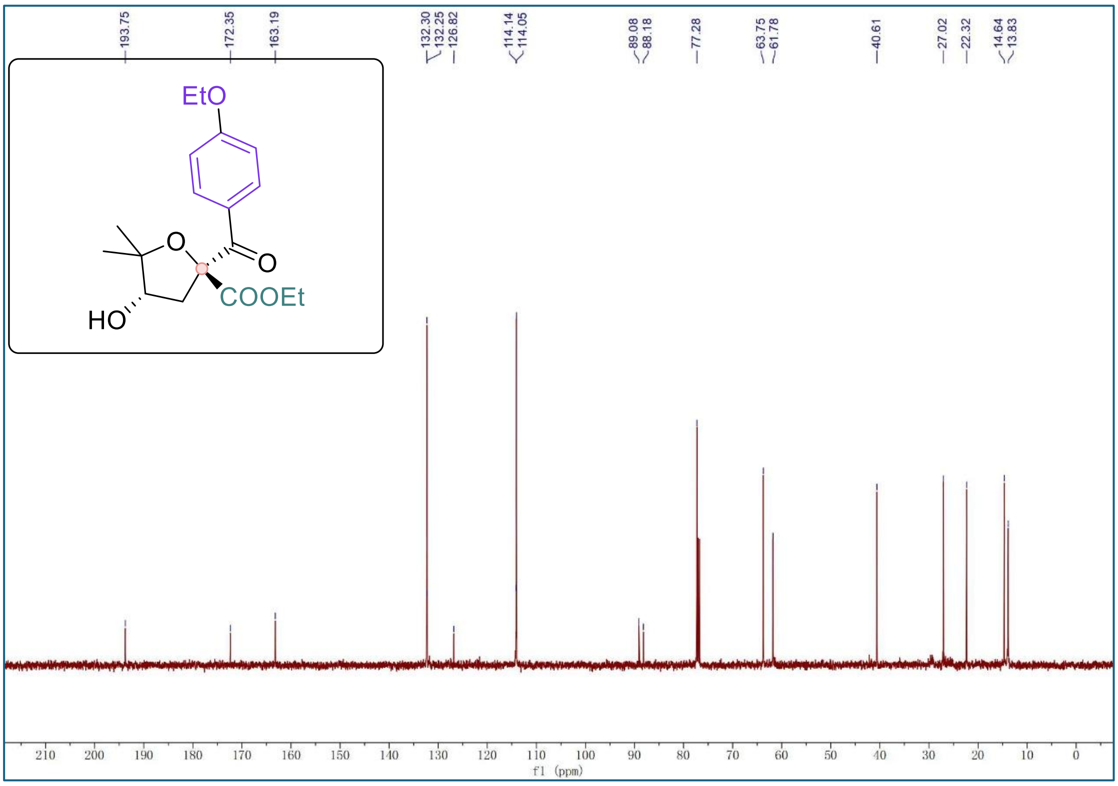

^{13}C NMR-spectrum (126 MHz, Chloroform-*d*) of **46**

ethyl 4-hydroxy-2-(2-(methoxycarbonyl)benzoyl)-5,5-dimethyltetrahydrofuran-2-carboxylate (47)

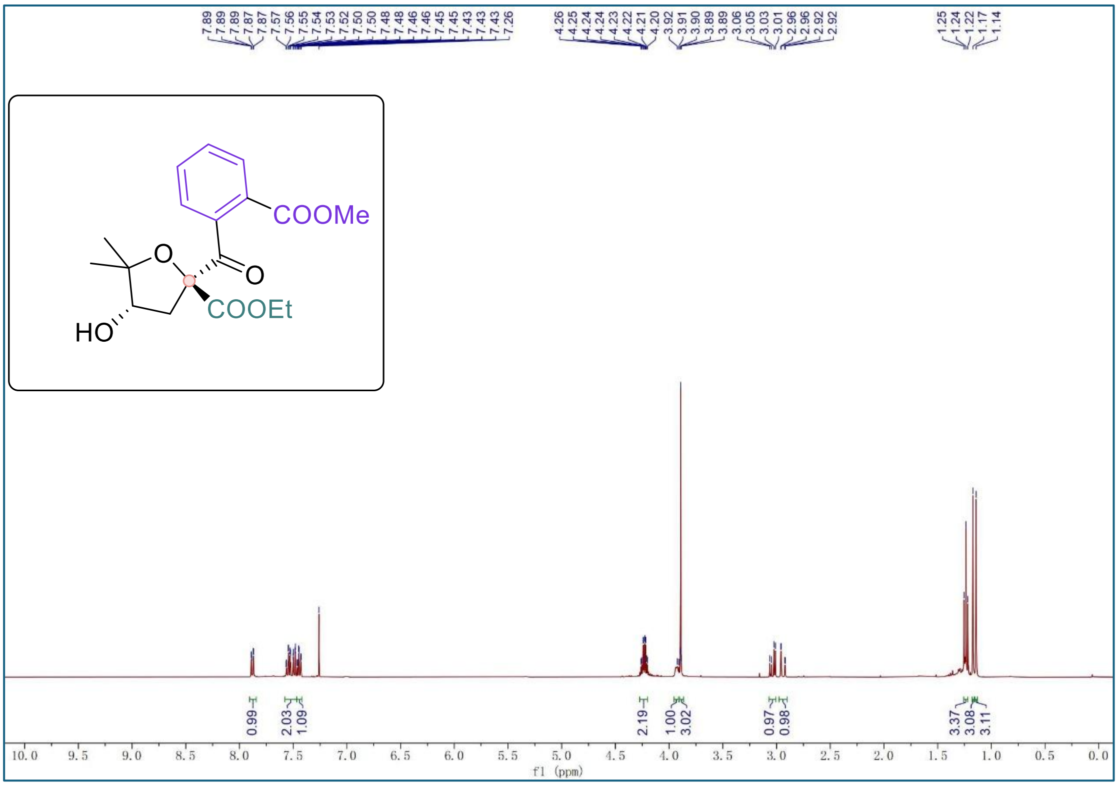

^{1}H NMR-spectrum (400 MHz, Chloroform-*d*) of **47**

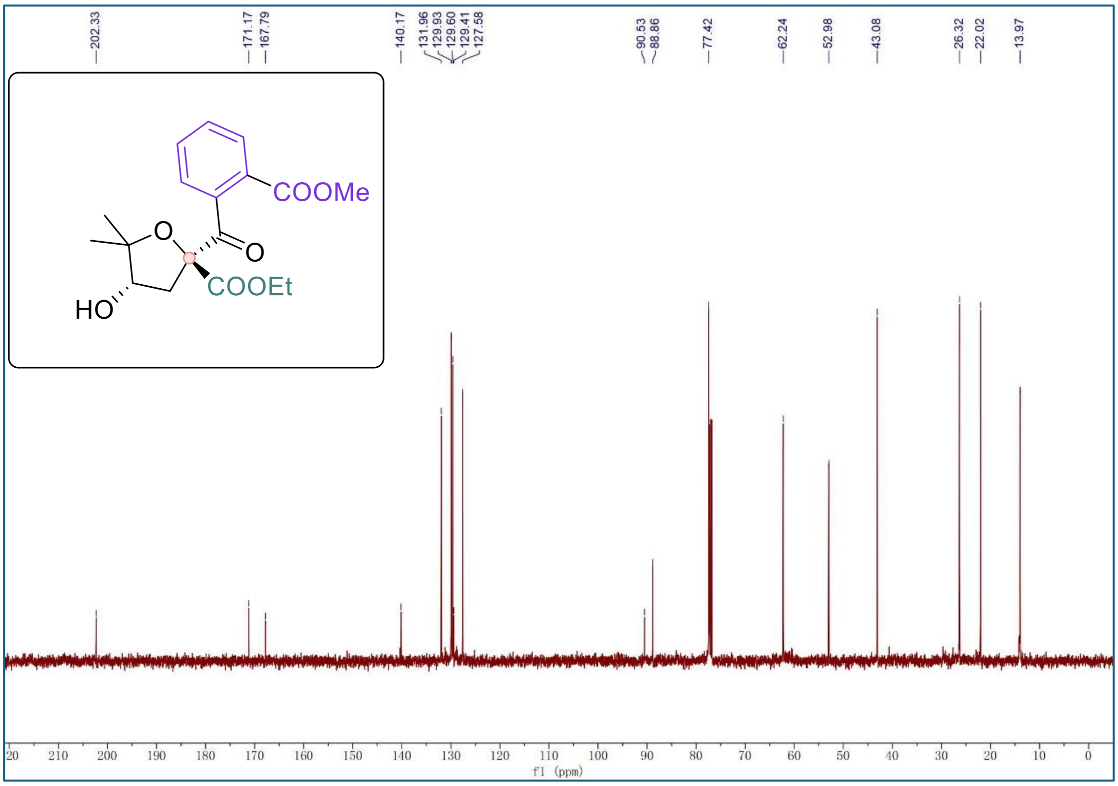

^{13}C NMR-spectrum (126 MHz, Chloroform-*d*) of **47**

ethyl 2-(3,5-dimethylbenzoyl)-4-hydroxy-5,5-dimethyltetrahydrofuran-2-carboxylate (48)

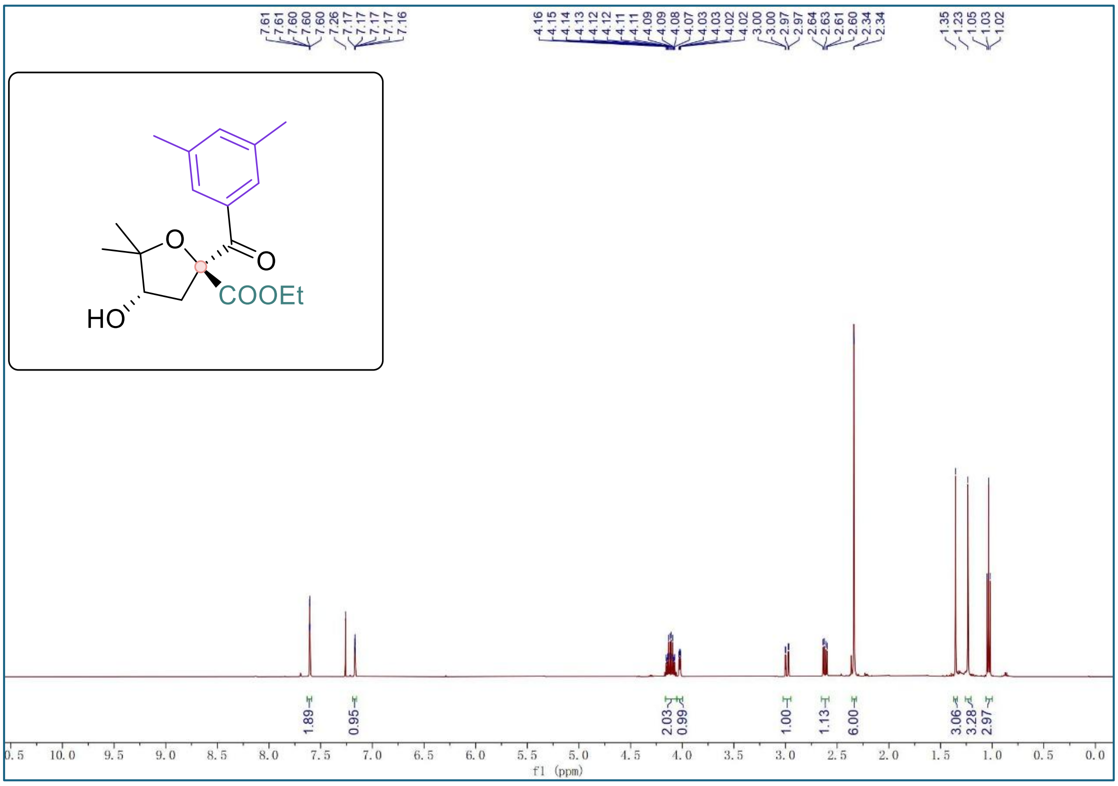

^{1}H NMR-spectrum (500 MHz, Chloroform-*d*) of **48**

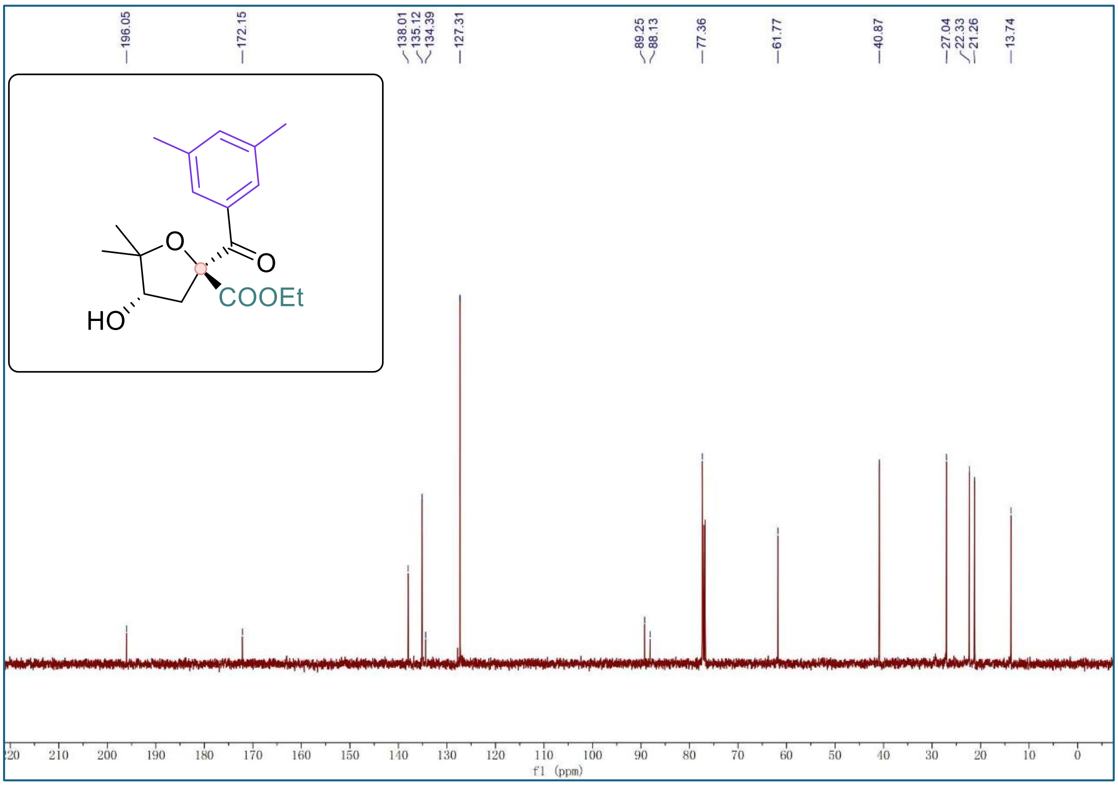

^{13}C NMR-spectrum (126 MHz, Chloroform-*d*) of **48**

ethyl 2-(5-chloro-2-fluorobenzoyl)-4-hydroxy-5,5-dimethyltetrahydrofuran-2-carboxylate (49)

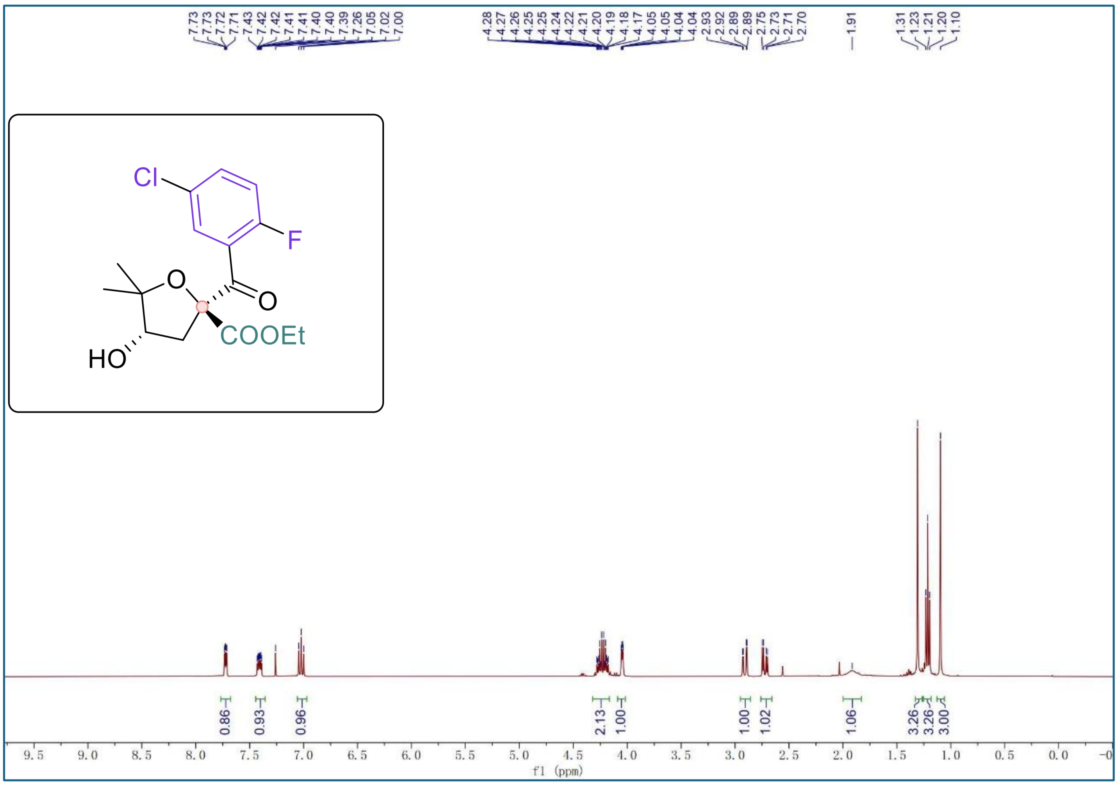

^{1}H NMR-spectrum (400 MHz, Chloroform-*d*) of **49**

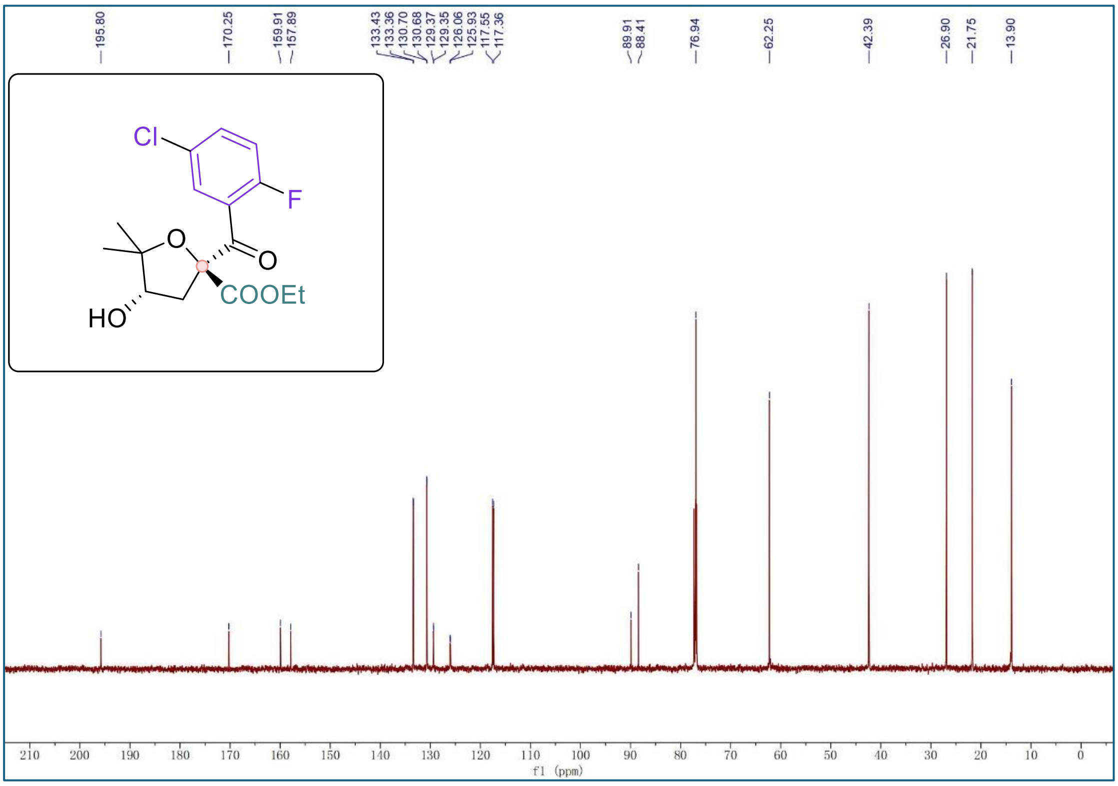

^{13}C NMR-spectrum (126 MHz, Chloroform-*d*) of **49**

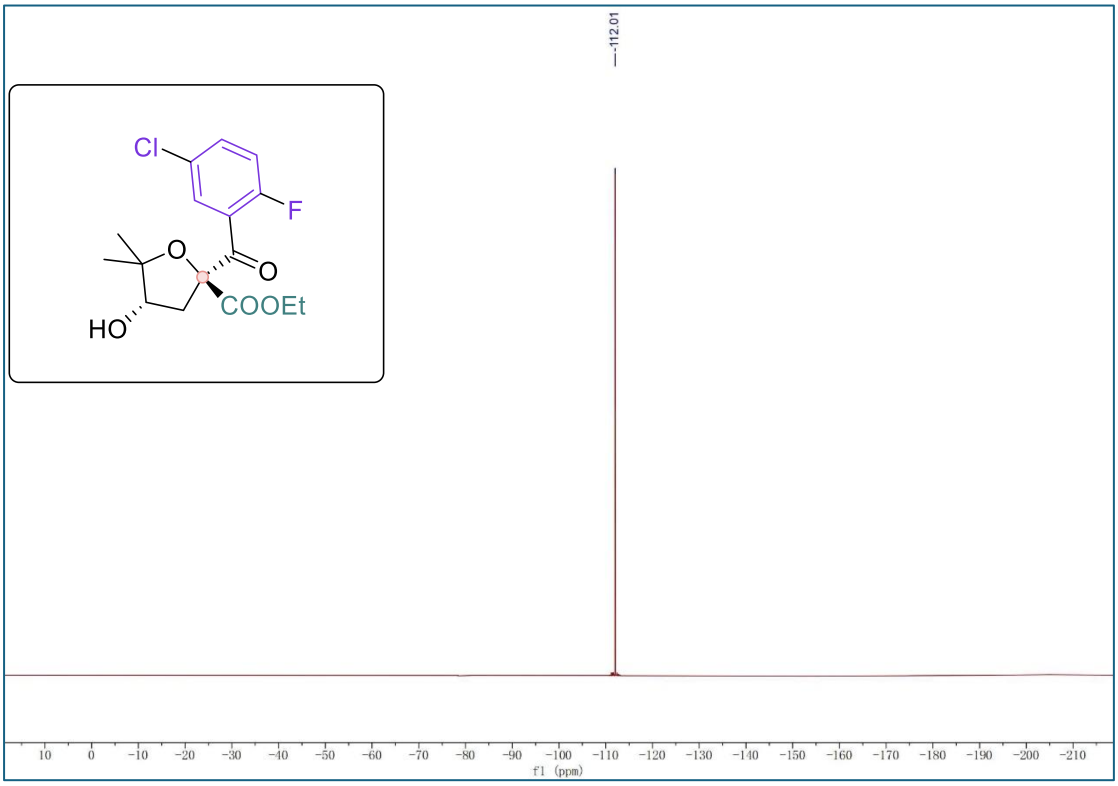

^{19}F NMR-spectrum (377 MHz, Chloroform-*d*) of **49**

ethyl 2-(3,4-dichlorobenzoyl)-4-hydroxy-5,5-dimethyltetrahydrofuran-2-carboxylate (50)

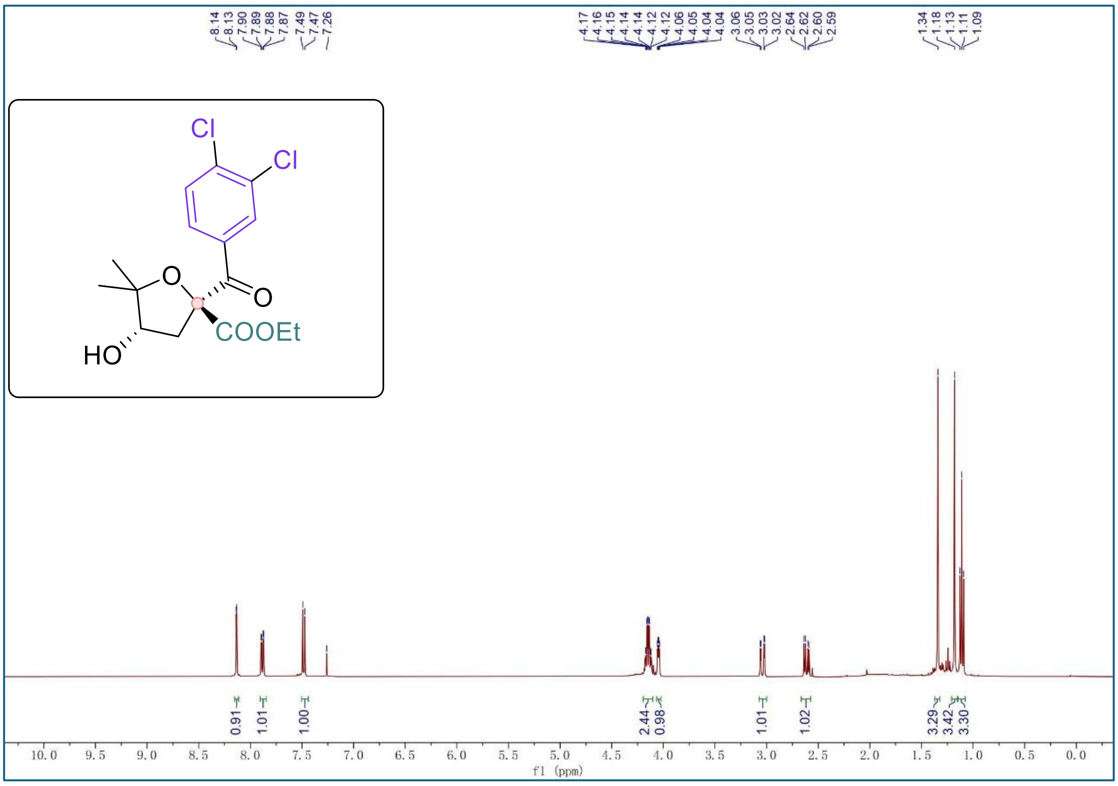

^{1}H NMR-spectrum (400 MHz, Chloroform-*d*) of **50**

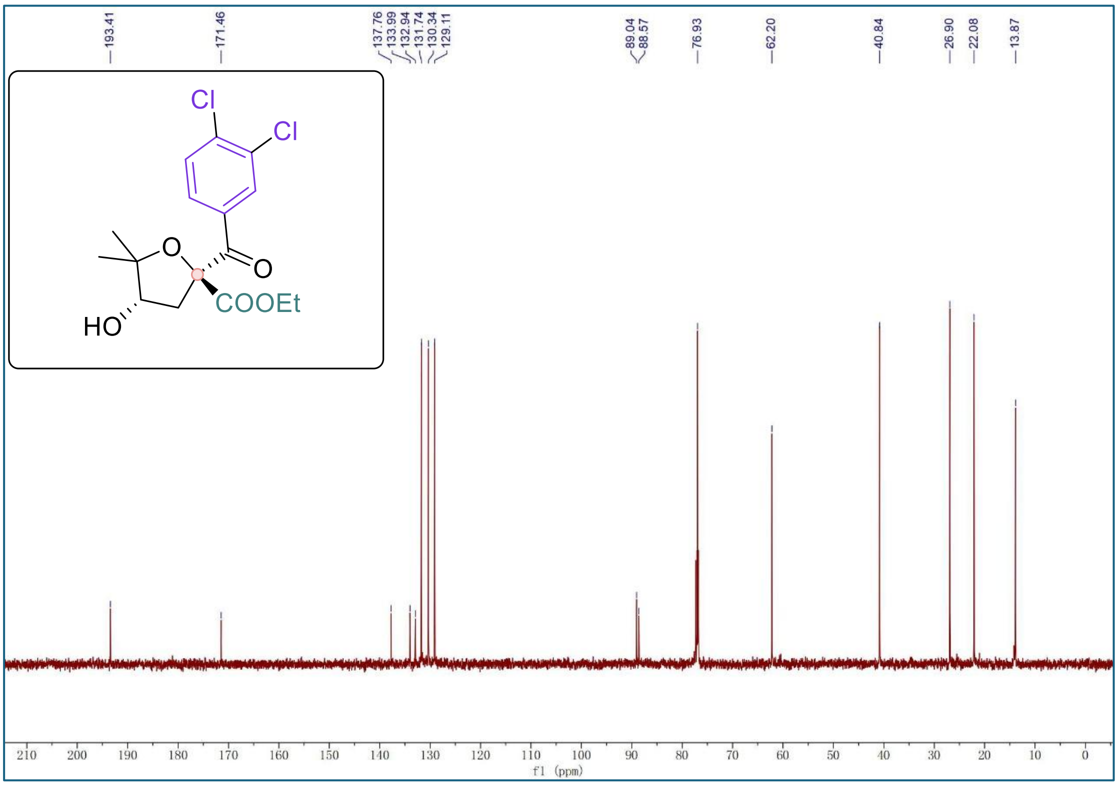

^{13}C NMR-spectrum (126 MHz, Chloroform-*d*) of **50**

ethyl 2-(3,4-difluorobenzoyl)-4-hydroxy-5,5-dimethyltetrahydrofuran-2-carboxylate (51)

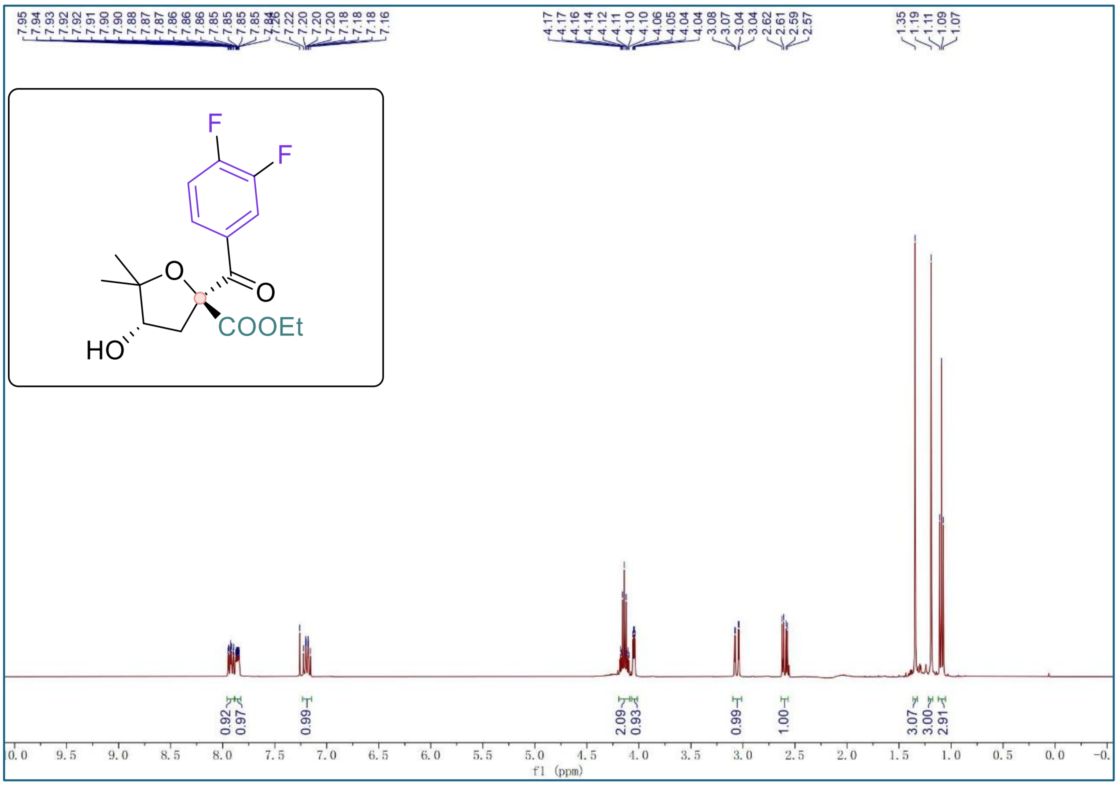

^{1}H NMR-spectrum (400 MHz, Chloroform-*d*) of **51**

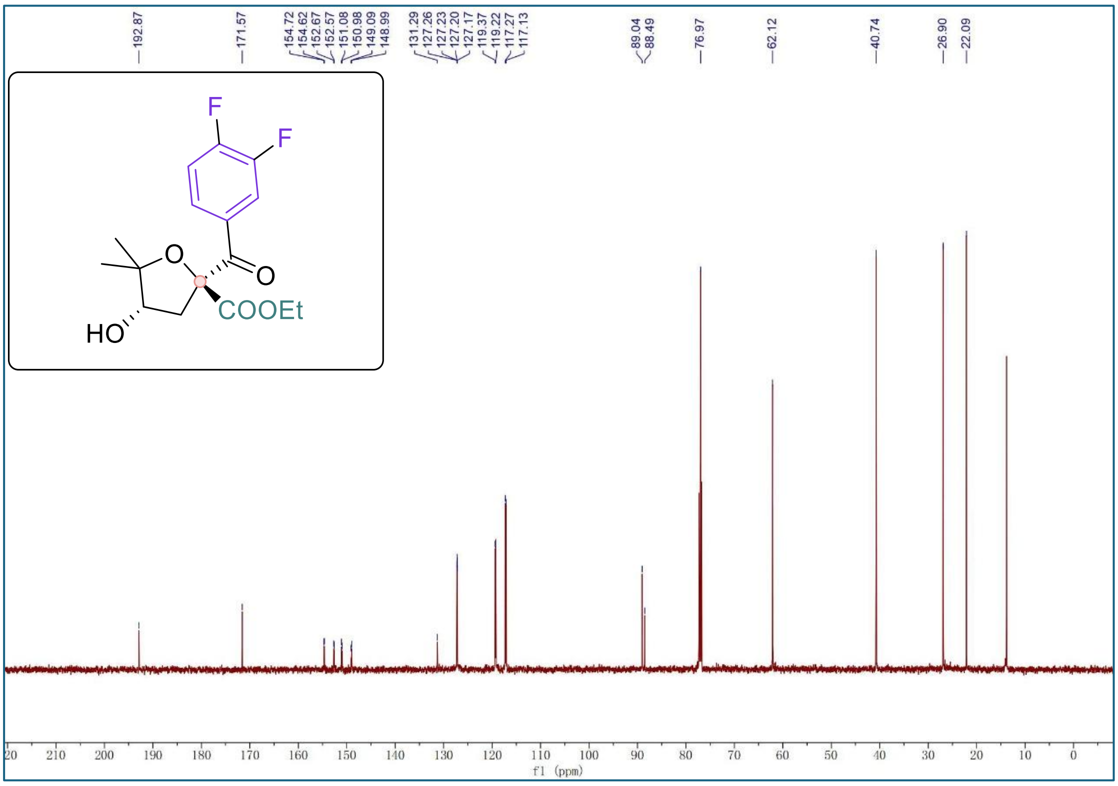

^{13}C NMR-spectrum (126 MHz, Chloroform-*d*) of **51**

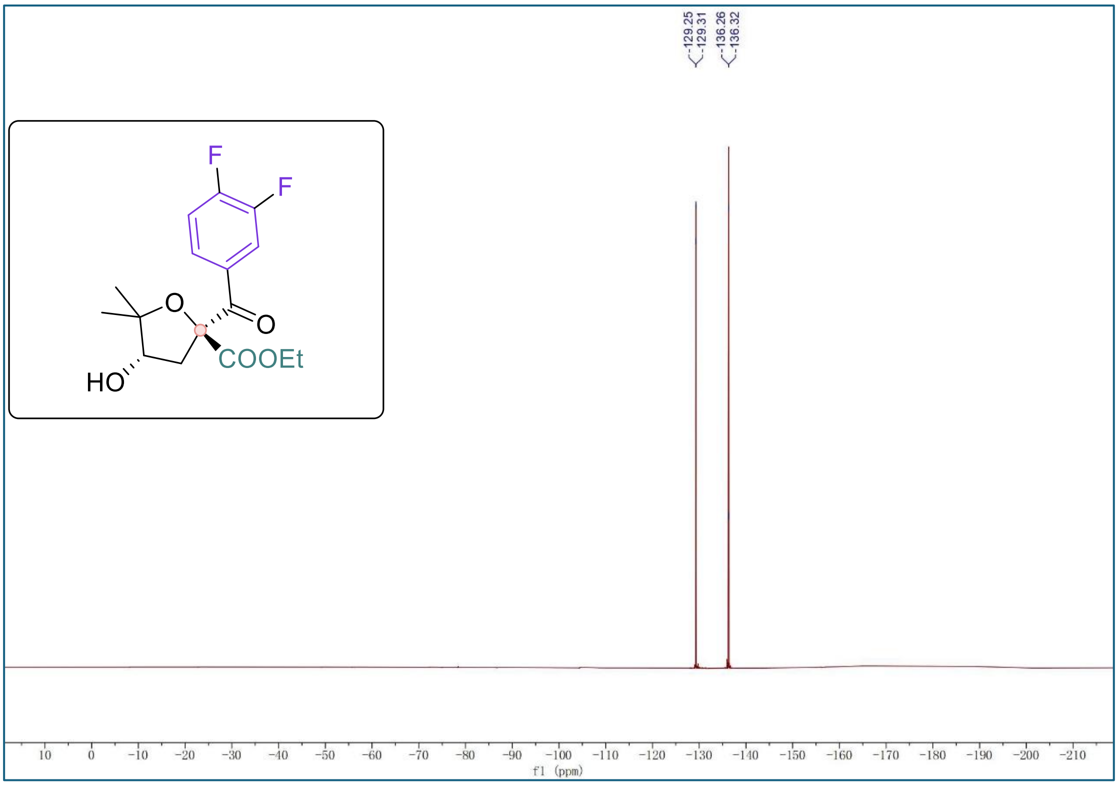

^{19}F NMR-spectrum (377 MHz, Chloroform-*d*) of **51**

ethyl 2-(3-chloro-4-methylbenzoyl)-4-hydroxy-5,5-dimethyltetrahydrofuran-2-carboxylate (52)

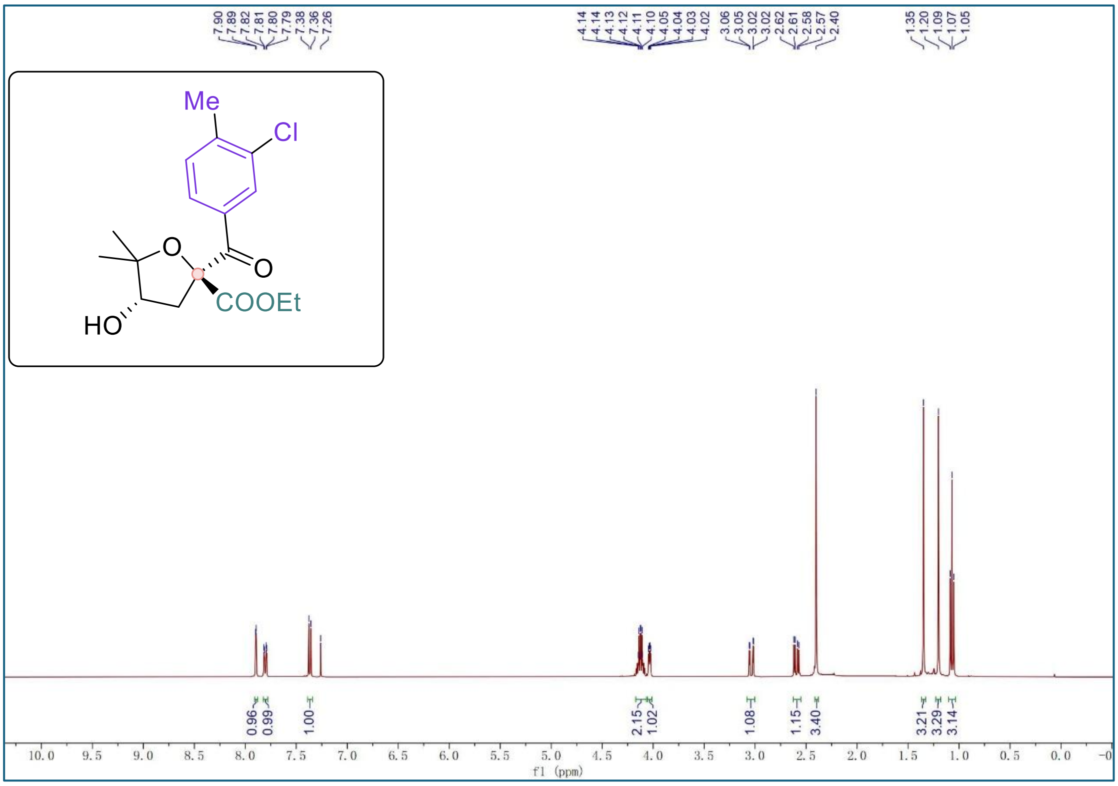

^{1}H NMR-spectrum (400 MHz, Chloroform-*d*) of **52**

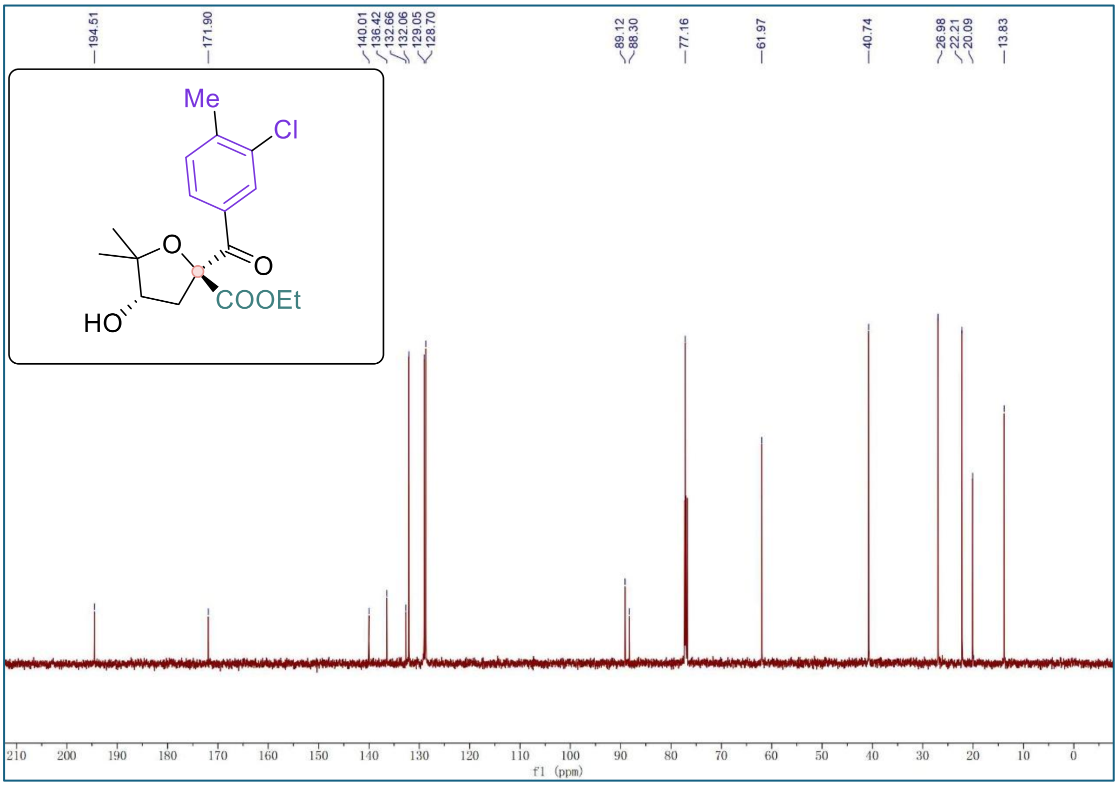

^{13}C NMR-spectrum (126 MHz, Chloroform-*d*) of **52**

ethyl 2-(5-chloro-2-(trifluoromethyl)benzoyl)-4-hydroxy-5,5-dimethyltetrahydrofuran-2-carboxylate (53)

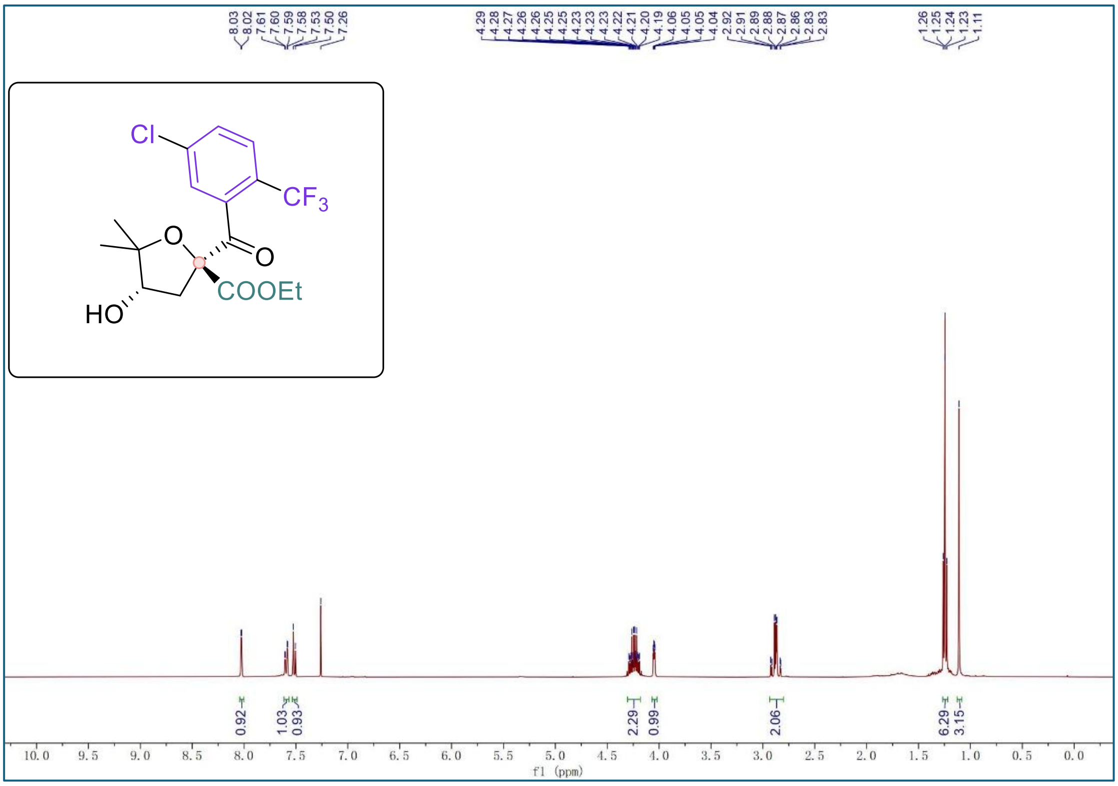

^{1}H NMR-spectrum (400 MHz, Chloroform-*d*) of **53**

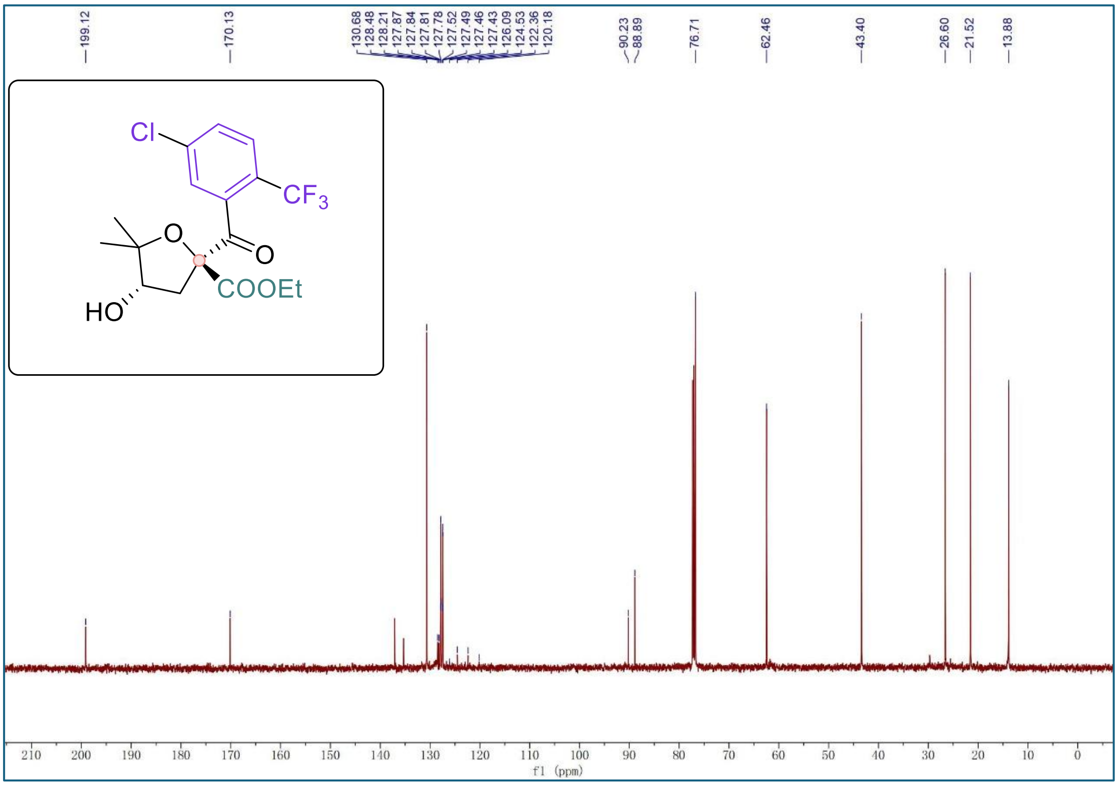

^{13}C NMR-spectrum (126 MHz, Chloroform-*d*) of **53**

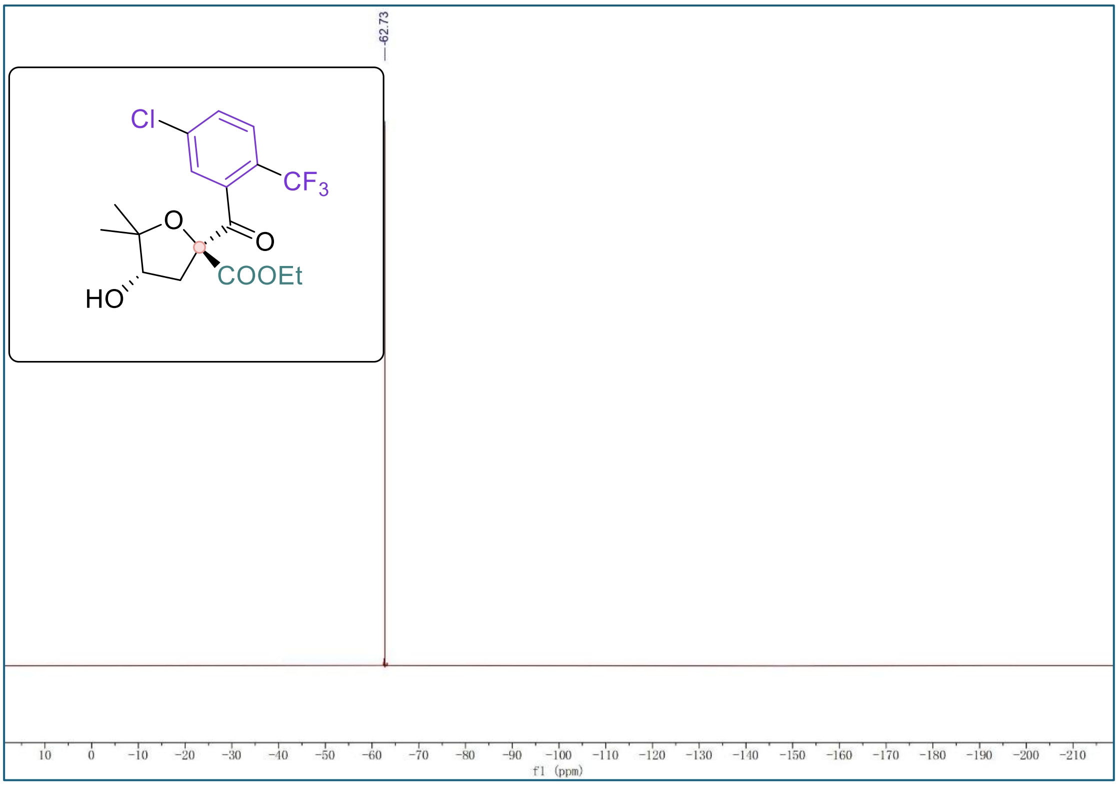

^{19}F NMR-spectrum (377 MHz, Chloroform-*d*) of **53**

ethyl 2-(6-bromo-2-naphthoyl)-4-hydroxy-5,5-dimethyltetrahydrofuran-2-carboxylate (54)

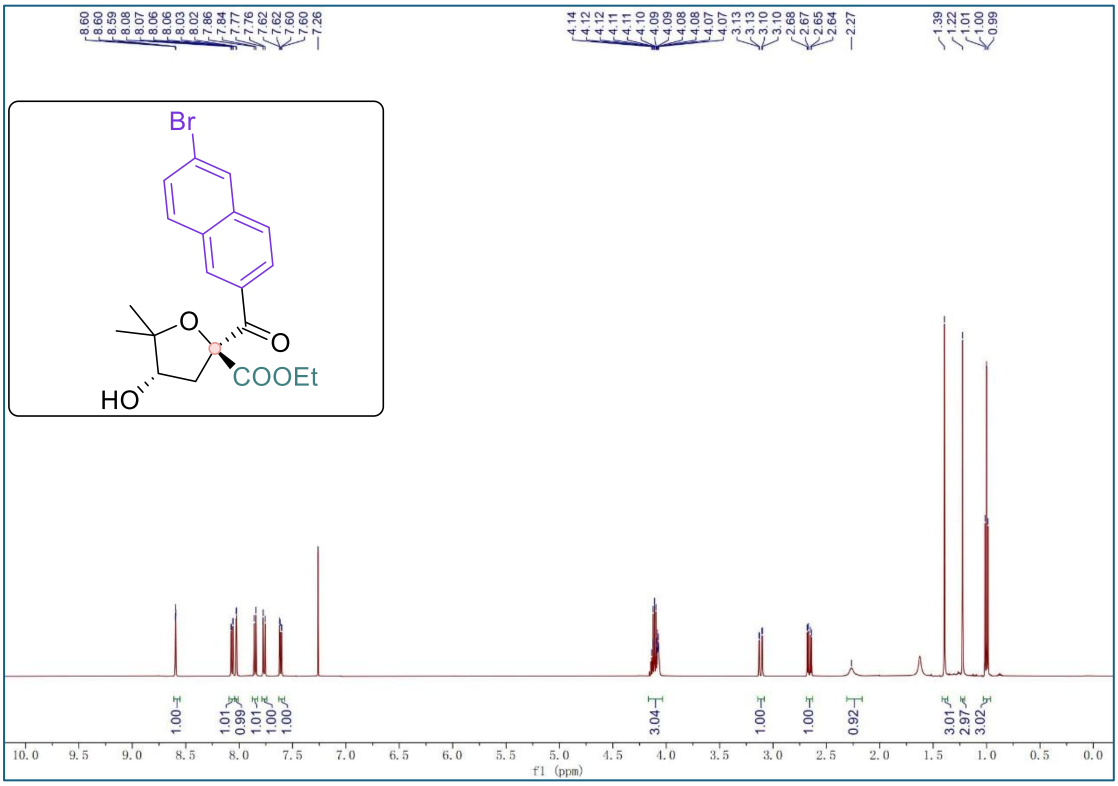

^{1}H NMR-spectrum (500 MHz, Chloroform-*d*) of **54**

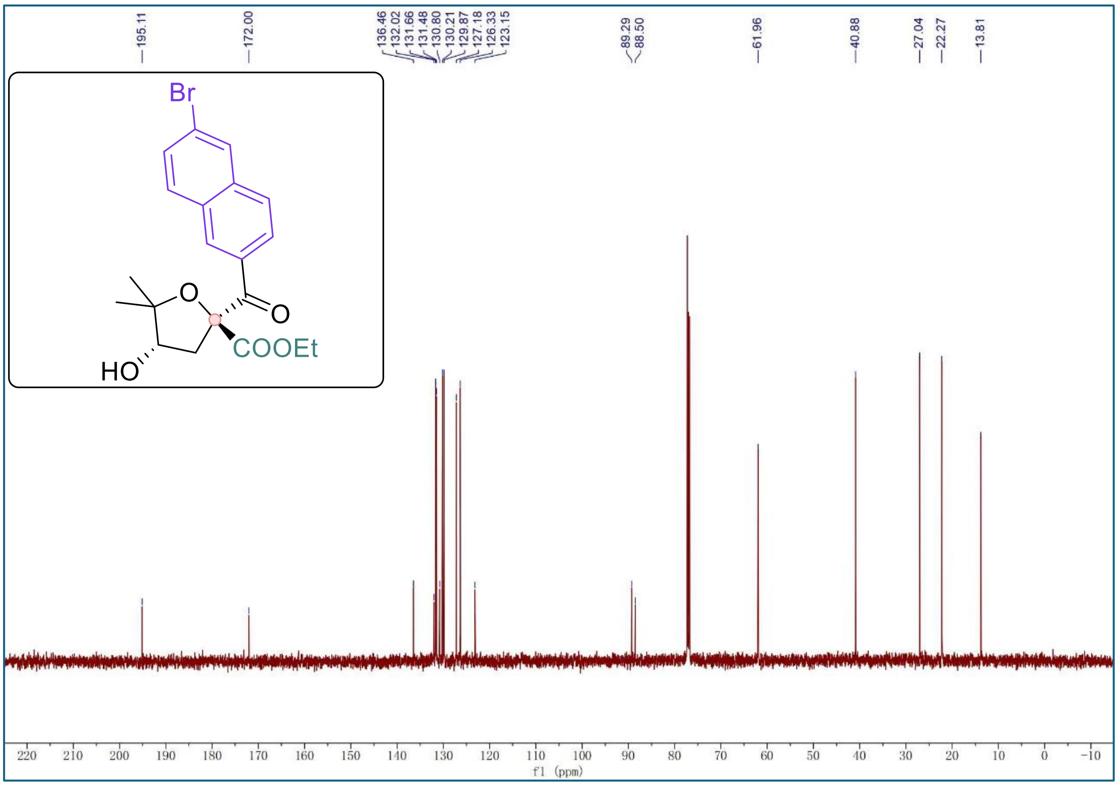

^{13}C NMR-spectrum (126 MHz, Chloroform-*d*) of **54**

ethyl 2-(5-chlorothiophene-2-carbonyl)-4-hydroxy-1-oxaspiro[4.5]decane-2-carboxylate (55)

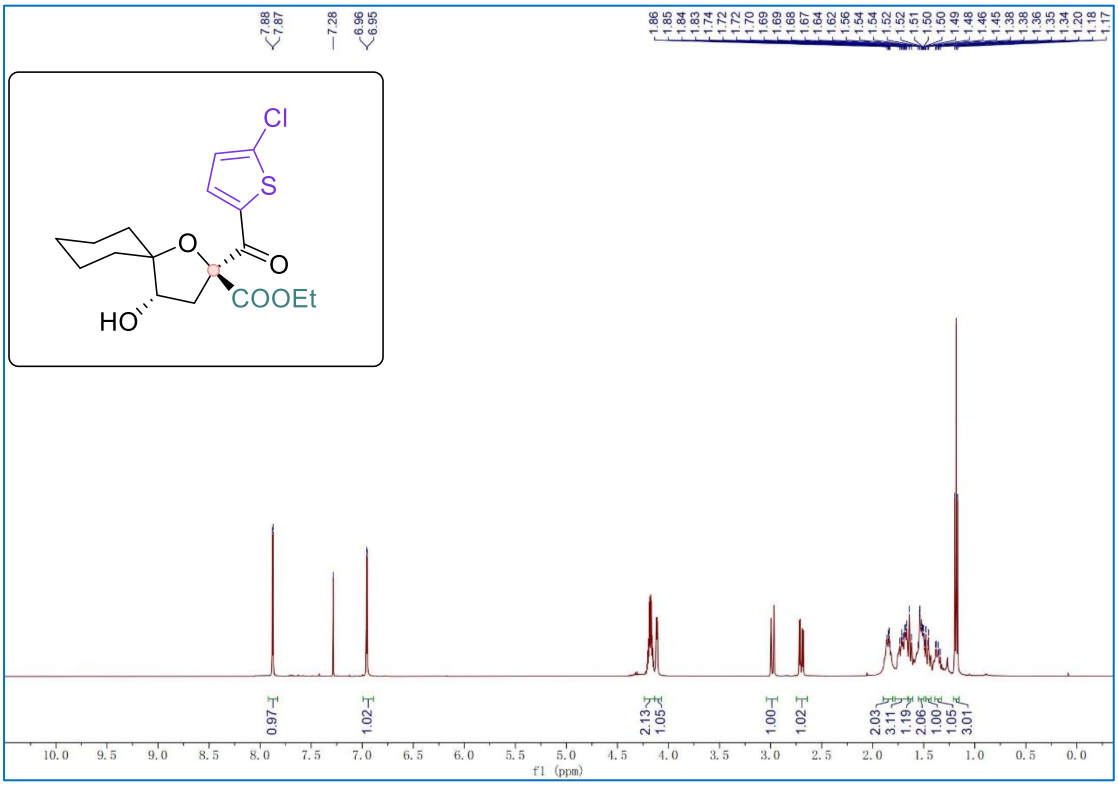

^{1}H NMR-spectrum (500 MHz, Chloroform-*d*) of **55**

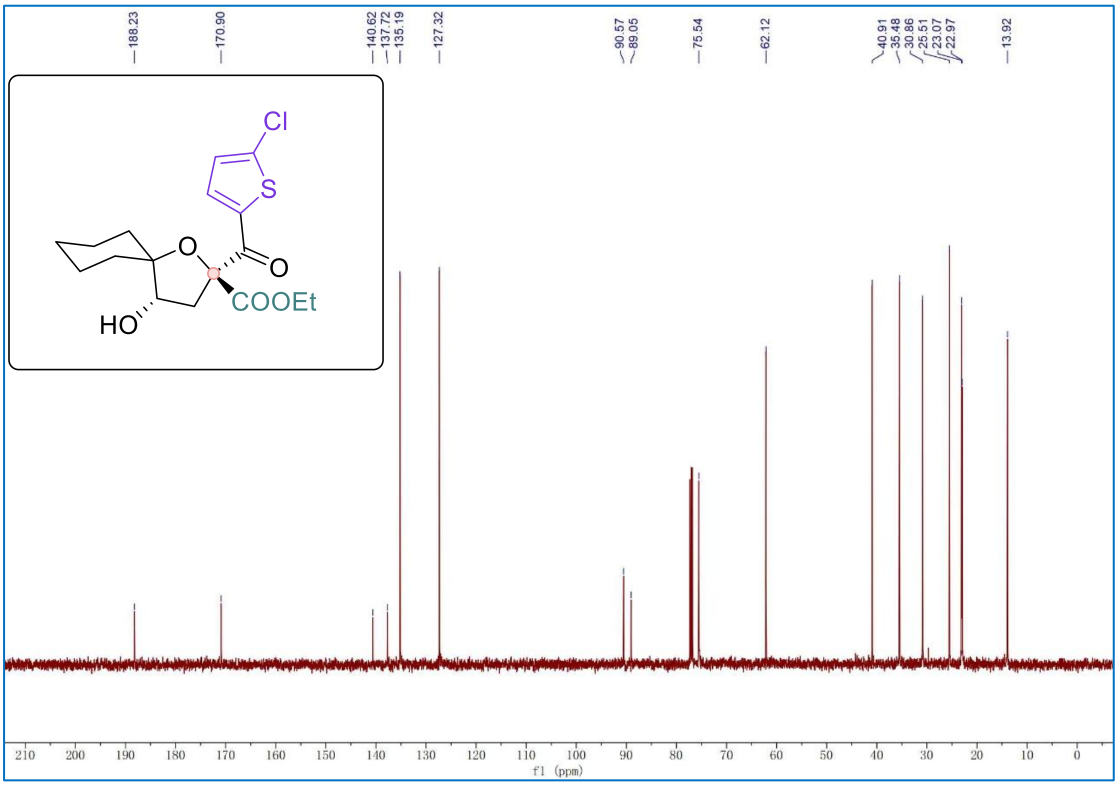

^{13}C NMR-spectrum (126 MHz, Chloroform-*d*) of **55**

ethyl 4-hydroxy-5,5-dimethyl-2-(thiophene-2-carbonyl)tetrahydrofuran-2-carboxylate (56)

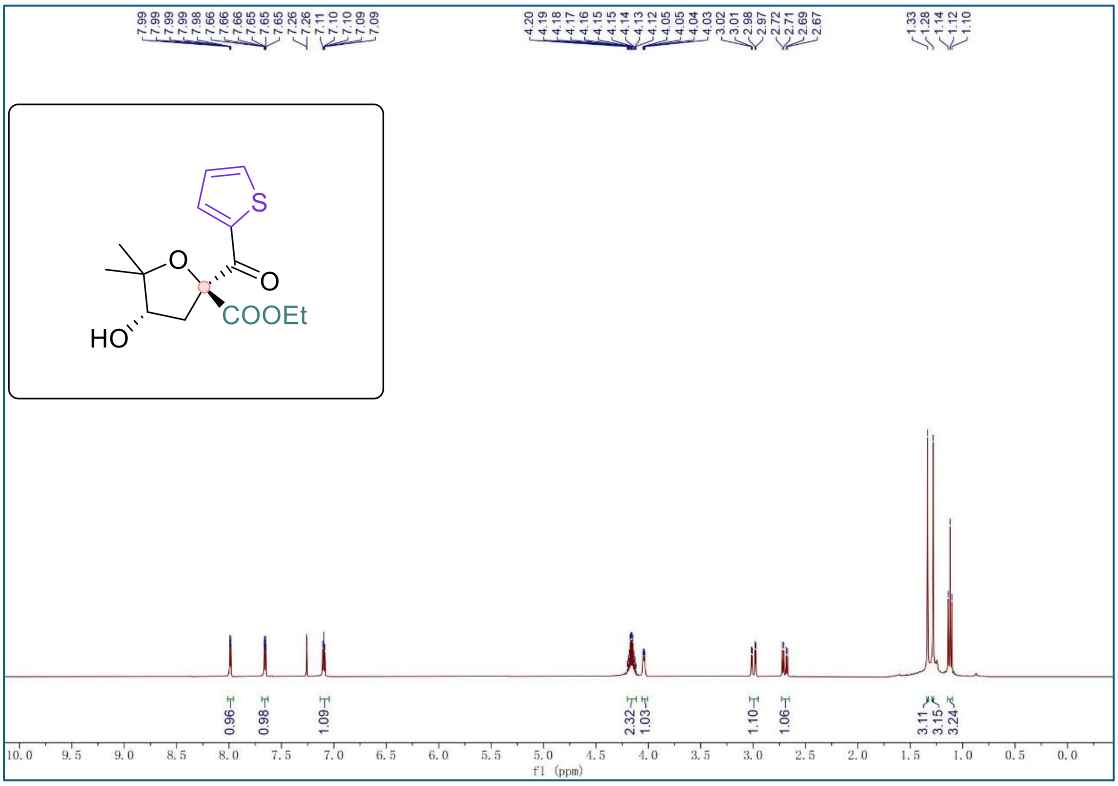

^{1}H NMR-spectrum (400 MHz, Chloroform-*d*) of **56**

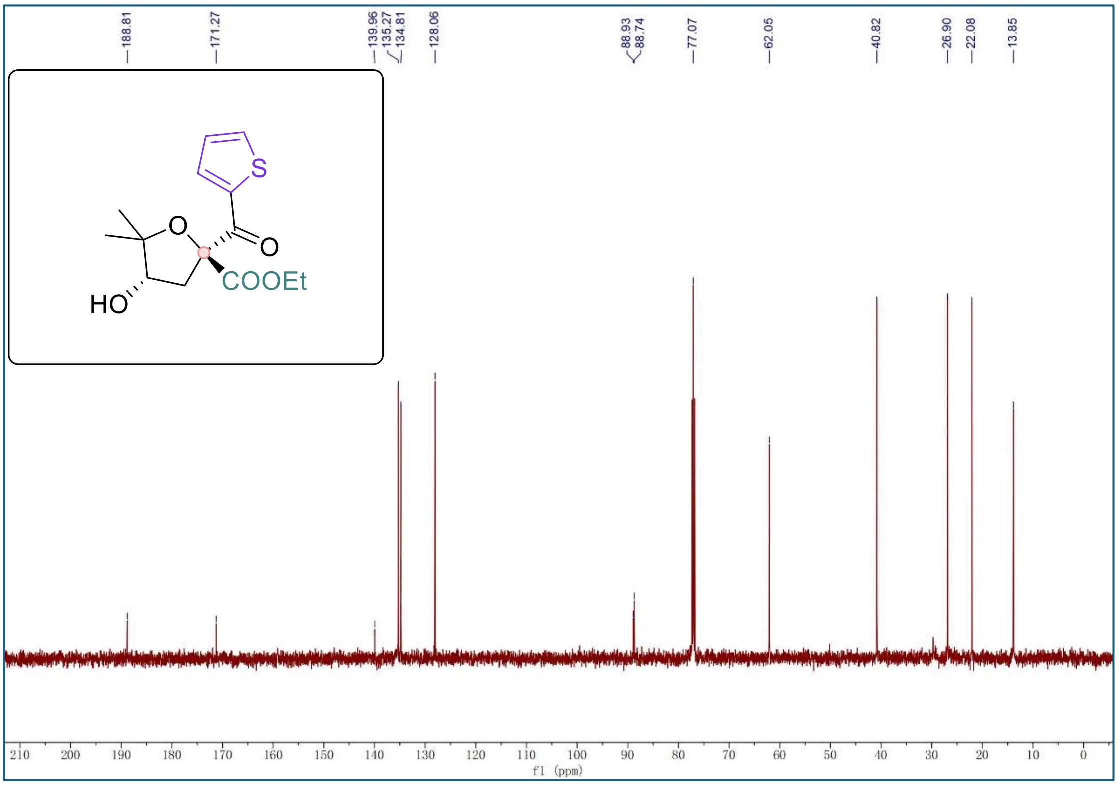

^{13}C NMR-spectrum (126 MHz, Chloroform-*d*) of **56**

ethyl 2-(5-bromothiophene-2-carbonyl)-4-hydroxy-5,5-dimethyltetrahydrofuran-2-carboxylate (57)

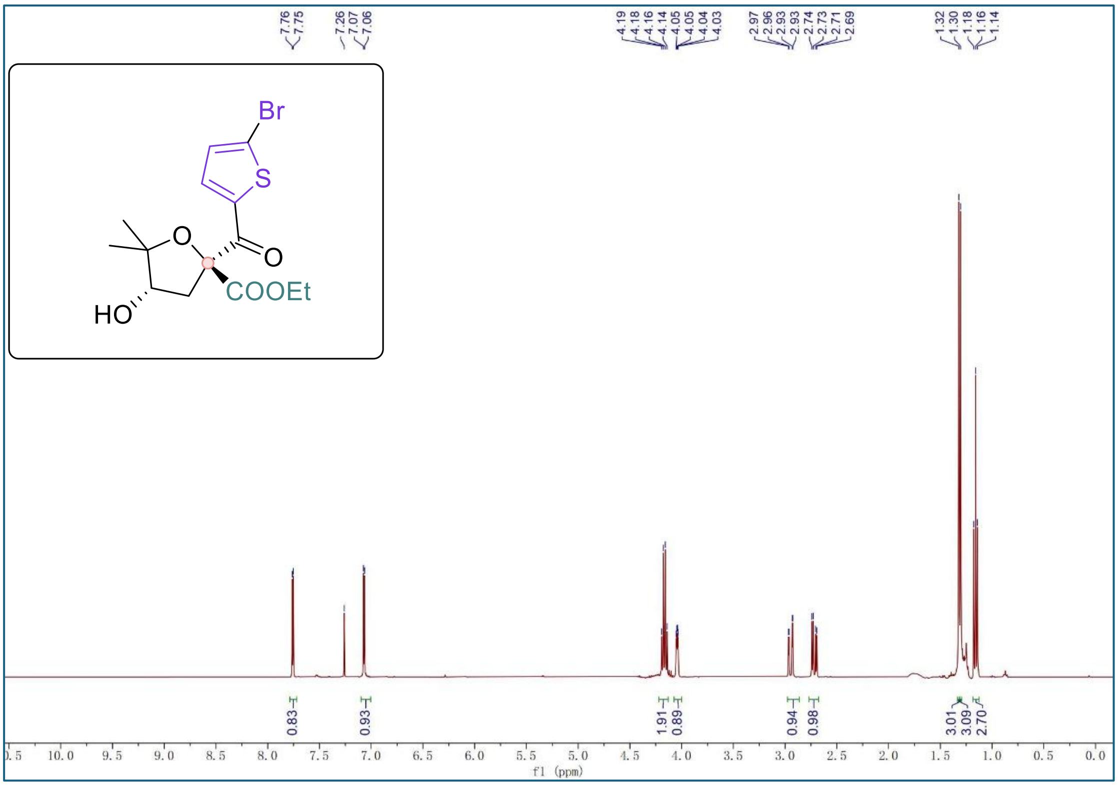

^{1}H NMR-spectrum (400 MHz, Chloroform-*d*) of **57**

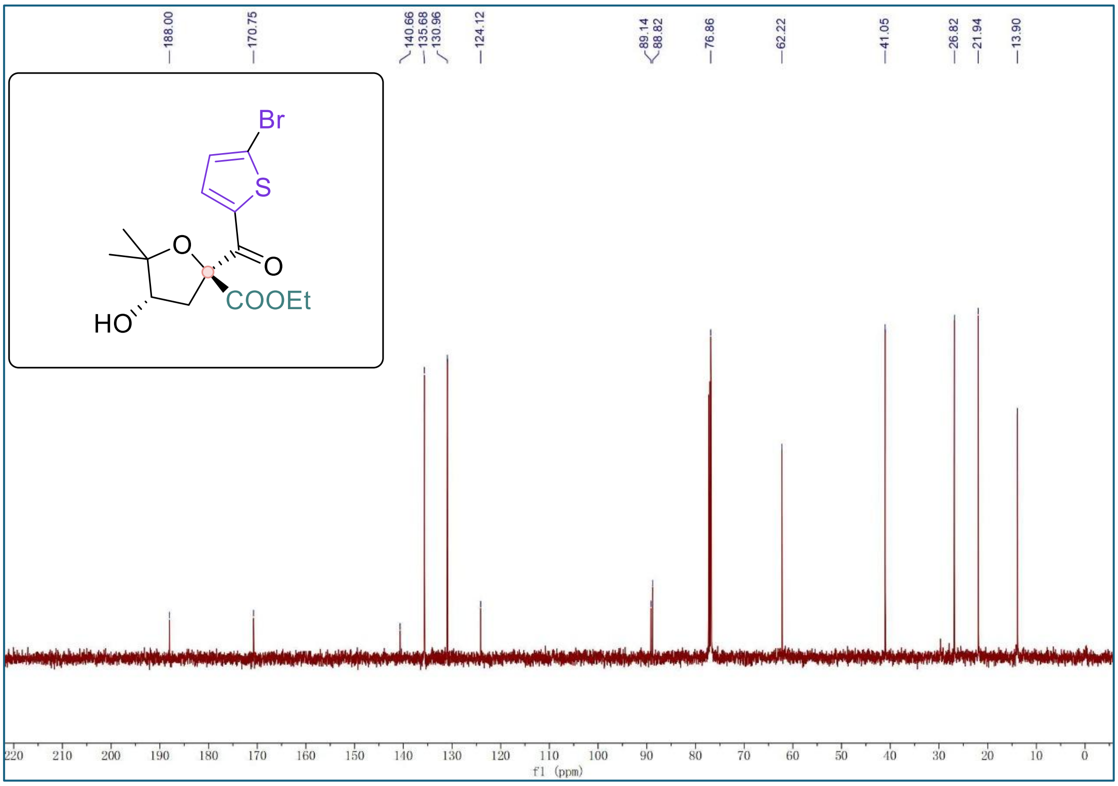

^{13}C NMR-spectrum (126 MHz, Chloroform-*d*) of **57**

ethyl 2-(5-(ethoxycarbonyl)thiophene-2-carbonyl)-4-hydroxy-5,5-dimethyltetrahydrofuran-2-carboxylate (58)

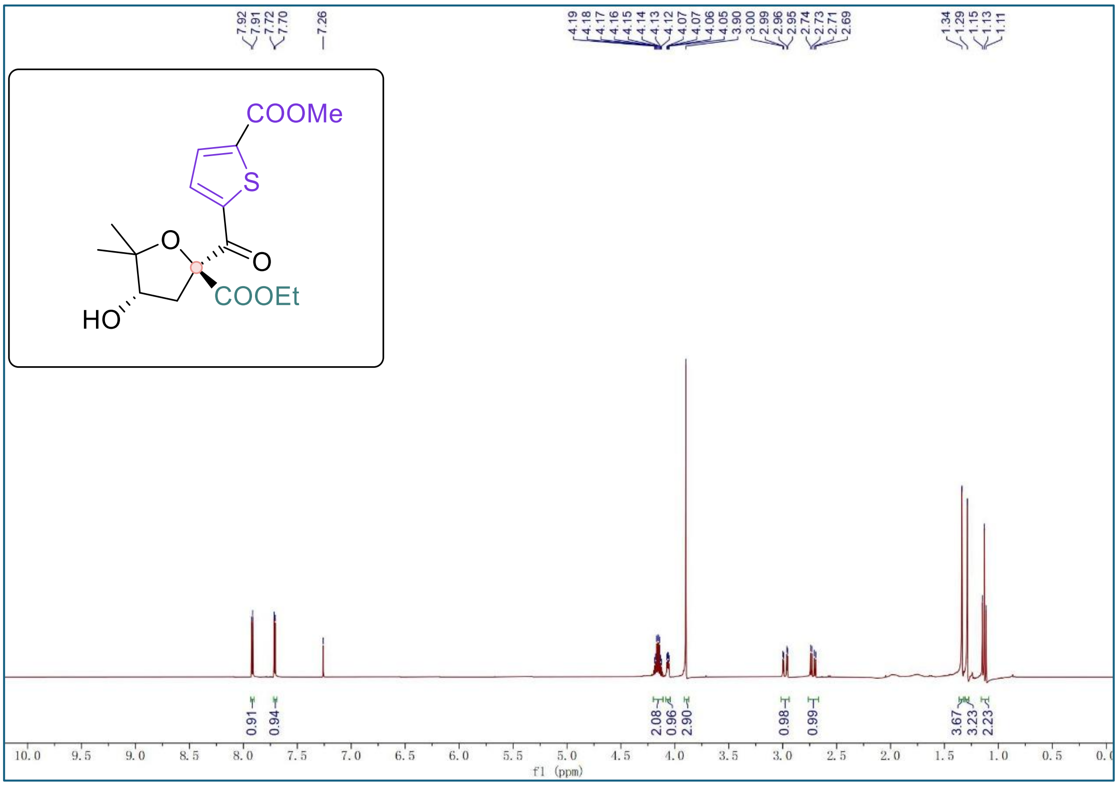

^{1}H NMR-spectrum (400 MHz, Chloroform-*d*) of **58**

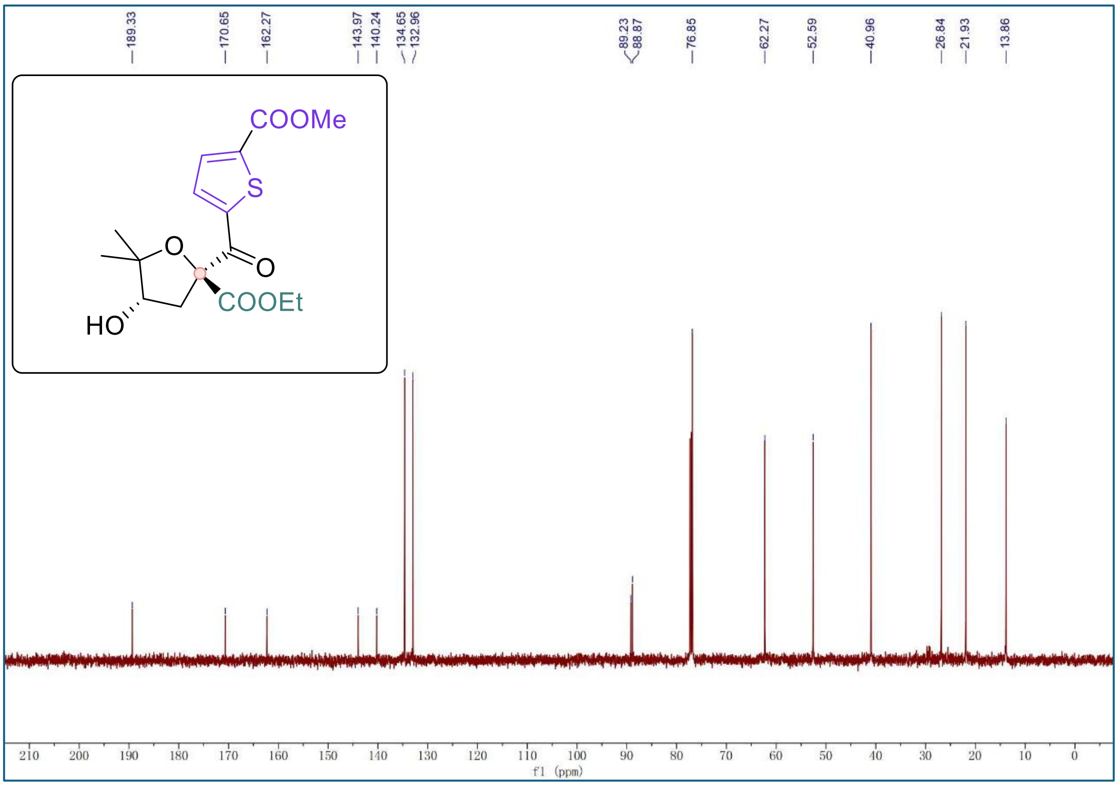

^{13}C NMR-spectrum (126 MHz, Chloroform-*d*) of **58**

ethyl 2-(benzo[b]thiophene-2-carbonyl)-4-hydroxy-5,5-dimethyltetrahydrofuran-2-carboxylate (59)

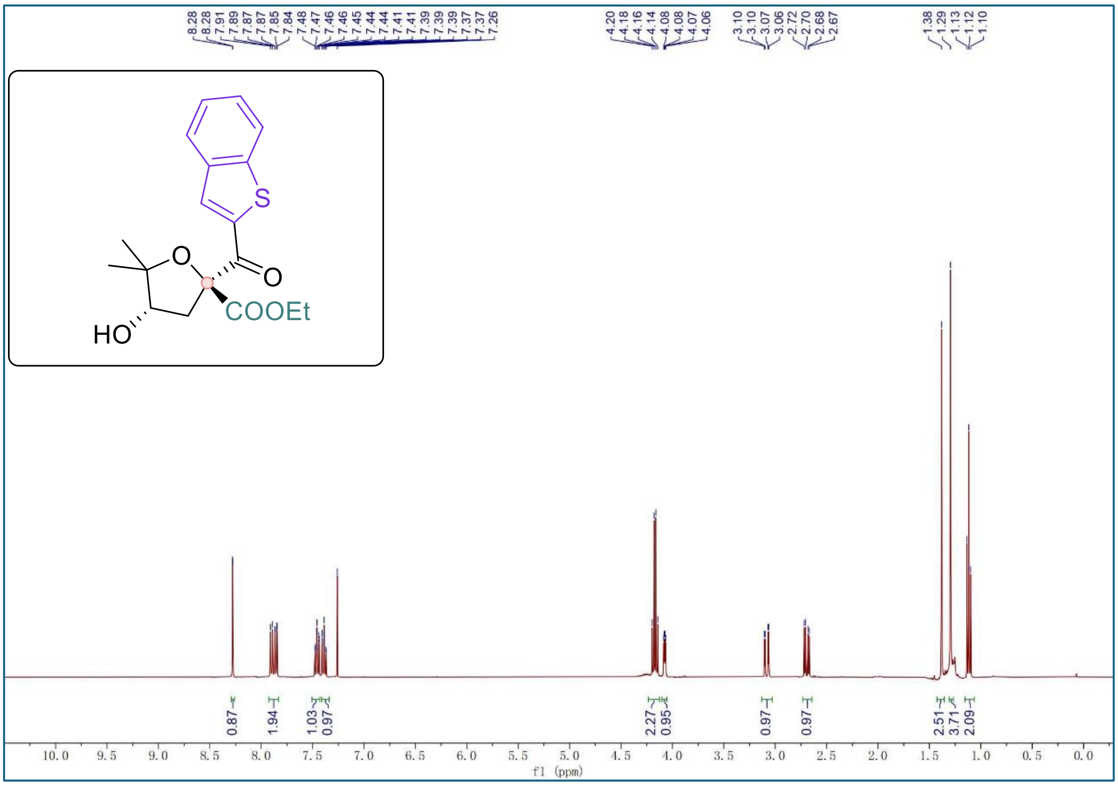

^{1}H NMR-spectrum (400 MHz, Chloroform-*d*) of **59**

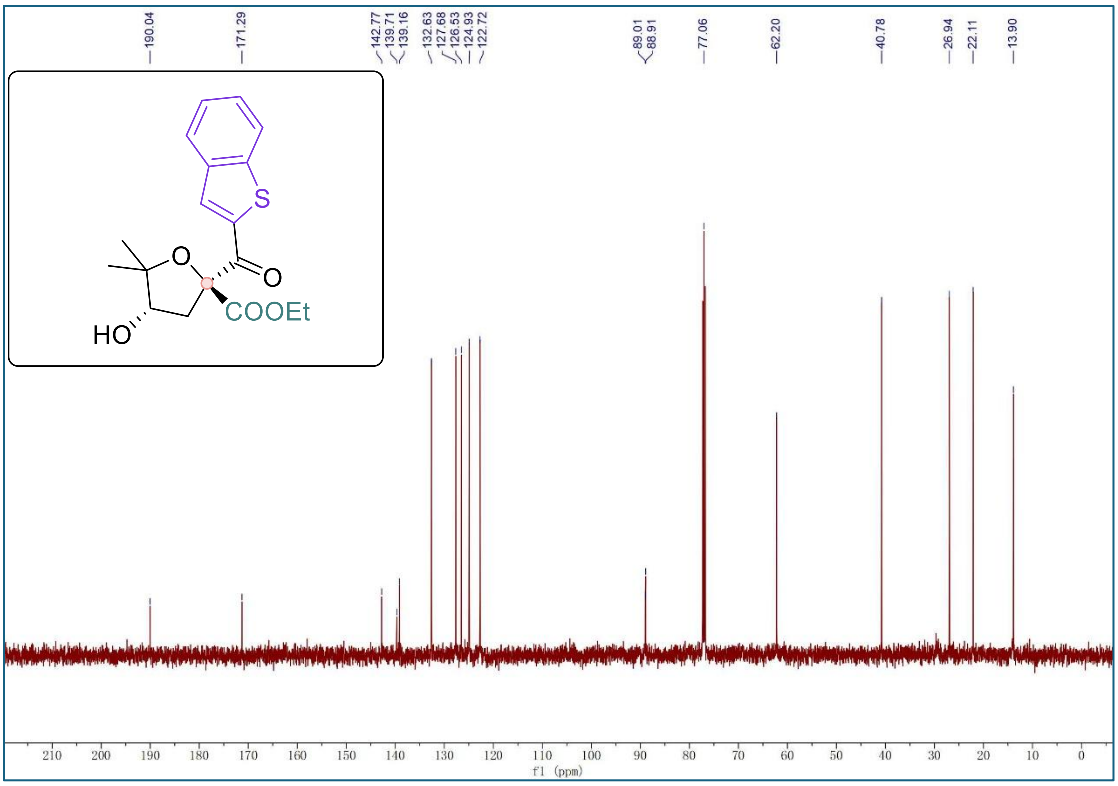

^{13}C NMR-spectrum (126 MHz, Chloroform-*d*) of **59**

SUPPORTING INFORMATION

ethyl 2-(9,10-dioxo-9,10-dihydroanthracene-2-carbonyl)-4-hydroxy-5,5-dimethyltetrahydrofuran-2-carboxylate (60)

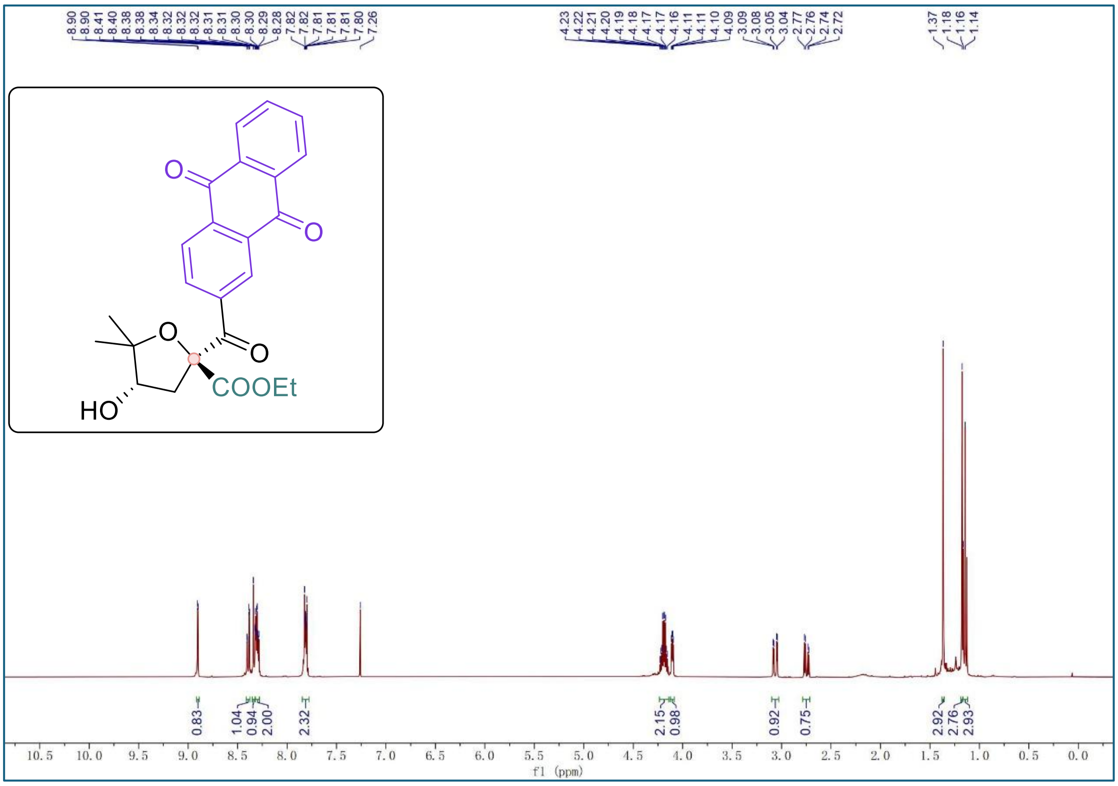

^{1}H NMR-spectrum (400 MHz, Chloroform-*d*) of **60**

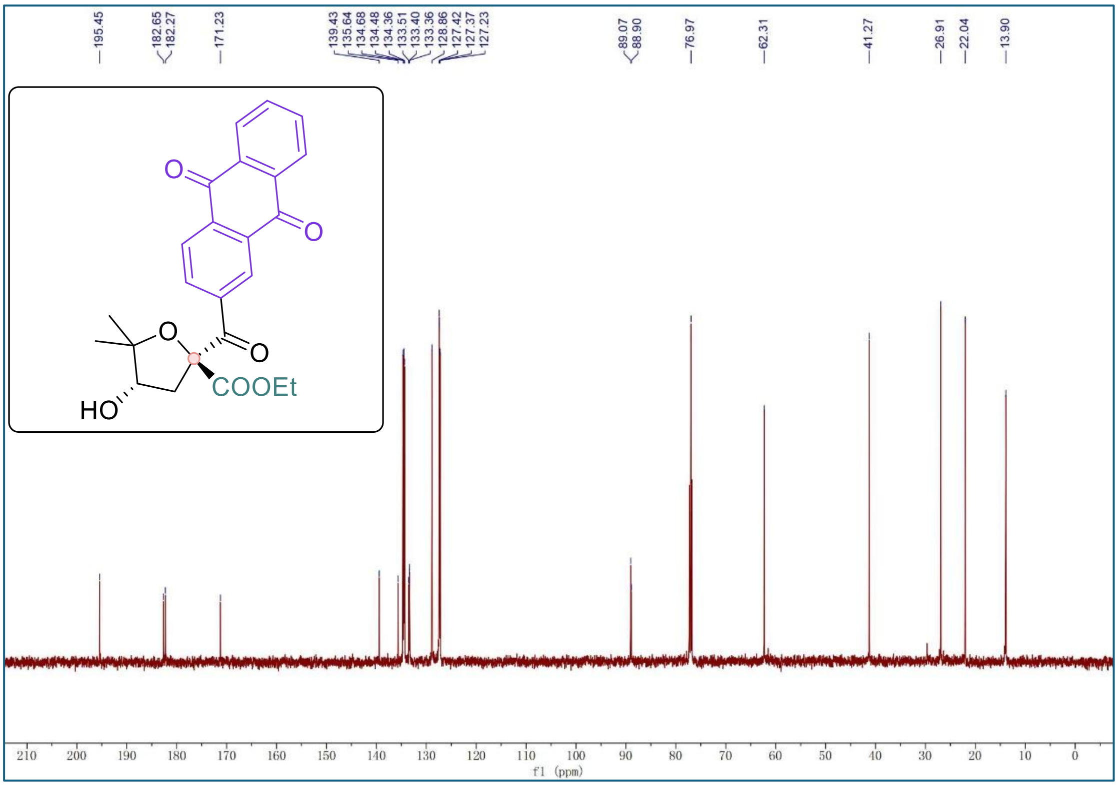

^{13}C NMR-spectrum (126 MHz, Chloroform-*d*) of **60**

ethyl 2-(4-(*N,N*-dipropylsulfamoyl)benzoyl)-4-hydroxy-1-oxaspiro[4.5]decane-2-carboxylate (61)

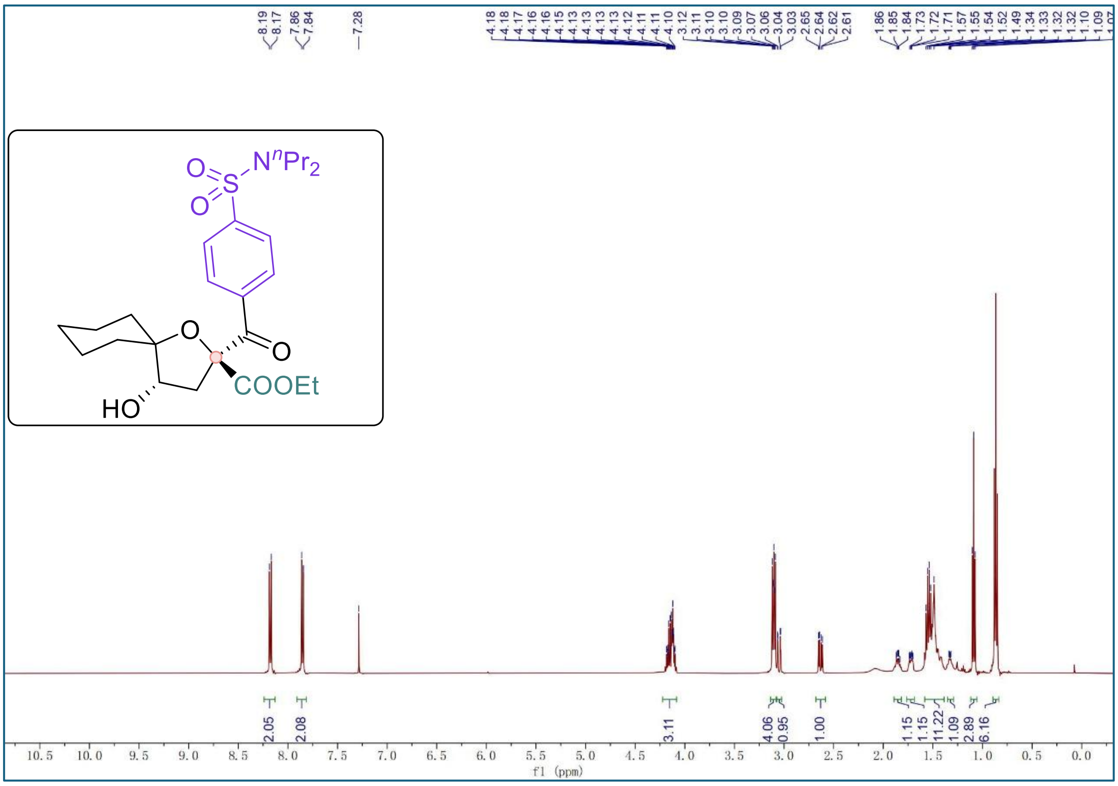

^{1}H NMR-spectrum (500 MHz, Chloroform-*d*) of **61**

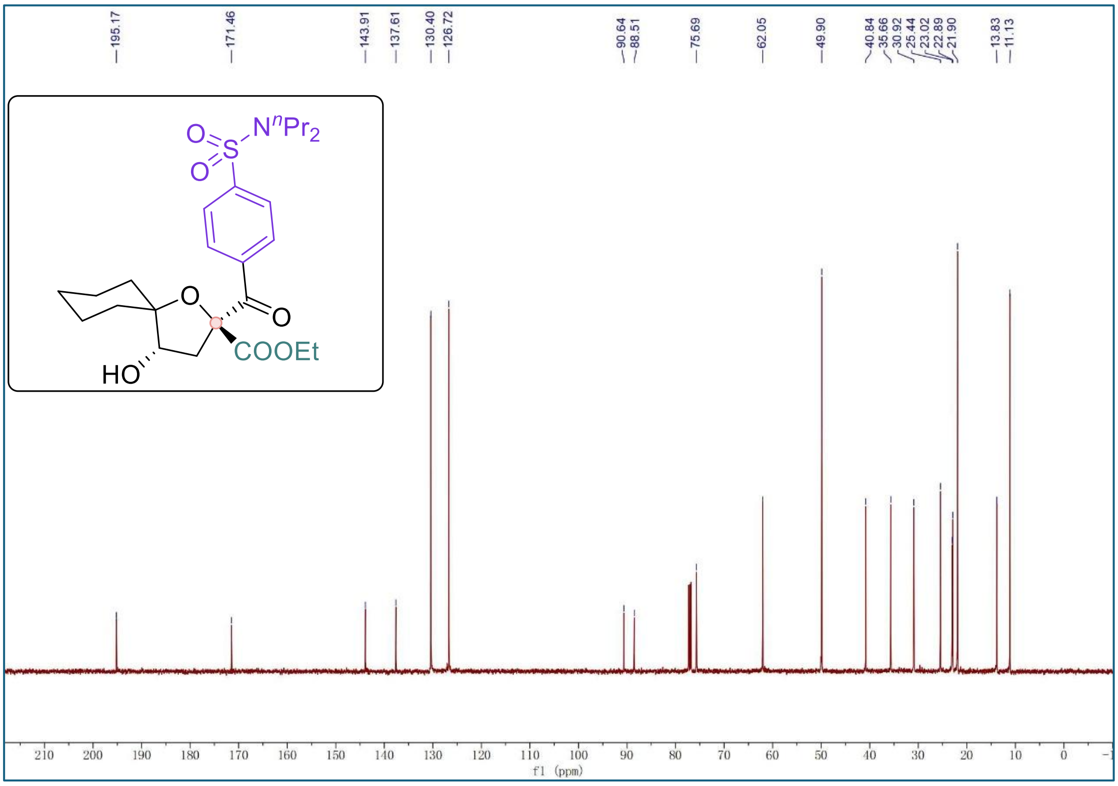

^{13}C NMR-spectrum (126 MHz, Chloroform-*d*) of **61**

ethyl 2-(4-(5-(2-fluorophenyl)-1,2,4-oxadiazol-3-yl)benzoyl)-4-hydroxy-1-oxaspiro[4.5]decane-2-carboxylate (62)

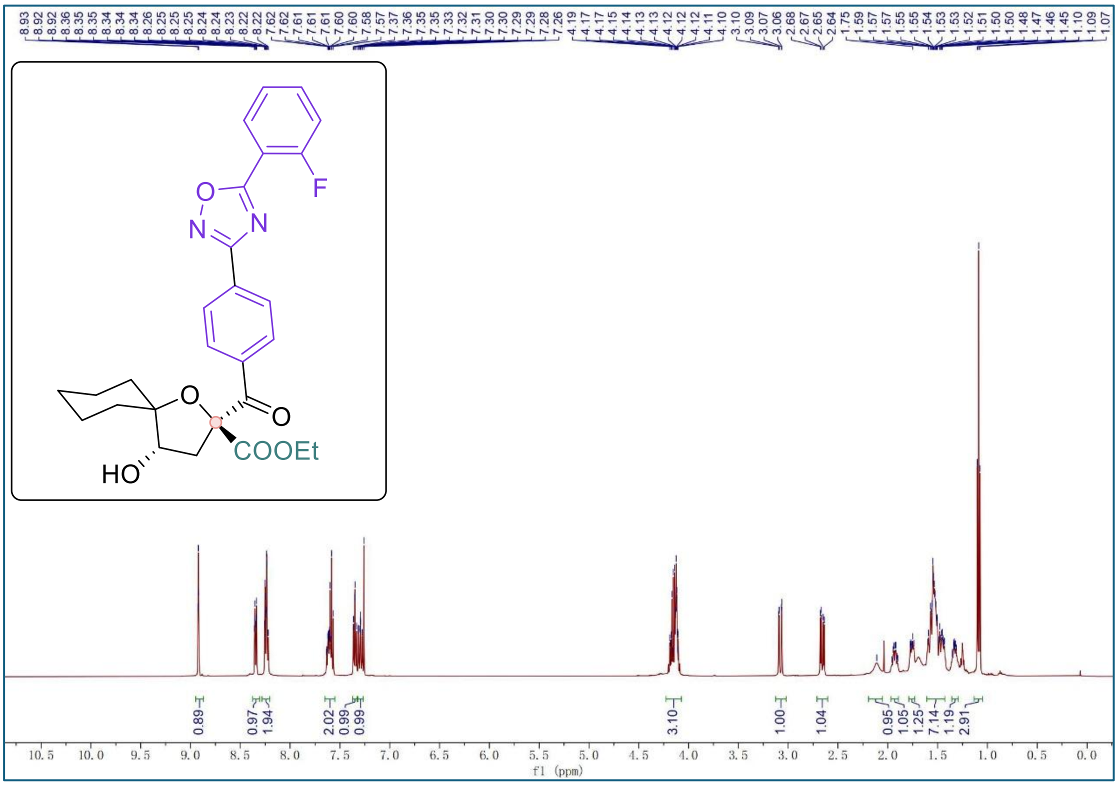

^{1}H NMR-spectrum (400 MHz, Chloroform-*d*) of **62**

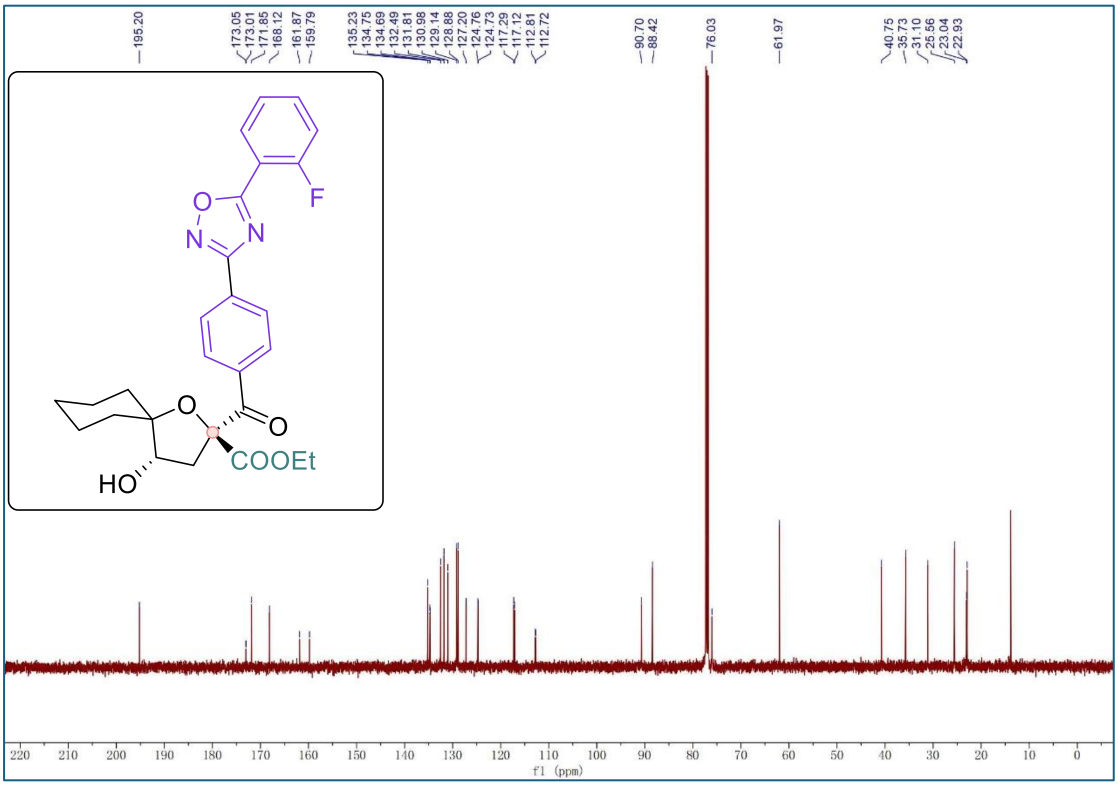

^{13}C NMR-spectrum (126 MHz, Chloroform-*d*) of **62**

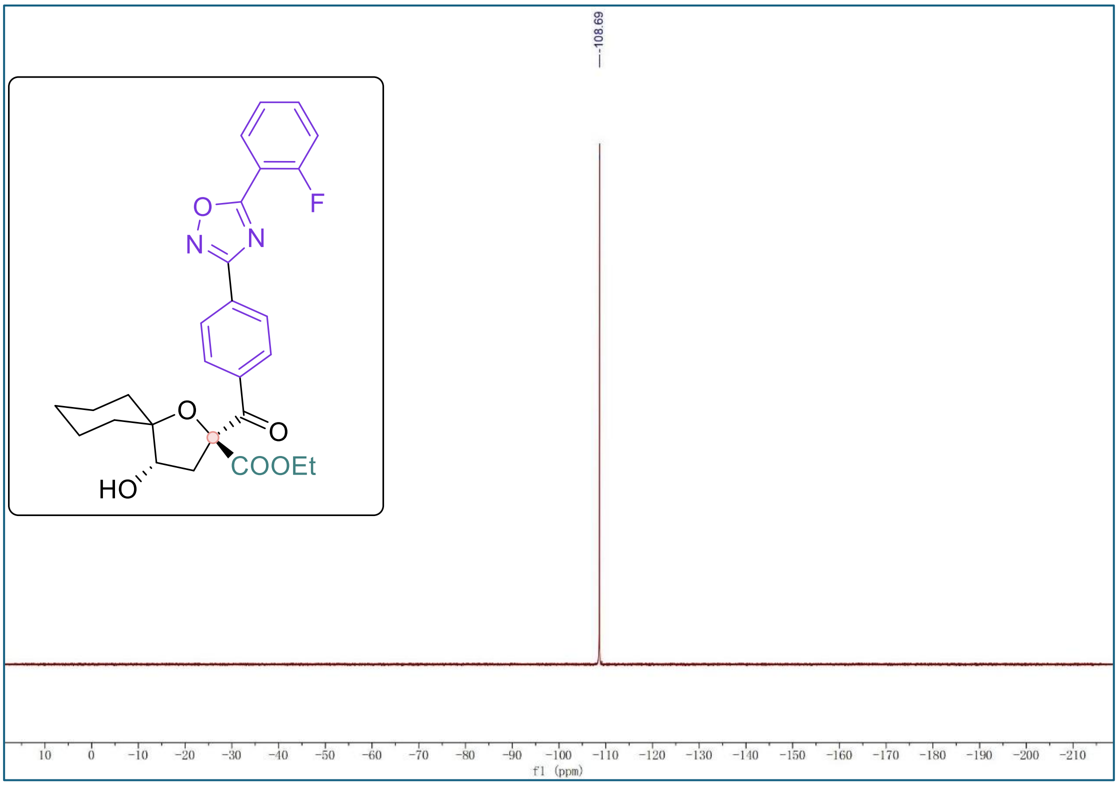

^{19}F NMR-spectrum (377 MHz, Chloroform-*d*) of **62**

ethyl 4-hydroxy-5,5-dimethyl-2-(3-methyl-4-oxo-2-phenyl-4H-chromene-6-carbonyl)tetrahydrofuran-2-carboxylate (63)

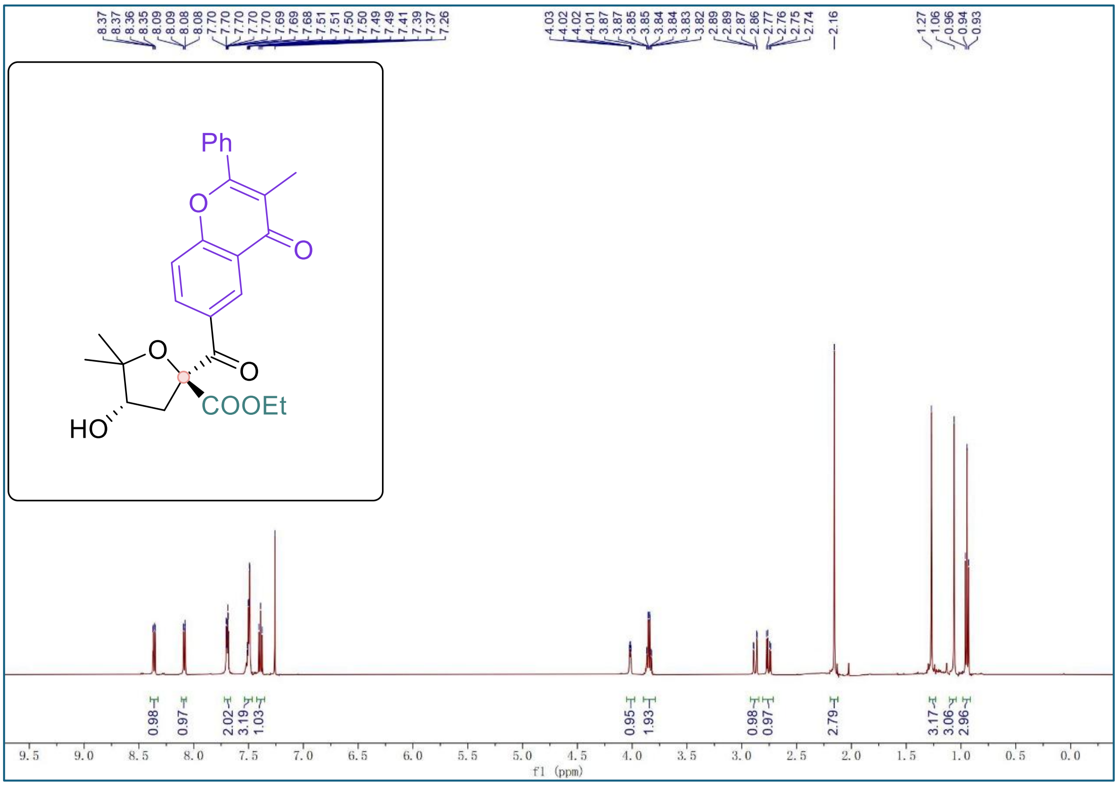

^{1}H NMR-spectrum (500 MHz, Chloroform-*d*) of **63**

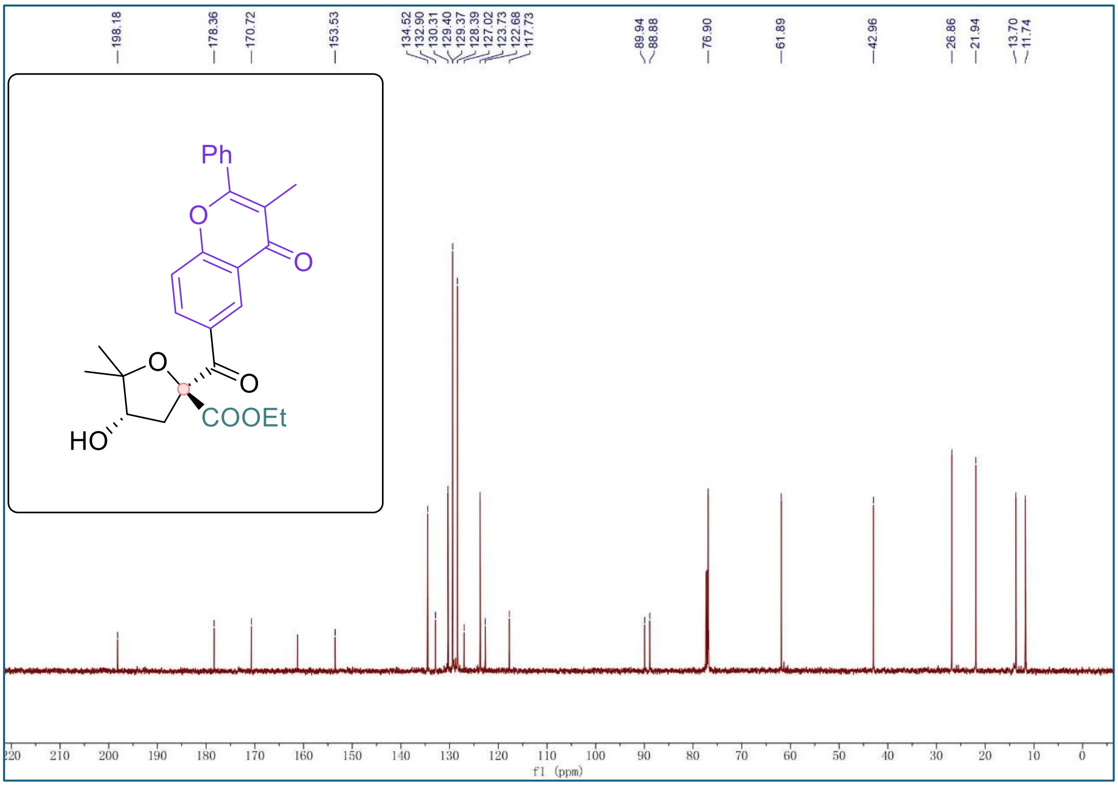

^{13}C NMR-spectrum (126 MHz, Chloroform-*d*) of **63**

ethyl 2-(4-(*N,N*-dipropylsulfamoyl)benzoyl)-4-hydroxy-5,5-dimethyltetrahydrofuran-2-carboxylate (64)

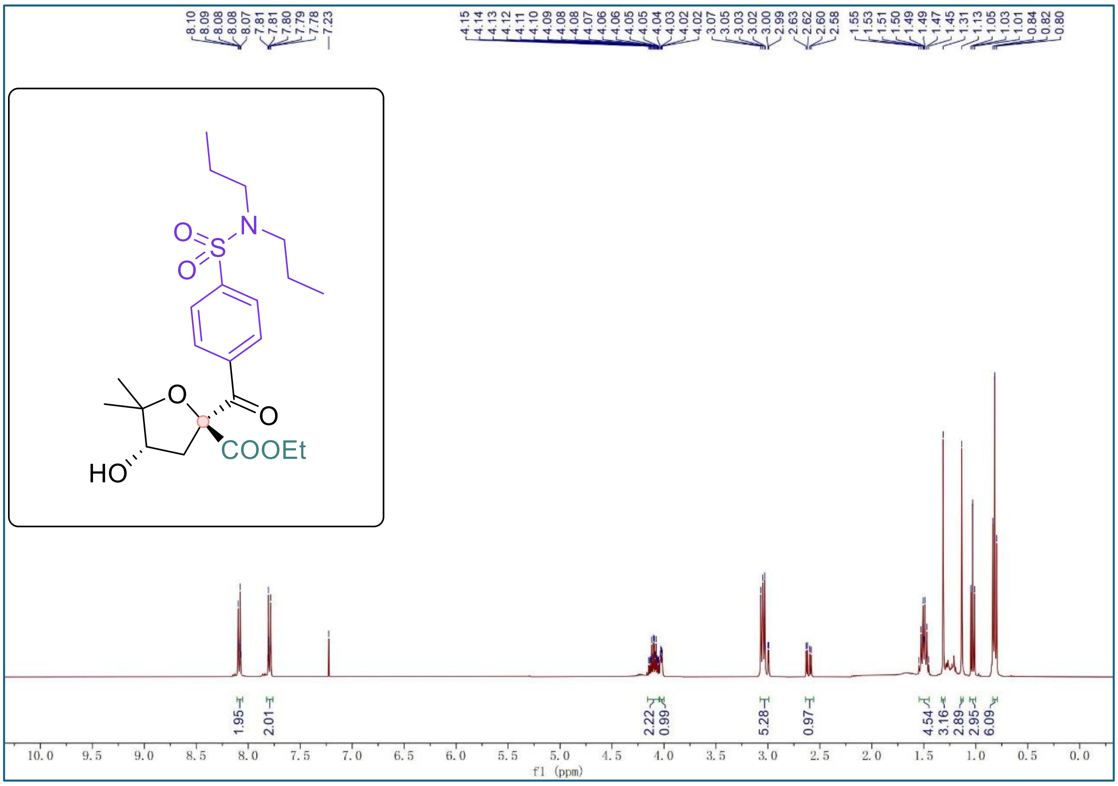

^{1}H NMR-spectrum (400 MHz, Chloroform-*d*) of **64**

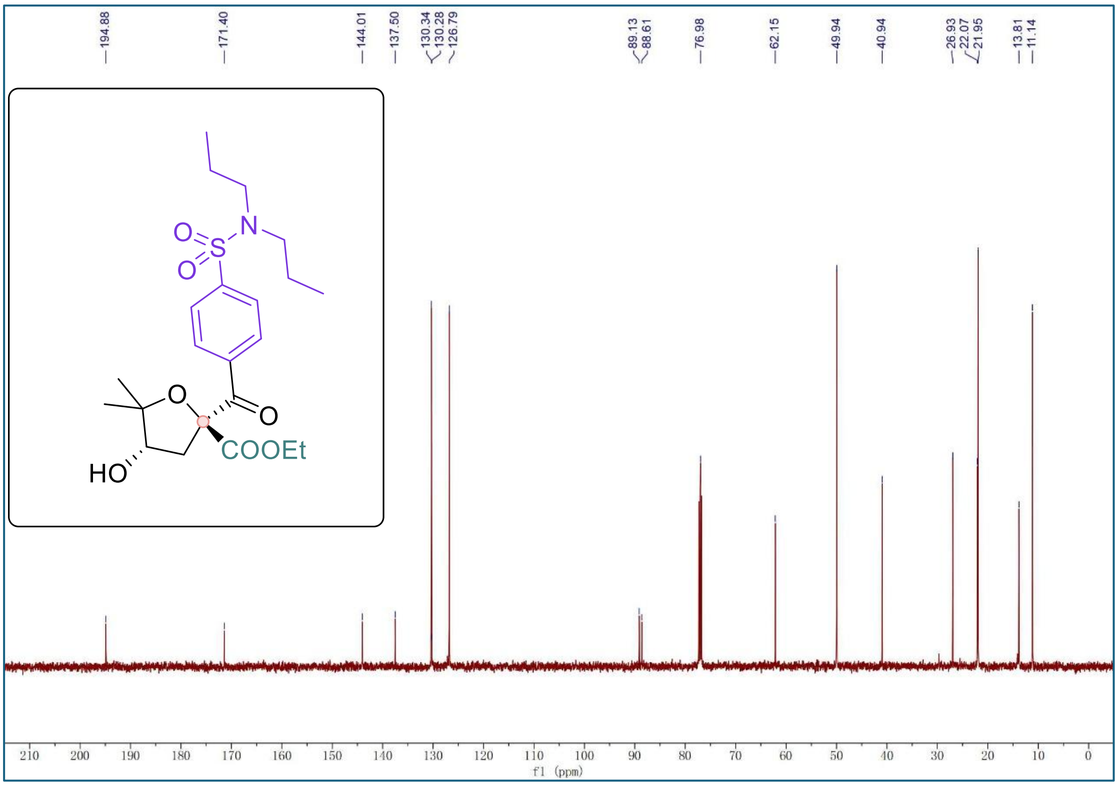

^{13}C NMR-spectrum (126 MHz, Chloroform-*d*) of **64**

ethyl 2-(2-(3-cyano-4-isobutoxyphenyl)-4-methylthiazole-5-carbonyl)-4-hydroxy-5,5-dimethyltetrahydrofuran-2-carboxylate (65)

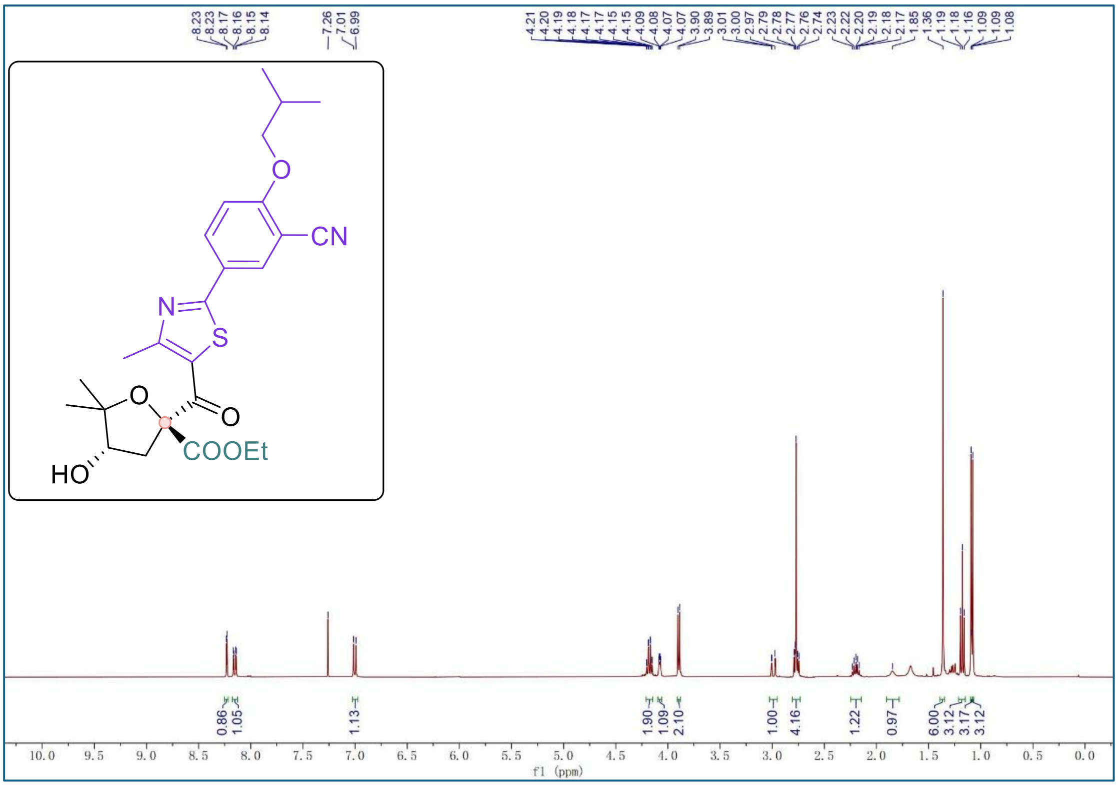

^{1}H NMR-spectrum (400 MHz, Chloroform-*d*) of **65**

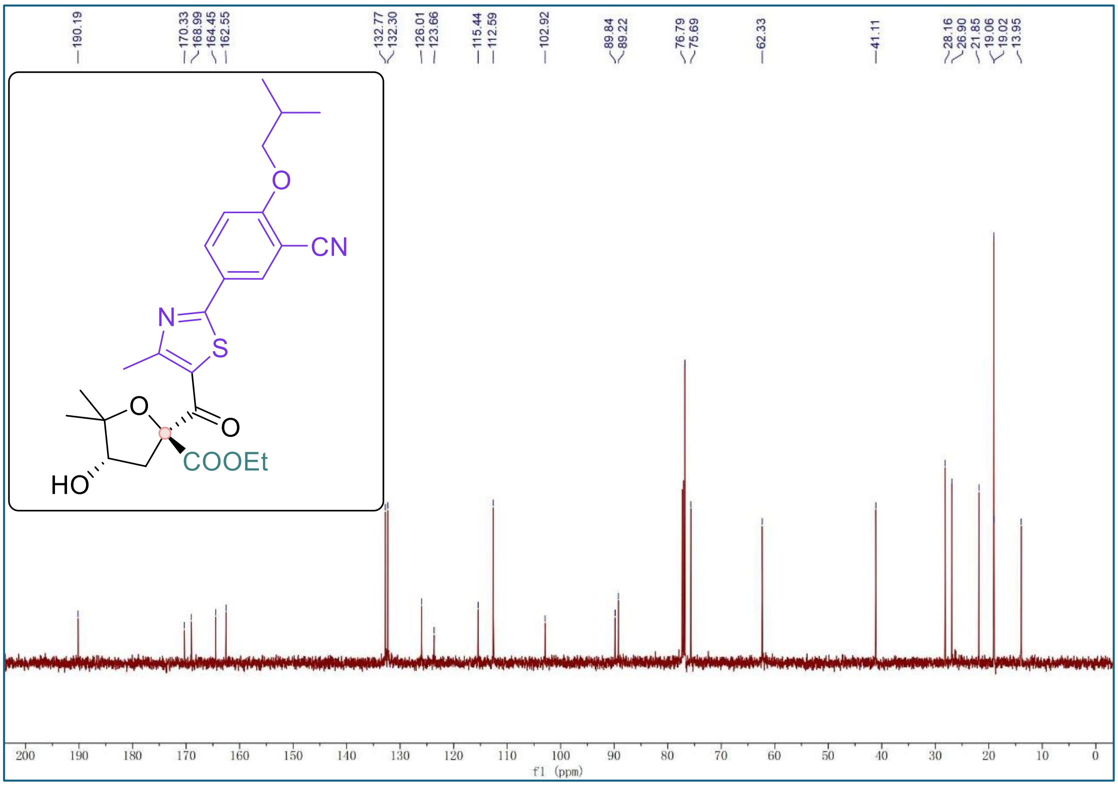

^{13}C NMR-spectrum (126 MHz, Chloroform-*d*) of **65**

ethyl 4-hydroxy-2-(4-methylbenzoyl)-1,9-dioxaspiro[5.5]undecane-2-carboxylate (66)

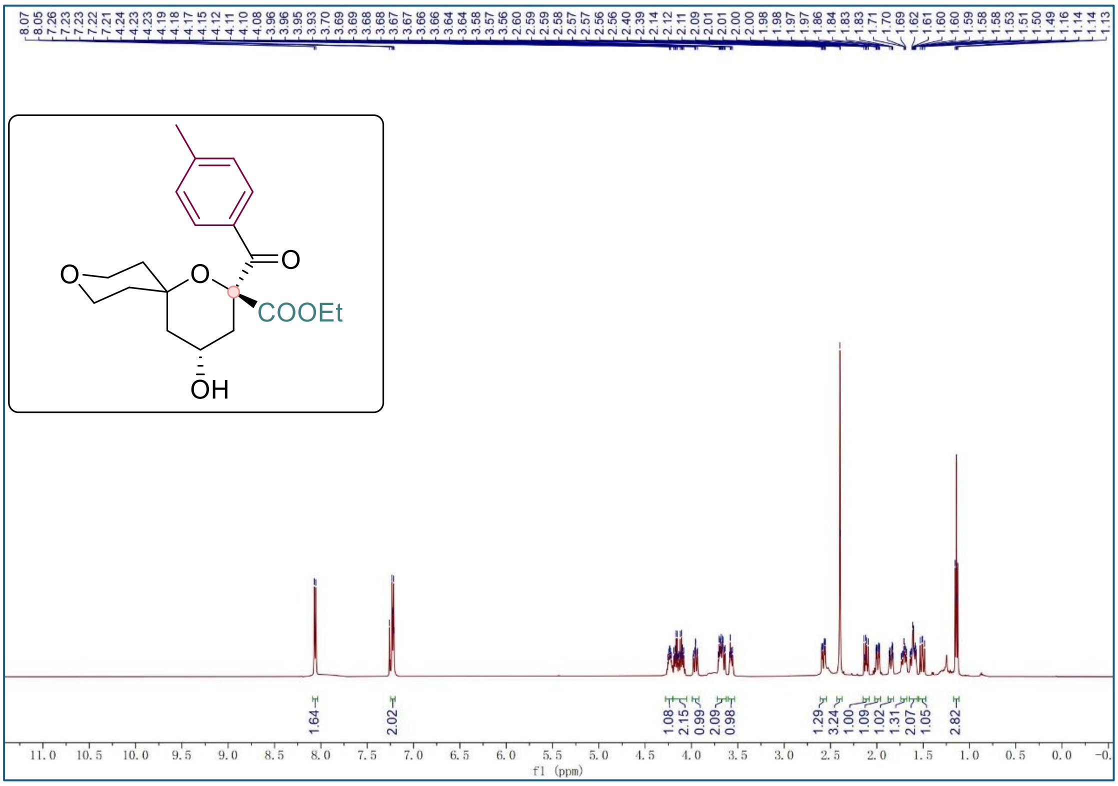

^{1}H NMR-spectrum (400 MHz, Chloroform-*d*) of **66**

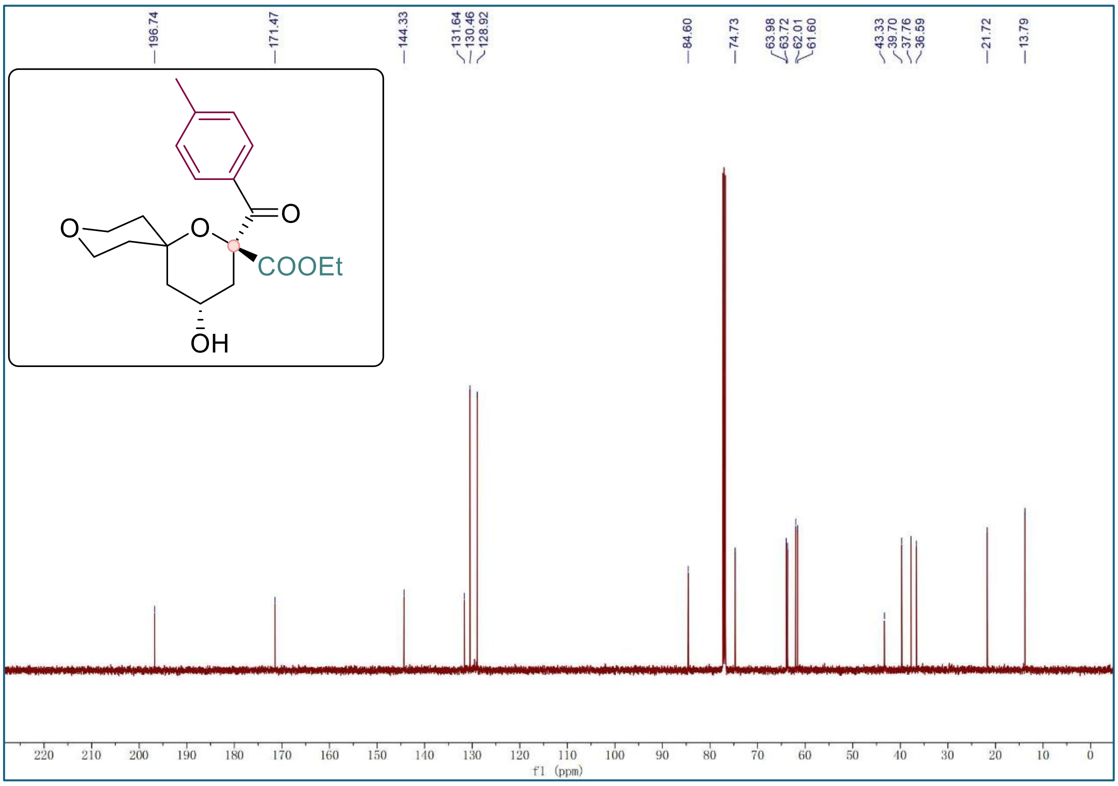

^{13}C NMR-spectrum (126 MHz, Chloroform-*d*) of **66**

SUPPORTING INFORMATION

ethyl 4-hydroxy-2-(4-(methoxycarbonyl)benzoyl)-1,9-dioxaspiro[5.5]undecane-2-carboxylate (67)

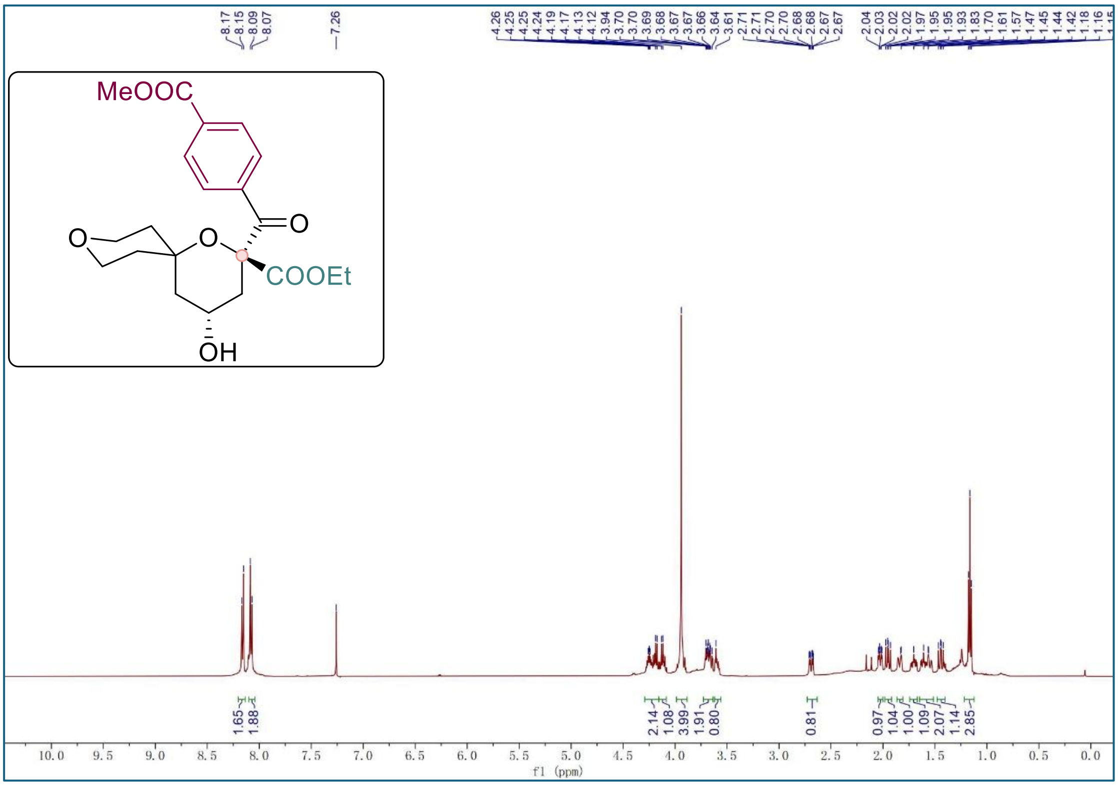

^{1}H NMR-spectrum (500 MHz, Chloroform-*d*) of **67**

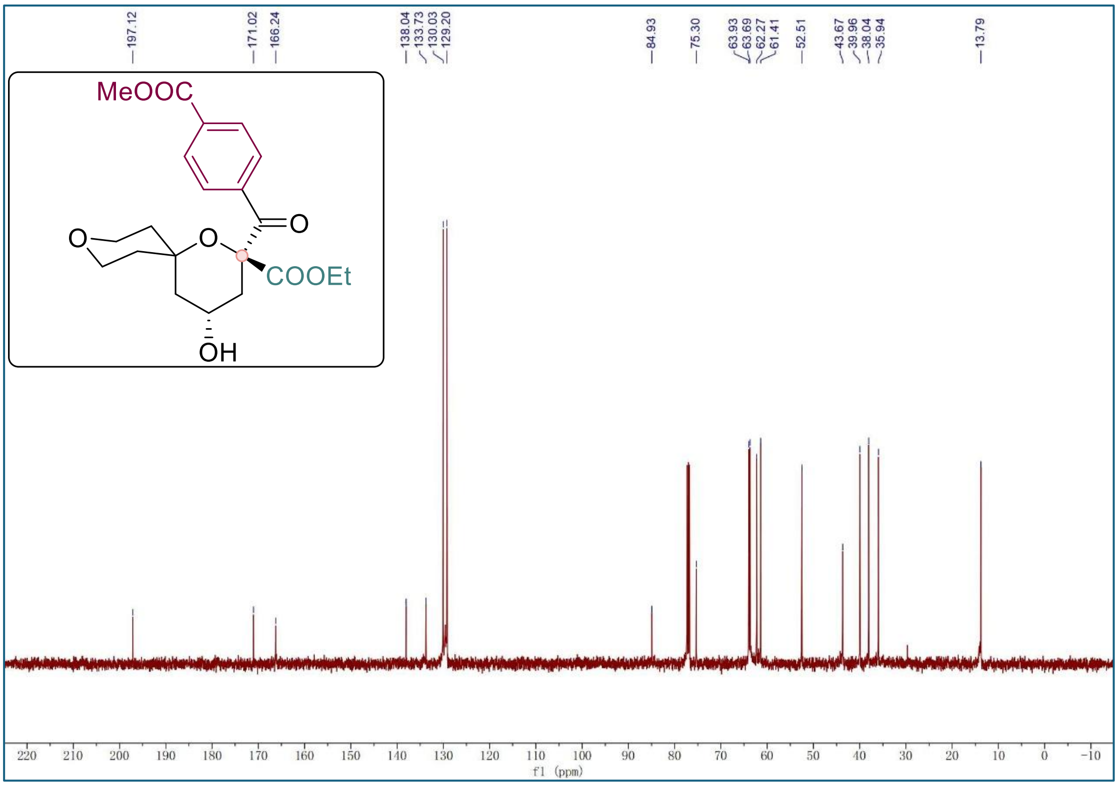

^{13}C NMR-spectrum (126 MHz, Chloroform-*d*) of **67**

ethyl 4-hydroxy-2-(4-nitrobenzoyl)-1,9-dioxaspiro[5.5]undecane-2-carboxylate (68)

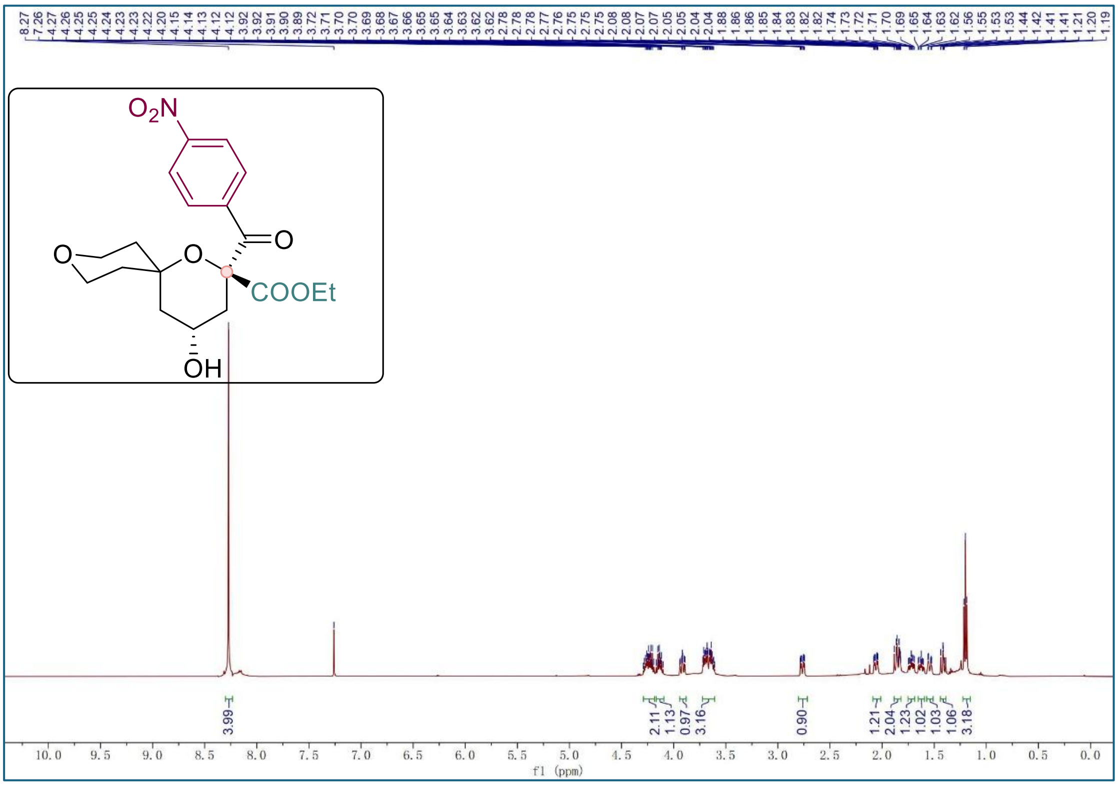

^{1}H NMR-spectrum (500 MHz, Chloroform-*d*) of **68**

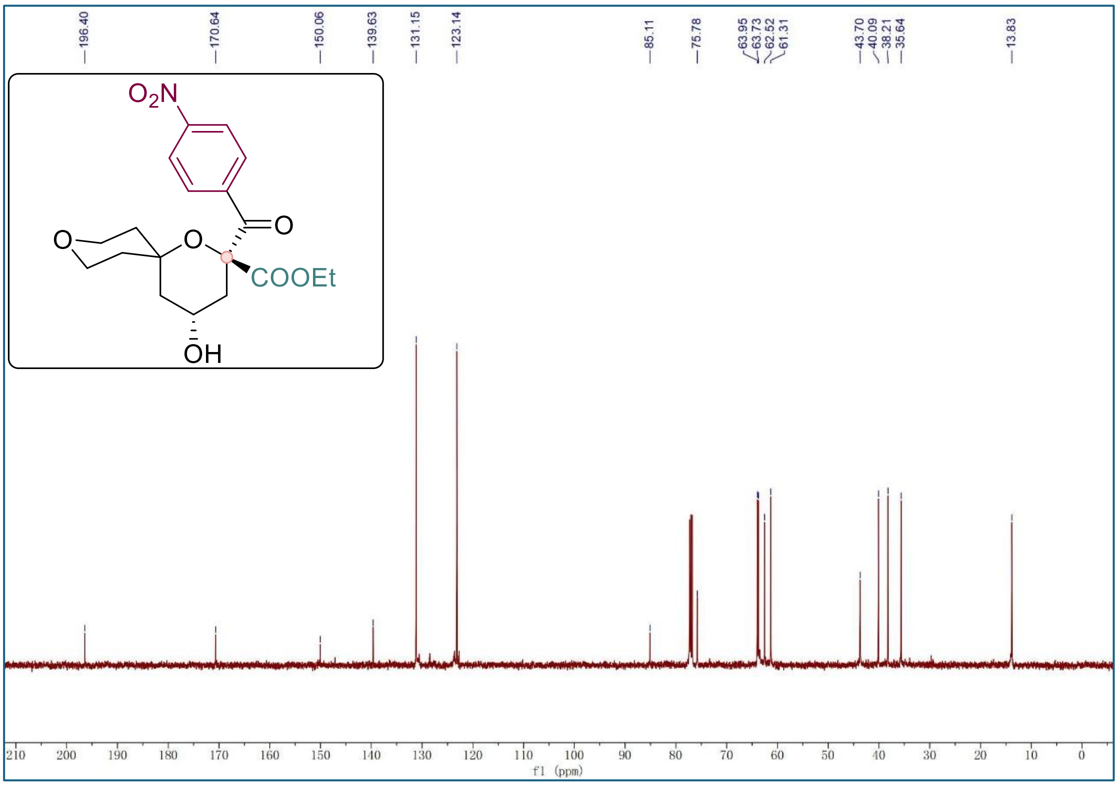

^{13}C NMR-spectrum (126 MHz, Chloroform-*d*) of **68**

ethyl 4-hydroxy-2-(3-methylbenzoyl)-1,9-dioxaspiro[5.5]undecane-2-carboxylate (69)

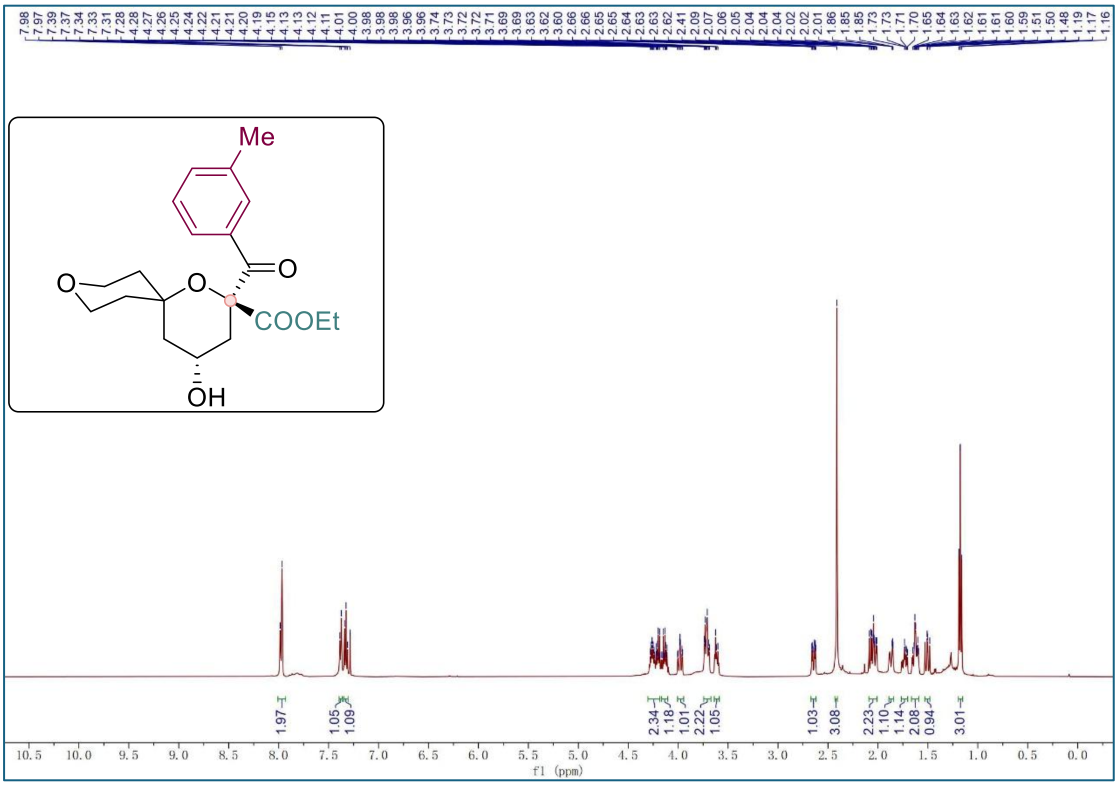

^{1}H NMR-spectrum (500 MHz, Chloroform-*d*) of **69**

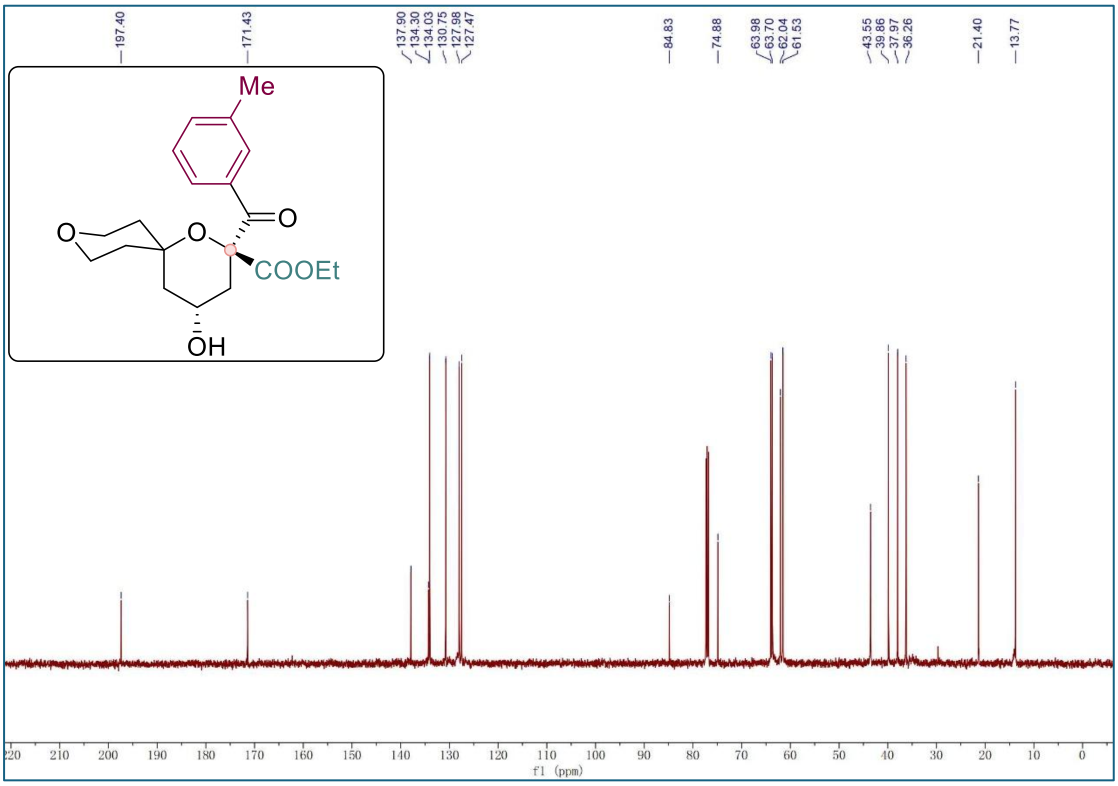

^{13}C NMR-spectrum (126 MHz, Chloroform-*d*) of **69**

ethyl 2-(3,5-difluorobenzoyl)-4-hydroxy-1,9-dioxaspiro[5.5]undecane-2-carboxylate (70)

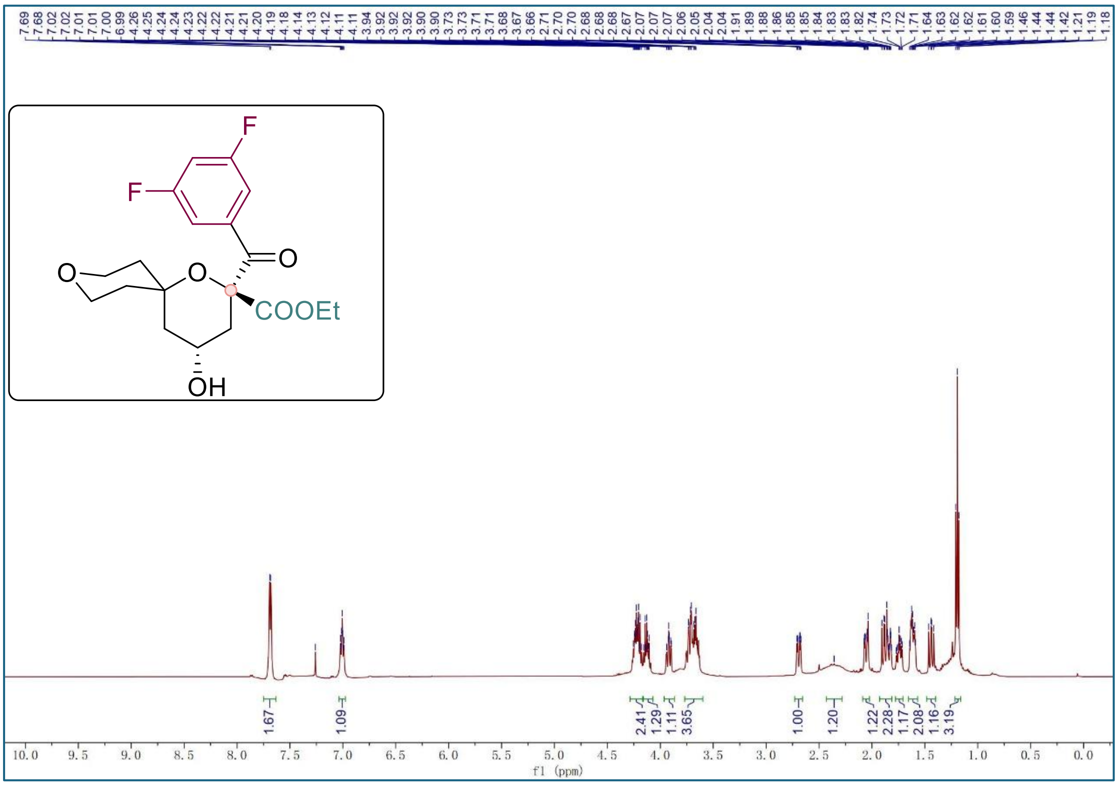

^{1}H NMR-spectrum (500 MHz, Chloroform-*d*) of **70**

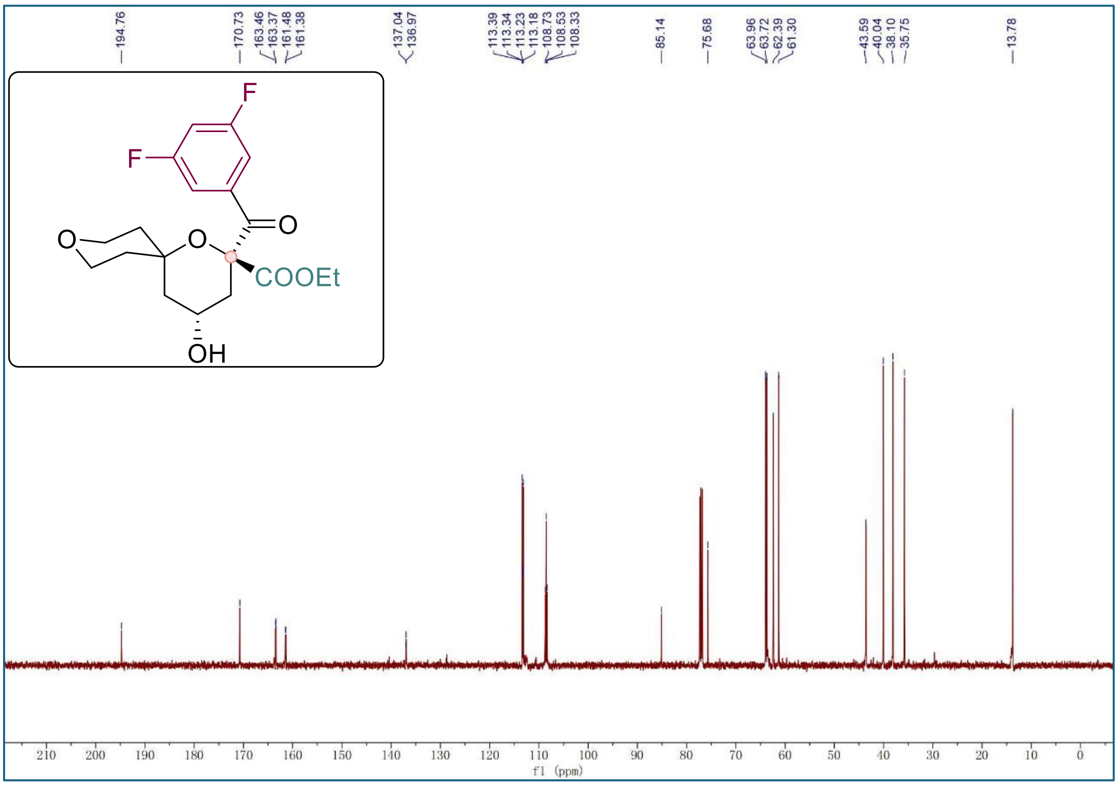

^{13}C NMR-spectrum (126 MHz, Chloroform-*d*) of **70**

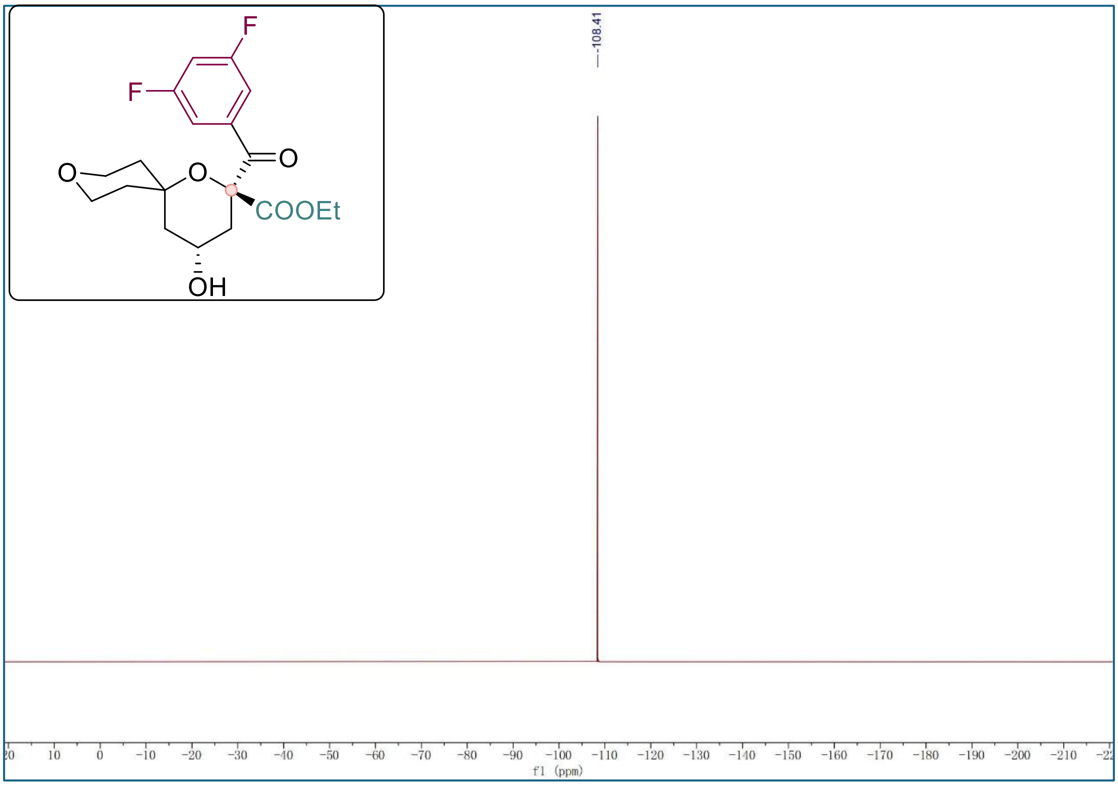

^{19}F NMR-spectrum (377 MHz, Chloroform-*d*) of **70**

phenyl 2-benzoyl-4-hydroxy-4-methyl-1,8-dioxaspiro[4.5]decane-2-carboxylate (71)

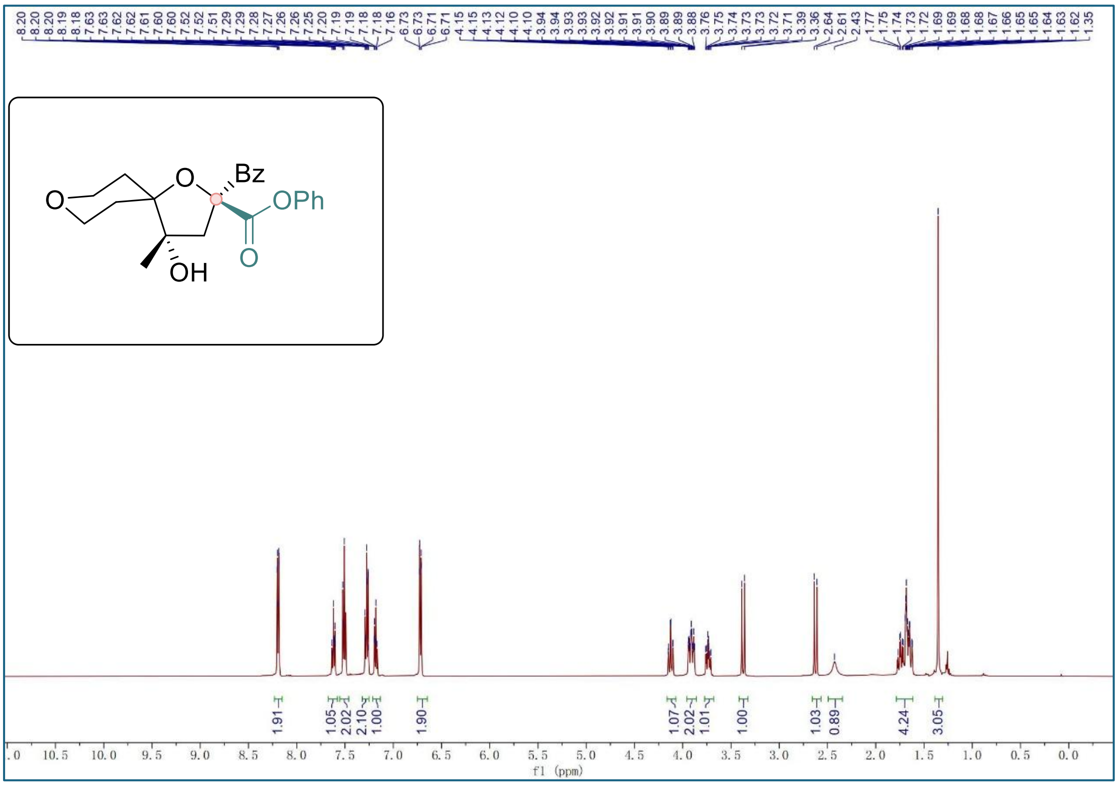

^{1}H NMR-spectrum (500 MHz, Chloroform-*d*) of **71**

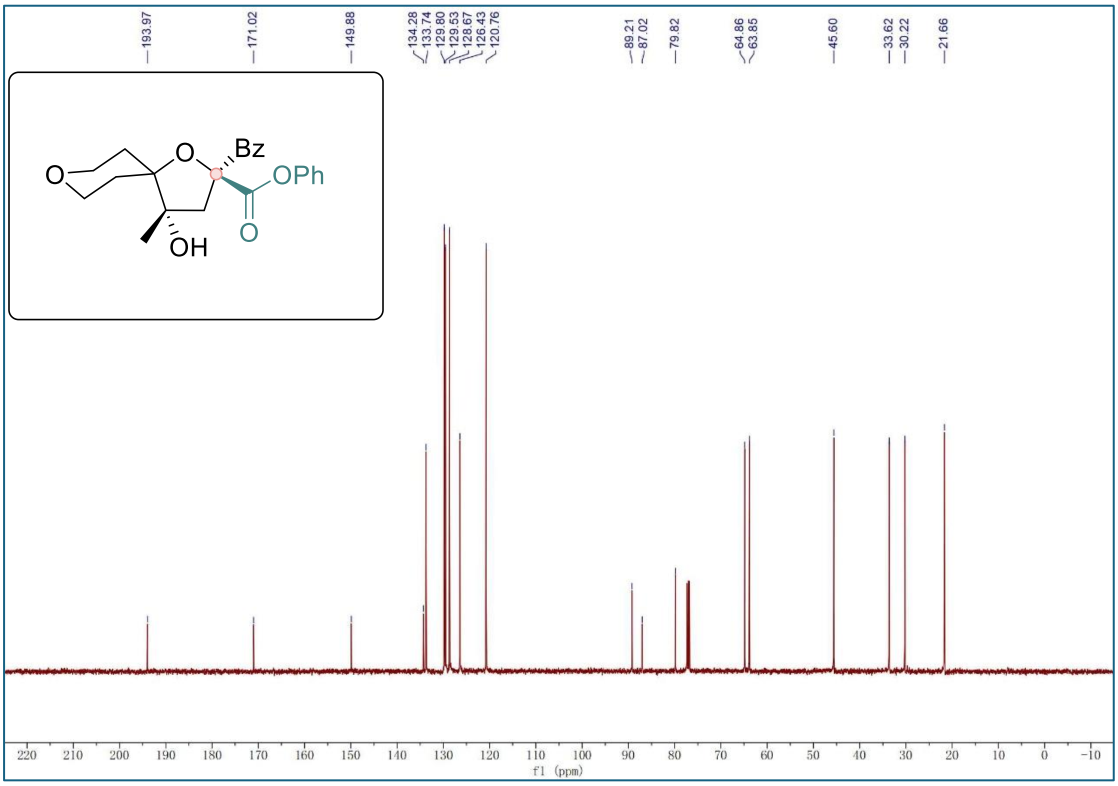

^{13}C NMR-spectrum (126 MHz, Chloroform-*d*) of **71**

(2S,5R)-2-isopropyl-5-methylcyclohexyl 2-benzoyl-4-hydroxy-4-methyl-1,8-dioxaspiro[4.5]decane-2-carboxylate (72)

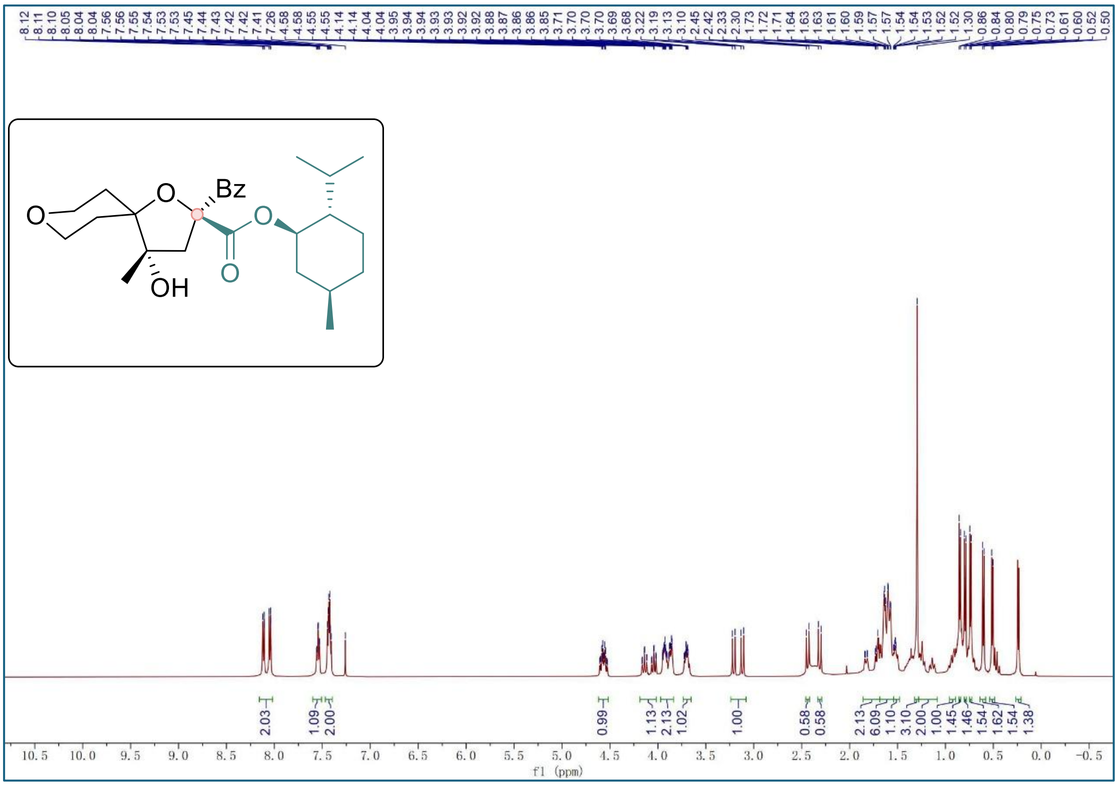

^{1}H NMR-spectrum (500 MHz, Chloroform-*d*) of **72**

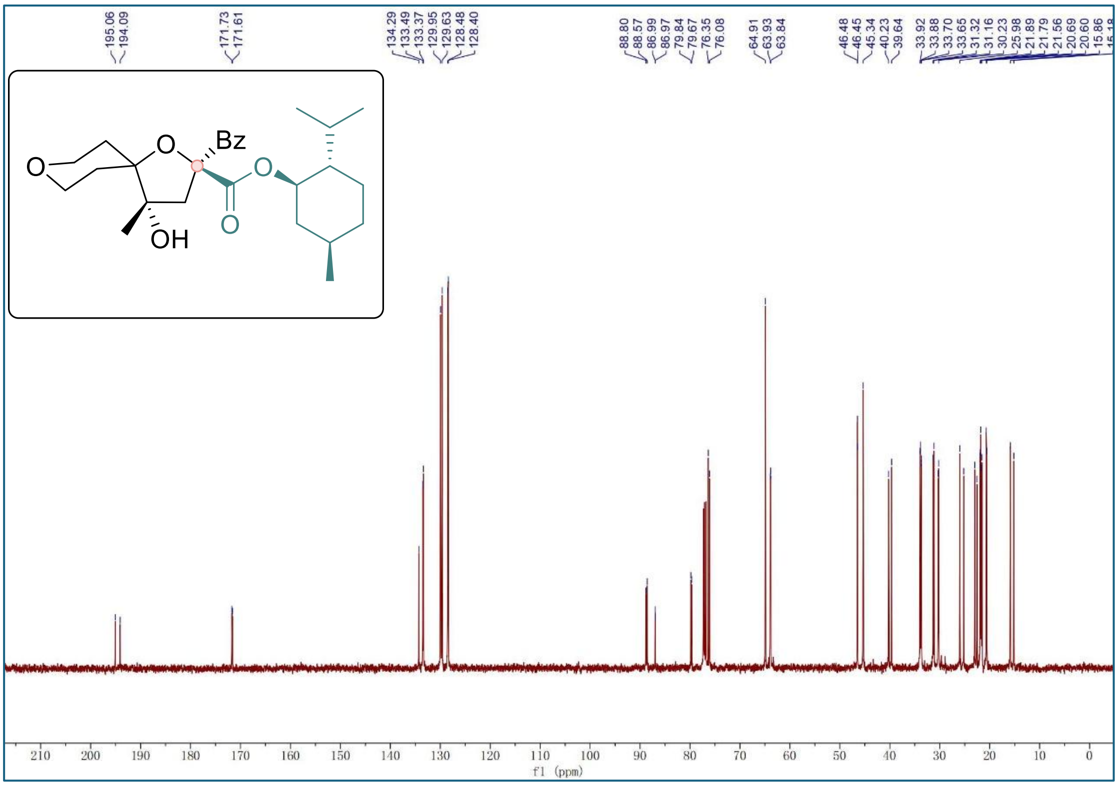

^{13}C NMR-spectrum (126 MHz, Chloroform-*d*) of **72**

tert-butyl 2-benzoyl-4-hydroxy-4-methyl-1,8-dioxaspiro[4.5]decane-2-carboxylate (73)

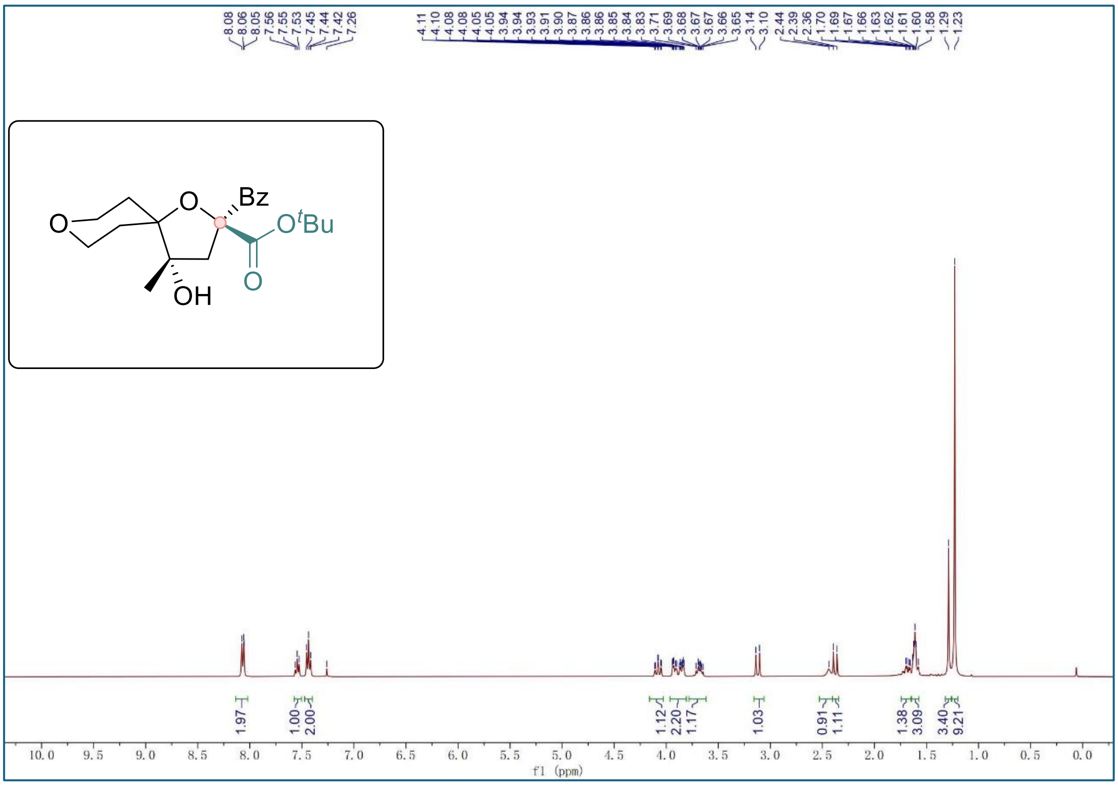

^{1}H NMR-spectrum (400 MHz, Chloroform-*d*) of **73**

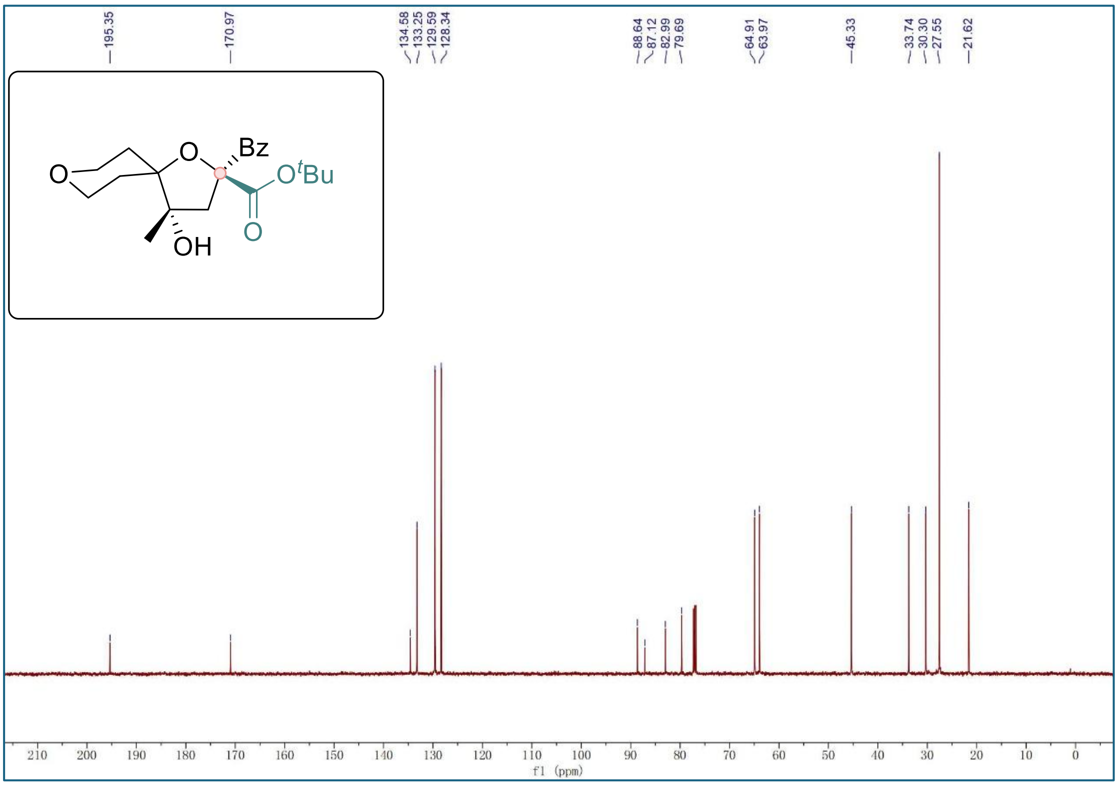

^{13}C NMR-spectrum (126 MHz, Chloroform-*d*) of **73**

SUPPORTING INFORMATION

(1R,2R,4R)-1,7,7-trimethylbicyclo[2.2.1]heptan-2-yl 2-benzoyl-4-hydroxy-4-methyl-1,8-dioxaspiro[4.5]decane-2-carboxylate (74)

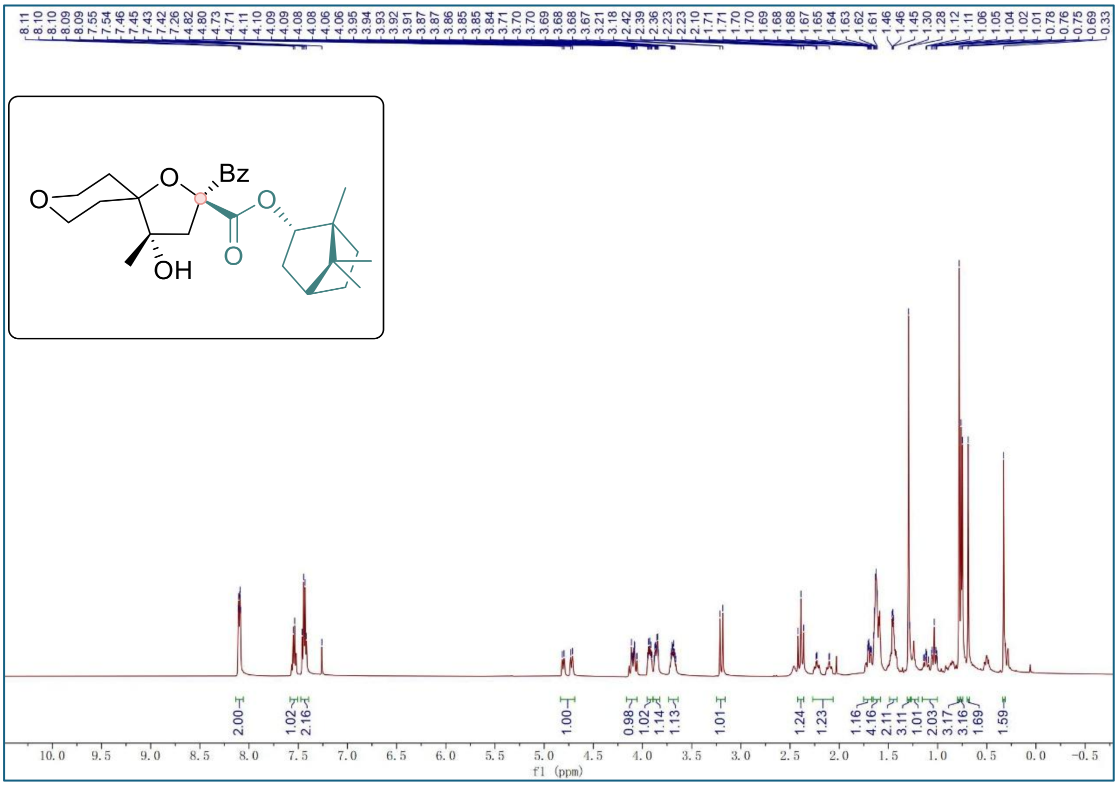

^{1}H NMR-spectrum (500 MHz, Chloroform-*d*) of **74**

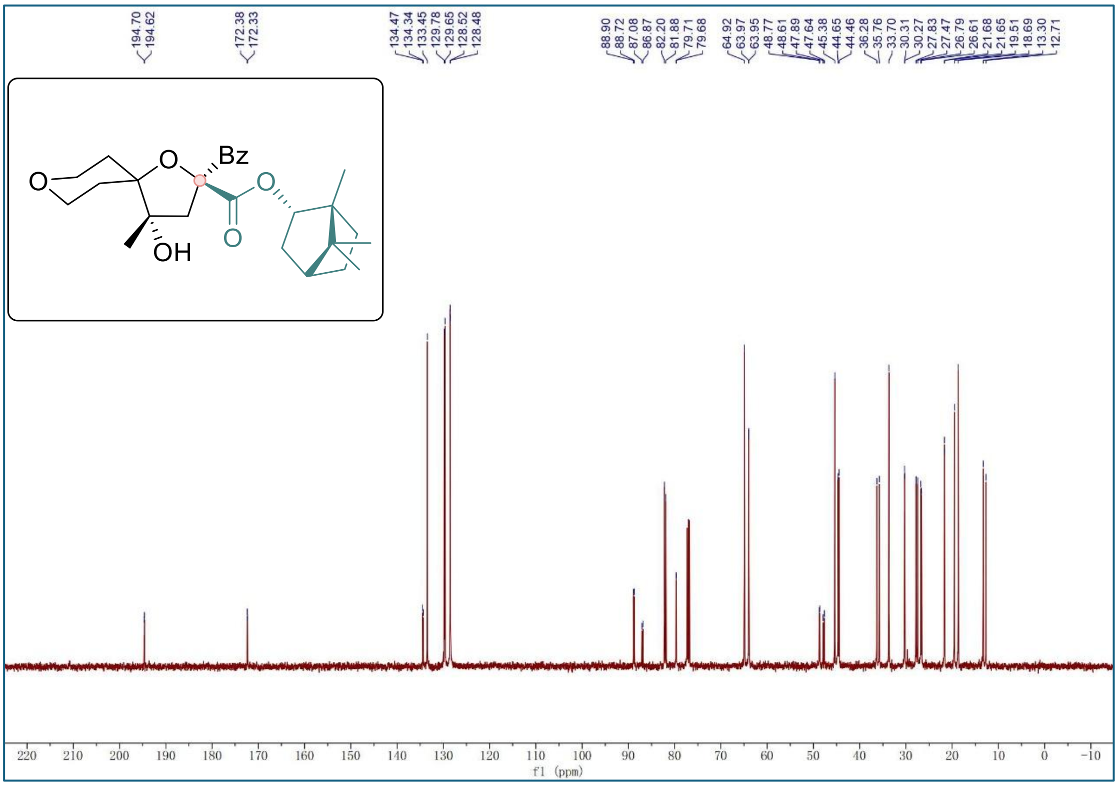

^{13}C NMR-spectrum (126 MHz, Chloroform-*d*) of **74**

tert-butyl 2-benzoyl-4-hydroxy-1-oxaspiro[4.5]decane-2-carboxylate (75)

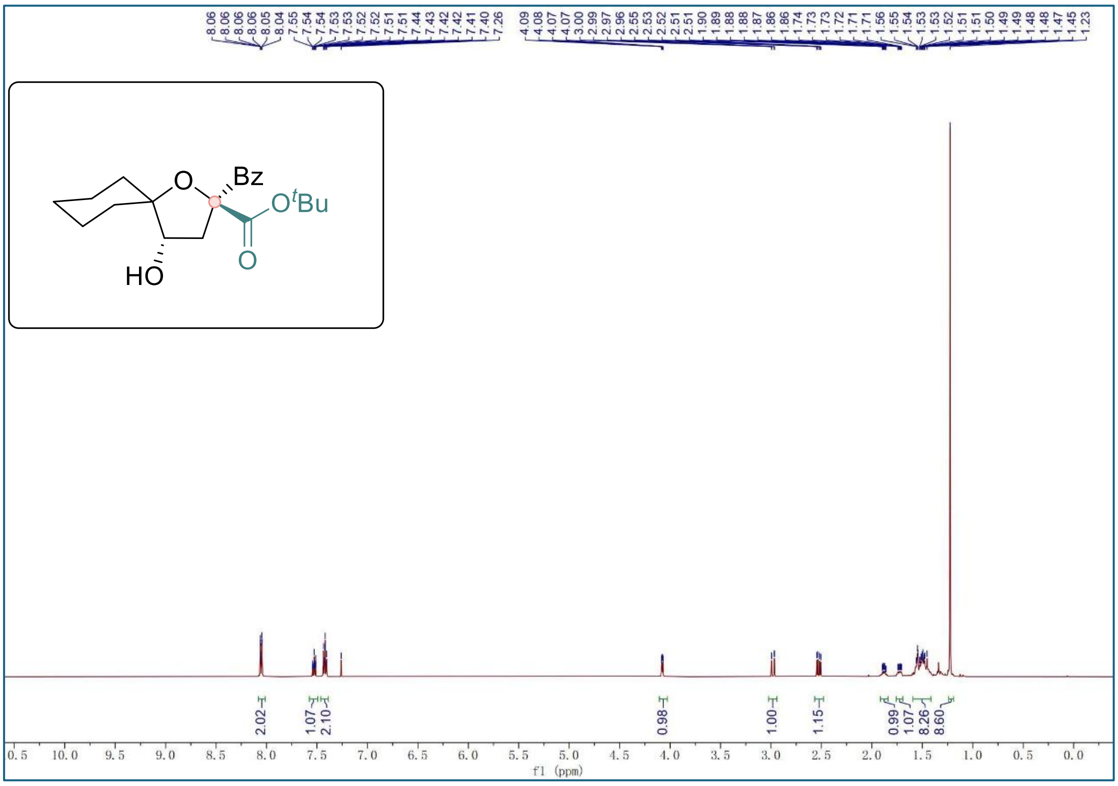

^{1}H NMR-spectrum (500 MHz, Chloroform-*d*) of **75**

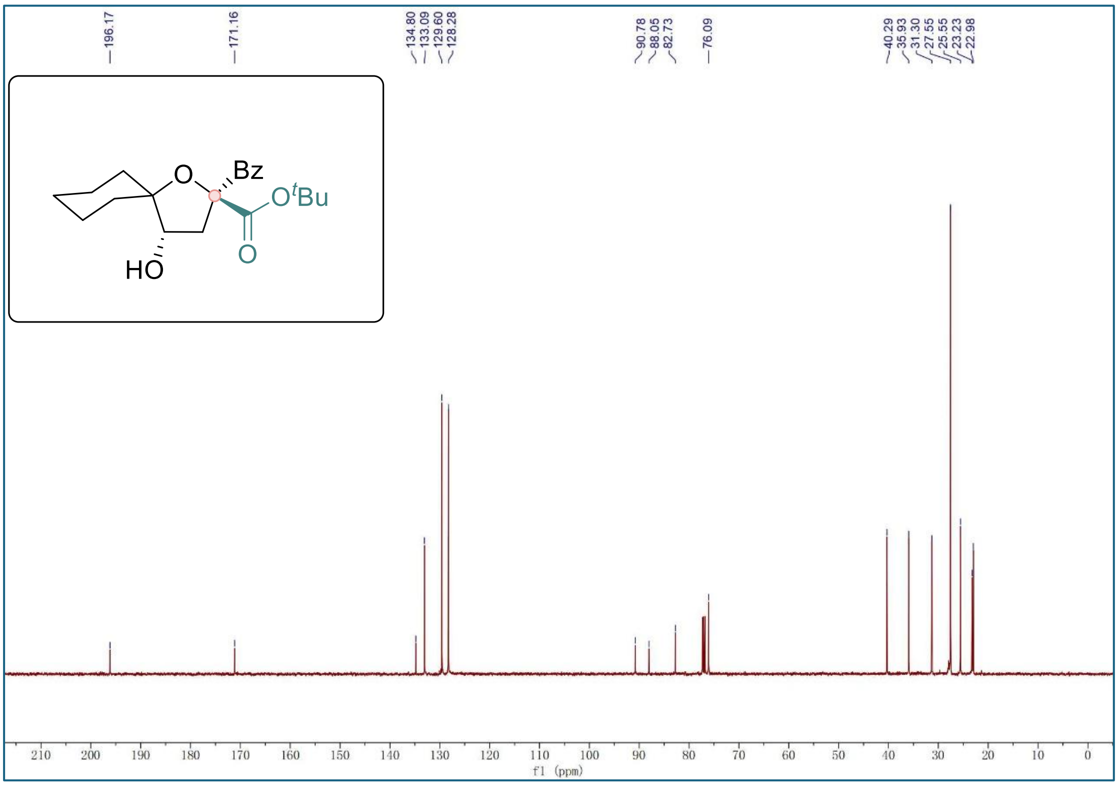

^{13}C NMR-spectrum (126 MHz, Chloroform-*d*) of **75**

benzyl 2-benzoyl-4-hydroxy-1-oxaspiro[4.5]decane-2-carboxylate (76)

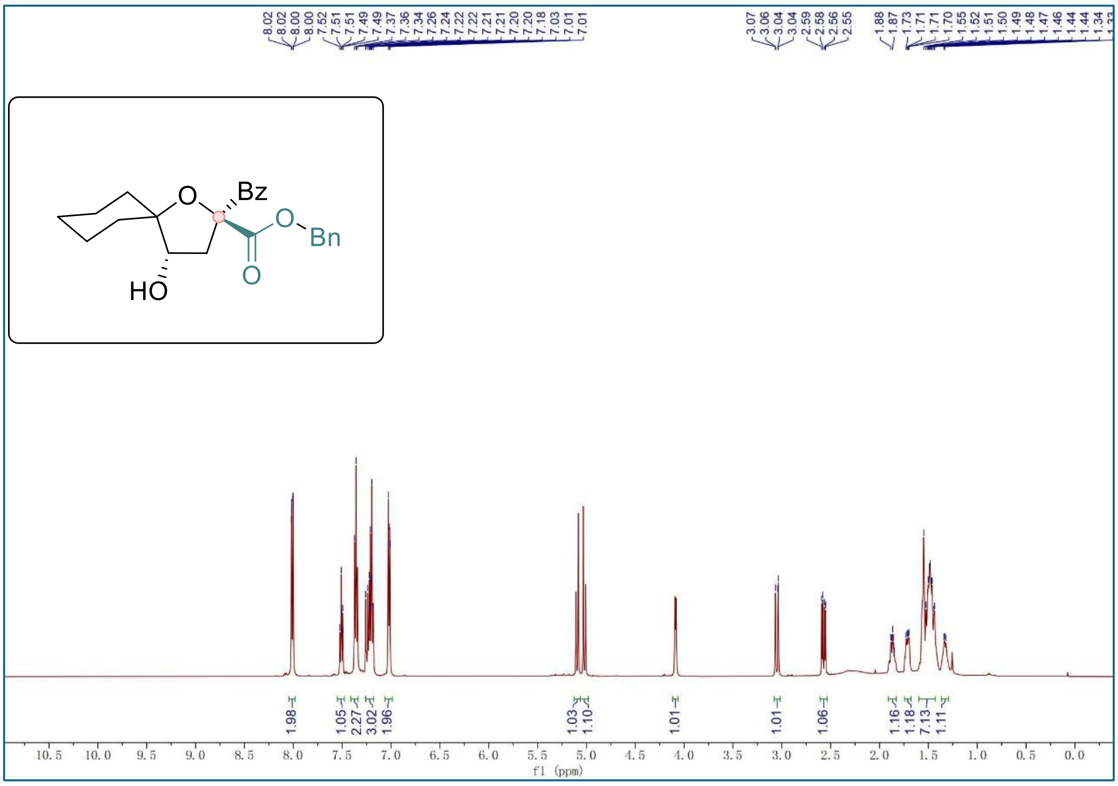

^{1}H NMR-spectrum (500 MHz, Chloroform-*d*) of **76**

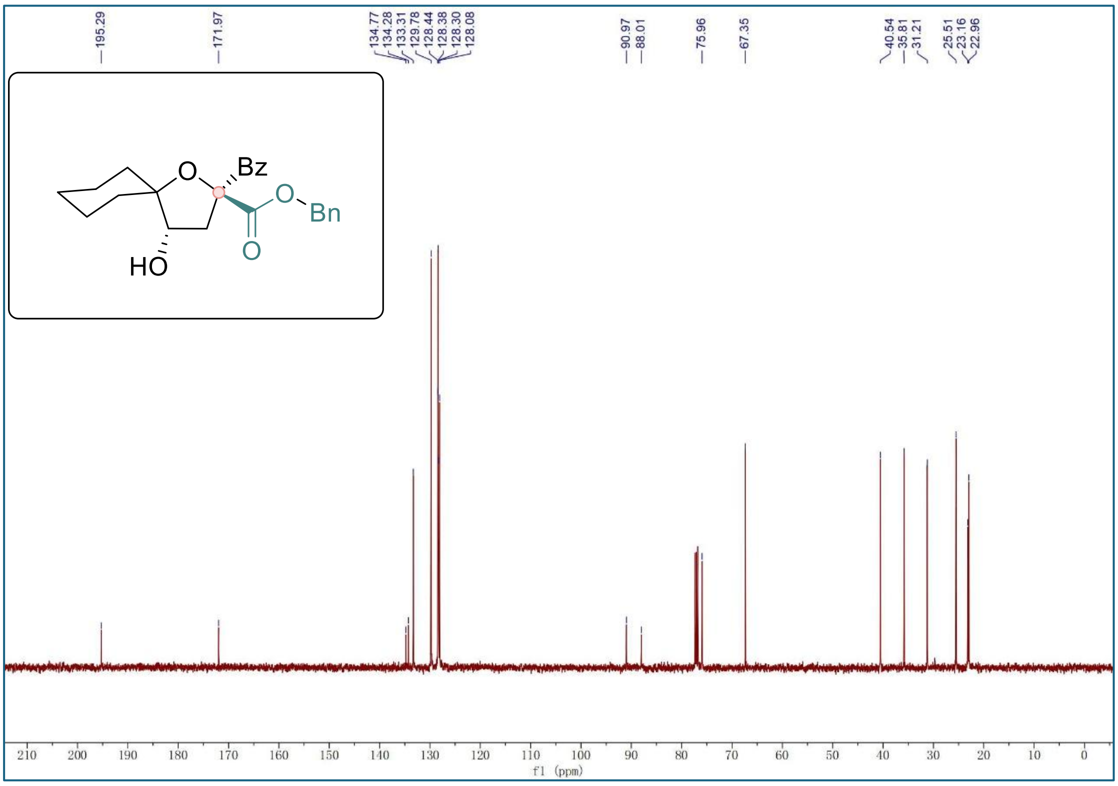

^{13}C NMR-spectrum (126 MHz, Chloroform-*d*) of **76**

benzyl 10-benzoyl-12-hydroxy-1,4,9-trioxadispiro[4.2.4^8.2^5]tetradecane-10-carboxylate (77)

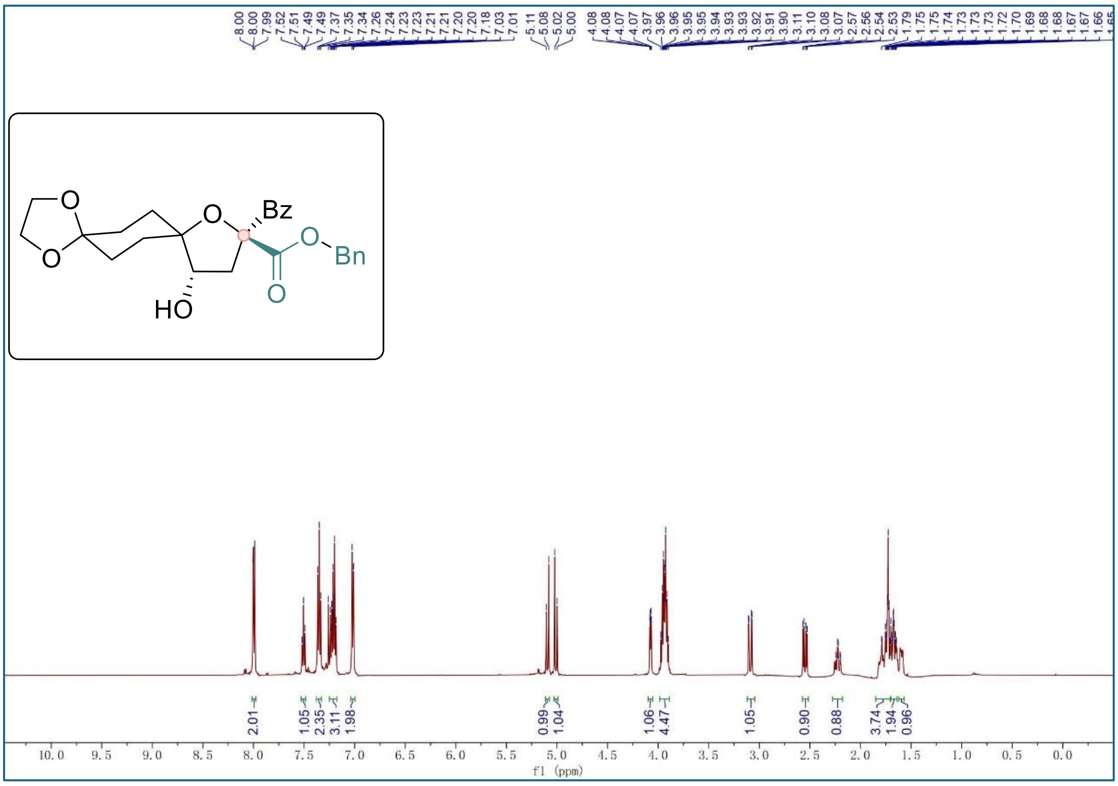

^{1}H NMR-spectrum (500 MHz, Chloroform-*d*) of **77**

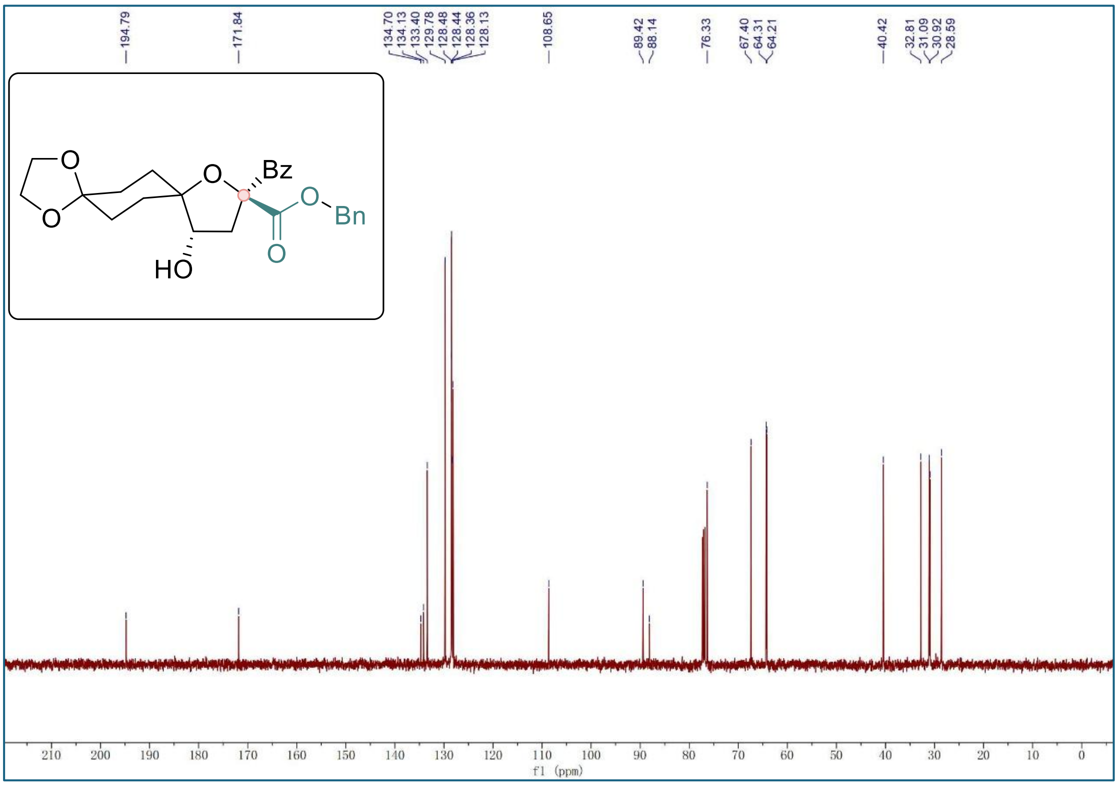

^{13}C NMR-spectrum (126 MHz, Chloroform-*d*) of **77**

SUPPORTING INFORMATION

(1R,4R)-1,7,7-trimethylbicyclo[2.2.1]heptan-2-yl 2-benzoyl-4-hydroxy-1-oxaspiro[4.5]decane-2-carboxylate (78)

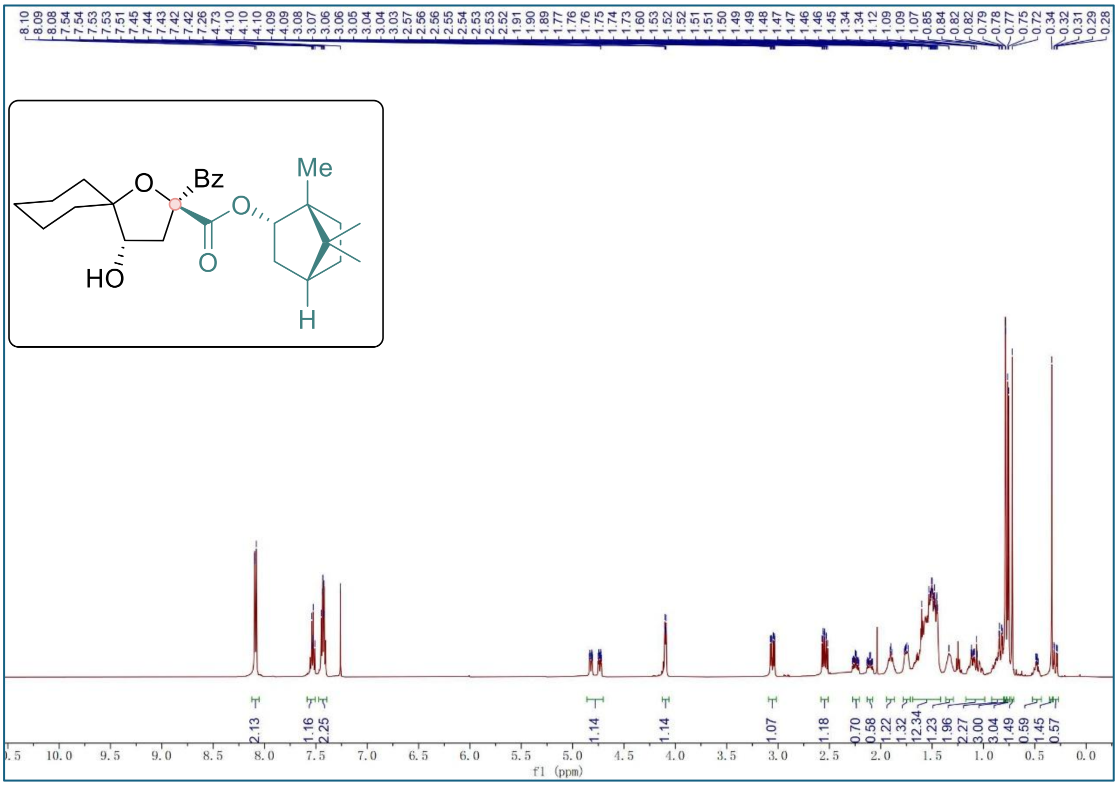

^{1}H NMR-spectrum (500 MHz, Chloroform-*d*) of **78**

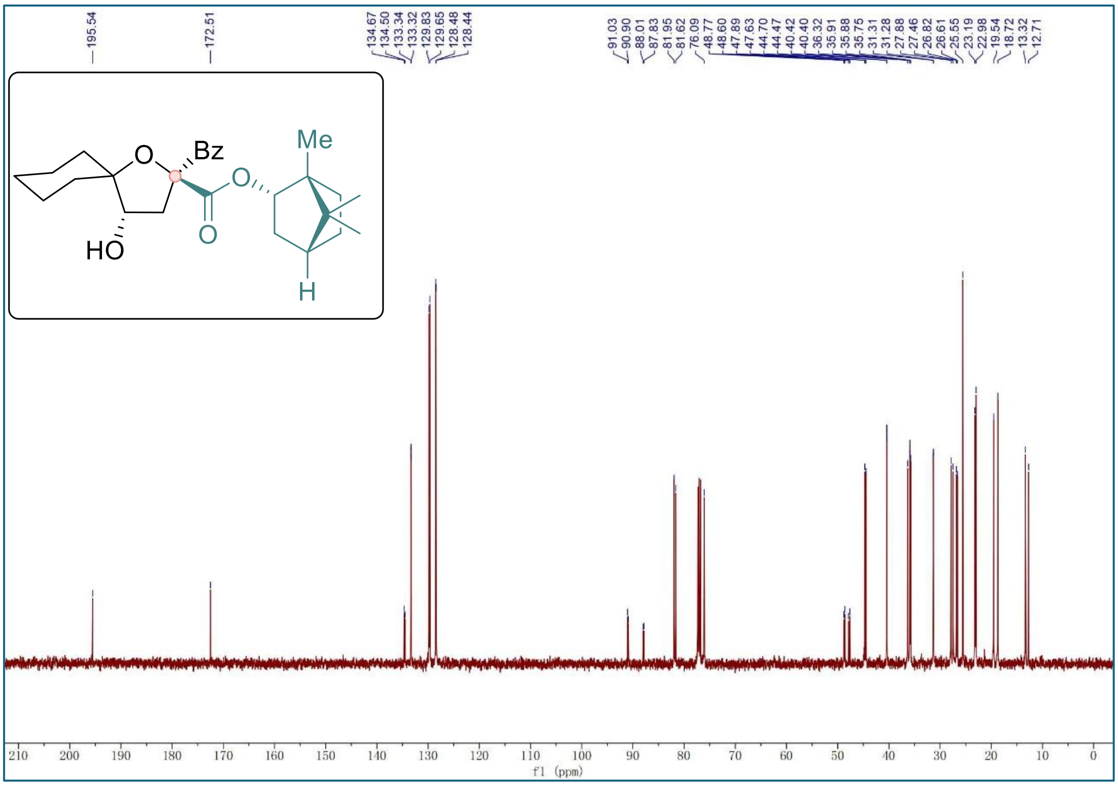

^{13}C NMR-spectrum (126 MHz, Chloroform-*d*) of **78**

SUPPORTING INFORMATION

1-(2-benzoyl-4-hydroxy-4-methyl-1,8-dioxaspiro[4.5]decan-2-yl)ethan-1-one (79)

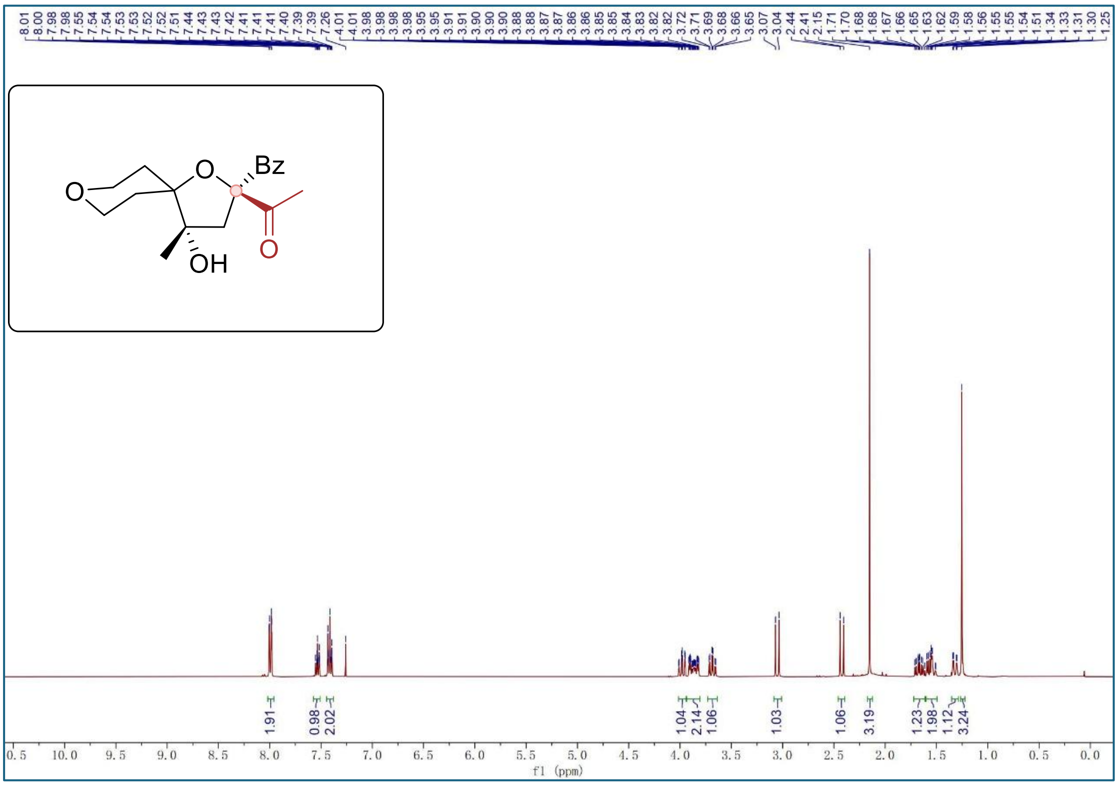

^{1}H NMR-spectrum (400 MHz, Chloroform-*d*) of **79**

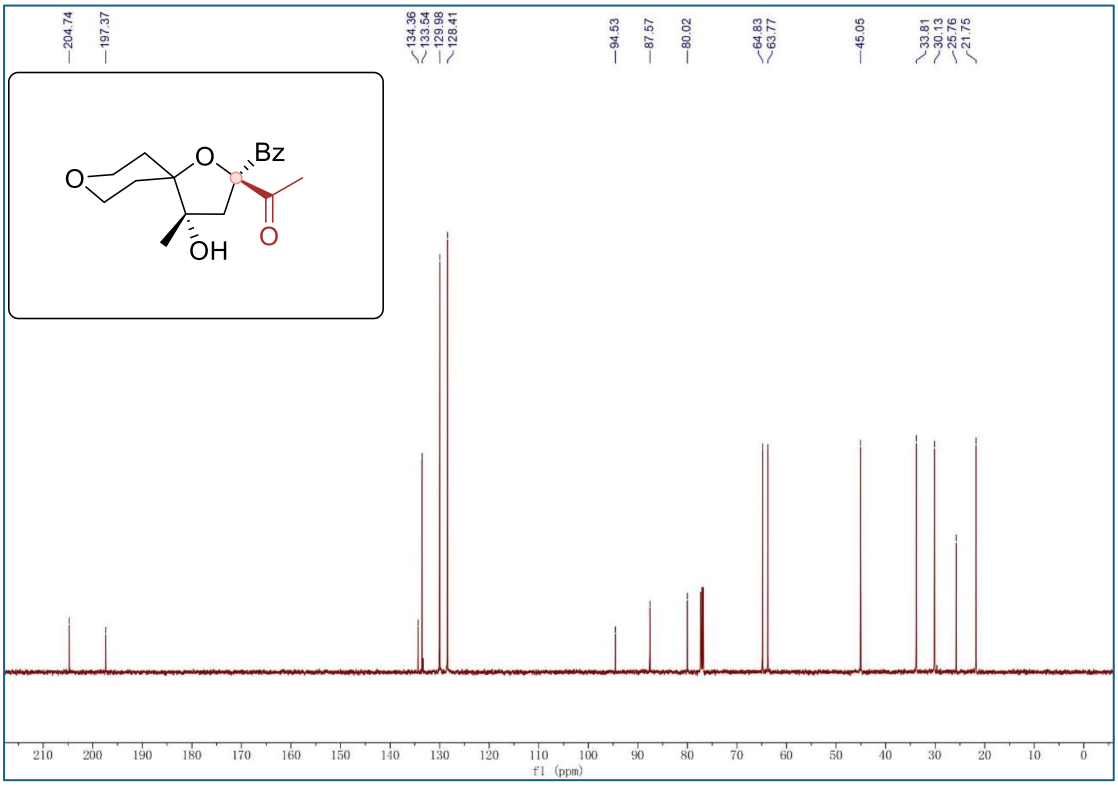

^{13}C NMR-spectrum (126 MHz, Chloroform-*d*) of **79**

(4-hydroxy-4-methyl-1,8-dioxaspiro[4.5]decane-2,2-diyl)bis(phenylmethanone) (80)

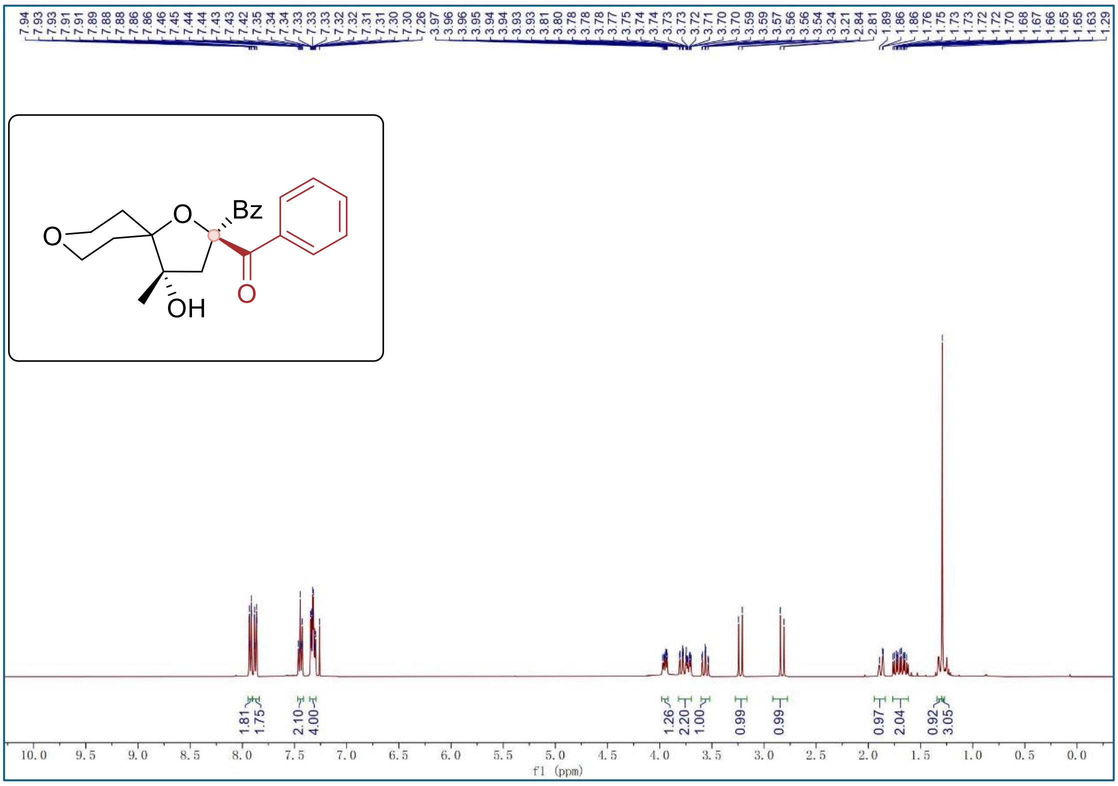

^{1}H NMR-spectrum (400 MHz, Chloroform-*d*) of **80**

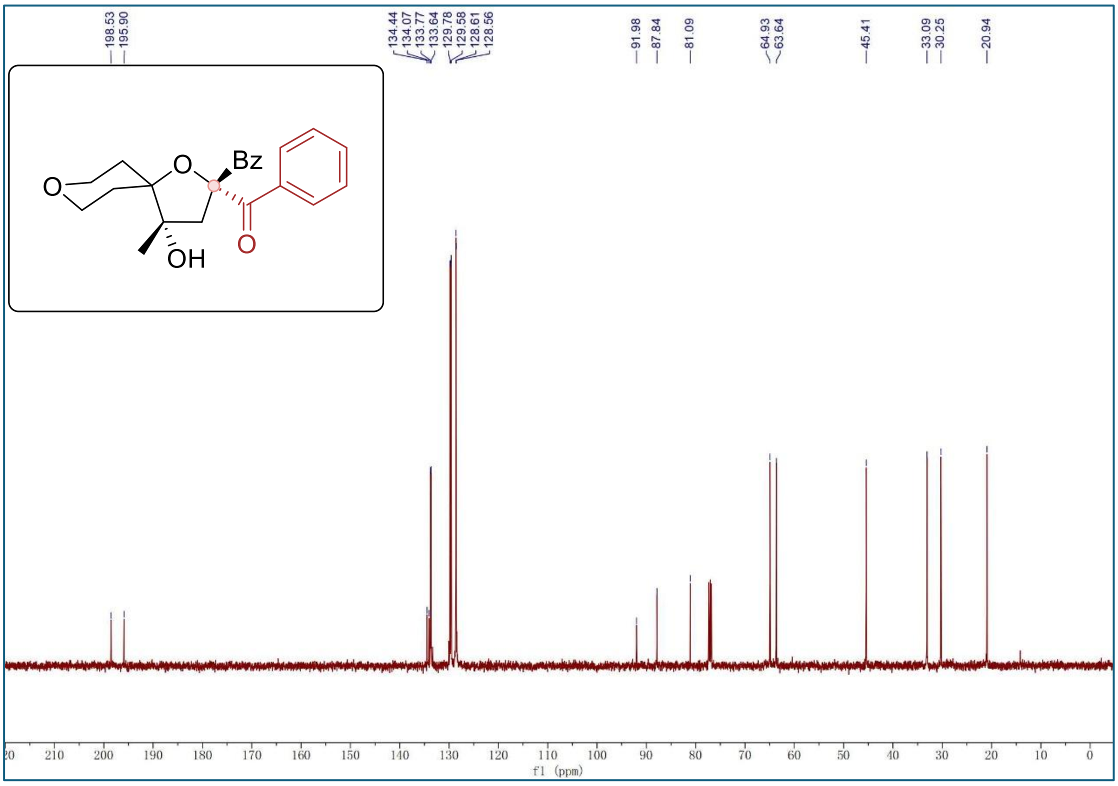

^{13}C NMR-spectrum (126 MHz, Chloroform-*d*) of **80**

1-(2-benzoyl-4-hydroxy-4-phenyl-1,8-dioxaspiro[4.5]decan-2-yl)ethan-1-one (81)

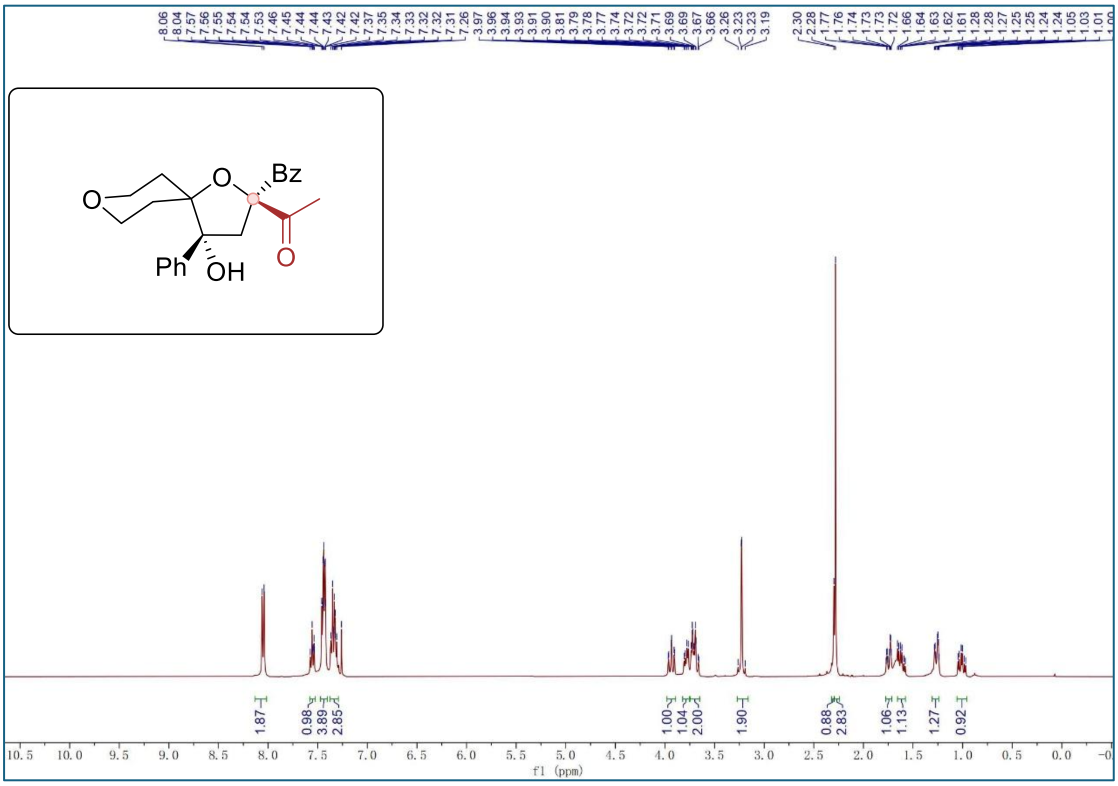

^{1}H NMR-spectrum (400 MHz, Chloroform-*d*) of **81**

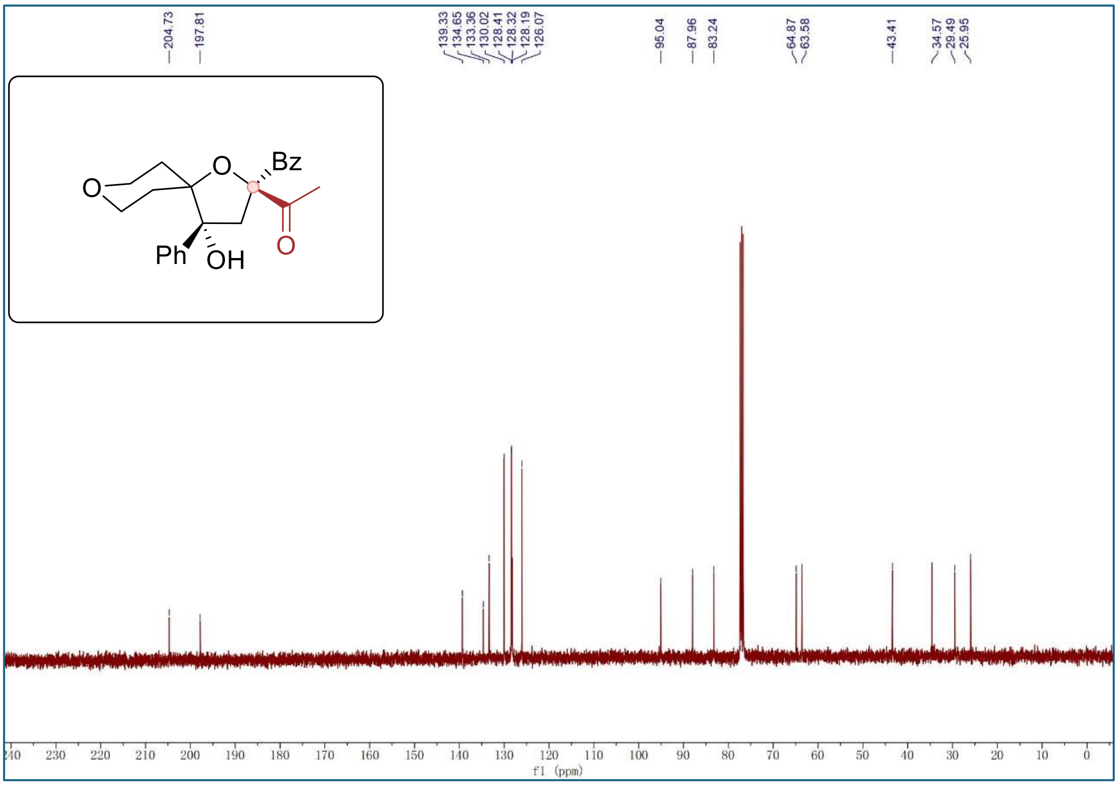

^{13}C NMR-spectrum (101 MHz, Chloroform-*d*) of **81**

SUPPORTING INFORMATION

dimethyl (2-benzoyl-4-hydroxy-4-phenyl-1-oxaspiro[4.6]undecan-2-yl)phosphonate (82)

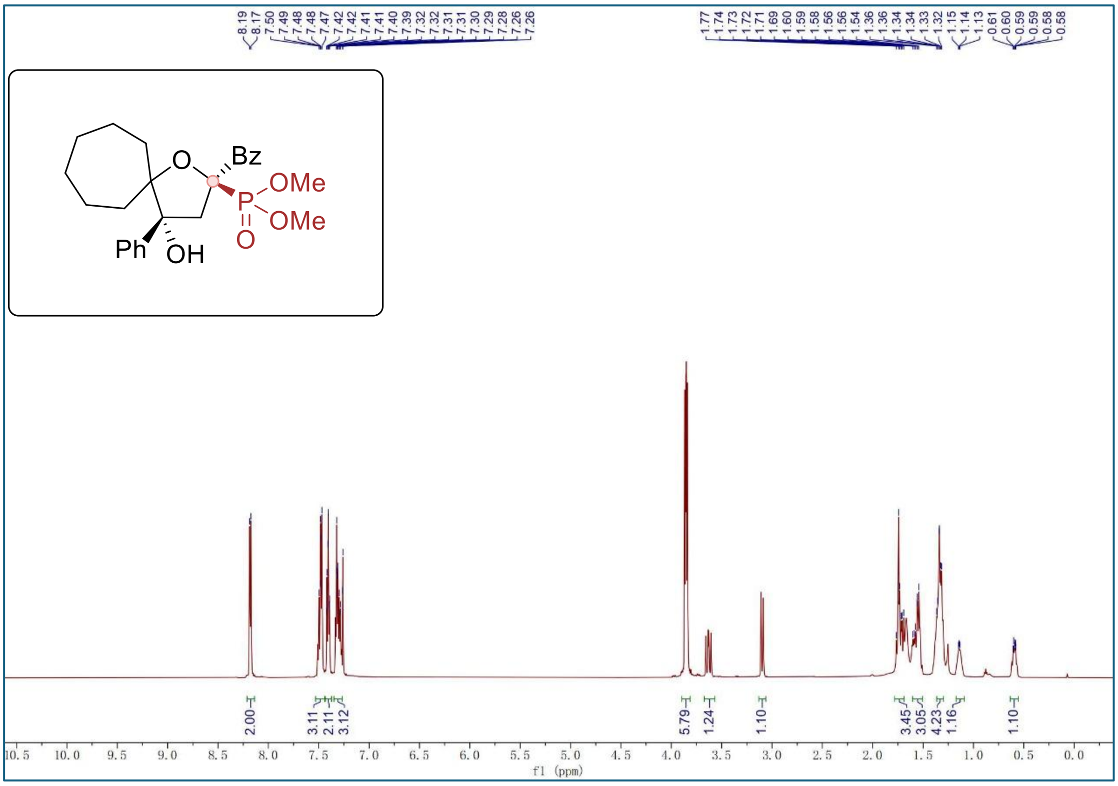

^{1}H NMR-spectrum (600 MHz, Chloroform-*d*) of **82**

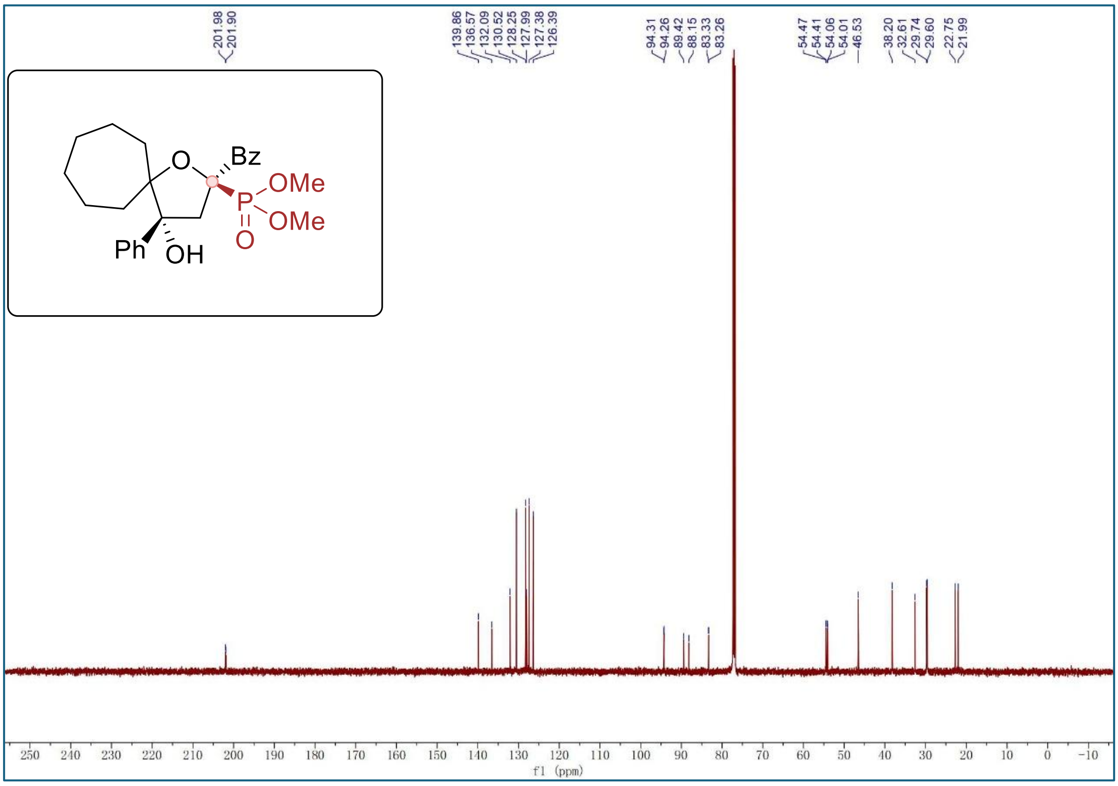

^{13}C NMR-spectrum (126 MHz, Chloroform-*d*) of **82**

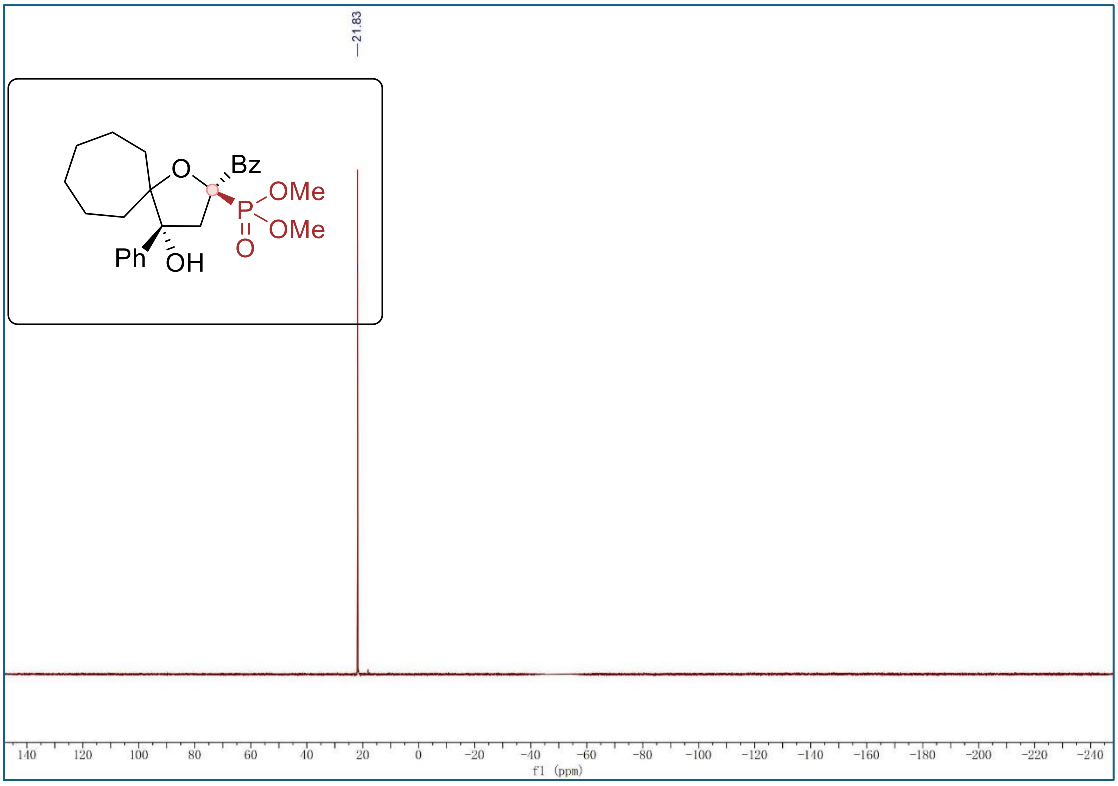

^{31}P NMR-spectrum (243 MHz, Chloroform-*d*) of **82**

diethyl (2-benzoyl-4-hydroxy-4-methyl-1,8-dioxaspiro[4.5]decan-2-yl)phosphonate (83)

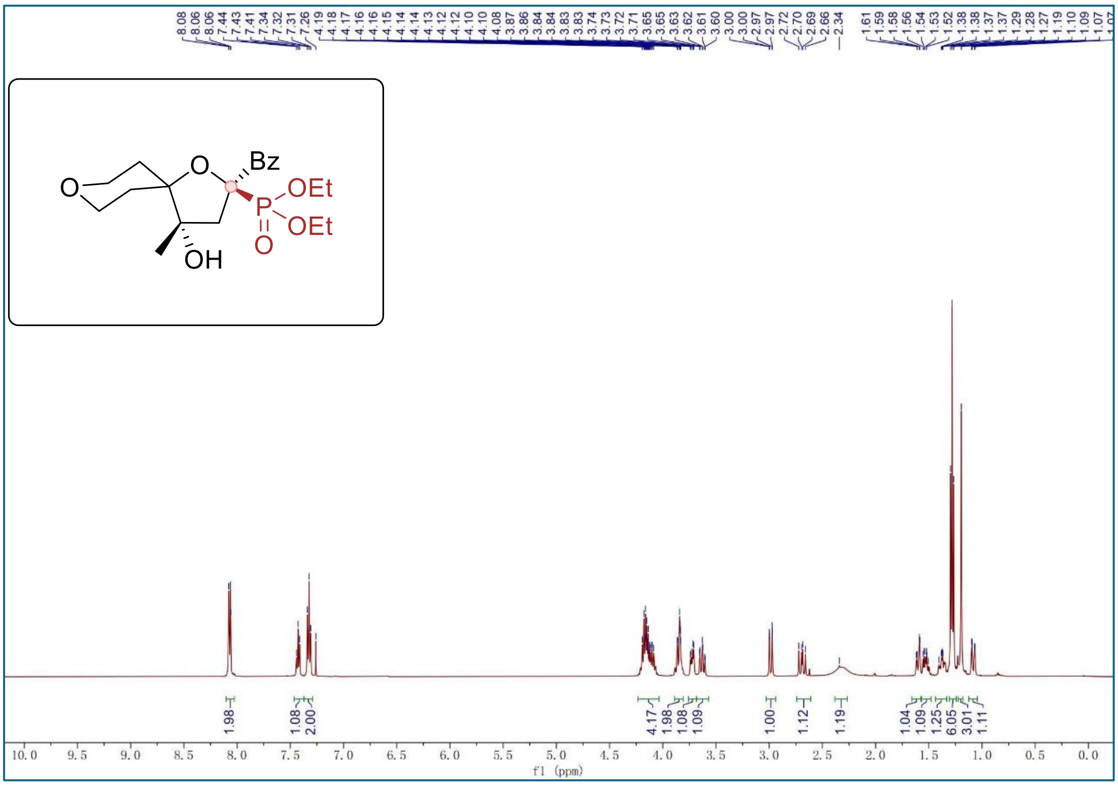

^{1}H NMR-spectrum (500 MHz, Chloroform-*d*) of **83**

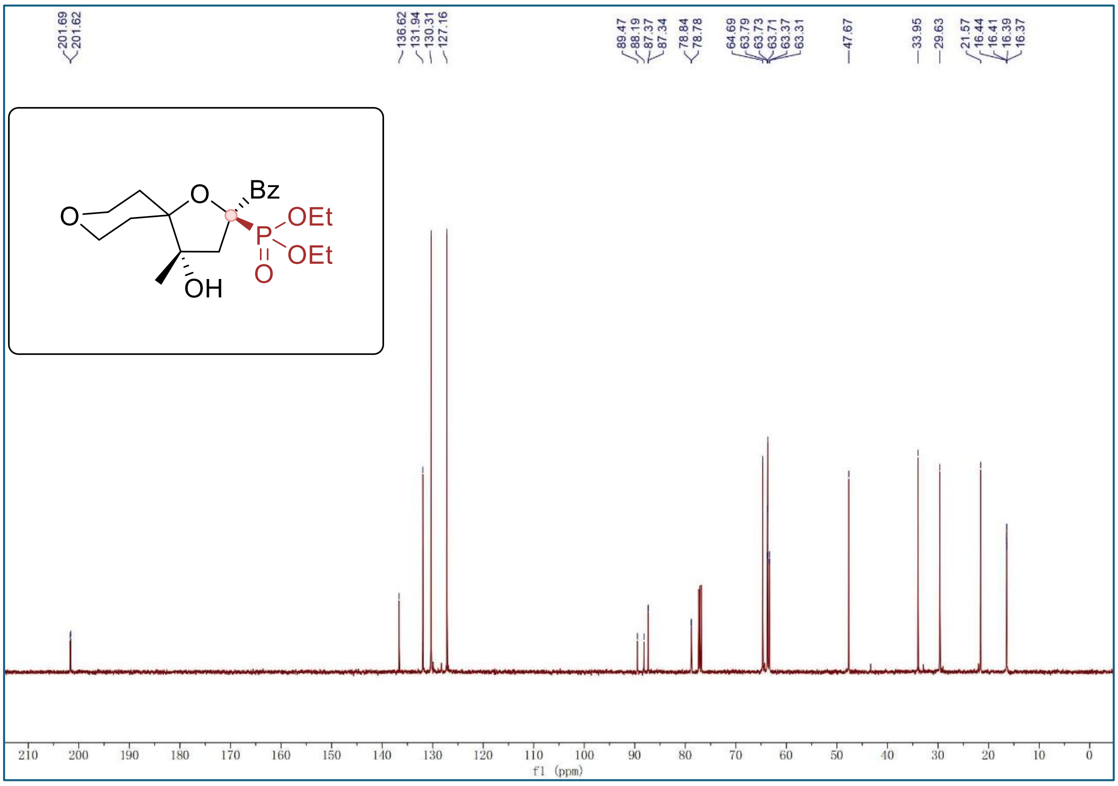

^{13}C NMR-spectrum (126 MHz, Chloroform-*d*) of **83**

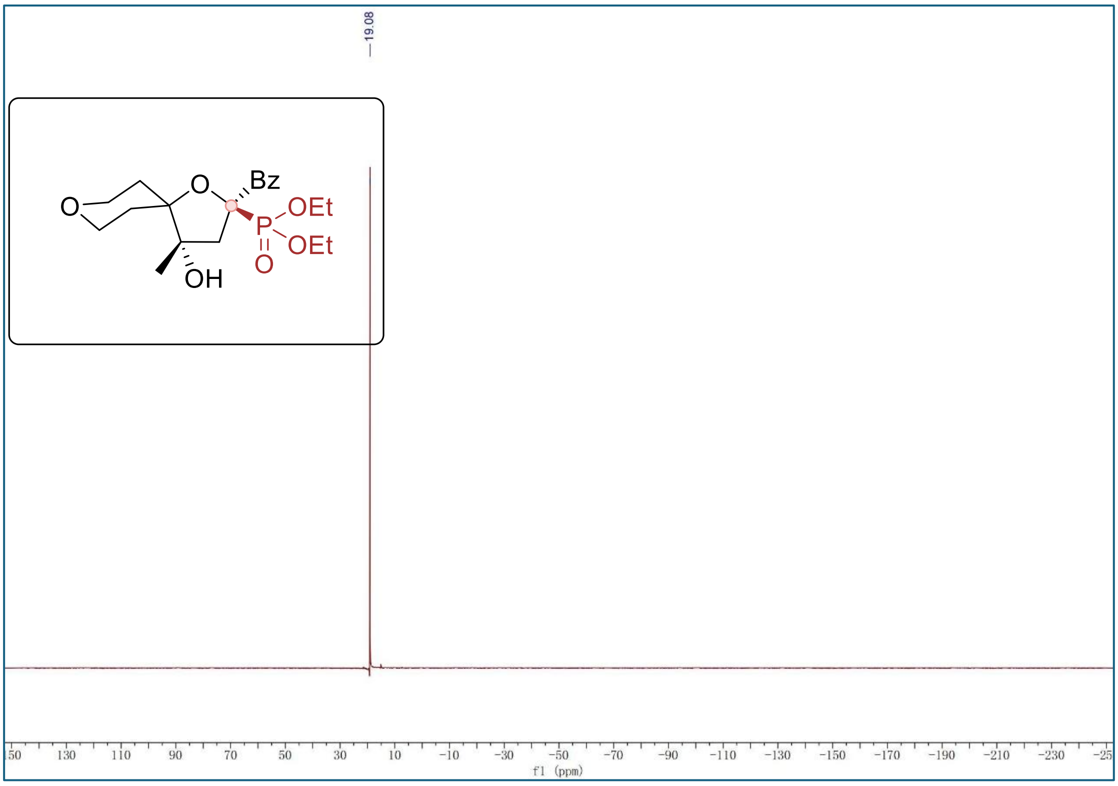

^{31}P NMR-spectrum (202 MHz, Chloroform-*d*) of **83**

diethyl (2-benzoyl-4-hydroxy-4-methyl-1-oxaspiro[4.14]nonadecan-2-yl)phosphonate (84)

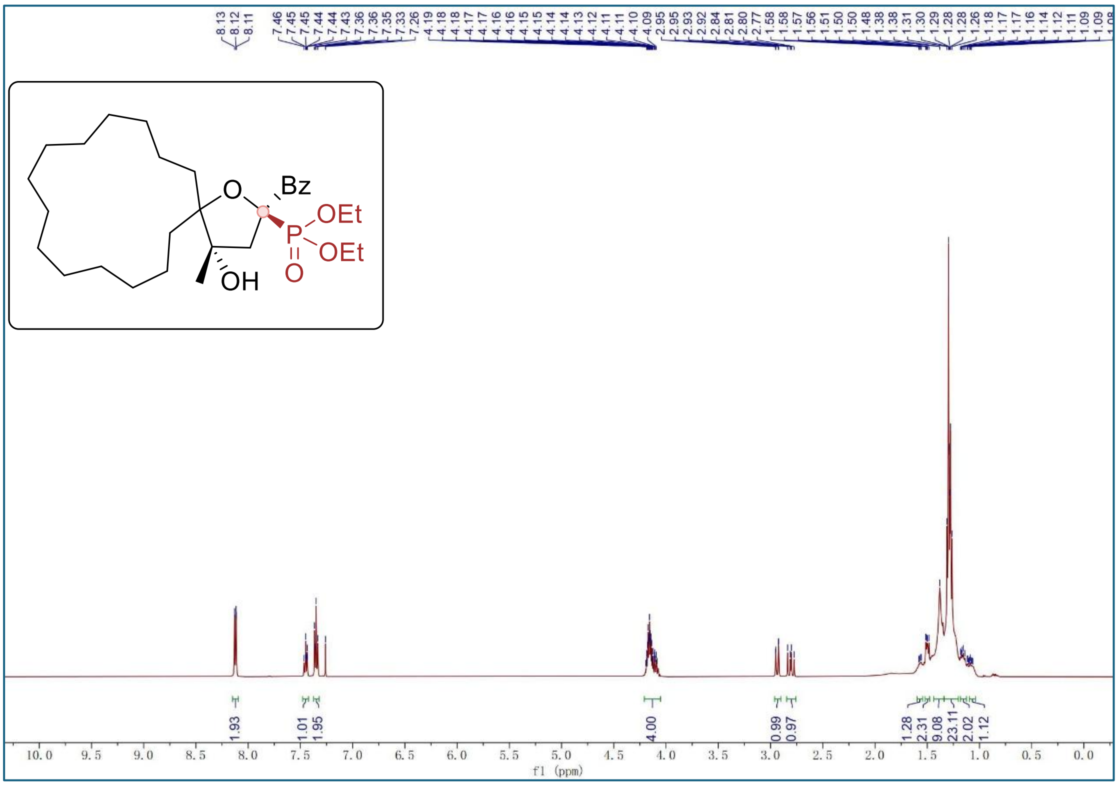

^{1}H NMR-spectrum (600 MHz, Chloroform-*d*) of **84**

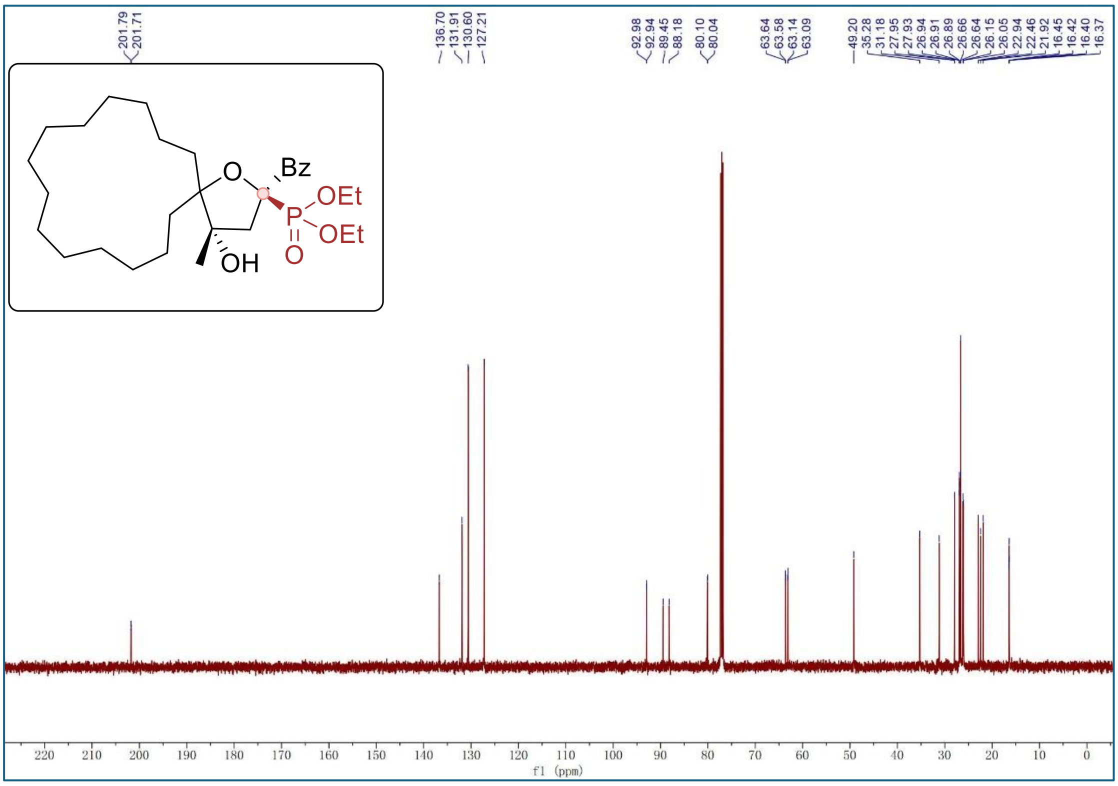

^{13}C NMR-spectrum (126 MHz, Chloroform-*d*) of **84**

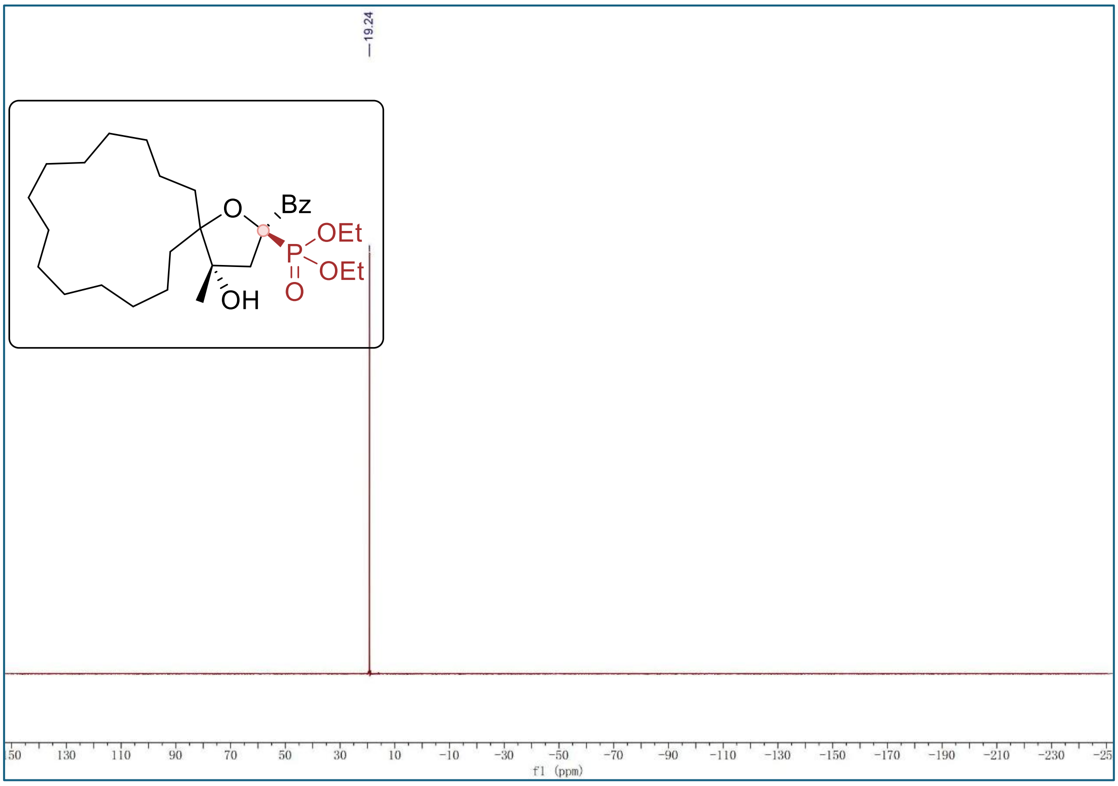

^{31}P NMR-spectrum (202 MHz, Chloroform-*d*) of **84**

SUPPORTING INFORMATION

2-benzoyl-4-hydroxy-N-methoxy-N,4-dimethyl-1,8-dioxaspiro[4.5]decane-2-carboxamide (85)

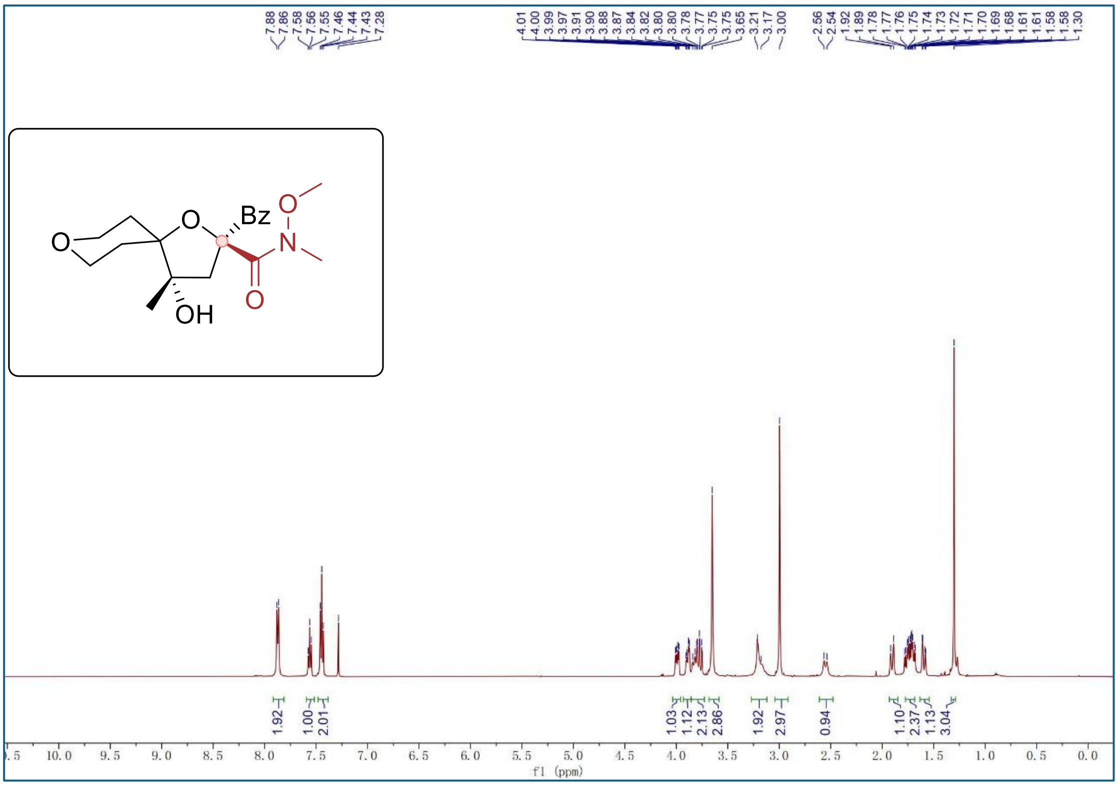

^{1}H NMR-spectrum (500 MHz, Chloroform-*d*) of **85**

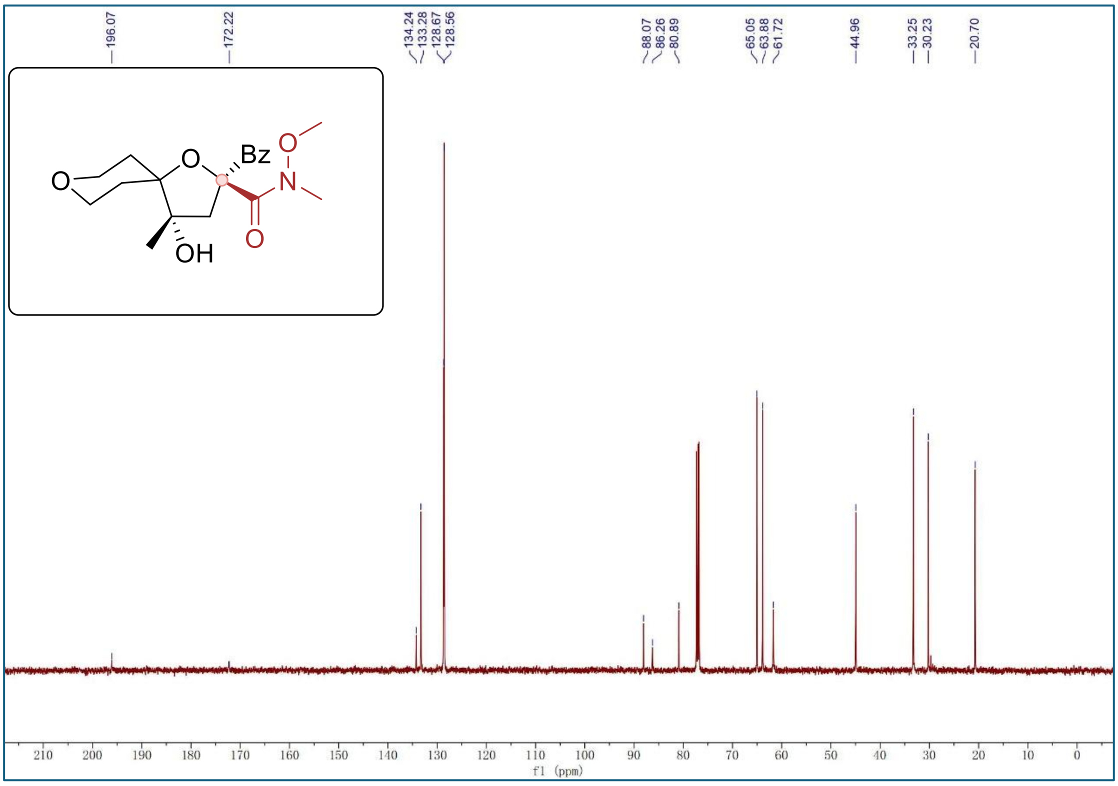

^{13}C NMR-spectrum (126 MHz, Chloroform-*d*) of **85**

(4-hydroxy-5,5-dimethyl-2-(trifluoromethyl)tetrahydrofuran-2-yl)(phenyl)methanone (86)

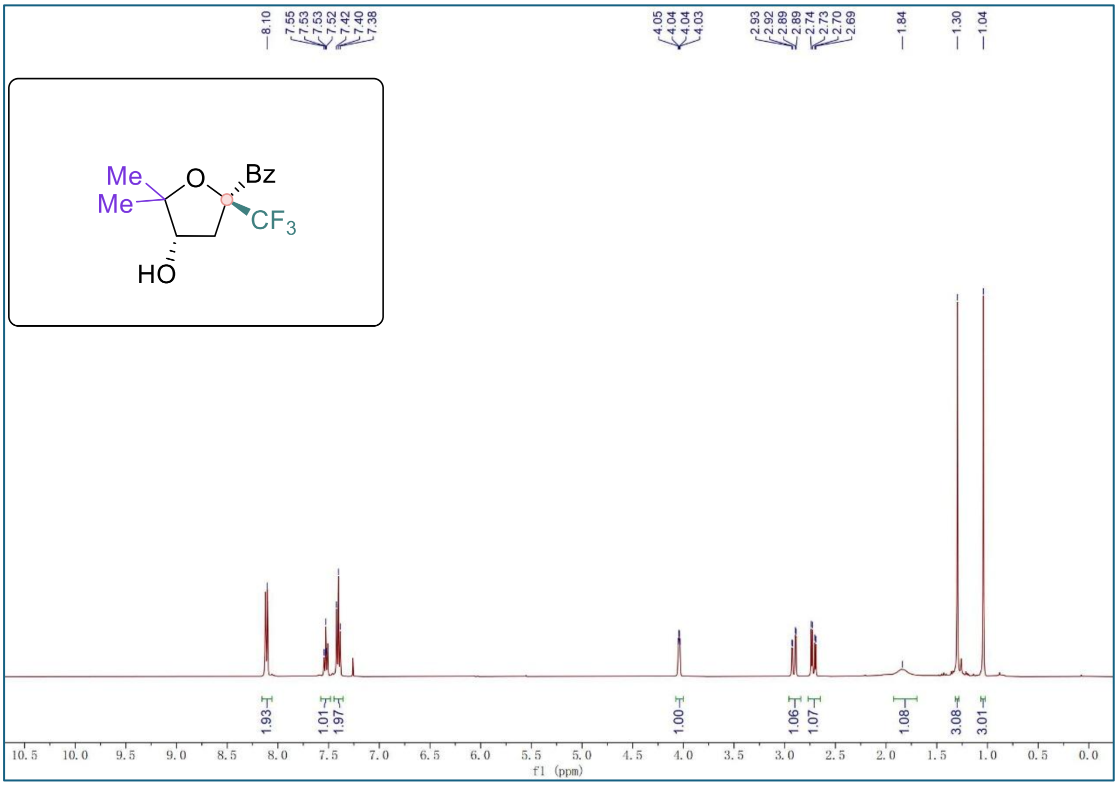

^{1}H NMR-spectrum (400 MHz, Chloroform-*d*) of **86**

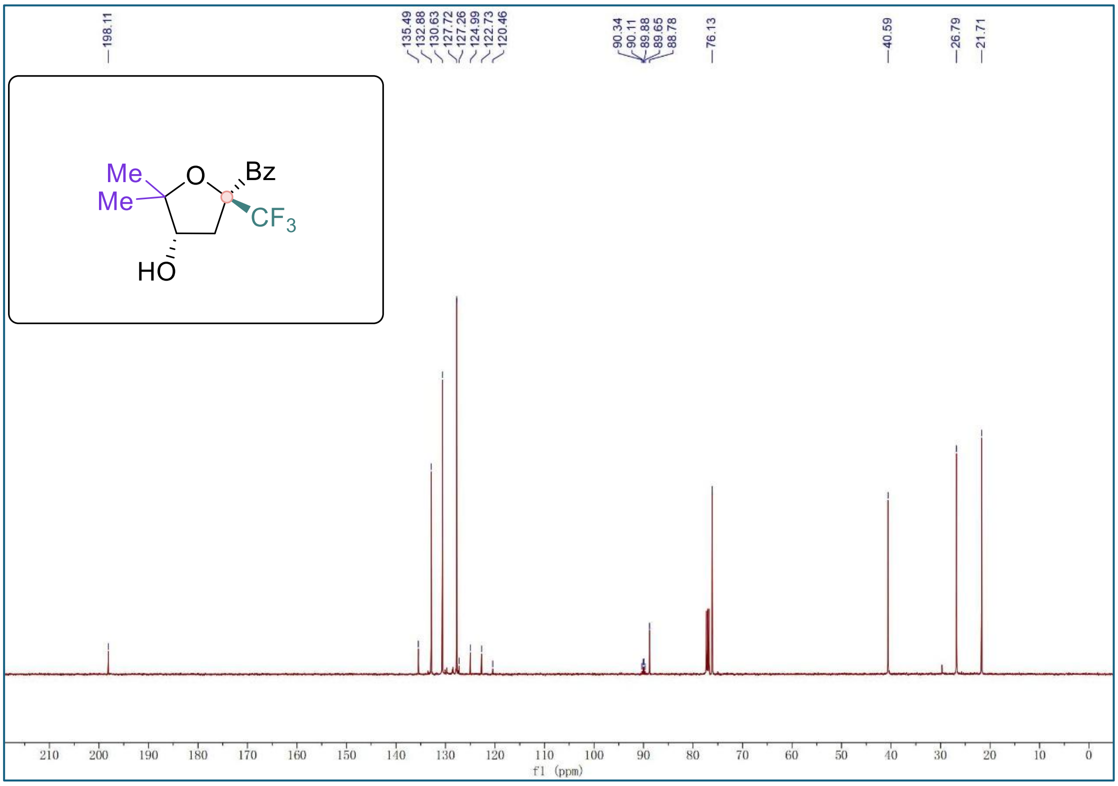

^{13}C NMR-spectrum (126 MHz, Chloroform-*d*) of **86**

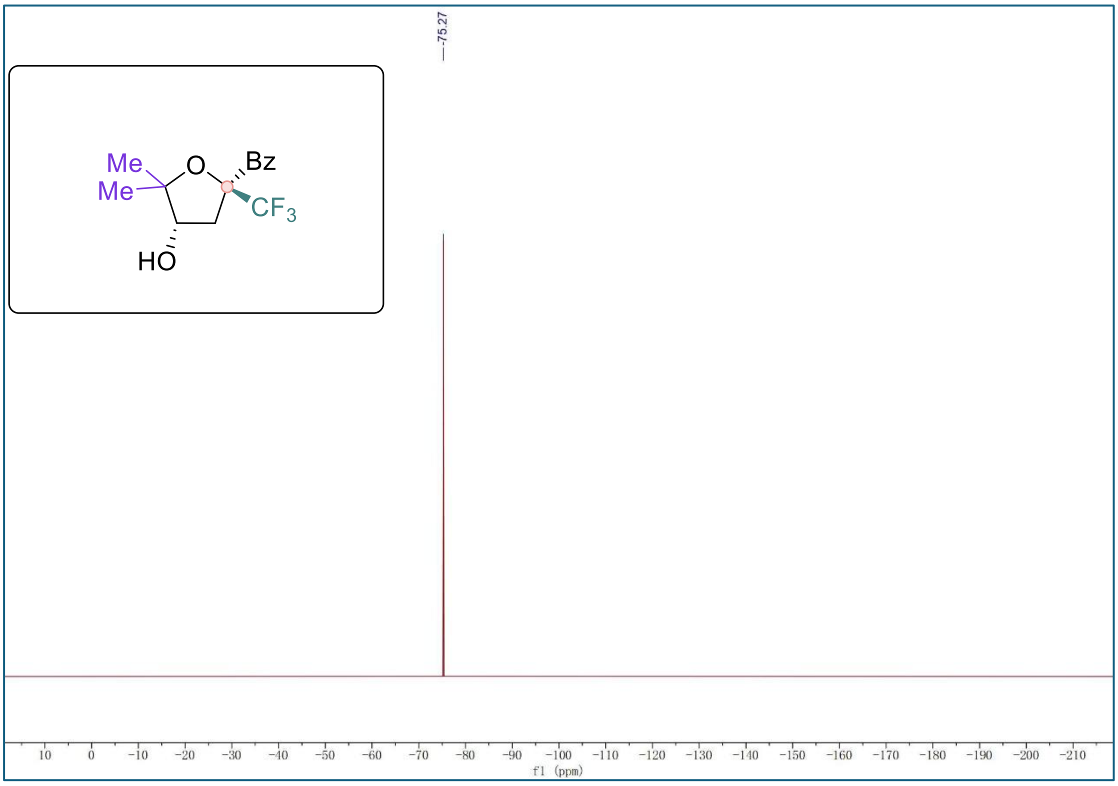

^{19}F NMR-spectrum (377 MHz, Chloroform-*d*) of **86**

(4-hydroxy-2-(trifluoromethyl)-1-oxaspiro[4.11]hexadecan-2-yl)(phenyl)methanone (87)

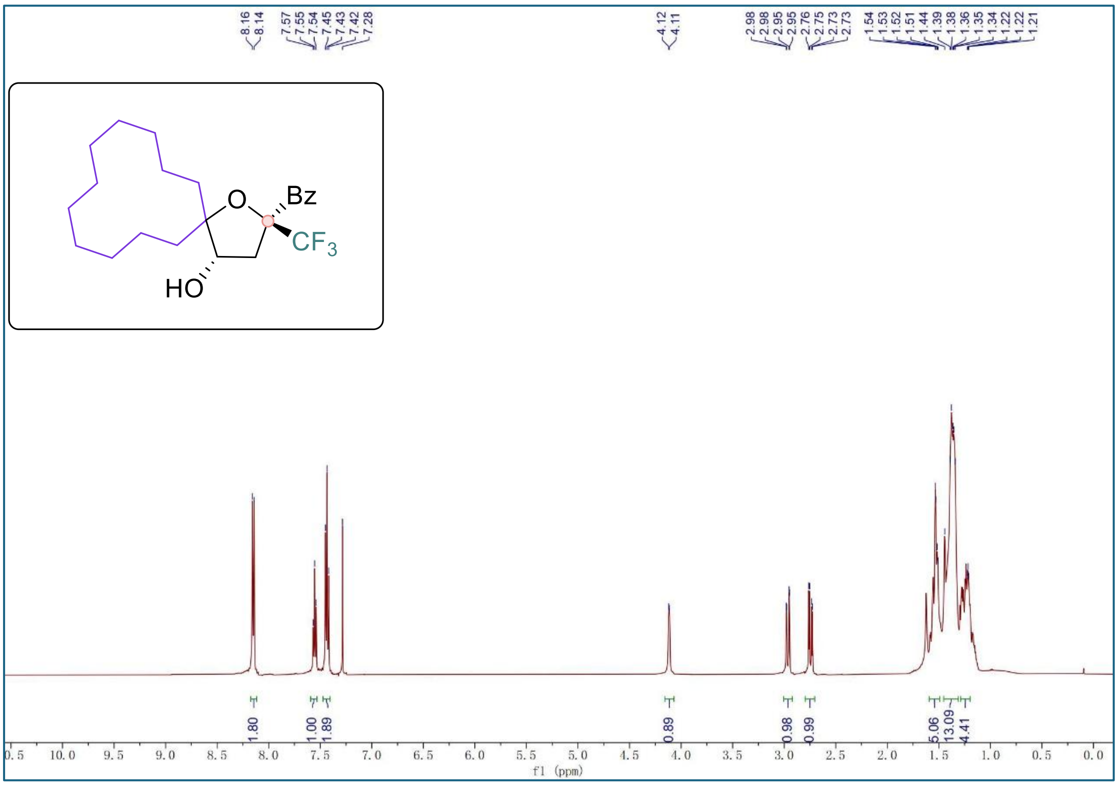

^{1}H NMR-spectrum (500 MHz, Chloroform-*d*) of **87**

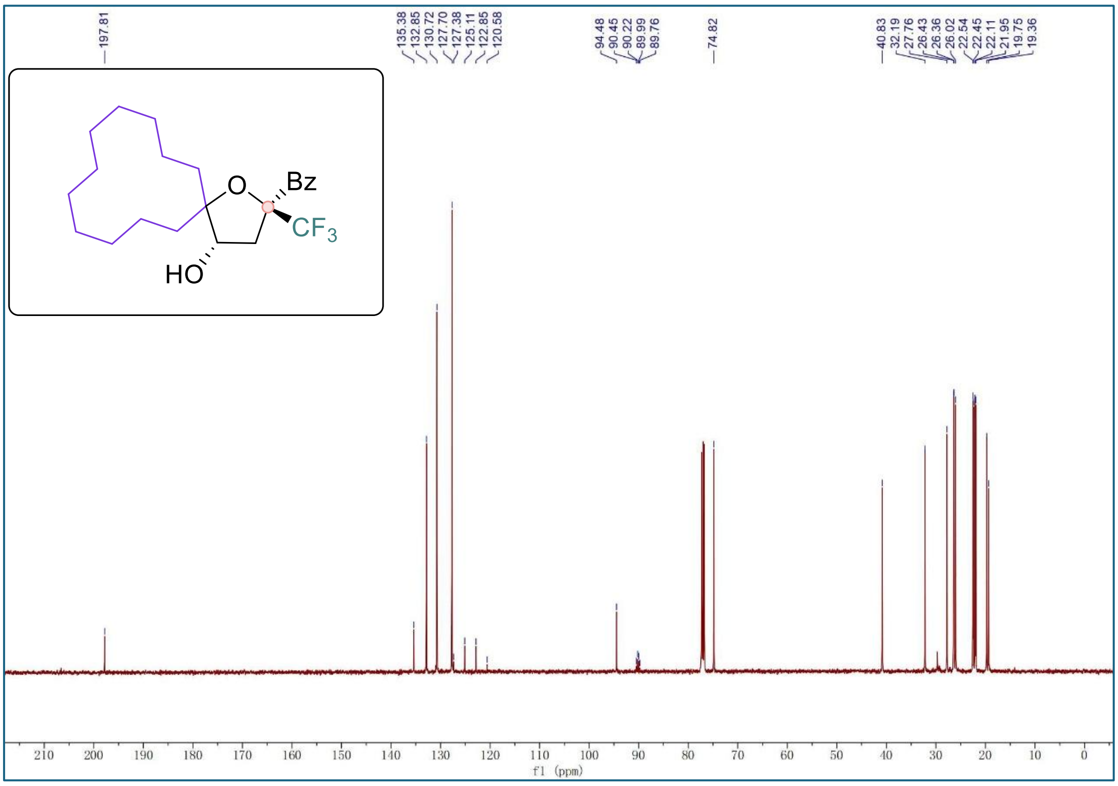

^{13}C NMR-spectrum (126 MHz, Chloroform-*d*) of **87**

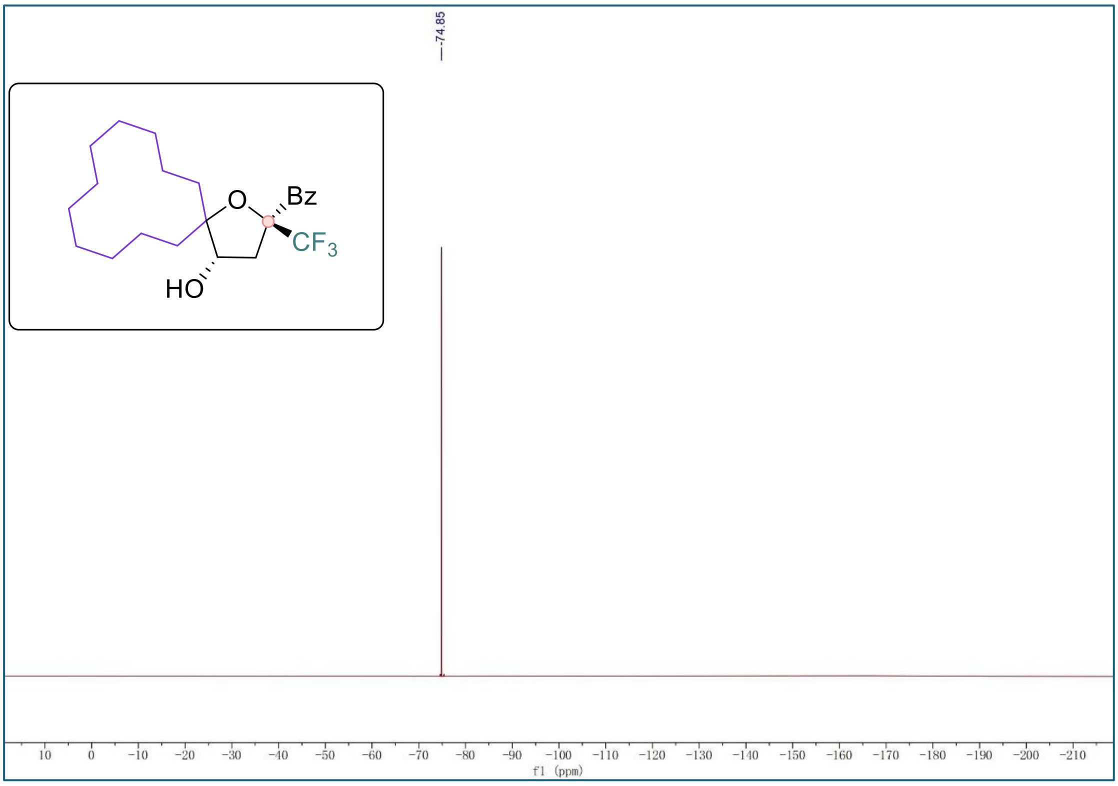

^{19}F NMR-spectrum (377 MHz, Chloroform-*d*) of **87**

(4-hydroxy-4-methyl-2-(trifluoromethyl)-1,8-dioxaspiro[4.5]decan-2-yl)(phenyl)methanone (88)

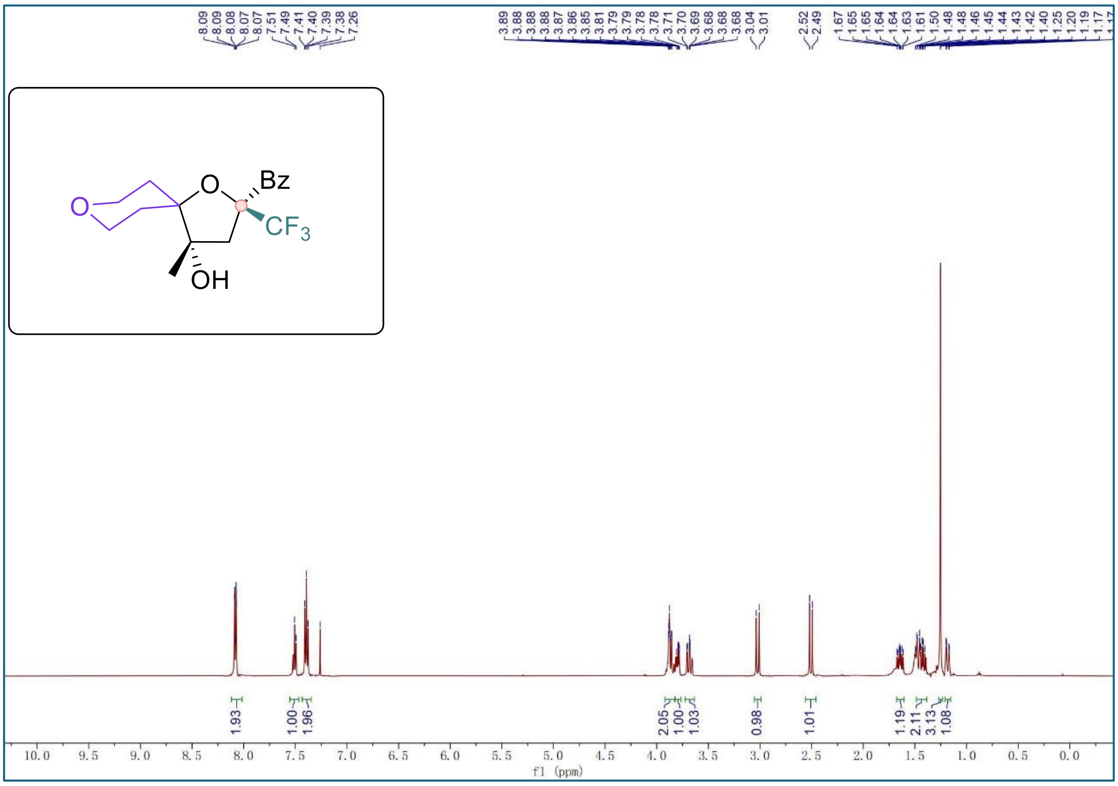

^{1}H NMR-spectrum (500 MHz, Chloroform-*d*) of **88**

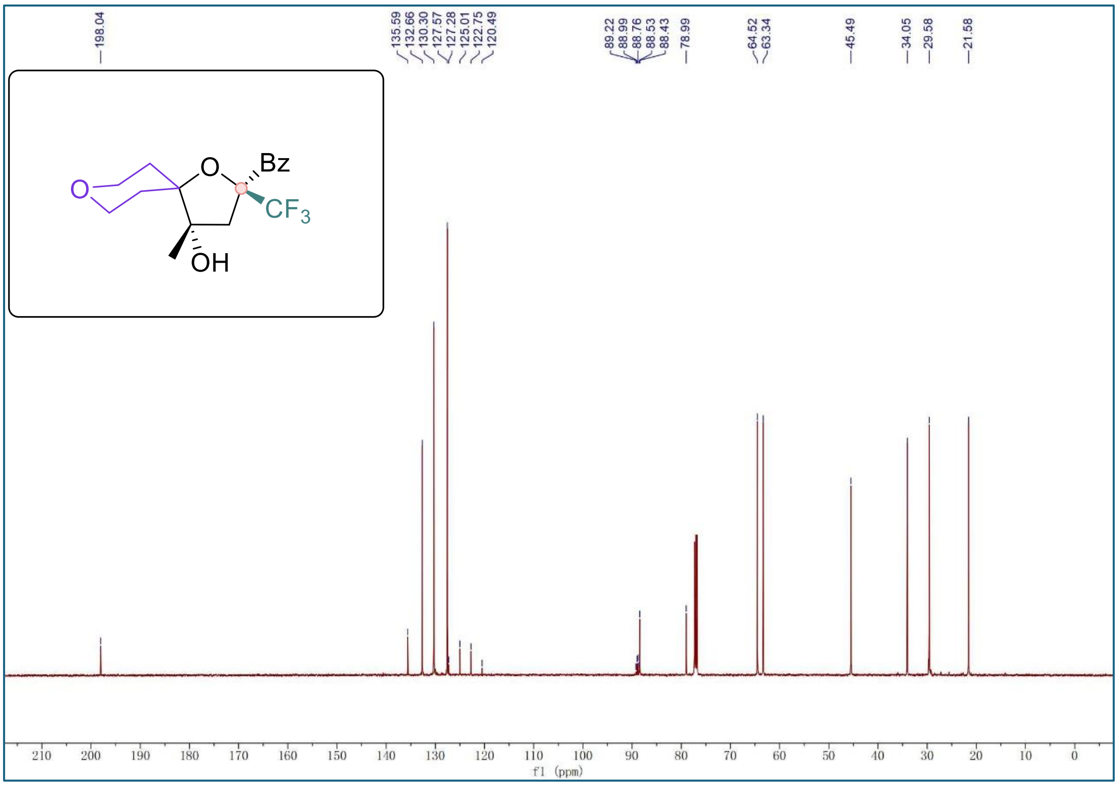

^{13}C NMR-spectrum (126 MHz, Chloroform-*d*) of **88**

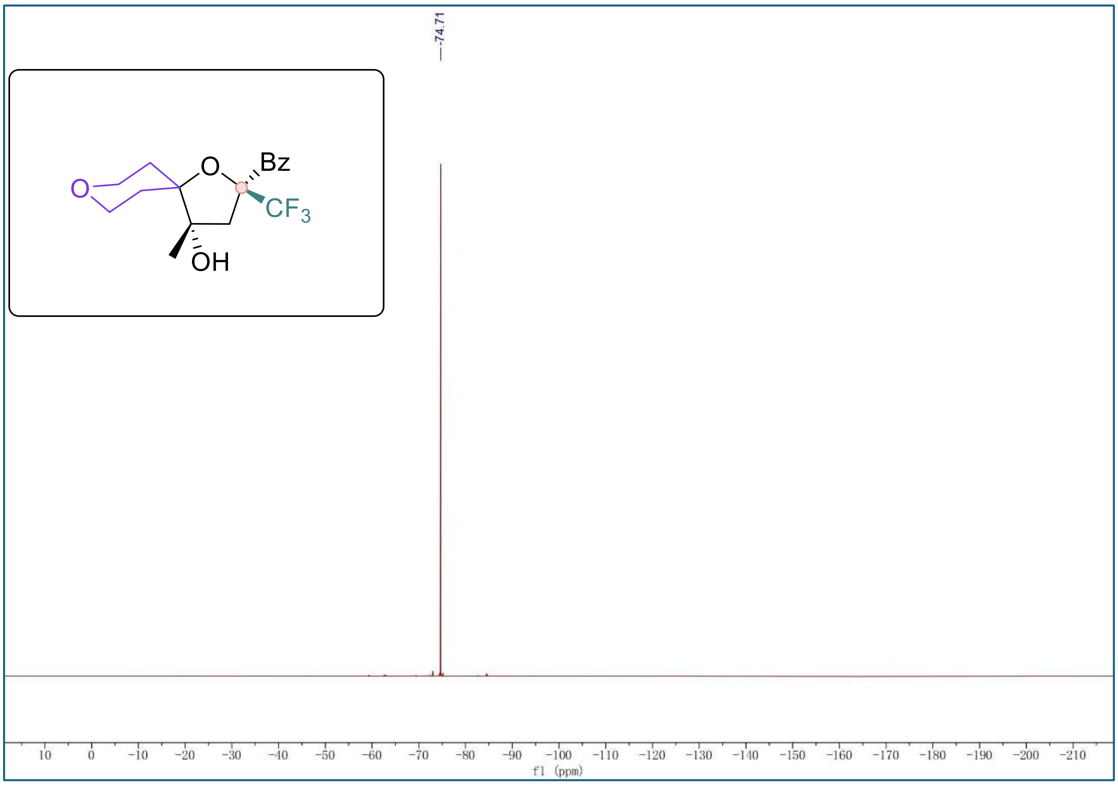

^{19}F NMR-spectrum (377 MHz, Chloroform-*d*) of **88**

(4-hydroxy-4-phenyl-2-(trifluoromethyl)-1,8-dioxaspiro[4.5]decan-2-yl)(phenyl)methanone (89)

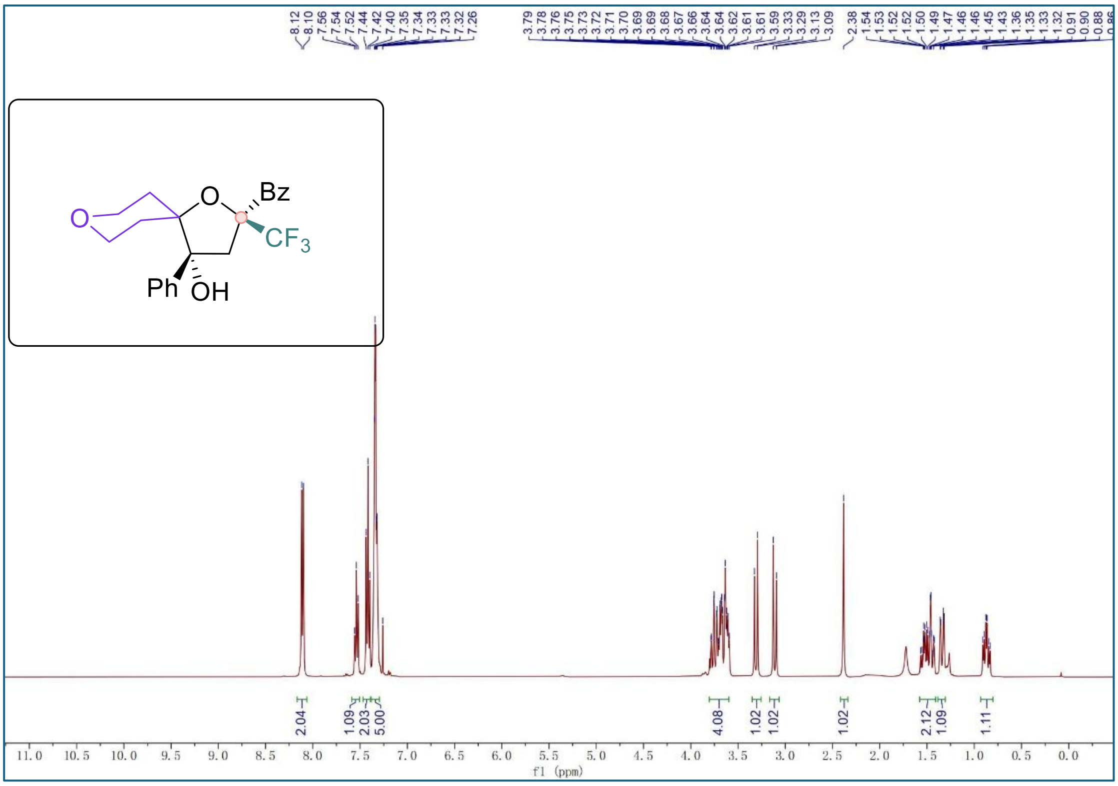

^{1}H NMR-spectrum (400 MHz, Chloroform-*d*) of **89**

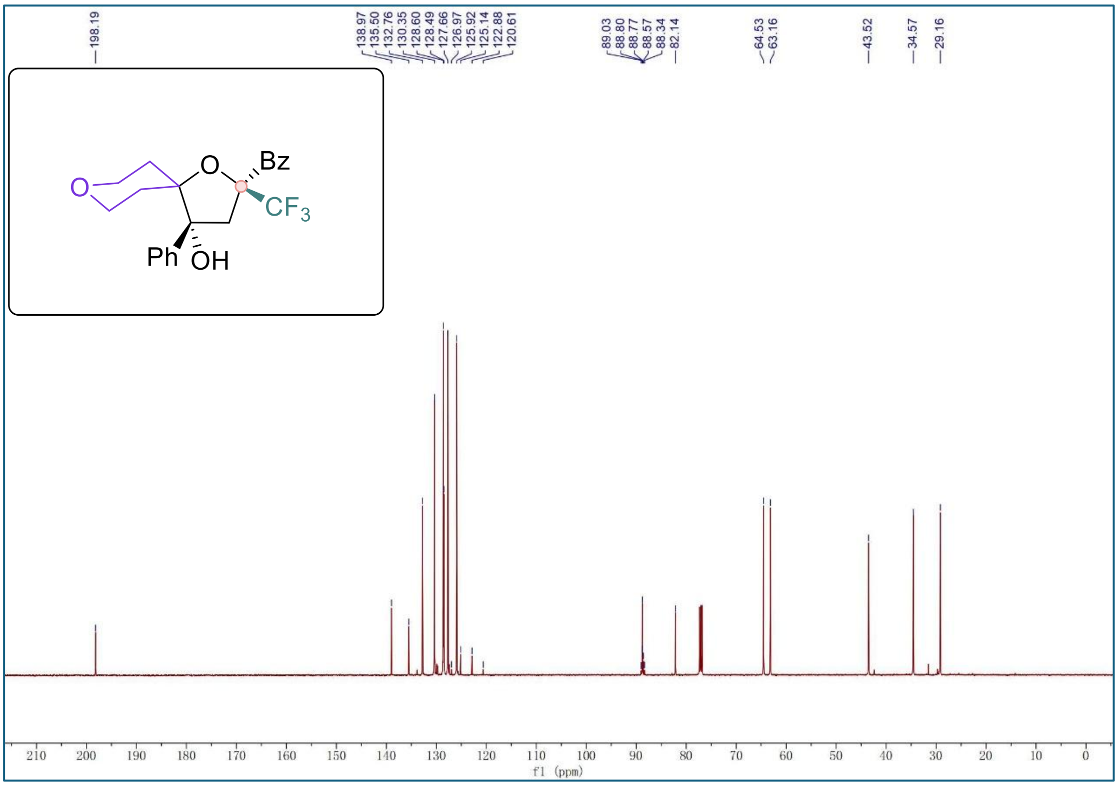

^{13}C NMR-spectrum (126 MHz, Chloroform-*d*) of **89**

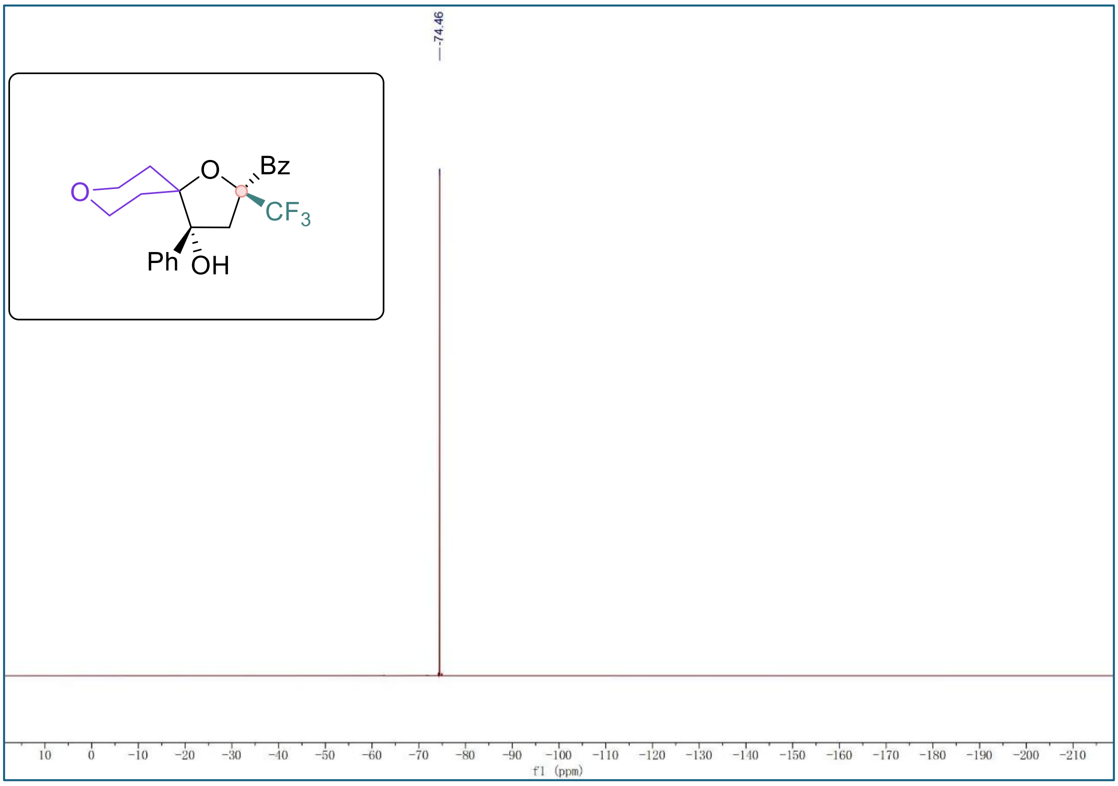

^{19}F NMR-spectrum (377 MHz, Chloroform-*d*) of **89**

ethyl 2-benzoyl-4-(((S)-3-((tert-butoxycarbonyl)amino)-3-phenylpropanoyl)oxy)-5,5-dimethyltetrahydrofuran-2-carboxylate (90)

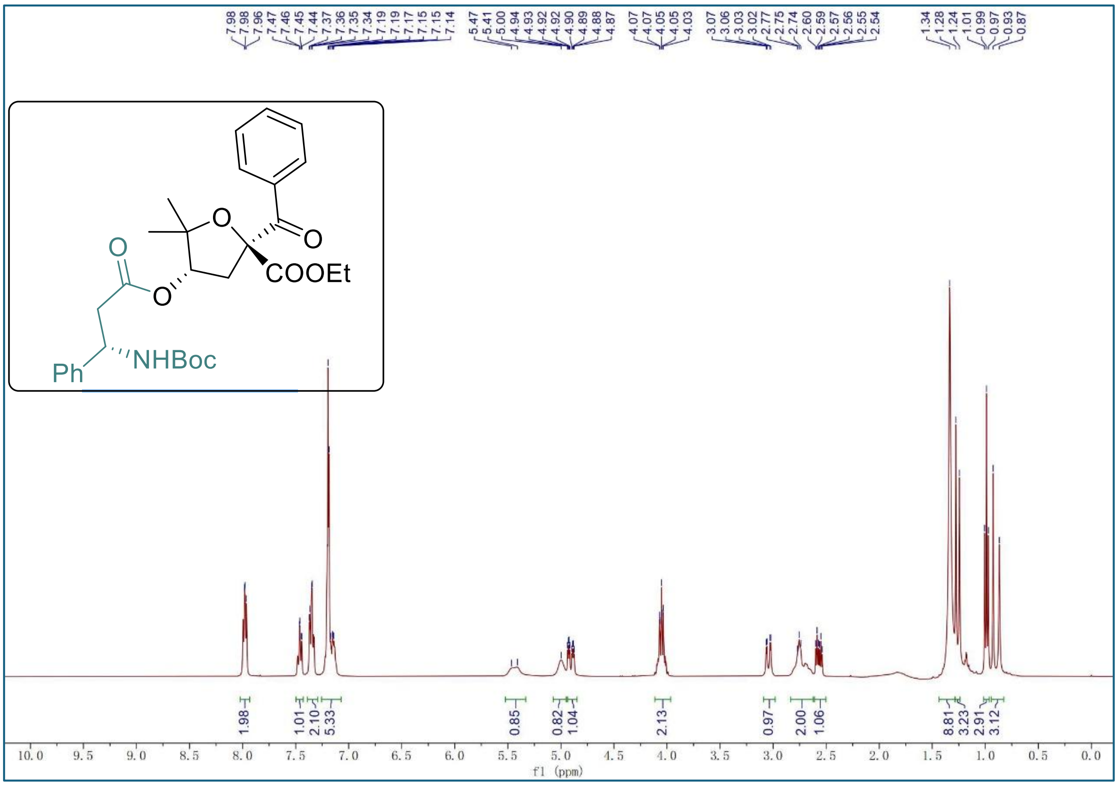

^{1}H NMR-spectrum (400 MHz, Chloroform-*d*) of **90**

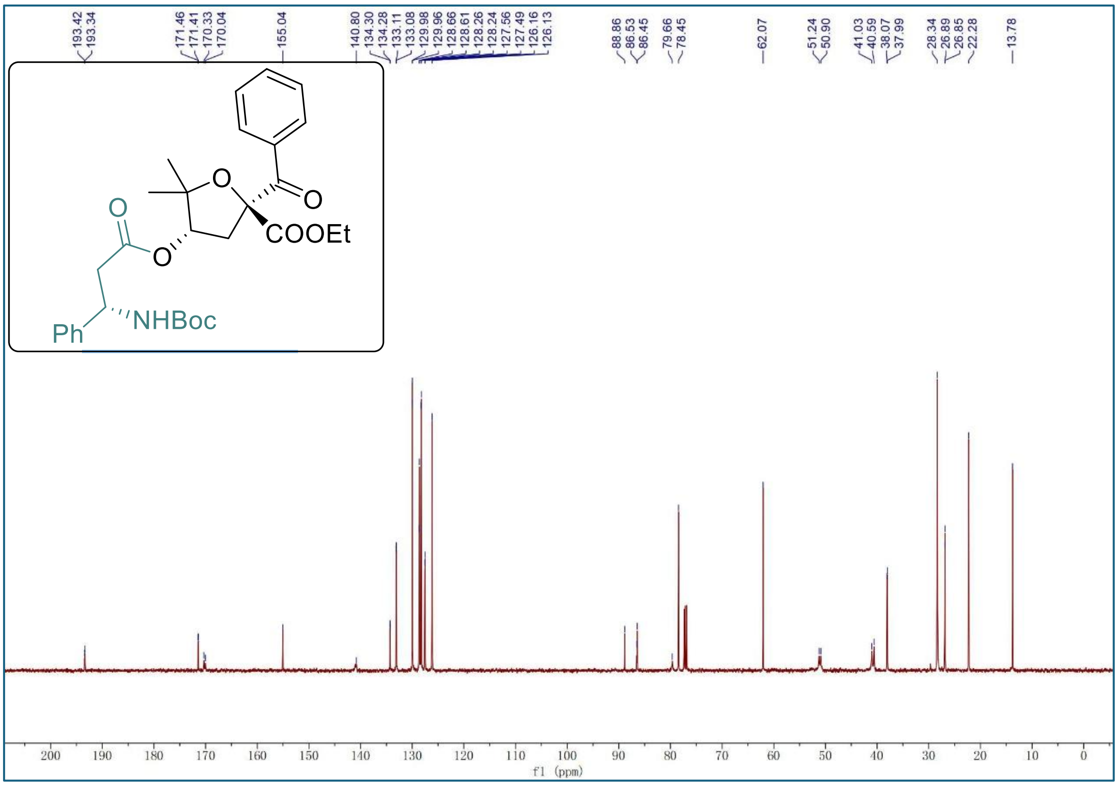

^{13}C NMR-spectrum (126 MHz, Chloroform-*d*) of **90**

ethyl 2-benzoyl-5,5-dimethyl-4-oxotetrahydrofuran-2-carboxylate (91)

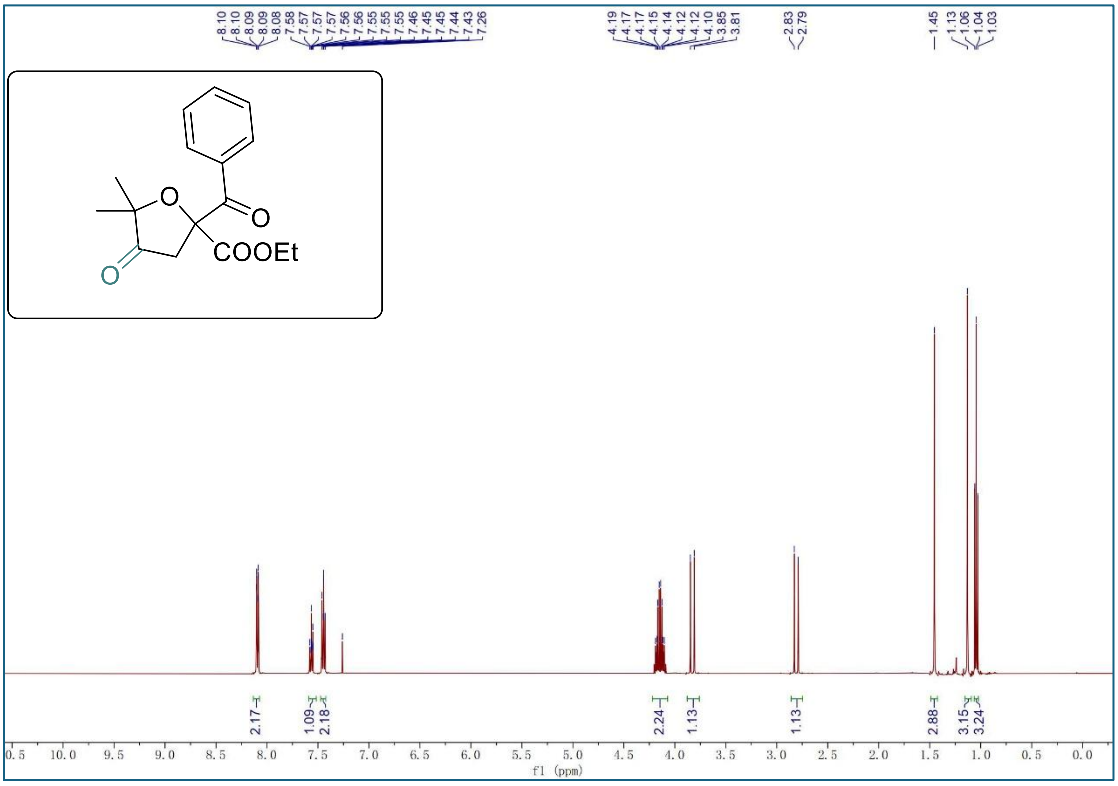

^{1}H NMR-spectrum (500 MHz, Chloroform-*d*) of **91**

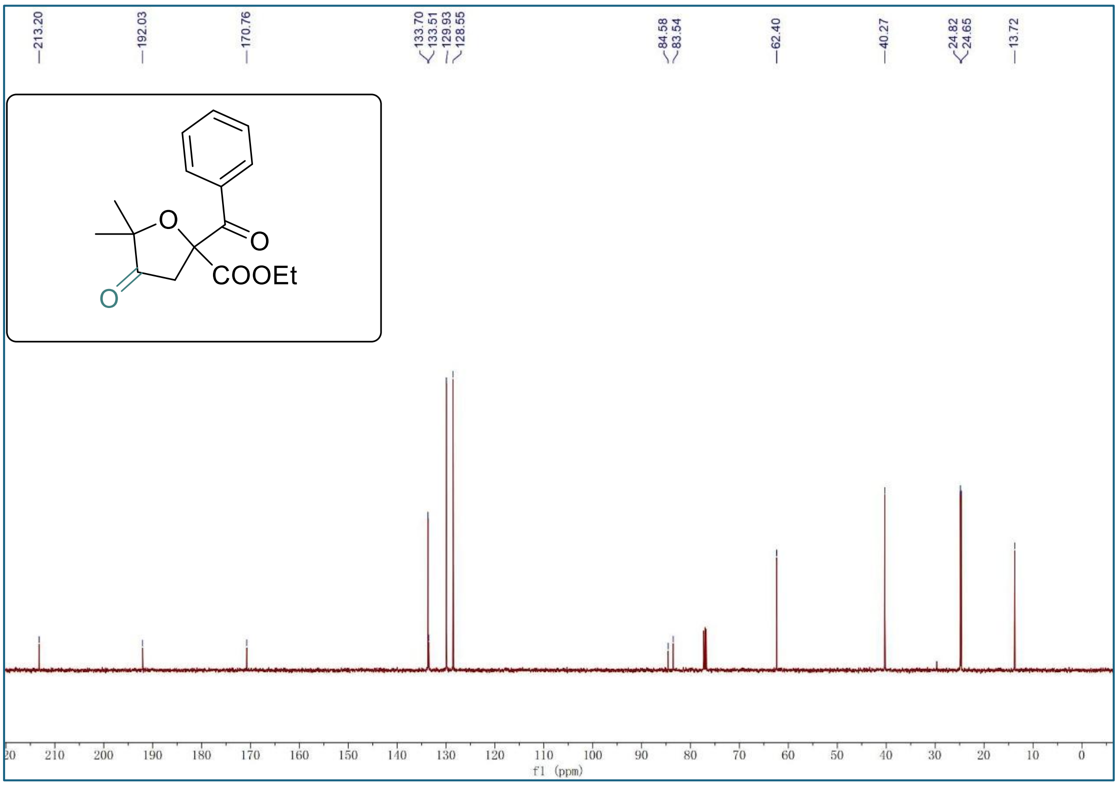

^{13}C NMR-spectrum (126 MHz, Chloroform-*d*) of **91**

SUPPORTING INFORMATION

ethyl 2-benzoyl-5,5-dimethyl-2,5-dihydrofuran-2-carboxylate (92)

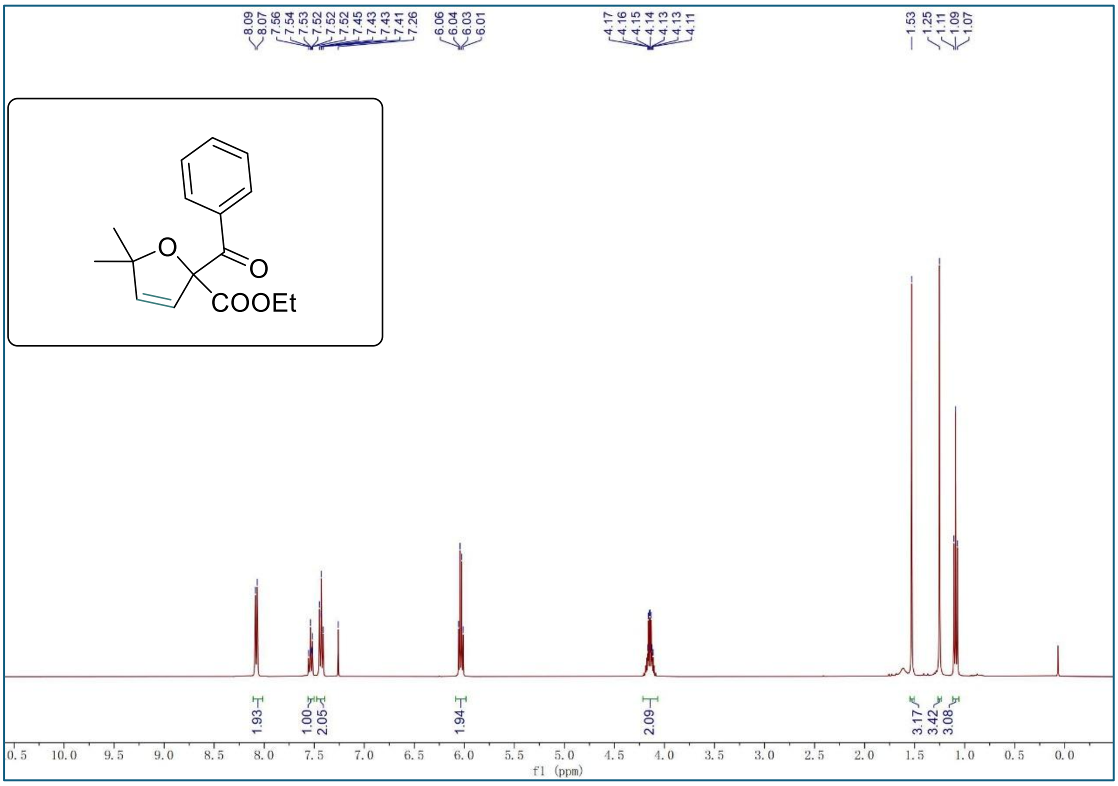

^{1}H NMR-spectrum (400 MHz, Chloroform-*d*) of **92**

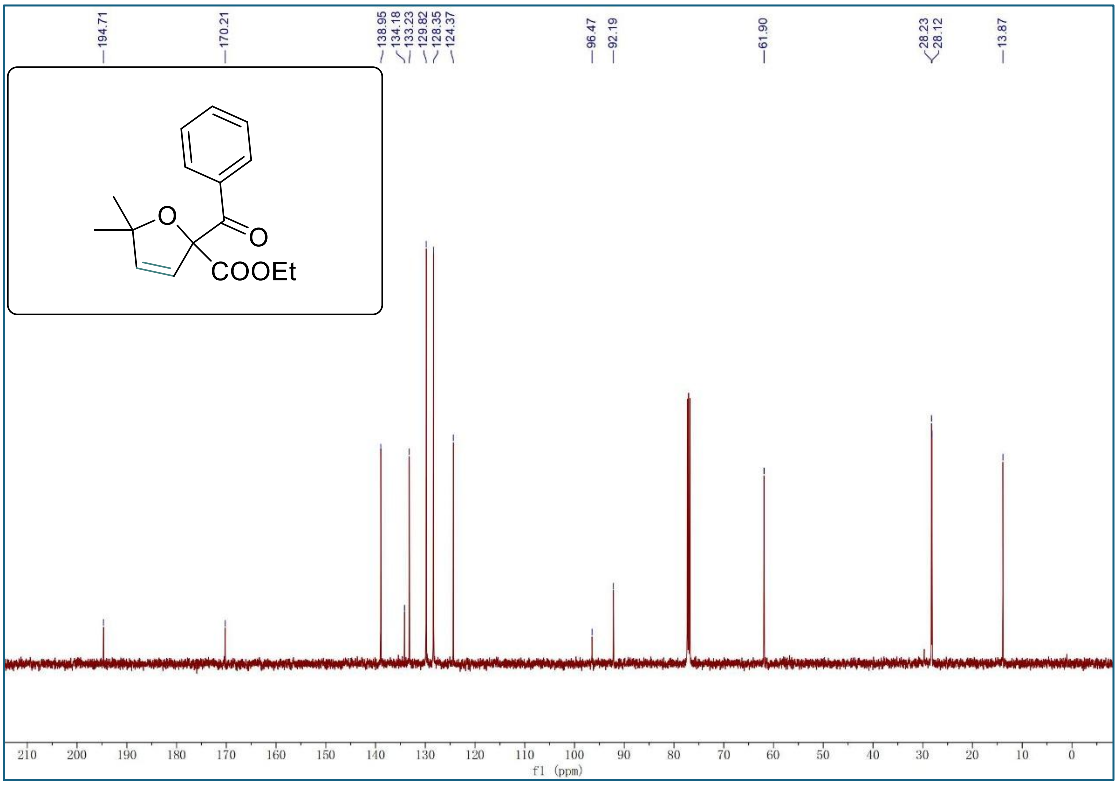

^{13}C NMR-spectrum (126 MHz, Chloroform-*d*) of **92**

ethyl 2-benzoyl-4-bromo-5,5-dimethyltetrahydrofuran-2-carboxylate (93)

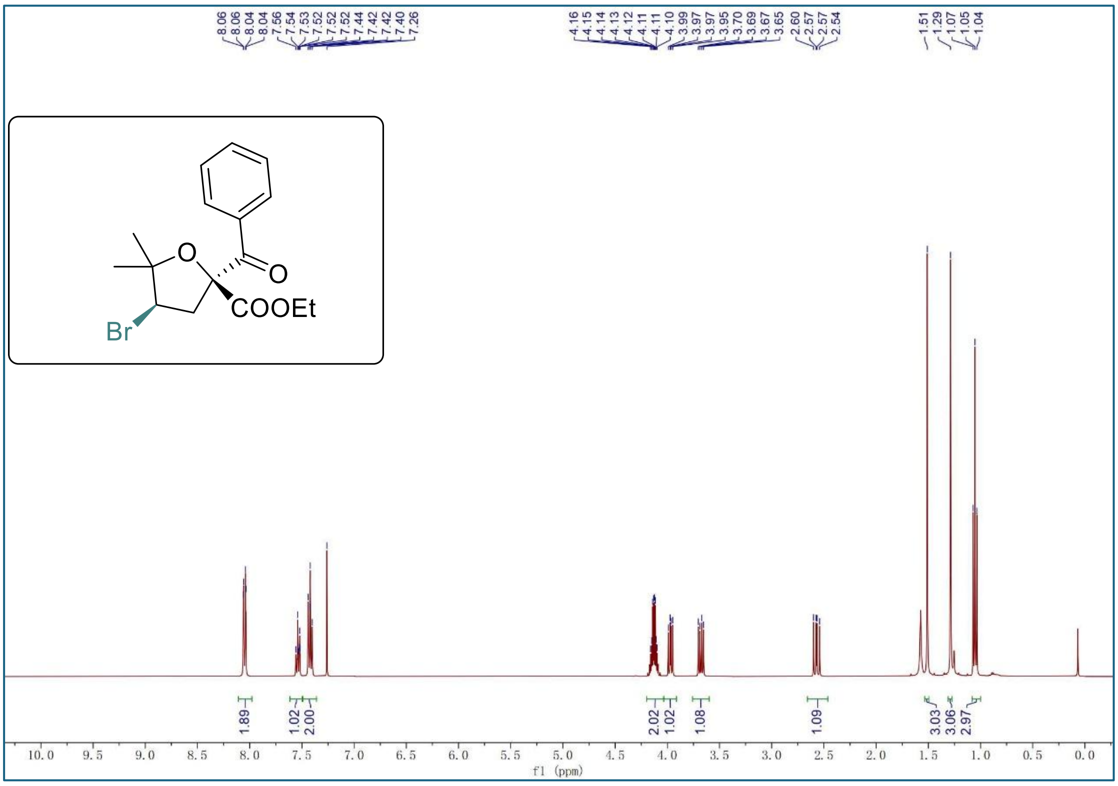

^{1}H NMR-spectrum (400 MHz, Chloroform-*d*) of **93**

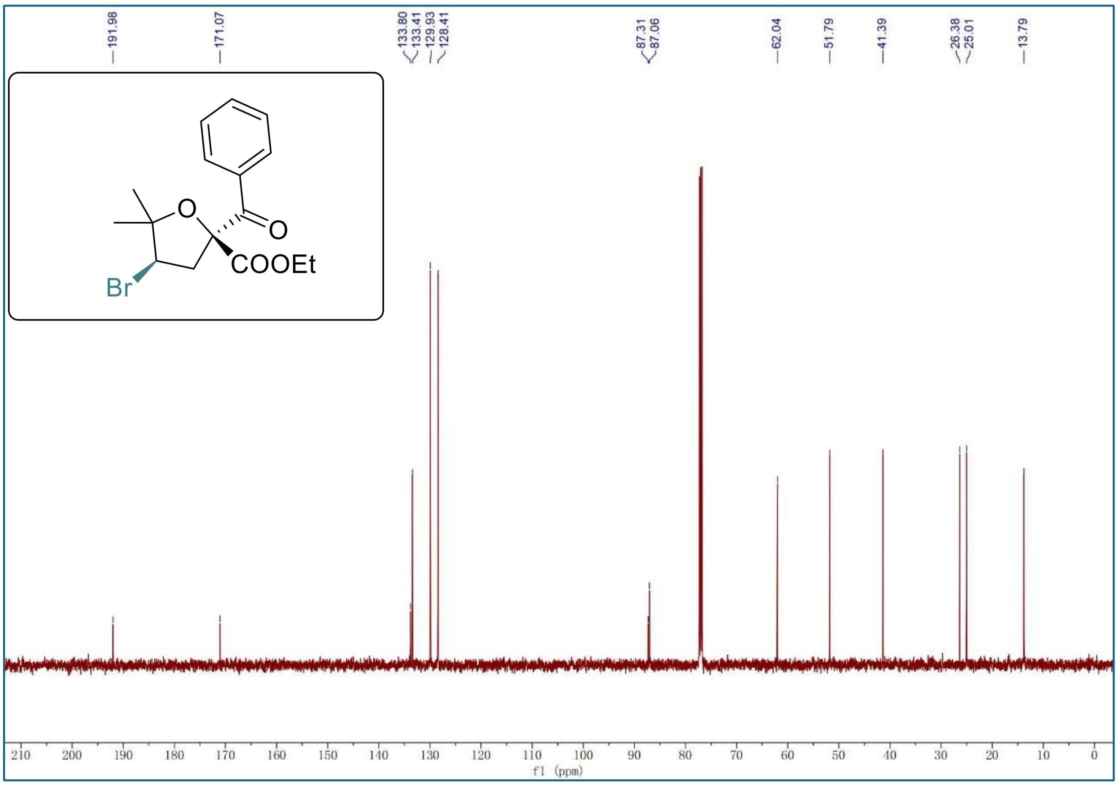

^{13}C NMR-spectrum (126 MHz, Chloroform-*d*) of **93**

ethyl 2-benzoyl-4- (Ferrocenoyloxy)-5,5-dimethyltetrahydrofuran-2-carboxylate (94)

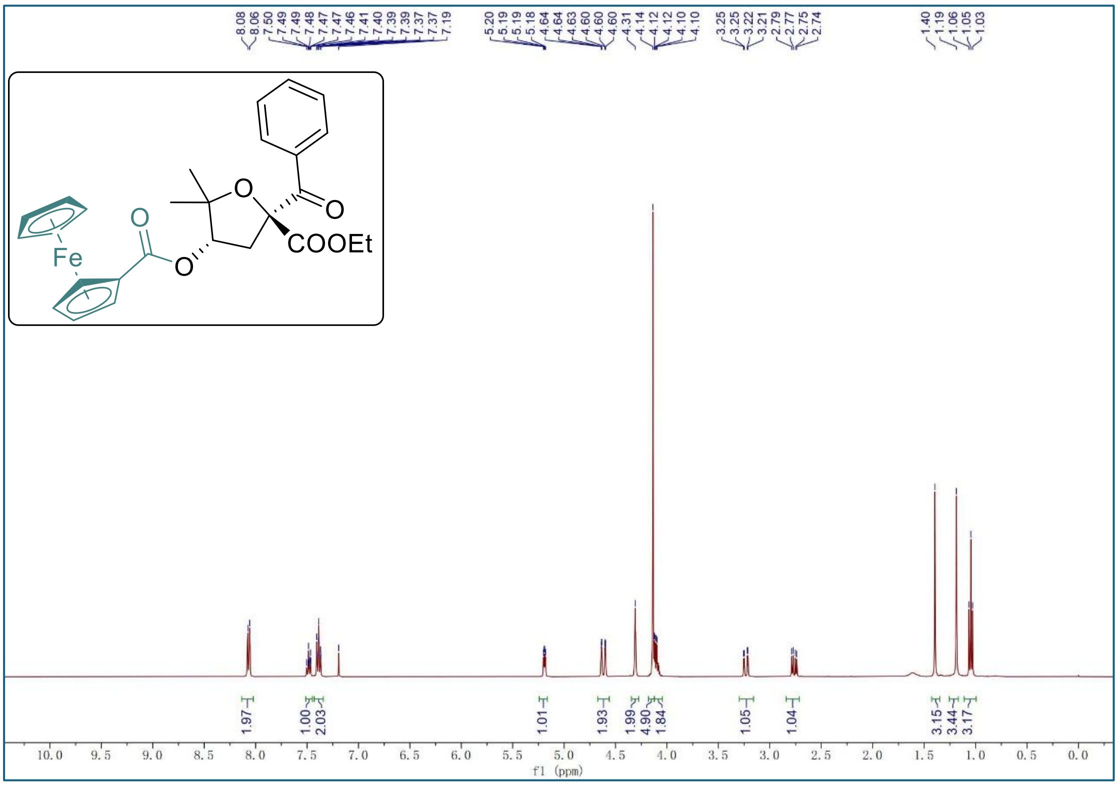

^{1}H NMR-spectrum (400 MHz, Chloroform-*d*) of **94**

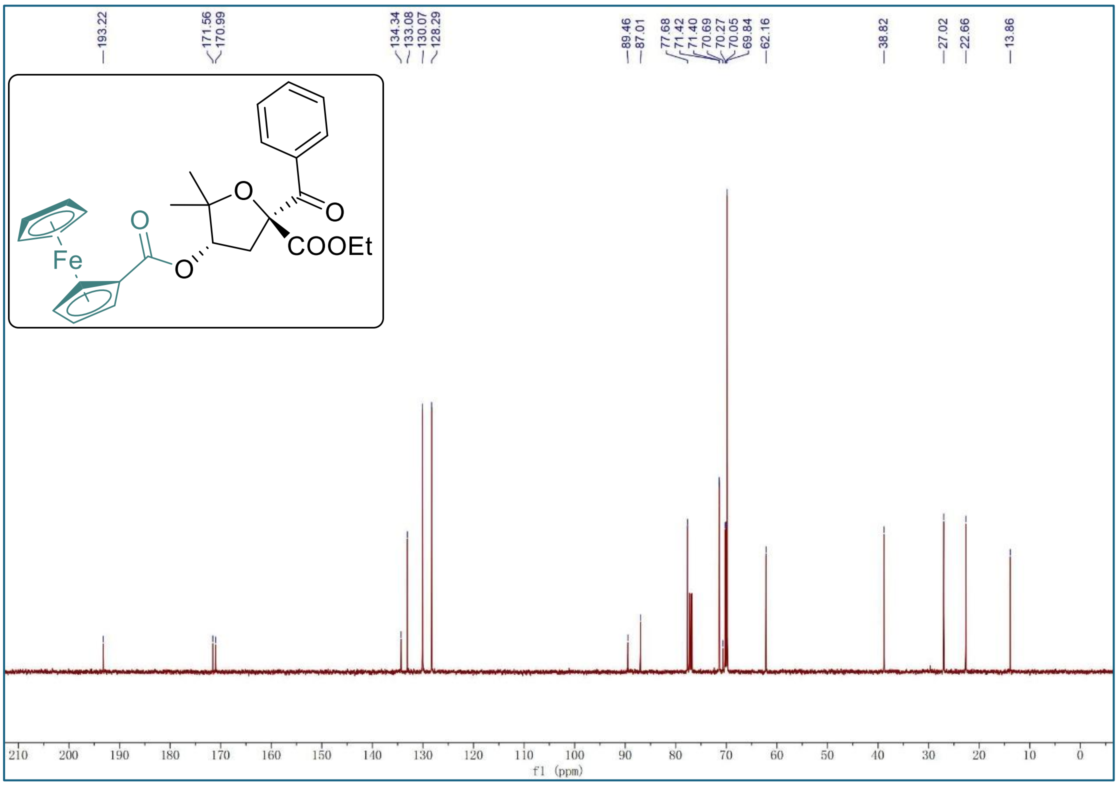

^{13}C NMR-spectrum (126 MHz, Chloroform-*d*) of **94**

ethyl 2-benzoyl-4-((tert-butyldimethylsilyl)oxy)-5,5-dimethyltetrahydrofuran-2-carboxylate (95)

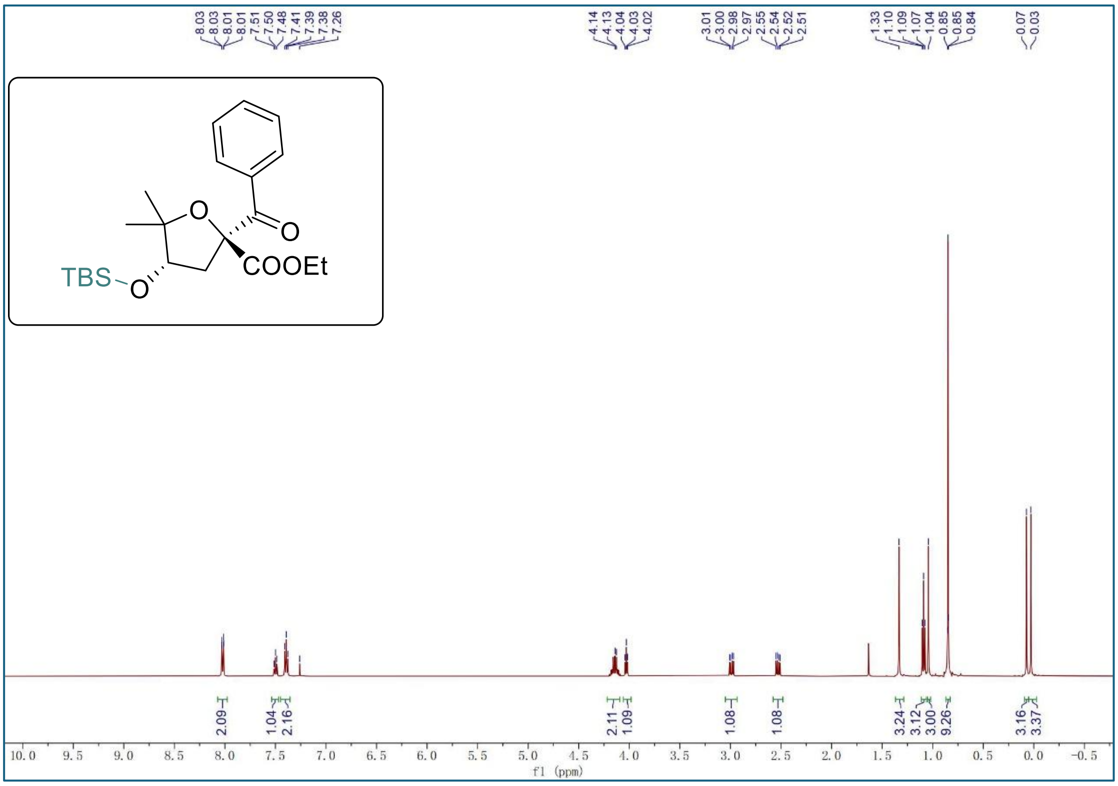

^{1}H NMR-spectrum (500 MHz, Chloroform-*d*) of **95**

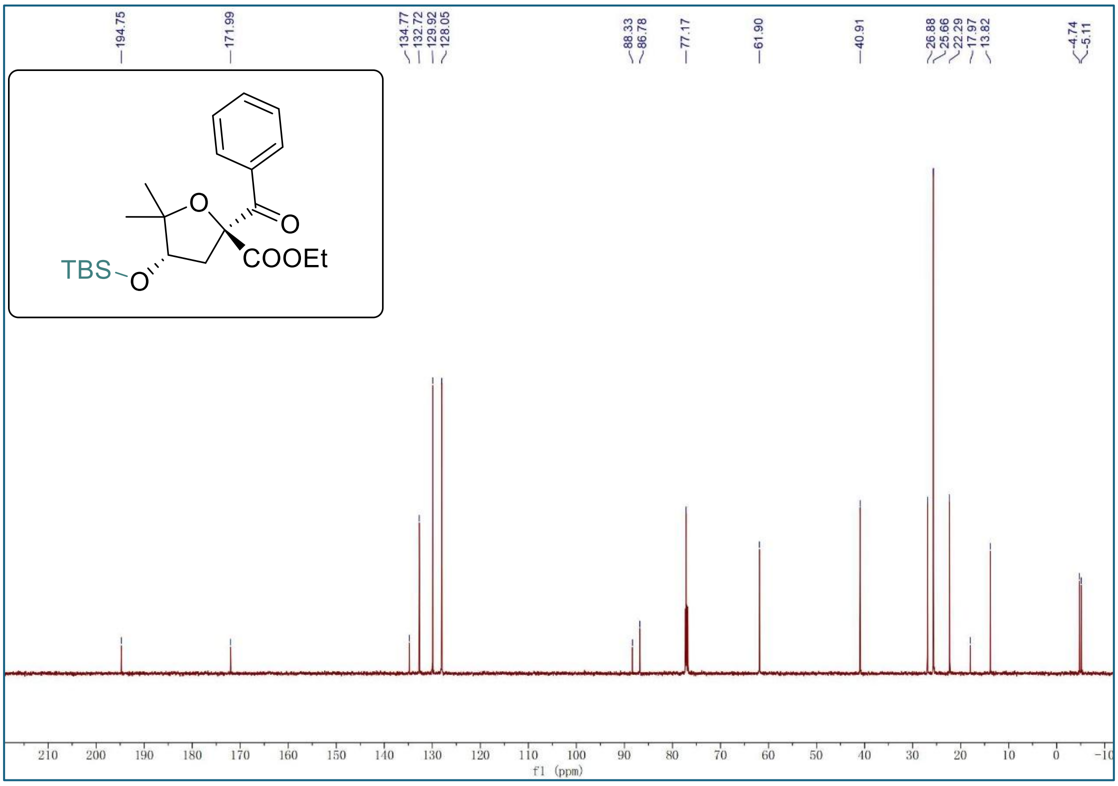

^{13}C NMR-spectrum (126 MHz, Chloroform-*d*) of **95**

ethyl4-hydroxy-2-((hydroxyimino)(phenyl)methyl)-5,5-dimethyltetrahydrofuran-2-carboxylate (96)

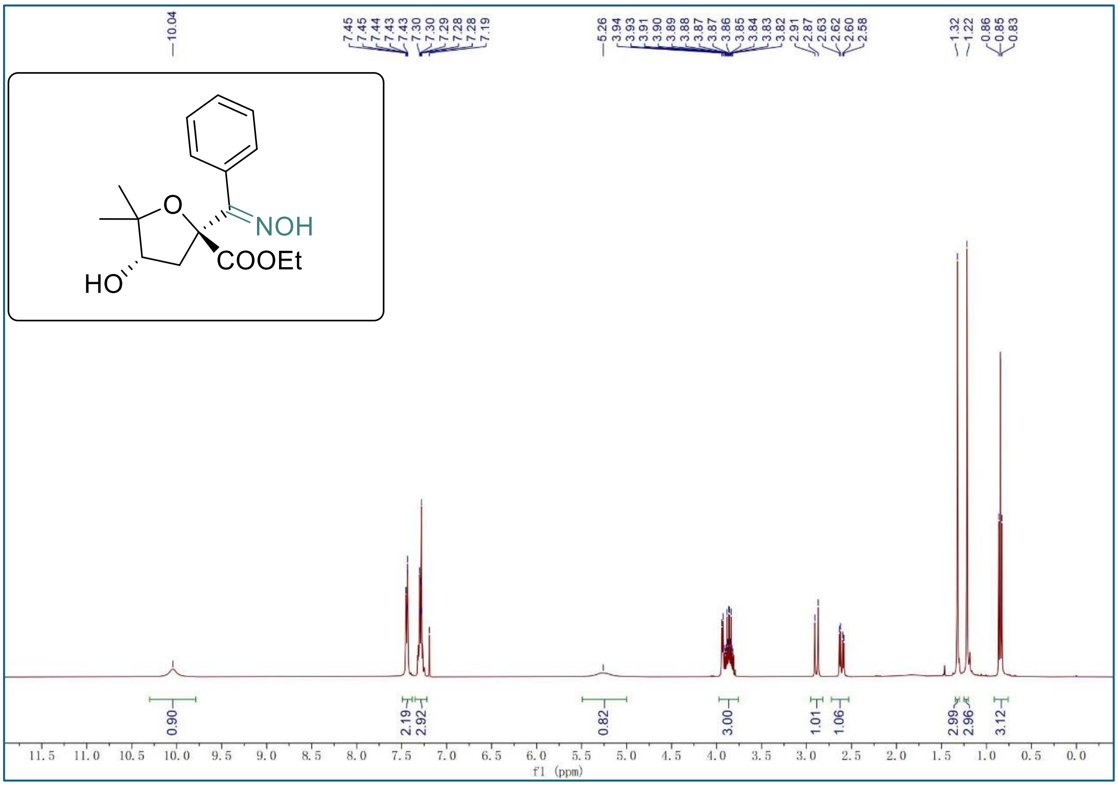

^{1}H NMR-spectrum (400 MHz, Chloroform-*d*) of **96**

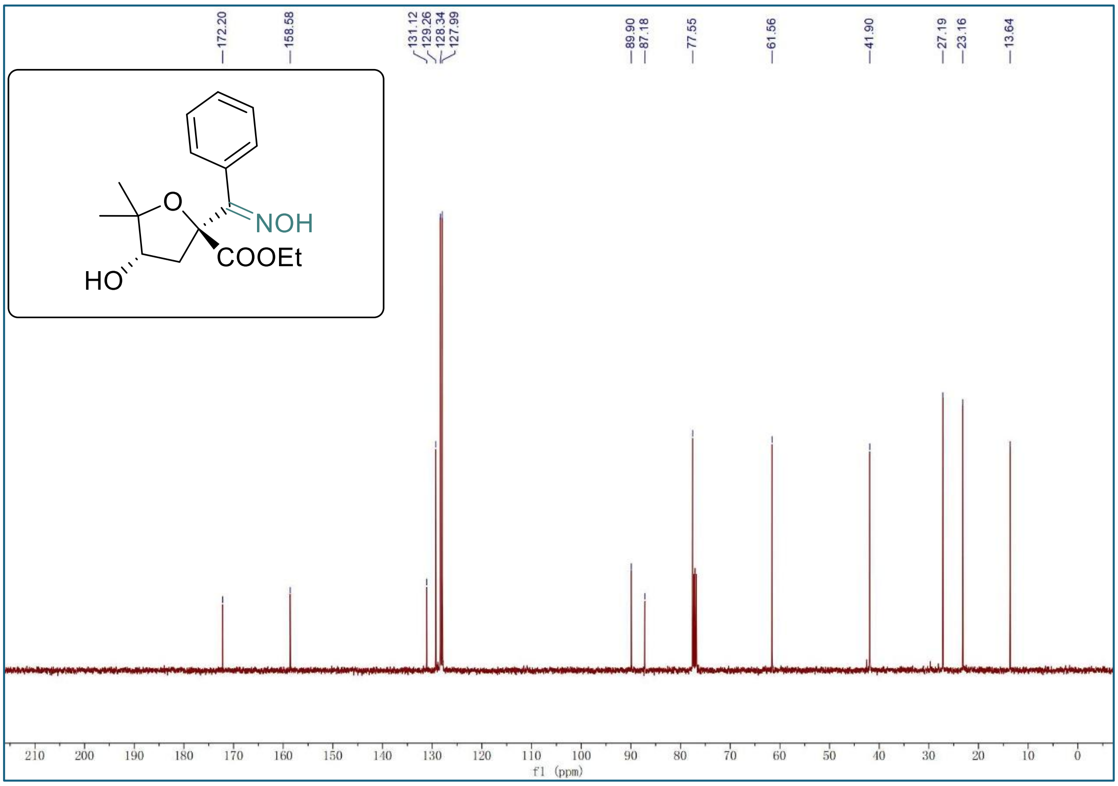

^{13}C NMR-spectrum (126 MHz, Chloroform-*d*) of **96**

diethyl 2,3-bis((2,2,6,6-tetramethylpiperidin-1-yl)oxy)maleate (98)

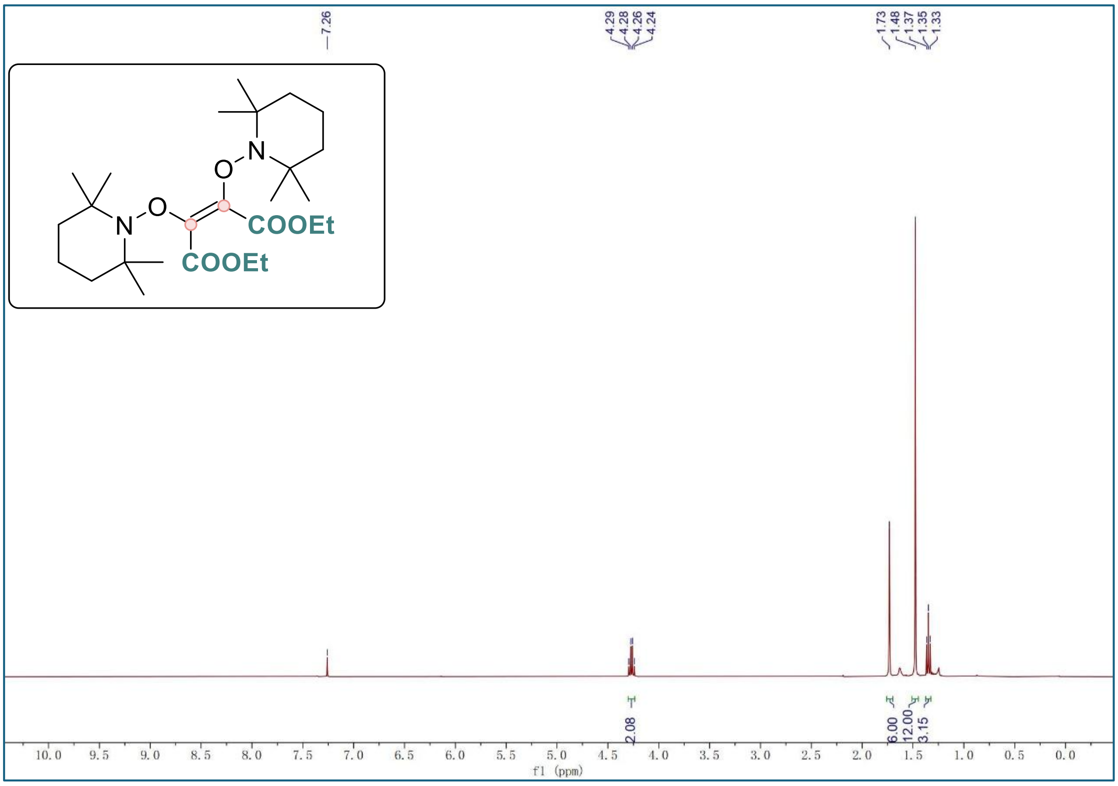

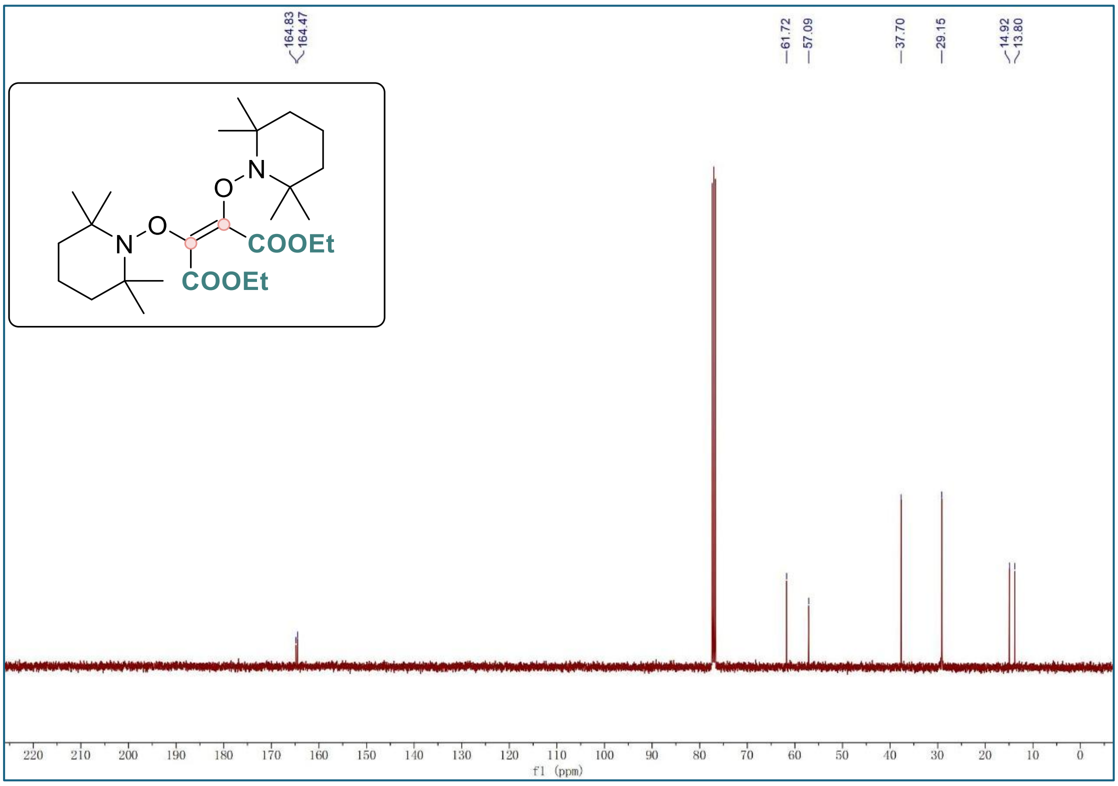

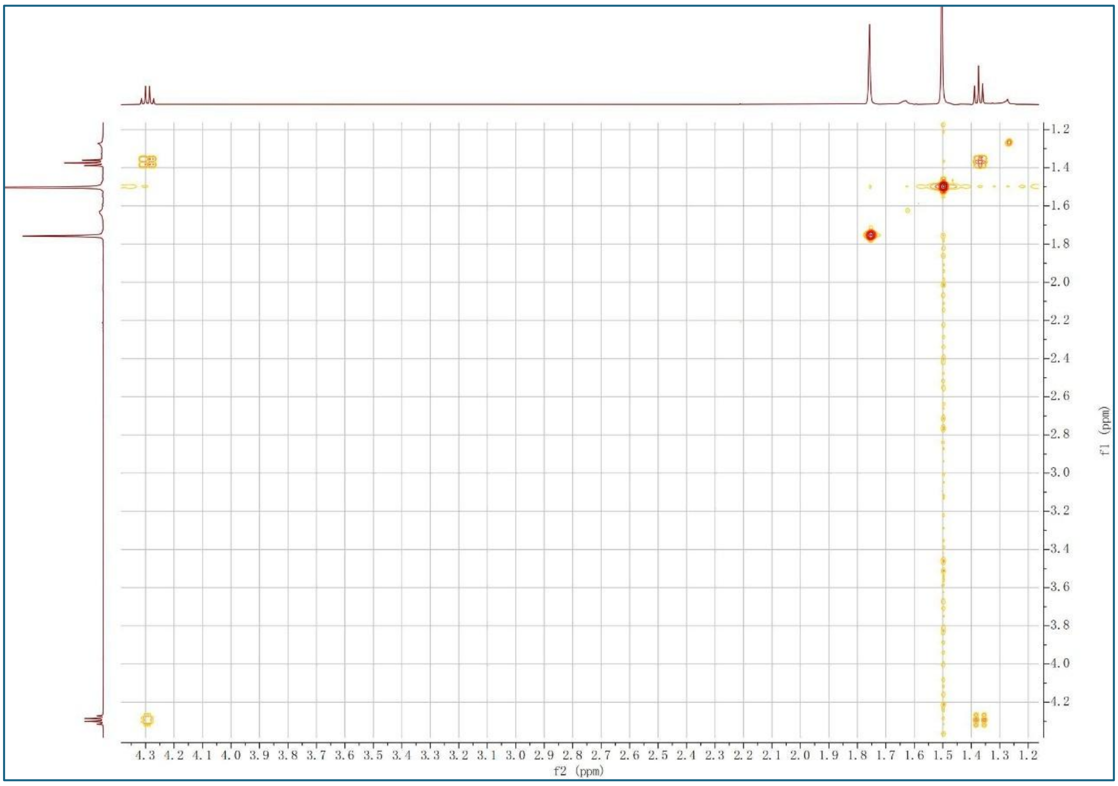

COSY of **98**

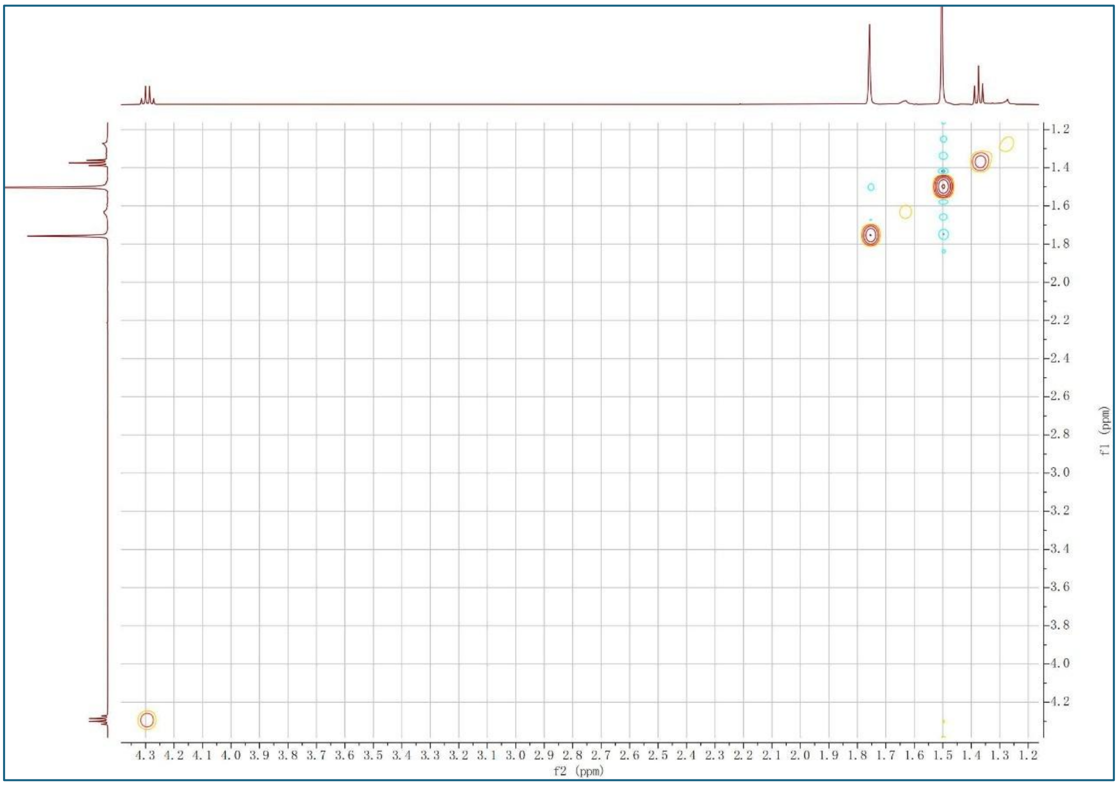

NOESY of **98**

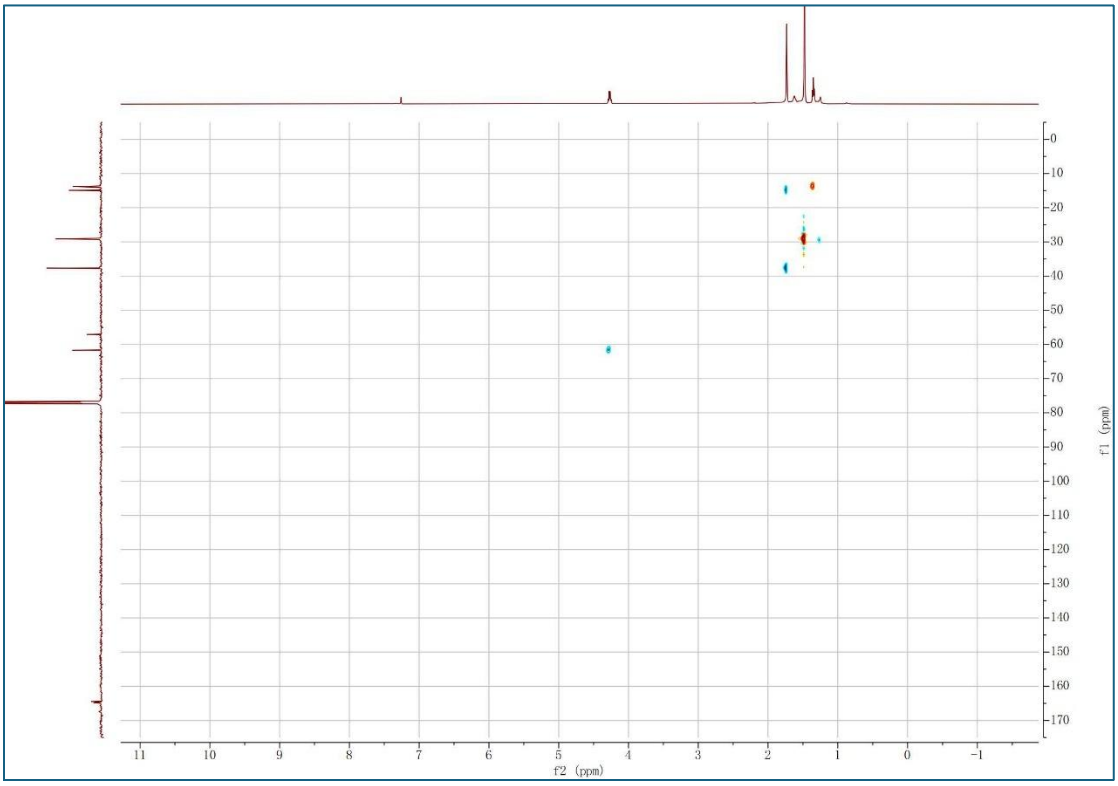

HSQC of **98**

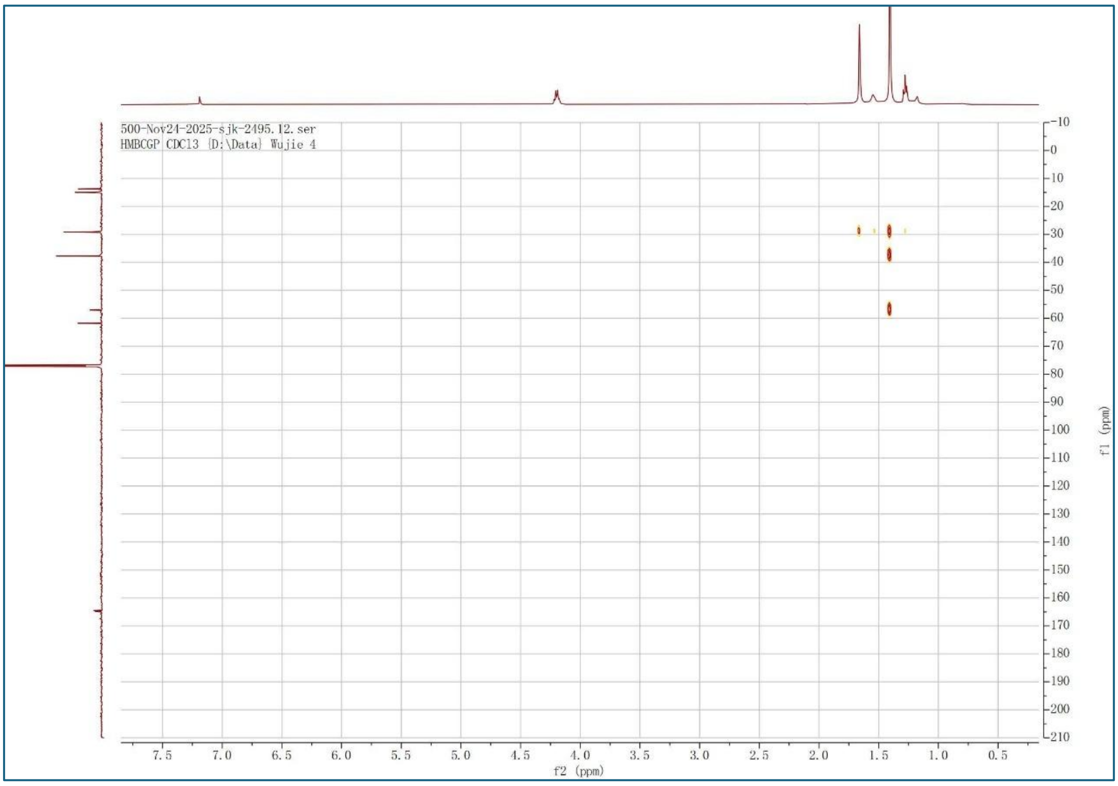

HMBC of **98**

SUPPORTING INFORMATION

ethyl 2-benzoyl-4-hydroxy-1,9-dioxaspiro[5.5]undecane-2-carboxylate (102)

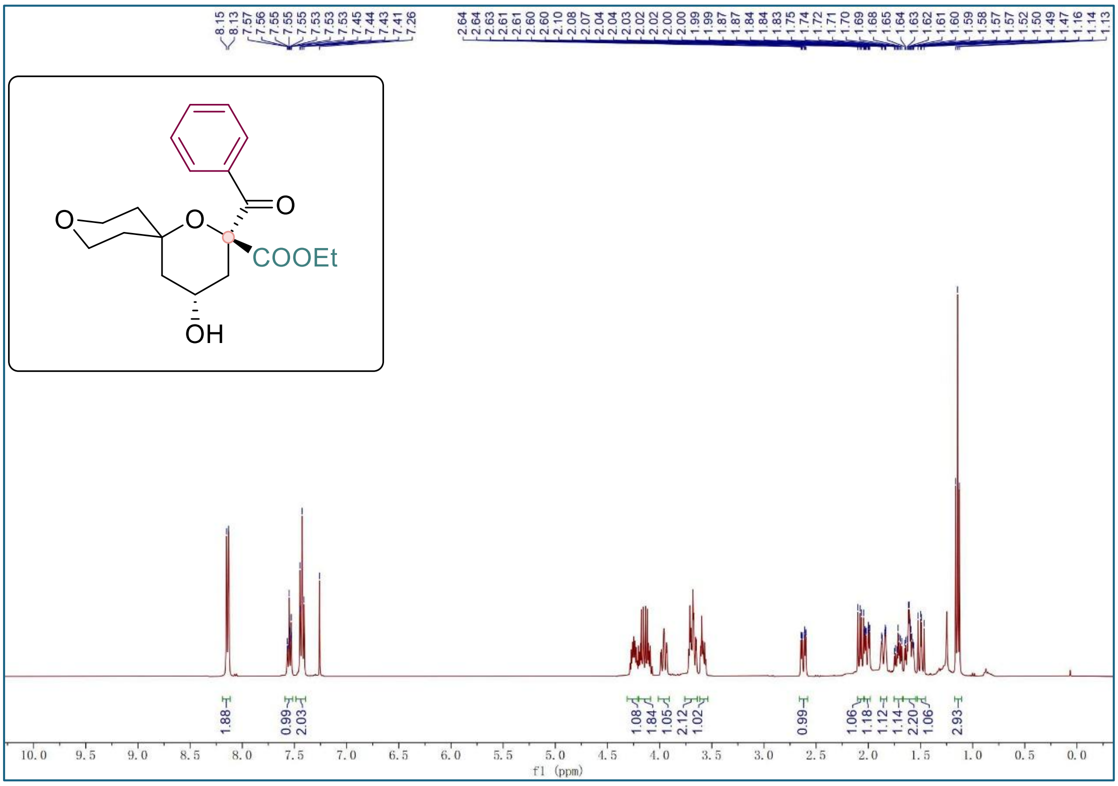

^{1}H NMR-spectrum (400 MHz, Chloroform-*d*) of **102**

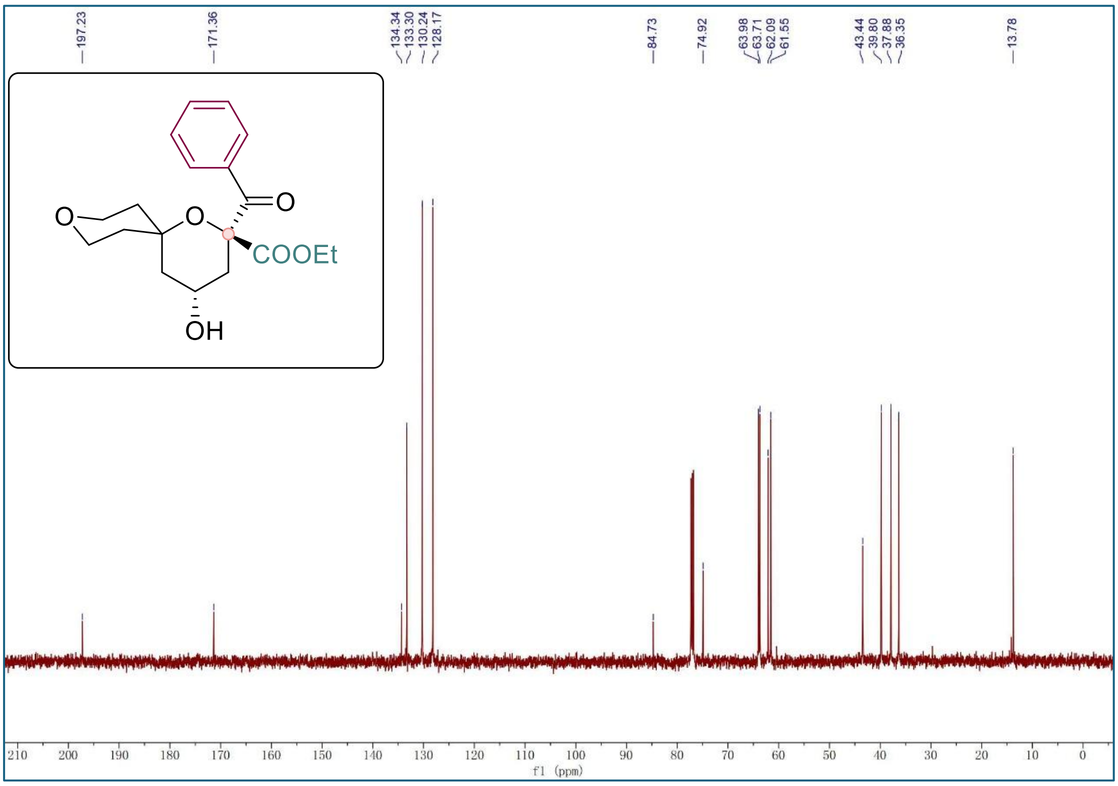

^{13}C NMR-spectrum (126 MHz, Chloroform-*d*) of **102**

cyclopropyl(2-((ethylperoxy)-λ^2-methyl)-4-hydroxy-5,5-dimethyltetrahydrofuran-2-yl)methanone (104)

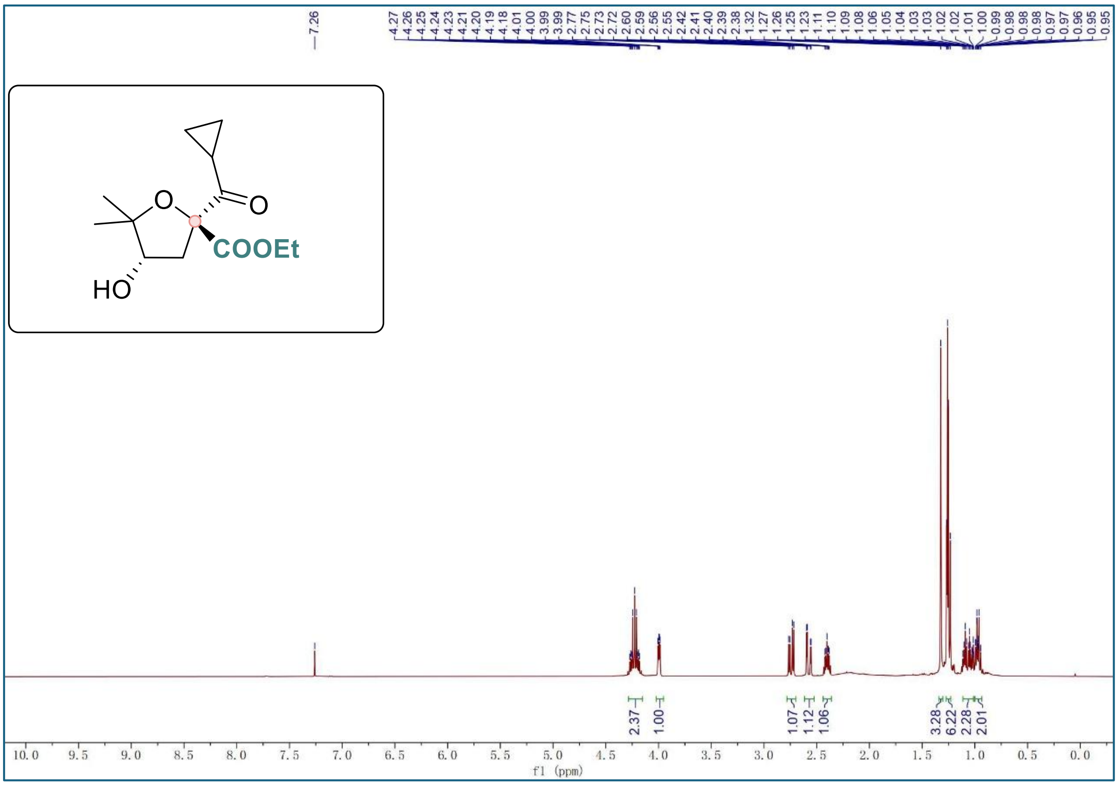

^{1}H NMR-spectrum (400 MHz, Chloroform-*d*) of **104**

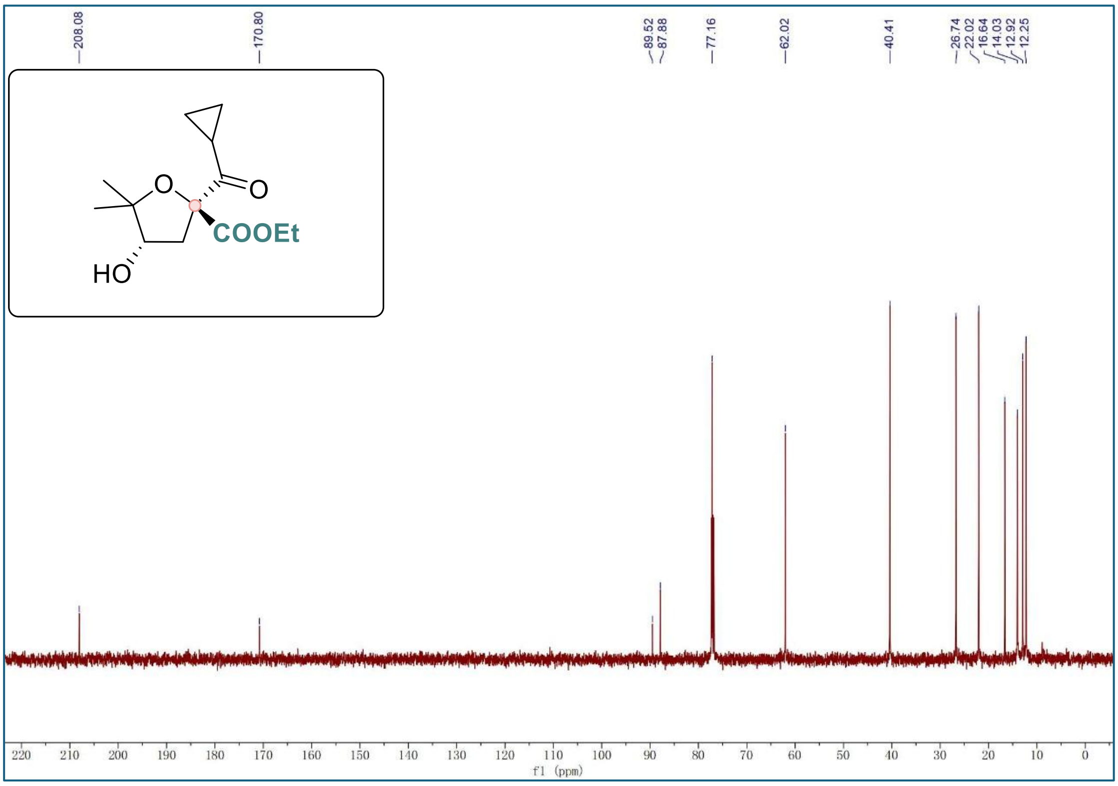

^{13}C NMR-spectrum (126 MHz, Chloroform-*d*) of **104**

SUPPORTING INFORMATION

(2-((ethylperoxy)-l2-methyl)-4-hydroxy-4-methyl-1,8-dioxaspiro[4.5]decan-2-yl)(phenyl)methanone oxime (108)

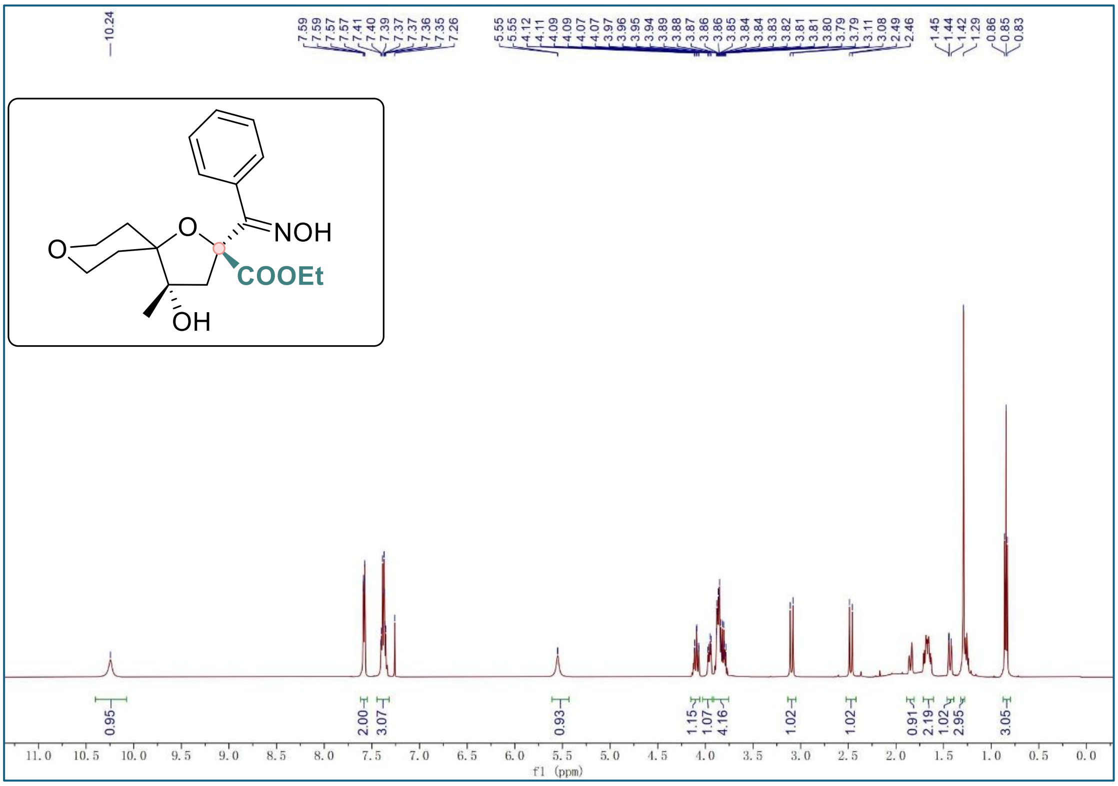

^{1}H NMR-spectrum (500 MHz, Chloroform-*d*) of **108**

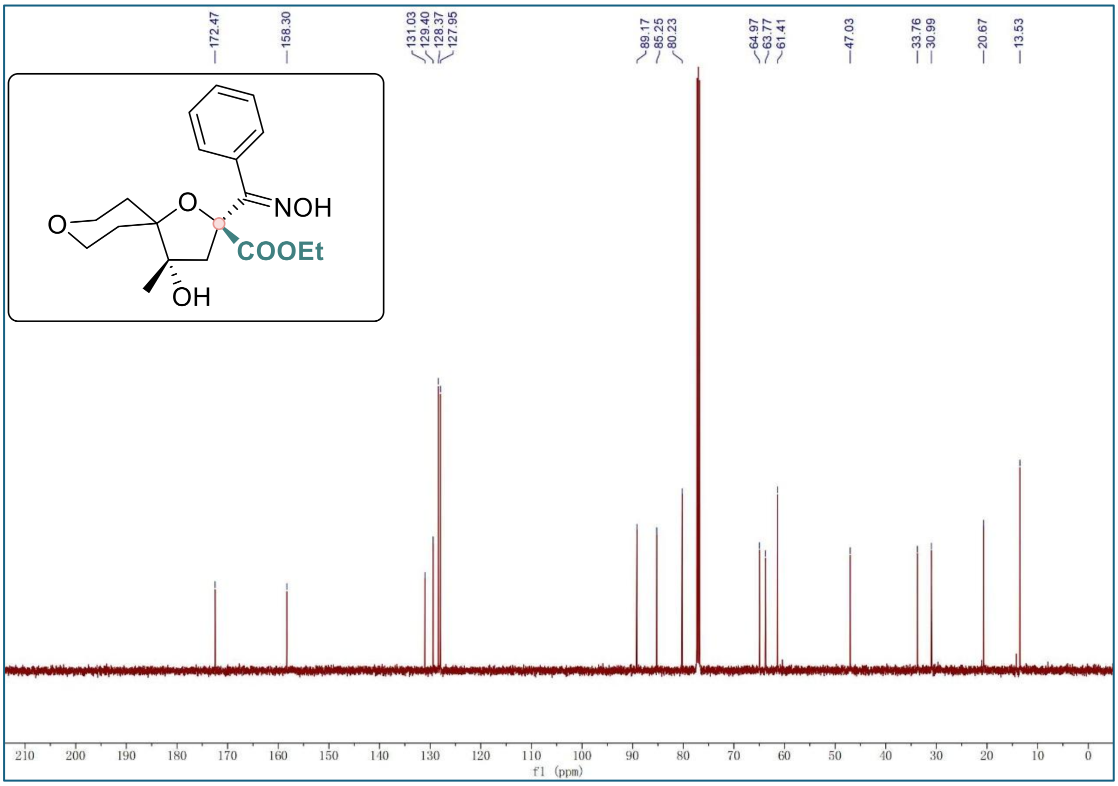

^{13}C NMR-spectrum (126 MHz, Chloroform-*d*) of **108**

ethyl 2-diazo-3-(4,4-dimethyl-2-phenyl-4,5-dihydrooxazol-5-yl)propanoate (110)

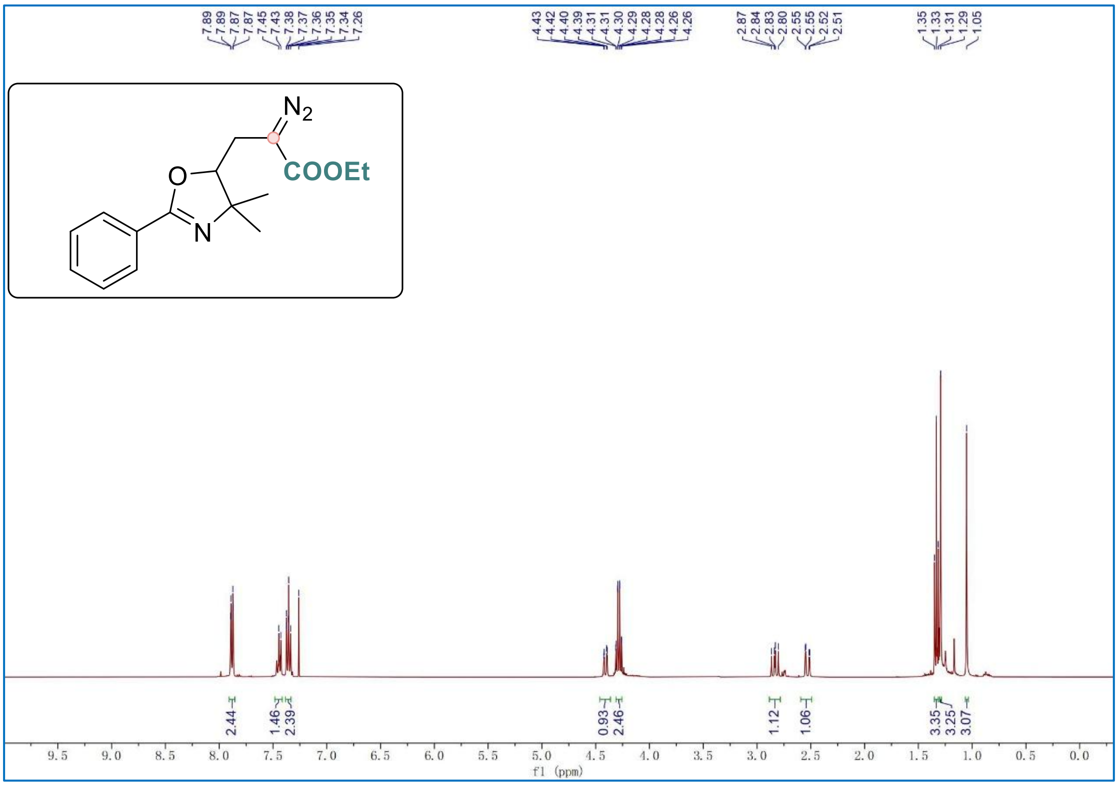

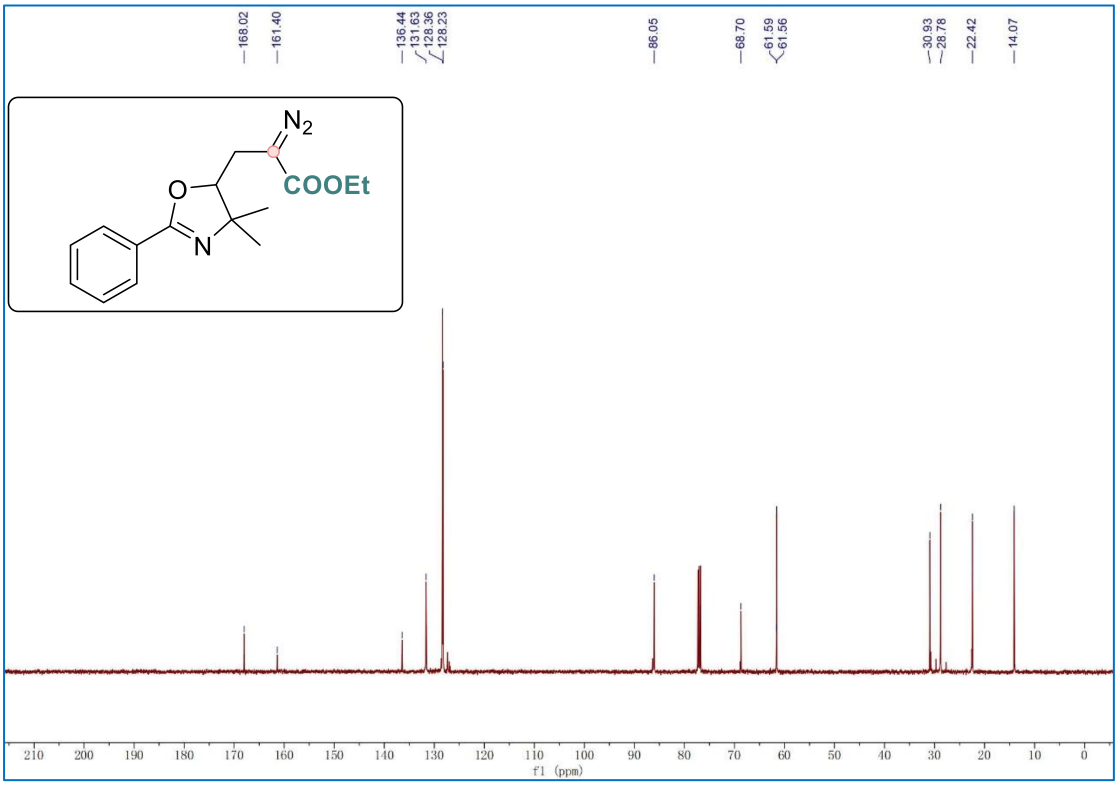

Supplement: Supplementary file 1 — Supporting File 1: anie71868‐sup‐0001‐SuppMat.pdf. [file ANIE-65-e1489528-s001.pdf]
